# Supplementary material for: Coordination between ESCRT function and Rab conversion during endosome maturation
Source: EMBO J. 2025 Feb 5;44(6):1574–607. doi: 10.1038/s44318-025-00367-7 (PMC11914609; doi:10.1038/s44318-025-00367-7)
Supplement: Supplementary file 1 — Appendix [file 44318_2025_367_MOESM1_ESM.pdf]

Appendix

Coordination between ESCRT function and Rab conversion during endosome maturation

Daniel P. Ott, Samit Desai, Jachen A. Solinger, Andres Kaech, and Anne Spang

Corresponding Author: Anne Spang

Table of Contents

Appendix Figure S1. The ESCRT machinery in *C. elegans* at a glance 3-4

Appendix Figure S2. Model of a TEM ultra-thin section *C. elegans* specimen. 5

Appendix Figure S3. The reduction of the UBQ levels affects the abundance of hTfR in *C. elegans* intestinal cells and the depletion of UBQ together with HGRS-1 causes a severe gut phenotype for RAB-5 and RABX-5. 6

Appendix Table S1. Overview of the ESCRT components in *C. elegans* and *H. sapiens* 7

Appendix Table S2. ESCRT screen growth phenotypes in WT and *sand-1(KO)* background (main table) 8

Appendix Table S3. ESCRT screen growth phenotypes in *sand-1(KO)* background (supporting table) 9

Appendix Table S4. ESCRT screen intestinal phenotypes (WT background) 10

Appendix Table S5. ESCRT screen intestinal phenotypes (*sand-1(KO)* background) 11

Appendix Table S6. Knockdowns of *vps-39* and *usp-50* are lethal in *sand-1(KO)* background (RAB-5 and RAB-7 expression) 12

Appendix Table S7. RAB-7 overexpression does not prevent the effects of *hgrs-1* and *vps-39* knockdowns on the viability in *sand-1(KO)* background (LMP-1 and RAB-7 expression) 13

Appendix Table S8. The knockdown of *rabx-5* shows no growth phenotype in *sand-1(KO)* background (RAB-5 and RAB-7 expression) 14

Appendix Table S9. Intestinal phenotypes caused by ubiquitin depletion and *usp-50* knockdown in WT background (RAB-5 and RAB-7 expression) 15

Appendix Table S10. Intestinal phenotypes caused by ubiquitin depletion and *usp-50* knockdown in WT background (RAB-5 and UBQ expression) 16

Appendix Table S11. *C. elegans* strains 17

Appendix Table S12. *H. sapiens* cell lines 18

Appendix Table S13. Primer sequences (Cells) 19

Appendix Table S14. Plasmids (Cells) 20

Appendix Table S15. Primer sequences (*C. elegans*) 21

Appendix Table S16. Antibodies (*C. elegans* and cells) 22

Appendix Table S17. Software compilation 23

Appendix Supplementary Methods 24-25

- Epon-Araldite protocol 24
- Reynold's lead citrate protocol 25

Appendix References 26

Appendix Figures

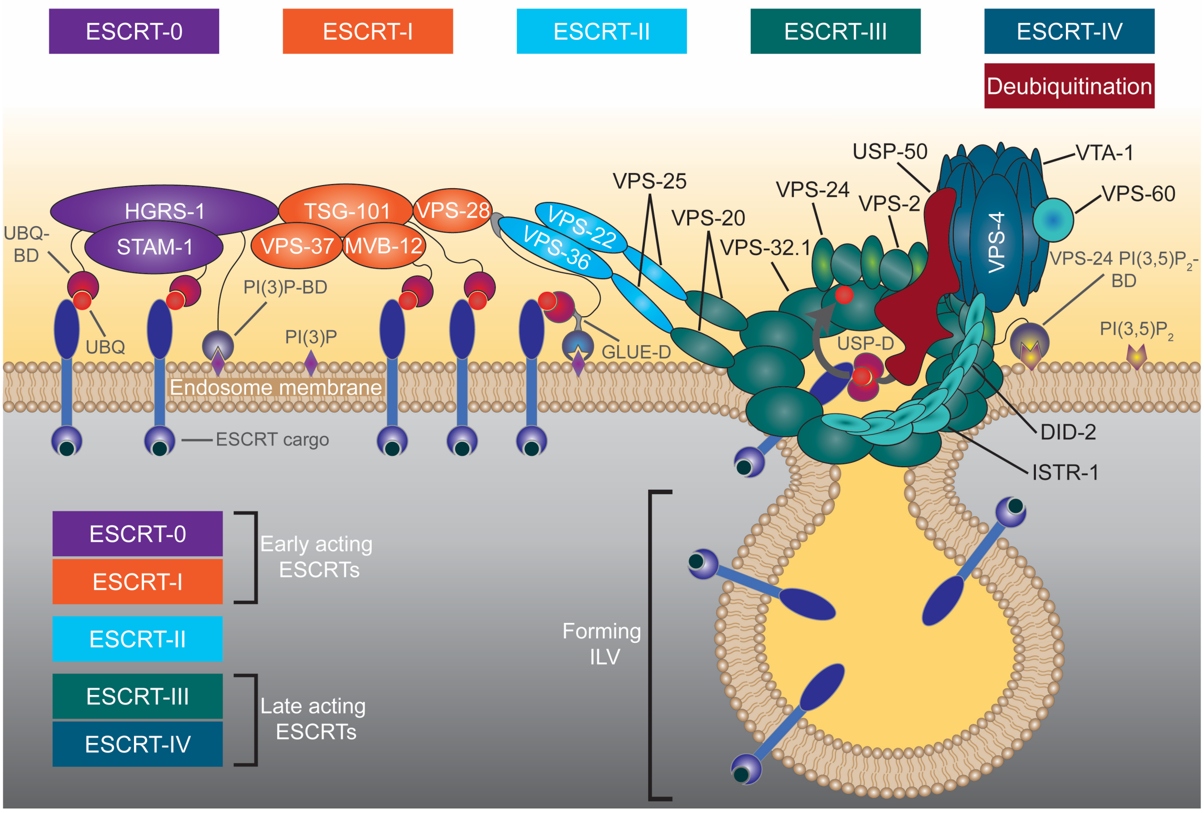

Appendix Figure S1. The ESCRT machinery in *C. elegans* at a glance, Related to Fig. 1 and Fig. EV1.

Overview model of the simplified endosomal sorting complexes required for transport (ESCRT) machinery in *C. elegans* forming an ILV. This machinery consists of five distinct complexes (0-IV) which are depicted with their individual factors. The shown ESCRT factors are color coded with respect to the complex they belong to. The sequential ESCRT machinery assembly on the endosome starts with the recruitment of the first early acting ESCRT, ESCRT-0 (purple) which is composed of HGRS-1 and STAM-1. This complex binds to the ESCRT cargo (blue) which is marked via ubiquitin (UBQ) (red) through the UBQ binding domains (UBQ-BD) (crimson red) of HGRS-1 and STAM-1. In addition ESCRT-0 also interacts with PI(3)P (violet/raspberry) through the PI(3)P binding domain (PI(3)P-BD (dark violet/white) of HGRS-1. After binding ESCRT-0 starts to concentrate the cargo on the endosome to prepare the ILV formation and recruits the early acting ESCRT, ESCRT-I (orange). ESCRT-I consists of TSG-101, VPS-37, MVB-12 and VPS-28 and interacts with the cargo through the UBQ-BDs of TSG-101 and MVB-12. Moreover, the complex can interact with several PIPs through the MVB12-associated β-prism (MABP) domain of MVB-12 (not shown for simplification). This complex further concentrates the cargo and supports the membrane remodeling performed by ESCRT-III (dark teal) together with ESCRT-II (slight blue). ESCRT-II consists of VPS-36, VPS-22 and VPS-25 and gets recruited via ESCRT-I. The complex binds to the ubiquitinated cargo and several PIPs via the GRAM-like UBQ-binding in EAP45 (GLUE) domain (GLUE-D) of VPS-36. After binding to the cargo ESCRT-II is involved in the recruitment of the late acting ESCRT, ESCRT-III, and supports the transfer of cargos from early acting ESCRTs to late acting ESCRTs. The filament forming ESCRT-III is the main driver of membrane deformation during ILV formation and its stepwise filament assembly and disassembly is highly orchestrated. The assembly cascade starts with the VPS-20, VPS-32.1 filament formation and is followed by the formation of filaments of VPS-24, VPS-2 and the ESCRT-III additional factors (light teal) DID-2 and ISTR-1. During this formation sequence the cargo gets internalized into the ILV and deubiquitinated by USP-50 (dark red) via its UBQ specific protease domain (USP-D). Moreover, during this assembly cascade ESCRT-III factors recruit the late acting ESCRT, ESCRT-IV (ocean blue), which

consists of the type I AAA-ATPase VPS-4 and VTA-1. This complex is required for the remodeling of the ESCRT-III filaments and can also disassemble them to regulate the cascade. In which way ESCRT-IV acts on ESCRT-III is regulated via the activity of VPS-4 which is controlled via several core and additional ESCRT-III factors (VPS-2, DID-2, VPS-60 and ISTR-1) and VTA-1. Finally, ESCRT-IV disassembles the last ESCRT-III filaments which causes the pinch off of the ILV from the endosomal membrane (Cullen & Steinberg, 2018; Henne *et al*, 2011; Michelet *et al*, 2010; Pfitzner *et al*, 2020; Schmidt & Teis, 2012; Schuh & Audhya, 2014).

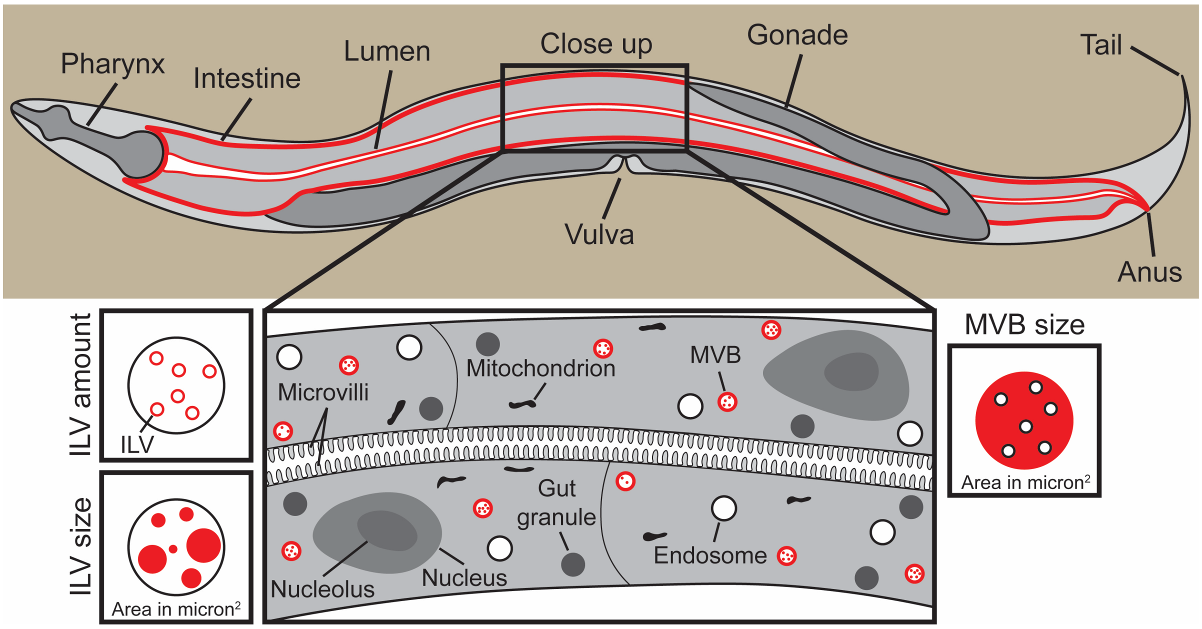

Appendix Figure S2. Model of a TEM ultra-thin section *C. elegans* specimen, Related to Fig. 2 and Fig. EV2.

Graphical illustration of an ultra-thin section *C. elegans* specimen embedded in Epon-Araldite used for TEM. The embedded worm is depicted with its pharynx, gonade vulva, anus and tail. Highlighted in red is the intestine together with the lumen it harbors. Moreover, a close up of the intestine is shown which illustrates a detailed view of the intestine and lumen in *C. elegans*. The depicted intestinal cells in the close up are shown with mitochondria, endosomes, MVBs (highlighted in red), gut granules, nuclei, nucleoli and microvilli. In this study the MVBs and ILVs were analyzed in three different ways. The three different analyzing approaches are shown schematically in the inlays next to the close up (corresponding quantifications are shown in Fig. 2B, D and F). In each inlay the part which was quantified is highlighted in red.

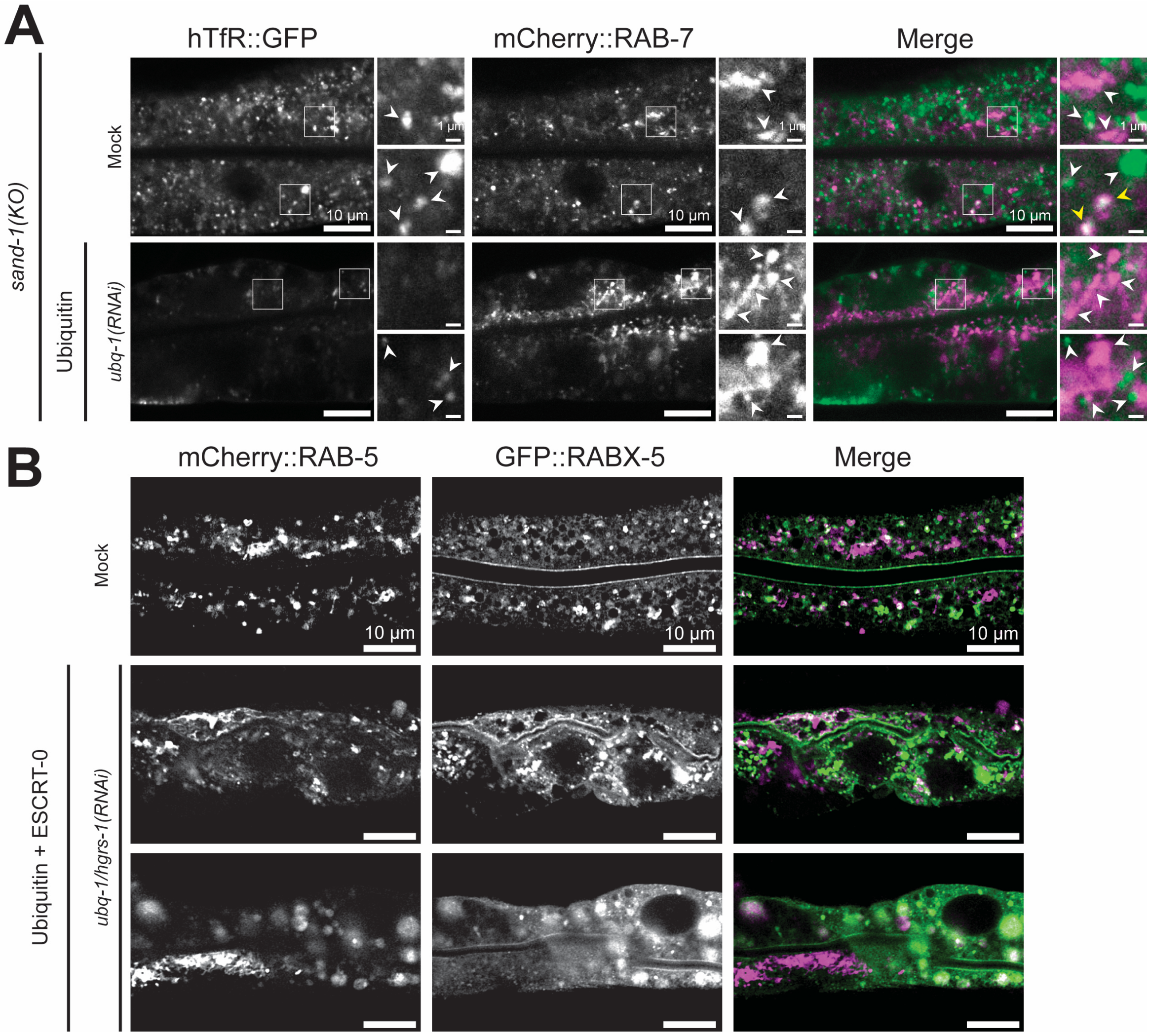

Appendix Figure S3. The reduction of the UBQ levels affects the abundance of hTfR in *C. elegans* intestinal cells and the depletion of UBQ together with HGRS-1 causes a severe gut phenotype for RAB-5 and RABX-5, Related to Fig. 5 and Fig. 7.

A. The reduction of the UBQ levels decreases the amount of hTfR::GFP in *C. elegans* intestinal cells in *sand-1(KO)* background. To visualize this effect the GFP channel is shown with the same brightness settings in all examined conditions. The mCherry channel and the merges are shown with the same settings like in Fig. 5D for comparison. White arrowheads pointing to hTfR::GFP and mCherry::RAB-7 positive structures, respectively in the individual channels. Colocalization events are marked with yellow arrowheads in the merges (Signals: hTfR > green and RAB-7 > magenta).

B. Depletion of UBQ together with the ESCRT-0 factor HGRS-1 causes a strong gut phenotype and severely affect the overall viability and health of the worm which is visible through the impaired gut morphology. mCherry::RAB-5 and GFP::RABX-5 can be found on enlarged structures individually or colocalizing in some cells (upper panel). Additionally, there are also cells exhibiting a more cytosolic GFP::RABX-5 localization in combination or without enlarged mCherry::RAB-5 accumulations (lower panel).

Data information: Merges were individually adjusted in all panels (**A** and **B**). Representative pictures with close ups (white box) on the right are shown for each experiment (scale bars: 10 µm (main pictures) and 1 µm (close ups)) (**A**). In (**B**) two example pictures of the same double knockdown are shown to illustrate the diversity of the observed phenotypes (scale bars: 10 µm). Unprocessed images are available as source data for (**A**) and (**B**).

Appendix Tables

Appendix Table S1. **Overview of the ESCRT components in *C. elegans* and *H. sapiens***

	C. elegans [a]			*H. sapiens* [b]		
**ESCRT**	**Sequence**	**Gene**	**Other names**	**UniProtKB**	**Gene**	**Synonyms**
0	C07G1.5	*hgrs-1*	CELE_C07G1.5, *pqn-9*, *vps-27*	O14964	*HGS*	*HRS*
	C34G6.7	*stam-1*	CELE_C34G6.7, *pqn-19*	Q92783; O75886	*STAM*; *STAM2*	*STAM1*; *HBP*
I	C09G12.9	*tsg-101*	CELE_C09G12.9	Q99816	*TSG101*	-
	CD4.4	*vps-37*	CELE_CD4.4	Q8NEZ2; Q9H9H4; A5D8V6; Q86XT2	*VPS37A*; *B*; *C*; *D*	*HCRP1*; -; *PML39*; *WBSCR24*
	C06A6.3	*mvb-12*	CELE_C06A6.3	Q96EY5; Q9H7P6	*MVB12A*; *MVB12B*	*CFBP*, *FAM125A*; *C9orf28*, *FAM125B*
	Y87G2A.10	*vps-28*	CELE_Y87G2A.10	Q9UK41	*VPS28*	-
II	F17C11.8	*vps-36*	CELE_F17C11.8, *tag-318*	Q86VN1	*VPS36*	*C13orf9*, *EAP45*
	C27F2.5	*vps-22*	CELE_C27F2.5	Q96H20	*SNF8*	*EAP30*
	W02A11.2	*vps-25*	CELE_W02A11.2	Q9BRG1	*VPS25*	*DERP9*, *EAP20*
III	Y65B4A.3	*vps-20*	CELE_ Y65B4A.3, Y65B4A.d, Y65B4A.h	Q96FZ7	*CHMP6*	*VPS20*
	C56C10.3	*vps-32.1*	CELE_C56C10.3, *phi-27*, *tag-309*	Q9BY43; Q9H444; Q96CF2	*CHMP4A*; *B*; *C*	*C14orf123*, *SHAX2*; *C20orf178*, *SHAX1*; *SHAX3*
	T27F7.1	*vps-24*	CELE_T27F7.1	Q9Y3E7	*CHMP3*	*CGI149*, *NEDF*, *VPS24*
	Y46G5A.12	*vps-2*	CELE_Y46G5A.12	O43633; Q9UQN3	*CHMP2A*; *CHMP2B*	*BC2*, *CHMP2*; -
III add.	F23C8.6	*did-2*	CELE_F23C8.6, *phi-24*	Q9HD42; Q7LBR1	*CHMP1A*; *CHMP1B*	*CHMP1*, *KIAA0047*, *PCOLN3*, *PRSM1*; *C18orf2*
	F41E6.9	*vps-60*	CELE_F41E6.9	Q9NZZ3	*CHMP5*	*C9orf83*, *SNF7DC2*
	K10C8.3	*istr-1*	CELE_K10C8.3	P53990	*IST1*	*KIAA0174*
	T24B8.2	*chmp-7*	CELE_T24B8.2	Q8WUX9	*CHMP7*	-
	Y74C10AL.2	*Y74C10AL.2*	CELE_Y74C10AL.2, Y74C10AL.a	O95807; P56557	*TMEM50A*; *TMEM50B*	*SMP1*; *C21orf4*
IV	Y34D9A.10	*vps-4*	CELE_Y34D9A.10, CELE_Y34D9A.b, *phi-25*	Q9UN37; O75351	*VPS4A*; *VPS4B*	*VPS4*; *SKD1*, *VPS42*
	T23G11.7	*T23G11.7*	CELE_T23G11.7, *vta-1*	Q9NP79	*VTA1*	*C60orf55*
DUB	E01B7.1	*usp-50*	CELE_E01B7.1, Y59A8B.b, Y59A8B.a, *phi-33*	P40818	*USP8*	*KIAA0055*, *UBPY*
Assoc.	R10E12.1	*alx-1*	CELE_R10E12.1, YNK1, *pqn-58*	Q8WUM4	*PDCD6IP*	*AIP1*, *ALIX*, *KIAA1375*
	Y53H1C.2	*ego-2*	CELE_Y53H1C.2	Q9H3S7	*PTPN23*	*KIAA1471*

[a]: Nomenclature of *C. elegans* genes in accordance to Wormbase (https: www.wormbase.org)
[b]: Nomenclature of *H. sapiens* genes in accordance to UniProt (https: www.uniprot.org)

Appendix Table S2. **ESCRT screen growth phenotypes in WT and *sand-1(KO)* background (main table)**

		**Strain background**	
		WT	*sand-1(ok1963)IV*
**ESCRT**	**RNAi**	**Phenotype**	
	Control	+	+
0	*hgrs-1**	D	D/DA
I	*tsg-101*	+	+
	vps-37	D/DA/Ro	D/Ro
	vps-28	+	+
III	*vps-20*	D/DA	D
	*vps-32.1**	Let/D/Ro	Let/D/DA
	vps-24	+	+
	*vps-2**	D/DA	D
	*did-2**	D/DA	Let/D/DA
	vps-60	D	D
IV	*vps-4**	Ro/D/DA/Let	D/DA

+: Growth/development unaffected
Let: Lethal
D: Developmental delay
DA: Developmental arrest
Ro: Reduced offspring
*: Pre-feeding till L3 with Control RNAi in *sand-1(KO)* background

Appendix Table S3. **ESCRT screen growth phenotypes in *sand-1(KO)* background (supporting table)**

		Strain background
		sand-1(ok1963)IV
**ESCRT**	**RNAi**	**Phenotype**
0	*hgrs-1*	Ste/Ro/D
III	*vps-32.1*	Ste/Let
	vps-2	Ro/Let/D/DA
	did-2	Ro/Let/D/DA
IV	*vps-4*	Ro/Let/D/DA

Let: Lethal
D: Developmental delay
DA: Developmental arrest
Ste: Sterile
Ro: Reduced offspring

Appendix Table S4. **ESCRT screen intestinal phenotypes (WT background)**

RNAi	RAB-5 distribution	RAB-7 distribution	RAB-5 structure size	RAB-7 structure size	RAB-5 and RAB-7 colocalization structures	RAB-5 and RAB-7 colocalization aggregates
Control	⁝⁝⁝ and __	•••	.	.	X	NP
hgrs-1	••• and ⁝⁝⁝	⁖ and •••	. and o	. and o	+	+
tsg-101	••• and __	⁖ and •••	o and O	. and o	++	++
vps-37	••• and __	⁖ and •••	o and O	. and o	++	++
vps-28	••• and __	⁖ and •••	o and O	o and O	+++	+++
vps-20	••• and ☁	⁖ and •••	. and O	o and O	+++	++
vps-32.1	—	⁖	. and o	. and o	++++	+++
vps-24	☁ and __	⁖ and ☁	. and O	o and O	+++	+++
vps-2	—	⁖	. and o	. and o	++++	+++
did-2	••• and ☁	⁖ and ☁	. and O	o and O	+++	+++
vps-60	••• and ⁝⁝⁝	•••	. and o	. and o	+	+
vps-4	—	⁖	. and O	. and o	++++	+++

RAB-5 distribution: ⁝⁝⁝ > accumulation in two apical stripes, ••• > accumulation in a region more basal, __ > distributed in the whole cell, ☁ > cloud like accumulation with distinct structures inside
RAB-7 distribution: ••• > accumulation in a region more basal, ⁖ > dispersed pattern, ☁ > cloud like accumulation with distinct structures inside
RAB-5 structure size: . > small, o > enlarged, O > strong enlarged
RAB-7 structure size: . > small, o > enlarged, O > strong enlarged
RAB-5/RAB-7 colocalization (structures): X > baseline, + > slight increased, ++ > increased, +++ > strong increased, ++++ > very strong increased
RAB-5/RAB-7 colocalization (aggregates): NP > Not present, + > slight increased, ++ > increased, +++ > strong increased

Appendix Table S5. **ESCRT screen intestinal phenotypes (*sand-1(KO)* background*)**

**RNAi**	**RAB-5 distribution**	**RAB-7 distribution**	**RAB-5 structure size**	**RAB-7 structure size**	**RAB-5 and RAB-7 colocalization structures**	**RAB-5 and RAB-7 colocalization aggregates**
Control	☁ and __	∴ and ☁	o and O	. and O	X	+++
hgrs-1	⠿ and ☁	∴	. and O	. and O	U	++
tsg-101	⠿ and __	∴ and ⠿	. and o	. and O	U	++
vps-37	⠿ and __	∴ and ⠿	. and o	. and O	U	++
vps-28	⠿ and __	∴ and ⣿	. and o	. and O	U	++
vps-20	⠿ and ☁	∴ and ☁	. and O	. and O	U	+++
vps-32.1	☁ and __	∴ and ☁	. and O	. and O	+/U	+++
vps-24	⠿ and ☁	∴ and ☁	. and o	. and O	U	++
vps-2	⣿ and ☁	∴ and ☁	. and o	. and O	U	+++
did-2	⠿ and ☁	∴ and ☁	. and o	. and O	+/U	+++
vps-60	⠿ and __	∴ and ⣿	. and o	. and O	U	++
vps-4	☁ and __	∴ and ☁	. and O	. and O	+	+++

RAB-5 distribution: ⠿ > accumulation close to the gut lumen, ⣿ > broader accumulation in a region more basal, __ > distributed in the whole cell, ☁ > cloud like accumulation with distinct structures inside

RAB-7 distribution: ⠿ > accumulation close to the gut lumen, ∴ > dispersed pattern, ☁ > cloud like accumulation with distinct structures inside

RAB-5 structure size: . > small, o > medium, O > big

RAB-7 structure size: . > small, O > big

RAB-5/RAB-7 colocalization (structures): X > baseline, U > unchanged, + > weak colocalization

RAB-5/RAB-7 colocalization (aggregates): + > weak colocalization, ++ > colocalization, +++ > strong colocalization

Red highlighted : L3 pre-feeding

*: The worms which were used for the experiments shown in this table carried the *sand-1(ok1963)IV* deletion.

Appendix Table S6. **Knockdowns of *vps-39* and *usp-50* are lethal in *sand-1(KO)* background (RAB-5 and RAB-7 expression)**

		**Strain background**	
		WT	*sand-1(ok1963)IV*
		**Marker expression**	
		GFP::RAB-5 and mCherry::RAB-7	
**Complex**	**RNAi**	**Phenotype**	
	Control	+	+
	*ubq-1**	Let	Let
	ubq-1 [a] (1:250)	D/DA	+
Deubiquitination	*usp-50*	D/DA	D/DA/Ro/Let
HOPS	*vps-39*	ND	D/DA/Ro/Emb/Let
	ubq-1:vps-39 [a] (1:250)	ND	D/DA/Ro/Emb

+: Growth/development unaffected
Let: Lethal
Emb: Embryonic lethal
D: Developmental delay
DA: Developmental arrest
Ro: Reduced offspring
ND: Not determined
*: *ubq-1* RNAi without dilution
[a]: Diluted *ubq-1* RNAi

Appendix Table S7. **RAB-7 overexpression does not prevent the effects of *hgrs-1* and *vps-39* knockdowns on the viability in *sand-1(KO)* background (LMP-1 and RAB-7 expression)**

		**Strain background**	
		sand-1(ok1963)IV	
		**Marker expression**	
		mCherry::RAB-7, LMP-1::GFP	mCherry::RAB-7, hTfR::GFP
**Complex**	**RNAi**	**Phenotype**	
	Control	+	+
	*ubq-1**	Let	Let
	ubq-1 [a] (1:250)	+	D/DA/Ro
ESCRT-0	*hgrs-1*	D/DA/Ste	Let/Emb/DA
HOPS	*vps-39*	D/DA/Emb	ND
	ubq-1:vps-39 [a] (1:250)	D/DA/Emb	ND

+: Growth/development unaffected
Let: Lethal
Emb: Embryonic lethal
D: Developmental delay
DA: Developmental arrest
Ste: Sterile
Ro: Reduced offspring
ND: Not determined
*: *ubq-1* RNAi without dilution
[a]: Diluted *ubq-1* RNAi

Appendix Table S8. **The knockdown of *rabx-5* shows no growth phenotype in *sand-1(KO)* background (RAB-5 and RAB-7 expression)**

		**Strain background**
		sand-1(ok1963)IV
		**Marker expression**
		GFP::RAB-5 and mCherry::RAB-7
**Function**	**RNAi**	**Phenotype**
	Control	+
RAB-5 GEF	*rabx-5*	+

+: Growth/development unaffected

Appendix Table S9. **Intestinal phenotypes caused by ubiquitin depletion and *usp-50* knockdown in WT background (RAB-5 and RAB-7 expression)**

**RNAi**	**RAB-5 distribution**	**RAB-5 structure size**	**RAB-5/RAB-7 structure amount**	**RAB-7 distribution**	**RAB-5 and RAB-7 colocalization**
Control	⁝⁝⁝ and __	.	X	⁝⁝⁝	+
ubq-1 (1:250) [a]	—	.	---/-	⁝⁝⁝ and __	N.D.
usp-50	✸ and __	. and o	N.D.	•⁚⁚ and ✸	+++

RAB-5 distribution: ⁝⁝⁝ > accumulation in two apical stripes, __ > distributed in the whole cell, ✸ > aggregate formation
RAB-5 structure size: . > small, o > enlarged
RAB-5/RAB-7 structure amount: X > baseline, - > reduced, --- > strongly reduced, N.D. > not determined
RAB-7 distribution: ⁝⁝⁝ > accumulation in two more basal stripes, __ > distributed in the whole cell, •⁚⁚ > dispersed pattern,
✸ > aggregate formation
RAB-5/RAB-7 colocalization: + > weak colocalization, +++ > strong colocalization, N.D. > not determined
[a]: Diluted *ubq-1* RNAi

Appendix Table S10. **Intestinal phenotypes caused by ubiquitin depletion and *usp-50* knockdown in WT background (RAB-5 and UBQ expression)**

RNAi	RAB-5 distribution	RAB-5 aggregation	UBQ structure size	UBQ abundance (nucleus)	RAB-5 and UBQ colocalization
Control	::: and __	+	.	+	+
ubq-1 (1:250) [a]	::: and __	-	.	+	N.D.
usp-50	__	+++	o and O	-	+++

RAB-5 distribution: ::: > accumulation in two apical stripes, __ > distributed in the whole cell
RAB-5 aggregation: + > small, +++ > big, - > smaller than Mock
UBQ structure size: . > small, o > enlarged, O > strong enlarged
UBQ abundance (nucleus): + > UBQ accumulates in the nucleus, - > UBQ is less abundant in the nucleus
RAB-5/UBQ colocalization: + > weak colocalization, +++ > strong colocalization, N.D. > not determined
[a]: Diluted *ubq-1* RNAi

Appendix Table S11. ***C. elegans* strains**

C. elegans	**Source**	**Identifier**
C. elegans N2 Bristol	CGC/Attila Stetak	N2
sand-1(ok1963)IV	CGC	RB1598
pwIs429[pvha-6::mCherry::rab-7]; pwIs72[pvha6::GFP::rab-5 + unc-119(+)]	Solinger JA, Spang A. Loss of the Sec1/Munc18-family proteins VPS-33.2 and VPS-33.1 bypasses a block in endosome maturation in Caenorhabditis elegans. Mol Biol Cell. 2014 Dec 1;25(24):3909-25.	FA086
sand-1(ok1963)IV; pwIs429[pvha-6::mCherry::rab-7]; pwIs72[pvha6::GFP::rab-5 + unc-119(+)]	Solinger JA, Spang A. Loss of the Sec1/Munc18-family proteins VPS-33.2 and VPS-33.1 bypasses a block in endosome maturation in Caenorhabditis elegans. Mol Biol Cell. 2014 Dec 1;25(24):3909-25.	AG Spang
sand-1(ok1963)IV; pwIs429[vha-6::mCherry::rab-7]; pwIs90[Pvha-6::hTfR-GFP; Cbr-unc-119(+)]	Solinger JA, Spang A. Loss of the Sec1/Munc18-family proteins VPS-33.2 and VPS-33.1 bypasses a block in endosome maturation in Caenorhabditis elegans. Mol Biol Cell. 2014 Dec 1;25(24):3909-25.	AG Spang
pwIs429[pvha-6::mCherry::rab-7] + pwIs50[Plmp-1::lmp-1::GFP + Cb-unc-119(+)]; sand-1(ok1963)IV	This study	AG Spang
pwIs518[vha-6::GFP-HGRS-1]; pwIs846[Pvha-6-RFP-rab-5; Cb.unc-119(+)]	This study	AG Spang
unc-119(ed3)III;pwIs429 [Pvha-6::mCherry::rab-7+Cb-unc-119]; pwIs518[vha-6::GFP-HGRS-1]	This study	AG Spang
jyEx128 [vha-6p:::GFP::UBQ, cb-unc-119(+)]; unc-119(ed3)III	Bakowski MA, Desjardins CA, Smelkinson MG, Dunbar TL, Lopez-Moyado IF, Rifkin SA, Cuomo CA, Troemel ER. Ubiquitin-mediated response to microsporidia and virus infection in C. elegans. PLoS Pathog. 2014 Jun 19;10(6):e1004200.	ERT261
unc-119(ed3)III; pwIs846[Pvha-6-RFP-rab-5; Cb.unc-119(+)]; jyEx128 [vha-6p:::GFP::UBQ, cb-unc-119(+)]; unc-119(ed3)III	This study	AG Spang
ycxIs333[Pvha-6::GFP::RABX-5]; ycxEX1259[Pvha-6::mCherry::RAB-5]	Zhang W, Wang S, Yang C, Hu C, Chen D, Luo Q, He Z, Liao Y, Yao Y, Chen J, He J, Hu J, Xia T, Lin L, Shi A. LET-502/ROCK Regulates Endocytic Recycling by Promoting Activation of RAB-5 in a Distinct Subpopulation of Sorting Endosomes. Cell Rep. 2020 Sep 22;32(12):108173.	HUS5573

Appendix Table S12. ***H. sapiens* cell lines**

**Cell lines**	**Source**	**Identifier**
HeLa CCL2	ATCC	RRID: CVCL_0030
HeLa CCL2 *HGS* knockout	This study	AG Spang
HeLa CCL2 *CHMP6* knockout	This study	AG Spang
HeLa CCL2 *CCZ1* knockout	Podinovskaia M, Prescianotto-Baschong C, Buser DP, Spang A. A novel live-cell imaging assay reveals regulation of endosome maturation. Elife. 2021 Nov 30;10:e70982.	AG Spang
HeLa CCL2 stably expression mApple-RAB5 and GFP-RAB7	Podinovskaia M, Prescianotto-Baschong C, Buser DP, Spang A. A novel live-cell imaging assay reveals regulation of endosome maturation. Elife. 2021 Nov 30;10:e70982.	AG Spang

Appendix Table S13. **Primer sequences (Cells)**

			Primer sequence	
**Function**	**Gene name**	Targeted Exons	Forward	Reverse
ESCRT-0	*HGS*	Exon1	CACCGGTAACGCGTCCCCACCCGA	AAACTCGGGTGGGGACGCGTTACC
		Exon22	CACCGTCATTTCATTCGACTGACCC	AAACGGGTCAGTCGAATGAAATGAC
ESCRT-III	*CHMP6*	Exon3	CACCGGCTCAAGAAGAAGCGATACC	AAACGGTATCGCTTCTTCTTGAGCC
		Exon 5	CACCGGATCCTGGACGAGACGCAGG	AAACCCTGCGTCTCGTCCAGGATCC

Appendix Table S14. **Plasmids (Cells)**

**Construct**	**Source**	**Identifier**
mApple Rab5	Addgene	#54944
GFP Rab7	Addgene	#12605
GFP Rab5	Addgene	#49888.
pCS2 HRS-RFP	Addgene	#29685
Rabex5 (myc-tagged)	Mattera R, Bonifacino JS. Ubiquitin binding and conjugation regulate the recruitment of Rabex-5 to early endosomes. EMBO J. 2008 Oct 8;27(19):2484-94.	N/A
Rabex5 A58D (myc-tagged)	Mattera R, Bonifacino JS. Ubiquitin binding and conjugation regulate the recruitment of Rabex-5 to early endosomes. EMBO J. 2008 Oct 8;27(19):2484-94.	N/A
Rabex5 Y25A / A58D (myc-tagged)	Mattera R, Bonifacino JS. Ubiquitin binding and conjugation regulate the recruitment of Rabex-5 to early endosomes. EMBO J. 2008 Oct 8;27(19):2484-94.	N/A

Appendix Table S15. **Primer sequences (*C. elegans*)**

		Primer sequence	
**Function**	**Gene name**	Forward	Revers
ESCRT-I	*tsg-101*	gggagaccggcagatctgatatcATGTCGGCCCACCAGGTAC	acggtatcgataagcttgatatcCTGAAGAAGTCCACTGAGATCATATCC
ESCRT-III	*vps-2*	gggagaccggcagatctgatatcATGGATTTCCTGTTCGGAC	acggtatcgataagcttgatatcTCATTCTCTTCTAAGCTGATC
	vps-60	gggagaccggcagatctgatatcATGAATCGGATTTTTGGAAC	acggtatcgataagcttgatatcATCAGGAGTATCGTACTG
RAB-5 GEF	*rabx-5*	gggagaccggcagatctgatatcAAGCTTCAACATCGGCTG	acggtatcgataagcttgatatcAGATTGTTTCATGTTCATATATTCATC

Primer parts homolog to the pDT7 vector are written in lowercase letters and primer parts homolog to the corresponding gene are written in capital letters.

Appendix Table S16. **Antibodies (*C. elegans* and Cells)**

Type and specifications	Target	Detection	Reference or Source	Catalog number	Used concentration and purpose
Primary (polyclonal, generated in rabbit)	RAB-5	Secondary AB	Poteryaev D, Fares H, Bowerman B, Spang A. Caenorhabditis elegans SAND-1 is essential for RAB-7 function in endosomal traffic. EMBO J. 2007 Jan 24;26(2):301-12.	-	1:500 (5% milk + TBS-T), western blot
Primary (polyclonal, generated in rabbit)	RAB-7	Secondary AB			1:500 (5% milk + TBS-T), western blot
Primary (monoclonal, generated with mouse cells)	α-Tubulin	Secondary AB	Merck KGaA, Germany.	T5168	1:10000 (5% milk + TBS-T), western blot
Primary (- , originated from mouse)	RAB5	Secondary AB	Gift from Martin Spiess	-	1:1000 (5% milk + TBS-T), western blot
Primary (monoclonal, generated with rabbit cells)	RAB7	Secondary AB	Cell Signaling Technology Inc., USA.	9367	1:1000 (Can Get Signal solution), western blot
Primary (monoclonal, generated with mouse cells)	C-MYC	Secondary AB	Thermo Fisher Scientific Inc., USA.	MA1-980	1:200 (5% FBS in PBS), immunofluorescence
Secondary (polyclonal, generated in goat)	Rabbit IgG (H+L)	HRP (chemiluminescence)	Thermo Fisher Scientific Inc., USA.	31460	1:10000 (TBS-T), western blot
Secondary (polyclonal, generated in goat)	Mouse IgG (H+L)	HRP (chemiluminescence)	Thermo Fisher Scientific Inc., USA.	31430	1:10000 (TBS-T), western blot
Secondary (polyclonal, generated in goat)	Mouse IgG (gamma 1)	Alexa Fluor™ 488 (fluorescence)	Thermo Fisher Scientific Inc., USA.	A-21121	1:500 (5% FBS in PBS), immunofluorescence

Appendix Table S17. **Software compilation**

Software	Source	Website
Fiji/ImageJ2	Rueden CT, Schindelin J, Hiner MC, DeZonia BE, Walter AE, Arena ET, Eliceiri KW. ImageJ2: ImageJ for the next generation of scientific image data. BMC Bioinformatics. 2017 Nov 29;18(1):529.	https://imagej.net/software/fiji/
MultiStackReg	Brad Busse, National Institutes of Heatlh, USA.	https://biii.eu/multistackreg
TurboReg	Thévenaz P, Ruttimann UE, Unser M. A pyramid approach to subpixel registration based on intensity. IEEE Trans Image Process. 1998;7(1):27-41.	http://bigwww.epfl.ch/thevenaz/turboreg/
JaCoP	Bolte S, Cordelières FP. A guided tour into subcellular colocalization analysis in light microscopy. J Microsc. 2006 Dec;224(Pt 3):213-32.	https://imagej.net/plugins/jacop
Huygens Software	Scientific Volume Imaging B.V., Netherlands.	https://svi.nl/Huygens-Software
GraphPad Prism 9	GraphPad Software LLC., USA.	https://www.graphpad.com/scientific-software/prism/
Microsoft Excel	Microsoft Corp., USA.	https://www.microsoft.com/de-ch/
Microsoft Word	Microsoft Corp., USA.	https://www.microsoft.com/de-ch/
Microsoft PowerPoint	Microsoft Corp., USA.	https://www.microsoft.com/de-ch/
OMERO	University of Dundee & Open Microscopy Environment, UK.	https://www.openmicroscopy.org/omero/
Olympus FV31S-SW	Olympus Corp., Japan.	https://www.olympus-lifescience.com/en/
Zeiss Zen Blue	Carl Zeiss AG, Germany.	https://www.zeiss.com/microscopy/de/produkte/software/zeiss-zen.html
Maps Offline Viewer	Thermo Fisher Scientific Inc., USA.	https://www.thermofisher.com/ch/en/home/global/forms/industrial/maps-offline-viewer-v3.html
EndNote	Clarivate Plc., USA/UK.	https://endnote.com
NEBuilder Assembly Tool	New England BioLabs Inc., USA.	https://nebuilder.neb.com/#!/
CHOPCHOP	Labun K, Montague TG, Krause M, Torres Cleuren YN, Tjeldnes H, Valen E. CHOPCHOP v3: expanding the CRISPR web toolbox beyond genome editing. Nucleic Acids Res. 2019 Jul 2;47(W1):W171-W174.	https://chopchop.cbu.uib.no
Adobe Illustrator	Adobe Inc., USA.	https://www.adobe.com/ch_de/products/illustrator.html
Fusion-CAPT (FUSION-FX7 advanced)	Vilber Lourmat SAS, France.	https://www.vilber.com

Appendix Supplementary Methods

Epon-Araldite protocol

Stock solution:

	ca. 150 ml	ca. 50 ml
Epon 812 (Sigma-Aldrich 45345)	70.89 g	23.63 g
Durcupan ACM (single component A, M epoxy resin) (Sigma-Aldrich 44611)	92.35 g	30.78 g
Dibutyl phthalate (Sigma-Aldrich 524980)	8.68 g	2.89 g

The stock solution can be stored at ambient temperature.

Working solution:

	ca. 20 ml	ca. 7 ml
Stock solution	11.7 g	3.90 g
Dodecenylsuccinic anhydride (DDSA) (Sigma-Aldrich 45346)	10.0 g	3.33 g
2,4,6-Tris-(dimethylaminomethyl)phenol (DMP-30) (Sigma-Aldrich 45348)	620 mg	206 mg

The working solution starts to polymerize immediately after adding the accelerator and should be used within 60 min.
If more than one hour is needed for embedding, reduce amount of accelerator DMP-30 (reduce by 10-20%).

Reynold's lead citrate protocol

Materials

Chemicals:

Lead (II) nitrate $Pb(NO_3)2$, MW: 331.21, Sigma-Aldrich #228621-100G
Lead (II) citrate tribasic trihydrate $C_{12}H_{10}O_{14}Pb_3*3H_2O$, MW: 1053.83, Sigma-Aldrich #15326
Sodium hydroxide pellets, MW: 40.0, Merck #106482

Methods

- Prepare in 3 Erlenmeyer flasks:
  A: Dissolve 1.33 g lead (II) nitrate in 15 ml H_2O
  B: Dissolve 1.76 g lead (II) citrate in 15 ml H_2O
  C: Prepare 8 ml of 1 M NaOH solution
- Mix A+B together into a 50 ml volumetric flask, shake it well until the solution becomes homogenous and milky.
- Add 8 ml of solution C. The solution becomes clear.
- Fill up to 50 ml with H_2O.

Note: The finished solution can be stored at 4°C for several months.

Appendix References

Cullen PJ, Steinberg F (2018) To degrade or not to degrade: mechanisms and significance of endocytic recycling. *Nat Rev Mol Cell Biol* 19: 679-696

Henne WM, Buchkovich NJ, Emr SD (2011) The ESCRT pathway. *Dev Cell* 21: 77-91

Michelet X, Djeddi A, Legouis R (2010) Developmental and cellular functions of the ESCRT machinery in pluricellular organisms. *Biol Cell* 102: 191-202

Pfitzner A-K, Mercier V, Jiang X, Moser von Filseck J, Baum B, Šarić A, Roux A (2020) An ESCRT-III Polymerization Sequence Drives Membrane Deformation and Fission. *Cell* 182: 1140-1155 e1118

Schmidt O, Teis D (2012) The ESCRT machinery. *Curr Biol* 22: R116-120

Schuh AL, Audhya A (2014) The ESCRT machinery: from the plasma membrane to endosomes and back again. *Crit Rev Biochem Mol Biol* 49: 242-261
