# Supplementary figures and images for: Coordination between ESCRT function and Rab conversion during endosome maturation (part 2 of 9)
Source: EMBO J. 2025 Feb 5;44(6):1574–607. doi: 10.1038/s44318-025-00367-7 (PMC11914609; doi:10.1038/s44318-025-00367-7)

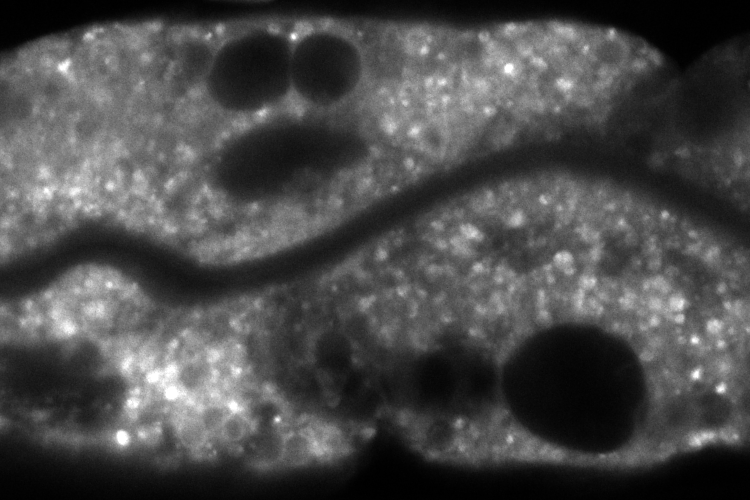

Supplement: Supplementary file 3 — Source data Fig. 1 [file 44318_2025_367_MOESM3_ESM.zip › SD figure 1 /1A/Fig_1_A_Roi/vps-2 (RNAi)/Gut/GFP ART G rab5 rab7 vps2 rnai front_0009-1-1-1-1.tif]

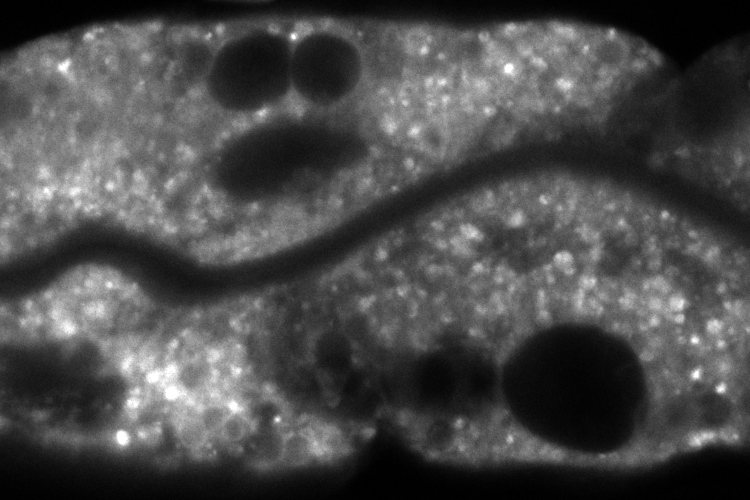

Supplement: Supplementary file 3 — Source data Fig. 1 [file 44318_2025_367_MOESM3_ESM.zip › SD figure 1 /1A/Fig_1_A_Roi/vps-2 (RNAi)/Gut/Merge ART MGM rab5 rab7 vps2 rnai front_0009-1-1-1.tif]

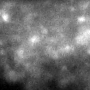

Supplement: Supplementary file 3 — Source data Fig. 1 [file 44318_2025_367_MOESM3_ESM.zip › SD figure 1 /1A/Fig_1_A_Roi/vps-4 (RNAi)/Gut close up/Merge ART C2 MGM rab5 rab7 vps4 rnai front_0001-1-1-1-1-1.tif]

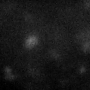

Supplement: Supplementary file 3 — Source data Fig. 1 [file 44318_2025_367_MOESM3_ESM.zip › SD figure 1 /1A/Fig_1_A_Roi/vps-4 (RNAi)/Gut close up/mCherry ART C2 MC rab5 rab7 vps4 rnai front_0001-1-1-1-1-1-1.tif]

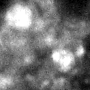

Supplement: Supplementary file 3 — Source data Fig. 1 [file 44318_2025_367_MOESM3_ESM.zip › SD figure 1 /1A/Fig_1_A_Roi/vps-4 (RNAi)/Gut close up/Merge ART C MGM rab5 rab7 vps4 rnai front_0001-1-1-1-1-1.tif]

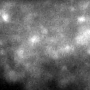

Supplement: Supplementary file 3 — Source data Fig. 1 [file 44318_2025_367_MOESM3_ESM.zip › SD figure 1 /1A/Fig_1_A_Roi/vps-4 (RNAi)/Gut close up/GFP ART C2 G rab5 rab7 vps4 rnai front_0001-1-1-1-1-1-1.tif]

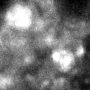

Supplement: Supplementary file 3 — Source data Fig. 1 [file 44318_2025_367_MOESM3_ESM.zip › SD figure 1 /1A/Fig_1_A_Roi/vps-4 (RNAi)/Gut close up/GFP ART C G rab5 rab7 vps4 rnai front_0001-1-1-1-1-1-1.tif]

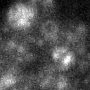

Supplement: Supplementary file 3 — Source data Fig. 1 [file 44318_2025_367_MOESM3_ESM.zip › SD figure 1 /1A/Fig_1_A_Roi/vps-4 (RNAi)/Gut close up/mCherry ART C MC rab5 rab7 vps4 rnai front_0001-1-1-1-1-1-1.tif]

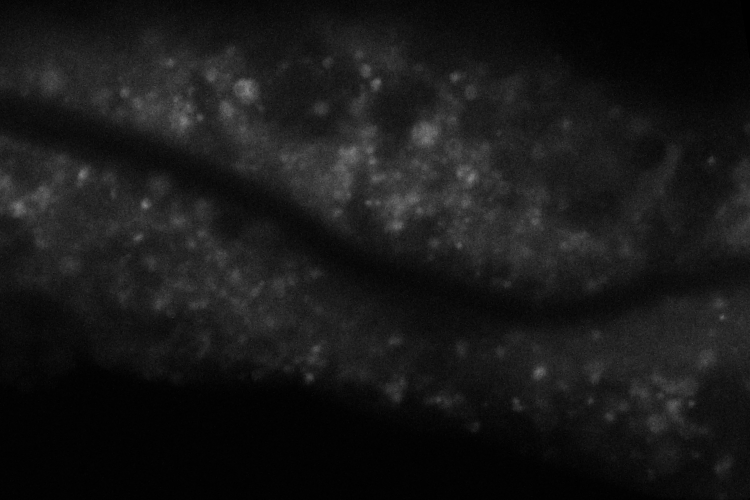

Supplement: Supplementary file 3 — Source data Fig. 1 [file 44318_2025_367_MOESM3_ESM.zip › SD figure 1 /1A/Fig_1_A_Roi/vps-4 (RNAi)/Gut/mCherry ART MC rab5 rab7 vps4 rnai front_0001-1-1-1-1-1.tif]

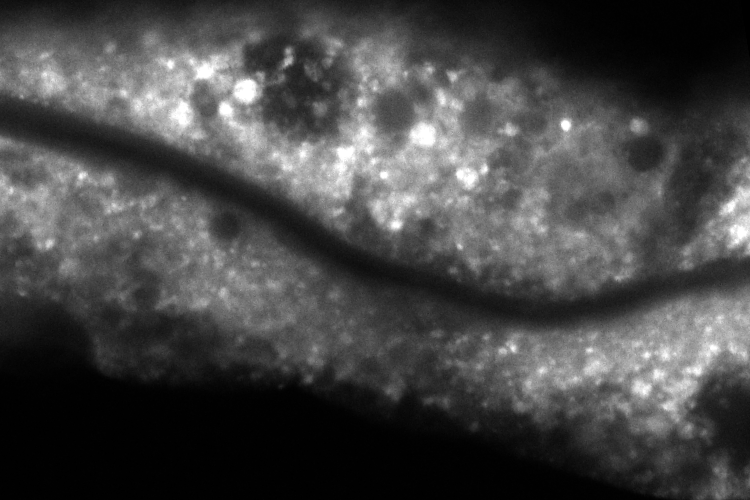

Supplement: Supplementary file 3 — Source data Fig. 1 [file 44318_2025_367_MOESM3_ESM.zip › SD figure 1 /1A/Fig_1_A_Roi/vps-4 (RNAi)/Gut/GFP ART G rab5 rab7 vps4 rnai front_0001-1-1-1-1-1.tif]

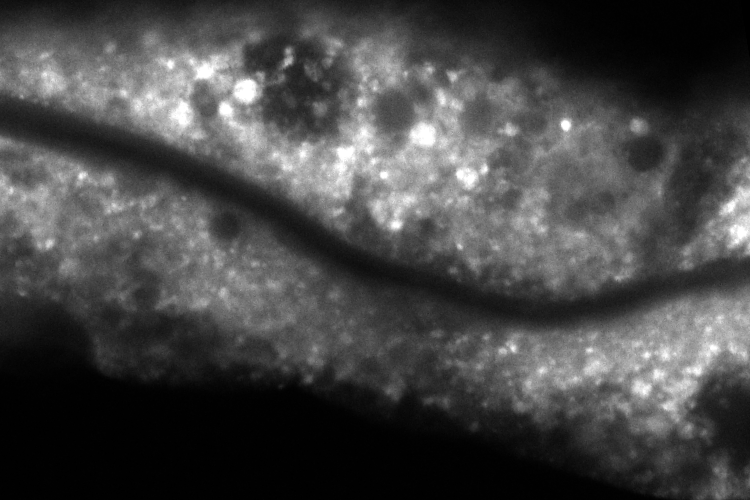

Supplement: Supplementary file 3 — Source data Fig. 1 [file 44318_2025_367_MOESM3_ESM.zip › SD figure 1 /1A/Fig_1_A_Roi/vps-4 (RNAi)/Gut/Merge ART MGM rab5 rab7 vps4 rnai front_0001-1-1-1-1.tif]

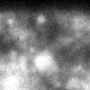

Supplement: Supplementary file 3 — Source data Fig. 1 [file 44318_2025_367_MOESM3_ESM.zip › SD figure 1 /1A/Fig_1_A_Roi/vps-20 (RNAi)/Gut close up/Merge ART C2 MGM rab 5 rab7 vps20 rnai front_0007-1-1-1-1.tif]

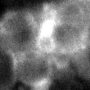

Supplement: Supplementary file 3 — Source data Fig. 1 [file 44318_2025_367_MOESM3_ESM.zip › SD figure 1 /1A/Fig_1_A_Roi/vps-20 (RNAi)/Gut close up/GFP ART C G rab 5 rab7 vps20 rnai front_0007-1-1-1-1-1.tif]

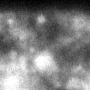

Supplement: Supplementary file 3 — Source data Fig. 1 [file 44318_2025_367_MOESM3_ESM.zip › SD figure 1 /1A/Fig_1_A_Roi/vps-20 (RNAi)/Gut close up/GFP ART C2 G rab 5 rab7 vps20 rnai front_0007-1-1-1-1-1.tif]

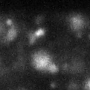

Supplement: Supplementary file 3 — Source data Fig. 1 [file 44318_2025_367_MOESM3_ESM.zip › SD figure 1 /1A/Fig_1_A_Roi/vps-20 (RNAi)/Gut close up/mCherry ART C2 MC rab 5 rab7 vps20 rnai front_0007-1-1-1-1-1.tif]

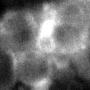

Supplement: Supplementary file 3 — Source data Fig. 1 [file 44318_2025_367_MOESM3_ESM.zip › SD figure 1 /1A/Fig_1_A_Roi/vps-20 (RNAi)/Gut close up/Merge ART C MGM rab 5 rab7 vps20 rnai front_0007-1-1-1-1.tif]

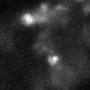

Supplement: Supplementary file 3 — Source data Fig. 1 [file 44318_2025_367_MOESM3_ESM.zip › SD figure 1 /1A/Fig_1_A_Roi/vps-20 (RNAi)/Gut close up/mCherry ART C MC rab 5 rab7 vps20 rnai front_0007-1-1-1-1-1.tif]

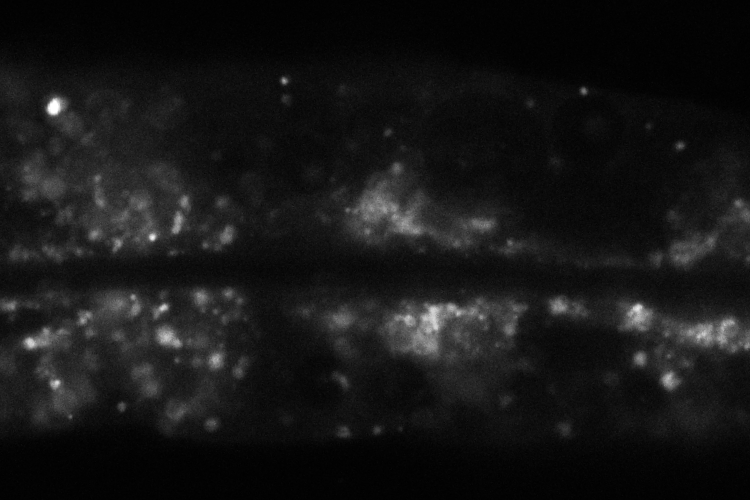

Supplement: Supplementary file 3 — Source data Fig. 1 [file 44318_2025_367_MOESM3_ESM.zip › SD figure 1 /1A/Fig_1_A_Roi/vps-20 (RNAi)/Gut/mCherry ART MC rab 5 rab7 vps20 rnai front_0007-1-1-1-1.tif]

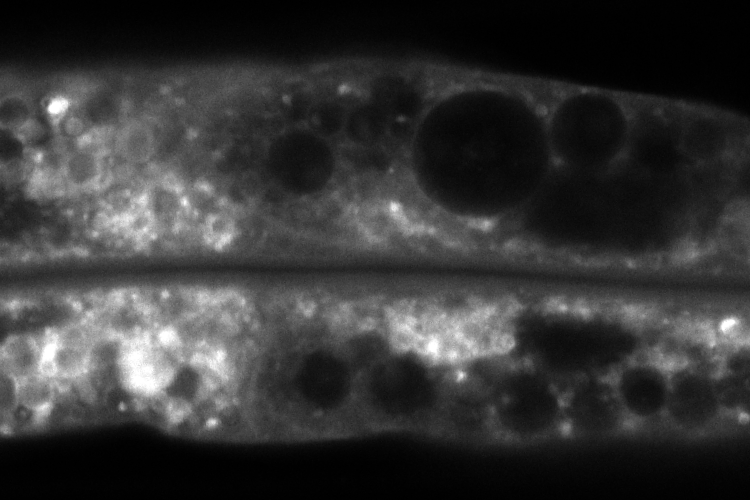

Supplement: Supplementary file 3 — Source data Fig. 1 [file 44318_2025_367_MOESM3_ESM.zip › SD figure 1 /1A/Fig_1_A_Roi/vps-20 (RNAi)/Gut/GFP ART G rab 5 rab7 vps20 rnai front_0007-1-1-1-1.tif]

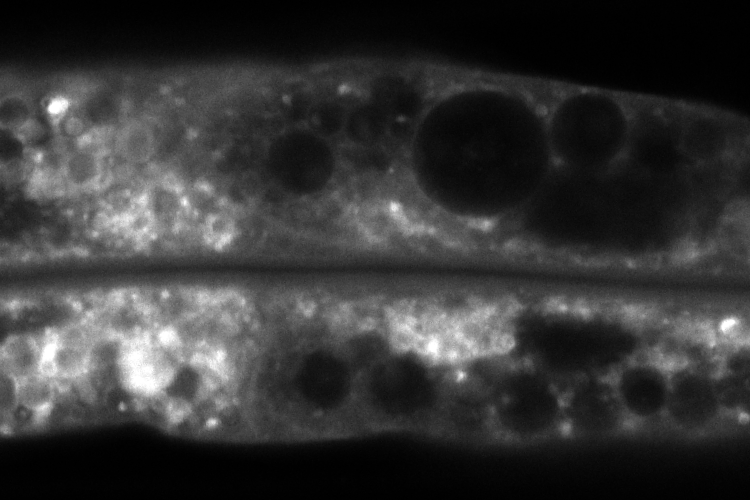

Supplement: Supplementary file 3 — Source data Fig. 1 [file 44318_2025_367_MOESM3_ESM.zip › SD figure 1 /1A/Fig_1_A_Roi/vps-20 (RNAi)/Gut/Merge ART MGM rab 5 rab7 vps20 rnai front_0007-1-1-1.tif]

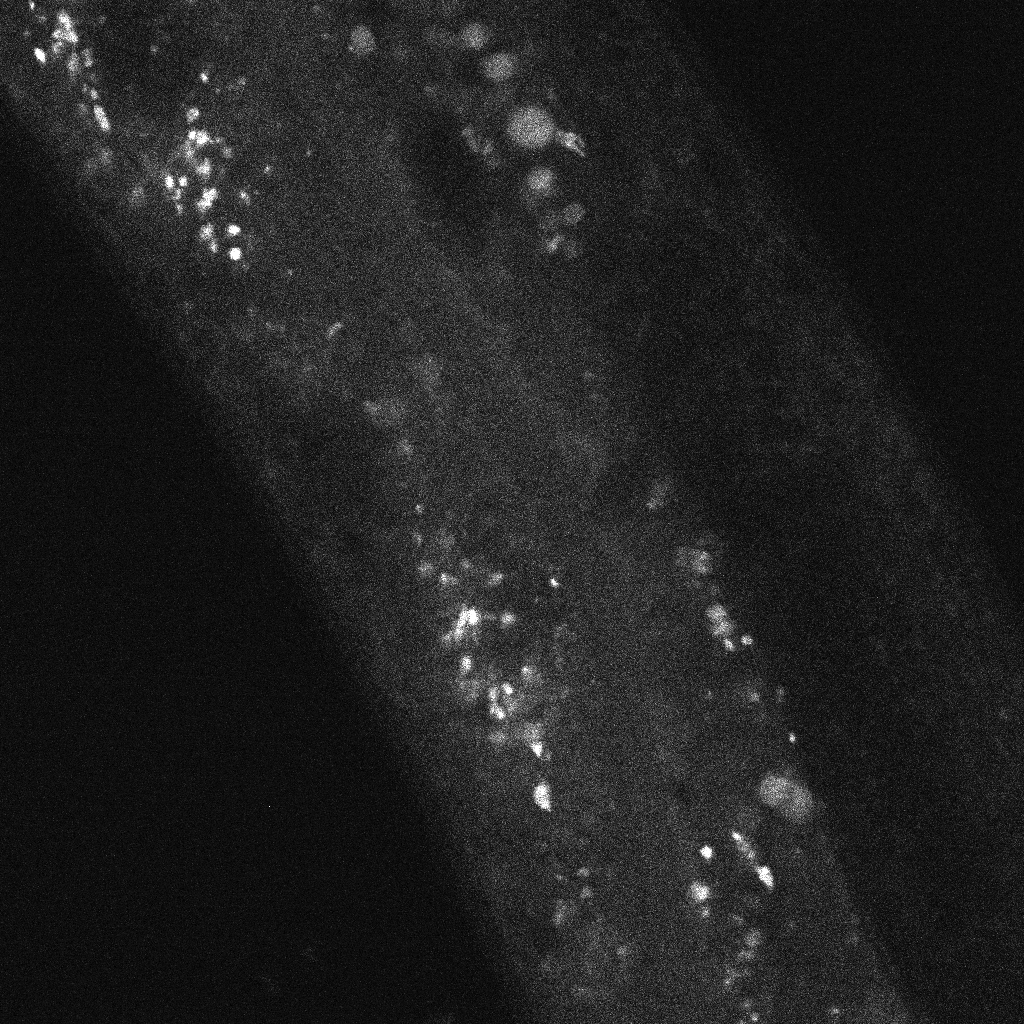

Supplement: Supplementary file 5 — Source data Fig. 3 [file 44318_2025_367_MOESM5_ESM.zip › SD figure 3/3E/Fig_3_E_data/vps-20 (RNAi)/A mCherrzRab7 GFPvps27 vps20 RNAi front_0014-1.tif]

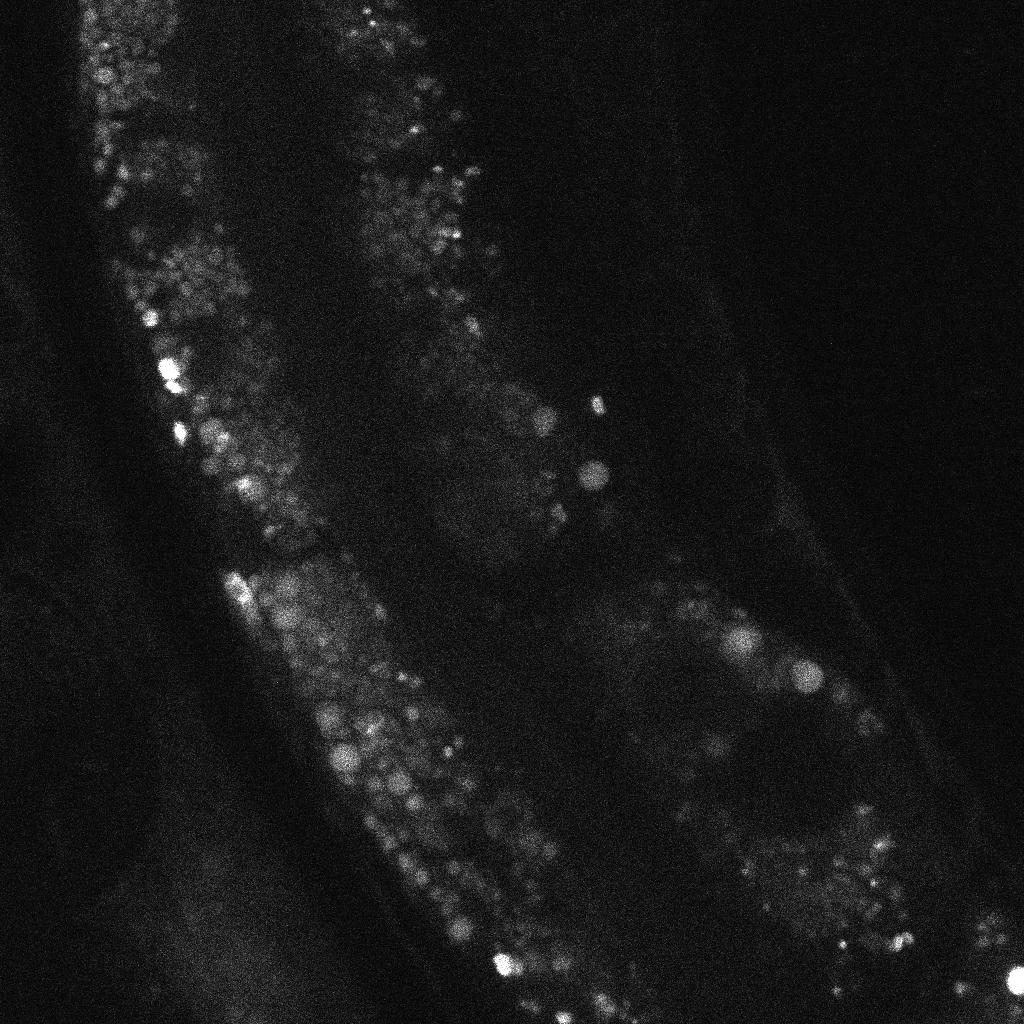

Supplement: Supplementary file 5 — Source data Fig. 3 [file 44318_2025_367_MOESM5_ESM.zip › SD figure 3/3E/Fig_3_E_data/Mock/A mCherrzRab7 GFPvps27 control RNAi front_0018-1.tif]

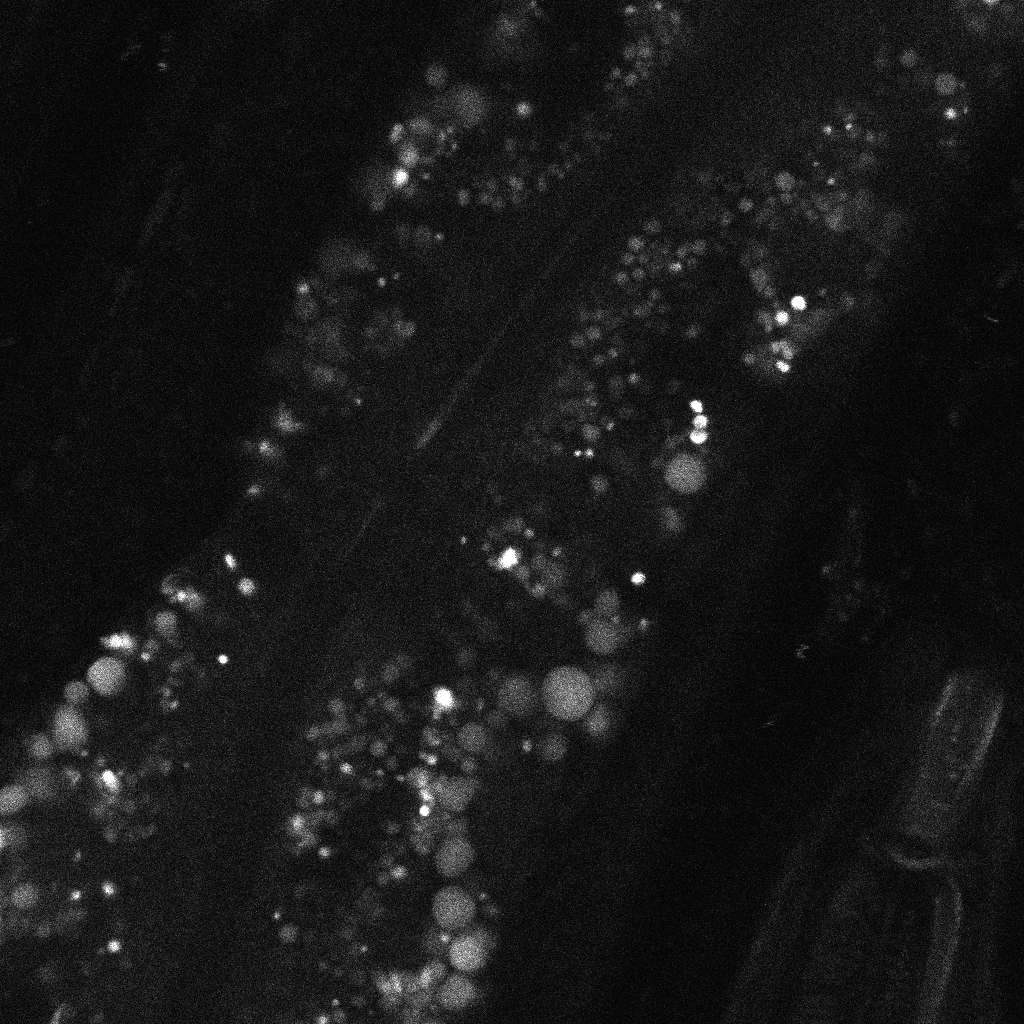

Supplement: Supplementary file 5 — Source data Fig. 3 [file 44318_2025_367_MOESM5_ESM.zip › SD figure 3/3E/Fig_3_E_data/vps-24 (RNAi)/A mCherrzRab7 GFPvps27 vps24 RNAi front_0013-1.tif]

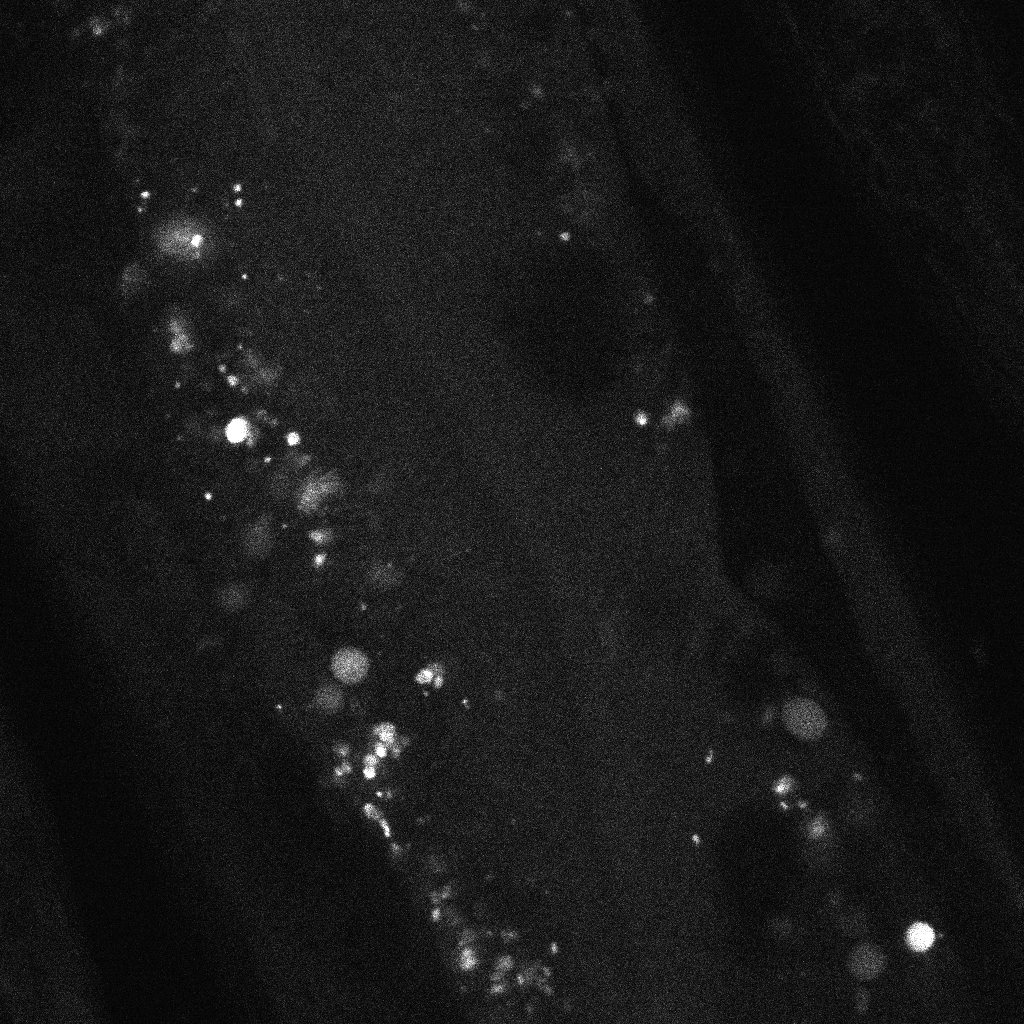

Supplement: Supplementary file 5 — Source data Fig. 3 [file 44318_2025_367_MOESM5_ESM.zip › SD figure 3/3E/Fig_3_E_data/vps-2 (RNAi)/A mCherrzrab7 GFPvps27 vps2 RNAi front_0011-1.tif]

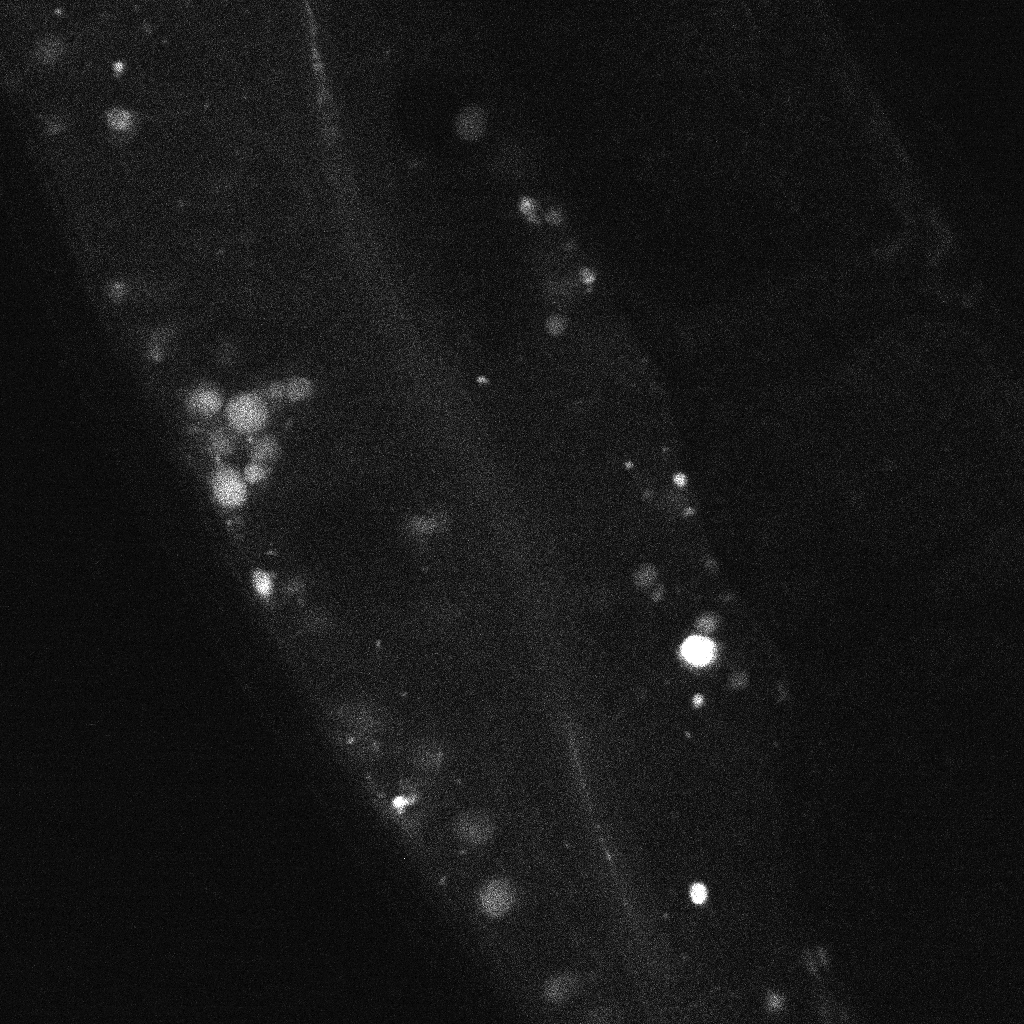

Supplement: Supplementary file 5 — Source data Fig. 3 [file 44318_2025_367_MOESM5_ESM.zip › SD figure 3/3E/Fig_3_E_data/tsg-101 (RNAi)/A mCherrzRab7 GFPvps27 tsg101 RNAi front_0002-1.tif]

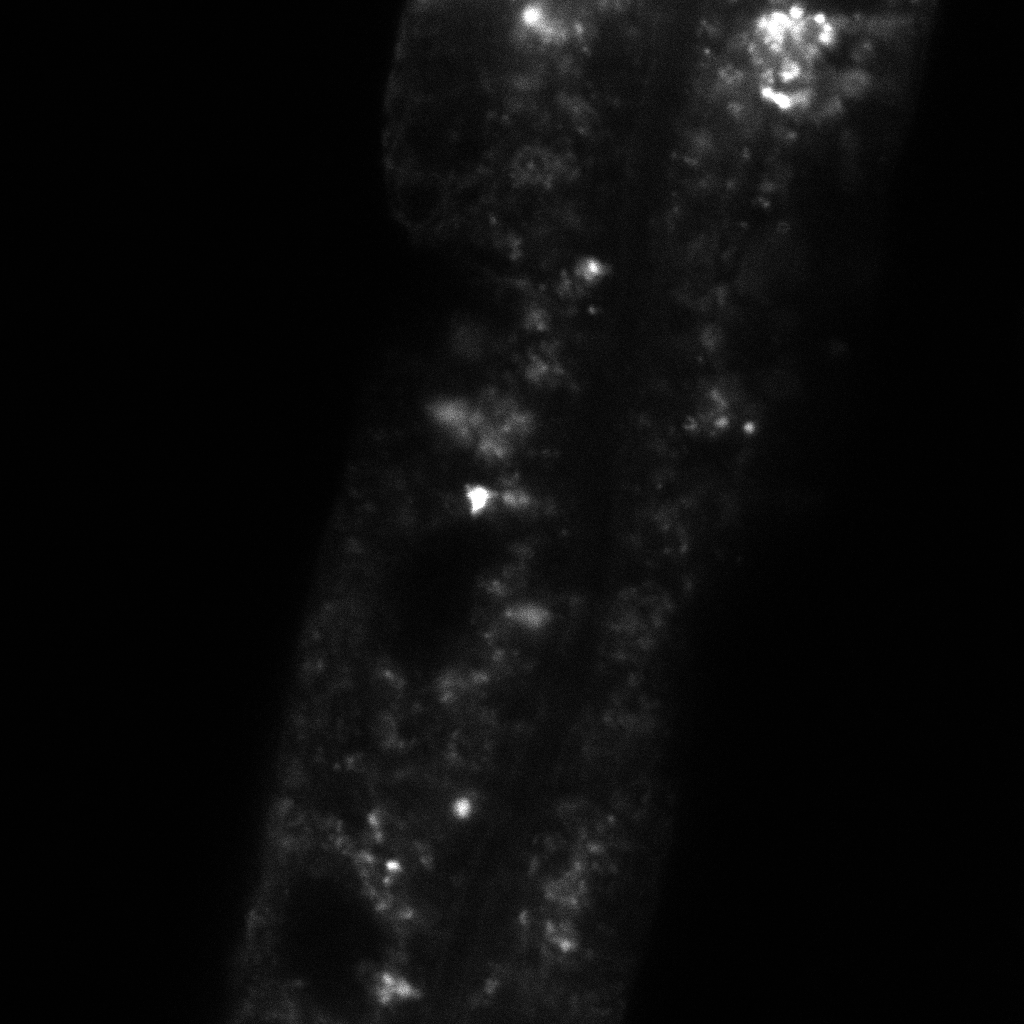

Supplement: Supplementary file 5 — Source data Fig. 3 [file 44318_2025_367_MOESM5_ESM.zip › SD figure 3/3A/Fig_3_A_data/vps-20 (RNAi)/GFPvps27 RFPrab5 vps20 RNAi front_0005-1.tif]

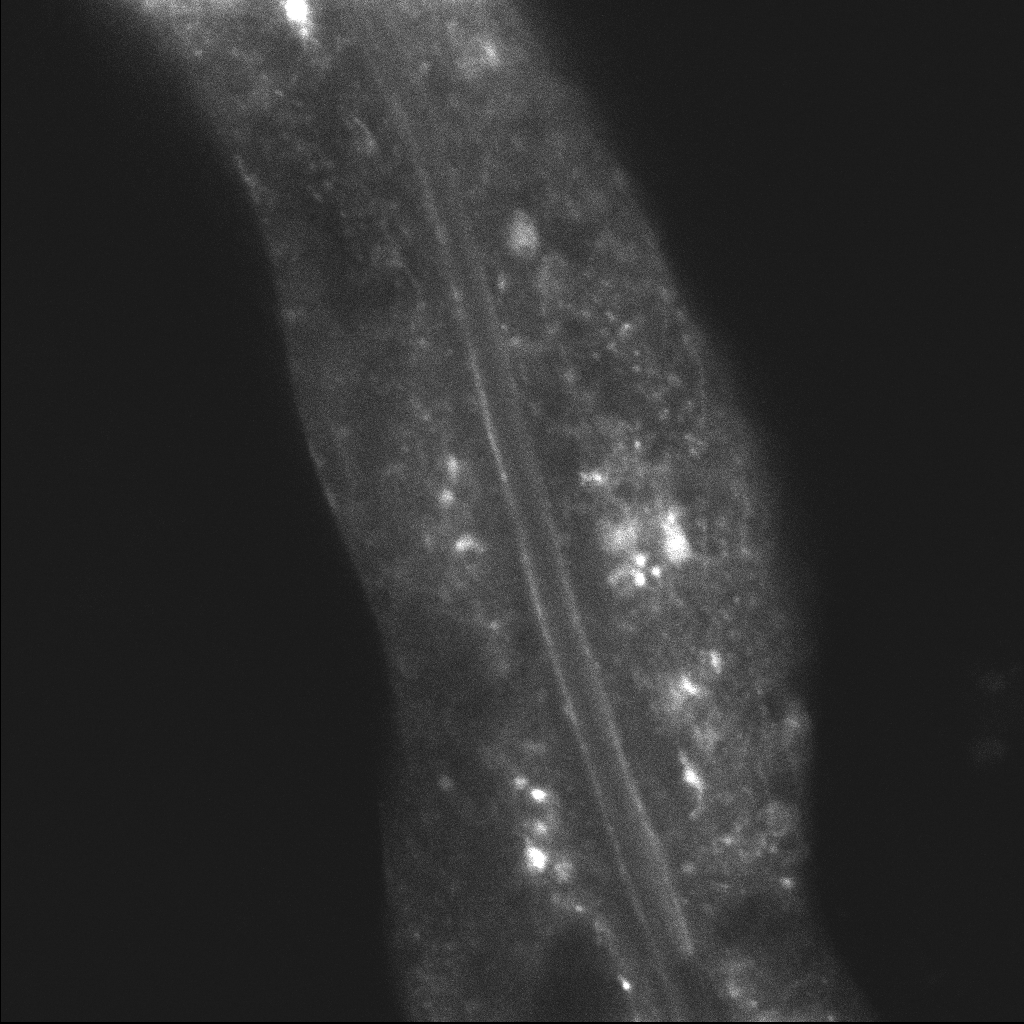

Supplement: Supplementary file 5 — Source data Fig. 3 [file 44318_2025_367_MOESM5_ESM.zip › SD figure 3/3A/Fig_3_A_data/Mock/GFPvps27 RFPrab5 control RNAi front mz final setting_0010 aligned.tif]

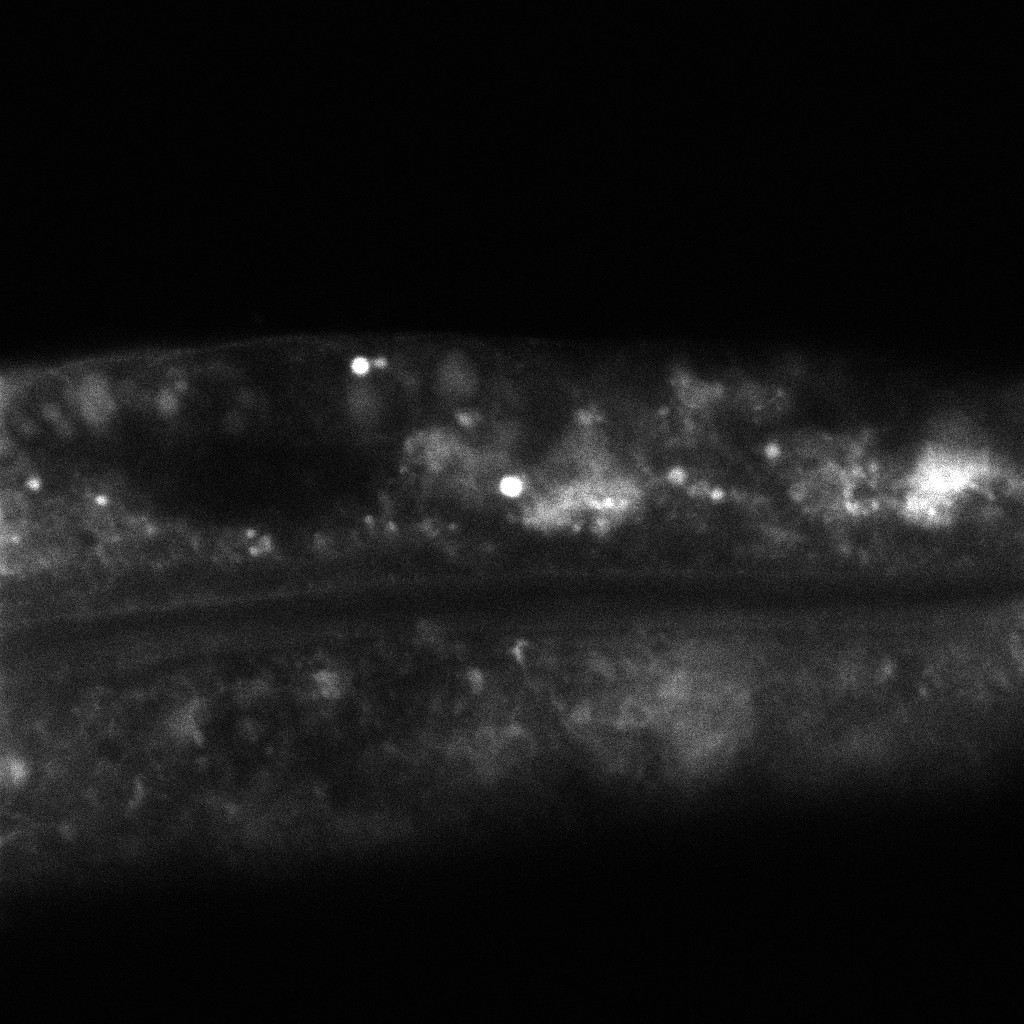

Supplement: Supplementary file 5 — Source data Fig. 3 [file 44318_2025_367_MOESM5_ESM.zip › SD figure 3/3A/Fig_3_A_data/vps-24 (RNAi)/GFPvps27 RFPrab5 vps24 RNAi front_0010-1.tif]

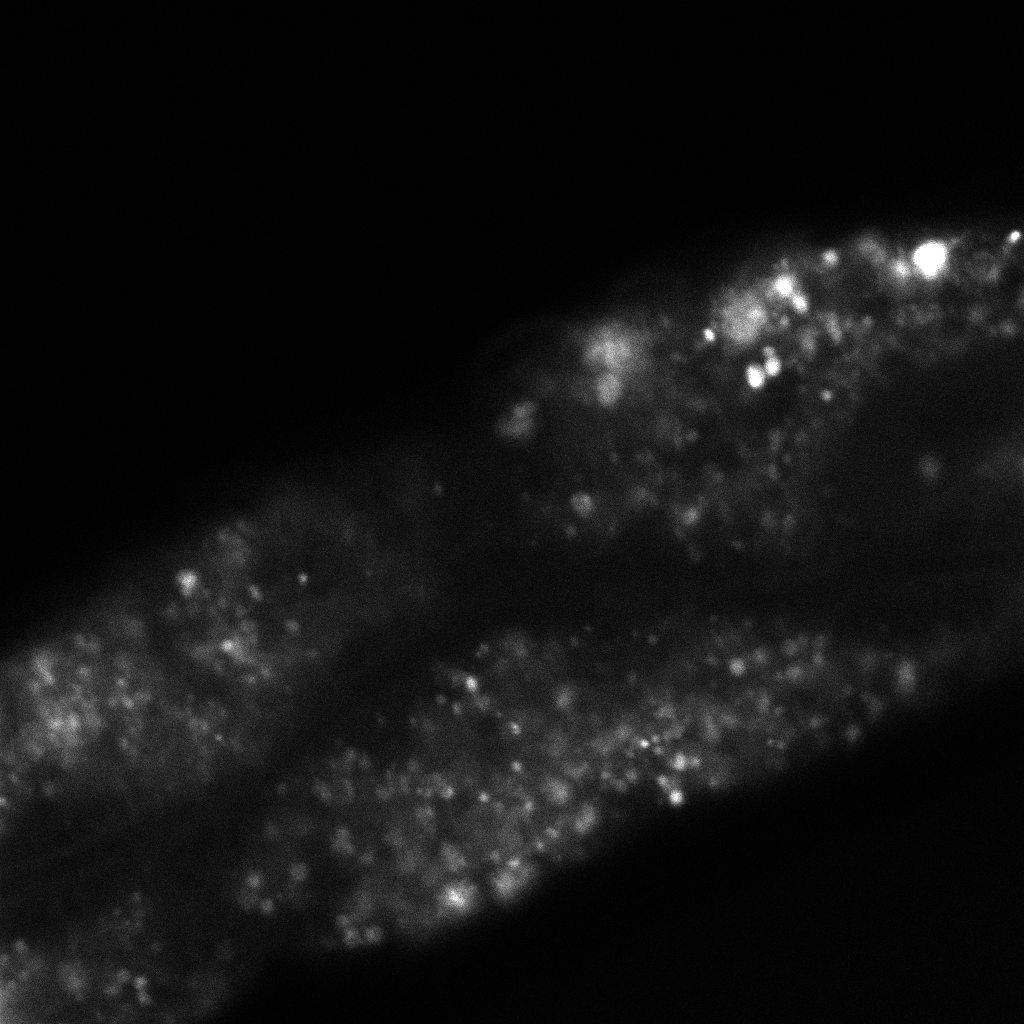

Supplement: Supplementary file 5 — Source data Fig. 3 [file 44318_2025_367_MOESM5_ESM.zip › SD figure 3/3A/Fig_3_A_data/vps-2 (RNAi)/GFPvps27 RFPrab5 vps2 RNAi front_0017-1.tif]

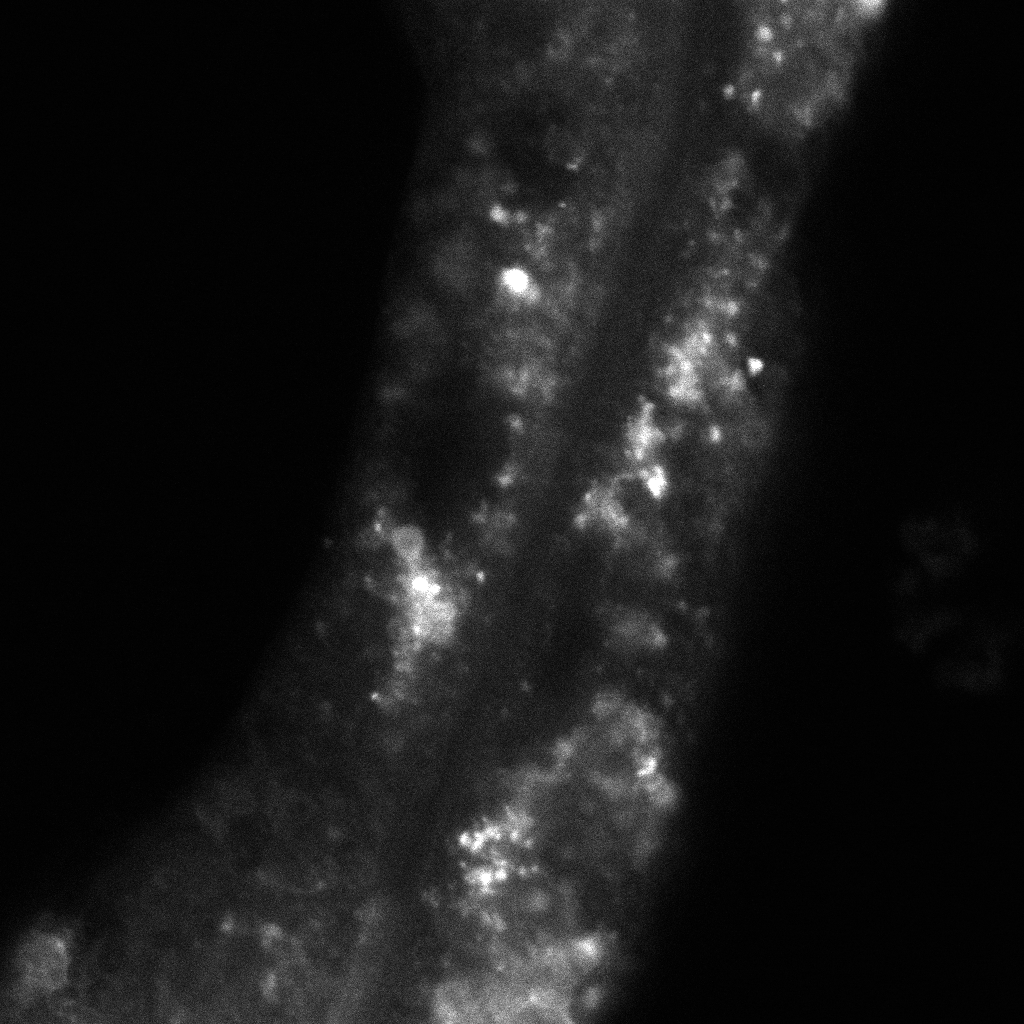

Supplement: Supplementary file 5 — Source data Fig. 3 [file 44318_2025_367_MOESM5_ESM.zip › SD figure 3/3A/Fig_3_A_data/vps-4 (RNAi) pre fed/GFPvps27 RFPrab5 vps4 RNAi L3 pre front_0007-1.tif]

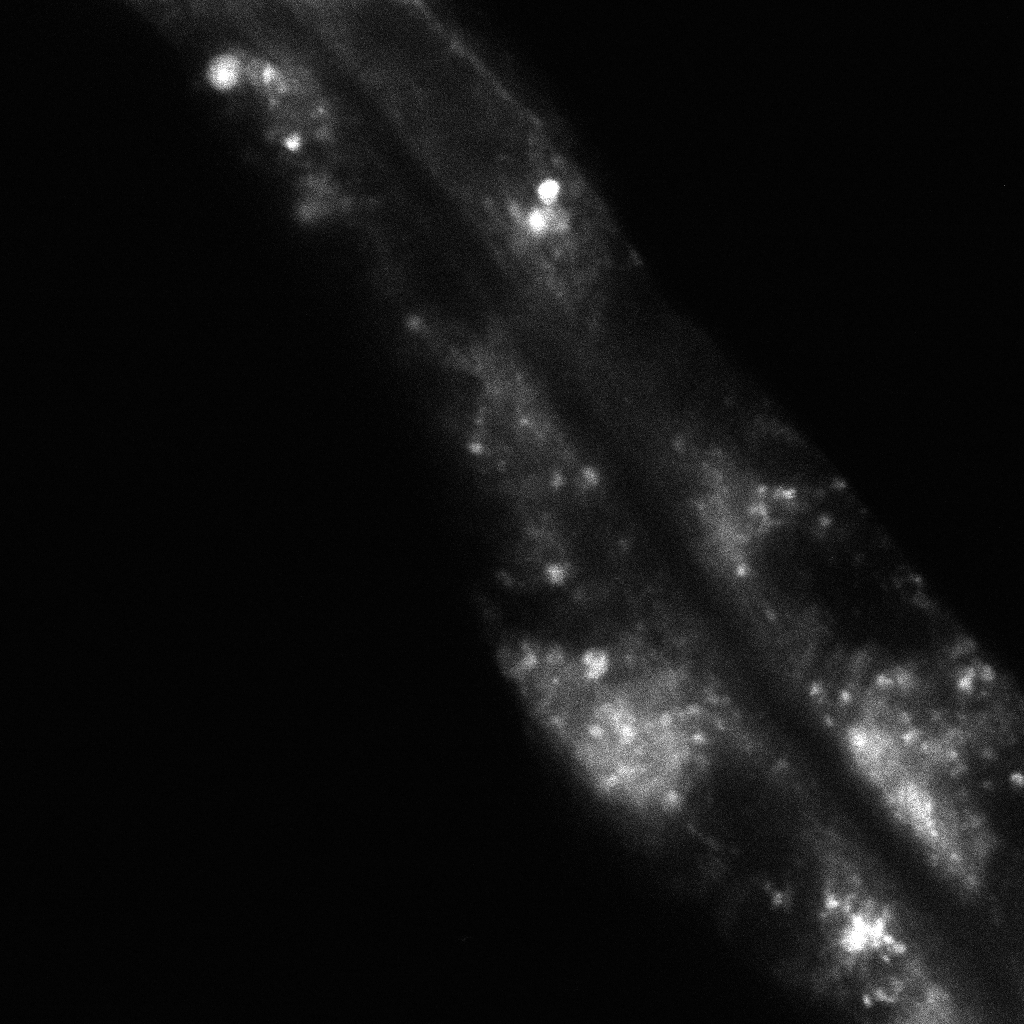

Supplement: Supplementary file 5 — Source data Fig. 3 [file 44318_2025_367_MOESM5_ESM.zip › SD figure 3/3A/Fig_3_A_data/tsg-101 (RNAi)/GFPvps27 RFPrab5 tsg101 RNAi front_0010-1.tif]

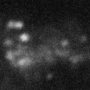

Supplement: Supplementary file 5 — Source data Fig. 3 [file 44318_2025_367_MOESM5_ESM.zip › SD figure 3/3E/Fig_3_E_Roi/vps-20 (RNAi)/Gut close up/mCherry ART C2 MC mCherrzRab7 GFPvps27 vps20 RNAi front_0014-1-1-1-1-1.tif]

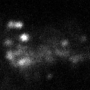

Supplement: Supplementary file 5 — Source data Fig. 3 [file 44318_2025_367_MOESM5_ESM.zip › SD figure 3/3E/Fig_3_E_Roi/vps-20 (RNAi)/Gut close up/Merge ART C2 MGM mCherrzRab7 GFPvps27 vps20 RNAi front_0014-1-1-1-1-1.tif]

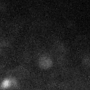

Supplement: Supplementary file 5 — Source data Fig. 3 [file 44318_2025_367_MOESM5_ESM.zip › SD figure 3/3E/Fig_3_E_Roi/vps-20 (RNAi)/Gut close up/mCherry ART C MC mCherrzRab7 GFPvps27 vps20 RNAi front_0014-1-1-1-1-1.tif]

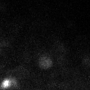

Supplement: Supplementary file 5 — Source data Fig. 3 [file 44318_2025_367_MOESM5_ESM.zip › SD figure 3/3E/Fig_3_E_Roi/vps-20 (RNAi)/Gut close up/Merge ART C MGM mCherrzRab7 GFPvps27 vps20 RNAi front_0014-1-1-1-1-1.tif]

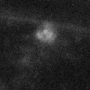

Supplement: Supplementary file 5 — Source data Fig. 3 [file 44318_2025_367_MOESM5_ESM.zip › SD figure 3/3E/Fig_3_E_Roi/vps-20 (RNAi)/Gut close up/GFP ART C2 G mCherrzRab7 GFPvps27 vps20 RNAi front_0014-1-1-1-1-1.tif]

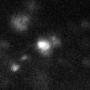

Supplement: Supplementary file 5 — Source data Fig. 3 [file 44318_2025_367_MOESM5_ESM.zip › SD figure 3/3E/Fig_3_E_Roi/vps-20 (RNAi)/Gut close up/GFP ART C G mCherrzRab7 GFPvps27 vps20 RNAi front_0014-1-1-1-1-1.tif]

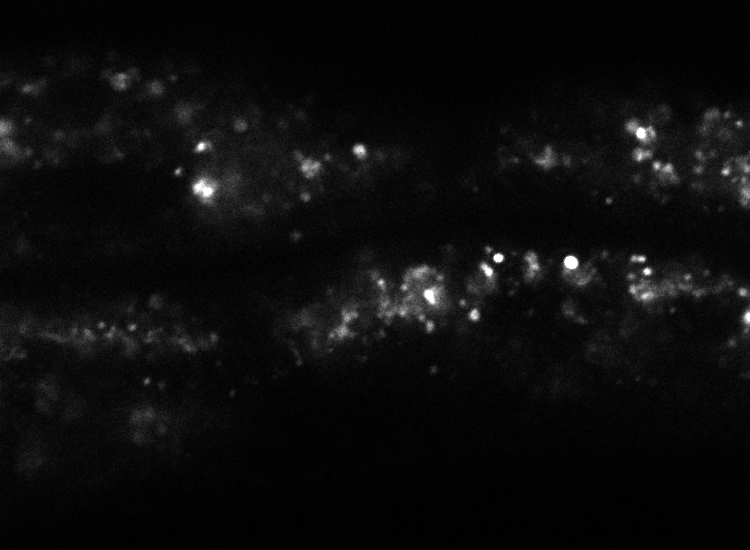

Supplement: Supplementary file 5 — Source data Fig. 3 [file 44318_2025_367_MOESM5_ESM.zip › SD figure 3/3E/Fig_3_E_Roi/vps-20 (RNAi)/Gut/Merge ART MGM mCherrzRab7 GFPvps27 vps20 RNAi front_0014-1-1-1-1.tif]

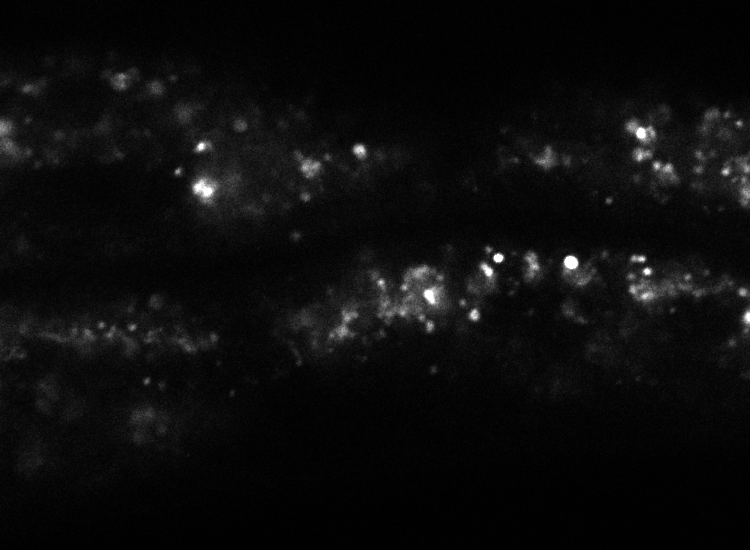

Supplement: Supplementary file 5 — Source data Fig. 3 [file 44318_2025_367_MOESM5_ESM.zip › SD figure 3/3E/Fig_3_E_Roi/vps-20 (RNAi)/Gut/mCherry ART MC mCherrzRab7 GFPvps27 vps20 RNAi front_0014-1-1-1-1.tif]

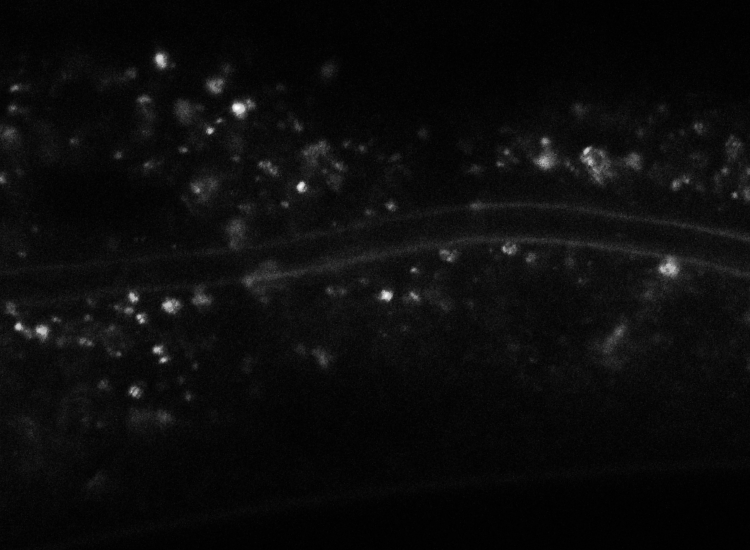

Supplement: Supplementary file 5 — Source data Fig. 3 [file 44318_2025_367_MOESM5_ESM.zip › SD figure 3/3E/Fig_3_E_Roi/vps-20 (RNAi)/Gut/GFP ART G mCherrzRab7 GFPvps27 vps20 RNAi front_0014-1-1-1-1.tif]

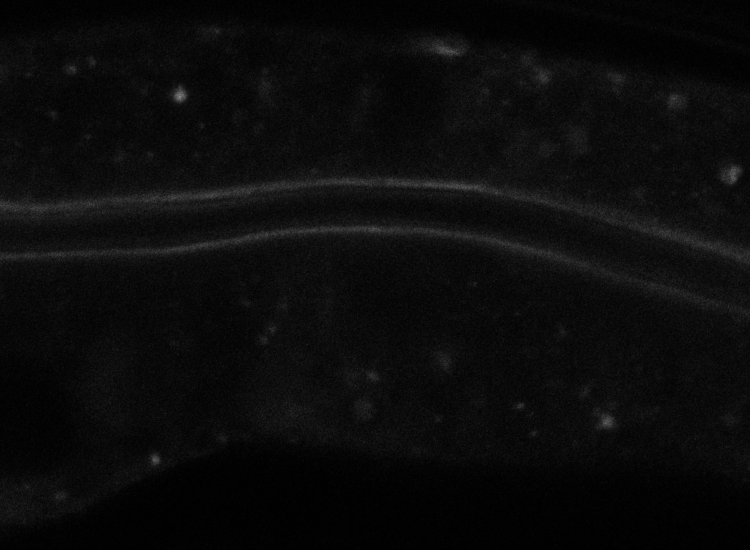

Supplement: Supplementary file 5 — Source data Fig. 3 [file 44318_2025_367_MOESM5_ESM.zip › SD figure 3/3E/Fig_3_E_Roi/Mock/Gut /GFP ART G mCherrzRab7 GFPvps27 control RNAi front_0018-1-1-1-1.tif]

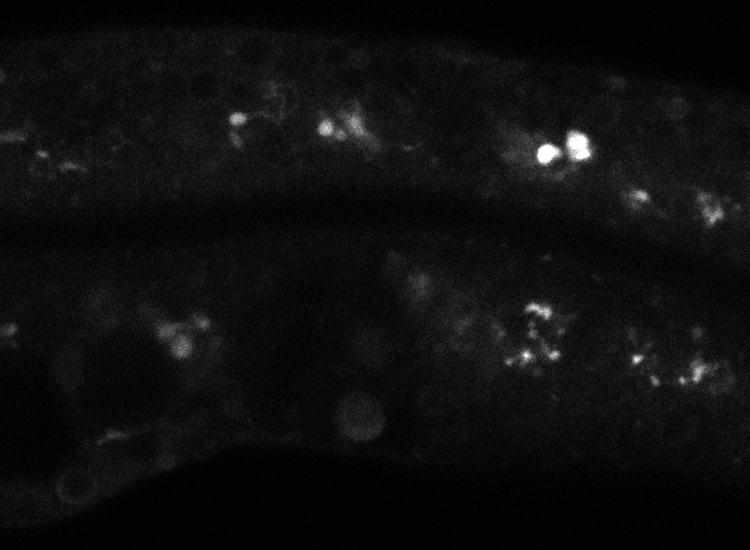

Supplement: Supplementary file 5 — Source data Fig. 3 [file 44318_2025_367_MOESM5_ESM.zip › SD figure 3/3E/Fig_3_E_Roi/Mock/Gut /Merge ART MGM mCherrzRab7 GFPvps27 control RNAi front_0018-1-1-1-1.tif]

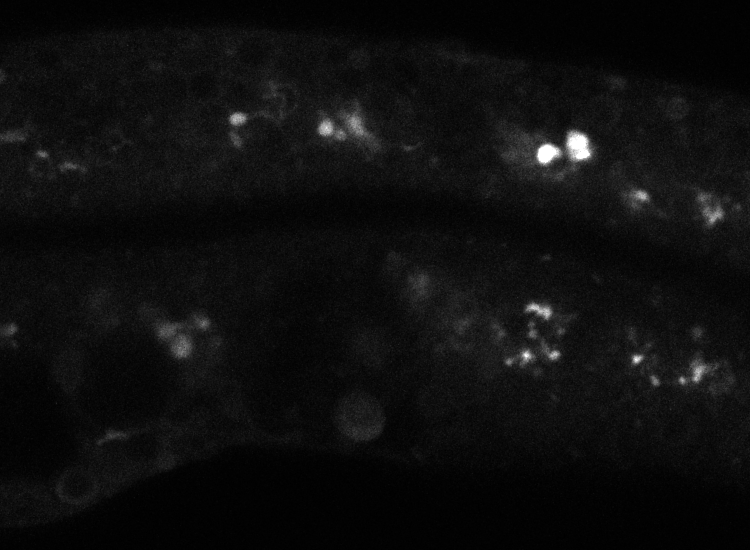

Supplement: Supplementary file 5 — Source data Fig. 3 [file 44318_2025_367_MOESM5_ESM.zip › SD figure 3/3E/Fig_3_E_Roi/Mock/Gut /mCherry ART MC mCherrzRab7 GFPvps27 control RNAi front_0018-1-1-1-1.tif]

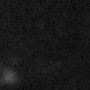

Supplement: Supplementary file 5 — Source data Fig. 3 [file 44318_2025_367_MOESM5_ESM.zip › SD figure 3/3E/Fig_3_E_Roi/Mock/Gut close up/GFP ART C4 G mCherrzRab7 GFPvps27 control RNAi front_0018-1-1-1-1-1.tif]

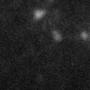

Supplement: Supplementary file 5 — Source data Fig. 3 [file 44318_2025_367_MOESM5_ESM.zip › SD figure 3/3E/Fig_3_E_Roi/Mock/Gut close up/mCherry ART C4 MC mCherrzRab7 GFPvps27 control RNAi front_0018-1-1-1-1-1.tif]

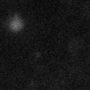

Supplement: Supplementary file 5 — Source data Fig. 3 [file 44318_2025_367_MOESM5_ESM.zip › SD figure 3/3E/Fig_3_E_Roi/Mock/Gut close up/GFP ART C3 G mCherrzRab7 GFPvps27 control RNAi front_0018-1-1-1-1-1.tif]

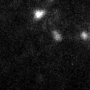

Supplement: Supplementary file 5 — Source data Fig. 3 [file 44318_2025_367_MOESM5_ESM.zip › SD figure 3/3E/Fig_3_E_Roi/Mock/Gut close up/Merge ART C4 MGM mCherrzRab7 GFPvps27 control RNAi front_0018-1-1-1-1-1.tif]

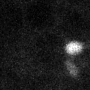

Supplement: Supplementary file 5 — Source data Fig. 3 [file 44318_2025_367_MOESM5_ESM.zip › SD figure 3/3E/Fig_3_E_Roi/Mock/Gut close up/Merge ART C3 MGM mCherrzRab7 GFPvps27 control RNAi front_0018-1-1-1-1-1.tif]

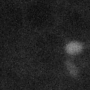

Supplement: Supplementary file 5 — Source data Fig. 3 [file 44318_2025_367_MOESM5_ESM.zip › SD figure 3/3E/Fig_3_E_Roi/Mock/Gut close up/mCherry ART C3 MC mCherrzRab7 GFPvps27 control RNAi front_0018-1-1-1-1-1.tif]

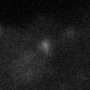

Supplement: Supplementary file 5 — Source data Fig. 3 [file 44318_2025_367_MOESM5_ESM.zip › SD figure 3/3E/Fig_3_E_Roi/vps-24 (RNAi)/Gut close up/GFP ART C G mCherrzRab7 GFPvps27 vps24 RNAi front_0013-1-1-1-1-1.tif]

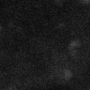

Supplement: Supplementary file 5 — Source data Fig. 3 [file 44318_2025_367_MOESM5_ESM.zip › SD figure 3/3E/Fig_3_E_Roi/vps-24 (RNAi)/Gut close up/GFP ART C2 G mCherrzRab7 GFPvps27 vps24 RNAi front_0013-1-1-1-1-1.tif]

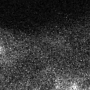

Supplement: Supplementary file 5 — Source data Fig. 3 [file 44318_2025_367_MOESM5_ESM.zip › SD figure 3/3E/Fig_3_E_Roi/vps-24 (RNAi)/Gut close up/Merge ART C MGM mCherrzRab7 GFPvps27 vps24 RNAi front_0013-1-1-1-1-1.tif]

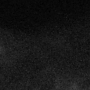

Supplement: Supplementary file 5 — Source data Fig. 3 [file 44318_2025_367_MOESM5_ESM.zip › SD figure 3/3E/Fig_3_E_Roi/vps-24 (RNAi)/Gut close up/mCherry ART C MC mCherrzRab7 GFPvps27 vps24 RNAi front_0013-1-1-1-1-1.tif]

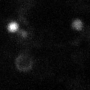

Supplement: Supplementary file 5 — Source data Fig. 3 [file 44318_2025_367_MOESM5_ESM.zip › SD figure 3/3E/Fig_3_E_Roi/vps-24 (RNAi)/Gut close up/Merge ART C2 MGM mCherrzRab7 GFPvps27 vps24 RNAi front_0013-1-1-1-1-1.tif]

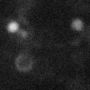

Supplement: Supplementary file 5 — Source data Fig. 3 [file 44318_2025_367_MOESM5_ESM.zip › SD figure 3/3E/Fig_3_E_Roi/vps-24 (RNAi)/Gut close up/mCherry ART C2 MC mCherrzRab7 GFPvps27 vps24 RNAi front_0013-1-1-1-1-1.tif]

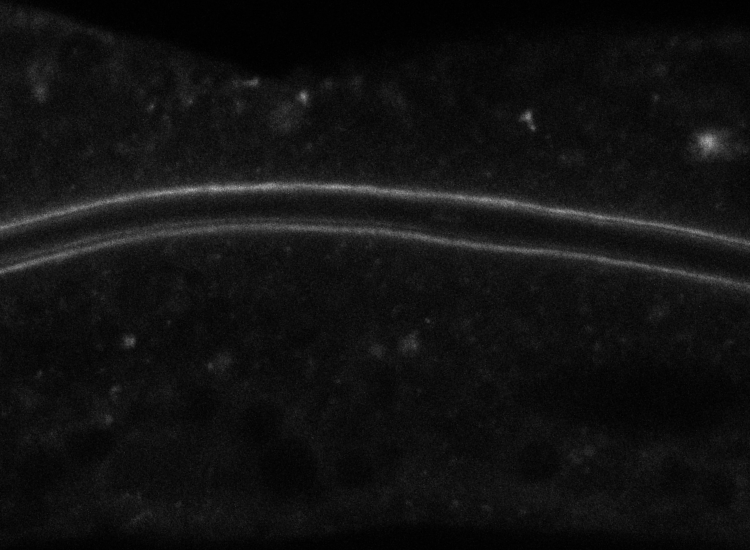

Supplement: Supplementary file 5 — Source data Fig. 3 [file 44318_2025_367_MOESM5_ESM.zip › SD figure 3/3E/Fig_3_E_Roi/vps-24 (RNAi)/Gut/GFP ART G mCherrzRab7 GFPvps27 vps24 RNAi front_0013-1-1-1-1.tif]

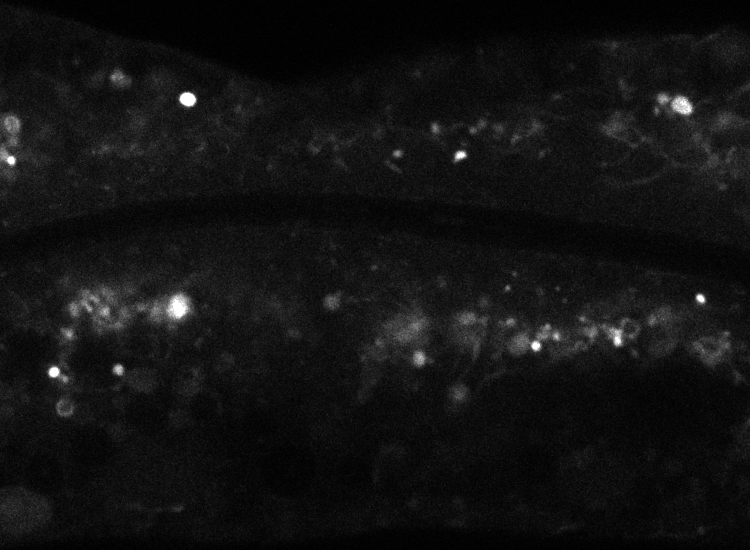

Supplement: Supplementary file 5 — Source data Fig. 3 [file 44318_2025_367_MOESM5_ESM.zip › SD figure 3/3E/Fig_3_E_Roi/vps-24 (RNAi)/Gut/Merge ART MGM mCherrzRab7 GFPvps27 vps24 RNAi front_0013-1-1-1-1.tif]

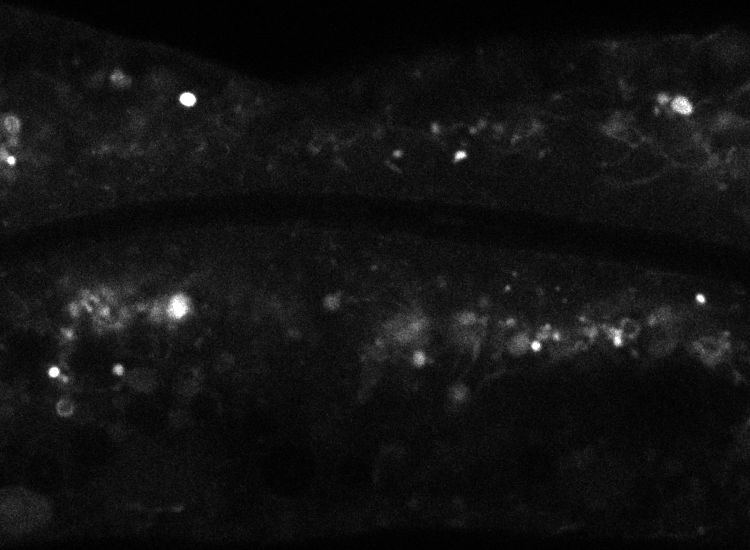

Supplement: Supplementary file 5 — Source data Fig. 3 [file 44318_2025_367_MOESM5_ESM.zip › SD figure 3/3E/Fig_3_E_Roi/vps-24 (RNAi)/Gut/mCherry ART MC mCherrzRab7 GFPvps27 vps24 RNAi front_0013-1-1-1-1.tif]

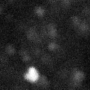

Supplement: Supplementary file 5 — Source data Fig. 3 [file 44318_2025_367_MOESM5_ESM.zip › SD figure 3/3E/Fig_3_E_Roi/vps-2 (RNAi)/Gut close up/mCherry ART C2 MC mCherrzrab7 GFPvps27 vps2 RNAi front_0011-1-1-1-1-1.tif]

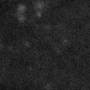

Supplement: Supplementary file 5 — Source data Fig. 3 [file 44318_2025_367_MOESM5_ESM.zip › SD figure 3/3E/Fig_3_E_Roi/vps-2 (RNAi)/Gut close up/mCherry ART C3 MC mCherrzrab7 GFPvps27 vps2 RNAi front_0011-1-1-1-1-1.tif]

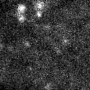

Supplement: Supplementary file 5 — Source data Fig. 3 [file 44318_2025_367_MOESM5_ESM.zip › SD figure 3/3E/Fig_3_E_Roi/vps-2 (RNAi)/Gut close up/Merge ART C3 MGM mCherrzrab7 GFPvps27 vps2 RNAi front_0011-1-1-1-1-1.tif]

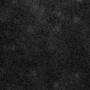

Supplement: Supplementary file 5 — Source data Fig. 3 [file 44318_2025_367_MOESM5_ESM.zip › SD figure 3/3E/Fig_3_E_Roi/vps-2 (RNAi)/Gut close up/GFP ART C2 G mCherrzrab7 GFPvps27 vps2 RNAi front_0011-1-1-1-1-1.tif]

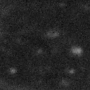

Supplement: Supplementary file 5 — Source data Fig. 3 [file 44318_2025_367_MOESM5_ESM.zip › SD figure 3/3E/Fig_3_E_Roi/vps-2 (RNAi)/Gut close up/GFP ART C3 G mCherrzrab7 GFPvps27 vps2 RNAi front_0011-1-1-1-1-1.tif]

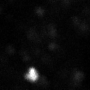

Supplement: Supplementary file 5 — Source data Fig. 3 [file 44318_2025_367_MOESM5_ESM.zip › SD figure 3/3E/Fig_3_E_Roi/vps-2 (RNAi)/Gut close up/Merge ART C2 MGM mCherrzrab7 GFPvps27 vps2 RNAi front_0011-1-1-1-1-1.tif]

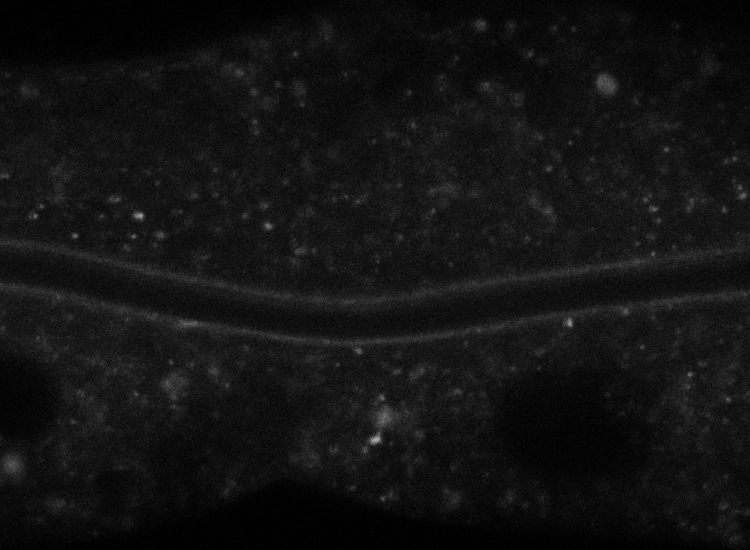

Supplement: Supplementary file 5 — Source data Fig. 3 [file 44318_2025_367_MOESM5_ESM.zip › SD figure 3/3E/Fig_3_E_Roi/vps-2 (RNAi)/Gut/GFP ART G mCherrzrab7 GFPvps27 vps2 RNAi front_0011-1-1-1-1.tif]

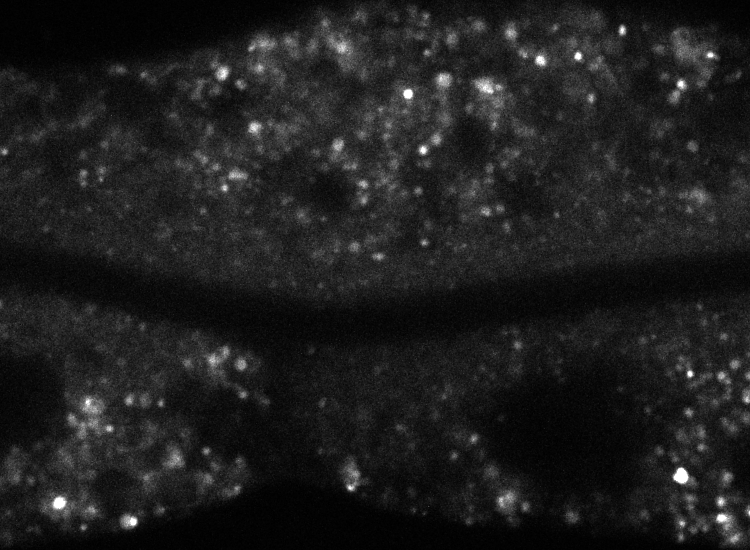

Supplement: Supplementary file 5 — Source data Fig. 3 [file 44318_2025_367_MOESM5_ESM.zip › SD figure 3/3E/Fig_3_E_Roi/vps-2 (RNAi)/Gut/mCherry ART MC mCherrzrab7 GFPvps27 vps2 RNAi front_0011-1-1-1-1.tif]

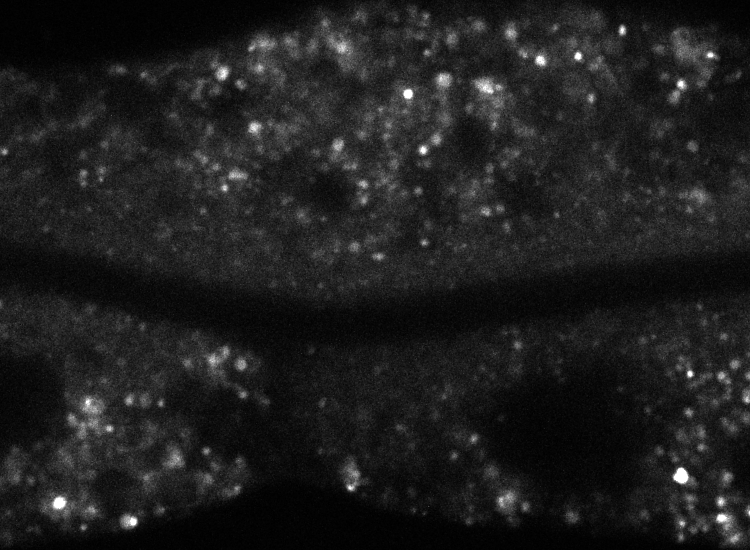

Supplement: Supplementary file 5 — Source data Fig. 3 [file 44318_2025_367_MOESM5_ESM.zip › SD figure 3/3E/Fig_3_E_Roi/vps-2 (RNAi)/Gut/Merge ART MGM mCherrzrab7 GFPvps27 vps2 RNAi front_0011-1-1-1-1.tif]

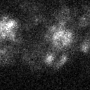

Supplement: Supplementary file 5 — Source data Fig. 3 [file 44318_2025_367_MOESM5_ESM.zip › SD figure 3/3E/Fig_3_E_Roi/tsg-101 (RNAi)/Gut close up/Merge ART C MGM mCherrzRab7 GFPvps27 tsg101 RNAi front_0002-1-1-1-1-1.tif]

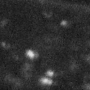

Supplement: Supplementary file 5 — Source data Fig. 3 [file 44318_2025_367_MOESM5_ESM.zip › SD figure 3/3E/Fig_3_E_Roi/tsg-101 (RNAi)/Gut close up/GFP ART C2 G mCherrzRab7 GFPvps27 tsg101 RNAi front_0002-1-1-1-1-1.tif]

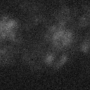

Supplement: Supplementary file 5 — Source data Fig. 3 [file 44318_2025_367_MOESM5_ESM.zip › SD figure 3/3E/Fig_3_E_Roi/tsg-101 (RNAi)/Gut close up/mCherry ART C MC mCherrzRab7 GFPvps27 tsg101 RNAi front_0002-1-1-1-1-1.tif]

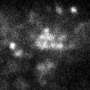

Supplement: Supplementary file 5 — Source data Fig. 3 [file 44318_2025_367_MOESM5_ESM.zip › SD figure 3/3E/Fig_3_E_Roi/tsg-101 (RNAi)/Gut close up/mCherry ART C2 MC mCherrzRab7 GFPvps27 tsg101 RNAi front_0002-1-1-1-1-1.tif]

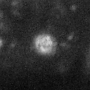

Supplement: Supplementary file 5 — Source data Fig. 3 [file 44318_2025_367_MOESM5_ESM.zip › SD figure 3/3E/Fig_3_E_Roi/tsg-101 (RNAi)/Gut close up/GFP ART C G mCherrzRab7 GFPvps27 tsg101 RNAi front_0002-1-1-1-1-1.tif]

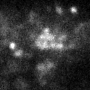

Supplement: Supplementary file 5 — Source data Fig. 3 [file 44318_2025_367_MOESM5_ESM.zip › SD figure 3/3E/Fig_3_E_Roi/tsg-101 (RNAi)/Gut close up/Merge ART C2 MGM mCherrzRab7 GFPvps27 tsg101 RNAi front_0002-1-1-1-1-1.tif]

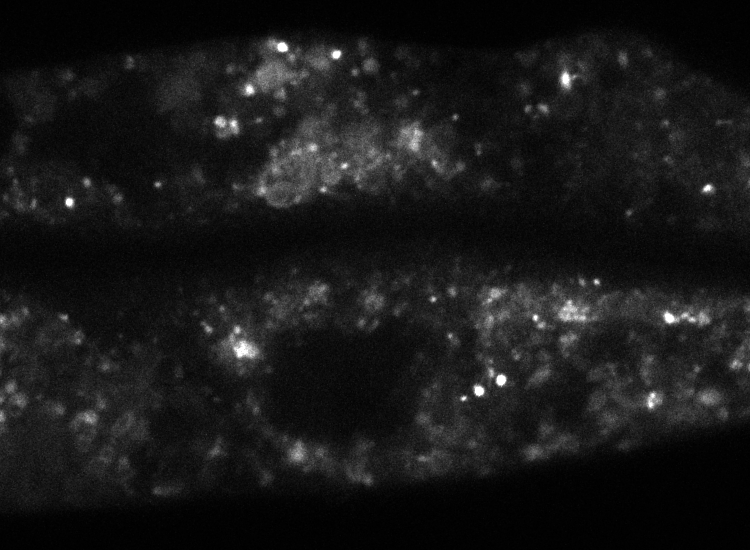

Supplement: Supplementary file 5 — Source data Fig. 3 [file 44318_2025_367_MOESM5_ESM.zip › SD figure 3/3E/Fig_3_E_Roi/tsg-101 (RNAi)/Gut/Merge ART MGM mCherrzRab7 GFPvps27 tsg101 RNAi front_0002-1-1-1-1.tif]

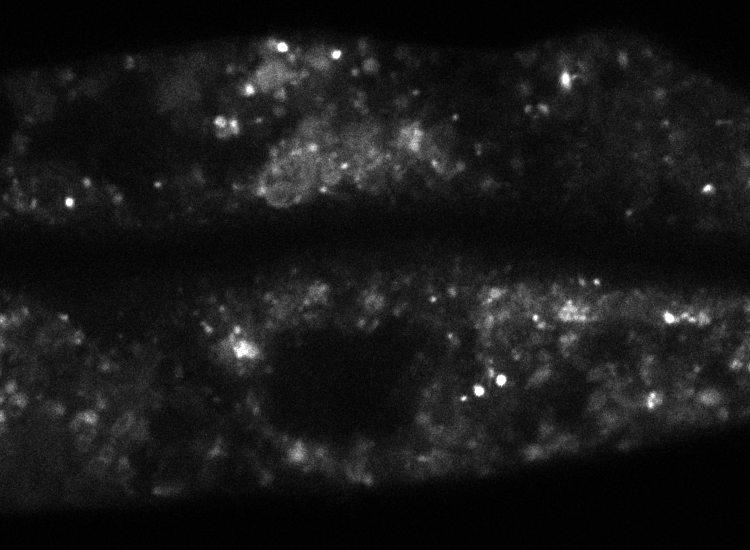

Supplement: Supplementary file 5 — Source data Fig. 3 [file 44318_2025_367_MOESM5_ESM.zip › SD figure 3/3E/Fig_3_E_Roi/tsg-101 (RNAi)/Gut/mCherry ART MC mCherrzRab7 GFPvps27 tsg101 RNAi front_0002-1-1-1-1.tif]

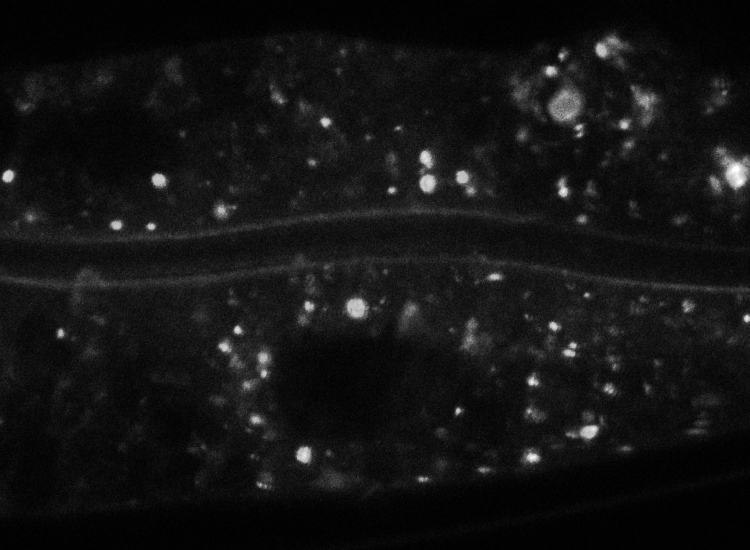

Supplement: Supplementary file 5 — Source data Fig. 3 [file 44318_2025_367_MOESM5_ESM.zip › SD figure 3/3E/Fig_3_E_Roi/tsg-101 (RNAi)/Gut/GFP ART G mCherrzRab7 GFPvps27 tsg101 RNAi front_0002-1-1-1-1.tif]

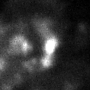

Supplement: Supplementary file 5 — Source data Fig. 3 [file 44318_2025_367_MOESM5_ESM.zip › SD figure 3/3A/Fig_3_A_Roi/vps-20 (RNAi)/Gut close up/Merge C2 MGRA GFPvps27 RFPrab5 vps20 RNAi front_0005-ART-3.tif]

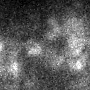

Supplement: Supplementary file 5 — Source data Fig. 3 [file 44318_2025_367_MOESM5_ESM.zip › SD figure 3/3A/Fig_3_A_Roi/vps-20 (RNAi)/Gut close up/Merge C MGRA GFPvps27 RFPrab5 vps20 RNAi front_0005-ART-1.tif]

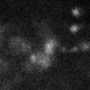

Supplement: Supplementary file 5 — Source data Fig. 3 [file 44318_2025_367_MOESM5_ESM.zip › SD figure 3/3A/Fig_3_A_Roi/vps-20 (RNAi)/Gut close up/GFP C2 G GFPvps27 RFPrab5 vps20 RNAi front_0005-ART-3-1.tif]

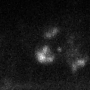

Supplement: Supplementary file 5 — Source data Fig. 3 [file 44318_2025_367_MOESM5_ESM.zip › SD figure 3/3A/Fig_3_A_Roi/vps-20 (RNAi)/Gut close up/GFP C G GFPvps27 RFPrab5 vps20 RNAi front_0005-ART-1-1.tif]

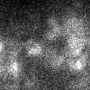

Supplement: Supplementary file 5 — Source data Fig. 3 [file 44318_2025_367_MOESM5_ESM.zip › SD figure 3/3A/Fig_3_A_Roi/vps-20 (RNAi)/Gut close up/RFP C R GFPvps27 RFPrab5 vps20 RNAi front_0005-ART-1-1.tif]

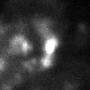

Supplement: Supplementary file 5 — Source data Fig. 3 [file 44318_2025_367_MOESM5_ESM.zip › SD figure 3/3A/Fig_3_A_Roi/vps-20 (RNAi)/Gut close up/RFP C2 R GFPvps27 RFPrab5 vps20 RNAi front_0005-ART-3-1.tif]

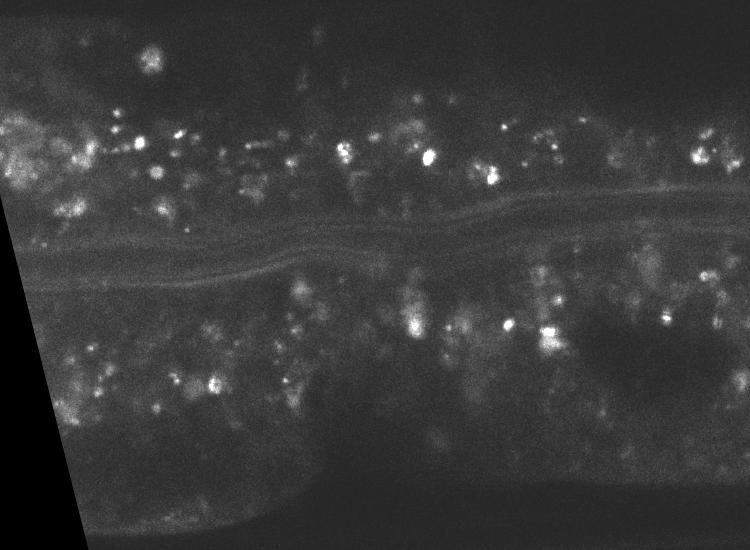

Supplement: Supplementary file 5 — Source data Fig. 3 [file 44318_2025_367_MOESM5_ESM.zip › SD figure 3/3A/Fig_3_A_Roi/vps-20 (RNAi)/Gut/GFP GFPvps27 RFPrab5 vps20 RNAi front_0005-ART-3.tif]

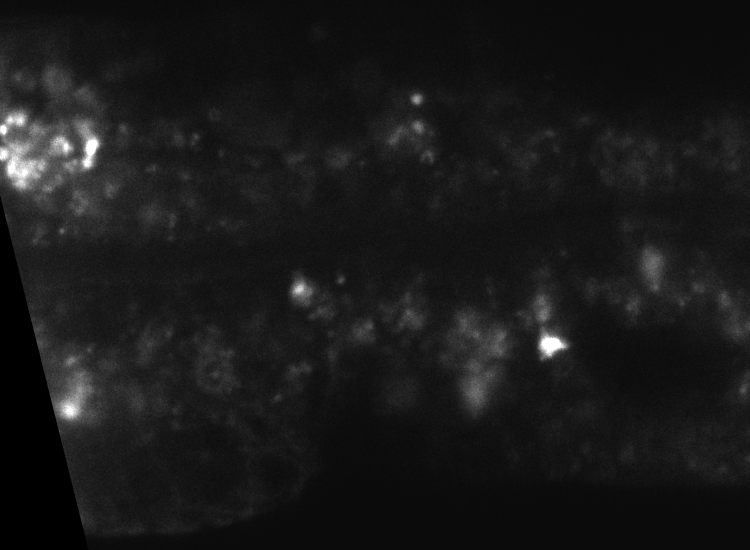

Supplement: Supplementary file 5 — Source data Fig. 3 [file 44318_2025_367_MOESM5_ESM.zip › SD figure 3/3A/Fig_3_A_Roi/vps-20 (RNAi)/Gut/RFP GFPvps27 RFPrab5 vps20 RNAi front_0005-ART-2.tif]

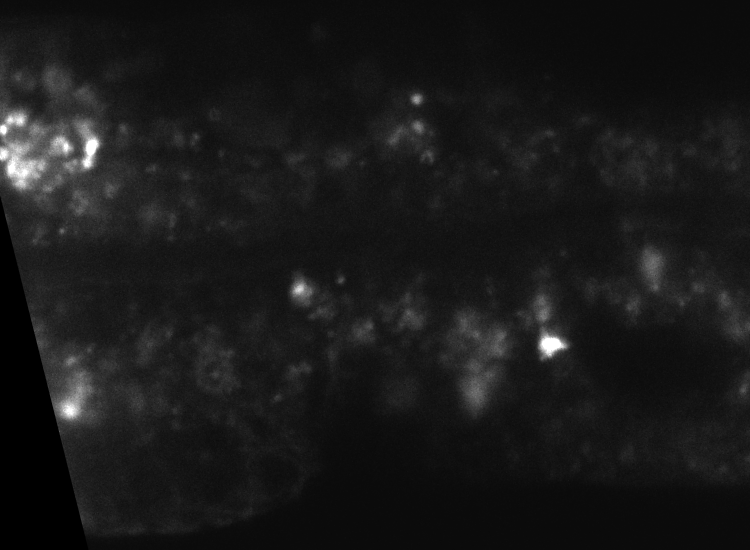

Supplement: Supplementary file 5 — Source data Fig. 3 [file 44318_2025_367_MOESM5_ESM.zip › SD figure 3/3A/Fig_3_A_Roi/vps-20 (RNAi)/Gut/Merge GFPvps27 RFPrab5 vps20 RNAi front_0005-ART.tif]

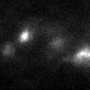

Supplement: Supplementary file 5 — Source data Fig. 3 [file 44318_2025_367_MOESM5_ESM.zip › SD figure 3/3A/Fig_3_A_Roi/Mock/Gut close up/Merge C2 MGRA GFPvps27 RFPrab5 control RNAi front mz final setting_0010 aligned-ART-1.tif]

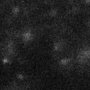

Supplement: Supplementary file 5 — Source data Fig. 3 [file 44318_2025_367_MOESM5_ESM.zip › SD figure 3/3A/Fig_3_A_Roi/Mock/Gut close up/GFP C2 G GFPvps27 RFPrab5 control RNAi front mz final setting_0010 aligned-ART-1-1.tif]

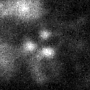

Supplement: Supplementary file 5 — Source data Fig. 3 [file 44318_2025_367_MOESM5_ESM.zip › SD figure 3/3A/Fig_3_A_Roi/Mock/Gut close up/Merge C MGRA GFPvps27 RFPrab5 control RNAi front mz final setting_0010 aligned-ART-1.tif]

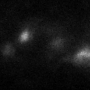

Supplement: Supplementary file 5 — Source data Fig. 3 [file 44318_2025_367_MOESM5_ESM.zip › SD figure 3/3A/Fig_3_A_Roi/Mock/Gut close up/RFP C2 R GFPvps27 RFPrab5 control RNAi front mz final setting_0010 aligned-ART-1-1.tif]

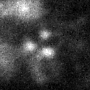

Supplement: Supplementary file 5 — Source data Fig. 3 [file 44318_2025_367_MOESM5_ESM.zip › SD figure 3/3A/Fig_3_A_Roi/Mock/Gut close up/RFP C R GFPvps27 RFPrab5 control RNAi front mz final setting_0010 aligned-ART-1-1.tif]

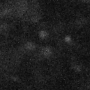

Supplement: Supplementary file 5 — Source data Fig. 3 [file 44318_2025_367_MOESM5_ESM.zip › SD figure 3/3A/Fig_3_A_Roi/Mock/Gut close up/GFP C G GFPvps27 RFPrab5 control RNAi front mz final setting_0010 aligned-ART-1-1.tif]

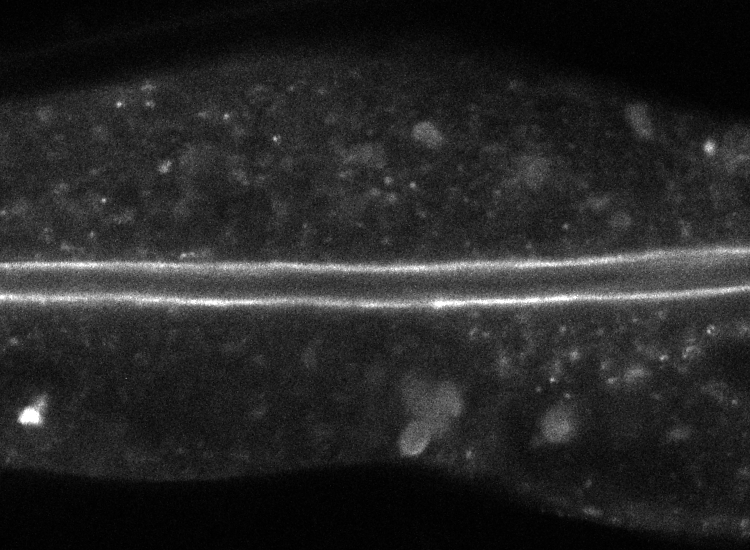

Supplement: Supplementary file 5 — Source data Fig. 3 [file 44318_2025_367_MOESM5_ESM.zip › SD figure 3/3A/Fig_3_A_Roi/Mock/Gut/GFP GFPvps27 RFPrab5 control RNAi front mz final setting_0010 aligned-ART-3.tif]

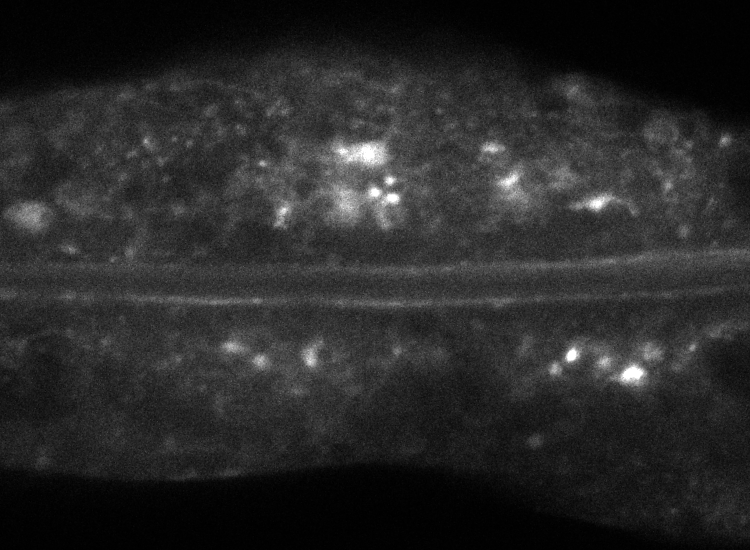

Supplement: Supplementary file 5 — Source data Fig. 3 [file 44318_2025_367_MOESM5_ESM.zip › SD figure 3/3A/Fig_3_A_Roi/Mock/Gut/Merge GFPvps27 RFPrab5 control RNAi front mz final setting_0010 aligned-ART.tif]

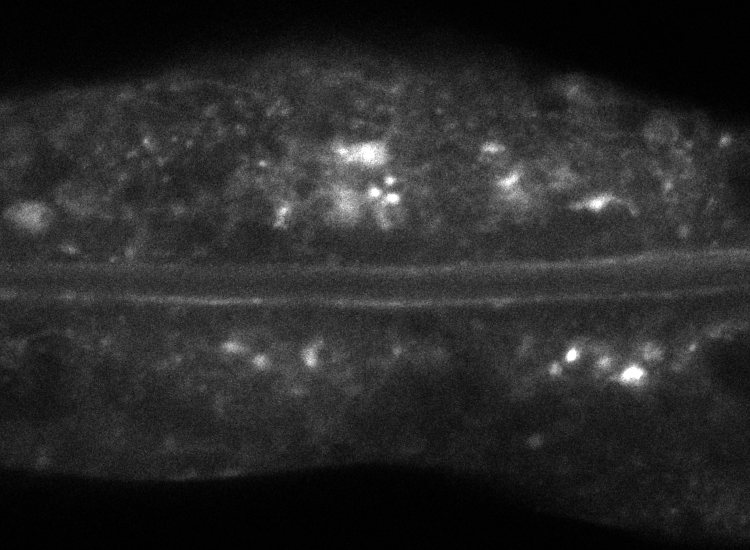

Supplement: Supplementary file 5 — Source data Fig. 3 [file 44318_2025_367_MOESM5_ESM.zip › SD figure 3/3A/Fig_3_A_Roi/Mock/Gut/RFP GFPvps27 RFPrab5 control RNAi front mz final setting_0010 aligned-ART-2.tif]

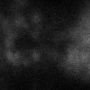

Supplement: Supplementary file 5 — Source data Fig. 3 [file 44318_2025_367_MOESM5_ESM.zip › SD figure 3/3A/Fig_3_A_Roi/vps-24 (RNAi)/Gut close up/RFP C R GFPvps27 RFPrab5 vps24 RNAi front_0010-ART-1-1.tif]

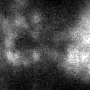

Supplement: Supplementary file 5 — Source data Fig. 3 [file 44318_2025_367_MOESM5_ESM.zip › SD figure 3/3A/Fig_3_A_Roi/vps-24 (RNAi)/Gut close up/Merge C MGRA GFPvps27 RFPrab5 vps24 RNAi front_0010-ART-1.tif]

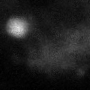

Supplement: Supplementary file 5 — Source data Fig. 3 [file 44318_2025_367_MOESM5_ESM.zip › SD figure 3/3A/Fig_3_A_Roi/vps-24 (RNAi)/Gut close up/RFP C2 R GFPvps27 RFPrab5 vps24 RNAi front_0010-ART-1-1.tif]

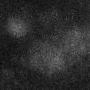

Supplement: Supplementary file 5 — Source data Fig. 3 [file 44318_2025_367_MOESM5_ESM.zip › SD figure 3/3A/Fig_3_A_Roi/vps-24 (RNAi)/Gut close up/GFP C2 G GFPvps27 RFPrab5 vps24 RNAi front_0010-ART-1-1.tif]

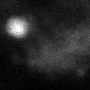

Supplement: Supplementary file 5 — Source data Fig. 3 [file 44318_2025_367_MOESM5_ESM.zip › SD figure 3/3A/Fig_3_A_Roi/vps-24 (RNAi)/Gut close up/Merge C2 MGRA GFPvps27 RFPrab5 vps24 RNAi front_0010-ART-1.tif]

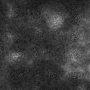

Supplement: Supplementary file 5 — Source data Fig. 3 [file 44318_2025_367_MOESM5_ESM.zip › SD figure 3/3A/Fig_3_A_Roi/vps-24 (RNAi)/Gut close up/GFP C G GFPvps27 RFPrab5 vps24 RNAi front_0010-ART-1-1.tif]
