# Supplementary figures and images for: Coordination between ESCRT function and Rab conversion during endosome maturation (part 1 of 9)
Source: EMBO J. 2025 Feb 5;44(6):1574–607. doi: 10.1038/s44318-025-00367-7 (PMC11914609; doi:10.1038/s44318-025-00367-7)

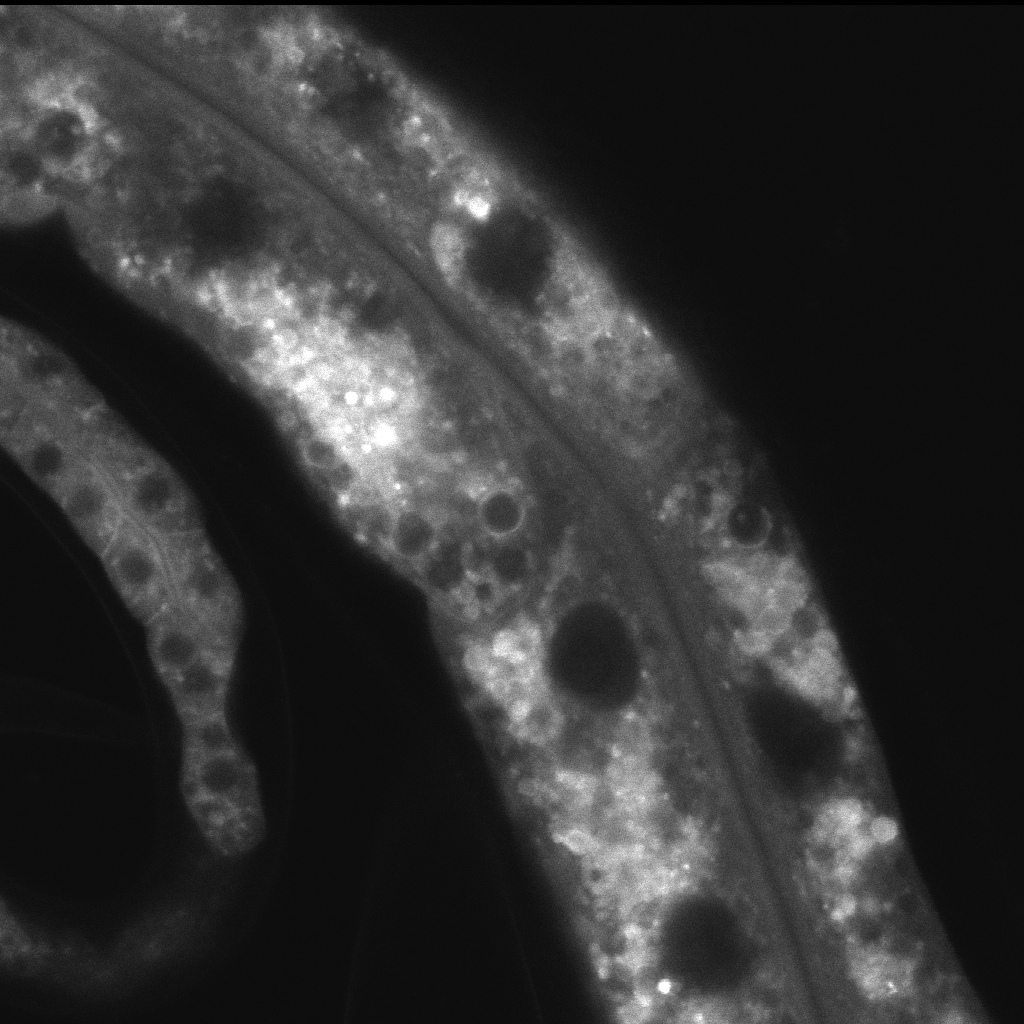

Supplement: Supplementary file 3 — Source data Fig. 1 [file 44318_2025_367_MOESM3_ESM.zip › SD figure 1 /1B/Fig_1_B_data/Mock/A rab5&rab7 sand1 control rnai front_0009-1.tif]

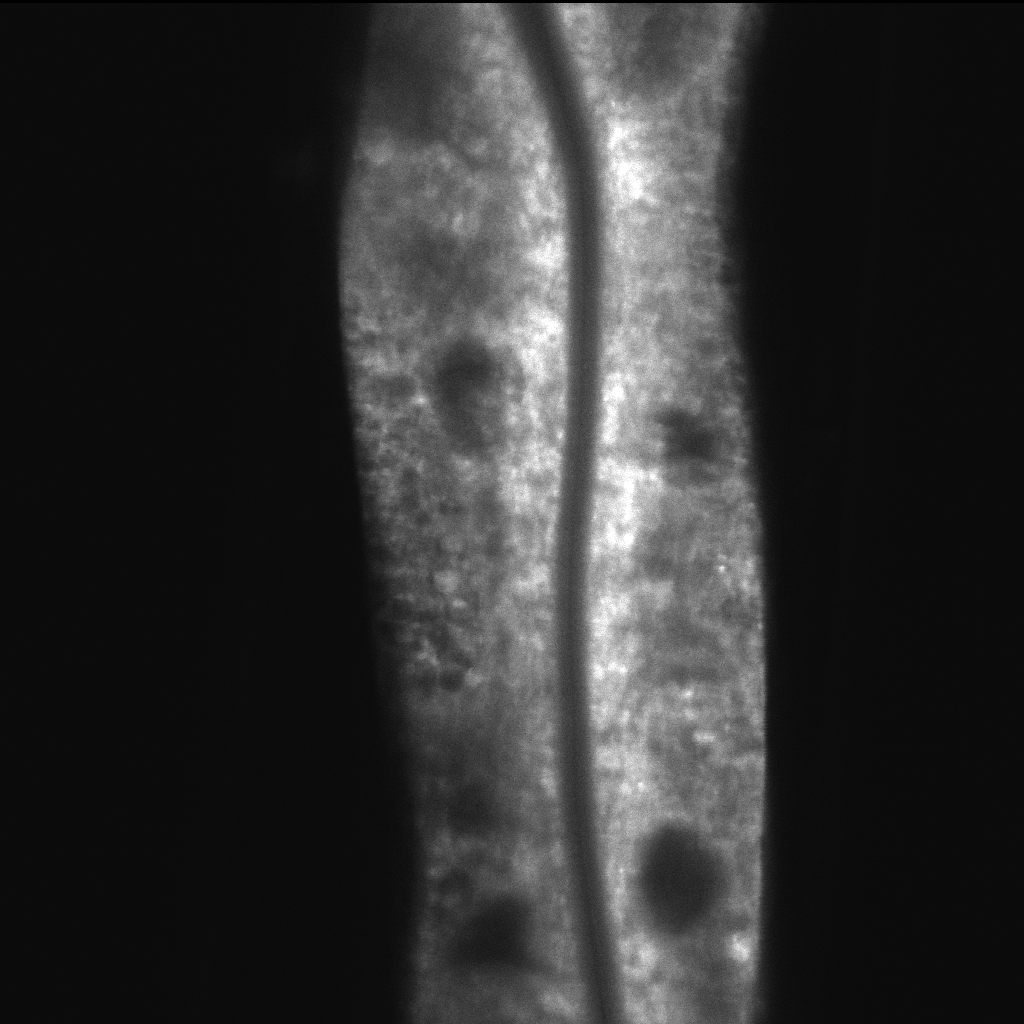

Supplement: Supplementary file 3 — Source data Fig. 1 [file 44318_2025_367_MOESM3_ESM.zip › SD figure 1 /1B/Fig_1_B_data/tsg-101 (RNAi) /A2 rab5 rab7 sand1 tsg101 rnai front_0001-1.tif]

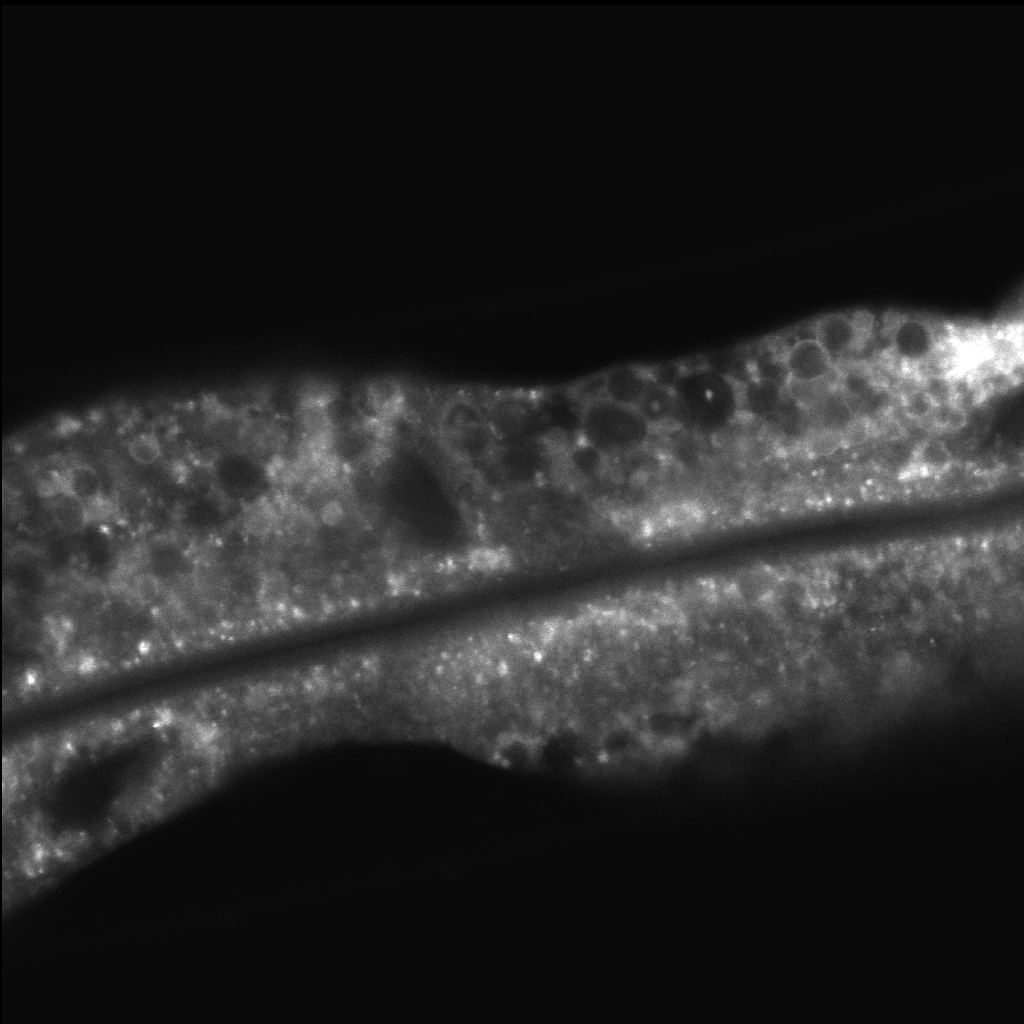

Supplement: Supplementary file 3 — Source data Fig. 1 [file 44318_2025_367_MOESM3_ESM.zip › SD figure 1 /1B/Fig_1_B_data/vps-20 (RNAi) /A Rab5&Rab7 sand1 vps20 RNAi front_0002-1.tif]

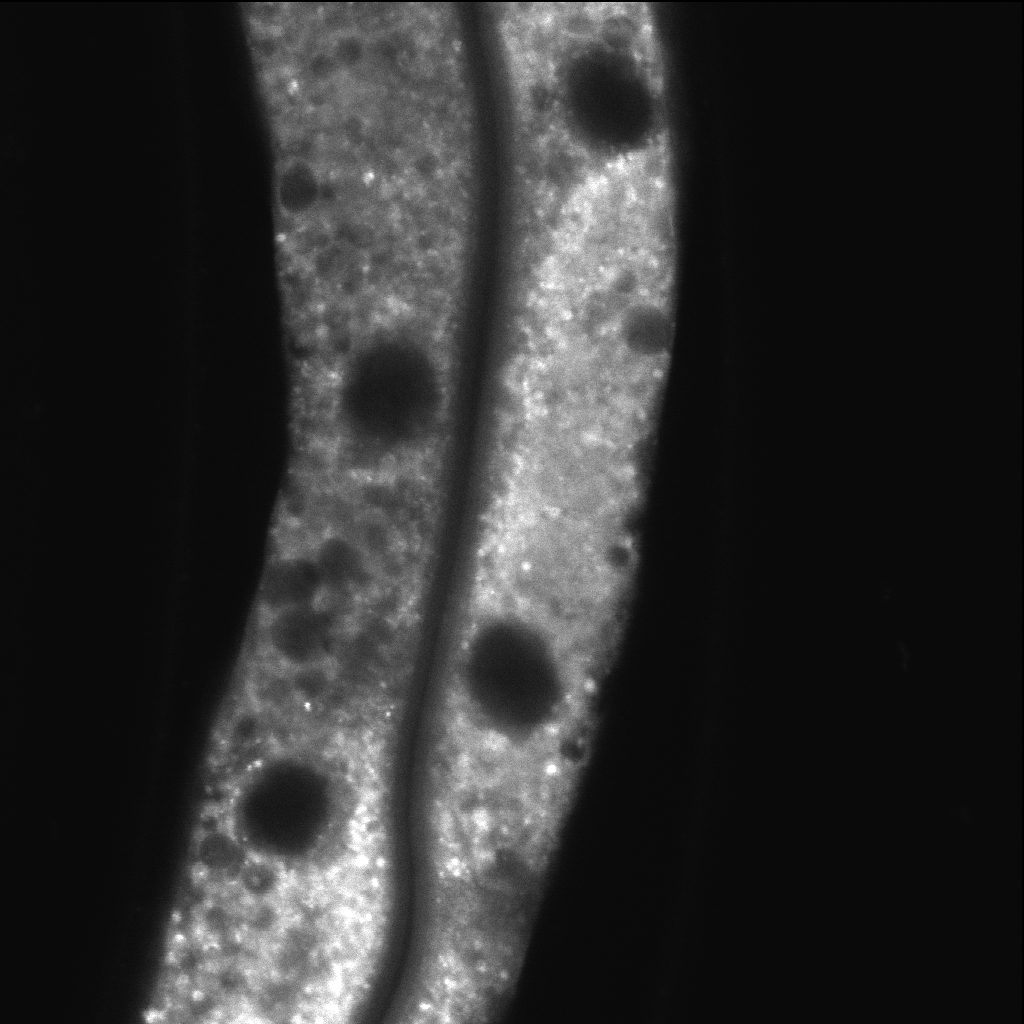

Supplement: Supplementary file 3 — Source data Fig. 1 [file 44318_2025_367_MOESM3_ESM.zip › SD figure 1 /1B/Fig_1_B_data/vps-2 (RNAi)/A rab5 rab7 sand1 vps 2rnai front-1.tif]

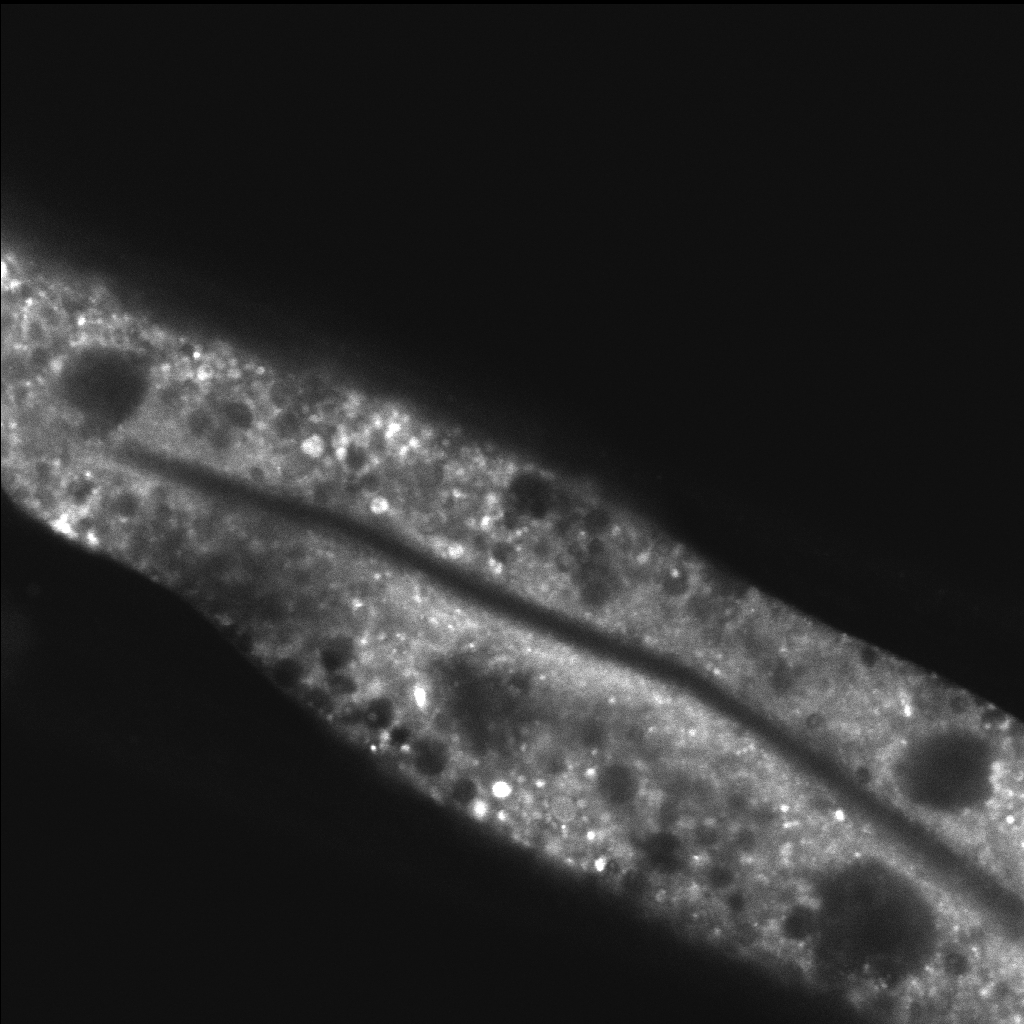

Supplement: Supplementary file 3 — Source data Fig. 1 [file 44318_2025_367_MOESM3_ESM.zip › SD figure 1 /1B/Fig_1_B_data/vps-4 (RNAi) pre fed/A rab5 raby sand1 vps4 rnai preefed front_0005-1-1.tif]

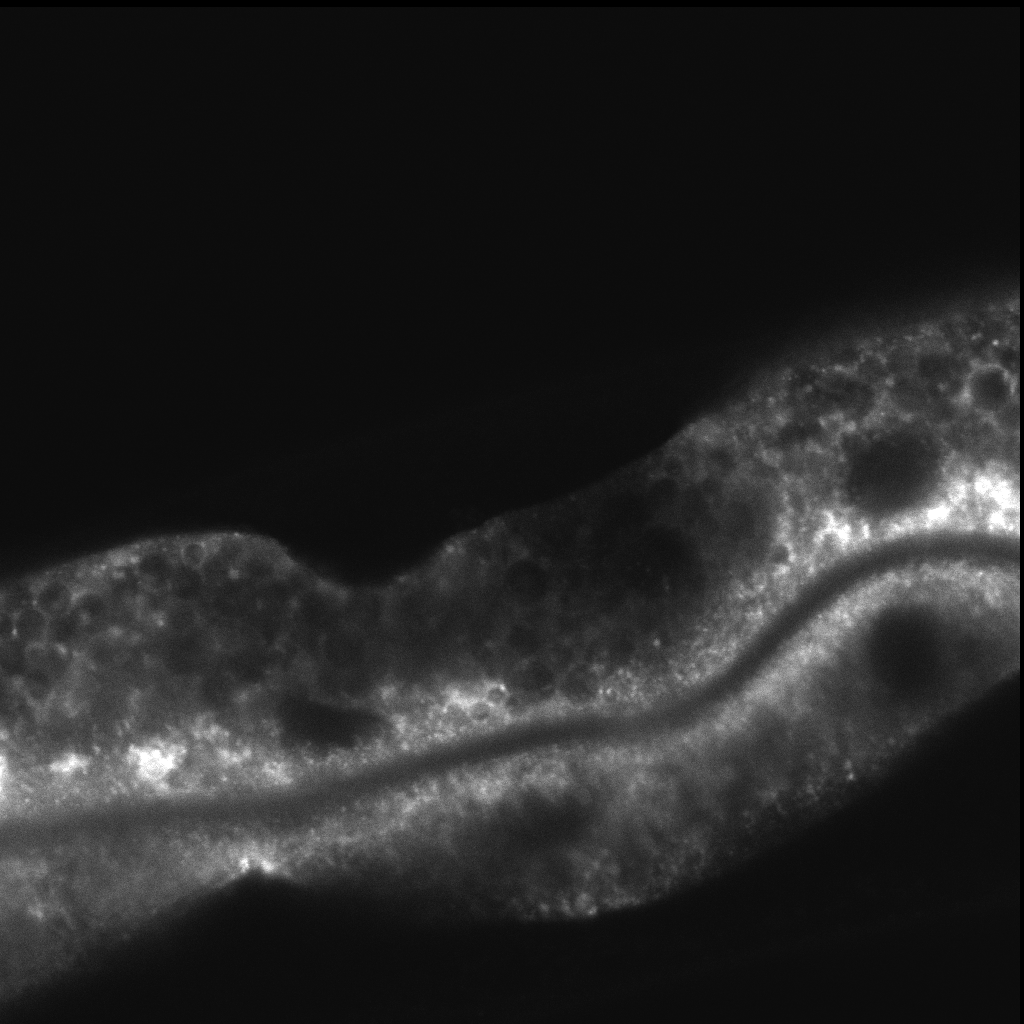

Supplement: Supplementary file 3 — Source data Fig. 1 [file 44318_2025_367_MOESM3_ESM.zip › SD figure 1 /1B/Fig_1_B_data/hgrs-1 (RNAi) pre fed/A rab5 rab7 sand1 pre fed vps27 rnai front_0005-1-1.tif]

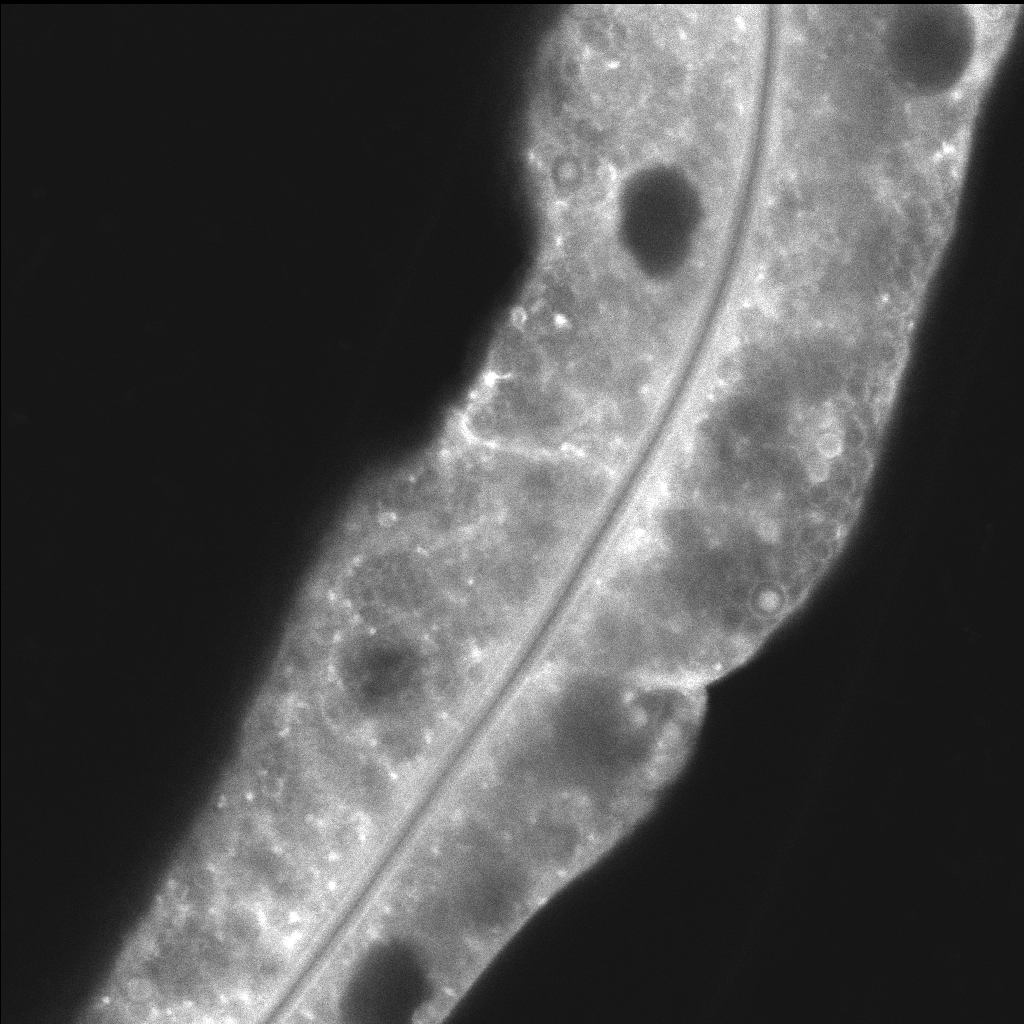

Supplement: Supplementary file 3 — Source data Fig. 1 [file 44318_2025_367_MOESM3_ESM.zip › SD figure 1 /1A/Fig_1_A_data/Mock/A rab5&rab7 control rnai front_0007-1.tif]

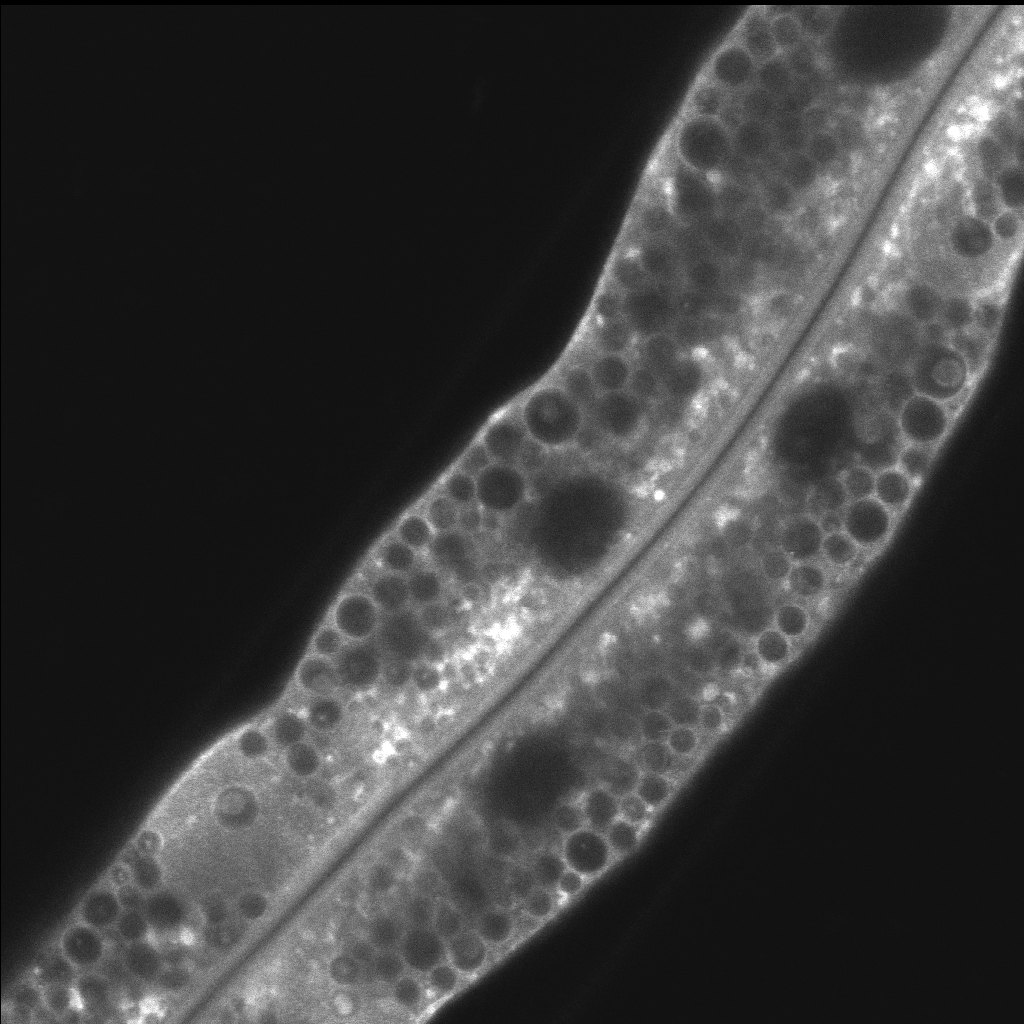

Supplement: Supplementary file 3 — Source data Fig. 1 [file 44318_2025_367_MOESM3_ESM.zip › SD figure 1 /1A/Fig_1_A_data/hgrs-1 (RNAi)/A rab5&rab7 vps27 rnai front_0012-1.tif]

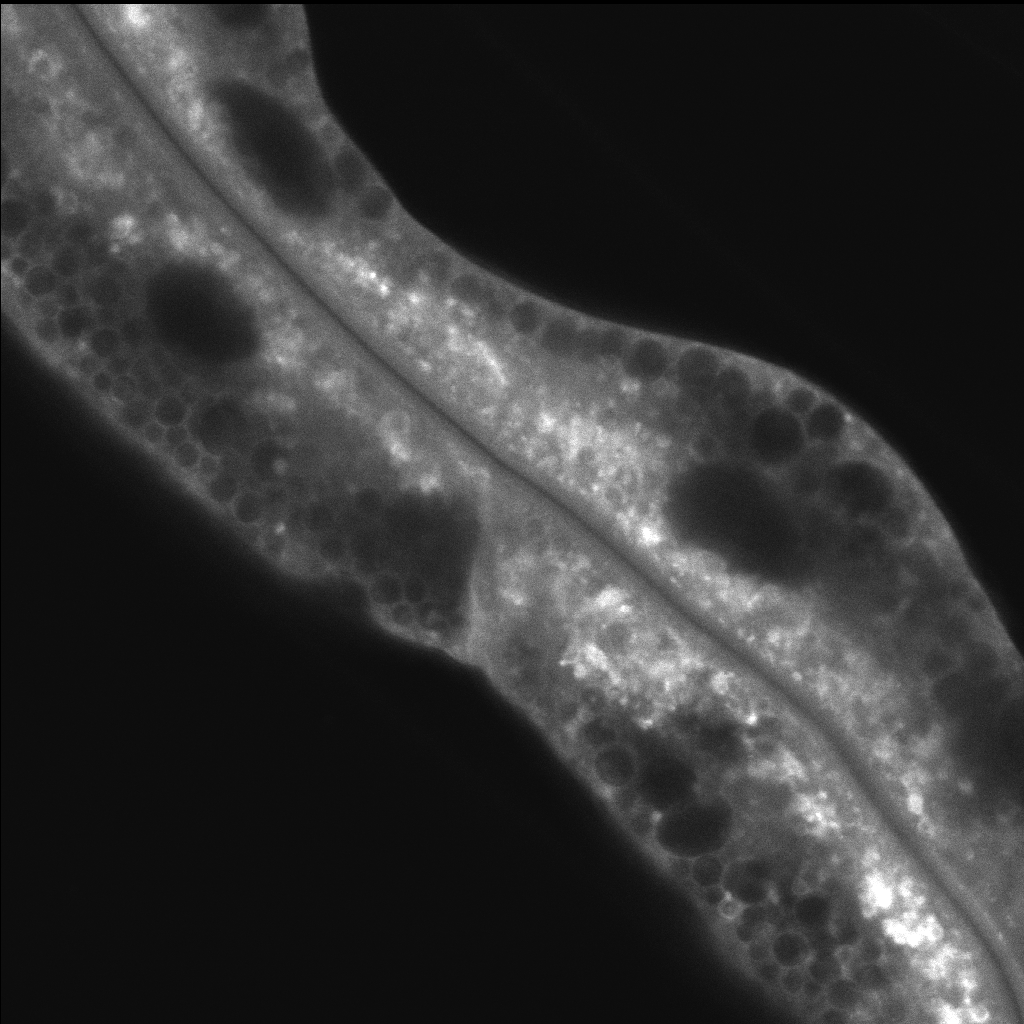

Supplement: Supplementary file 3 — Source data Fig. 1 [file 44318_2025_367_MOESM3_ESM.zip › SD figure 1 /1A/Fig_1_A_data/tsg-101 (RNAi)/A rab5 rab7 tsg101 short rnai front_0008-1.tif]

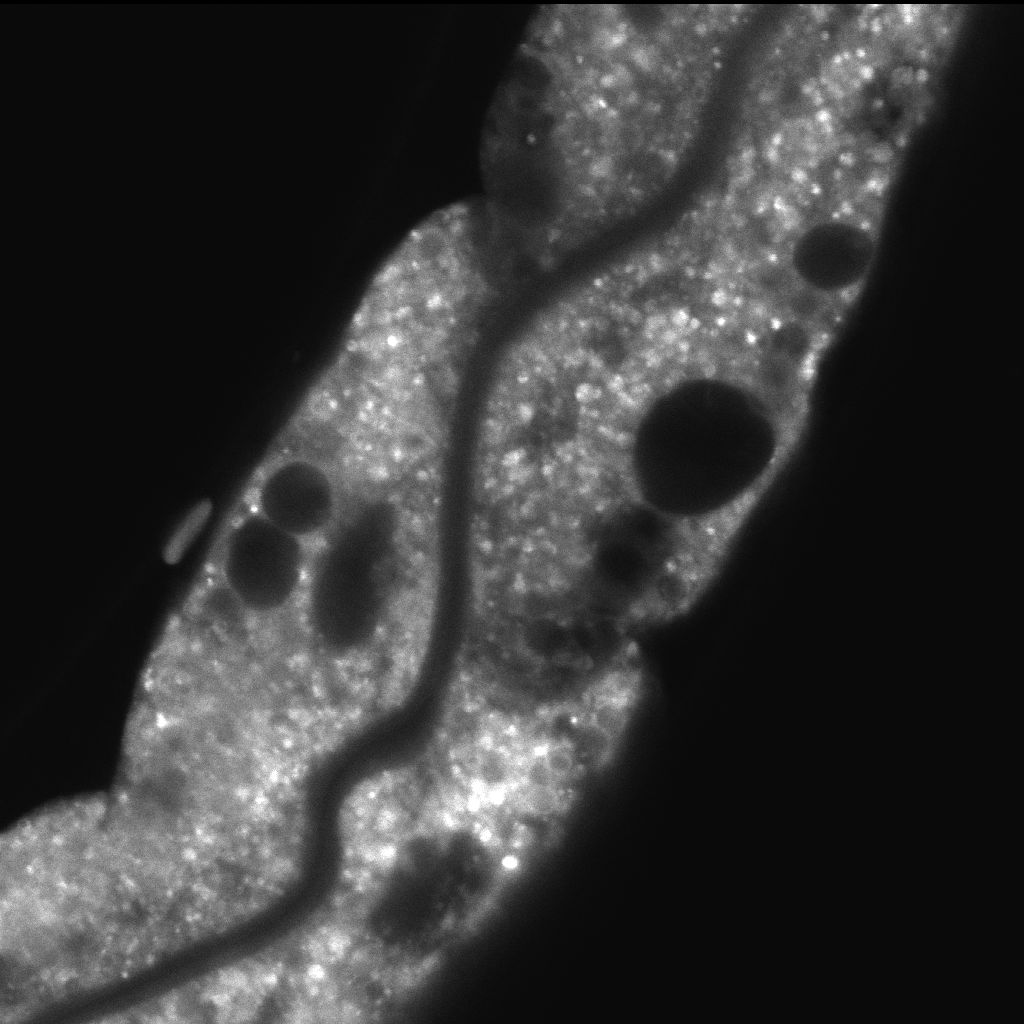

Supplement: Supplementary file 3 — Source data Fig. 1 [file 44318_2025_367_MOESM3_ESM.zip › SD figure 1 /1A/Fig_1_A_data/vps-2 (RNAi)/A rab5 rab7 vps2 rnai front_0009-1.tif]

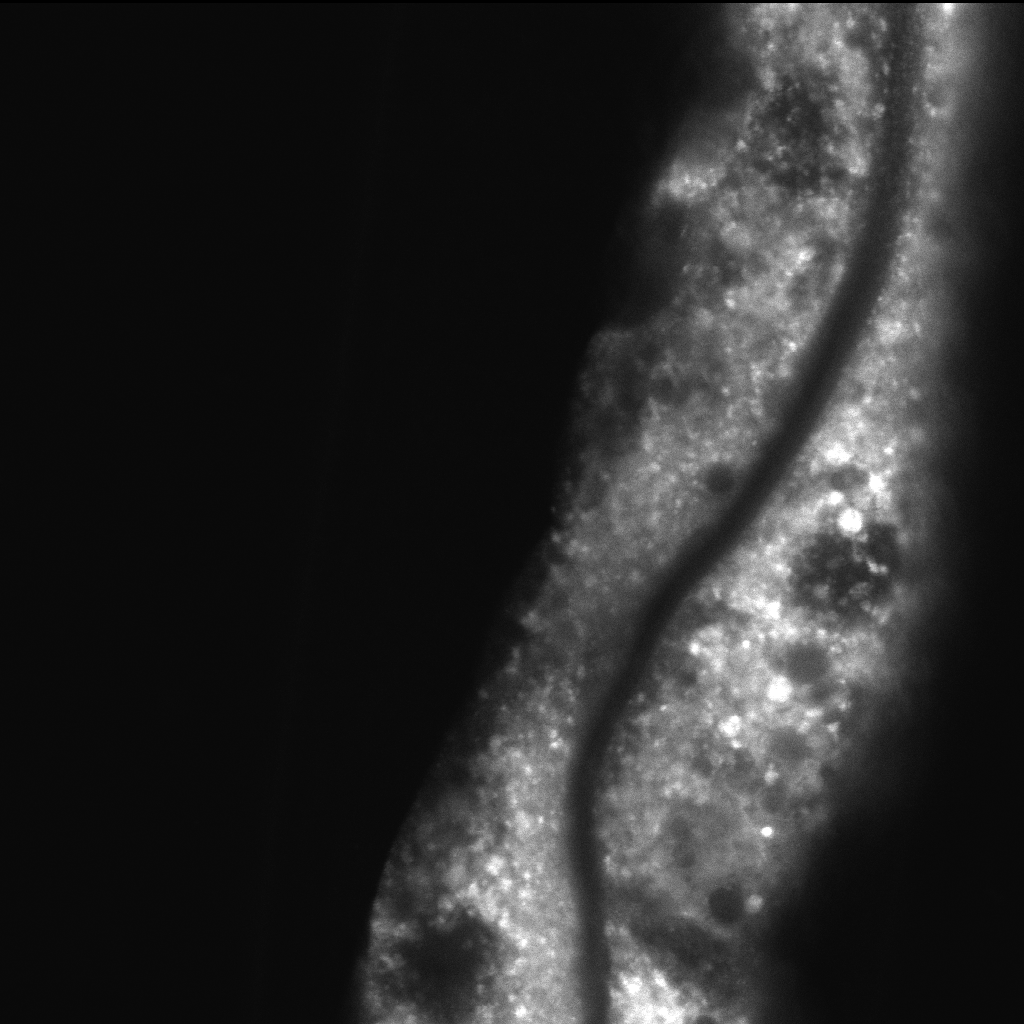

Supplement: Supplementary file 3 — Source data Fig. 1 [file 44318_2025_367_MOESM3_ESM.zip › SD figure 1 /1A/Fig_1_A_data/vps-4 (RNAi)/A rab5 rab7 vps4 rnai front_0001-1-1.tif]

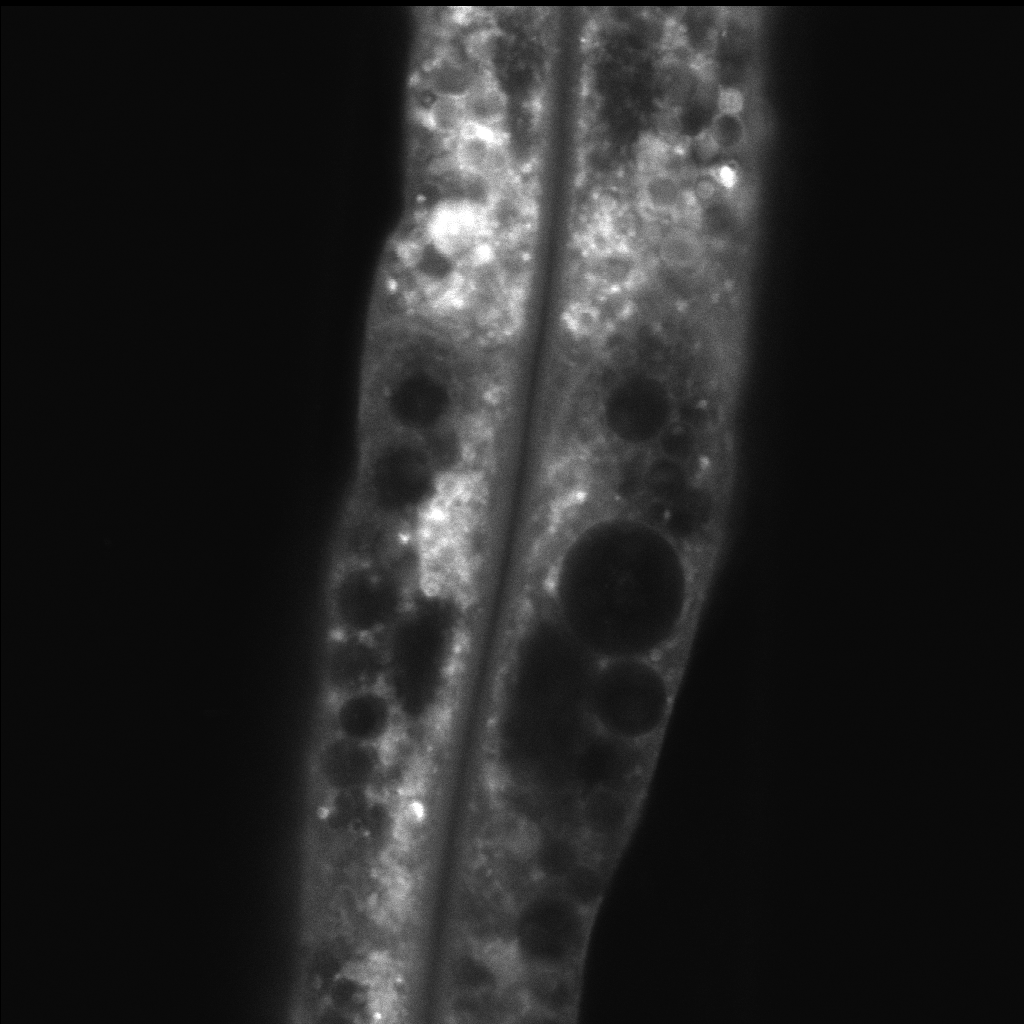

Supplement: Supplementary file 3 — Source data Fig. 1 [file 44318_2025_367_MOESM3_ESM.zip › SD figure 1 /1A/Fig_1_A_data/vps-20 (RNAi)/A rab 5 rab7 vps20 rnai front_0007-1.tif]

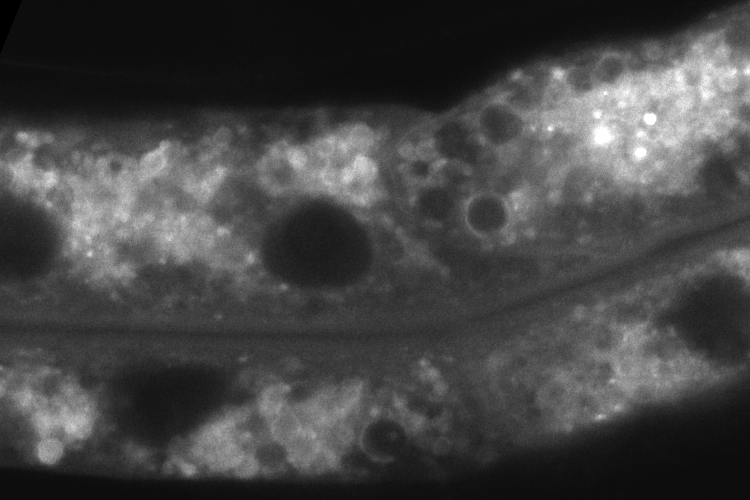

Supplement: Supplementary file 3 — Source data Fig. 1 [file 44318_2025_367_MOESM3_ESM.zip › SD figure 1 /1B/Fig_1_B_Roi/Mock/Gut /GFP ART G rab5&rab7 sand1 control rnai front_0009-1-1-1-1.tif]

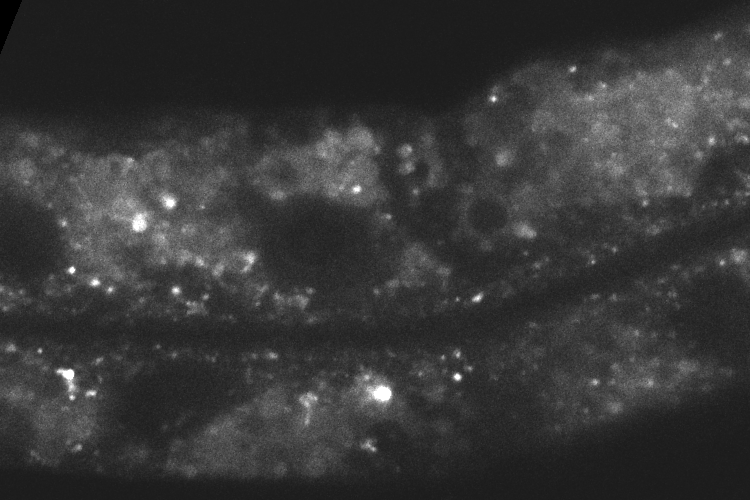

Supplement: Supplementary file 3 — Source data Fig. 1 [file 44318_2025_367_MOESM3_ESM.zip › SD figure 1 /1B/Fig_1_B_Roi/Mock/Gut /mCherry ART MC rab5&rab7 sand1 control rnai front_0009-1-1-1-1.tif]

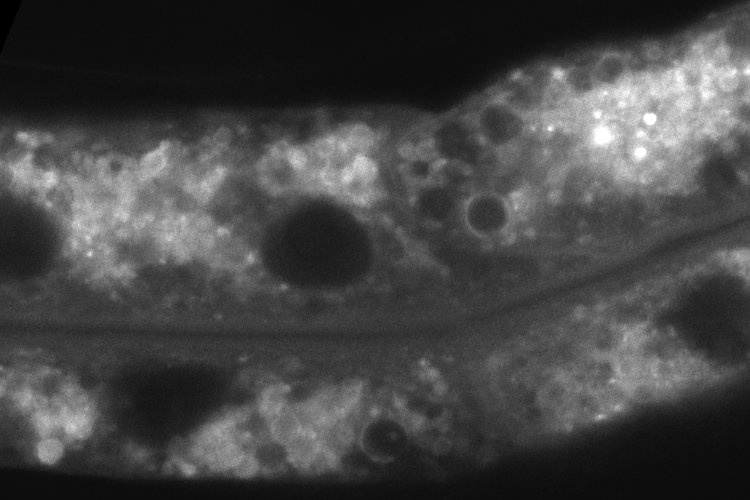

Supplement: Supplementary file 3 — Source data Fig. 1 [file 44318_2025_367_MOESM3_ESM.zip › SD figure 1 /1B/Fig_1_B_Roi/Mock/Gut /Merge ART MGM rab5&rab7 sand1 control rnai front_0009-1-1-1.tif]

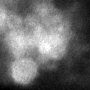

Supplement: Supplementary file 3 — Source data Fig. 1 [file 44318_2025_367_MOESM3_ESM.zip › SD figure 1 /1B/Fig_1_B_Roi/Mock/Gut close up/GFP ART C G rab5&rab7 sand1 control rnai front_0009-1-1-1-1-1.tif]

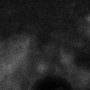

Supplement: Supplementary file 3 — Source data Fig. 1 [file 44318_2025_367_MOESM3_ESM.zip › SD figure 1 /1B/Fig_1_B_Roi/Mock/Gut close up/GFP ART C2 G rab5&rab7 sand1 control rnai front_0009-1-1-1-1-1.tif]

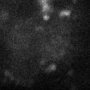

Supplement: Supplementary file 3 — Source data Fig. 1 [file 44318_2025_367_MOESM3_ESM.zip › SD figure 1 /1B/Fig_1_B_Roi/Mock/Gut close up/mCherry ART C MC rab5&rab7 sand1 control rnai front_0009-1-1-1-1-1.tif]

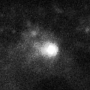

Supplement: Supplementary file 3 — Source data Fig. 1 [file 44318_2025_367_MOESM3_ESM.zip › SD figure 1 /1B/Fig_1_B_Roi/Mock/Gut close up/mCherry ART C2 MC rab5&rab7 sand1 control rnai front_0009-1-1-1-1-1.tif]

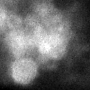

Supplement: Supplementary file 3 — Source data Fig. 1 [file 44318_2025_367_MOESM3_ESM.zip › SD figure 1 /1B/Fig_1_B_Roi/Mock/Gut close up/Merge ART C MGM rab5&rab7 sand1 control rnai front_0009-1-1-1-1.tif]

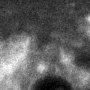

Supplement: Supplementary file 3 — Source data Fig. 1 [file 44318_2025_367_MOESM3_ESM.zip › SD figure 1 /1B/Fig_1_B_Roi/Mock/Gut close up/Merge ART C2 MGM rab5&rab7 sand1 control rnai front_0009-1-1-1-1.tif]

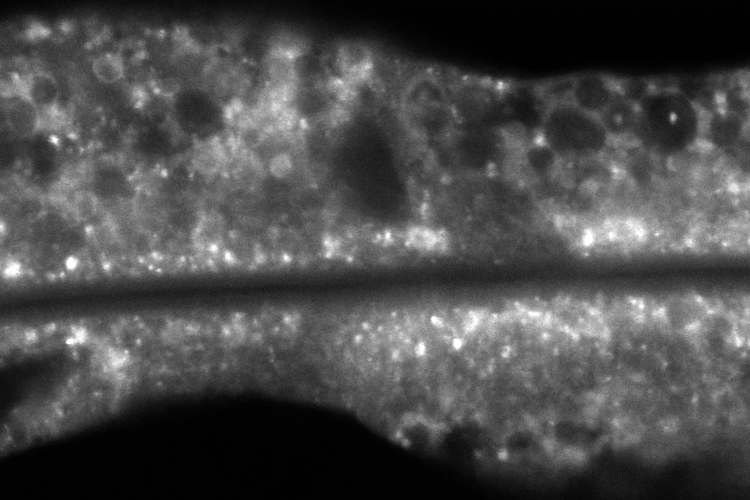

Supplement: Supplementary file 3 — Source data Fig. 1 [file 44318_2025_367_MOESM3_ESM.zip › SD figure 1 /1B/Fig_1_B_Roi/vps-20 (RNAi)/Gut /GFP ART G2 Rab5&Rab7 sand1 vps20 RNAi front_0002-1-1-1-1.tif]

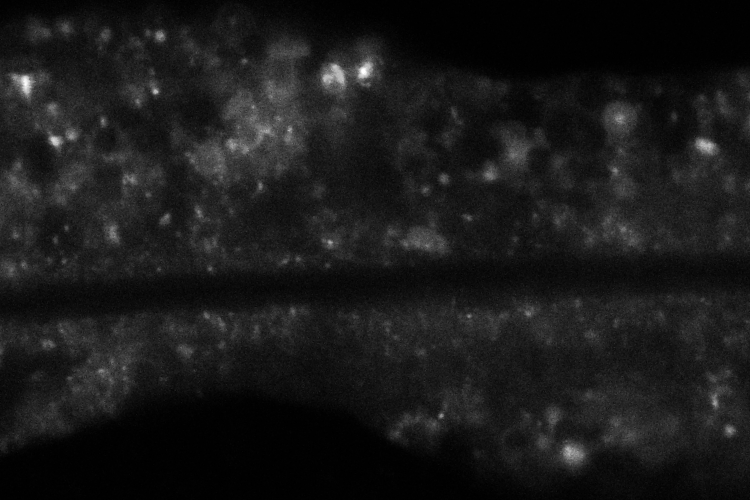

Supplement: Supplementary file 3 — Source data Fig. 1 [file 44318_2025_367_MOESM3_ESM.zip › SD figure 1 /1B/Fig_1_B_Roi/vps-20 (RNAi)/Gut /mCherry ART MC2 Rab5&Rab7 sand1 vps20 RNAi front_0002-1-1-1-1.tif]

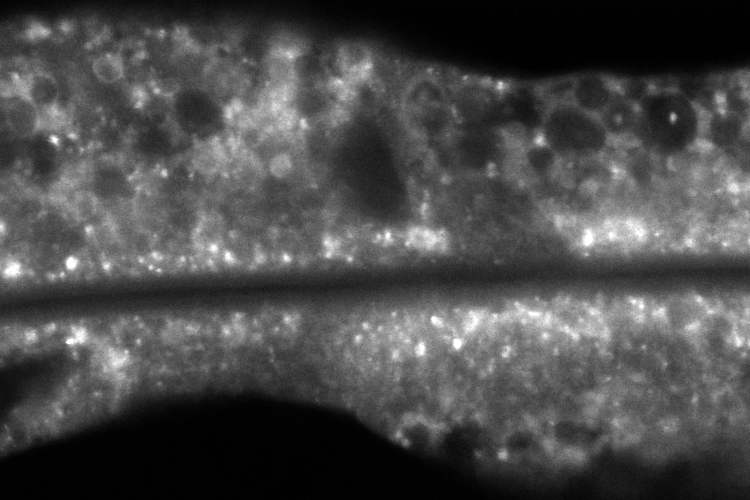

Supplement: Supplementary file 3 — Source data Fig. 1 [file 44318_2025_367_MOESM3_ESM.zip › SD figure 1 /1B/Fig_1_B_Roi/vps-20 (RNAi)/Gut /Merge ART MGM2 Rab5&Rab7 sand1 vps20 RNAi front_0002-1-1-1.tif]

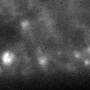

Supplement: Supplementary file 3 — Source data Fig. 1 [file 44318_2025_367_MOESM3_ESM.zip › SD figure 1 /1B/Fig_1_B_Roi/vps-20 (RNAi)/Gut close up/GFP ART C G2 Rab5&Rab7 sand1 vps20 RNAi front_0002-1-1-1-1-1.tif]

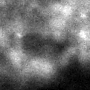

Supplement: Supplementary file 3 — Source data Fig. 1 [file 44318_2025_367_MOESM3_ESM.zip › SD figure 1 /1B/Fig_1_B_Roi/vps-20 (RNAi)/Gut close up/GFP ART C2 G Rab5&Rab7 sand1 vps20 RNAi front_0002-1-1-1-1-1.tif]

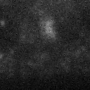

Supplement: Supplementary file 3 — Source data Fig. 1 [file 44318_2025_367_MOESM3_ESM.zip › SD figure 1 /1B/Fig_1_B_Roi/vps-20 (RNAi)/Gut close up/mCherry ART C MC2 Rab5&Rab7 sand1 vps20 RNAi front_0002-1-1-1-1-1.tif]

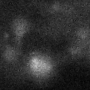

Supplement: Supplementary file 3 — Source data Fig. 1 [file 44318_2025_367_MOESM3_ESM.zip › SD figure 1 /1B/Fig_1_B_Roi/vps-20 (RNAi)/Gut close up/mCherry ART C2 MC Rab5&Rab7 sand1 vps20 RNAi front_0002-1-1-1-1-1.tif]

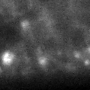

Supplement: Supplementary file 3 — Source data Fig. 1 [file 44318_2025_367_MOESM3_ESM.zip › SD figure 1 /1B/Fig_1_B_Roi/vps-20 (RNAi)/Gut close up/Merge ART C MGM2 Rab5&Rab7 sand1 vps20 RNAi front_0002-1-1-1-1.tif]

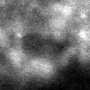

Supplement: Supplementary file 3 — Source data Fig. 1 [file 44318_2025_367_MOESM3_ESM.zip › SD figure 1 /1B/Fig_1_B_Roi/vps-20 (RNAi)/Gut close up/Merge ART C2 MGM Rab5&Rab7 sand1 vps20 RNAi front_0002-1-1-1-1.tif]

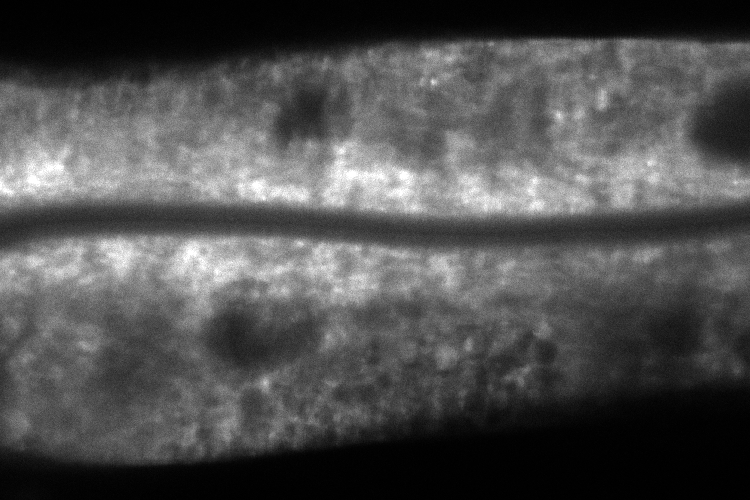

Supplement: Supplementary file 3 — Source data Fig. 1 [file 44318_2025_367_MOESM3_ESM.zip › SD figure 1 /1B/Fig_1_B_Roi/tsg-101 (RNAi)/Gut /GFP ART2 G rab5 rab7 sand1 tsg101 rnai front_0001-1-1-1-1.tif]

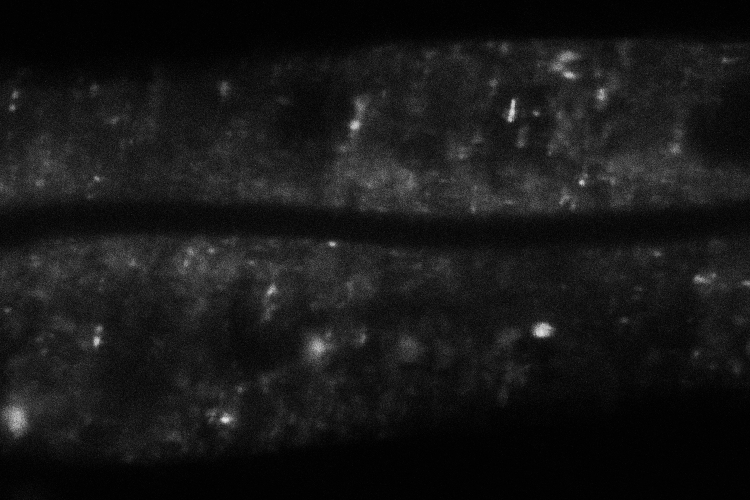

Supplement: Supplementary file 3 — Source data Fig. 1 [file 44318_2025_367_MOESM3_ESM.zip › SD figure 1 /1B/Fig_1_B_Roi/tsg-101 (RNAi)/Gut /mCherry ART2 MC rab5 rab7 sand1 tsg101 rnai front_0001-1-1-1-1.tif]

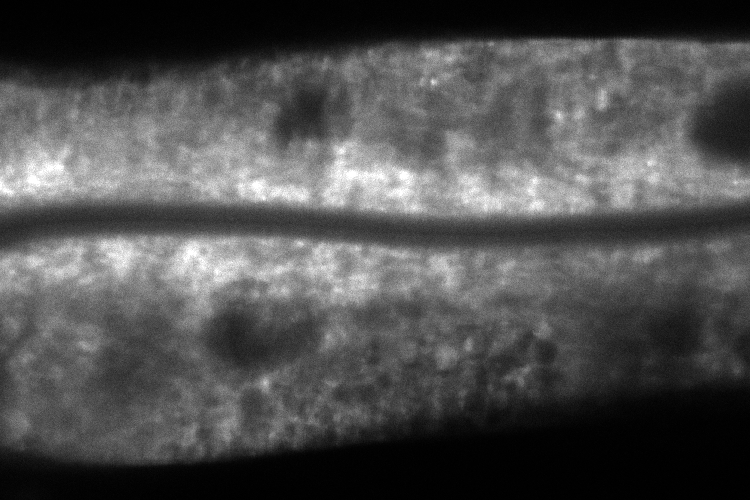

Supplement: Supplementary file 3 — Source data Fig. 1 [file 44318_2025_367_MOESM3_ESM.zip › SD figure 1 /1B/Fig_1_B_Roi/tsg-101 (RNAi)/Gut /Merge ART2 MGM rab5 rab7 sand1 tsg101 rnai front_0001-1-1-1.tif]

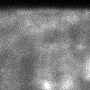

Supplement: Supplementary file 3 — Source data Fig. 1 [file 44318_2025_367_MOESM3_ESM.zip › SD figure 1 /1B/Fig_1_B_Roi/tsg-101 (RNAi)/Gut close up/GFP ART2 C G rab5 rab7 sand1 tsg101 rnai front_0001-1-1-1-1-1.tif]

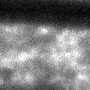

Supplement: Supplementary file 3 — Source data Fig. 1 [file 44318_2025_367_MOESM3_ESM.zip › SD figure 1 /1B/Fig_1_B_Roi/tsg-101 (RNAi)/Gut close up/GFP ART2 C2 G rab5 rab7 sand1 tsg101 rnai front_0001-1-1-1-1-1.tif]

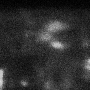

Supplement: Supplementary file 3 — Source data Fig. 1 [file 44318_2025_367_MOESM3_ESM.zip › SD figure 1 /1B/Fig_1_B_Roi/tsg-101 (RNAi)/Gut close up/mCherry ART2 C MC rab5 rab7 sand1 tsg101 rnai front_0001-1-1-1-1-1.tif]

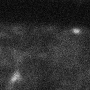

Supplement: Supplementary file 3 — Source data Fig. 1 [file 44318_2025_367_MOESM3_ESM.zip › SD figure 1 /1B/Fig_1_B_Roi/tsg-101 (RNAi)/Gut close up/mCherry ART2 C2 MC rab5 rab7 sand1 tsg101 rnai front_0001-1-1-1-1-1.tif]

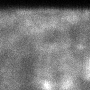

Supplement: Supplementary file 3 — Source data Fig. 1 [file 44318_2025_367_MOESM3_ESM.zip › SD figure 1 /1B/Fig_1_B_Roi/tsg-101 (RNAi)/Gut close up/Merge ART2 C MGM rab5 rab7 sand1 tsg101 rnai front_0001-1-1-1-1.tif]

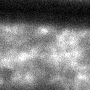

Supplement: Supplementary file 3 — Source data Fig. 1 [file 44318_2025_367_MOESM3_ESM.zip › SD figure 1 /1B/Fig_1_B_Roi/tsg-101 (RNAi)/Gut close up/Merge ART2 C2 MGM rab5 rab7 sand1 tsg101 rnai front_0001-1-1-1-1.tif]

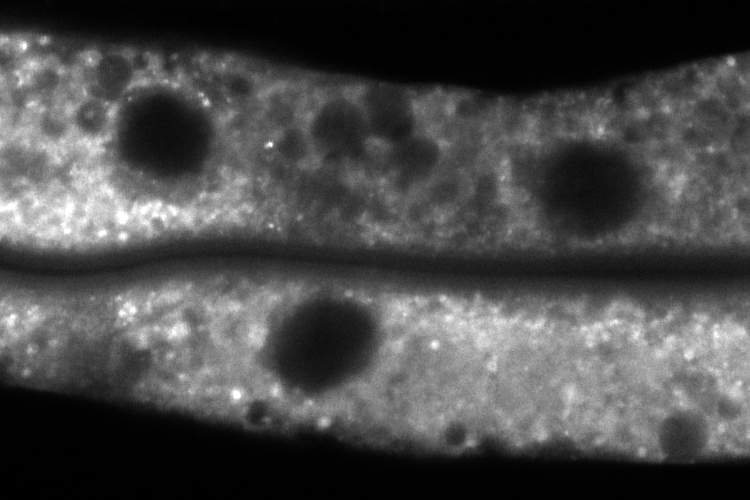

Supplement: Supplementary file 3 — Source data Fig. 1 [file 44318_2025_367_MOESM3_ESM.zip › SD figure 1 /1B/Fig_1_B_Roi/vps-2 (RNAi)/Gut /GFP ART G rab5 rab7 sand1 vps 2rnai front-1-1-1-1.tif]

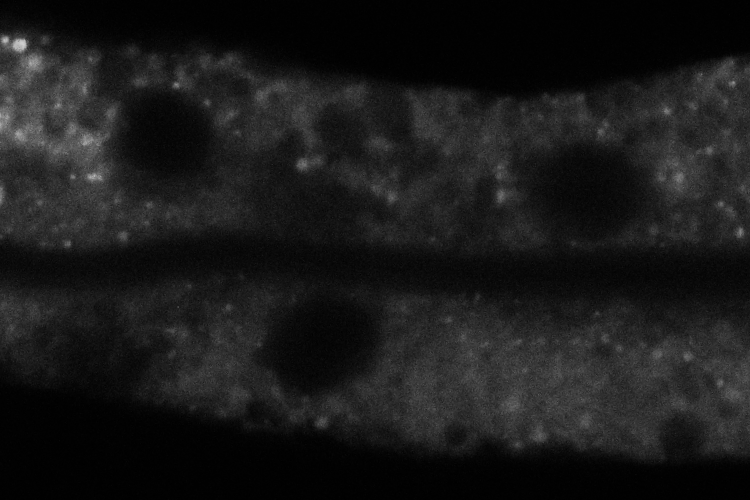

Supplement: Supplementary file 3 — Source data Fig. 1 [file 44318_2025_367_MOESM3_ESM.zip › SD figure 1 /1B/Fig_1_B_Roi/vps-2 (RNAi)/Gut /mCherry ART MC rab5 rab7 sand1 vps 2rnai front-1-1-1-1.tif]

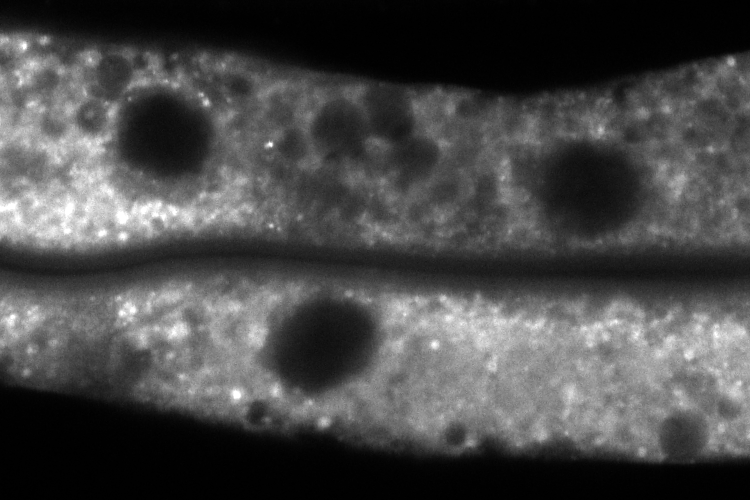

Supplement: Supplementary file 3 — Source data Fig. 1 [file 44318_2025_367_MOESM3_ESM.zip › SD figure 1 /1B/Fig_1_B_Roi/vps-2 (RNAi)/Gut /Merge ART MGM rab5 rab7 sand1 vps 2rnai front-1-1-1.tif]

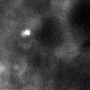

Supplement: Supplementary file 3 — Source data Fig. 1 [file 44318_2025_367_MOESM3_ESM.zip › SD figure 1 /1B/Fig_1_B_Roi/vps-2 (RNAi)/Gut close up/GFP ART C G rab5 rab7 sand1 vps 2rnai front-1-1-1-1-1.tif]

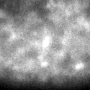

Supplement: Supplementary file 3 — Source data Fig. 1 [file 44318_2025_367_MOESM3_ESM.zip › SD figure 1 /1B/Fig_1_B_Roi/vps-2 (RNAi)/Gut close up/GFP ART C2 G rab5 rab7 sand1 vps 2rnai front-1-1-1-1-1.tif]

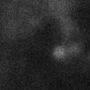

Supplement: Supplementary file 3 — Source data Fig. 1 [file 44318_2025_367_MOESM3_ESM.zip › SD figure 1 /1B/Fig_1_B_Roi/vps-2 (RNAi)/Gut close up/mCherry ART C MC rab5 rab7 sand1 vps 2rnai front-1-1-1-1-1.tif]

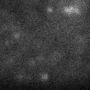

Supplement: Supplementary file 3 — Source data Fig. 1 [file 44318_2025_367_MOESM3_ESM.zip › SD figure 1 /1B/Fig_1_B_Roi/vps-2 (RNAi)/Gut close up/mCherry ART C2 MC rab5 rab7 sand1 vps 2rnai front-1-1-1-1-1.tif]

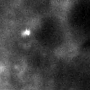

Supplement: Supplementary file 3 — Source data Fig. 1 [file 44318_2025_367_MOESM3_ESM.zip › SD figure 1 /1B/Fig_1_B_Roi/vps-2 (RNAi)/Gut close up/Merge ART C MGM rab5 rab7 sand1 vps 2rnai front-1-1-1-1.tif]

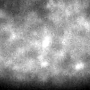

Supplement: Supplementary file 3 — Source data Fig. 1 [file 44318_2025_367_MOESM3_ESM.zip › SD figure 1 /1B/Fig_1_B_Roi/vps-2 (RNAi)/Gut close up/Merge ART C2 MGM rab5 rab7 sand1 vps 2rnai front-1-1-1-1.tif]

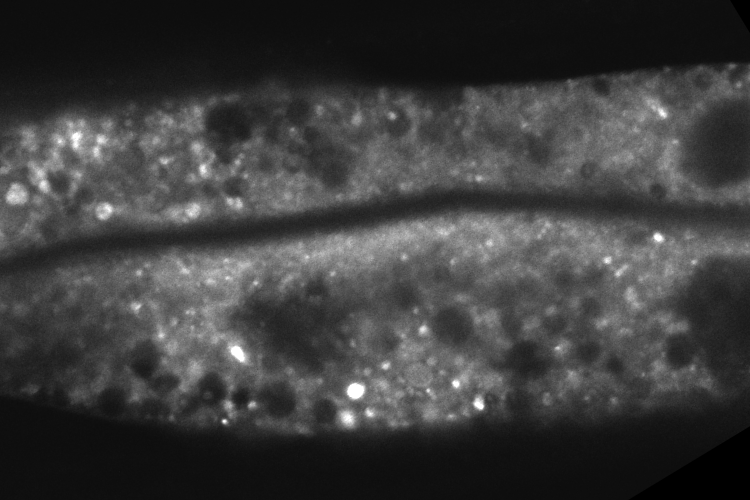

Supplement: Supplementary file 3 — Source data Fig. 1 [file 44318_2025_367_MOESM3_ESM.zip › SD figure 1 /1B/Fig_1_B_Roi/vps-4 (RNAi) pre fed/Gut /GFP ART G rab5 raby sand1 vps4 rnai preefed front_0005-1-1-1-1-1.tif]

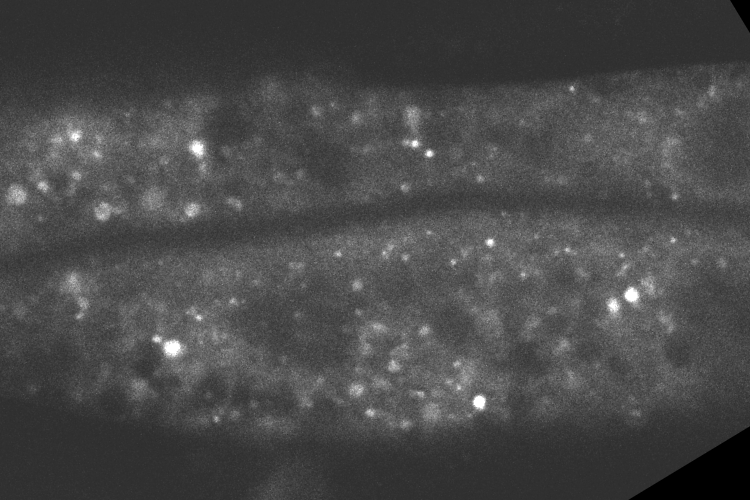

Supplement: Supplementary file 3 — Source data Fig. 1 [file 44318_2025_367_MOESM3_ESM.zip › SD figure 1 /1B/Fig_1_B_Roi/vps-4 (RNAi) pre fed/Gut /mCherry ART MC rab5 raby sand1 vps4 rnai preefed front_0005-1-1-1-1-1.tif]

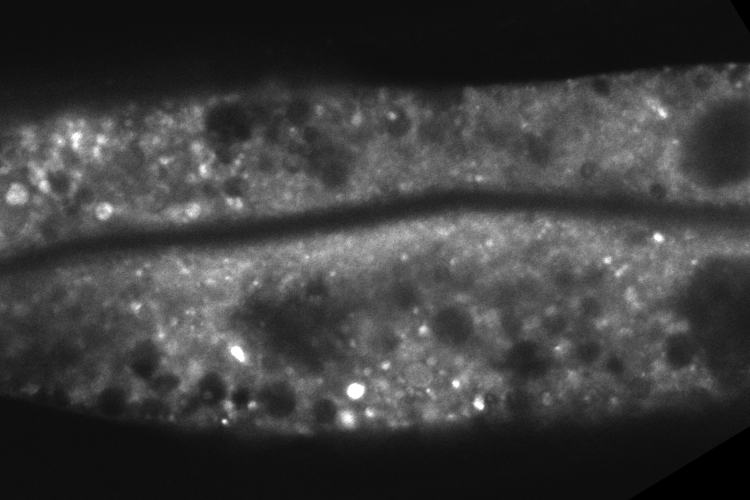

Supplement: Supplementary file 3 — Source data Fig. 1 [file 44318_2025_367_MOESM3_ESM.zip › SD figure 1 /1B/Fig_1_B_Roi/vps-4 (RNAi) pre fed/Gut /Merge ART MGM rab5 raby sand1 vps4 rnai preefed front_0005-1-1-1-1.tif]

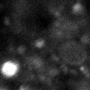

Supplement: Supplementary file 3 — Source data Fig. 1 [file 44318_2025_367_MOESM3_ESM.zip › SD figure 1 /1B/Fig_1_B_Roi/vps-4 (RNAi) pre fed/Gut close up/GFP ART C G rab5 raby sand1 vps4 rnai preefed front_0005-1-1-1-1-1-1.tif]

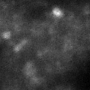

Supplement: Supplementary file 3 — Source data Fig. 1 [file 44318_2025_367_MOESM3_ESM.zip › SD figure 1 /1B/Fig_1_B_Roi/vps-4 (RNAi) pre fed/Gut close up/GFP ART C2 G rab5 raby sand1 vps4 rnai preefed front_0005-1-1-1-1-1-1.tif]

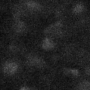

Supplement: Supplementary file 3 — Source data Fig. 1 [file 44318_2025_367_MOESM3_ESM.zip › SD figure 1 /1B/Fig_1_B_Roi/vps-4 (RNAi) pre fed/Gut close up/mCherry ART C MC rab5 raby sand1 vps4 rnai preefed front_0005-1-1-1-1-1-1.tif]

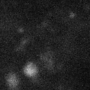

Supplement: Supplementary file 3 — Source data Fig. 1 [file 44318_2025_367_MOESM3_ESM.zip › SD figure 1 /1B/Fig_1_B_Roi/vps-4 (RNAi) pre fed/Gut close up/mCherry ART C2 MC rab5 raby sand1 vps4 rnai preefed front_0005-1-1-1-1-1-1.tif]

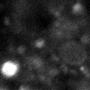

Supplement: Supplementary file 3 — Source data Fig. 1 [file 44318_2025_367_MOESM3_ESM.zip › SD figure 1 /1B/Fig_1_B_Roi/vps-4 (RNAi) pre fed/Gut close up/Merge ART C MGM rab5 raby sand1 vps4 rnai preefed front_0005-1-1-1-1-1.tif]

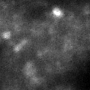

Supplement: Supplementary file 3 — Source data Fig. 1 [file 44318_2025_367_MOESM3_ESM.zip › SD figure 1 /1B/Fig_1_B_Roi/vps-4 (RNAi) pre fed/Gut close up/Merge ART C2 MGM rab5 raby sand1 vps4 rnai preefed front_0005-1-1-1-1-1.tif]

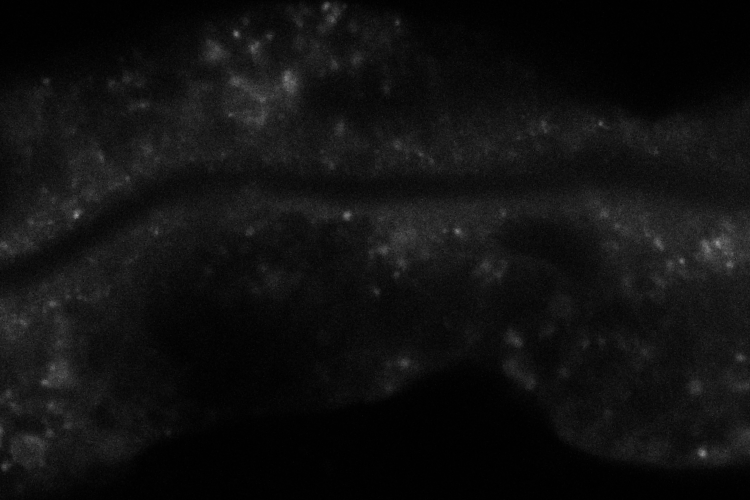

Supplement: Supplementary file 3 — Source data Fig. 1 [file 44318_2025_367_MOESM3_ESM.zip › SD figure 1 /1B/Fig_1_B_Roi/hgrs-1 (RNAi) pre fed/Gut /mCherry ART MC rab5 rab7 sand1 pre fed vps27 rnai front_0005-1-1-1-1-1.tif]

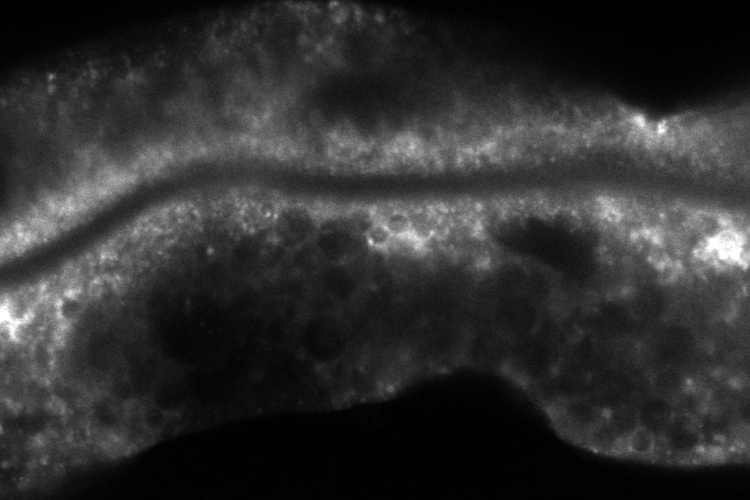

Supplement: Supplementary file 3 — Source data Fig. 1 [file 44318_2025_367_MOESM3_ESM.zip › SD figure 1 /1B/Fig_1_B_Roi/hgrs-1 (RNAi) pre fed/Gut /GFP ART G rab5 rab7 sand1 pre fed vps27 rnai front_0005-1-1-1-1-1.tif]

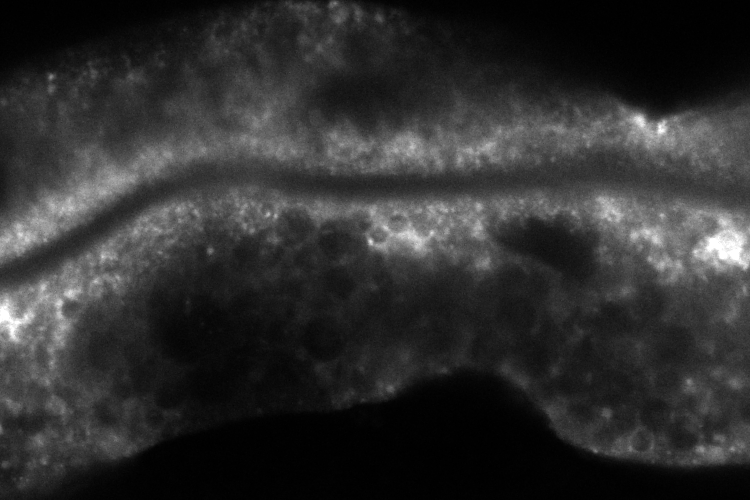

Supplement: Supplementary file 3 — Source data Fig. 1 [file 44318_2025_367_MOESM3_ESM.zip › SD figure 1 /1B/Fig_1_B_Roi/hgrs-1 (RNAi) pre fed/Gut /Merge ART MGM rab5 rab7 sand1 pre fed vps27 rnai front_0005-1-1-1-1.tif]

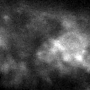

Supplement: Supplementary file 3 — Source data Fig. 1 [file 44318_2025_367_MOESM3_ESM.zip › SD figure 1 /1B/Fig_1_B_Roi/hgrs-1 (RNAi) pre fed/Gut close up/GFP ART C G rab5 rab7 sand1 pre fed vps27 rnai front_0005-1-1-1-1-1-1.tif]

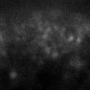

Supplement: Supplementary file 3 — Source data Fig. 1 [file 44318_2025_367_MOESM3_ESM.zip › SD figure 1 /1B/Fig_1_B_Roi/hgrs-1 (RNAi) pre fed/Gut close up/GFP ART C2 G rab5 rab7 sand1 pre fed vps27 rnai front_0005-1-1-1-1-1-1.tif]

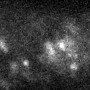

Supplement: Supplementary file 3 — Source data Fig. 1 [file 44318_2025_367_MOESM3_ESM.zip › SD figure 1 /1B/Fig_1_B_Roi/hgrs-1 (RNAi) pre fed/Gut close up/mCherry ART C MC rab5 rab7 sand1 pre fed vps27 rnai front_0005-1-1-1-1-1-1.tif]

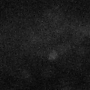

Supplement: Supplementary file 3 — Source data Fig. 1 [file 44318_2025_367_MOESM3_ESM.zip › SD figure 1 /1B/Fig_1_B_Roi/hgrs-1 (RNAi) pre fed/Gut close up/mCherry ART C2 MC rab5 rab7 sand1 pre fed vps27 rnai front_0005-1-1-1-1-1-1.tif]

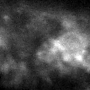

Supplement: Supplementary file 3 — Source data Fig. 1 [file 44318_2025_367_MOESM3_ESM.zip › SD figure 1 /1B/Fig_1_B_Roi/hgrs-1 (RNAi) pre fed/Gut close up/Merge ART C MGM rab5 rab7 sand1 pre fed vps27 rnai front_0005-1-1-1-1-1.tif]

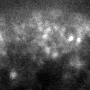

Supplement: Supplementary file 3 — Source data Fig. 1 [file 44318_2025_367_MOESM3_ESM.zip › SD figure 1 /1B/Fig_1_B_Roi/hgrs-1 (RNAi) pre fed/Gut close up/Merge ART C2 MGM rab5 rab7 sand1 pre fed vps27 rnai front_0005-1-1-1-1-1.tif]

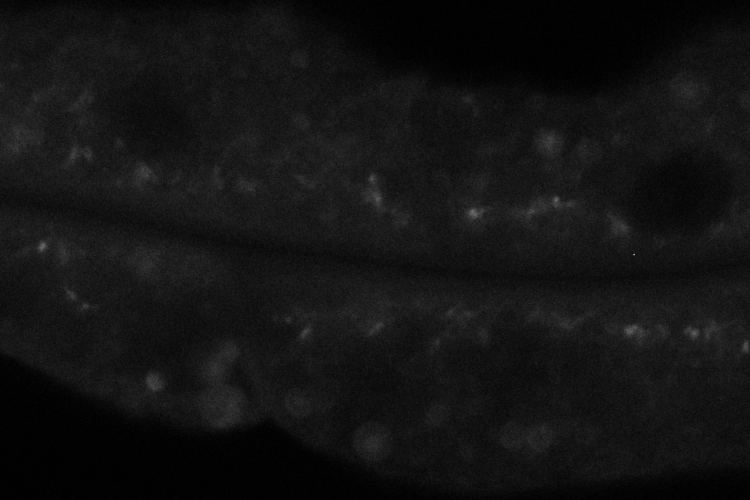

Supplement: Supplementary file 3 — Source data Fig. 1 [file 44318_2025_367_MOESM3_ESM.zip › SD figure 1 /1A/Fig_1_A_Roi/Mock/Gut/mCherry ART MC rab5&rab7 control rnai front_0007-1-1-1-1.tif]

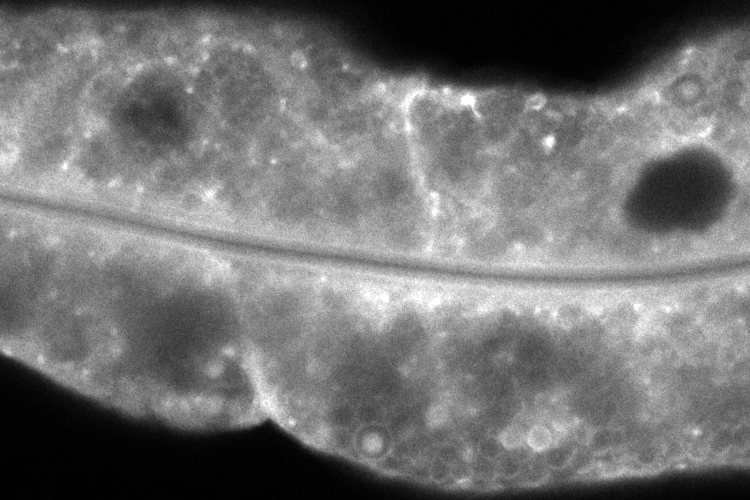

Supplement: Supplementary file 3 — Source data Fig. 1 [file 44318_2025_367_MOESM3_ESM.zip › SD figure 1 /1A/Fig_1_A_Roi/Mock/Gut/GFP ART G rab5&rab7 control rnai front_0007-1-1-1-1.tif]

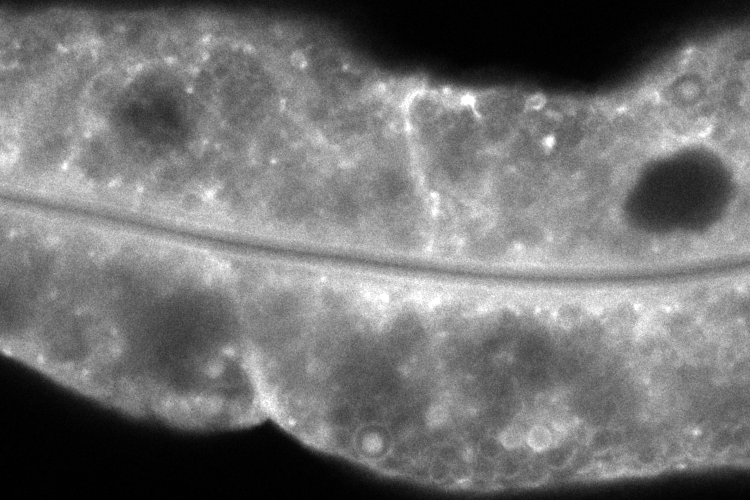

Supplement: Supplementary file 3 — Source data Fig. 1 [file 44318_2025_367_MOESM3_ESM.zip › SD figure 1 /1A/Fig_1_A_Roi/Mock/Gut/Merge ART MGM rab5&rab7 control rnai front_0007-1-1-1.tif]

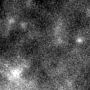

Supplement: Supplementary file 3 — Source data Fig. 1 [file 44318_2025_367_MOESM3_ESM.zip › SD figure 1 /1A/Fig_1_A_Roi/Mock/Gut close up/Merge ART C2 MGM rab5&rab7 control rnai front_0007-1-1-1-1.tif]

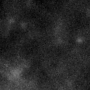

Supplement: Supplementary file 3 — Source data Fig. 1 [file 44318_2025_367_MOESM3_ESM.zip › SD figure 1 /1A/Fig_1_A_Roi/Mock/Gut close up/GFP ART C2 G rab5&rab7 control rnai front_0007-1-1-1-1-1.tif]

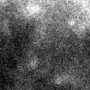

Supplement: Supplementary file 3 — Source data Fig. 1 [file 44318_2025_367_MOESM3_ESM.zip › SD figure 1 /1A/Fig_1_A_Roi/Mock/Gut close up/Merge ART C MGM rab5&rab7 control rnai front_0007-1-1-1-1.tif]

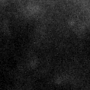

Supplement: Supplementary file 3 — Source data Fig. 1 [file 44318_2025_367_MOESM3_ESM.zip › SD figure 1 /1A/Fig_1_A_Roi/Mock/Gut close up/GFP ART C G rab5&rab7 control rnai front_0007-1-1-1-1-1.tif]

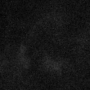

Supplement: Supplementary file 3 — Source data Fig. 1 [file 44318_2025_367_MOESM3_ESM.zip › SD figure 1 /1A/Fig_1_A_Roi/Mock/Gut close up/mCherry ART C2 MC rab5&rab7 control rnai front_0007-1-1-1-1-1.tif]

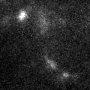

Supplement: Supplementary file 3 — Source data Fig. 1 [file 44318_2025_367_MOESM3_ESM.zip › SD figure 1 /1A/Fig_1_A_Roi/Mock/Gut close up/mCherry ART C MC rab5&rab7 control rnai front_0007-1-1-1-1-1.tif]

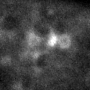

Supplement: Supplementary file 3 — Source data Fig. 1 [file 44318_2025_367_MOESM3_ESM.zip › SD figure 1 /1A/Fig_1_A_Roi/hgrs-1 (RNAi)/Gut close up/Merge ART C2 MGM rab5&rab7 vps27 rnai front_0012-1-1-1-1.tif]

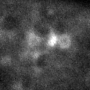

Supplement: Supplementary file 3 — Source data Fig. 1 [file 44318_2025_367_MOESM3_ESM.zip › SD figure 1 /1A/Fig_1_A_Roi/hgrs-1 (RNAi)/Gut close up/GFP ART C2 G rab5&rab7 vps27 rnai front_0012-1-1-1-1-1.tif]

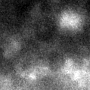

Supplement: Supplementary file 3 — Source data Fig. 1 [file 44318_2025_367_MOESM3_ESM.zip › SD figure 1 /1A/Fig_1_A_Roi/hgrs-1 (RNAi)/Gut close up/Merge ART C MGM rab5&rab7 vps27 rnai front_0012-1-1-1-1.tif]

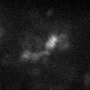

Supplement: Supplementary file 3 — Source data Fig. 1 [file 44318_2025_367_MOESM3_ESM.zip › SD figure 1 /1A/Fig_1_A_Roi/hgrs-1 (RNAi)/Gut close up/mCherry ART C2 MC rab5&rab7 vps27 rnai front_0012-1-1-1-1-1.tif]

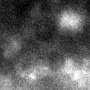

Supplement: Supplementary file 3 — Source data Fig. 1 [file 44318_2025_367_MOESM3_ESM.zip › SD figure 1 /1A/Fig_1_A_Roi/hgrs-1 (RNAi)/Gut close up/GFP ART C G rab5&rab7 vps27 rnai front_0012-1-1-1-1-1.tif]

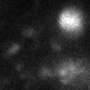

Supplement: Supplementary file 3 — Source data Fig. 1 [file 44318_2025_367_MOESM3_ESM.zip › SD figure 1 /1A/Fig_1_A_Roi/hgrs-1 (RNAi)/Gut close up/mCherry ART C MC rab5&rab7 vps27 rnai front_0012-1-1-1-1-1.tif]

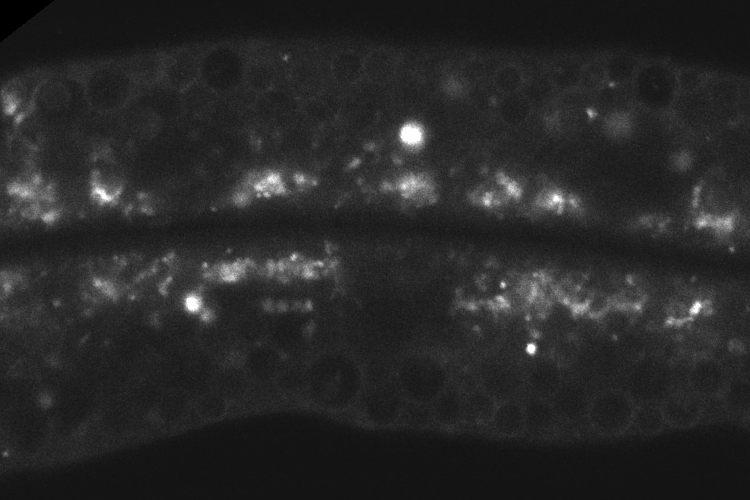

Supplement: Supplementary file 3 — Source data Fig. 1 [file 44318_2025_367_MOESM3_ESM.zip › SD figure 1 /1A/Fig_1_A_Roi/hgrs-1 (RNAi)/Gut/mCherry ART MC rab5&rab7 vps27 rnai front_0012-1-1-1-1.tif]

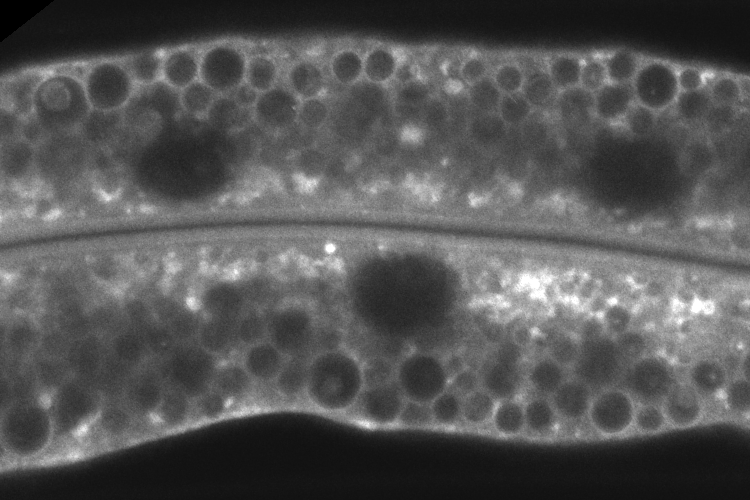

Supplement: Supplementary file 3 — Source data Fig. 1 [file 44318_2025_367_MOESM3_ESM.zip › SD figure 1 /1A/Fig_1_A_Roi/hgrs-1 (RNAi)/Gut/GFP ART G rab5&rab7 vps27 rnai front_0012-1-1-1-1.tif]

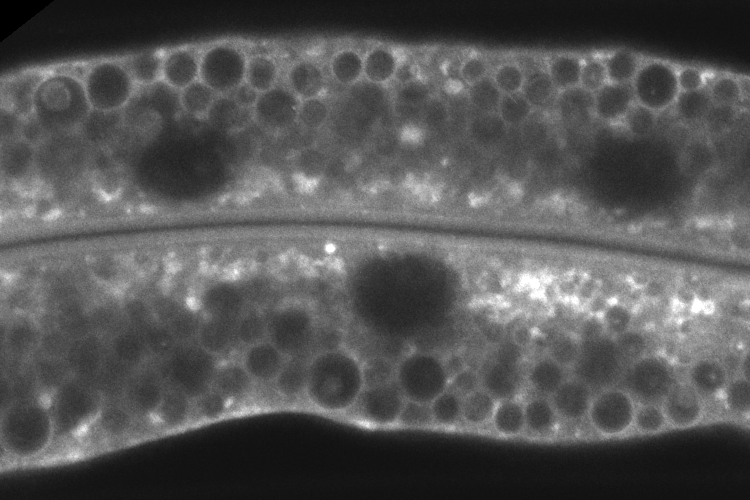

Supplement: Supplementary file 3 — Source data Fig. 1 [file 44318_2025_367_MOESM3_ESM.zip › SD figure 1 /1A/Fig_1_A_Roi/hgrs-1 (RNAi)/Gut/Merge ART MGM rab5&rab7 vps27 rnai front_0012-1-1-1.tif]

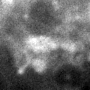

Supplement: Supplementary file 3 — Source data Fig. 1 [file 44318_2025_367_MOESM3_ESM.zip › SD figure 1 /1A/Fig_1_A_Roi/tsg-101 (RNAi)/Gut close up/GFP ART C2 G rab5 rab7 tsg101 short rnai front_0008-1-1-1-1-1.tif]

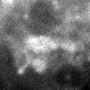

Supplement: Supplementary file 3 — Source data Fig. 1 [file 44318_2025_367_MOESM3_ESM.zip › SD figure 1 /1A/Fig_1_A_Roi/tsg-101 (RNAi)/Gut close up/Merge ART C2 MGM rab5 rab7 tsg101 short rnai front_0008-1-1-1-1.tif]

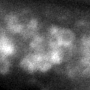

Supplement: Supplementary file 3 — Source data Fig. 1 [file 44318_2025_367_MOESM3_ESM.zip › SD figure 1 /1A/Fig_1_A_Roi/tsg-101 (RNAi)/Gut close up/Merge ART C MGM rab5 rab7 tsg101 short rnai front_0008-1-1-1-1.tif]

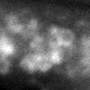

Supplement: Supplementary file 3 — Source data Fig. 1 [file 44318_2025_367_MOESM3_ESM.zip › SD figure 1 /1A/Fig_1_A_Roi/tsg-101 (RNAi)/Gut close up/GFP ART C G rab5 rab7 tsg101 short rnai front_0008-1-1-1-1-1.tif]

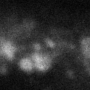

Supplement: Supplementary file 3 — Source data Fig. 1 [file 44318_2025_367_MOESM3_ESM.zip › SD figure 1 /1A/Fig_1_A_Roi/tsg-101 (RNAi)/Gut close up/mCherry ART C MC rab5 rab7 tsg101 short rnai front_0008-1-1-1-1-1.tif]

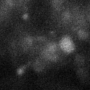

Supplement: Supplementary file 3 — Source data Fig. 1 [file 44318_2025_367_MOESM3_ESM.zip › SD figure 1 /1A/Fig_1_A_Roi/tsg-101 (RNAi)/Gut close up/mCherry ART C2 MC rab5 rab7 tsg101 short rnai front_0008-1-1-1-1-1.tif]

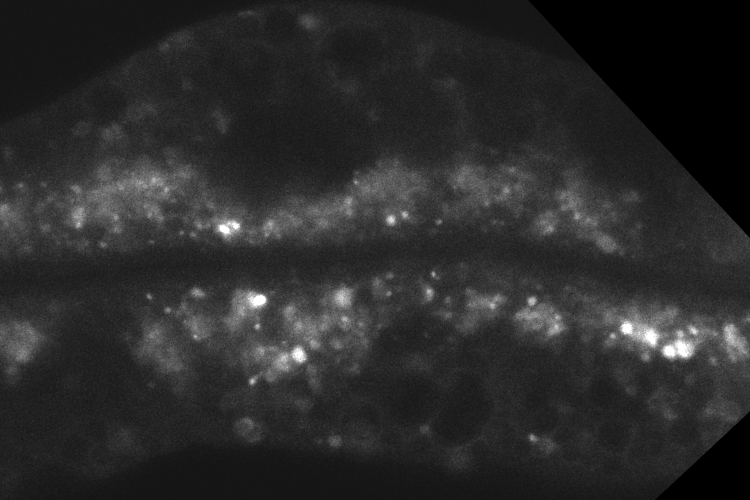

Supplement: Supplementary file 3 — Source data Fig. 1 [file 44318_2025_367_MOESM3_ESM.zip › SD figure 1 /1A/Fig_1_A_Roi/tsg-101 (RNAi)/Gut/mCherry ART MC rab5 rab7 tsg101 short rnai front_0008-1-1-1-1.tif]

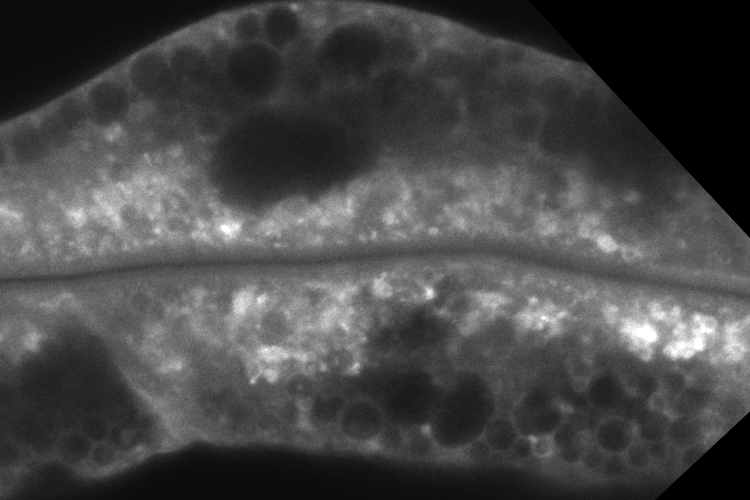

Supplement: Supplementary file 3 — Source data Fig. 1 [file 44318_2025_367_MOESM3_ESM.zip › SD figure 1 /1A/Fig_1_A_Roi/tsg-101 (RNAi)/Gut/GFP ART G rab5 rab7 tsg101 short rnai front_0008-1-1-1-1.tif]

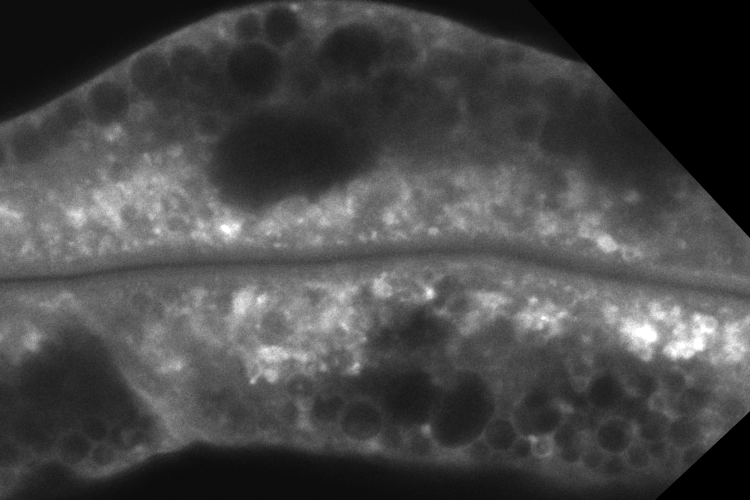

Supplement: Supplementary file 3 — Source data Fig. 1 [file 44318_2025_367_MOESM3_ESM.zip › SD figure 1 /1A/Fig_1_A_Roi/tsg-101 (RNAi)/Gut/Merge ART MGM rab5 rab7 tsg101 short rnai front_0008-1-1-1.tif]

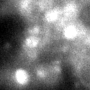

Supplement: Supplementary file 3 — Source data Fig. 1 [file 44318_2025_367_MOESM3_ESM.zip › SD figure 1 /1A/Fig_1_A_Roi/vps-2 (RNAi)/Gut close up/Merge ART C2 MGM rab5 rab7 vps2 rnai front_0009-1-1-1-1.tif]

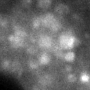

Supplement: Supplementary file 3 — Source data Fig. 1 [file 44318_2025_367_MOESM3_ESM.zip › SD figure 1 /1A/Fig_1_A_Roi/vps-2 (RNAi)/Gut close up/Merge ART C MGM rab5 rab7 vps2 rnai front_0009-1-1-1-1.tif]

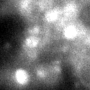

Supplement: Supplementary file 3 — Source data Fig. 1 [file 44318_2025_367_MOESM3_ESM.zip › SD figure 1 /1A/Fig_1_A_Roi/vps-2 (RNAi)/Gut close up/GFP ART C2 G rab5 rab7 vps2 rnai front_0009-1-1-1-1-1.tif]

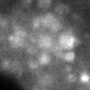

Supplement: Supplementary file 3 — Source data Fig. 1 [file 44318_2025_367_MOESM3_ESM.zip › SD figure 1 /1A/Fig_1_A_Roi/vps-2 (RNAi)/Gut close up/GFP ART C G rab5 rab7 vps2 rnai front_0009-1-1-1-1-1.tif]

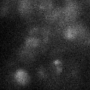

Supplement: Supplementary file 3 — Source data Fig. 1 [file 44318_2025_367_MOESM3_ESM.zip › SD figure 1 /1A/Fig_1_A_Roi/vps-2 (RNAi)/Gut close up/mCherry ART C2 MC rab5 rab7 vps2 rnai front_0009-1-1-1-1-1.tif]

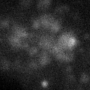

Supplement: Supplementary file 3 — Source data Fig. 1 [file 44318_2025_367_MOESM3_ESM.zip › SD figure 1 /1A/Fig_1_A_Roi/vps-2 (RNAi)/Gut close up/mCherry ART C MC rab5 rab7 vps2 rnai front_0009-1-1-1-1-1.tif]

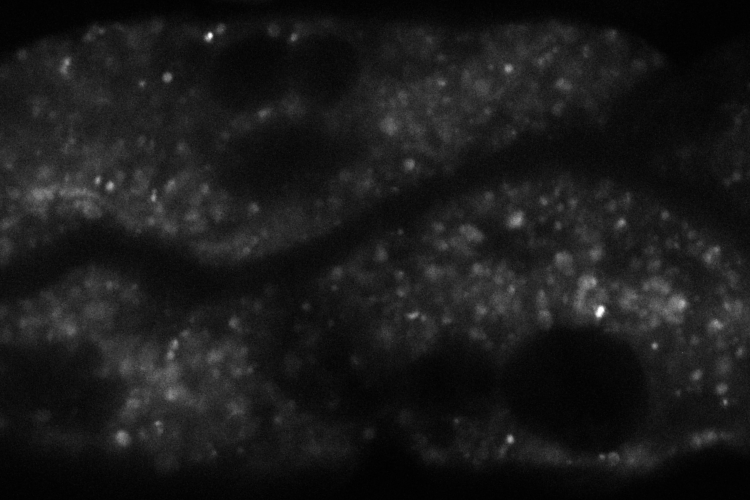

Supplement: Supplementary file 3 — Source data Fig. 1 [file 44318_2025_367_MOESM3_ESM.zip › SD figure 1 /1A/Fig_1_A_Roi/vps-2 (RNAi)/Gut/mCherry ART MC rab5 rab7 vps2 rnai front_0009-1-1-1-1.tif]
