# Supplementary figures and images for: Coordination between ESCRT function and Rab conversion during endosome maturation (part 3 of 9)
Source: EMBO J. 2025 Feb 5;44(6):1574–607. doi: 10.1038/s44318-025-00367-7 (PMC11914609; doi:10.1038/s44318-025-00367-7)

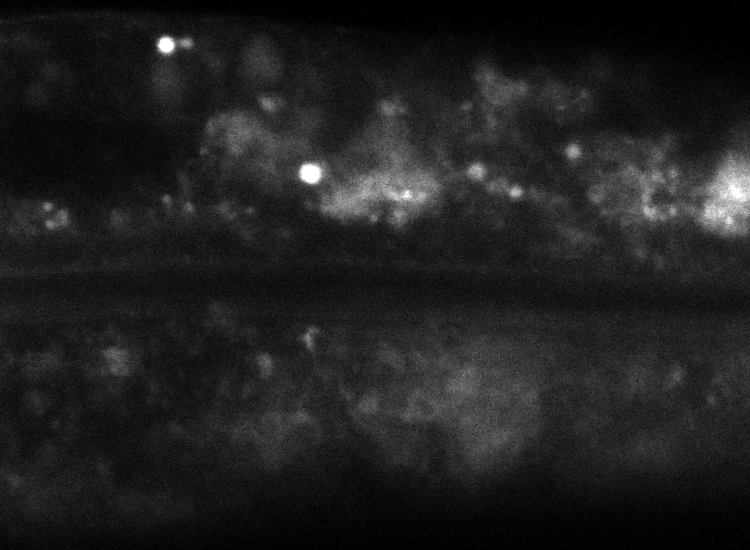

Supplement: Supplementary file 5 — Source data Fig. 3 [file 44318_2025_367_MOESM5_ESM.zip › SD figure 3/3A/Fig_3_A_Roi/vps-24 (RNAi)/Gut/Merge GFPvps27 RFPrab5 vps24 RNAi front_0010-ART.tif]

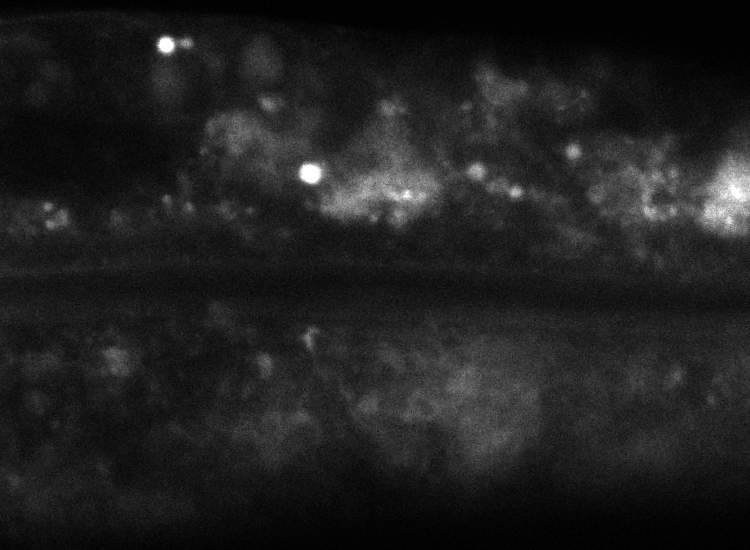

Supplement: Supplementary file 5 — Source data Fig. 3 [file 44318_2025_367_MOESM5_ESM.zip › SD figure 3/3A/Fig_3_A_Roi/vps-24 (RNAi)/Gut/RFP GFPvps27 RFPrab5 vps24 RNAi front_0010-ART-2.tif]

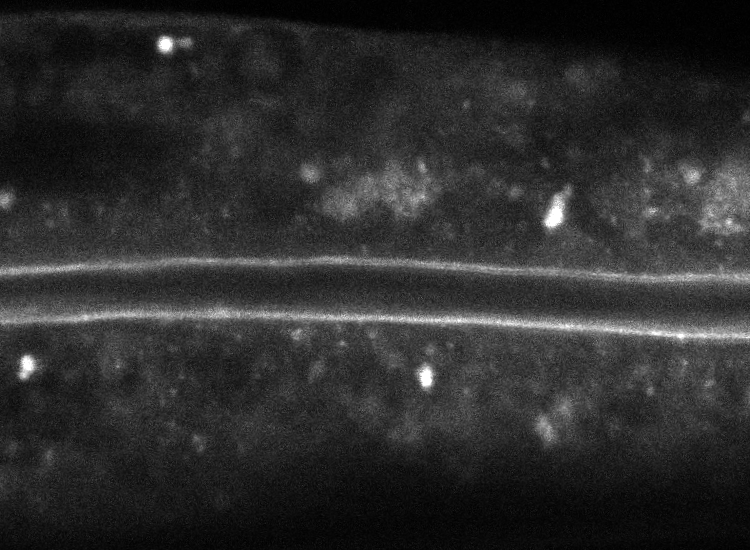

Supplement: Supplementary file 5 — Source data Fig. 3 [file 44318_2025_367_MOESM5_ESM.zip › SD figure 3/3A/Fig_3_A_Roi/vps-24 (RNAi)/Gut/GFP GFPvps27 RFPrab5 vps24 RNAi front_0010-ART-3.tif]

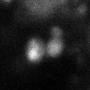

Supplement: Supplementary file 5 — Source data Fig. 3 [file 44318_2025_367_MOESM5_ESM.zip › SD figure 3/3A/Fig_3_A_Roi/vps-2 (RNAi)/Gut close up/RFP C2 R GFPvps27 RFPrab5 vps2 RNAi front_0017-ART-2-1.tif]

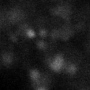

Supplement: Supplementary file 5 — Source data Fig. 3 [file 44318_2025_367_MOESM5_ESM.zip › SD figure 3/3A/Fig_3_A_Roi/vps-2 (RNAi)/Gut close up/RFP C R GFPvps27 RFPrab5 vps2 RNAi front_0017-ART-1-1.tif]

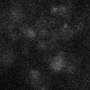

Supplement: Supplementary file 5 — Source data Fig. 3 [file 44318_2025_367_MOESM5_ESM.zip › SD figure 3/3A/Fig_3_A_Roi/vps-2 (RNAi)/Gut close up/GFP C G GFPvps27 RFPrab5 vps2 RNAi front_0017-ART-1-1.tif]

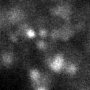

Supplement: Supplementary file 5 — Source data Fig. 3 [file 44318_2025_367_MOESM5_ESM.zip › SD figure 3/3A/Fig_3_A_Roi/vps-2 (RNAi)/Gut close up/Merge C MGRA GFPvps27 RFPrab5 vps2 RNAi front_0017-ART-1.tif]

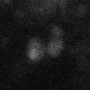

Supplement: Supplementary file 5 — Source data Fig. 3 [file 44318_2025_367_MOESM5_ESM.zip › SD figure 3/3A/Fig_3_A_Roi/vps-2 (RNAi)/Gut close up/GFP C2 G GFPvps27 RFPrab5 vps2 RNAi front_0017-ART-2-1.tif]

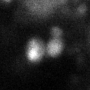

Supplement: Supplementary file 5 — Source data Fig. 3 [file 44318_2025_367_MOESM5_ESM.zip › SD figure 3/3A/Fig_3_A_Roi/vps-2 (RNAi)/Gut close up/Merge C2 MGRA GFPvps27 RFPrab5 vps2 RNAi front_0017-ART-2.tif]

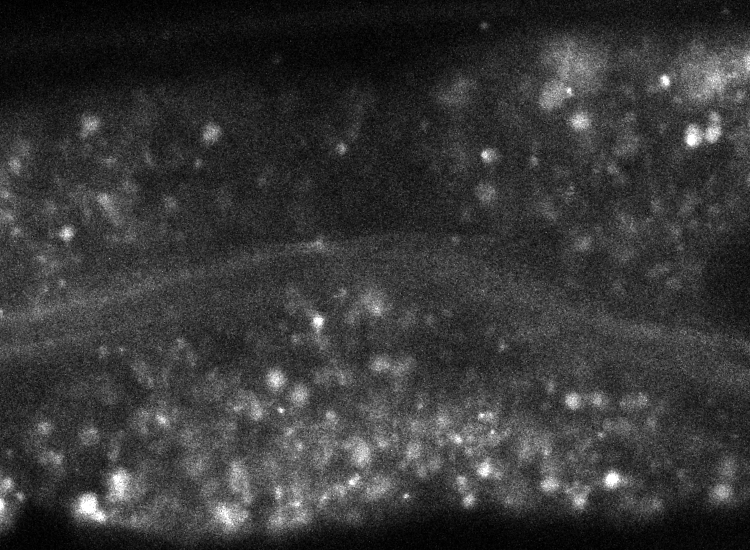

Supplement: Supplementary file 5 — Source data Fig. 3 [file 44318_2025_367_MOESM5_ESM.zip › SD figure 3/3A/Fig_3_A_Roi/vps-2 (RNAi)/Gut/GFP GFPvps27 RFPrab5 vps2 RNAi front_0017-ART-3.tif]

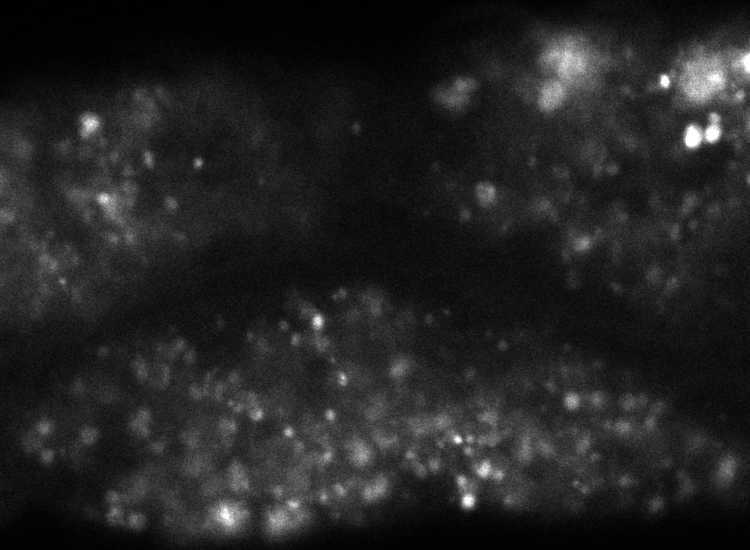

Supplement: Supplementary file 5 — Source data Fig. 3 [file 44318_2025_367_MOESM5_ESM.zip › SD figure 3/3A/Fig_3_A_Roi/vps-2 (RNAi)/Gut/Merge GFPvps27 RFPrab5 vps2 RNAi front_0017-ART.tif]

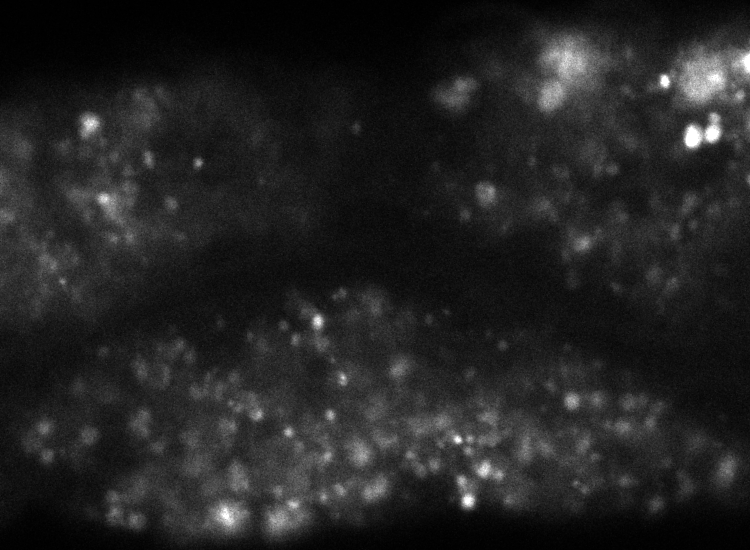

Supplement: Supplementary file 5 — Source data Fig. 3 [file 44318_2025_367_MOESM5_ESM.zip › SD figure 3/3A/Fig_3_A_Roi/vps-2 (RNAi)/Gut/RFP GFPvps27 RFPrab5 vps2 RNAi front_0017-ART-2.tif]

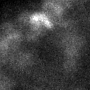

Supplement: Supplementary file 5 — Source data Fig. 3 [file 44318_2025_367_MOESM5_ESM.zip › SD figure 3/3A/Fig_3_A_Roi/vps-4 (RNAi) pre fed/Gut close up/Merge C2 MGRA GFPvps27 RFPrab5 vps4 RNAi L3 pre front_0007-ART-1.tif]

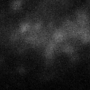

Supplement: Supplementary file 5 — Source data Fig. 3 [file 44318_2025_367_MOESM5_ESM.zip › SD figure 3/3A/Fig_3_A_Roi/vps-4 (RNAi) pre fed/Gut close up/RFP C R GFPvps27 RFPrab5 vps4 RNAi L3 pre front_0007-ART-1-1.tif]

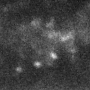

Supplement: Supplementary file 5 — Source data Fig. 3 [file 44318_2025_367_MOESM5_ESM.zip › SD figure 3/3A/Fig_3_A_Roi/vps-4 (RNAi) pre fed/Gut close up/GFP C G GFPvps27 RFPrab5 vps4 RNAi L3 pre front_0007-ART-1-1.tif]

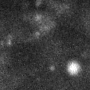

Supplement: Supplementary file 5 — Source data Fig. 3 [file 44318_2025_367_MOESM5_ESM.zip › SD figure 3/3A/Fig_3_A_Roi/vps-4 (RNAi) pre fed/Gut close up/GFP C2 G GFPvps27 RFPrab5 vps4 RNAi L3 pre front_0007-ART-1-1.tif]

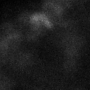

Supplement: Supplementary file 5 — Source data Fig. 3 [file 44318_2025_367_MOESM5_ESM.zip › SD figure 3/3A/Fig_3_A_Roi/vps-4 (RNAi) pre fed/Gut close up/RFP C2 R GFPvps27 RFPrab5 vps4 RNAi L3 pre front_0007-ART-1-1.tif]

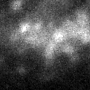

Supplement: Supplementary file 5 — Source data Fig. 3 [file 44318_2025_367_MOESM5_ESM.zip › SD figure 3/3A/Fig_3_A_Roi/vps-4 (RNAi) pre fed/Gut close up/Merge C MGRA GFPvps27 RFPrab5 vps4 RNAi L3 pre front_0007-ART-1.tif]

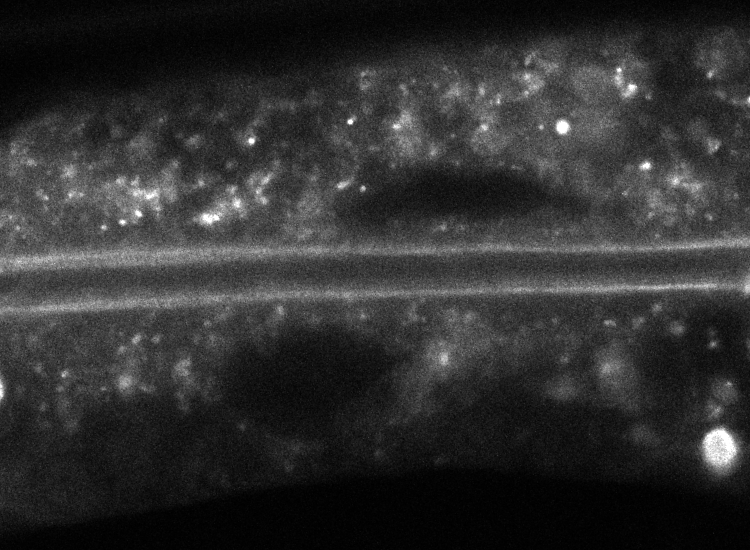

Supplement: Supplementary file 5 — Source data Fig. 3 [file 44318_2025_367_MOESM5_ESM.zip › SD figure 3/3A/Fig_3_A_Roi/vps-4 (RNAi) pre fed/Gut/GFP GFPvps27 RFPrab5 vps4 RNAi L3 pre front_0007-ART-3.tif]

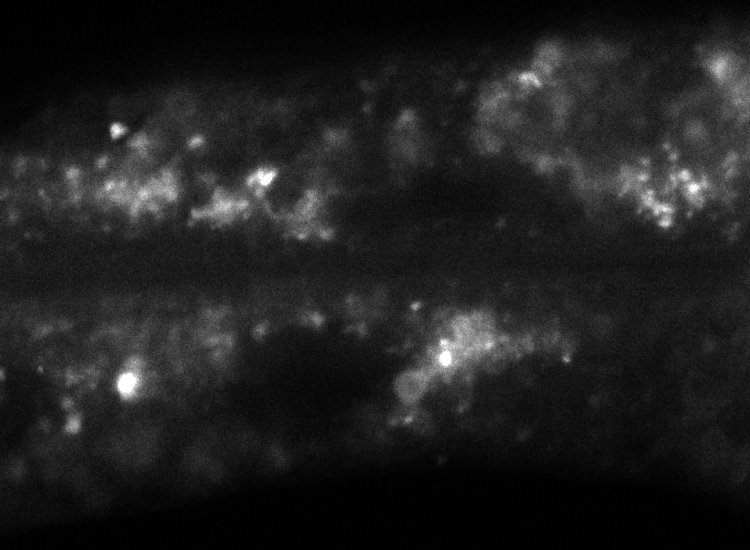

Supplement: Supplementary file 5 — Source data Fig. 3 [file 44318_2025_367_MOESM5_ESM.zip › SD figure 3/3A/Fig_3_A_Roi/vps-4 (RNAi) pre fed/Gut/Merge GFPvps27 RFPrab5 vps4 RNAi L3 pre front_0007-ART.tif]

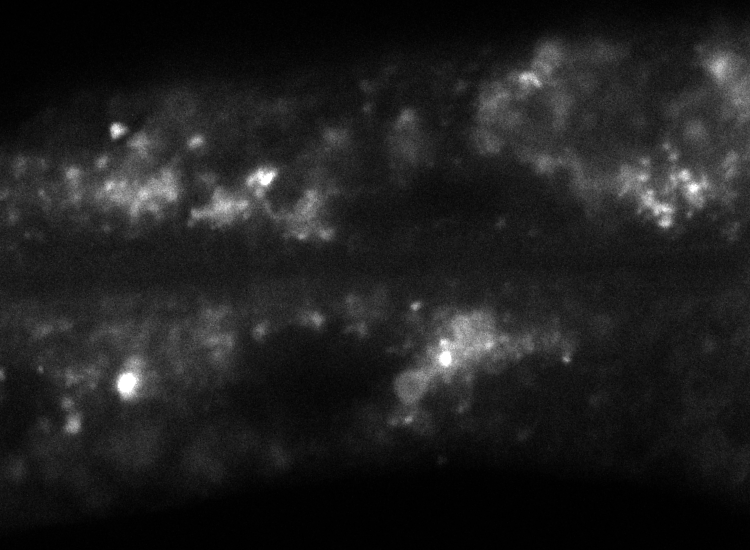

Supplement: Supplementary file 5 — Source data Fig. 3 [file 44318_2025_367_MOESM5_ESM.zip › SD figure 3/3A/Fig_3_A_Roi/vps-4 (RNAi) pre fed/Gut/RFP GFPvps27 RFPrab5 vps4 RNAi L3 pre front_0007-ART-2.tif]

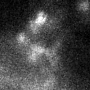

Supplement: Supplementary file 5 — Source data Fig. 3 [file 44318_2025_367_MOESM5_ESM.zip › SD figure 3/3A/Fig_3_A_Roi/tsg-101 (RNAi)/Gut close up/RFP C R GFPvps27 RFPrab5 tsg101 RNAi front_0010-ART-1-2-1.tif]

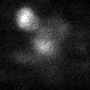

Supplement: Supplementary file 5 — Source data Fig. 3 [file 44318_2025_367_MOESM5_ESM.zip › SD figure 3/3A/Fig_3_A_Roi/tsg-101 (RNAi)/Gut close up/Merge C MGRA GFPvps27 RFPrab5 tsg101 RNAi front_0010-ART-1.tif]

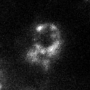

Supplement: Supplementary file 5 — Source data Fig. 3 [file 44318_2025_367_MOESM5_ESM.zip › SD figure 3/3A/Fig_3_A_Roi/tsg-101 (RNAi)/Gut close up/GFP C G GFPvps27 RFPrab5 tsg101 RNAi front_0010-ART-1-2-1.tif]

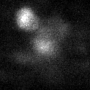

Supplement: Supplementary file 5 — Source data Fig. 3 [file 44318_2025_367_MOESM5_ESM.zip › SD figure 3/3A/Fig_3_A_Roi/tsg-101 (RNAi)/Gut close up/RFP C R GFPvps27 RFPrab5 tsg101 RNAi front_0010-ART-1-1.tif]

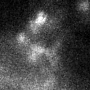

Supplement: Supplementary file 5 — Source data Fig. 3 [file 44318_2025_367_MOESM5_ESM.zip › SD figure 3/3A/Fig_3_A_Roi/tsg-101 (RNAi)/Gut close up/Merge C MGRA GFPvps27 RFPrab5 tsg101 RNAi front_0010-ART-1-2.tif]

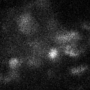

Supplement: Supplementary file 5 — Source data Fig. 3 [file 44318_2025_367_MOESM5_ESM.zip › SD figure 3/3A/Fig_3_A_Roi/tsg-101 (RNAi)/Gut close up/GFP C G GFPvps27 RFPrab5 tsg101 RNAi front_0010-ART-1-1.tif]

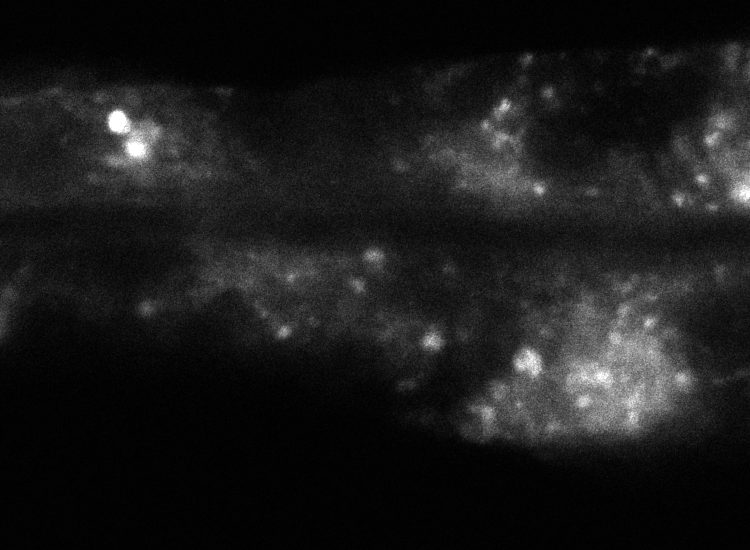

Supplement: Supplementary file 5 — Source data Fig. 3 [file 44318_2025_367_MOESM5_ESM.zip › SD figure 3/3A/Fig_3_A_Roi/tsg-101 (RNAi)/Gut/RFP GFPvps27 RFPrab5 tsg101 RNAi front_0010-ART-2.tif]

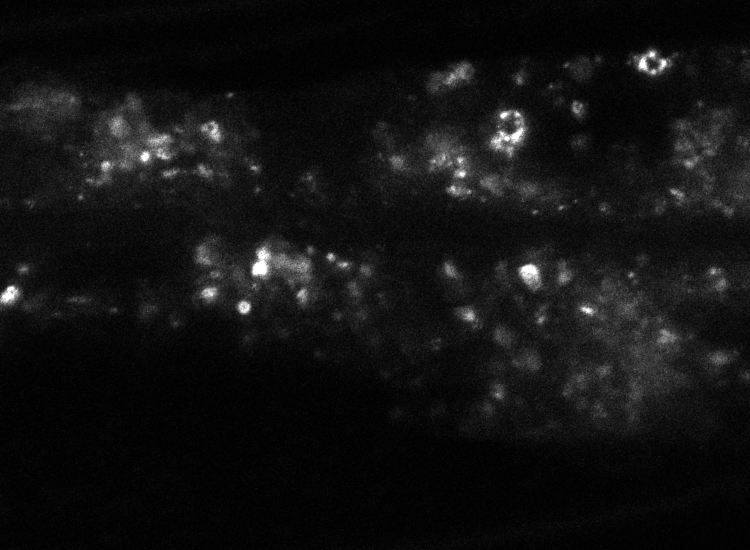

Supplement: Supplementary file 5 — Source data Fig. 3 [file 44318_2025_367_MOESM5_ESM.zip › SD figure 3/3A/Fig_3_A_Roi/tsg-101 (RNAi)/Gut/GFP GFPvps27 RFPrab5 tsg101 RNAi front_0010-ART-3.tif]

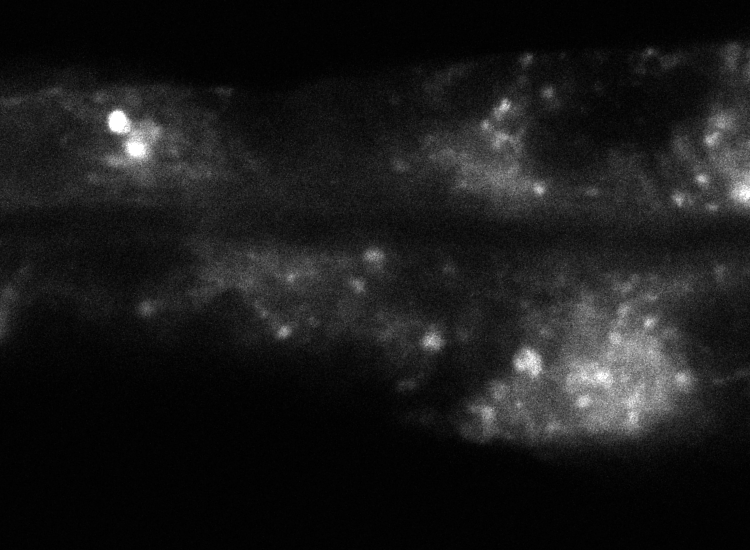

Supplement: Supplementary file 5 — Source data Fig. 3 [file 44318_2025_367_MOESM5_ESM.zip › SD figure 3/3A/Fig_3_A_Roi/tsg-101 (RNAi)/Gut/Merge GFPvps27 RFPrab5 tsg101 RNAi front_0010-ART.tif]

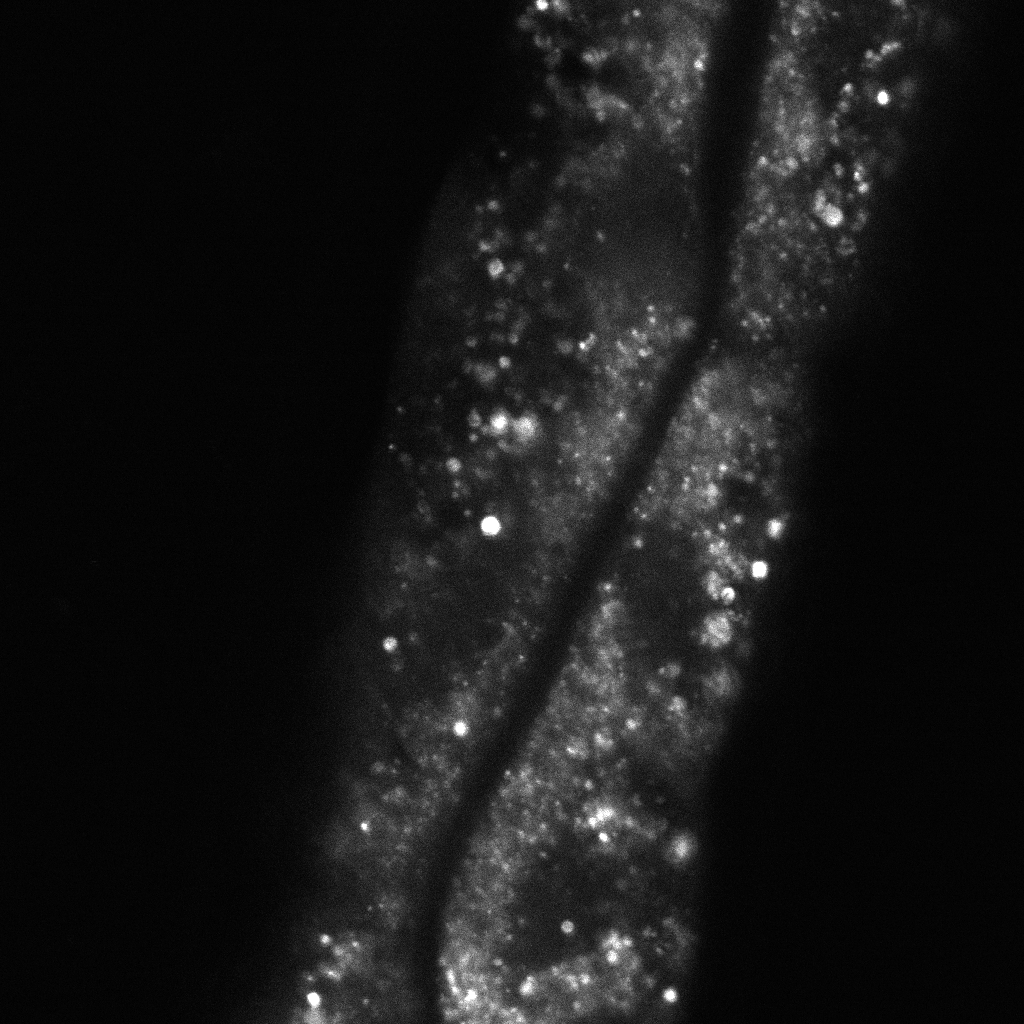

Supplement: Supplementary file 6 — Source data Fig. 4 [file 44318_2025_367_MOESM6_ESM.zip › SD figure 4/4A/Fig_4_A_data/vps-20 (RNAi)/A ERT261 GFPubq vps20 RNAi front_0017-1.tif]

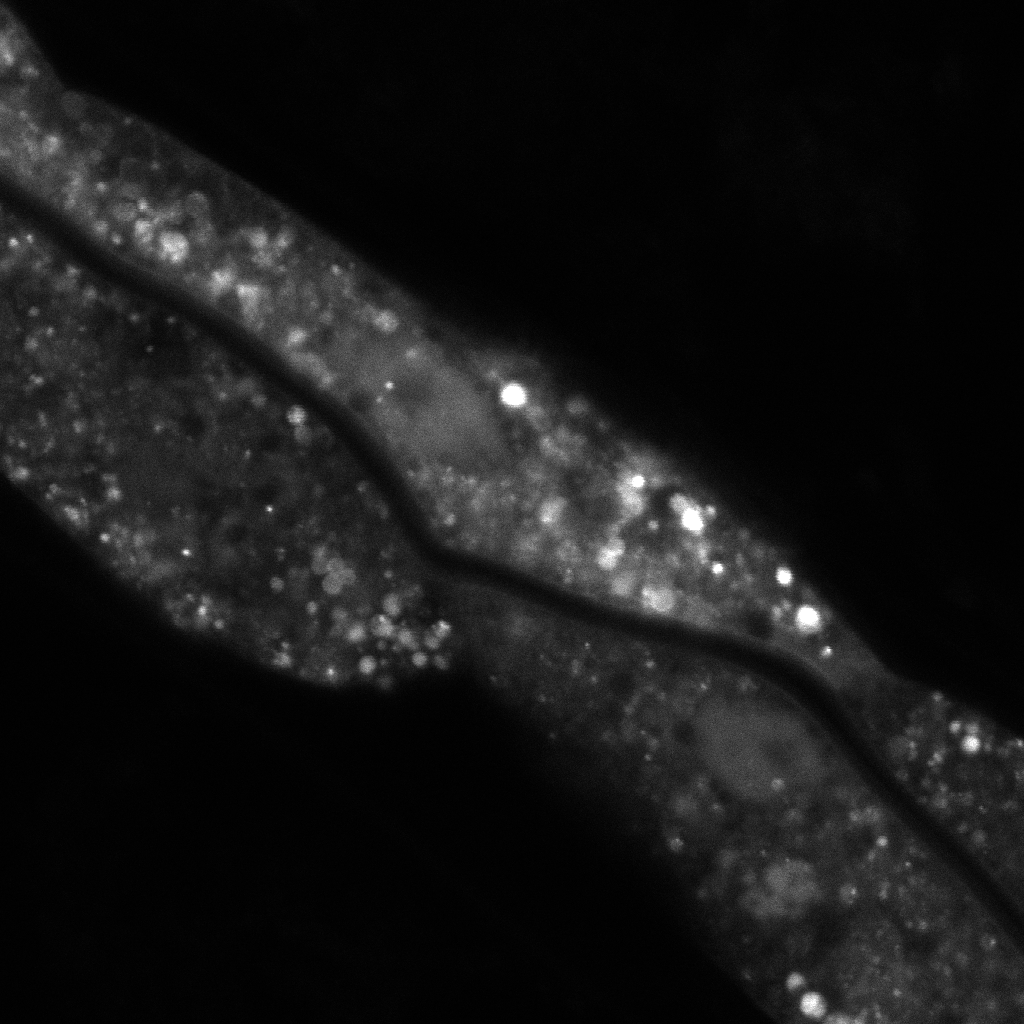

Supplement: Supplementary file 6 — Source data Fig. 4 [file 44318_2025_367_MOESM6_ESM.zip › SD figure 4/4A/Fig_4_A_data/vps-4 (RNAi) /A ERT261 GFPubq vps4 RNAi front_0011-1.tif]

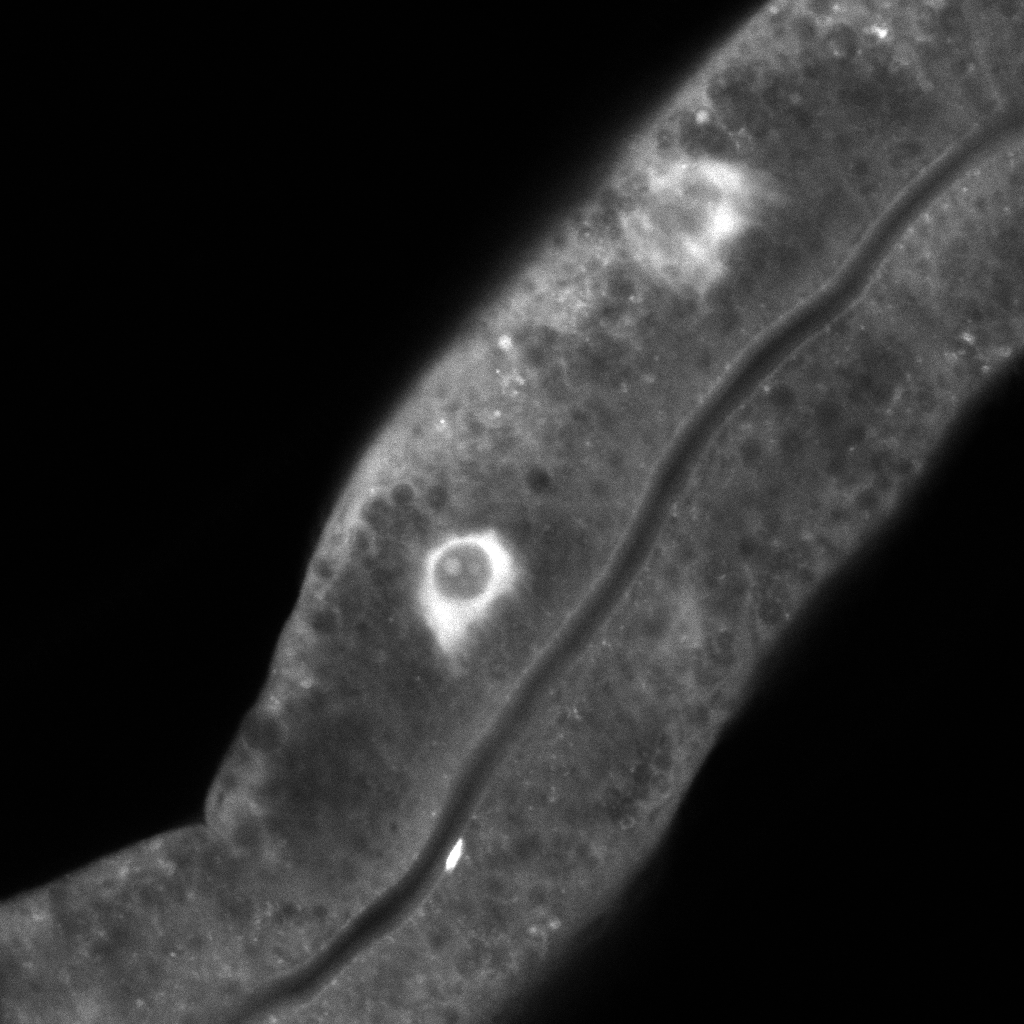

Supplement: Supplementary file 6 — Source data Fig. 4 [file 44318_2025_367_MOESM6_ESM.zip › SD figure 4/4A/Fig_4_A_data/Mock/A ERT261 GFPubq control RNAi front_0002-1.tif]

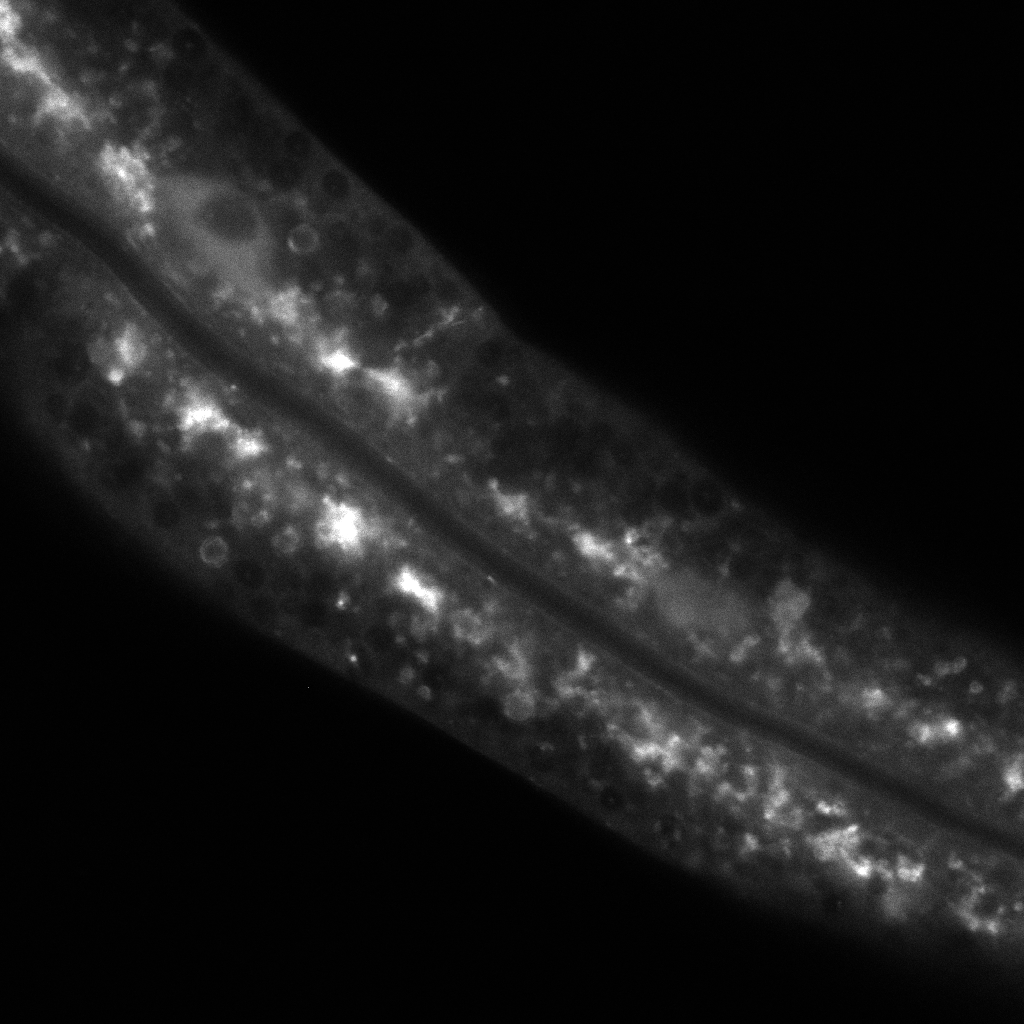

Supplement: Supplementary file 6 — Source data Fig. 4 [file 44318_2025_367_MOESM6_ESM.zip › SD figure 4/4A/Fig_4_A_data/vps-24 (RNAi)/A ERT261 GFPubq vps24 RNAi front_0018-1.tif]

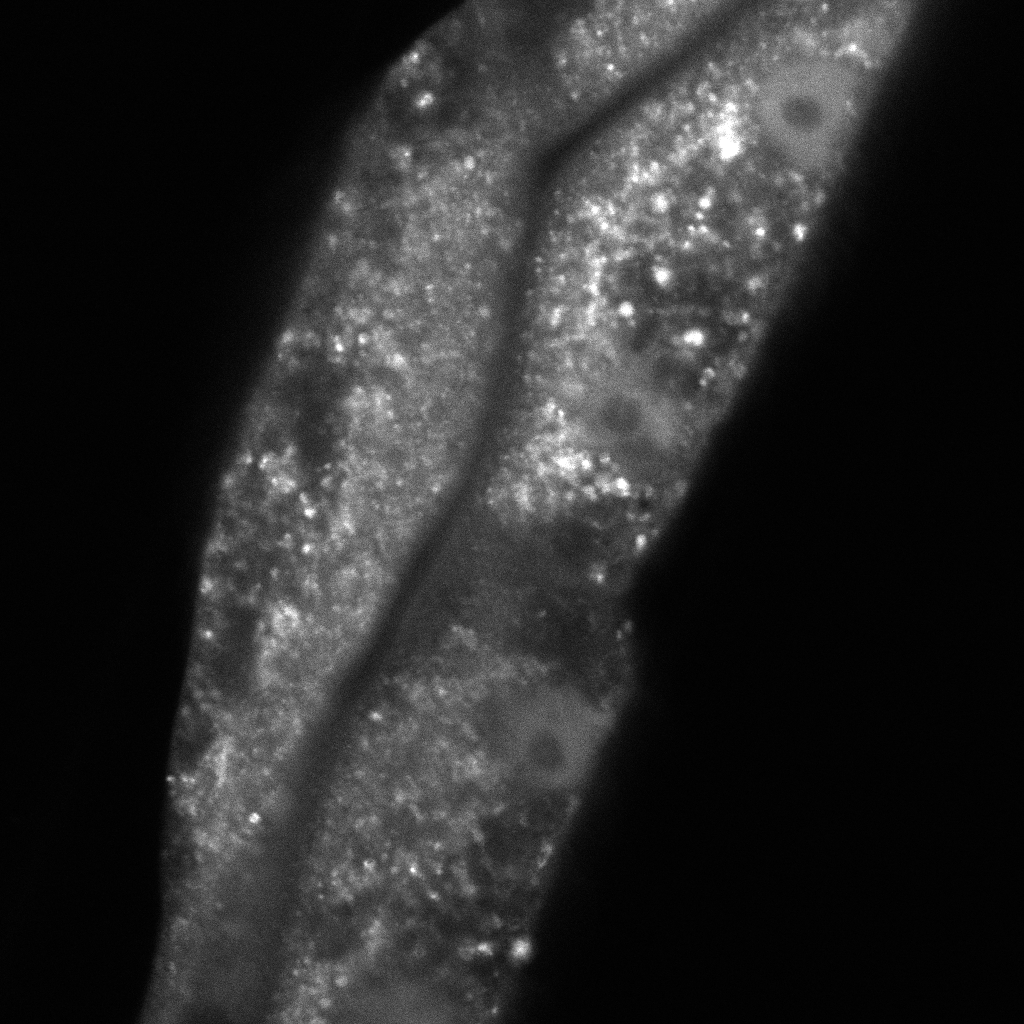

Supplement: Supplementary file 6 — Source data Fig. 4 [file 44318_2025_367_MOESM6_ESM.zip › SD figure 4/4A/Fig_4_A_data/vps-2 (RNAi)/A ERT261 GFPubq vps2 RNAi front_0005-1.tif]

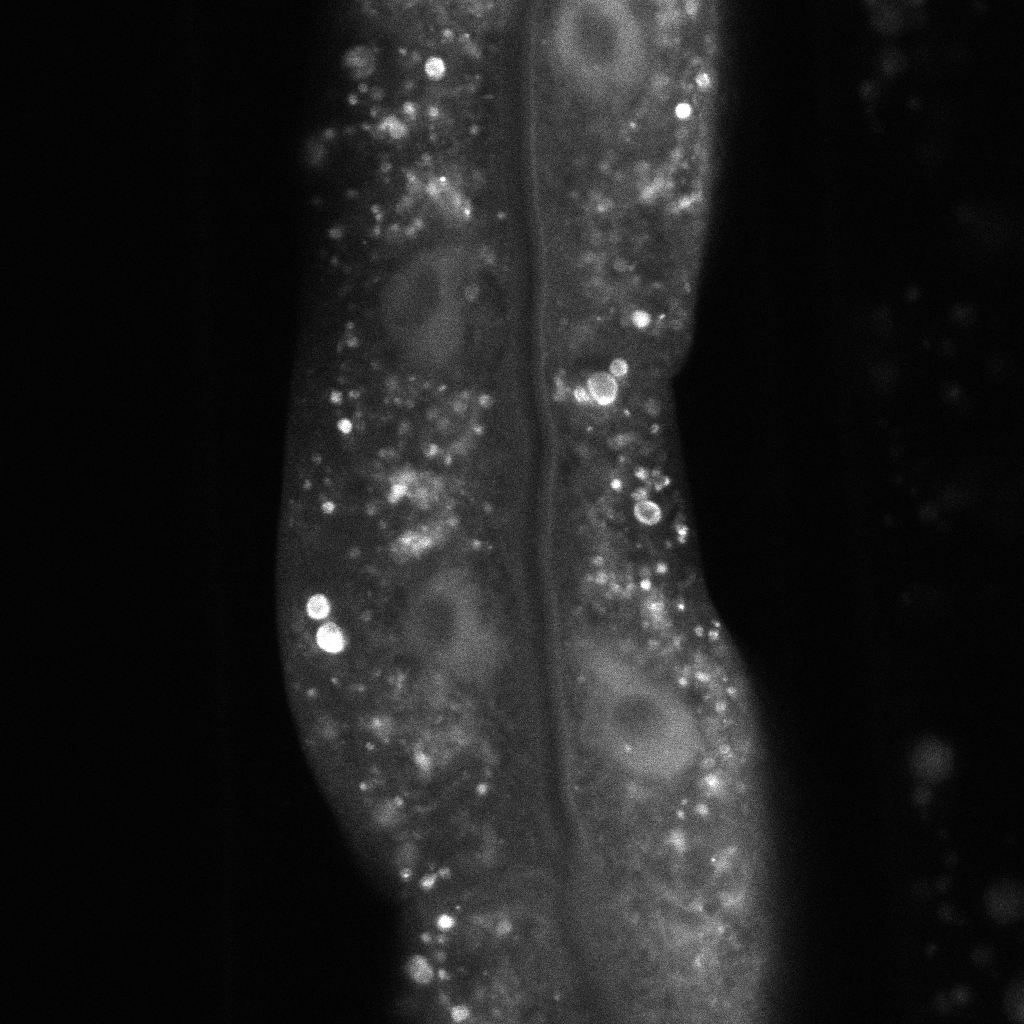

Supplement: Supplementary file 6 — Source data Fig. 4 [file 44318_2025_367_MOESM6_ESM.zip › SD figure 4/4A/Fig_4_A_data/hgrs-1 (RNAi)/A ERT261 GFPubq vps27 RNAi front_0007-1.tif]

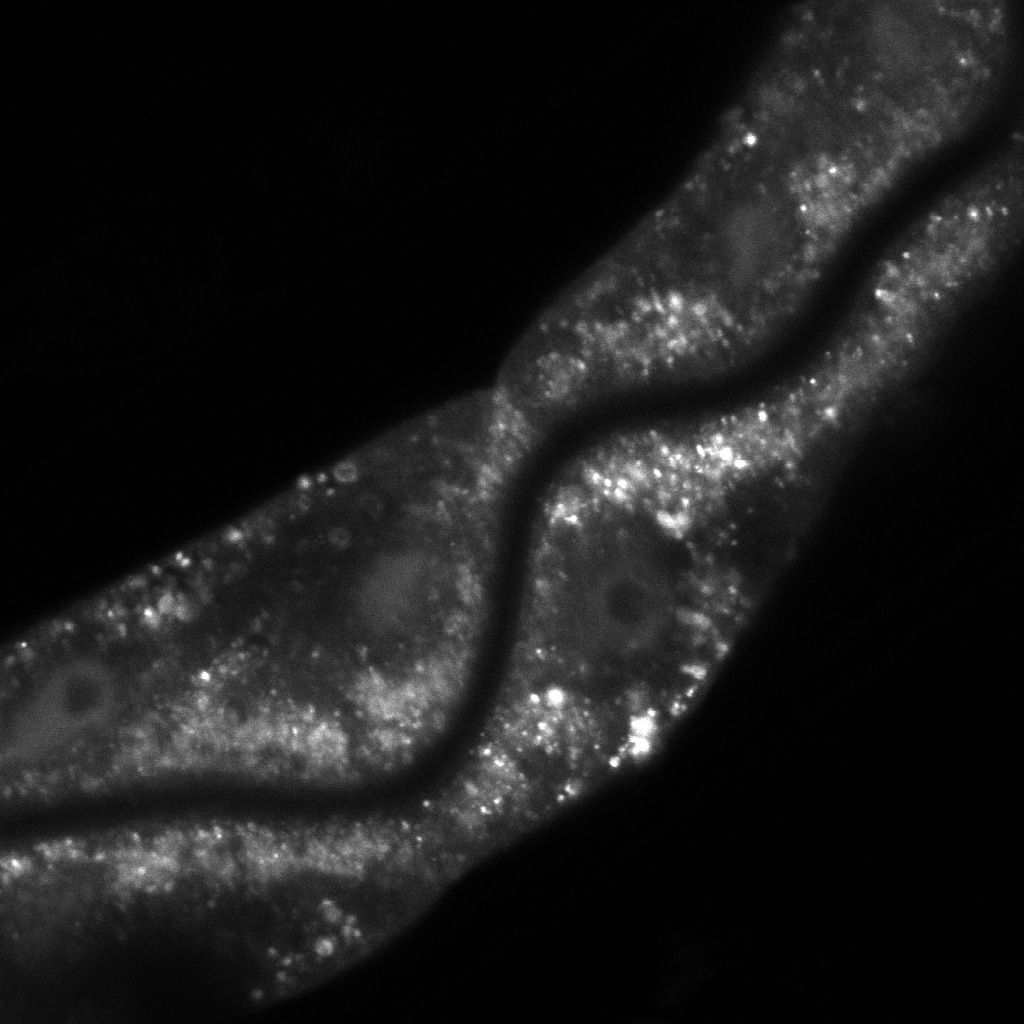

Supplement: Supplementary file 6 — Source data Fig. 4 [file 44318_2025_367_MOESM6_ESM.zip › SD figure 4/4A/Fig_4_A_data/tsg-101 (RNAi)/A ERT261 GFPubq tsg101 RNAi front_0022-1.tif]

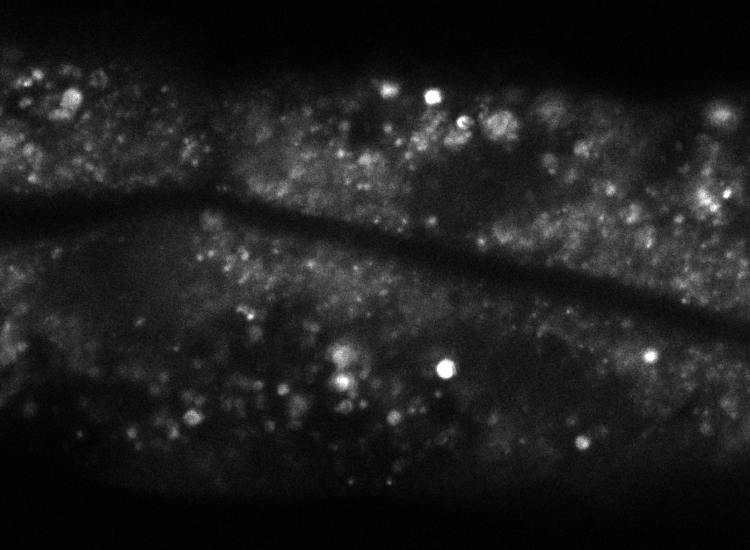

Supplement: Supplementary file 6 — Source data Fig. 4 [file 44318_2025_367_MOESM6_ESM.zip › SD figure 4/4A/Fig_4_A_Roi/vps-20 (RNAi)/Gut/ART GFP ERT261 GFPubq vps20 RNAi front_0017-1-1-1-1.tif]

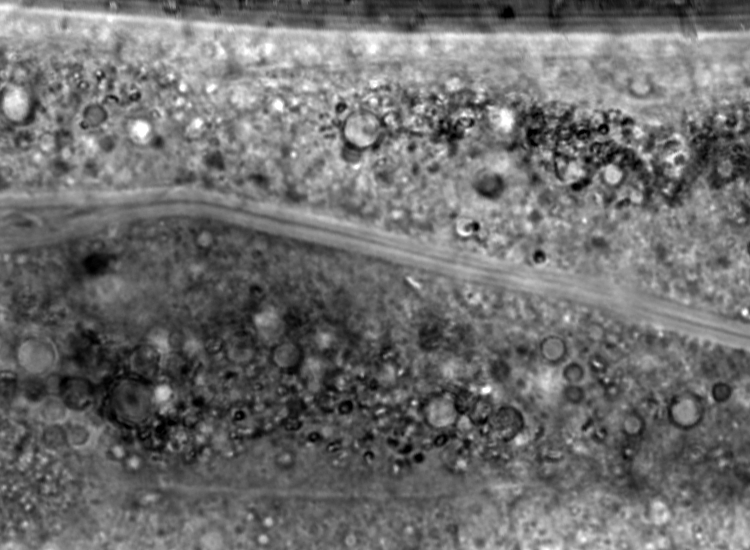

Supplement: Supplementary file 6 — Source data Fig. 4 [file 44318_2025_367_MOESM6_ESM.zip › SD figure 4/4A/Fig_4_A_Roi/vps-20 (RNAi)/Gut/ART DIC ERT261 GFPubq vps20 RNAi front_0017-1-1-1-1.tif]

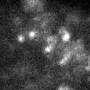

Supplement: Supplementary file 6 — Source data Fig. 4 [file 44318_2025_367_MOESM6_ESM.zip › SD figure 4/4A/Fig_4_A_Roi/vps-20 (RNAi)/Gut close up/ART GFP CU2 ERT261 GFPubq vps20 RNAi front_0017-1-1-1-1-1.tif]

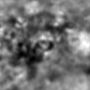

Supplement: Supplementary file 6 — Source data Fig. 4 [file 44318_2025_367_MOESM6_ESM.zip › SD figure 4/4A/Fig_4_A_Roi/vps-20 (RNAi)/Gut close up/ART DIC CU ERT261 GFPubq vps20 RNAi front_0017-1-1-1-1-1.tif]

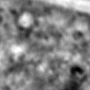

Supplement: Supplementary file 6 — Source data Fig. 4 [file 44318_2025_367_MOESM6_ESM.zip › SD figure 4/4A/Fig_4_A_Roi/vps-20 (RNAi)/Gut close up/ART DIC CU2 ERT261 GFPubq vps20 RNAi front_0017-1-1-1-1-1.tif]

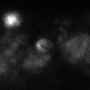

Supplement: Supplementary file 6 — Source data Fig. 4 [file 44318_2025_367_MOESM6_ESM.zip › SD figure 4/4A/Fig_4_A_Roi/vps-20 (RNAi)/Gut close up/ART GFP CU ERT261 GFPubq vps20 RNAi front_0017-1-1-1-1-1.tif]

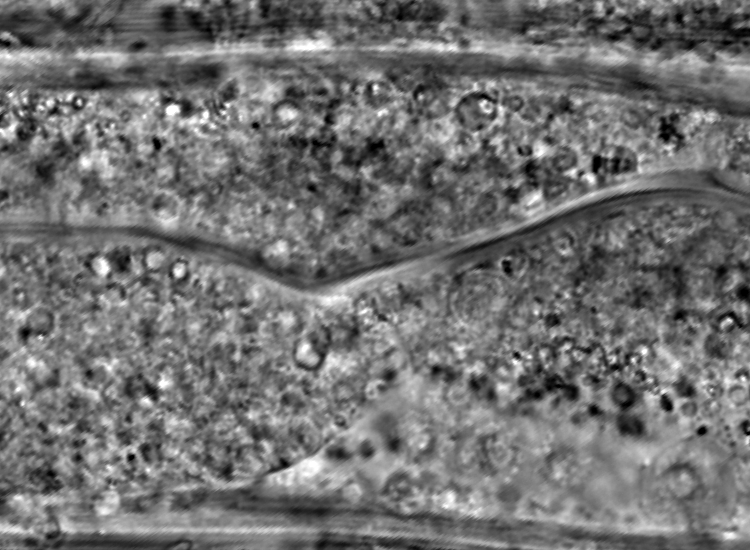

Supplement: Supplementary file 6 — Source data Fig. 4 [file 44318_2025_367_MOESM6_ESM.zip › SD figure 4/4A/Fig_4_A_Roi/vps-4 (RNAi) /Gut/ART DIC ERT261 GFPubq vps4 RNAi front_0011-1-1-1-1.tif]

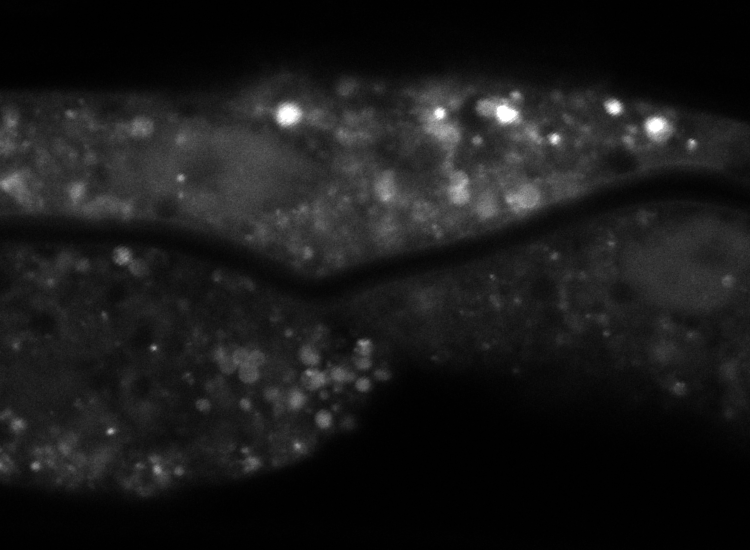

Supplement: Supplementary file 6 — Source data Fig. 4 [file 44318_2025_367_MOESM6_ESM.zip › SD figure 4/4A/Fig_4_A_Roi/vps-4 (RNAi) /Gut/ART GFP ERT261 GFPubq vps4 RNAi front_0011-1-1-1-1.tif]

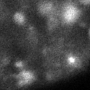

Supplement: Supplementary file 6 — Source data Fig. 4 [file 44318_2025_367_MOESM6_ESM.zip › SD figure 4/4A/Fig_4_A_Roi/vps-4 (RNAi) /Gut close up/ART GFP CU ERT261 GFPubq vps4 RNAi front_0011-1-1-1-1-1.tif]

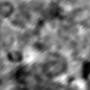

Supplement: Supplementary file 6 — Source data Fig. 4 [file 44318_2025_367_MOESM6_ESM.zip › SD figure 4/4A/Fig_4_A_Roi/vps-4 (RNAi) /Gut close up/ART DIC CU2 ERT261 GFPubq vps4 RNAi front_0011-1-1-1-1-1.tif]

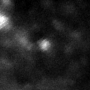

Supplement: Supplementary file 6 — Source data Fig. 4 [file 44318_2025_367_MOESM6_ESM.zip › SD figure 4/4A/Fig_4_A_Roi/vps-4 (RNAi) /Gut close up/ART GFP CU2 ERT261 GFPubq vps4 RNAi front_0011-1-1-1-1-1.tif]

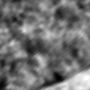

Supplement: Supplementary file 6 — Source data Fig. 4 [file 44318_2025_367_MOESM6_ESM.zip › SD figure 4/4A/Fig_4_A_Roi/vps-4 (RNAi) /Gut close up/ART DIC CU ERT261 GFPubq vps4 RNAi front_0011-1-1-1-1-1.tif]

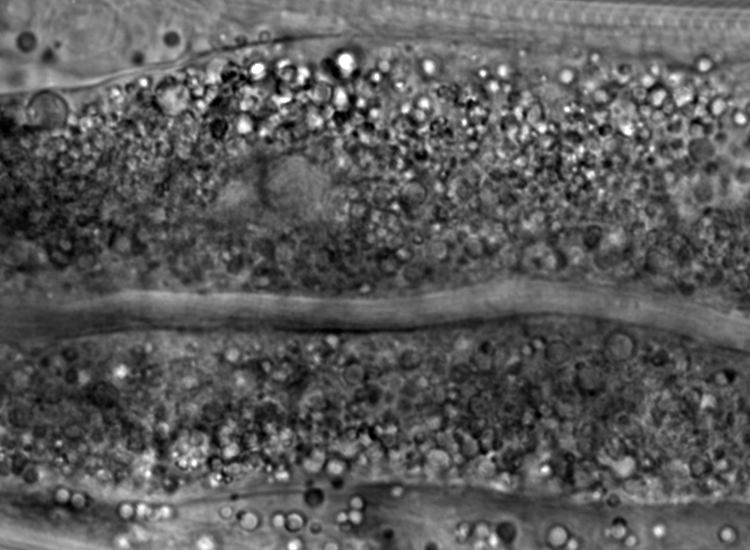

Supplement: Supplementary file 6 — Source data Fig. 4 [file 44318_2025_367_MOESM6_ESM.zip › SD figure 4/4A/Fig_4_A_Roi/Mock/Gut/ART DIC ERT261 GFPubq control RNAi front_0002-1-1-1-1.tif]

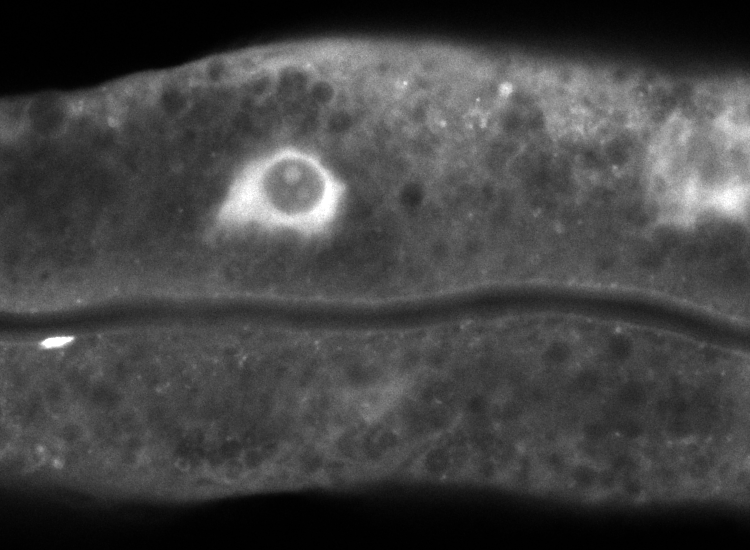

Supplement: Supplementary file 6 — Source data Fig. 4 [file 44318_2025_367_MOESM6_ESM.zip › SD figure 4/4A/Fig_4_A_Roi/Mock/Gut/ART GFP ERT261 GFPubq control RNAi front_0002-1-1-1-1.tif]

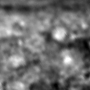

Supplement: Supplementary file 6 — Source data Fig. 4 [file 44318_2025_367_MOESM6_ESM.zip › SD figure 4/4A/Fig_4_A_Roi/Mock/Gut close up/ART DIC CU ERT261 GFPubq control RNAi front_0002-1-1-1-1-1.tif]

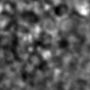

Supplement: Supplementary file 6 — Source data Fig. 4 [file 44318_2025_367_MOESM6_ESM.zip › SD figure 4/4A/Fig_4_A_Roi/Mock/Gut close up/ART DIC CU2 ERT261 GFPubq control RNAi front_0002-1-1-1-1-1.tif]

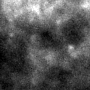

Supplement: Supplementary file 6 — Source data Fig. 4 [file 44318_2025_367_MOESM6_ESM.zip › SD figure 4/4A/Fig_4_A_Roi/Mock/Gut close up/ART GFP CU2 ERT261 GFPubq control RNAi front_0002-1-1-1-1-1.tif]

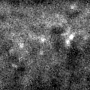

Supplement: Supplementary file 6 — Source data Fig. 4 [file 44318_2025_367_MOESM6_ESM.zip › SD figure 4/4A/Fig_4_A_Roi/Mock/Gut close up/ART GFP CU ERT261 GFPubq control RNAi front_0002-1-1-1-1-1.tif]

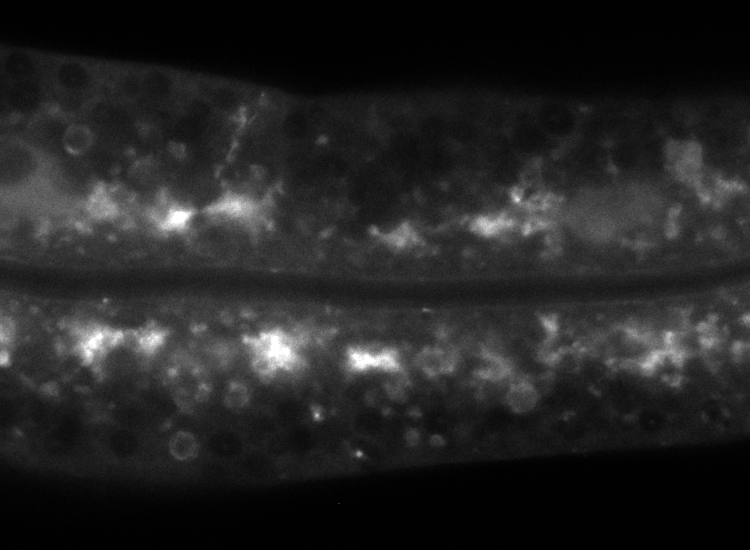

Supplement: Supplementary file 6 — Source data Fig. 4 [file 44318_2025_367_MOESM6_ESM.zip › SD figure 4/4A/Fig_4_A_Roi/vps-24 (RNAi)/Gut/ART GFP ERT261 GFPubq vps24 RNAi front_0018-1-1-1-1.tif]

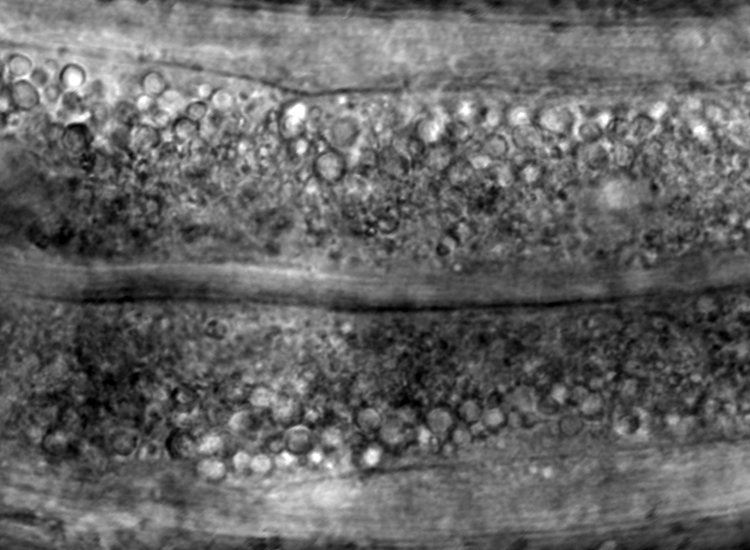

Supplement: Supplementary file 6 — Source data Fig. 4 [file 44318_2025_367_MOESM6_ESM.zip › SD figure 4/4A/Fig_4_A_Roi/vps-24 (RNAi)/Gut/ART DIC ERT261 GFPubq vps24 RNAi front_0018-1-1-1-1.tif]

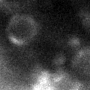

Supplement: Supplementary file 6 — Source data Fig. 4 [file 44318_2025_367_MOESM6_ESM.zip › SD figure 4/4A/Fig_4_A_Roi/vps-24 (RNAi)/Gut close up/ART GFP CU2 ERT261 GFPubq vps24 RNAi front_0018-1-1-1-1-1.tif]

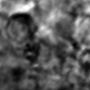

Supplement: Supplementary file 6 — Source data Fig. 4 [file 44318_2025_367_MOESM6_ESM.zip › SD figure 4/4A/Fig_4_A_Roi/vps-24 (RNAi)/Gut close up/ART DIC CU2 ERT261 GFPubq vps24 RNAi front_0018-1-1-1-1-1.tif]

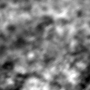

Supplement: Supplementary file 6 — Source data Fig. 4 [file 44318_2025_367_MOESM6_ESM.zip › SD figure 4/4A/Fig_4_A_Roi/vps-24 (RNAi)/Gut close up/ART DIC CU ERT261 GFPubq vps24 RNAi front_0018-1-1-1-1-1.tif]

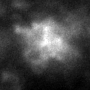

Supplement: Supplementary file 6 — Source data Fig. 4 [file 44318_2025_367_MOESM6_ESM.zip › SD figure 4/4A/Fig_4_A_Roi/vps-24 (RNAi)/Gut close up/ART GFP CU ERT261 GFPubq vps24 RNAi front_0018-1-1-1-1-1.tif]

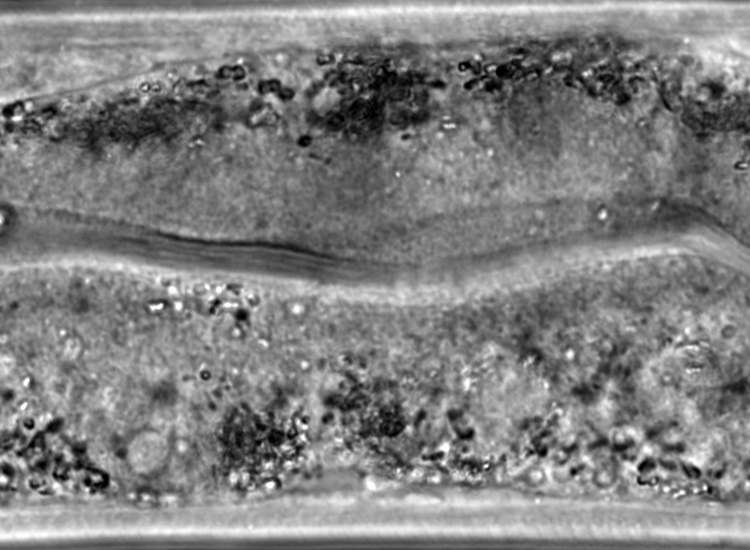

Supplement: Supplementary file 6 — Source data Fig. 4 [file 44318_2025_367_MOESM6_ESM.zip › SD figure 4/4A/Fig_4_A_Roi/vps-2 (RNAi)/Gut/ART DIC ERT261 GFPubq vps2 RNAi front_0005-1-1-1-1.tif]

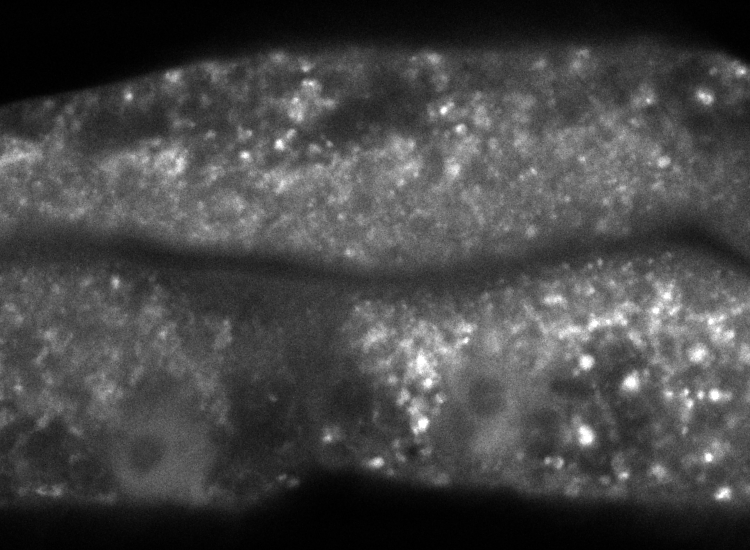

Supplement: Supplementary file 6 — Source data Fig. 4 [file 44318_2025_367_MOESM6_ESM.zip › SD figure 4/4A/Fig_4_A_Roi/vps-2 (RNAi)/Gut/ART GFP ERT261 GFPubq vps2 RNAi front_0005-1-1-1-1.tif]

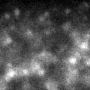

Supplement: Supplementary file 6 — Source data Fig. 4 [file 44318_2025_367_MOESM6_ESM.zip › SD figure 4/4A/Fig_4_A_Roi/vps-2 (RNAi)/Gut close up/ART GFP CU ERT261 GFPubq vps2 RNAi front_0005-1-1-1-1-1.tif]

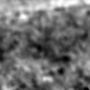

Supplement: Supplementary file 6 — Source data Fig. 4 [file 44318_2025_367_MOESM6_ESM.zip › SD figure 4/4A/Fig_4_A_Roi/vps-2 (RNAi)/Gut close up/ART DIC CU ERT261 GFPubq vps2 RNAi front_0005-1-1-1-1-1.tif]

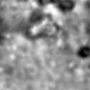

Supplement: Supplementary file 6 — Source data Fig. 4 [file 44318_2025_367_MOESM6_ESM.zip › SD figure 4/4A/Fig_4_A_Roi/vps-2 (RNAi)/Gut close up/ART DIC CU2 ERT261 GFPubq vps2 RNAi front_0005-1-1-1-1-1.tif]

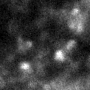

Supplement: Supplementary file 6 — Source data Fig. 4 [file 44318_2025_367_MOESM6_ESM.zip › SD figure 4/4A/Fig_4_A_Roi/vps-2 (RNAi)/Gut close up/ART GFP CU2 ERT261 GFPubq vps2 RNAi front_0005-1-1-1-1-1.tif]

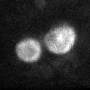

Supplement: Supplementary file 6 — Source data Fig. 4 [file 44318_2025_367_MOESM6_ESM.zip › SD figure 4/4A/Fig_4_A_Roi/hgrs-1 (RNAi)/hgrs-1 (RNAi) Gut close up/ART GFP CU2 ERT261 GFPubq vps27 RNAi front_0007-1-1-1-1-1.tif]

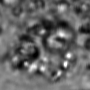

Supplement: Supplementary file 6 — Source data Fig. 4 [file 44318_2025_367_MOESM6_ESM.zip › SD figure 4/4A/Fig_4_A_Roi/hgrs-1 (RNAi)/hgrs-1 (RNAi) Gut close up/ART DIC CU2 ERT261 GFPubq vps27 RNAi front_0007-1-1-1-1-1.tif]

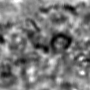

Supplement: Supplementary file 6 — Source data Fig. 4 [file 44318_2025_367_MOESM6_ESM.zip › SD figure 4/4A/Fig_4_A_Roi/hgrs-1 (RNAi)/hgrs-1 (RNAi) Gut close up/ART DIC CU ERT261 GFPubq vps27 RNAi front_0007-1-1-1-1-1.tif]

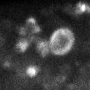

Supplement: Supplementary file 6 — Source data Fig. 4 [file 44318_2025_367_MOESM6_ESM.zip › SD figure 4/4A/Fig_4_A_Roi/hgrs-1 (RNAi)/hgrs-1 (RNAi) Gut close up/ART GFP CU ERT261 GFPubq vps27 RNAi front_0007-1-1-1-1-1.tif]

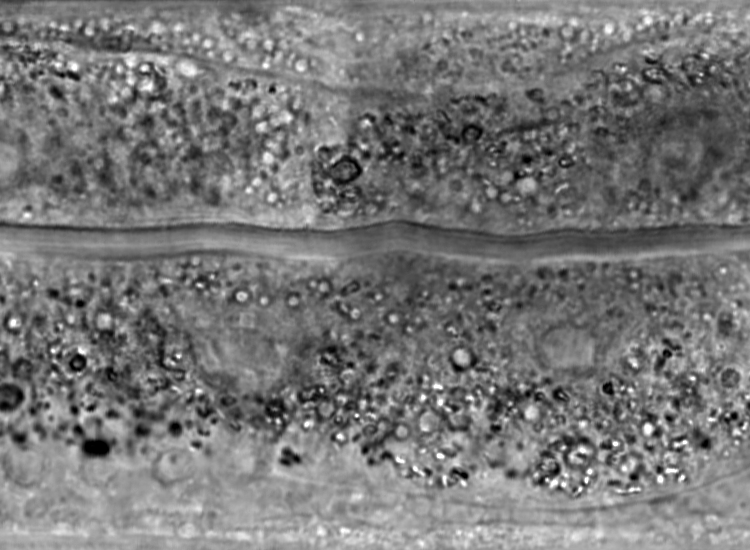

Supplement: Supplementary file 6 — Source data Fig. 4 [file 44318_2025_367_MOESM6_ESM.zip › SD figure 4/4A/Fig_4_A_Roi/hgrs-1 (RNAi)/Gut/ART DIC ERT261 GFPubq vps27 RNAi front_0007-1-1-1-1.tif]

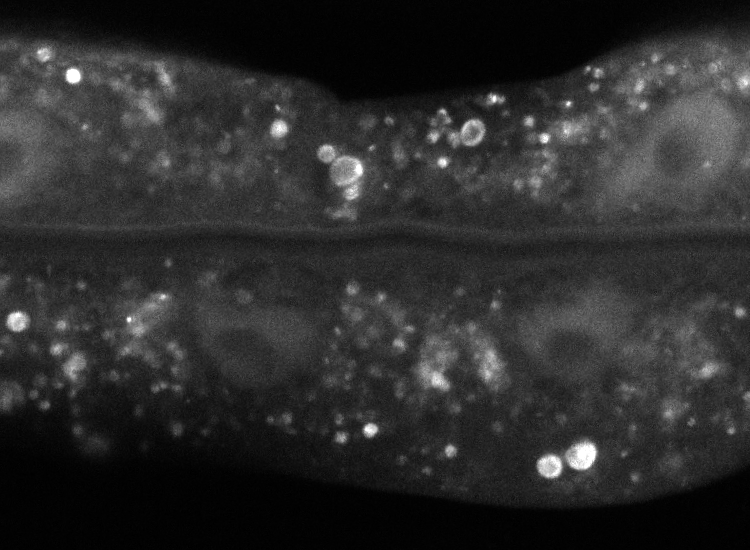

Supplement: Supplementary file 6 — Source data Fig. 4 [file 44318_2025_367_MOESM6_ESM.zip › SD figure 4/4A/Fig_4_A_Roi/hgrs-1 (RNAi)/Gut/ART GFP ERT261 GFPubq vps27 RNAi front_0007-1-1-1-1.tif]

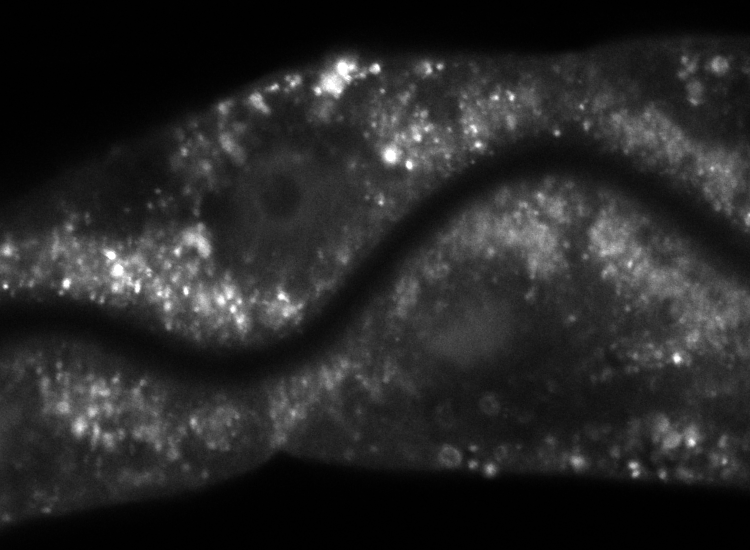

Supplement: Supplementary file 6 — Source data Fig. 4 [file 44318_2025_367_MOESM6_ESM.zip › SD figure 4/4A/Fig_4_A_Roi/tsg-101 (RNAi)/Gut/ART GFP ERT261 GFPubq tsg101 RNAi front_0022-1-1-1-1.tif]

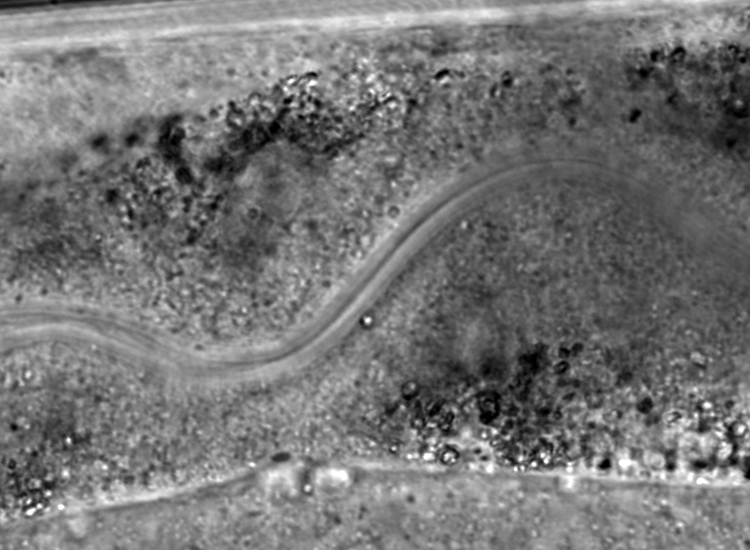

Supplement: Supplementary file 6 — Source data Fig. 4 [file 44318_2025_367_MOESM6_ESM.zip › SD figure 4/4A/Fig_4_A_Roi/tsg-101 (RNAi)/Gut/ART DIC ERT261 GFPubq tsg101 RNAi front_0022-1-1-1-1.tif]

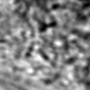

Supplement: Supplementary file 6 — Source data Fig. 4 [file 44318_2025_367_MOESM6_ESM.zip › SD figure 4/4A/Fig_4_A_Roi/tsg-101 (RNAi)/Gut close up/ART DIC CU2 ERT261 GFPubq tsg101 RNAi front_0022-1-1-1-1-1.tif]

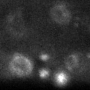

Supplement: Supplementary file 6 — Source data Fig. 4 [file 44318_2025_367_MOESM6_ESM.zip › SD figure 4/4A/Fig_4_A_Roi/tsg-101 (RNAi)/Gut close up/ART GFP CU ERT261 GFPubq tsg101 RNAi front_0022-1-1-1-1-1.tif]

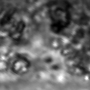

Supplement: Supplementary file 6 — Source data Fig. 4 [file 44318_2025_367_MOESM6_ESM.zip › SD figure 4/4A/Fig_4_A_Roi/tsg-101 (RNAi)/Gut close up/ART DIC CU ERT261 GFPubq tsg101 RNAi front_0022-1-1-1-1-1.tif]

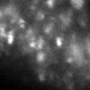

Supplement: Supplementary file 6 — Source data Fig. 4 [file 44318_2025_367_MOESM6_ESM.zip › SD figure 4/4A/Fig_4_A_Roi/tsg-101 (RNAi)/Gut close up/ART GFP CU2 ERT261 GFPubq tsg101 RNAi front_0022-1-1-1-1-1.tif]

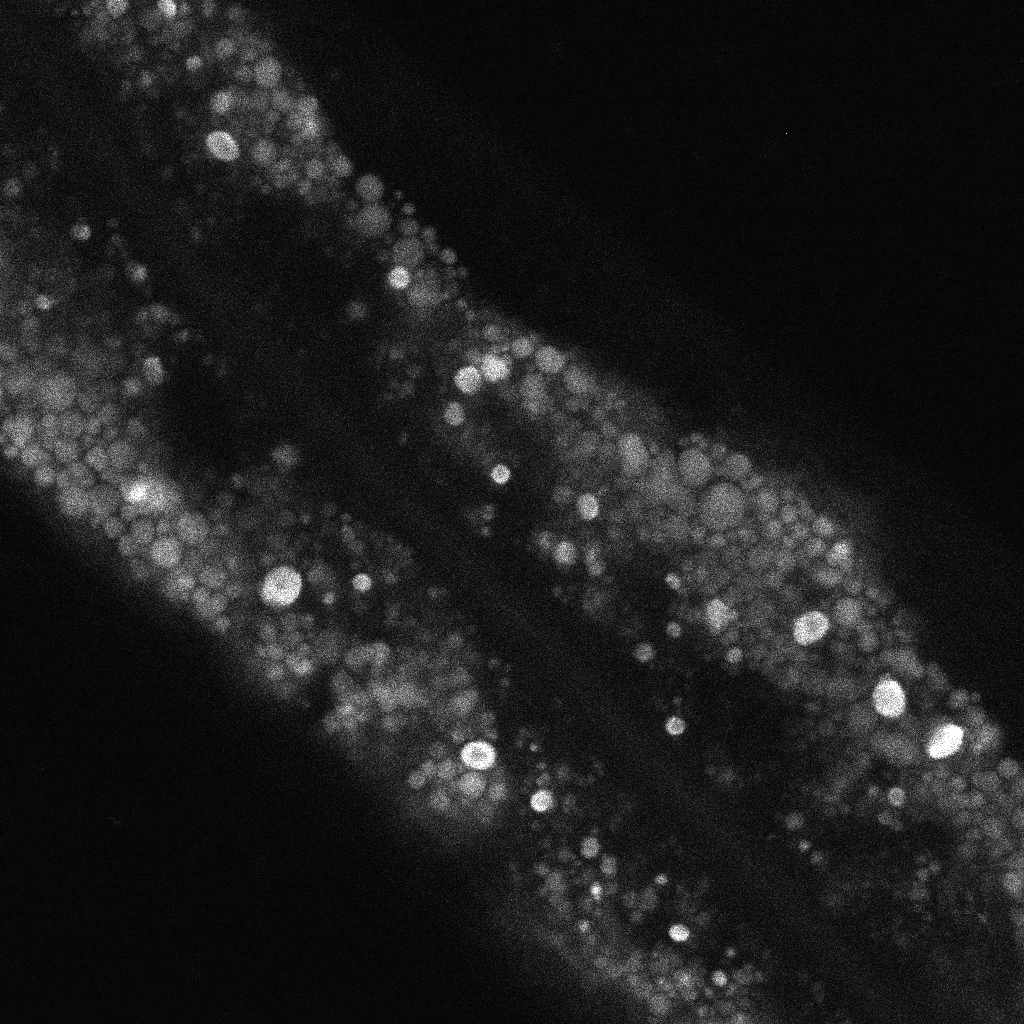

Supplement: Supplementary file 7 — Source data Fig. 5 [file 44318_2025_367_MOESM7_ESM.zip › SD figure 5/5I/Fig_5_I_data/ubq-1 (RNAi)/A sand1 ok1963 lmp1GFP RAB7mCherrz ubq1 and control RNAi 1 to 250 front_0018-1.tif]

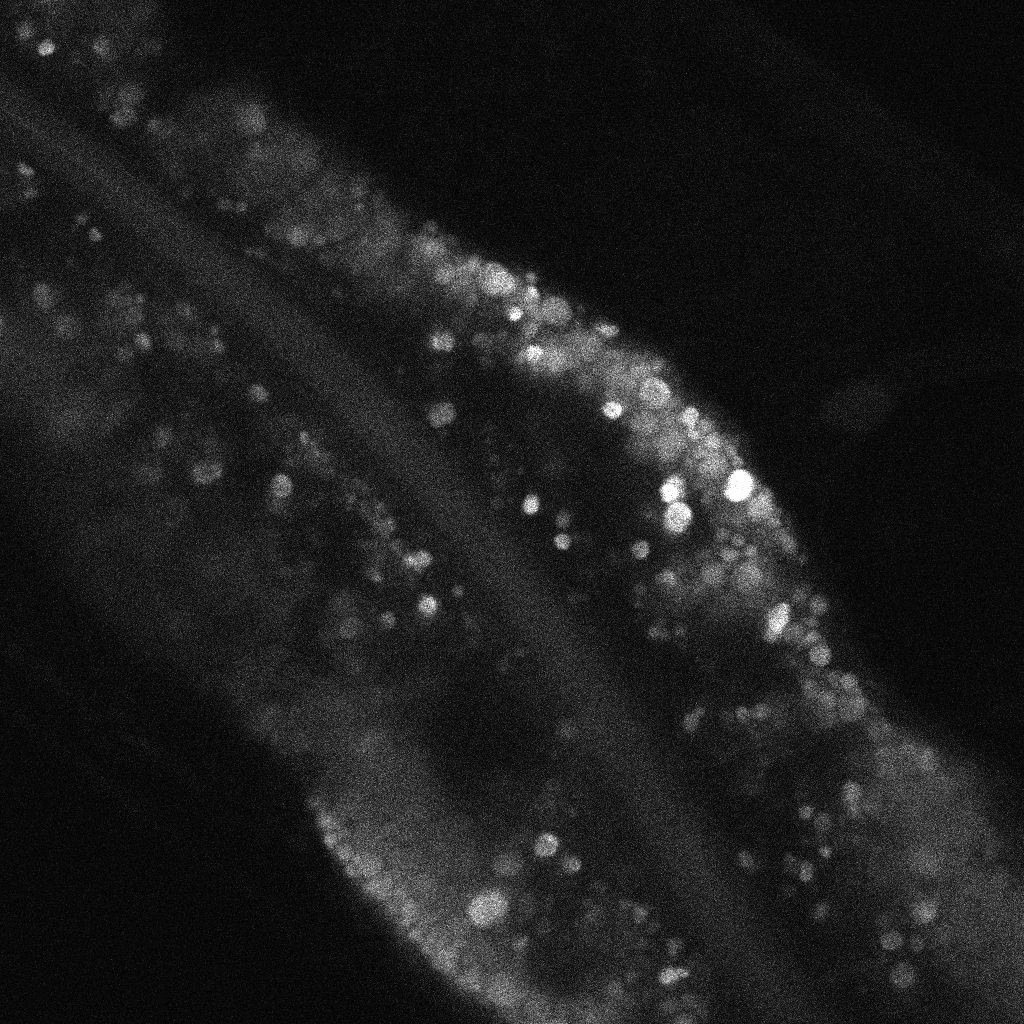

Supplement: Supplementary file 7 — Source data Fig. 5 [file 44318_2025_367_MOESM7_ESM.zip › SD figure 5/5I/Fig_5_I_data/Mock/A sand1 ok1963 lmp1GFP rab7mCherrz control RNAi front_0019-1.tif]

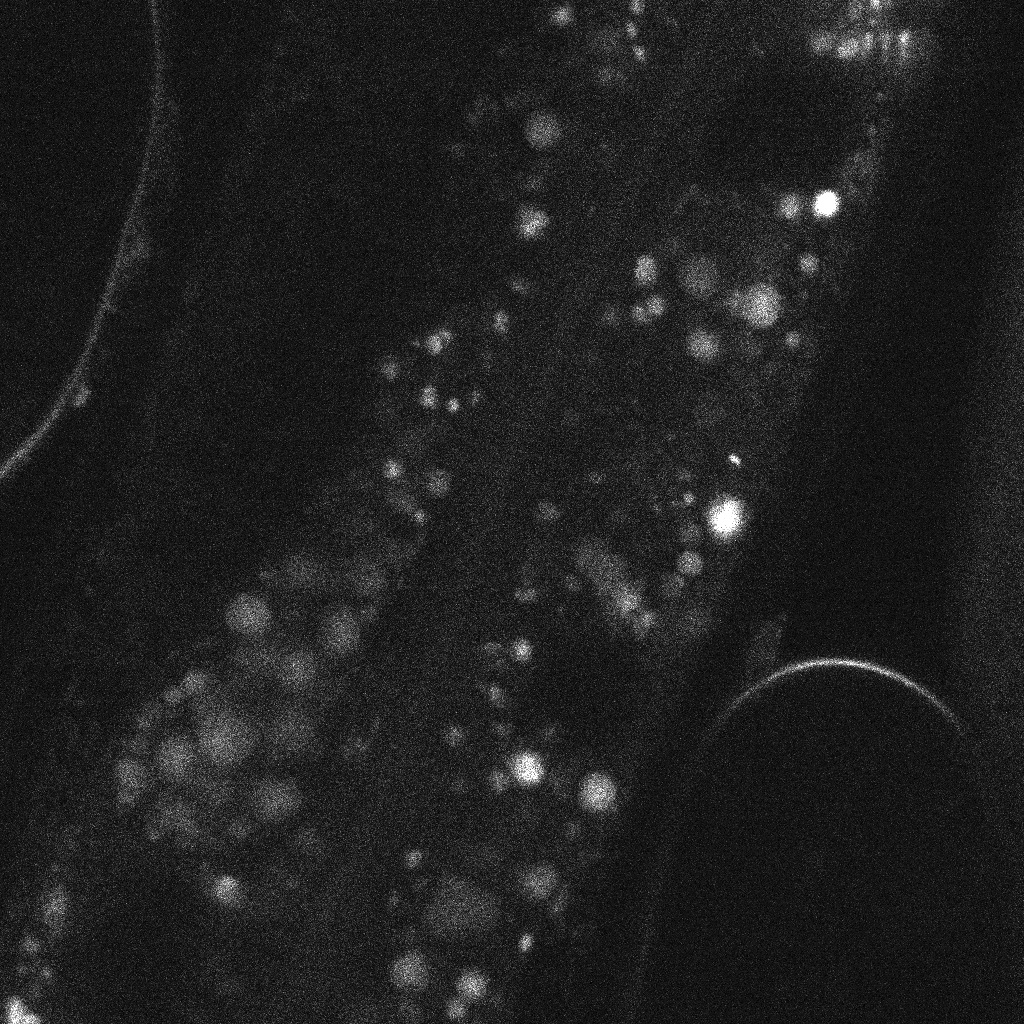

Supplement: Supplementary file 7 — Source data Fig. 5 [file 44318_2025_367_MOESM7_ESM.zip › SD figure 5/5A/Fig_5_A_data/ubq-1 (RNAi)/A GFPRab5 mCherrzRab7 sand1 ok1963 ubq1 and control RNAi 1 to 250 front_0013-1.tif]

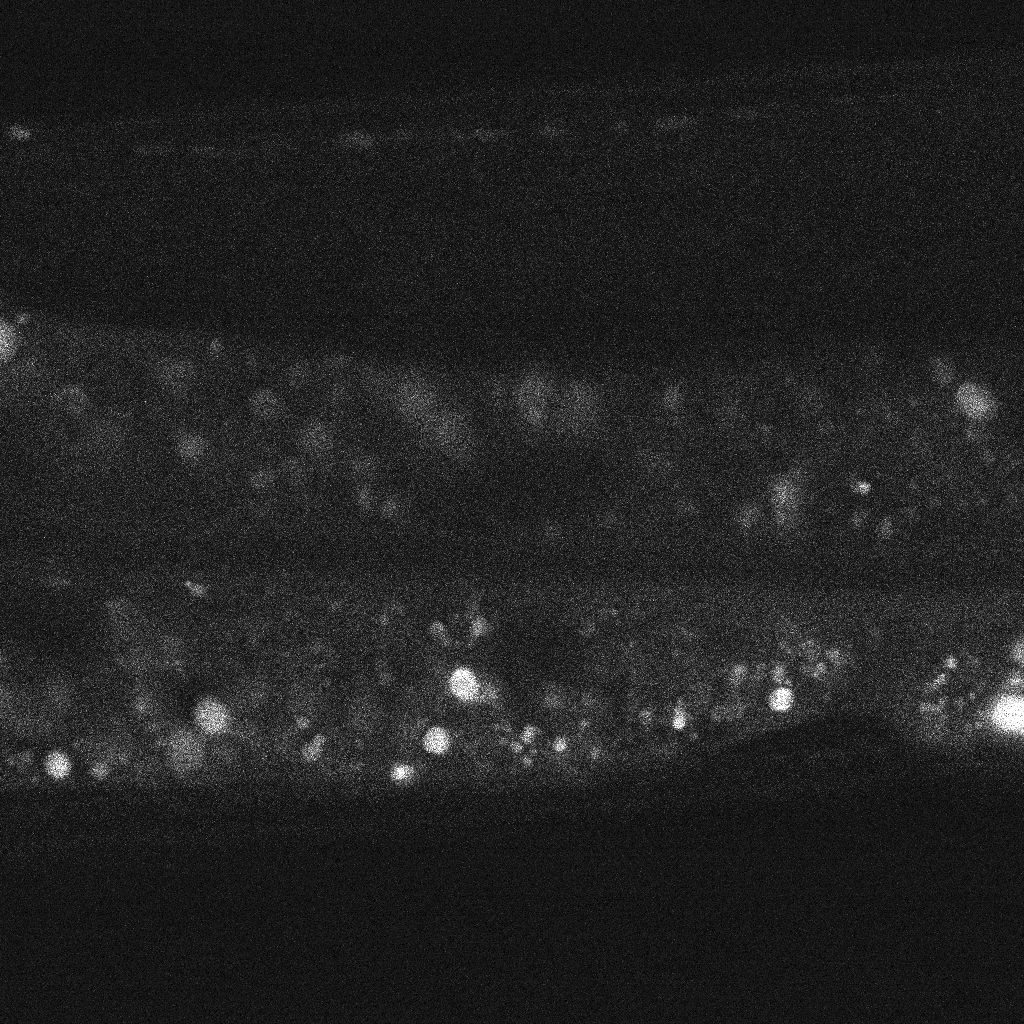

Supplement: Supplementary file 7 — Source data Fig. 5 [file 44318_2025_367_MOESM7_ESM.zip › SD figure 5/5A/Fig_5_A_data/Mock/A GFPRab5 mCherrzRab7 sand1 ok1963 control RNAi front_0011-1.tif]

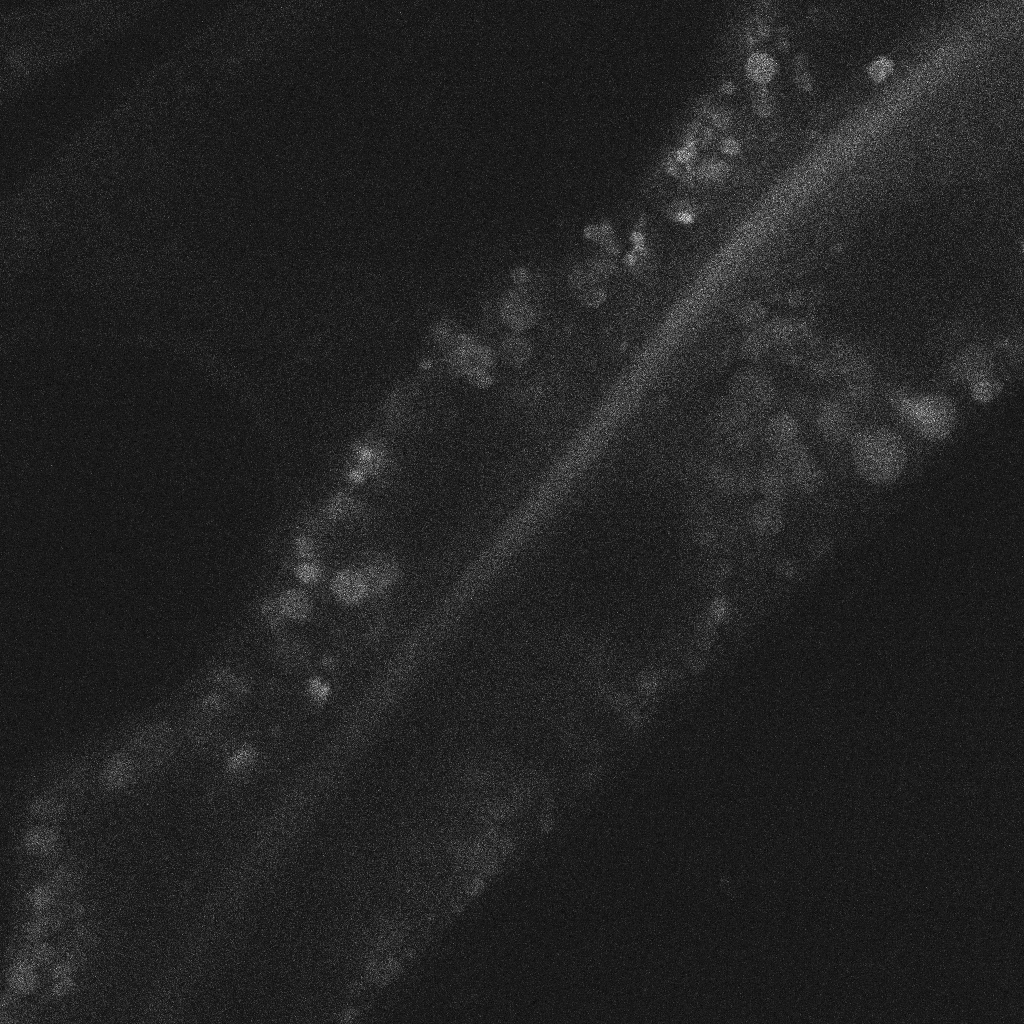

Supplement: Supplementary file 7 — Source data Fig. 5 [file 44318_2025_367_MOESM7_ESM.zip › SD figure 5/5D/Fig_5_D_data/ubq-1 (RNAi)/A htfrGP rab7mCherrz sand1 ok1963 ubq1 and control RNAi 1 to 250 front_0014-1.tif]

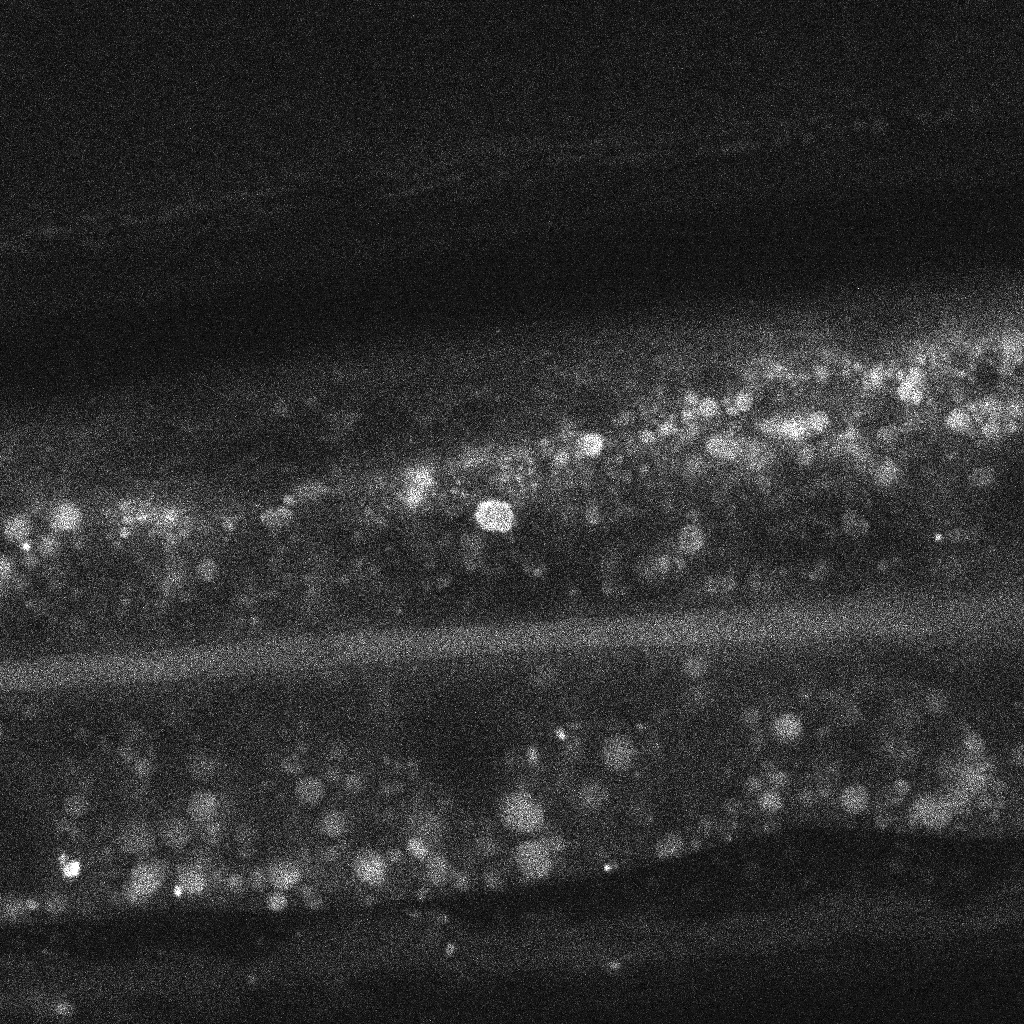

Supplement: Supplementary file 7 — Source data Fig. 5 [file 44318_2025_367_MOESM7_ESM.zip › SD figure 5/5D/Fig_5_D_data/Mock/A htfrGP rab7mCherrz sand1 ok1963 control RNAi front_0006-1.tif]

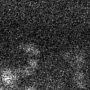

Supplement: Supplementary file 7 — Source data Fig. 5 [file 44318_2025_367_MOESM7_ESM.zip › SD figure 5/5I/Fig_5_I_Roi/ubq-1 (RNAi)/Gut close up/ART C AF sand1 ok1963 lmp1GFP RAB7mCherrz ubq1 and control RNAi 1 to 250 front_0018-1-1-1-1-1.tif]

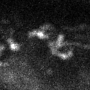

Supplement: Supplementary file 7 — Source data Fig. 5 [file 44318_2025_367_MOESM7_ESM.zip › SD figure 5/5I/Fig_5_I_Roi/ubq-1 (RNAi)/Gut close up/ART C MC sand1 ok1963 lmp1GFP RAB7mCherrz ubq1 and control RNAi 1 to 250 front_0018-1-1-1-1-1.tif]

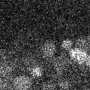

Supplement: Supplementary file 7 — Source data Fig. 5 [file 44318_2025_367_MOESM7_ESM.zip › SD figure 5/5I/Fig_5_I_Roi/ubq-1 (RNAi)/Gut close up/ART C2 MA sand1 ok1963 lmp1GFP RAB7mCherrz ubq1 and control RNAi 1 to 250 front_0018-1-1-1-1.tif]

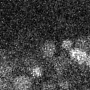

Supplement: Supplementary file 7 — Source data Fig. 5 [file 44318_2025_367_MOESM7_ESM.zip › SD figure 5/5I/Fig_5_I_Roi/ubq-1 (RNAi)/Gut close up/ART C2 AF sand1 ok1963 lmp1GFP RAB7mCherrz ubq1 and control RNAi 1 to 250 front_0018-1-1-1-1-1.tif]

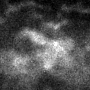

Supplement: Supplementary file 7 — Source data Fig. 5 [file 44318_2025_367_MOESM7_ESM.zip › SD figure 5/5I/Fig_5_I_Roi/ubq-1 (RNAi)/Gut close up/ART C2 G sand1 ok1963 lmp1GFP RAB7mCherrz ubq1 and control RNAi 1 to 250 front_0018-1-1-1-1-1.tif]

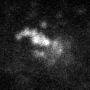

Supplement: Supplementary file 7 — Source data Fig. 5 [file 44318_2025_367_MOESM7_ESM.zip › SD figure 5/5I/Fig_5_I_Roi/ubq-1 (RNAi)/Gut close up/ART C2 MC sand1 ok1963 lmp1GFP RAB7mCherrz ubq1 and control RNAi 1 to 250 front_0018-1-1-1-1-1.tif]

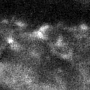

Supplement: Supplementary file 7 — Source data Fig. 5 [file 44318_2025_367_MOESM7_ESM.zip › SD figure 5/5I/Fig_5_I_Roi/ubq-1 (RNAi)/Gut close up/ART C G sand1 ok1963 lmp1GFP RAB7mCherrz ubq1 and control RNAi 1 to 250 front_0018-1-1-1-1-1.tif]

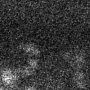

Supplement: Supplementary file 7 — Source data Fig. 5 [file 44318_2025_367_MOESM7_ESM.zip › SD figure 5/5I/Fig_5_I_Roi/ubq-1 (RNAi)/Gut close up/ART C MA sand1 ok1963 lmp1GFP RAB7mCherrz ubq1 and control RNAi 1 to 250 front_0018-1-1-1-1.tif]

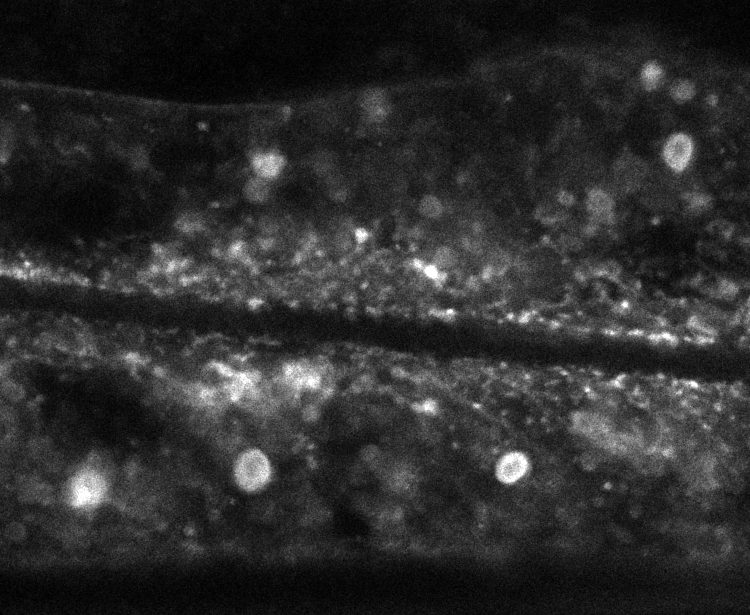

Supplement: Supplementary file 7 — Source data Fig. 5 [file 44318_2025_367_MOESM7_ESM.zip › SD figure 5/5I/Fig_5_I_Roi/ubq-1 (RNAi)/Gut/ART G sand1 ok1963 lmp1GFP RAB7mCherrz ubq1 and control RNAi 1 to 250 front_0018-1-1-1-1.tif]

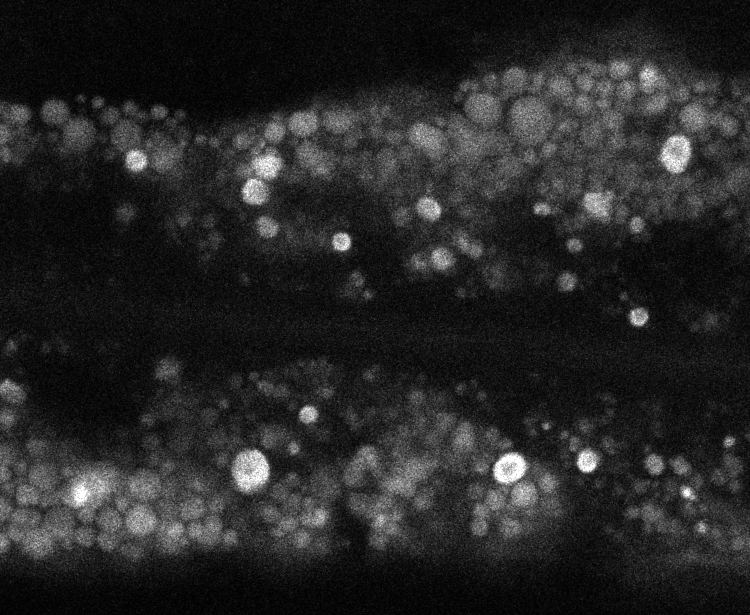

Supplement: Supplementary file 7 — Source data Fig. 5 [file 44318_2025_367_MOESM7_ESM.zip › SD figure 5/5I/Fig_5_I_Roi/ubq-1 (RNAi)/Gut/ART AF sand1 ok1963 lmp1GFP RAB7mCherrz ubq1 and control RNAi 1 to 250 front_0018-1-1-1-1.tif]

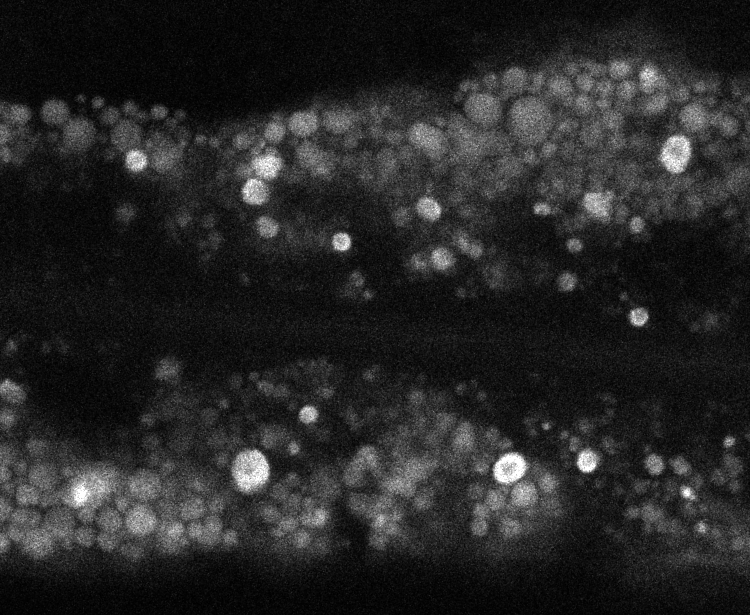

Supplement: Supplementary file 7 — Source data Fig. 5 [file 44318_2025_367_MOESM7_ESM.zip › SD figure 5/5I/Fig_5_I_Roi/ubq-1 (RNAi)/Gut/ART MA sand1 ok1963 lmp1GFP RAB7mCherrz ubq1 and control RNAi 1 to 250 front_0018-1-1-1.tif]

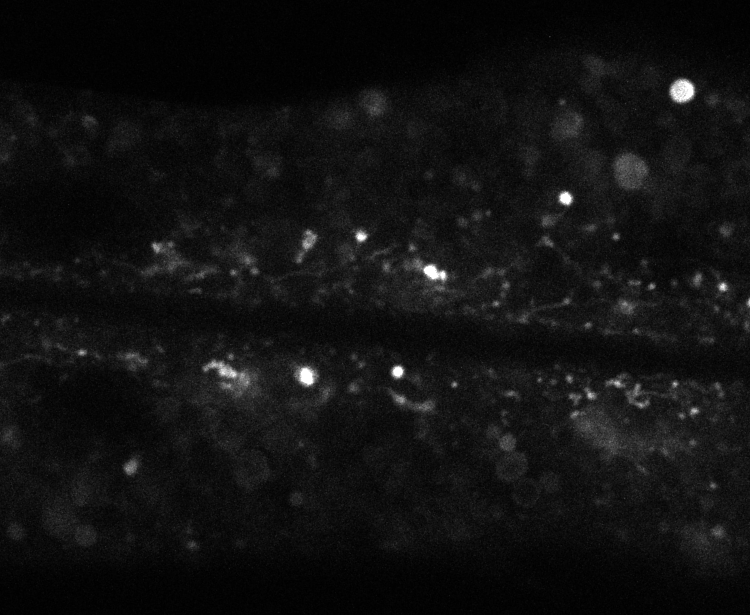

Supplement: Supplementary file 7 — Source data Fig. 5 [file 44318_2025_367_MOESM7_ESM.zip › SD figure 5/5I/Fig_5_I_Roi/ubq-1 (RNAi)/Gut/ART MC sand1 ok1963 lmp1GFP RAB7mCherrz ubq1 and control RNAi 1 to 250 front_0018-1-1-1-1.tif]

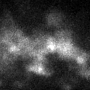

Supplement: Supplementary file 7 — Source data Fig. 5 [file 44318_2025_367_MOESM7_ESM.zip › SD figure 5/5I/Fig_5_I_Roi/Mock/Gut close up/ART C G sand1 ok1963 lmp1GFP rab7mCherrz control RNAi front_0019-1-1-1-1-1.tif]

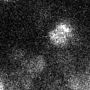

Supplement: Supplementary file 7 — Source data Fig. 5 [file 44318_2025_367_MOESM7_ESM.zip › SD figure 5/5I/Fig_5_I_Roi/Mock/Gut close up/ART C2 AF sand1 ok1963 lmp1GFP rab7mCherrz control RNAi front_0019-1-1-1-1-1.tif]

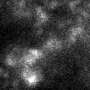

Supplement: Supplementary file 7 — Source data Fig. 5 [file 44318_2025_367_MOESM7_ESM.zip › SD figure 5/5I/Fig_5_I_Roi/Mock/Gut close up/ART C2 G sand1 ok1963 lmp1GFP rab7mCherrz control RNAi front_0019-1-1-1-1-1.tif]
