# Supplementary figures and images for: Coordination between ESCRT function and Rab conversion during endosome maturation (part 4 of 9)
Source: EMBO J. 2025 Feb 5;44(6):1574–607. doi: 10.1038/s44318-025-00367-7 (PMC11914609; doi:10.1038/s44318-025-00367-7)

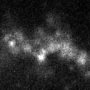

Supplement: Supplementary file 7 — Source data Fig. 5 [file 44318_2025_367_MOESM7_ESM.zip › SD figure 5/5I/Fig_5_I_Roi/Mock/Gut close up/ART C MC sand1 ok1963 lmp1GFP rab7mCherrz control RNAi front_0019-1-1-1-1-1.tif]

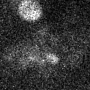

Supplement: Supplementary file 7 — Source data Fig. 5 [file 44318_2025_367_MOESM7_ESM.zip › SD figure 5/5I/Fig_5_I_Roi/Mock/Gut close up/ART C AF sand1 ok1963 lmp1GFP rab7mCherrz control RNAi front_0019-1-1-1-1-1.tif]

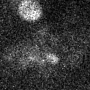

Supplement: Supplementary file 7 — Source data Fig. 5 [file 44318_2025_367_MOESM7_ESM.zip › SD figure 5/5I/Fig_5_I_Roi/Mock/Gut close up/ART C MA sand1 ok1963 lmp1GFP rab7mCherrz control RNAi front_0019-1-1-1-1.tif]

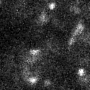

Supplement: Supplementary file 7 — Source data Fig. 5 [file 44318_2025_367_MOESM7_ESM.zip › SD figure 5/5I/Fig_5_I_Roi/Mock/Gut close up/ART C2 MC sand1 ok1963 lmp1GFP rab7mCherrz control RNAi front_0019-1-1-1-1-1.tif]

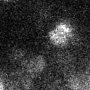

Supplement: Supplementary file 7 — Source data Fig. 5 [file 44318_2025_367_MOESM7_ESM.zip › SD figure 5/5I/Fig_5_I_Roi/Mock/Gut close up/ART C2 MA sand1 ok1963 lmp1GFP rab7mCherrz control RNAi front_0019-1-1-1-1.tif]

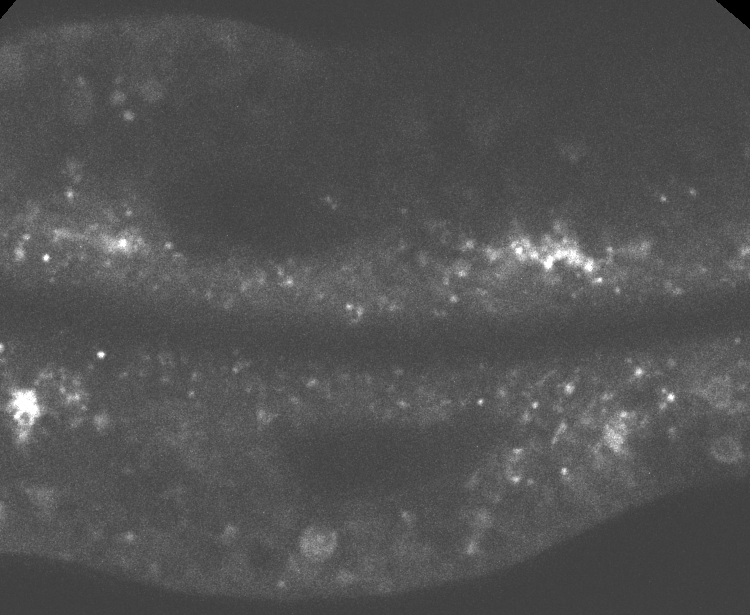

Supplement: Supplementary file 7 — Source data Fig. 5 [file 44318_2025_367_MOESM7_ESM.zip › SD figure 5/5I/Fig_5_I_Roi/Mock/Gut/ART MC sand1 ok1963 lmp1GFP rab7mCherrz control RNAi front_0019-1-1-1-1.tif]

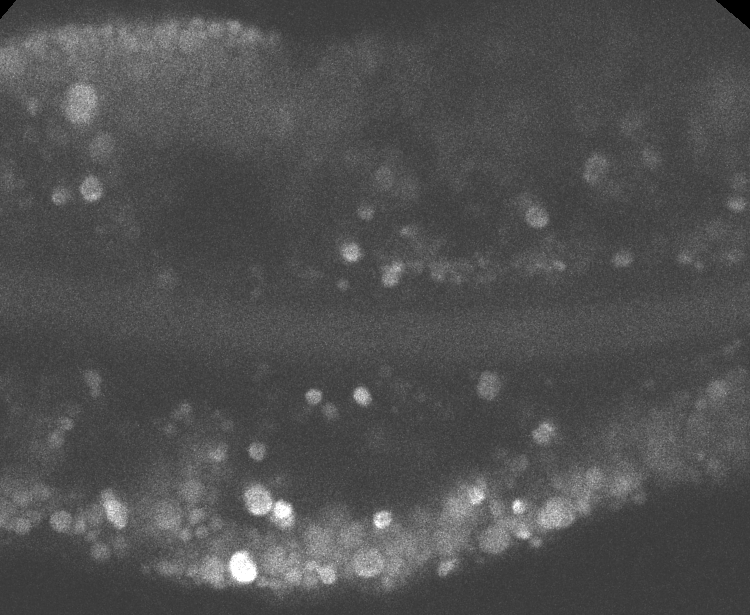

Supplement: Supplementary file 7 — Source data Fig. 5 [file 44318_2025_367_MOESM7_ESM.zip › SD figure 5/5I/Fig_5_I_Roi/Mock/Gut/ART MA sand1 ok1963 lmp1GFP rab7mCherrz control RNAi front_0019-1-1-1.tif]

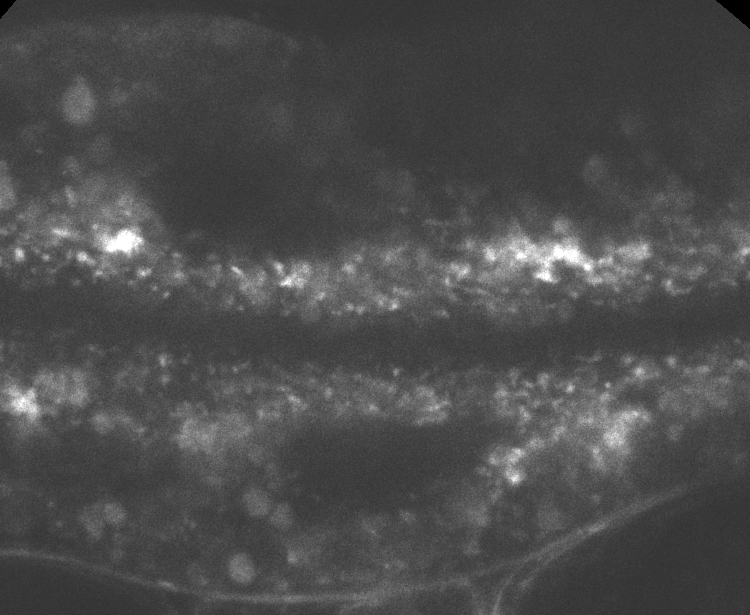

Supplement: Supplementary file 7 — Source data Fig. 5 [file 44318_2025_367_MOESM7_ESM.zip › SD figure 5/5I/Fig_5_I_Roi/Mock/Gut/ART G sand1 ok1963 lmp1GFP rab7mCherrz control RNAi front_0019-1-1-1-1.tif]

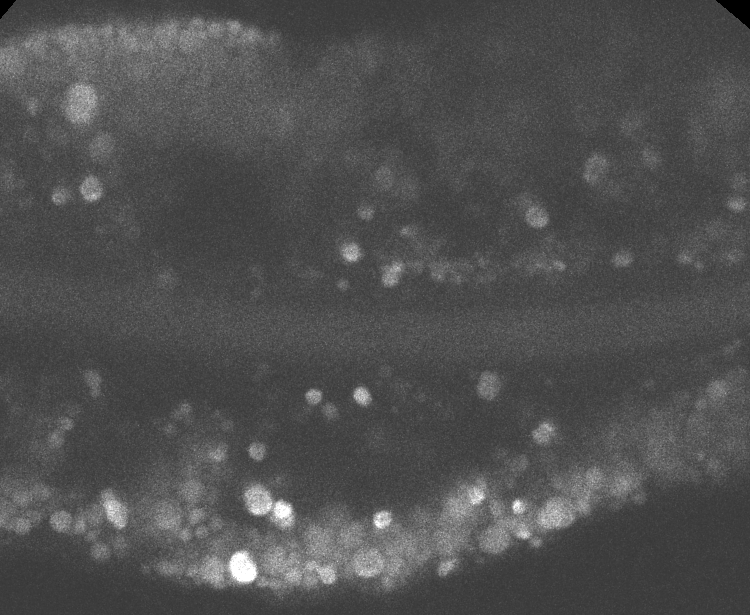

Supplement: Supplementary file 7 — Source data Fig. 5 [file 44318_2025_367_MOESM7_ESM.zip › SD figure 5/5I/Fig_5_I_Roi/Mock/Gut/ART AF sand1 ok1963 lmp1GFP rab7mCherrz control RNAi front_0019-1-1-1-1.tif]

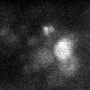

Supplement: Supplementary file 7 — Source data Fig. 5 [file 44318_2025_367_MOESM7_ESM.zip › SD figure 5/5A/Fig_5_A_Roi/ubq-1 (RNAi)/Gut close up/ART C2 G GFPRab5 mCherrzRab7 sand1 ok1963 ubq1 and control RNAi 1 to 250 front_0013-1-1-1-1-1.tif]

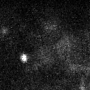

Supplement: Supplementary file 7 — Source data Fig. 5 [file 44318_2025_367_MOESM7_ESM.zip › SD figure 5/5A/Fig_5_A_Roi/ubq-1 (RNAi)/Gut close up/ART C2 MGM GFPRab5 mCherrzRab7 sand1 ok1963 ubq1 and control RNAi 1 to 250 front_0013-1-1-1-1-1.tif]

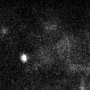

Supplement: Supplementary file 7 — Source data Fig. 5 [file 44318_2025_367_MOESM7_ESM.zip › SD figure 5/5A/Fig_5_A_Roi/ubq-1 (RNAi)/Gut close up/ART C2 MC GFPRab5 mCherrzRab7 sand1 ok1963 ubq1 and control RNAi 1 to 250 front_0013-1-1-1-1-1.tif]

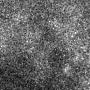

Supplement: Supplementary file 7 — Source data Fig. 5 [file 44318_2025_367_MOESM7_ESM.zip › SD figure 5/5A/Fig_5_A_Roi/ubq-1 (RNAi)/Gut close up/ART C G GFPRab5 mCherrzRab7 sand1 ok1963 ubq1 and control RNAi 1 to 250 front_0013-1-1-1-1-1.tif]

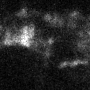

Supplement: Supplementary file 7 — Source data Fig. 5 [file 44318_2025_367_MOESM7_ESM.zip › SD figure 5/5A/Fig_5_A_Roi/ubq-1 (RNAi)/Gut close up/ART C MC GFPRab5 mCherrzRab7 sand1 ok1963 ubq1 and control RNAi 1 to 250 front_0013-1-1-1-1-1.tif]

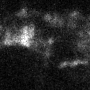

Supplement: Supplementary file 7 — Source data Fig. 5 [file 44318_2025_367_MOESM7_ESM.zip › SD figure 5/5A/Fig_5_A_Roi/ubq-1 (RNAi)/Gut close up/ART C MGM GFPRab5 mCherrzRab7 sand1 ok1963 ubq1 and control RNAi 1 to 250 front_0013-1-1-1-1-1.tif]

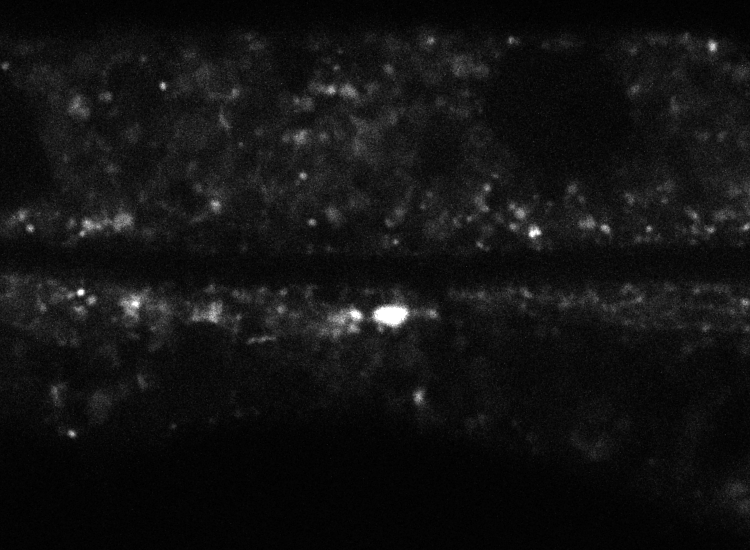

Supplement: Supplementary file 7 — Source data Fig. 5 [file 44318_2025_367_MOESM7_ESM.zip › SD figure 5/5A/Fig_5_A_Roi/ubq-1 (RNAi)/Gut/ART MC GFPRab5 mCherrzRab7 sand1 ok1963 ubq1 and control RNAi 1 to 250 front_0013-1-1-1-1.tif]

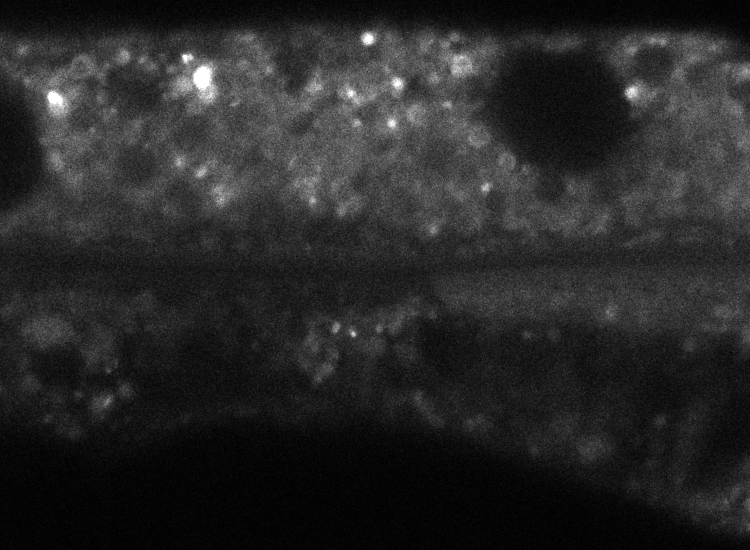

Supplement: Supplementary file 7 — Source data Fig. 5 [file 44318_2025_367_MOESM7_ESM.zip › SD figure 5/5A/Fig_5_A_Roi/ubq-1 (RNAi)/Gut/ART G GFPRab5 mCherrzRab7 sand1 ok1963 ubq1 and control RNAi 1 to 250 front_0013-1-1-1-1.tif]

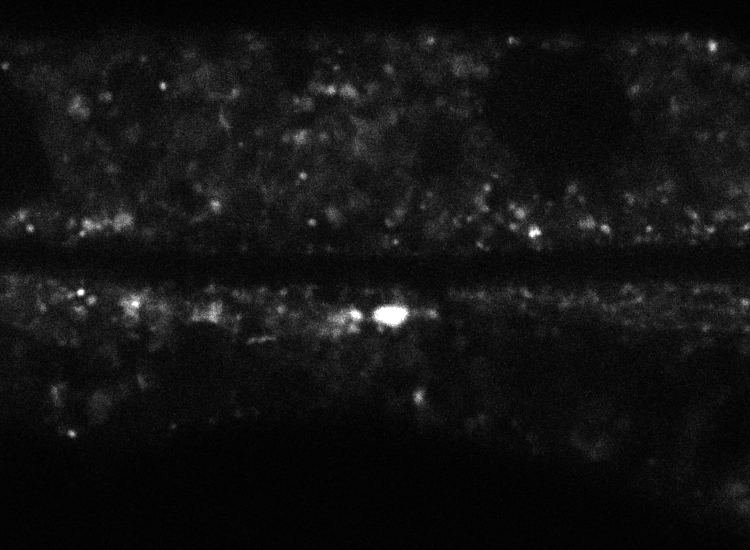

Supplement: Supplementary file 7 — Source data Fig. 5 [file 44318_2025_367_MOESM7_ESM.zip › SD figure 5/5A/Fig_5_A_Roi/ubq-1 (RNAi)/Gut/ART MGM GFPRab5 mCherrzRab7 sand1 ok1963 ubq1 and control RNAi 1 to 250 front_0013-1-1-1-1.tif]

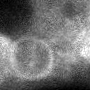

Supplement: Supplementary file 7 — Source data Fig. 5 [file 44318_2025_367_MOESM7_ESM.zip › SD figure 5/5A/Fig_5_A_Roi/Mock/Gut close up/ART C2 G GFPRab5 mCherrzRab7 sand1 ok1963 control RNAi front_0011-1-1-1-1-1.tif]

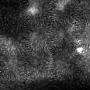

Supplement: Supplementary file 7 — Source data Fig. 5 [file 44318_2025_367_MOESM7_ESM.zip › SD figure 5/5A/Fig_5_A_Roi/Mock/Gut close up/ART C2 MGM GFPRab5 mCherrzRab7 sand1 ok1963 control RNAi front_0011-1-1-1-1-1.tif]

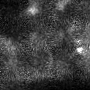

Supplement: Supplementary file 7 — Source data Fig. 5 [file 44318_2025_367_MOESM7_ESM.zip › SD figure 5/5A/Fig_5_A_Roi/Mock/Gut close up/ART C2 MC GFPRab5 mCherrzRab7 sand1 ok1963 control RNAi front_0011-1-1-1-1-1.tif]

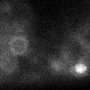

Supplement: Supplementary file 7 — Source data Fig. 5 [file 44318_2025_367_MOESM7_ESM.zip › SD figure 5/5A/Fig_5_A_Roi/Mock/Gut close up/ART C G GFPRab5 mCherrzRab7 sand1 ok1963 control RNAi front_0011-1-1-1-1-1.tif]

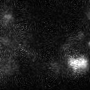

Supplement: Supplementary file 7 — Source data Fig. 5 [file 44318_2025_367_MOESM7_ESM.zip › SD figure 5/5A/Fig_5_A_Roi/Mock/Gut close up/ART C MGM GFPRab5 mCherrzRab7 sand1 ok1963 control RNAi front_0011-1-1-1-1-1.tif]

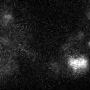

Supplement: Supplementary file 7 — Source data Fig. 5 [file 44318_2025_367_MOESM7_ESM.zip › SD figure 5/5A/Fig_5_A_Roi/Mock/Gut close up/ART C MC GFPRab5 mCherrzRab7 sand1 ok1963 control RNAi front_0011-1-1-1-1-1.tif]

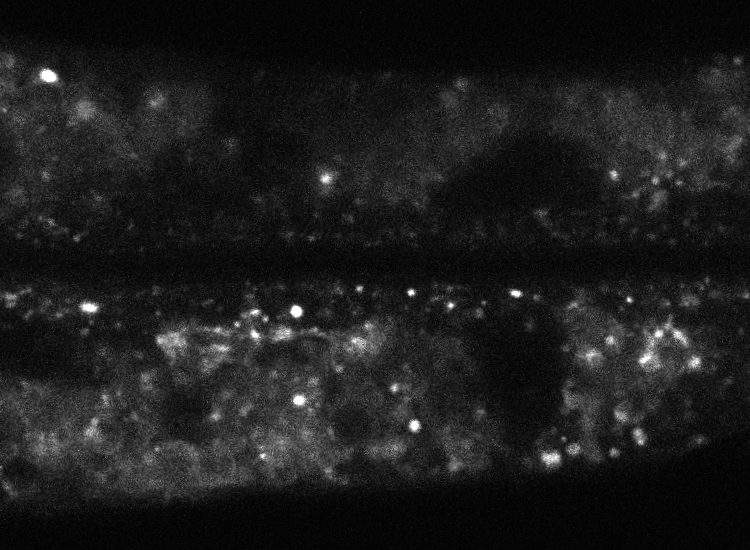

Supplement: Supplementary file 7 — Source data Fig. 5 [file 44318_2025_367_MOESM7_ESM.zip › SD figure 5/5A/Fig_5_A_Roi/Mock/Gut/ART MGM GFPRab5 mCherrzRab7 sand1 ok1963 control RNAi front_0011-1-1-1-1.tif]

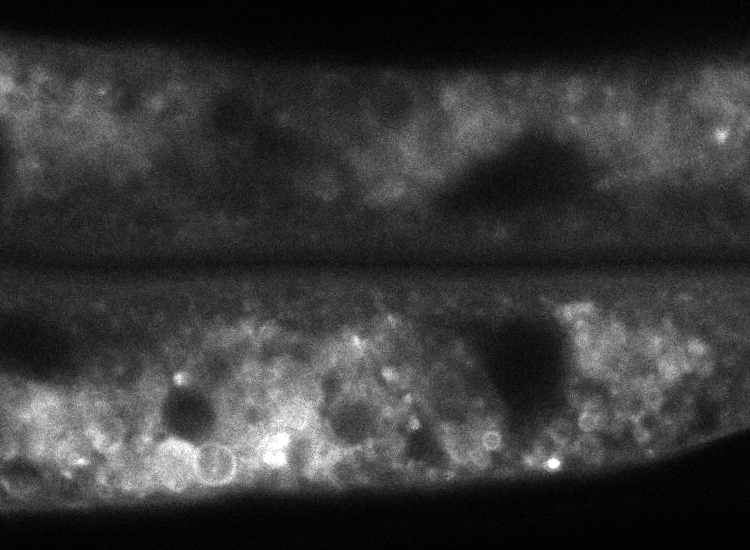

Supplement: Supplementary file 7 — Source data Fig. 5 [file 44318_2025_367_MOESM7_ESM.zip › SD figure 5/5A/Fig_5_A_Roi/Mock/Gut/ART G GFPRab5 mCherrzRab7 sand1 ok1963 control RNAi front_0011-1-1-1-1.tif]

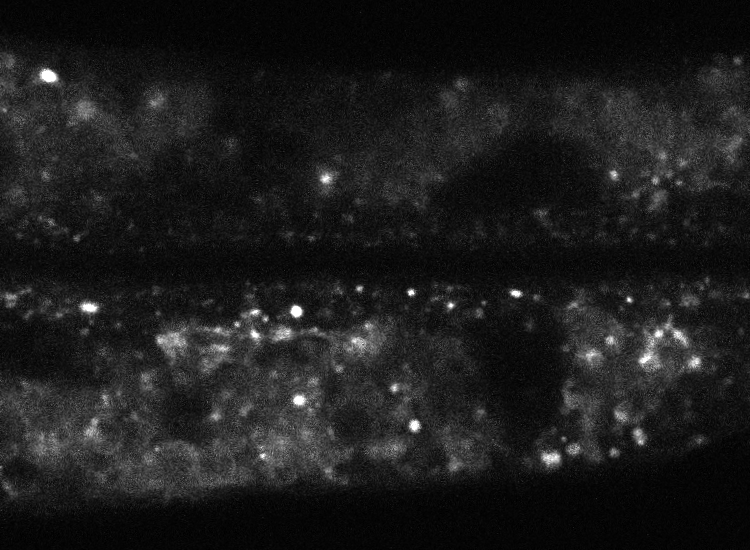

Supplement: Supplementary file 7 — Source data Fig. 5 [file 44318_2025_367_MOESM7_ESM.zip › SD figure 5/5A/Fig_5_A_Roi/Mock/Gut/ART MC GFPRab5 mCherrzRab7 sand1 ok1963 control RNAi front_0011-1-1-1-1.tif]

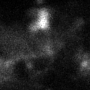

Supplement: Supplementary file 7 — Source data Fig. 5 [file 44318_2025_367_MOESM7_ESM.zip › SD figure 5/5D/Fig_5_D_Roi/ubq-1 (RNAi)/Gut close up/ART C MC htfrGP rab7mCherrz sand1 ok1963 ubq1 and control RNAi 1 to 250 front_0014-1-1-1-1-1.tif]

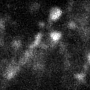

Supplement: Supplementary file 7 — Source data Fig. 5 [file 44318_2025_367_MOESM7_ESM.zip › SD figure 5/5D/Fig_5_D_Roi/ubq-1 (RNAi)/Gut close up/ART C2 MGM htfrGP rab7mCherrz sand1 ok1963 ubq1 and control RNAi 1 to 250 front_0014-1-1-1-1-1.tif]

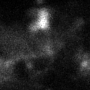

Supplement: Supplementary file 7 — Source data Fig. 5 [file 44318_2025_367_MOESM7_ESM.zip › SD figure 5/5D/Fig_5_D_Roi/ubq-1 (RNAi)/Gut close up/ART C MGM htfrGP rab7mCherrz sand1 ok1963 ubq1 and control RNAi 1 to 250 front_0014-1-1-1-1-1.tif]

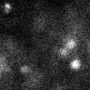

Supplement: Supplementary file 7 — Source data Fig. 5 [file 44318_2025_367_MOESM7_ESM.zip › SD figure 5/5D/Fig_5_D_Roi/ubq-1 (RNAi)/Gut close up/ART C G htfrGP rab7mCherrz sand1 ok1963 ubq1 and control RNAi 1 to 250 front_0014-1-1-1-1-1.tif]

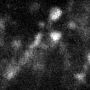

Supplement: Supplementary file 7 — Source data Fig. 5 [file 44318_2025_367_MOESM7_ESM.zip › SD figure 5/5D/Fig_5_D_Roi/ubq-1 (RNAi)/Gut close up/ART C2 MC htfrGP rab7mCherrz sand1 ok1963 ubq1 and control RNAi 1 to 250 front_0014-1-1-1-1-1.tif]

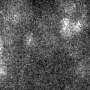

Supplement: Supplementary file 7 — Source data Fig. 5 [file 44318_2025_367_MOESM7_ESM.zip › SD figure 5/5D/Fig_5_D_Roi/ubq-1 (RNAi)/Gut close up/ART C2 G htfrGP rab7mCherrz sand1 ok1963 ubq1 and control RNAi 1 to 250 front_0014-1-1-1-1-1.tif]

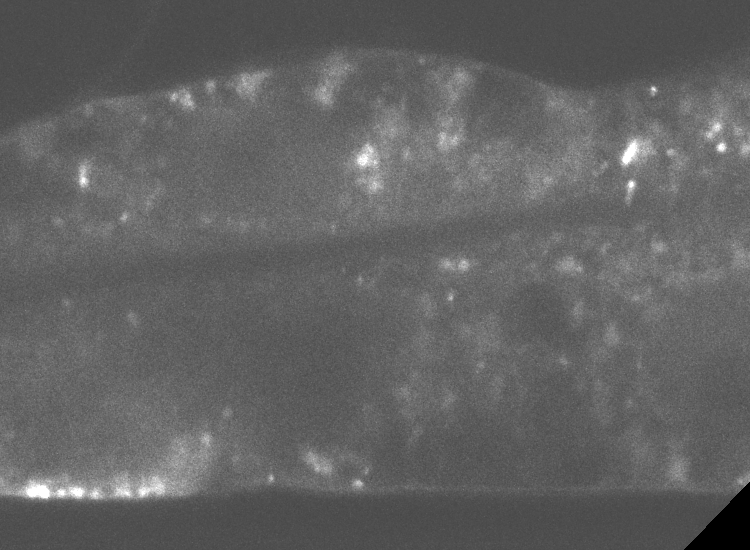

Supplement: Supplementary file 7 — Source data Fig. 5 [file 44318_2025_367_MOESM7_ESM.zip › SD figure 5/5D/Fig_5_D_Roi/ubq-1 (RNAi)/Gut/ART G htfrGP rab7mCherrz sand1 ok1963 ubq1 and control RNAi 1 to 250 front_0014-1-1-1-1.tif]

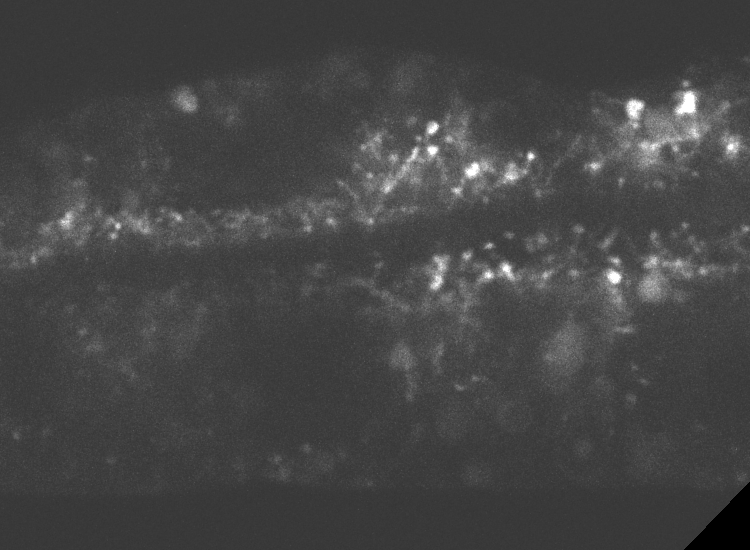

Supplement: Supplementary file 7 — Source data Fig. 5 [file 44318_2025_367_MOESM7_ESM.zip › SD figure 5/5D/Fig_5_D_Roi/ubq-1 (RNAi)/Gut/ART MC htfrGP rab7mCherrz sand1 ok1963 ubq1 and control RNAi 1 to 250 front_0014-1-1-1-1.tif]

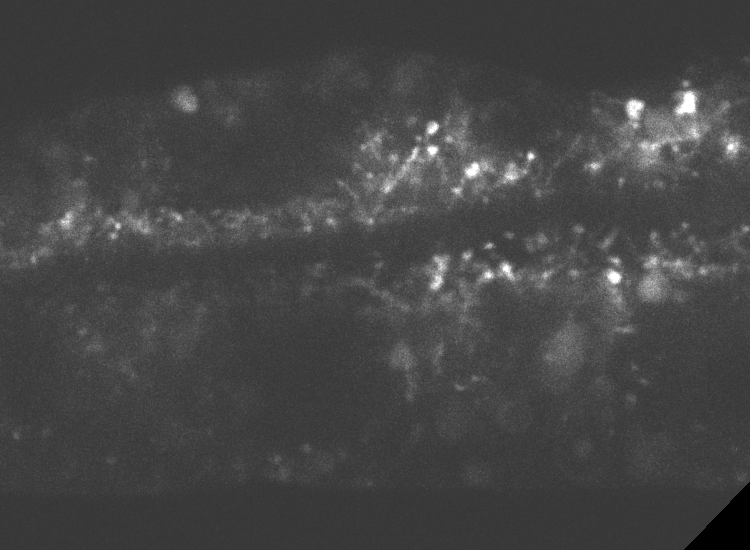

Supplement: Supplementary file 7 — Source data Fig. 5 [file 44318_2025_367_MOESM7_ESM.zip › SD figure 5/5D/Fig_5_D_Roi/ubq-1 (RNAi)/Gut/ART MGM htfrGP rab7mCherrz sand1 ok1963 ubq1 and control RNAi 1 to 250 front_0014-1-1-1-1.tif]

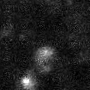

Supplement: Supplementary file 7 — Source data Fig. 5 [file 44318_2025_367_MOESM7_ESM.zip › SD figure 5/5D/Fig_5_D_Roi/Mock/Gut close up/ART C MC htfrGP rab7mCherrz sand1 ok1963 control RNAi front_0006-1-1-1-1-1.tif]

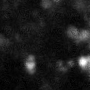

Supplement: Supplementary file 7 — Source data Fig. 5 [file 44318_2025_367_MOESM7_ESM.zip › SD figure 5/5D/Fig_5_D_Roi/Mock/Gut close up/ART C2 G htfrGP rab7mCherrz sand1 ok1963 control RNAi front_0006-1-1-1-1-1.tif]

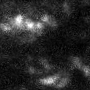

Supplement: Supplementary file 7 — Source data Fig. 5 [file 44318_2025_367_MOESM7_ESM.zip › SD figure 5/5D/Fig_5_D_Roi/Mock/Gut close up/ART C2 MGM htfrGP rab7mCherrz sand1 ok1963 control RNAi front_0006-1-1-1-1-1.tif]

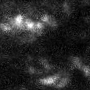

Supplement: Supplementary file 7 — Source data Fig. 5 [file 44318_2025_367_MOESM7_ESM.zip › SD figure 5/5D/Fig_5_D_Roi/Mock/Gut close up/ART C2 MC htfrGP rab7mCherrz sand1 ok1963 control RNAi front_0006-1-1-1-1-1.tif]

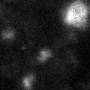

Supplement: Supplementary file 7 — Source data Fig. 5 [file 44318_2025_367_MOESM7_ESM.zip › SD figure 5/5D/Fig_5_D_Roi/Mock/Gut close up/ART C G htfrGP rab7mCherrz sand1 ok1963 control RNAi front_0006-1-1-1-1-1.tif]

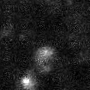

Supplement: Supplementary file 7 — Source data Fig. 5 [file 44318_2025_367_MOESM7_ESM.zip › SD figure 5/5D/Fig_5_D_Roi/Mock/Gut close up/ART C MGM htfrGP rab7mCherrz sand1 ok1963 control RNAi front_0006-1-1-1-1-1.tif]

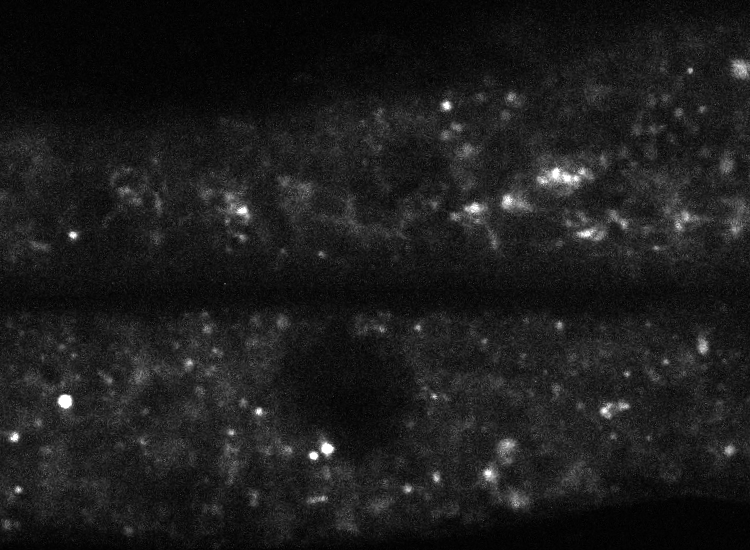

Supplement: Supplementary file 7 — Source data Fig. 5 [file 44318_2025_367_MOESM7_ESM.zip › SD figure 5/5D/Fig_5_D_Roi/Mock/Gut/ART MC htfrGP rab7mCherrz sand1 ok1963 control RNAi front_0006-1-1-1-1.tif]

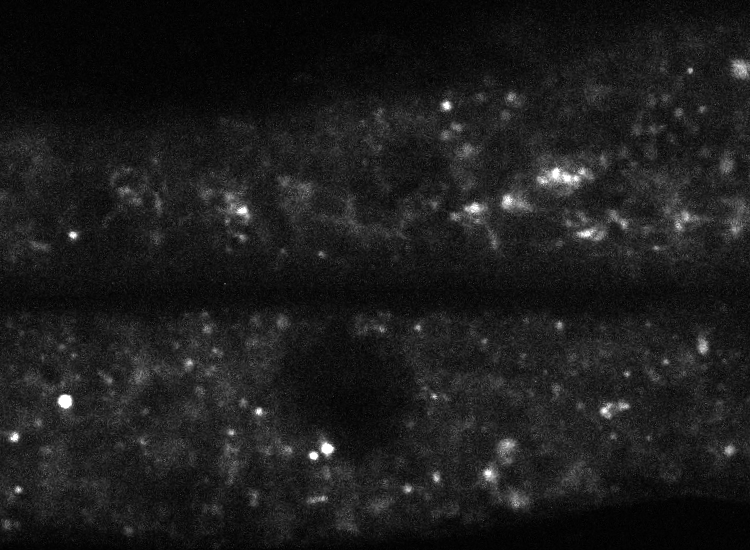

Supplement: Supplementary file 7 — Source data Fig. 5 [file 44318_2025_367_MOESM7_ESM.zip › SD figure 5/5D/Fig_5_D_Roi/Mock/Gut/ART MGM htfrGP rab7mCherrz sand1 ok1963 control RNAi front_0006-1-1-1-1.tif]

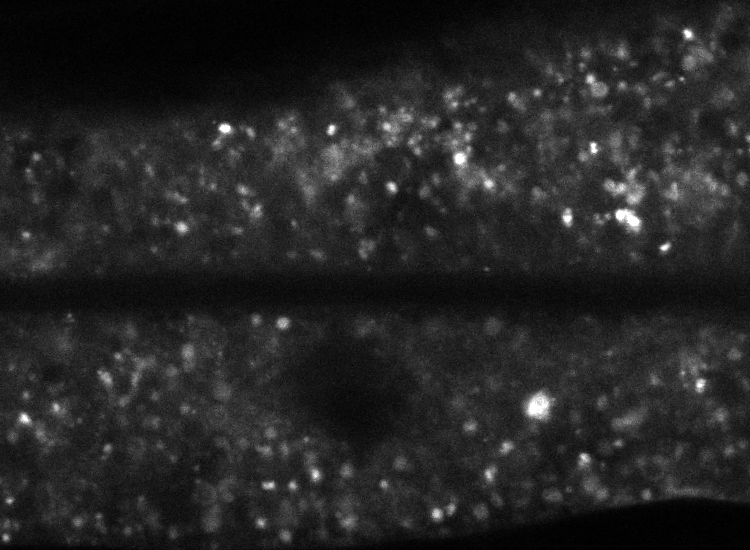

Supplement: Supplementary file 7 — Source data Fig. 5 [file 44318_2025_367_MOESM7_ESM.zip › SD figure 5/5D/Fig_5_D_Roi/Mock/Gut/ART G htfrGP rab7mCherrz sand1 ok1963 control RNAi front_0006-1-1-1-1.tif]

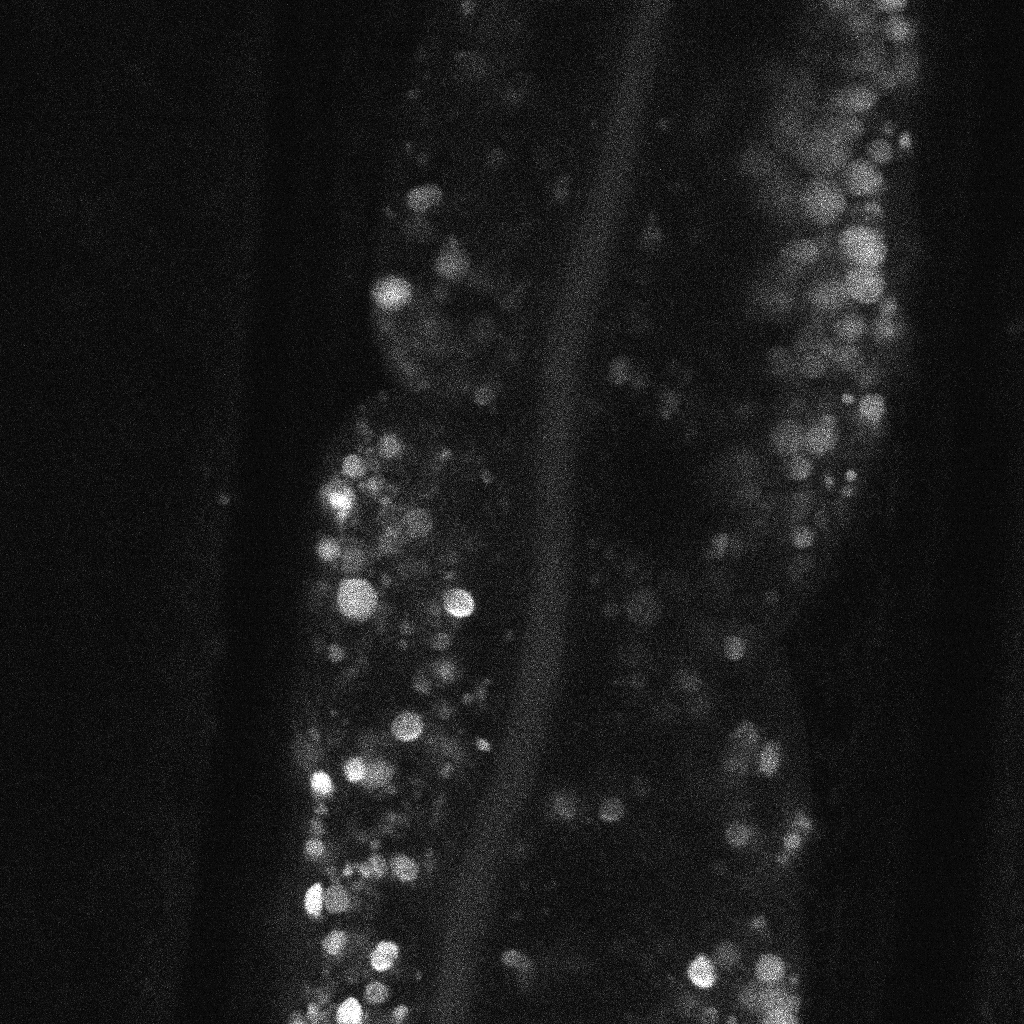

Supplement: Supplementary file 8 — Source data Fig. 6 [file 44318_2025_367_MOESM8_ESM.zip › SD figure 6/6A/Fig_6_A_data/Mock/A GFPRab5 mCherrzRab7 sand1 ok1963 control RNAi front_0016-1.tif]

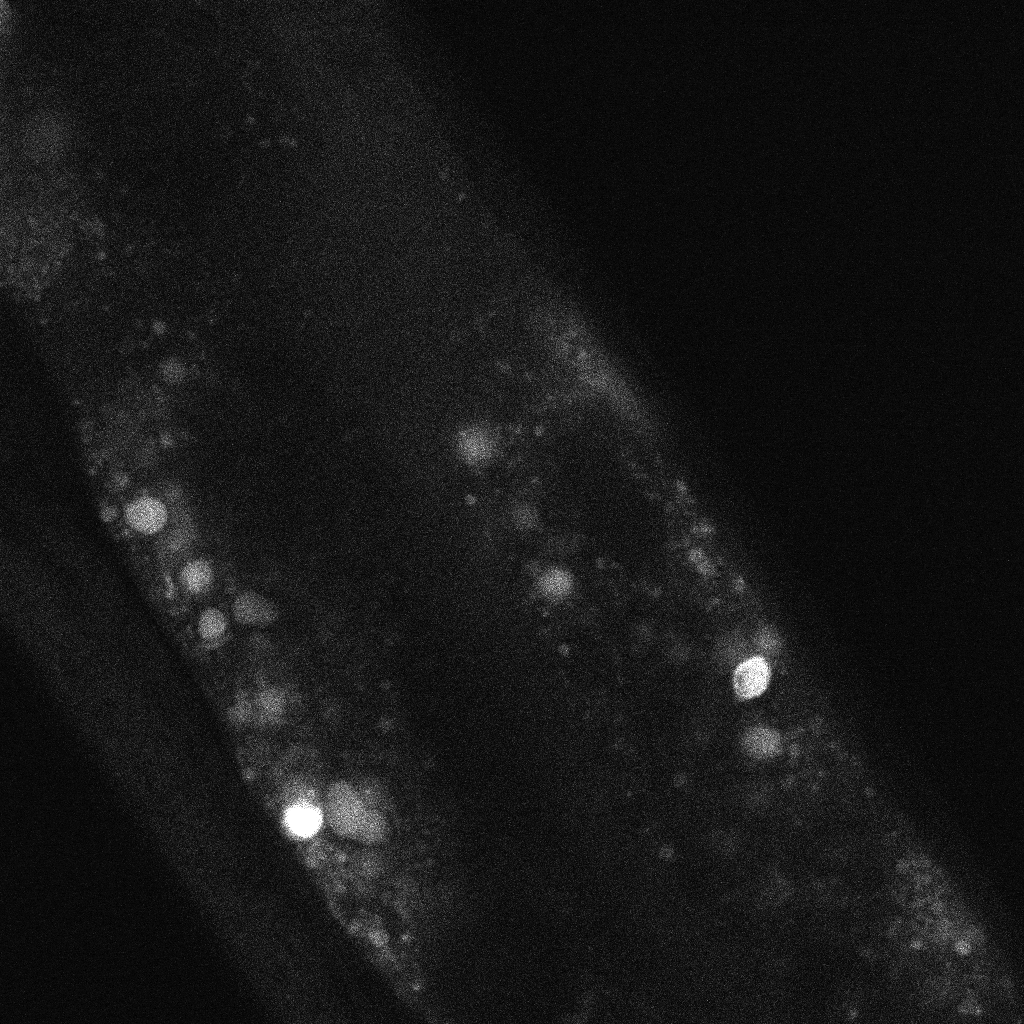

Supplement: Supplementary file 8 — Source data Fig. 6 [file 44318_2025_367_MOESM8_ESM.zip › SD figure 6/6A/Fig_6_A_data/ubq-1+vps-39 (RNAi)/A GFPRab5 mCherrzRab7 sand1 ok1963 ubq1 and vps39 RNAi 1 to 250 front_0010-1.tif]

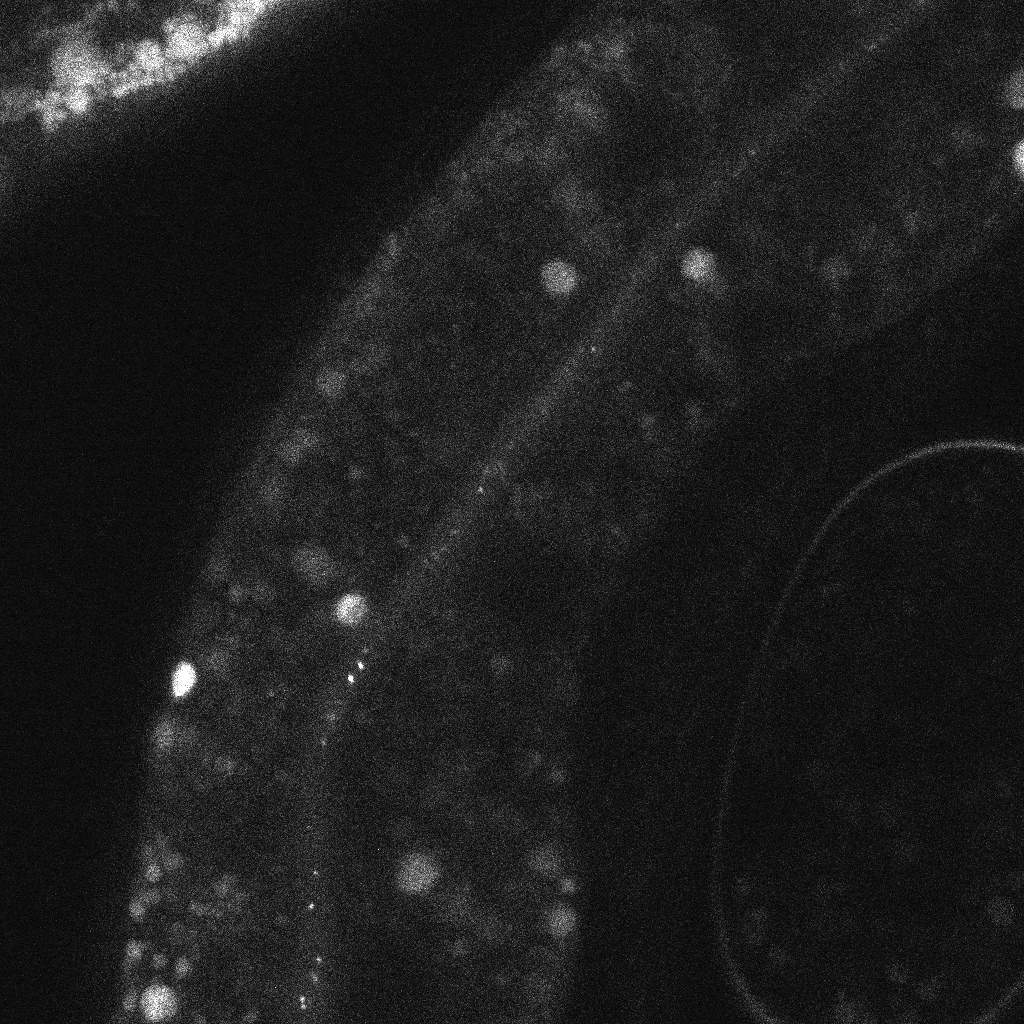

Supplement: Supplementary file 8 — Source data Fig. 6 [file 44318_2025_367_MOESM8_ESM.zip › SD figure 6/6A/Fig_6_A_data/vps-39 (RNAi)/A GFPRab5 mCherrzRab7 sand1 ok1963 vps39 RNAi front_0003-1.tif]

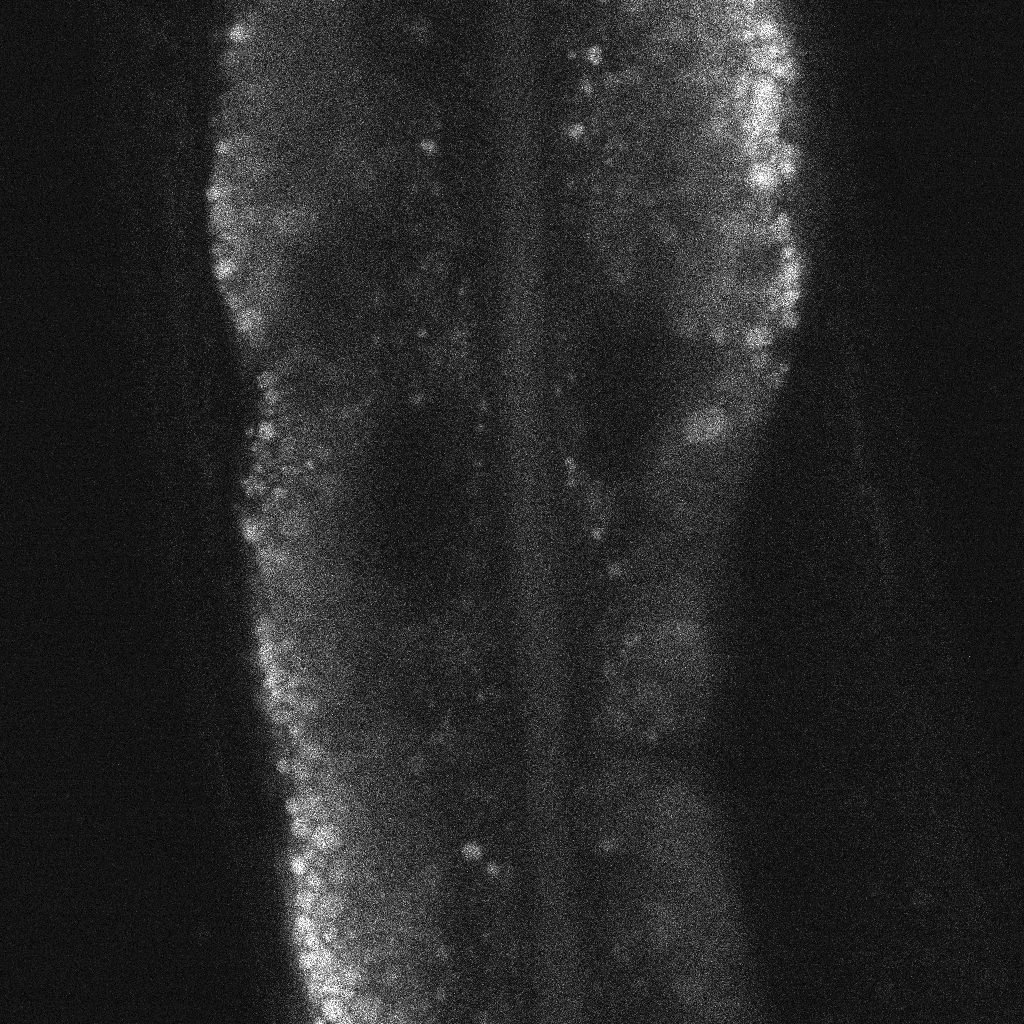

Supplement: Supplementary file 8 — Source data Fig. 6 [file 44318_2025_367_MOESM8_ESM.zip › SD figure 6/6C/Fig_6_C_data/Mock/A sand1 ok1963 lmp1GFP RAB7mCherrz control RNAi front_0013-1.tif]

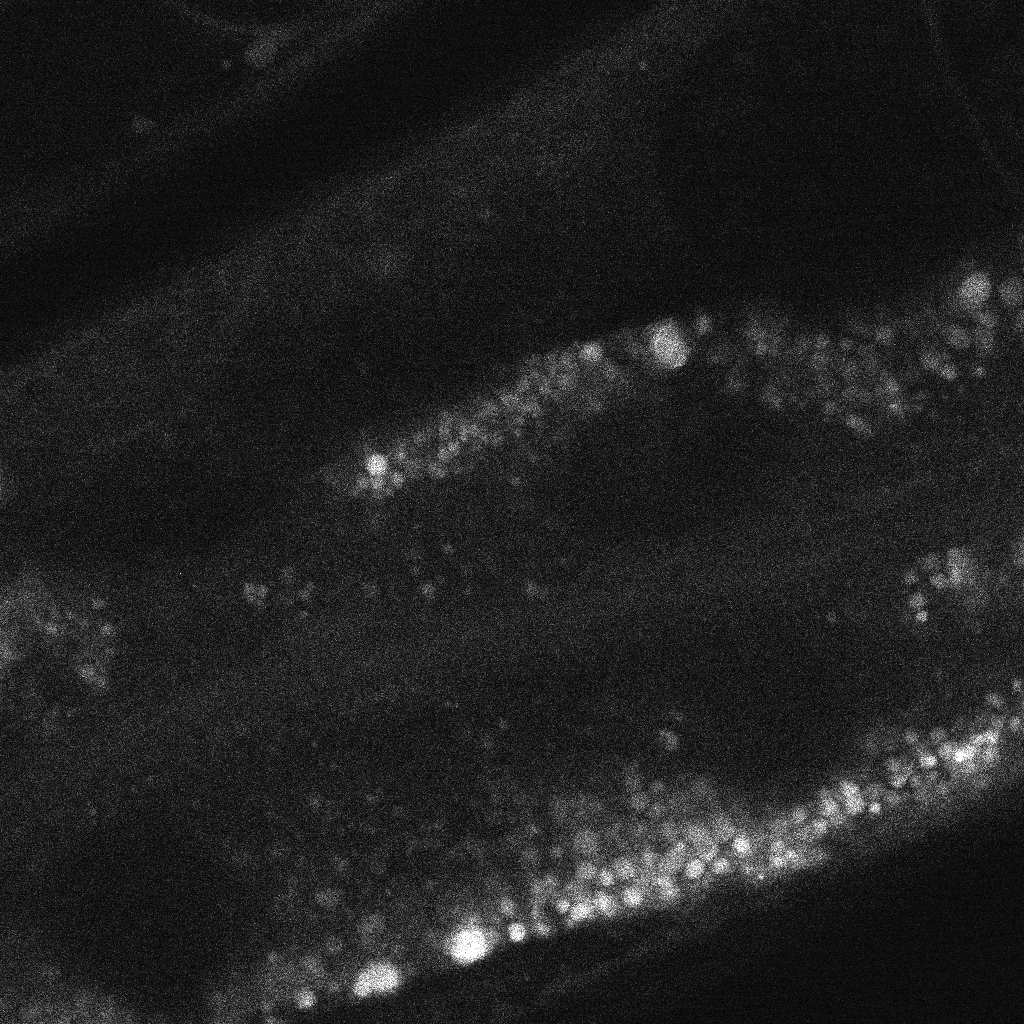

Supplement: Supplementary file 8 — Source data Fig. 6 [file 44318_2025_367_MOESM8_ESM.zip › SD figure 6/6C/Fig_6_C_data/ubq-1+vps-39 (RNAi)/A sand1 ok1963 lmp1GFP rab7mCherrz ubq1 and vps39 RNAi 1 to 250 front_0008-1.tif]

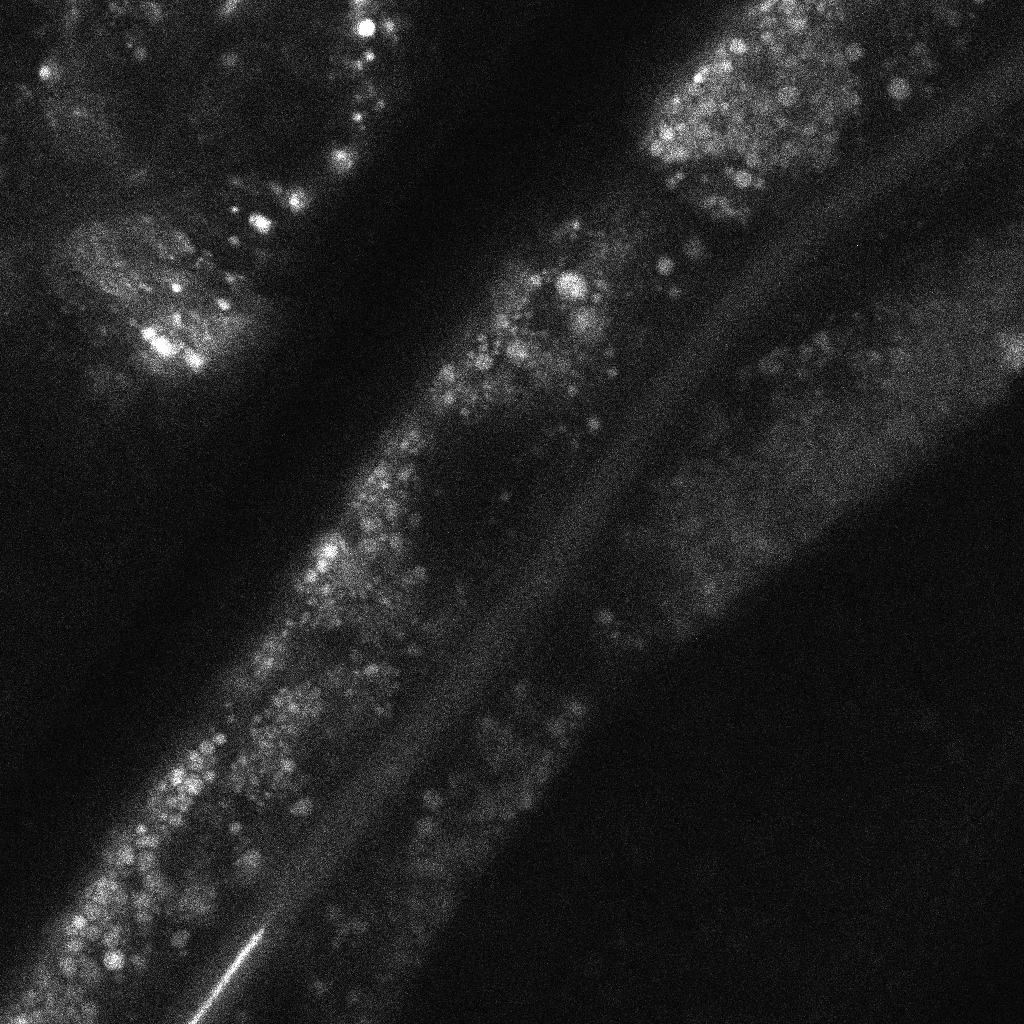

Supplement: Supplementary file 8 — Source data Fig. 6 [file 44318_2025_367_MOESM8_ESM.zip › SD figure 6/6C/Fig_6_C_data/vps-39 (RNAi)/A sand1 ok1963 lmp1GFP RAB7mCherrz vps39 RNAi front_0001-1.tif]

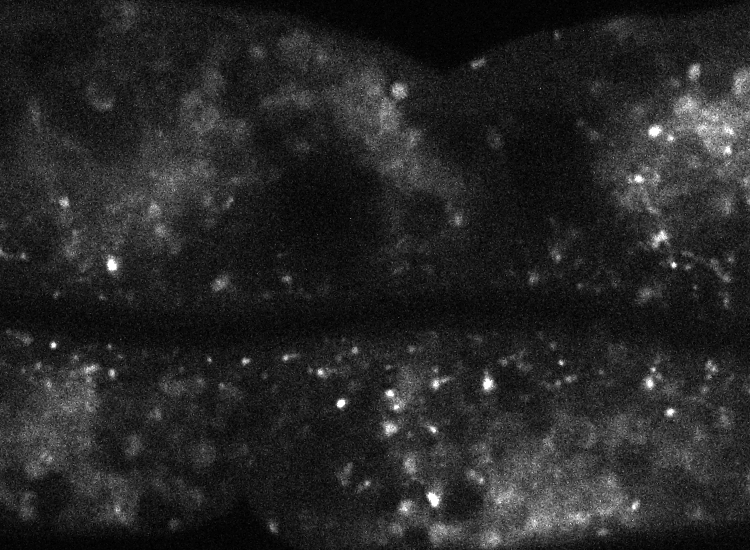

Supplement: Supplementary file 8 — Source data Fig. 6 [file 44318_2025_367_MOESM8_ESM.zip › SD figure 6/6A/Fig_6_A_Roi/Mock/Gut /ART MC GFPRab5 mCherrzRab7 sand1 ok1963 control RNAi front_0016-1-1-1-1.tif]

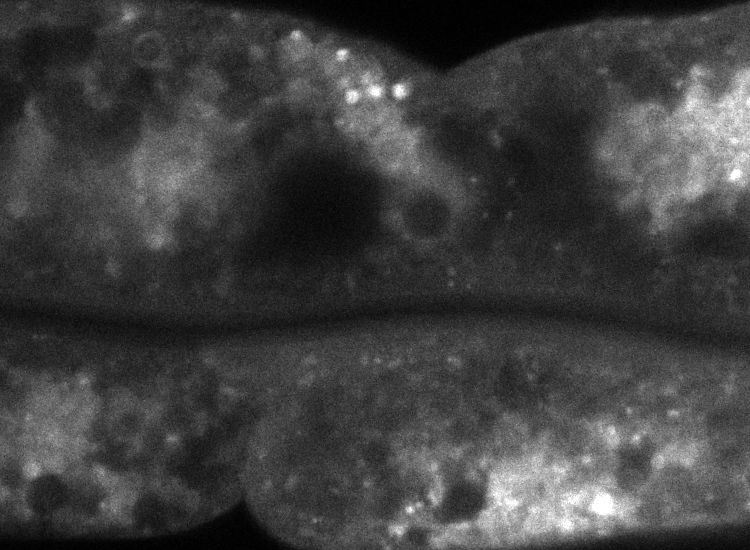

Supplement: Supplementary file 8 — Source data Fig. 6 [file 44318_2025_367_MOESM8_ESM.zip › SD figure 6/6A/Fig_6_A_Roi/Mock/Gut /ART G GFPRab5 mCherrzRab7 sand1 ok1963 control RNAi front_0016-1-1-1-1.tif]

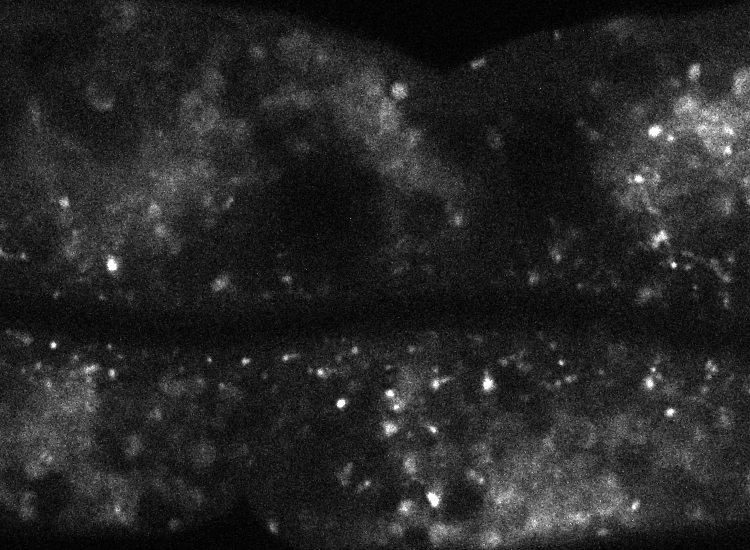

Supplement: Supplementary file 8 — Source data Fig. 6 [file 44318_2025_367_MOESM8_ESM.zip › SD figure 6/6A/Fig_6_A_Roi/Mock/Gut /ART MGM GFPRab5 mCherrzRab7 sand1 ok1963 control RNAi front_0016-1-1-1-1.tif]

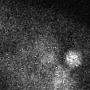

Supplement: Supplementary file 8 — Source data Fig. 6 [file 44318_2025_367_MOESM8_ESM.zip › SD figure 6/6A/Fig_6_A_Roi/Mock/Gut close up/ART C2 MC GFPRab5 mCherrzRab7 sand1 ok1963 control RNAi front_0016-1-1-1-1-1.tif]

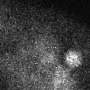

Supplement: Supplementary file 8 — Source data Fig. 6 [file 44318_2025_367_MOESM8_ESM.zip › SD figure 6/6A/Fig_6_A_Roi/Mock/Gut close up/ART C2 MGM GFPRab5 mCherrzRab7 sand1 ok1963 control RNAi front_0016-1-1-1-1-1.tif]

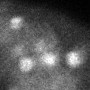

Supplement: Supplementary file 8 — Source data Fig. 6 [file 44318_2025_367_MOESM8_ESM.zip › SD figure 6/6A/Fig_6_A_Roi/Mock/Gut close up/ART C2 G GFPRab5 mCherrzRab7 sand1 ok1963 control RNAi front_0016-1-1-1-1-1.tif]

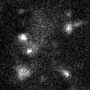

Supplement: Supplementary file 8 — Source data Fig. 6 [file 44318_2025_367_MOESM8_ESM.zip › SD figure 6/6A/Fig_6_A_Roi/Mock/Gut close up/ART C MGM GFPRab5 mCherrzRab7 sand1 ok1963 control RNAi front_0016-1-1-1-1-1.tif]

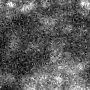

Supplement: Supplementary file 8 — Source data Fig. 6 [file 44318_2025_367_MOESM8_ESM.zip › SD figure 6/6A/Fig_6_A_Roi/Mock/Gut close up/ART C G GFPRab5 mCherrzRab7 sand1 ok1963 control RNAi front_0016-1-1-1-1-1.tif]

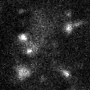

Supplement: Supplementary file 8 — Source data Fig. 6 [file 44318_2025_367_MOESM8_ESM.zip › SD figure 6/6A/Fig_6_A_Roi/Mock/Gut close up/ART C MC GFPRab5 mCherrzRab7 sand1 ok1963 control RNAi front_0016-1-1-1-1-1.tif]

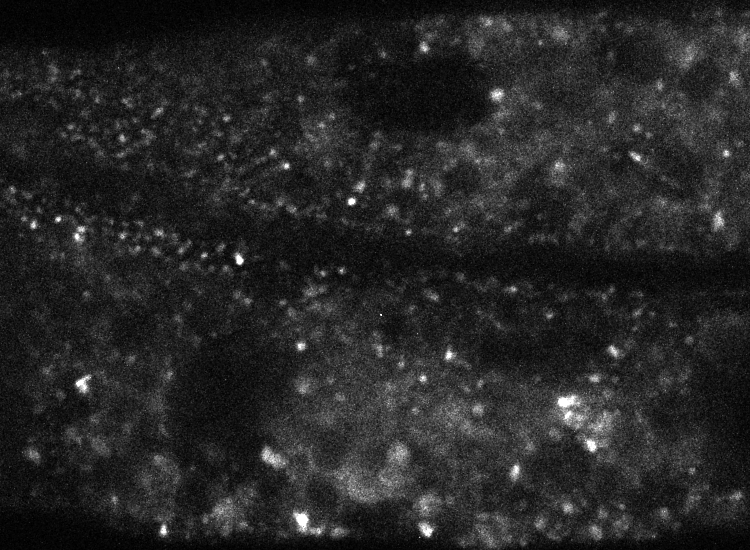

Supplement: Supplementary file 8 — Source data Fig. 6 [file 44318_2025_367_MOESM8_ESM.zip › SD figure 6/6A/Fig_6_A_Roi/ubq-1+vps-39 (RNAi)/Gut /ART MGM GFPRab5 mCherrzRab7 sand1 ok1963 ubq1 and vps39 RNAi 1 to 250 front_0010-1-1-1-1.tif]

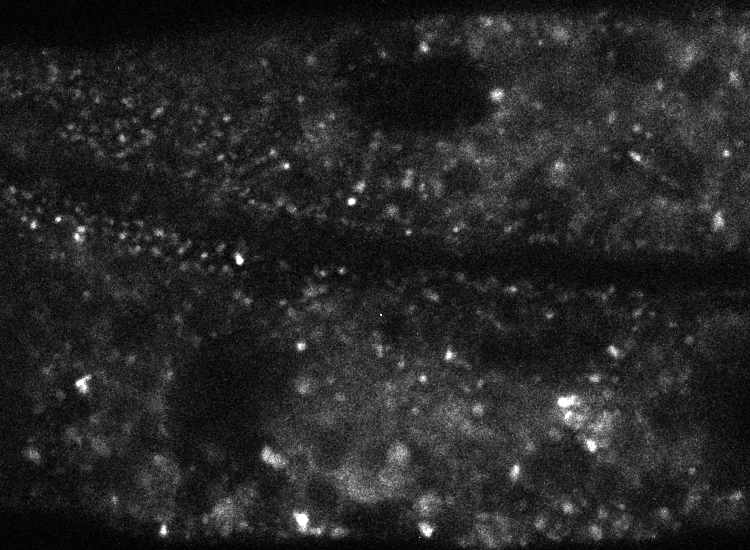

Supplement: Supplementary file 8 — Source data Fig. 6 [file 44318_2025_367_MOESM8_ESM.zip › SD figure 6/6A/Fig_6_A_Roi/ubq-1+vps-39 (RNAi)/Gut /ART MC GFPRab5 mCherrzRab7 sand1 ok1963 ubq1 and vps39 RNAi 1 to 250 front_0010-1-1-1-1.tif]

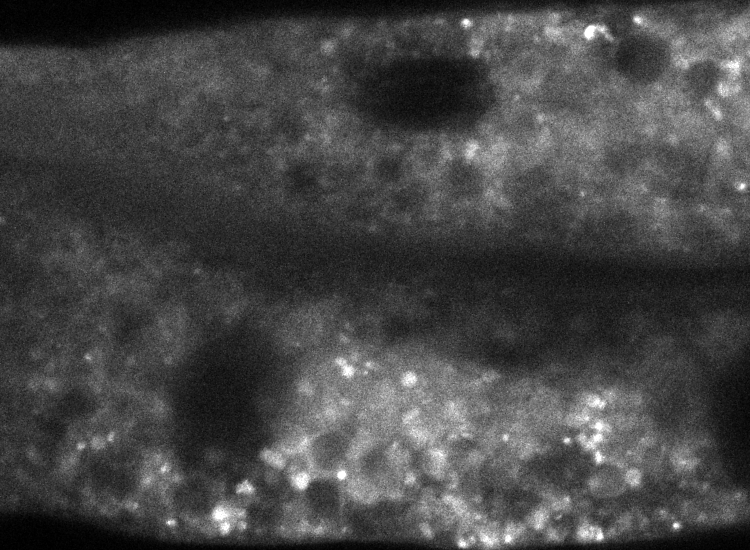

Supplement: Supplementary file 8 — Source data Fig. 6 [file 44318_2025_367_MOESM8_ESM.zip › SD figure 6/6A/Fig_6_A_Roi/ubq-1+vps-39 (RNAi)/Gut /ART G GFPRab5 mCherrzRab7 sand1 ok1963 ubq1 and vps39 RNAi 1 to 250 front_0010-1-1-1-1.tif]

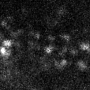

Supplement: Supplementary file 8 — Source data Fig. 6 [file 44318_2025_367_MOESM8_ESM.zip › SD figure 6/6A/Fig_6_A_Roi/ubq-1+vps-39 (RNAi)/Gut close up/ART C2 MGM GFPRab5 mCherrzRab7 sand1 ok1963 ubq1 and vps39 RNAi 1 to 250 front_0010-1-1-1-1-1.tif]

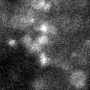

Supplement: Supplementary file 8 — Source data Fig. 6 [file 44318_2025_367_MOESM8_ESM.zip › SD figure 6/6A/Fig_6_A_Roi/ubq-1+vps-39 (RNAi)/Gut close up/ART C G GFPRab5 mCherrzRab7 sand1 ok1963 ubq1 and vps39 RNAi 1 to 250 front_0010-1-1-1-1-1.tif]

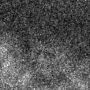

Supplement: Supplementary file 8 — Source data Fig. 6 [file 44318_2025_367_MOESM8_ESM.zip › SD figure 6/6A/Fig_6_A_Roi/ubq-1+vps-39 (RNAi)/Gut close up/ART C2 G GFPRab5 mCherrzRab7 sand1 ok1963 ubq1 and vps39 RNAi 1 to 250 front_0010-1-1-1-1-1.tif]

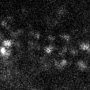

Supplement: Supplementary file 8 — Source data Fig. 6 [file 44318_2025_367_MOESM8_ESM.zip › SD figure 6/6A/Fig_6_A_Roi/ubq-1+vps-39 (RNAi)/Gut close up/ART C2 MC GFPRab5 mCherrzRab7 sand1 ok1963 ubq1 and vps39 RNAi 1 to 250 front_0010-1-1-1-1-1.tif]

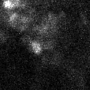

Supplement: Supplementary file 8 — Source data Fig. 6 [file 44318_2025_367_MOESM8_ESM.zip › SD figure 6/6A/Fig_6_A_Roi/ubq-1+vps-39 (RNAi)/Gut close up/ART C MGM GFPRab5 mCherrzRab7 sand1 ok1963 ubq1 and vps39 RNAi 1 to 250 front_0010-1-1-1-1-1.tif]

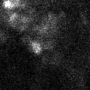

Supplement: Supplementary file 8 — Source data Fig. 6 [file 44318_2025_367_MOESM8_ESM.zip › SD figure 6/6A/Fig_6_A_Roi/ubq-1+vps-39 (RNAi)/Gut close up/ART C MC GFPRab5 mCherrzRab7 sand1 ok1963 ubq1 and vps39 RNAi 1 to 250 front_0010-1-1-1-1-1.tif]

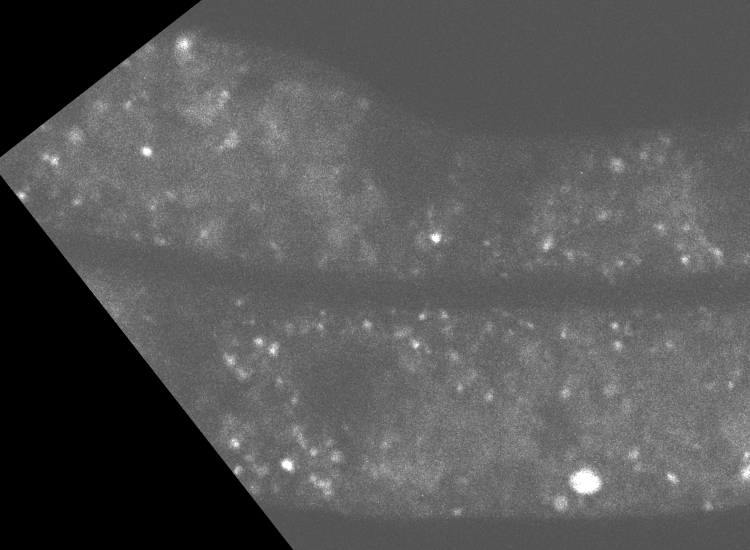

Supplement: Supplementary file 8 — Source data Fig. 6 [file 44318_2025_367_MOESM8_ESM.zip › SD figure 6/6A/Fig_6_A_Roi/vps-39 (RNAi)/Gut /ART MGM GFPRab5 mCherrzRab7 sand1 ok1963 vps39 RNAi front_0003-1-1-1-1.tif]

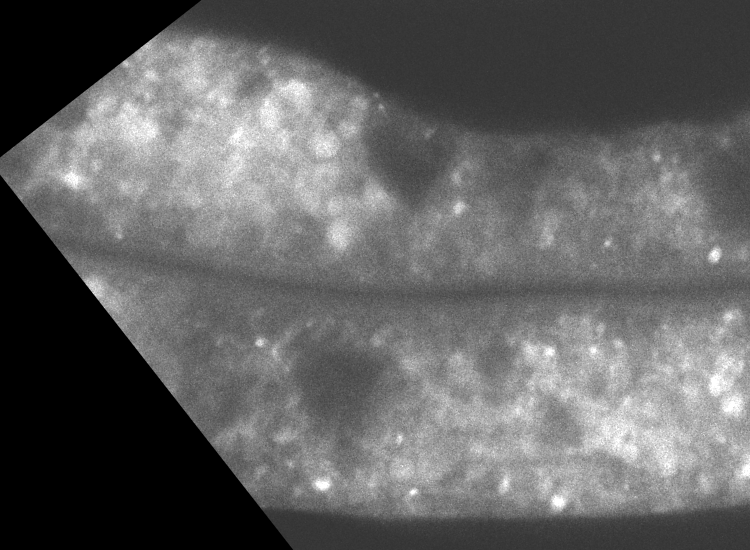

Supplement: Supplementary file 8 — Source data Fig. 6 [file 44318_2025_367_MOESM8_ESM.zip › SD figure 6/6A/Fig_6_A_Roi/vps-39 (RNAi)/Gut /ART G GFPRab5 mCherrzRab7 sand1 ok1963 vps39 RNAi front_0003-1-1-1-1.tif]

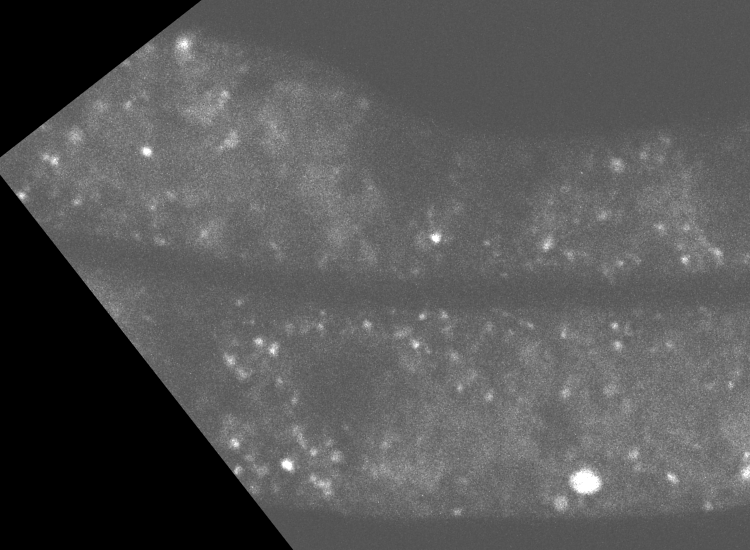

Supplement: Supplementary file 8 — Source data Fig. 6 [file 44318_2025_367_MOESM8_ESM.zip › SD figure 6/6A/Fig_6_A_Roi/vps-39 (RNAi)/Gut /ART MC GFPRab5 mCherrzRab7 sand1 ok1963 vps39 RNAi front_0003-1-1-1-1.tif]

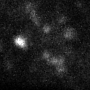

Supplement: Supplementary file 8 — Source data Fig. 6 [file 44318_2025_367_MOESM8_ESM.zip › SD figure 6/6A/Fig_6_A_Roi/vps-39 (RNAi)/Gut close up/ART C MC GFPRab5 mCherrzRab7 sand1 ok1963 vps39 RNAi front_0003-1-1-1-1-1.tif]

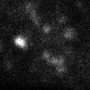

Supplement: Supplementary file 8 — Source data Fig. 6 [file 44318_2025_367_MOESM8_ESM.zip › SD figure 6/6A/Fig_6_A_Roi/vps-39 (RNAi)/Gut close up/ART C MGM GFPRab5 mCherrzRab7 sand1 ok1963 vps39 RNAi front_0003-1-1-1-1-1.tif]

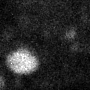

Supplement: Supplementary file 8 — Source data Fig. 6 [file 44318_2025_367_MOESM8_ESM.zip › SD figure 6/6A/Fig_6_A_Roi/vps-39 (RNAi)/Gut close up/ART C2 MC GFPRab5 mCherrzRab7 sand1 ok1963 vps39 RNAi front_0003-1-1-1-1-1.tif]

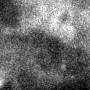

Supplement: Supplementary file 8 — Source data Fig. 6 [file 44318_2025_367_MOESM8_ESM.zip › SD figure 6/6A/Fig_6_A_Roi/vps-39 (RNAi)/Gut close up/ART C2 G GFPRab5 mCherrzRab7 sand1 ok1963 vps39 RNAi front_0003-1-1-1-1-1.tif]

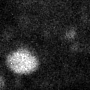

Supplement: Supplementary file 8 — Source data Fig. 6 [file 44318_2025_367_MOESM8_ESM.zip › SD figure 6/6A/Fig_6_A_Roi/vps-39 (RNAi)/Gut close up/ART C2 MGM GFPRab5 mCherrzRab7 sand1 ok1963 vps39 RNAi front_0003-1-1-1-1-1.tif]

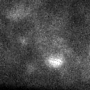

Supplement: Supplementary file 8 — Source data Fig. 6 [file 44318_2025_367_MOESM8_ESM.zip › SD figure 6/6A/Fig_6_A_Roi/vps-39 (RNAi)/Gut close up/ART C G GFPRab5 mCherrzRab7 sand1 ok1963 vps39 RNAi front_0003-1-1-1-1-1.tif]

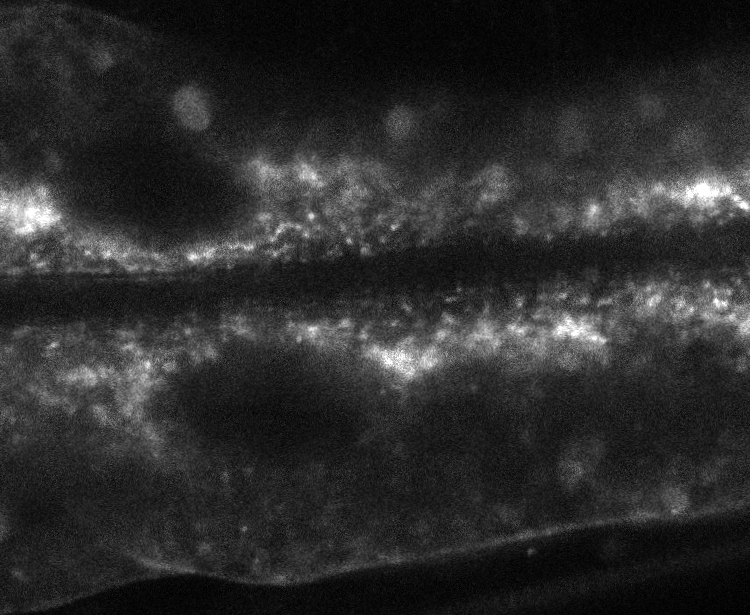

Supplement: Supplementary file 8 — Source data Fig. 6 [file 44318_2025_367_MOESM8_ESM.zip › SD figure 6/6C/Fig_6_C_Roi/Mock/Gut /ART G sand1 ok1963 lmp1GFP RAB7mCherrz control RNAi front_0013-1-1-1-1.tif]

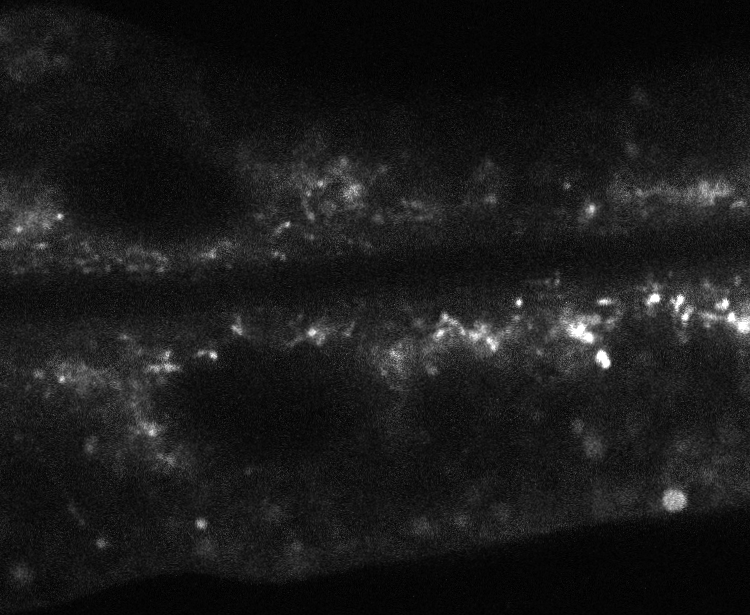

Supplement: Supplementary file 8 — Source data Fig. 6 [file 44318_2025_367_MOESM8_ESM.zip › SD figure 6/6C/Fig_6_C_Roi/Mock/Gut /ART MC sand1 ok1963 lmp1GFP RAB7mCherrz control RNAi front_0013-1-1-1-1.tif]

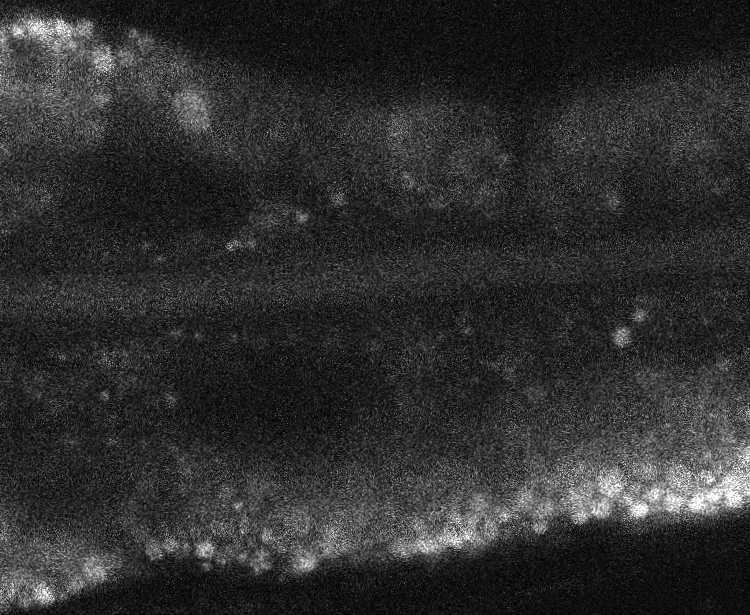

Supplement: Supplementary file 8 — Source data Fig. 6 [file 44318_2025_367_MOESM8_ESM.zip › SD figure 6/6C/Fig_6_C_Roi/Mock/Gut /ART AF sand1 ok1963 lmp1GFP RAB7mCherrz control RNAi front_0013-1-1-1-1.tif]

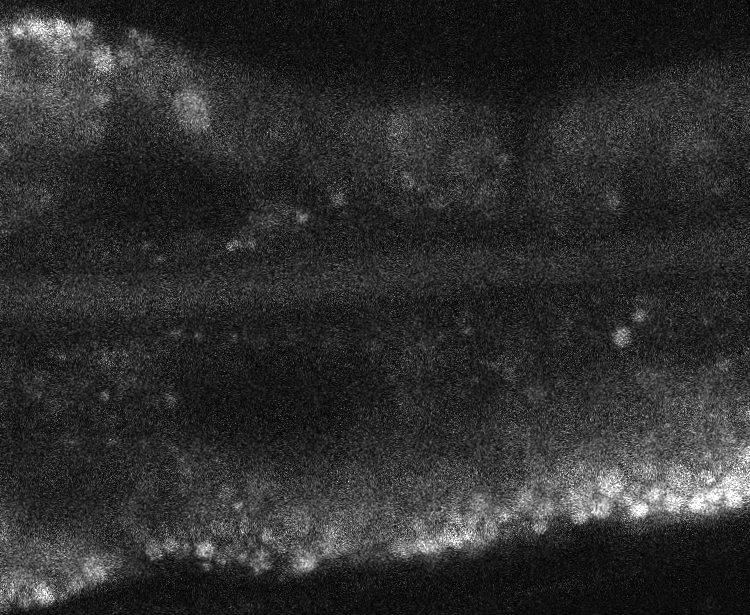

Supplement: Supplementary file 8 — Source data Fig. 6 [file 44318_2025_367_MOESM8_ESM.zip › SD figure 6/6C/Fig_6_C_Roi/Mock/Gut /ART MA sand1 ok1963 lmp1GFP RAB7mCherrz control RNAi front_0013-1-1-1.tif]

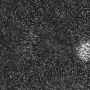

Supplement: Supplementary file 8 — Source data Fig. 6 [file 44318_2025_367_MOESM8_ESM.zip › SD figure 6/6C/Fig_6_C_Roi/Mock/Gut close up/ART C AF sand1 ok1963 lmp1GFP RAB7mCherrz control RNAi front_0013-1-1-1-1-1.tif]

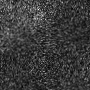

Supplement: Supplementary file 8 — Source data Fig. 6 [file 44318_2025_367_MOESM8_ESM.zip › SD figure 6/6C/Fig_6_C_Roi/Mock/Gut close up/ART C2 AF sand1 ok1963 lmp1GFP RAB7mCherrz control RNAi front_0013-1-1-1-1-1.tif]

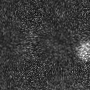

Supplement: Supplementary file 8 — Source data Fig. 6 [file 44318_2025_367_MOESM8_ESM.zip › SD figure 6/6C/Fig_6_C_Roi/Mock/Gut close up/ART C MA sand1 ok1963 lmp1GFP RAB7mCherrz control RNAi front_0013-1-1-1-1.tif]

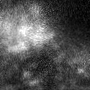

Supplement: Supplementary file 8 — Source data Fig. 6 [file 44318_2025_367_MOESM8_ESM.zip › SD figure 6/6C/Fig_6_C_Roi/Mock/Gut close up/ART C2 G sand1 ok1963 lmp1GFP RAB7mCherrz control RNAi front_0013-1-1-1-1-1.tif]

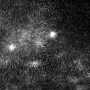

Supplement: Supplementary file 8 — Source data Fig. 6 [file 44318_2025_367_MOESM8_ESM.zip › SD figure 6/6C/Fig_6_C_Roi/Mock/Gut close up/ART C2 MC sand1 ok1963 lmp1GFP RAB7mCherrz control RNAi front_0013-1-1-1-1-1.tif]

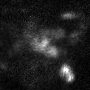

Supplement: Supplementary file 8 — Source data Fig. 6 [file 44318_2025_367_MOESM8_ESM.zip › SD figure 6/6C/Fig_6_C_Roi/Mock/Gut close up/ART C MC sand1 ok1963 lmp1GFP RAB7mCherrz control RNAi front_0013-1-1-1-1-1.tif]

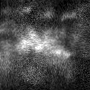

Supplement: Supplementary file 8 — Source data Fig. 6 [file 44318_2025_367_MOESM8_ESM.zip › SD figure 6/6C/Fig_6_C_Roi/Mock/Gut close up/ART C G sand1 ok1963 lmp1GFP RAB7mCherrz control RNAi front_0013-1-1-1-1-1.tif]

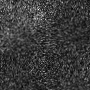

Supplement: Supplementary file 8 — Source data Fig. 6 [file 44318_2025_367_MOESM8_ESM.zip › SD figure 6/6C/Fig_6_C_Roi/Mock/Gut close up/ART C2 MA sand1 ok1963 lmp1GFP RAB7mCherrz control RNAi front_0013-1-1-1-1.tif]

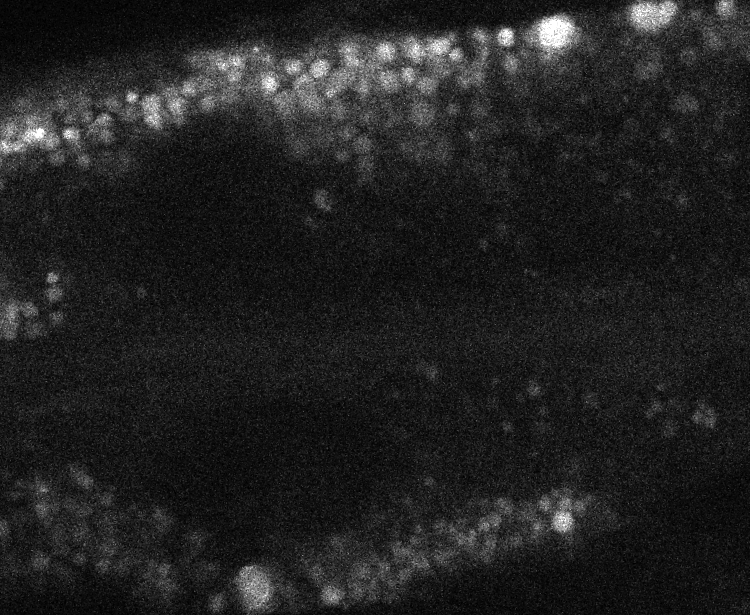

Supplement: Supplementary file 8 — Source data Fig. 6 [file 44318_2025_367_MOESM8_ESM.zip › SD figure 6/6C/Fig_6_C_Roi/ubq-1+vps-39 (RNAi)/Gut /ART AF sand1 ok1963 lmp1GFP rab7mCherrz ubq1 and vps39 RNAi 1 to 250 front_0008-1-1-1-1.tif]

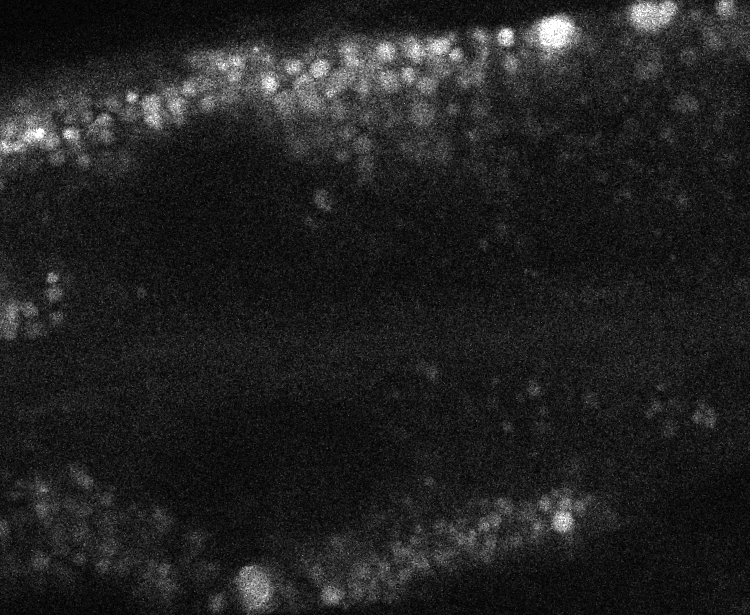

Supplement: Supplementary file 8 — Source data Fig. 6 [file 44318_2025_367_MOESM8_ESM.zip › SD figure 6/6C/Fig_6_C_Roi/ubq-1+vps-39 (RNAi)/Gut /ART MA sand1 ok1963 lmp1GFP rab7mCherrz ubq1 and vps39 RNAi 1 to 250 front_0008-1-1-1.tif]

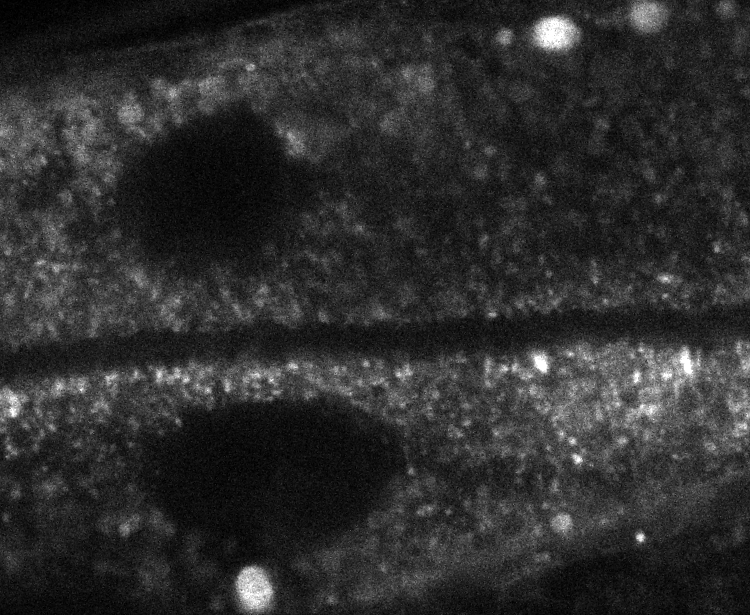

Supplement: Supplementary file 8 — Source data Fig. 6 [file 44318_2025_367_MOESM8_ESM.zip › SD figure 6/6C/Fig_6_C_Roi/ubq-1+vps-39 (RNAi)/Gut /ART G sand1 ok1963 lmp1GFP rab7mCherrz ubq1 and vps39 RNAi 1 to 250 front_0008-1-1-1-1.tif]

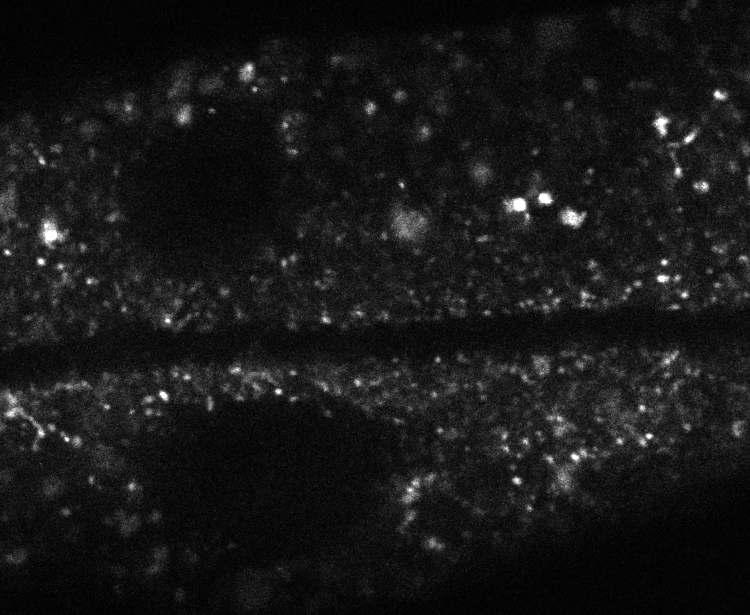

Supplement: Supplementary file 8 — Source data Fig. 6 [file 44318_2025_367_MOESM8_ESM.zip › SD figure 6/6C/Fig_6_C_Roi/ubq-1+vps-39 (RNAi)/Gut /ART MC sand1 ok1963 lmp1GFP rab7mCherrz ubq1 and vps39 RNAi 1 to 250 front_0008-1-1-1-1.tif]

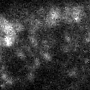

Supplement: Supplementary file 8 — Source data Fig. 6 [file 44318_2025_367_MOESM8_ESM.zip › SD figure 6/6C/Fig_6_C_Roi/ubq-1+vps-39 (RNAi)/Gut close up/ART C2 G sand1 ok1963 lmp1GFP rab7mCherrz ubq1 and vps39 RNAi 1 to 250 front_0008-1-1-1-1-1.tif]

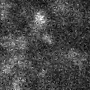

Supplement: Supplementary file 8 — Source data Fig. 6 [file 44318_2025_367_MOESM8_ESM.zip › SD figure 6/6C/Fig_6_C_Roi/ubq-1+vps-39 (RNAi)/Gut close up/ART C G sand1 ok1963 lmp1GFP rab7mCherrz ubq1 and vps39 RNAi 1 to 250 front_0008-1-1-1-1-1.tif]

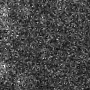

Supplement: Supplementary file 8 — Source data Fig. 6 [file 44318_2025_367_MOESM8_ESM.zip › SD figure 6/6C/Fig_6_C_Roi/ubq-1+vps-39 (RNAi)/Gut close up/ART C AF sand1 ok1963 lmp1GFP rab7mCherrz ubq1 and vps39 RNAi 1 to 250 front_0008-1-1-1-1-1.tif]

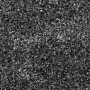

Supplement: Supplementary file 8 — Source data Fig. 6 [file 44318_2025_367_MOESM8_ESM.zip › SD figure 6/6C/Fig_6_C_Roi/ubq-1+vps-39 (RNAi)/Gut close up/ART C2 AF sand1 ok1963 lmp1GFP rab7mCherrz ubq1 and vps39 RNAi 1 to 250 front_0008-1-1-1-1-1.tif]

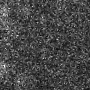

Supplement: Supplementary file 8 — Source data Fig. 6 [file 44318_2025_367_MOESM8_ESM.zip › SD figure 6/6C/Fig_6_C_Roi/ubq-1+vps-39 (RNAi)/Gut close up/ART C MA sand1 ok1963 lmp1GFP rab7mCherrz ubq1 and vps39 RNAi 1 to 250 front_0008-1-1-1-1.tif]

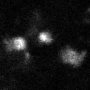

Supplement: Supplementary file 8 — Source data Fig. 6 [file 44318_2025_367_MOESM8_ESM.zip › SD figure 6/6C/Fig_6_C_Roi/ubq-1+vps-39 (RNAi)/Gut close up/ART C MC sand1 ok1963 lmp1GFP rab7mCherrz ubq1 and vps39 RNAi 1 to 250 front_0008-1-1-1-1-1.tif]
