# Supplementary figures and images for: Coordination between ESCRT function and Rab conversion during endosome maturation (part 5 of 9)
Source: EMBO J. 2025 Feb 5;44(6):1574–607. doi: 10.1038/s44318-025-00367-7 (PMC11914609; doi:10.1038/s44318-025-00367-7)

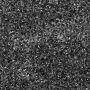

Supplement: Supplementary file 8 — Source data Fig. 6 [file 44318_2025_367_MOESM8_ESM.zip › SD figure 6/6C/Fig_6_C_Roi/ubq-1+vps-39 (RNAi)/Gut close up/ART C2 MA sand1 ok1963 lmp1GFP rab7mCherrz ubq1 and vps39 RNAi 1 to 250 front_0008-1-1-1-1.tif]

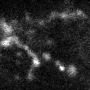

Supplement: Supplementary file 8 — Source data Fig. 6 [file 44318_2025_367_MOESM8_ESM.zip › SD figure 6/6C/Fig_6_C_Roi/ubq-1+vps-39 (RNAi)/Gut close up/ART C2 MC sand1 ok1963 lmp1GFP rab7mCherrz ubq1 and vps39 RNAi 1 to 250 front_0008-1-1-1-1-1.tif]

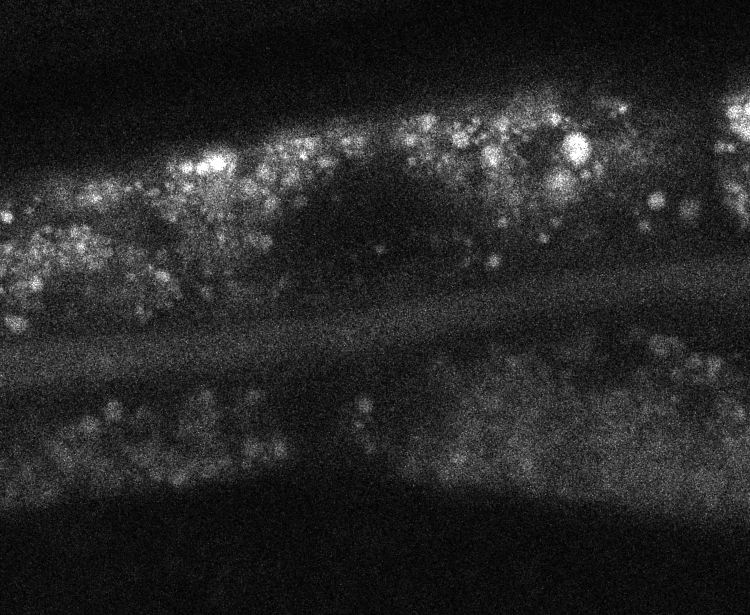

Supplement: Supplementary file 8 — Source data Fig. 6 [file 44318_2025_367_MOESM8_ESM.zip › SD figure 6/6C/Fig_6_C_Roi/vps-39 (RNAi)/Gut /ART AF sand1 ok1963 lmp1GFP RAB7mCherrz vps39 RNAi front_0001-1-1-1-1.tif]

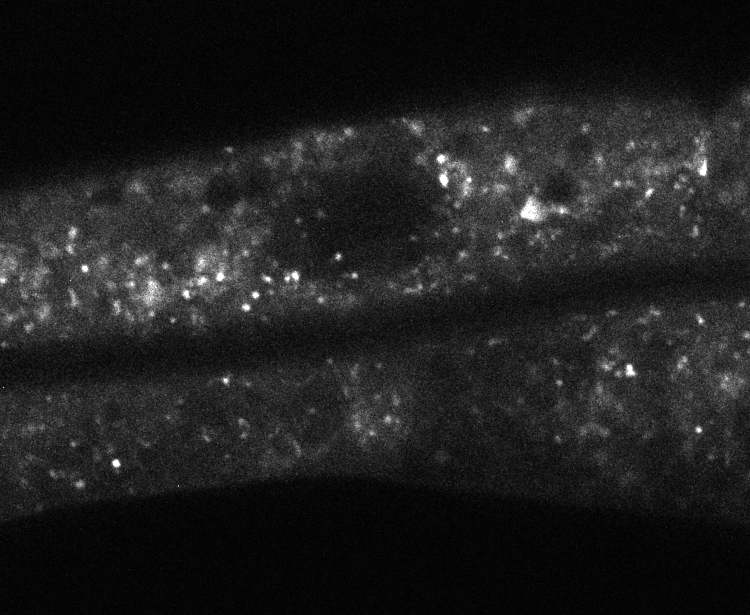

Supplement: Supplementary file 8 — Source data Fig. 6 [file 44318_2025_367_MOESM8_ESM.zip › SD figure 6/6C/Fig_6_C_Roi/vps-39 (RNAi)/Gut /ART MC sand1 ok1963 lmp1GFP RAB7mCherrz vps39 RNAi front_0001-1-1-1-1.tif]

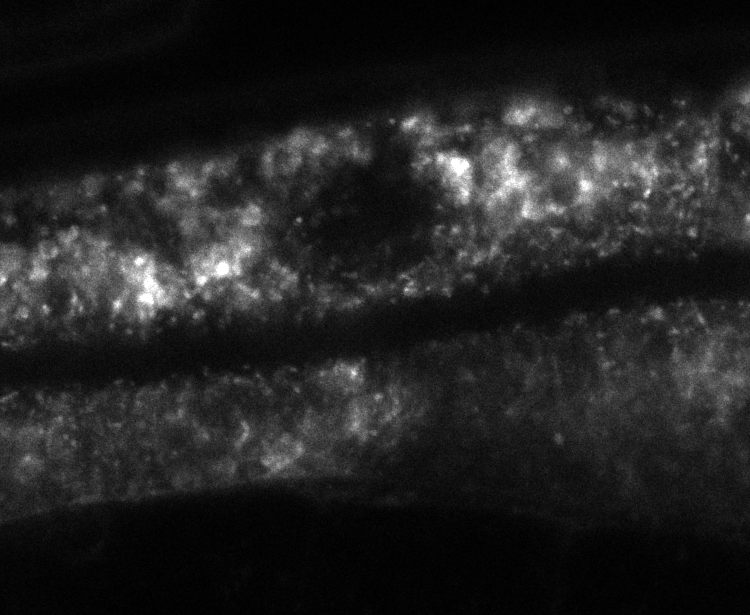

Supplement: Supplementary file 8 — Source data Fig. 6 [file 44318_2025_367_MOESM8_ESM.zip › SD figure 6/6C/Fig_6_C_Roi/vps-39 (RNAi)/Gut /ART G sand1 ok1963 lmp1GFP RAB7mCherrz vps39 RNAi front_0001-1-1-1-1.tif]

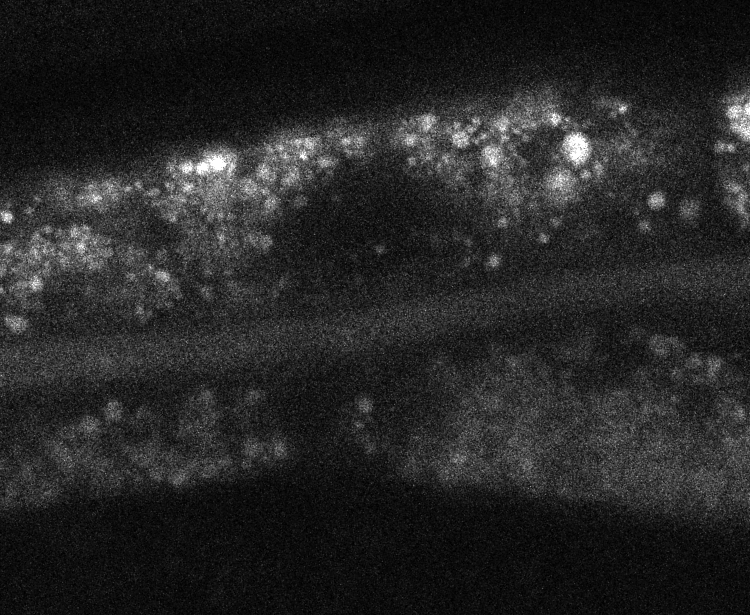

Supplement: Supplementary file 8 — Source data Fig. 6 [file 44318_2025_367_MOESM8_ESM.zip › SD figure 6/6C/Fig_6_C_Roi/vps-39 (RNAi)/Gut /ART MA sand1 ok1963 lmp1GFP RAB7mCherrz vps39 RNAi front_0001-1-1-1.tif]

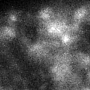

Supplement: Supplementary file 8 — Source data Fig. 6 [file 44318_2025_367_MOESM8_ESM.zip › SD figure 6/6C/Fig_6_C_Roi/vps-39 (RNAi)/Gut close up/ART C2 G sand1 ok1963 lmp1GFP RAB7mCherrz vps39 RNAi front_0001-1-1-1-1-1.tif]

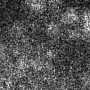

Supplement: Supplementary file 8 — Source data Fig. 6 [file 44318_2025_367_MOESM8_ESM.zip › SD figure 6/6C/Fig_6_C_Roi/vps-39 (RNAi)/Gut close up/ART C AF sand1 ok1963 lmp1GFP RAB7mCherrz vps39 RNAi front_0001-1-1-1-1-1.tif]

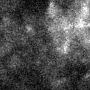

Supplement: Supplementary file 8 — Source data Fig. 6 [file 44318_2025_367_MOESM8_ESM.zip › SD figure 6/6C/Fig_6_C_Roi/vps-39 (RNAi)/Gut close up/ART C G sand1 ok1963 lmp1GFP RAB7mCherrz vps39 RNAi front_0001-1-1-1-1-1.tif]

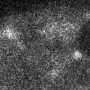

Supplement: Supplementary file 8 — Source data Fig. 6 [file 44318_2025_367_MOESM8_ESM.zip › SD figure 6/6C/Fig_6_C_Roi/vps-39 (RNAi)/Gut close up/ART C2 MC sand1 ok1963 lmp1GFP RAB7mCherrz vps39 RNAi front_0001-1-1-1-1-1.tif]

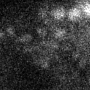

Supplement: Supplementary file 8 — Source data Fig. 6 [file 44318_2025_367_MOESM8_ESM.zip › SD figure 6/6C/Fig_6_C_Roi/vps-39 (RNAi)/Gut close up/ART C2 MA sand1 ok1963 lmp1GFP RAB7mCherrz vps39 RNAi front_0001-1-1-1-1.tif]

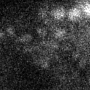

Supplement: Supplementary file 8 — Source data Fig. 6 [file 44318_2025_367_MOESM8_ESM.zip › SD figure 6/6C/Fig_6_C_Roi/vps-39 (RNAi)/Gut close up/ART C2 AF sand1 ok1963 lmp1GFP RAB7mCherrz vps39 RNAi front_0001-1-1-1-1-1.tif]

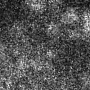

Supplement: Supplementary file 8 — Source data Fig. 6 [file 44318_2025_367_MOESM8_ESM.zip › SD figure 6/6C/Fig_6_C_Roi/vps-39 (RNAi)/Gut close up/ART C MA sand1 ok1963 lmp1GFP RAB7mCherrz vps39 RNAi front_0001-1-1-1-1.tif]

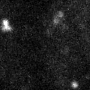

Supplement: Supplementary file 8 — Source data Fig. 6 [file 44318_2025_367_MOESM8_ESM.zip › SD figure 6/6C/Fig_6_C_Roi/vps-39 (RNAi)/Gut close up/ART C MC sand1 ok1963 lmp1GFP RAB7mCherrz vps39 RNAi front_0001-1-1-1-1-1.tif]

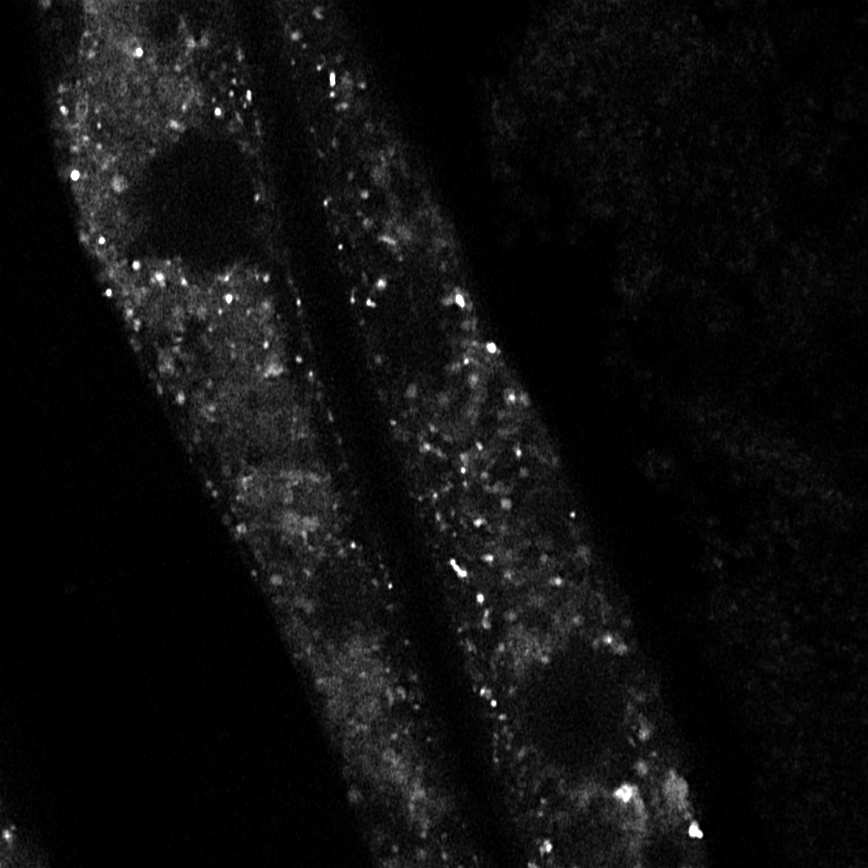

Supplement: Supplementary file 9 — Source data Fig. 7 [file 44318_2025_367_MOESM9_ESM.zip › SD figure 7 /7B/Fig_7_B_data/Mock/A 2023_03_02_RAB-5_RAB-7_sand-1_08_Airyscan Processing-1.tif]

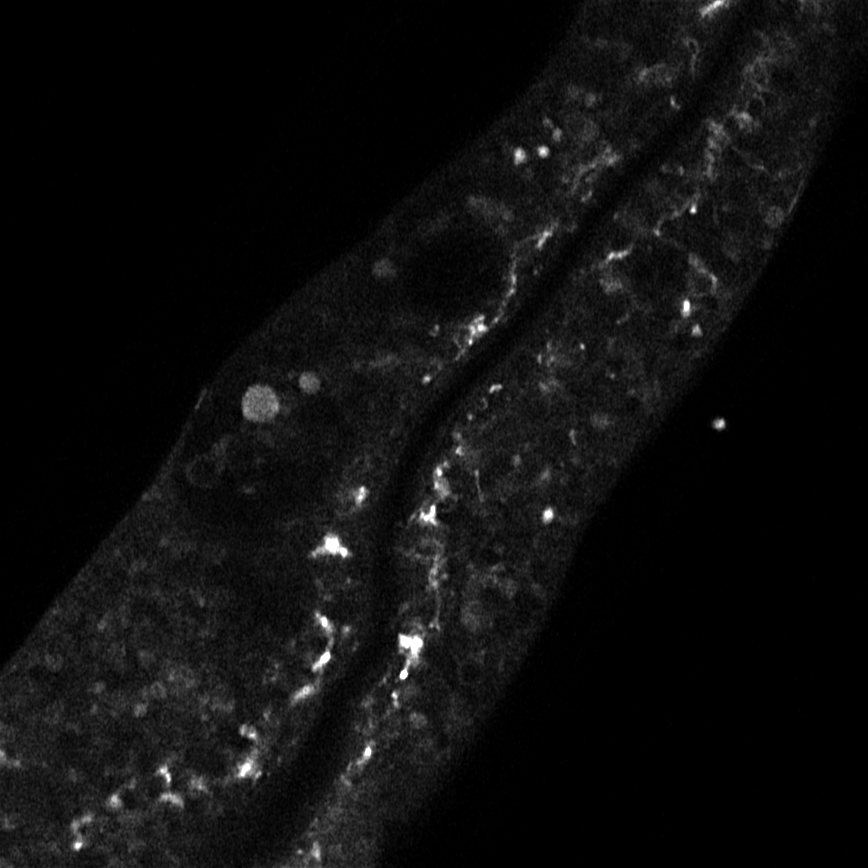

Supplement: Supplementary file 9 — Source data Fig. 7 [file 44318_2025_367_MOESM9_ESM.zip › SD figure 7 /7B/Fig_7_B_data/rabx-5 (RNAi)/A 2023_03_02_RAB-5_RAB-7_sand-1_rabx-5cloned_02_Airyscan Processing-1.tif]

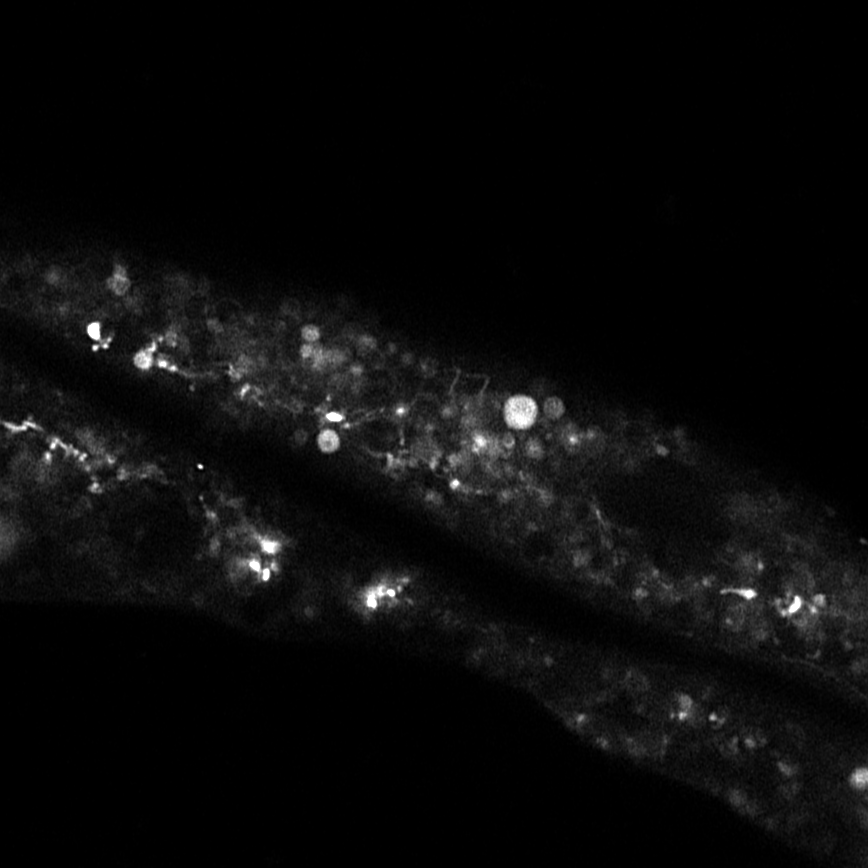

Supplement: Supplementary file 9 — Source data Fig. 7 [file 44318_2025_367_MOESM9_ESM.zip › SD figure 7 /7F/Fig_7_F_data/ubq-1 (RNAi)/2024_08_05_RABX-5_RAB-5_ubq-1_05_Airyscan Processing-1.tif]

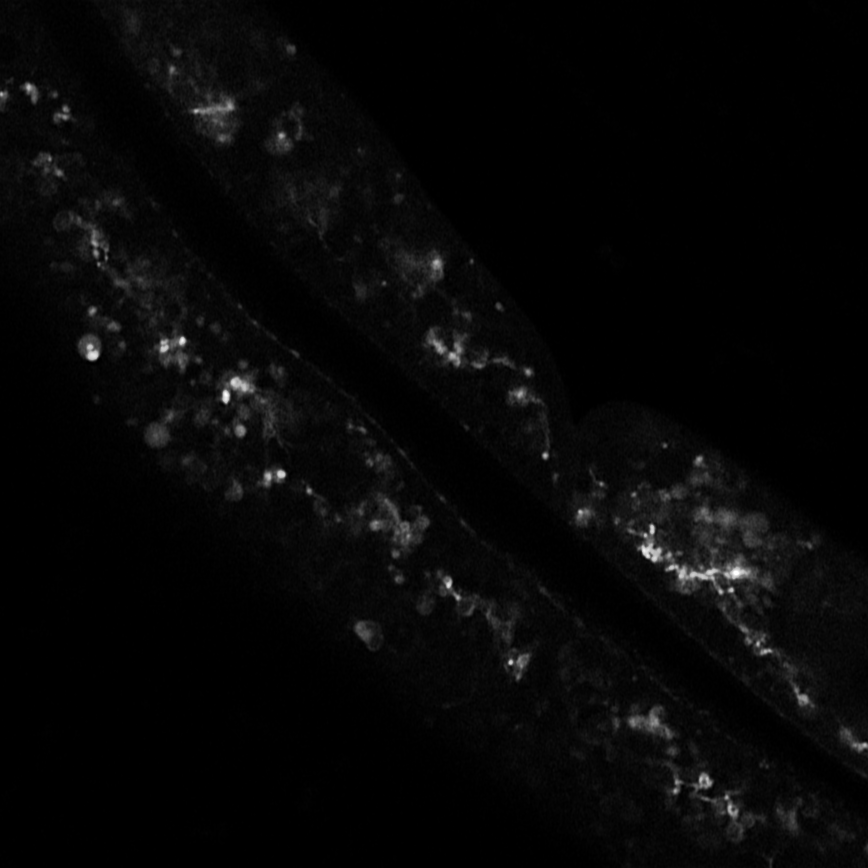

Supplement: Supplementary file 9 — Source data Fig. 7 [file 44318_2025_367_MOESM9_ESM.zip › SD figure 7 /7F/Fig_7_F_data/Mock/2024_08_05_RABX-5_RAB-5_neg_06_Airyscan Processing-1.tif]

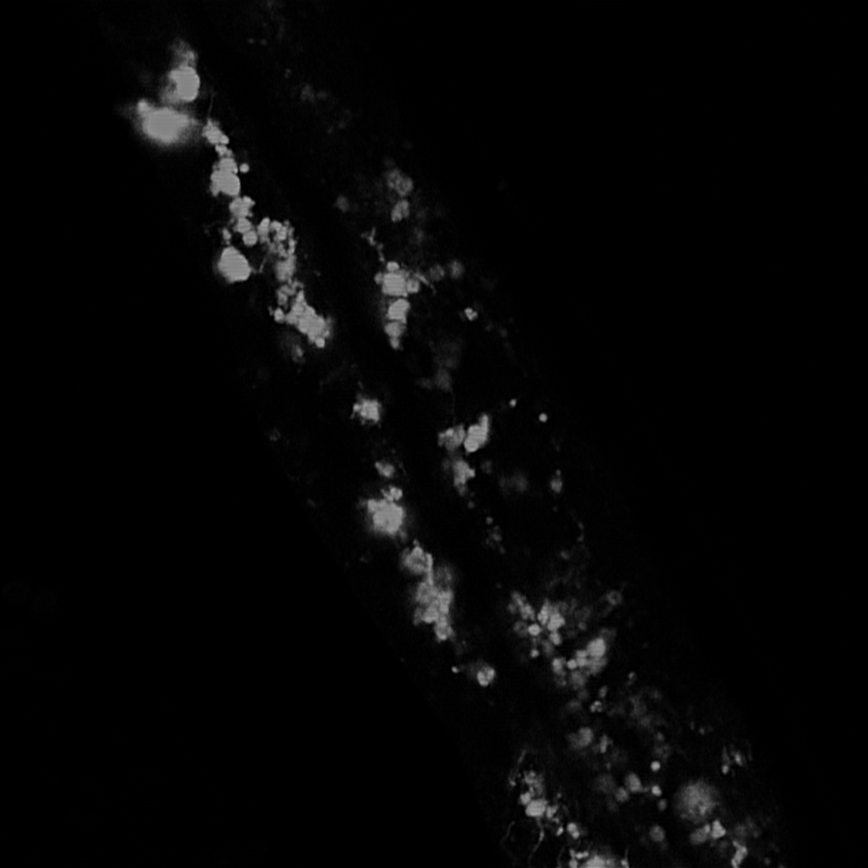

Supplement: Supplementary file 9 — Source data Fig. 7 [file 44318_2025_367_MOESM9_ESM.zip › SD figure 7 /7F/Fig_7_F_data/hgrs-1 (RNAi)/2024_08_06_RABX-5_RAB-5_hgrs-1_06_Airyscan Processing-1.tif]

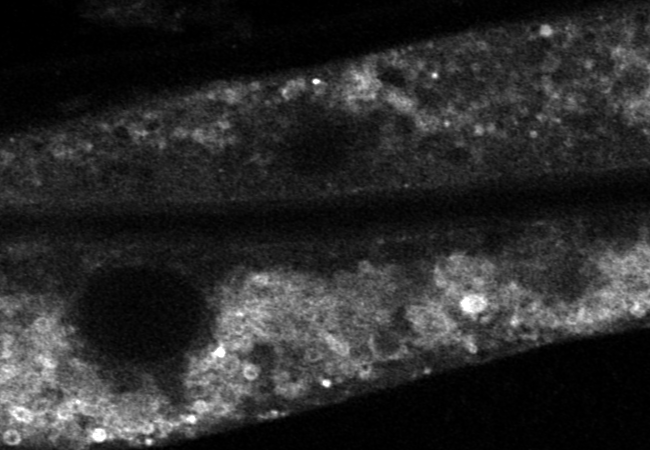

Supplement: Supplementary file 9 — Source data Fig. 7 [file 44318_2025_367_MOESM9_ESM.zip › SD figure 7 /7B/Fig_7_B_Roi/Mock/Gut /ART G 2023_03_02_RAB-5_RAB-7_sand-1_08_Airyscan Processing-1-1-1-1.tif]

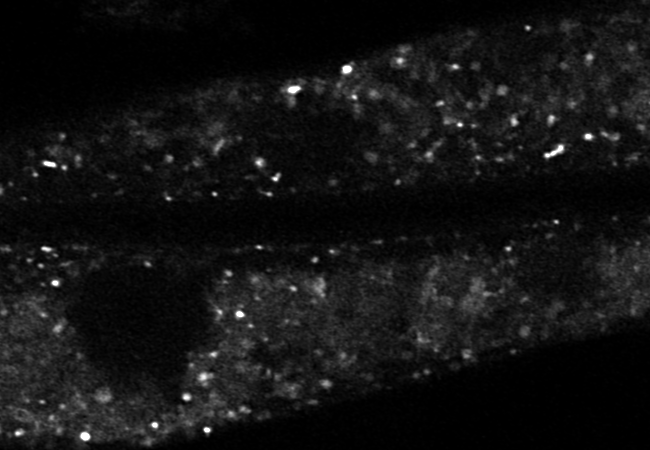

Supplement: Supplementary file 9 — Source data Fig. 7 [file 44318_2025_367_MOESM9_ESM.zip › SD figure 7 /7B/Fig_7_B_Roi/Mock/Gut /ART MGM 2023_03_02_RAB-5_RAB-7_sand-1_08_Airyscan Processing-1-1-1.tif]

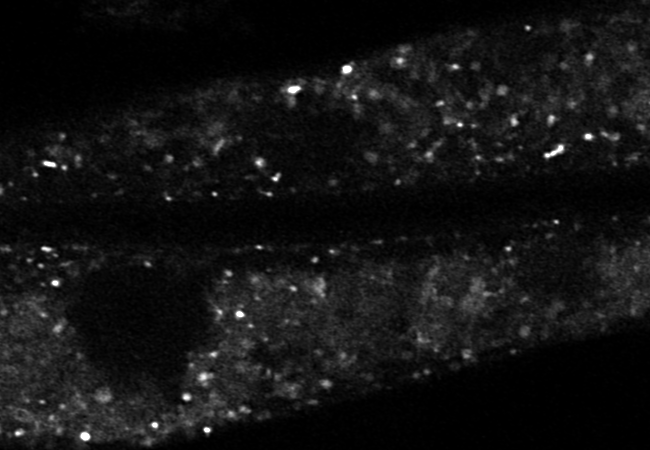

Supplement: Supplementary file 9 — Source data Fig. 7 [file 44318_2025_367_MOESM9_ESM.zip › SD figure 7 /7B/Fig_7_B_Roi/Mock/Gut /ART MC 2023_03_02_RAB-5_RAB-7_sand-1_08_Airyscan Processing-1-1-1-1.tif]

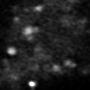

Supplement: Supplementary file 9 — Source data Fig. 7 [file 44318_2025_367_MOESM9_ESM.zip › SD figure 7 /7B/Fig_7_B_Roi/Mock/Gut close up/ART C MGM 2023_03_02_RAB-5_RAB-7_sand-1_08_Airyscan Processing-1-1-1-1.tif]

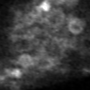

Supplement: Supplementary file 9 — Source data Fig. 7 [file 44318_2025_367_MOESM9_ESM.zip › SD figure 7 /7B/Fig_7_B_Roi/Mock/Gut close up/ART C G 2023_03_02_RAB-5_RAB-7_sand-1_08_Airyscan Processing-1-1-1-1-1.tif]

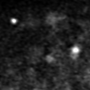

Supplement: Supplementary file 9 — Source data Fig. 7 [file 44318_2025_367_MOESM9_ESM.zip › SD figure 7 /7B/Fig_7_B_Roi/Mock/Gut close up/ART C2 MC 2023_03_02_RAB-5_RAB-7_sand-1_08_Airyscan Processing-1-1-1-1-1.tif]

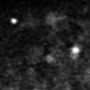

Supplement: Supplementary file 9 — Source data Fig. 7 [file 44318_2025_367_MOESM9_ESM.zip › SD figure 7 /7B/Fig_7_B_Roi/Mock/Gut close up/ART C2 MGM 2023_03_02_RAB-5_RAB-7_sand-1_08_Airyscan Processing-1-1-1-1.tif]

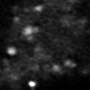

Supplement: Supplementary file 9 — Source data Fig. 7 [file 44318_2025_367_MOESM9_ESM.zip › SD figure 7 /7B/Fig_7_B_Roi/Mock/Gut close up/ART C MC 2023_03_02_RAB-5_RAB-7_sand-1_08_Airyscan Processing-1-1-1-1-1.tif]

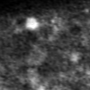

Supplement: Supplementary file 9 — Source data Fig. 7 [file 44318_2025_367_MOESM9_ESM.zip › SD figure 7 /7B/Fig_7_B_Roi/Mock/Gut close up/ART C2 G 2023_03_02_RAB-5_RAB-7_sand-1_08_Airyscan Processing-1-1-1-1-1.tif]

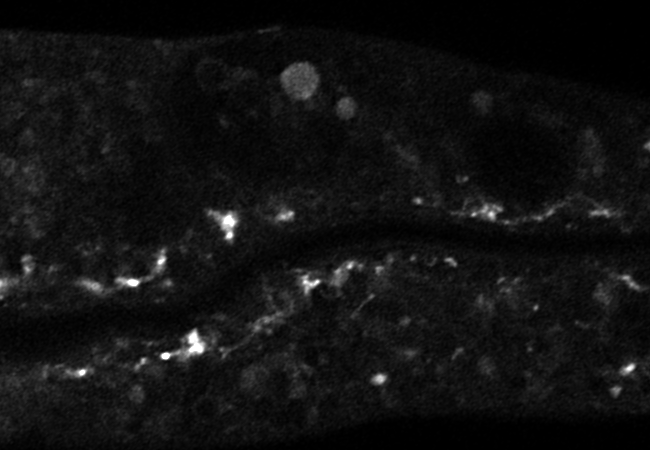

Supplement: Supplementary file 9 — Source data Fig. 7 [file 44318_2025_367_MOESM9_ESM.zip › SD figure 7 /7B/Fig_7_B_Roi/rabx-5 (RNAi)/Gut /ART MGM 2023_03_02_RAB-5_RAB-7_sand-1_rabx-5cloned_02_Airyscan Processing-1-1-1.tif]

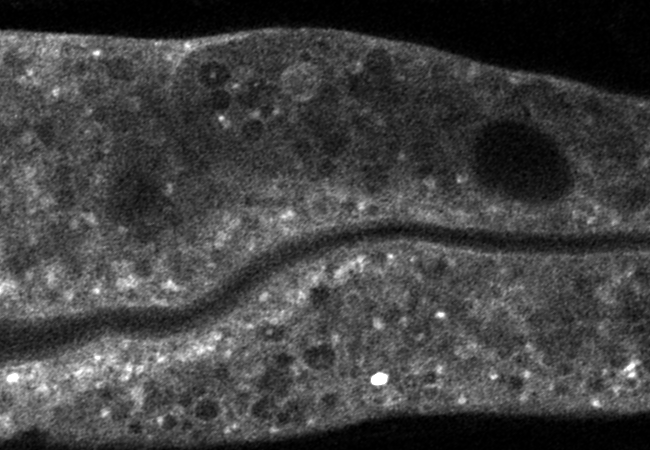

Supplement: Supplementary file 9 — Source data Fig. 7 [file 44318_2025_367_MOESM9_ESM.zip › SD figure 7 /7B/Fig_7_B_Roi/rabx-5 (RNAi)/Gut /ART G 2023_03_02_RAB-5_RAB-7_sand-1_rabx-5cloned_02_Airyscan Processing-1-1-1-1.tif]

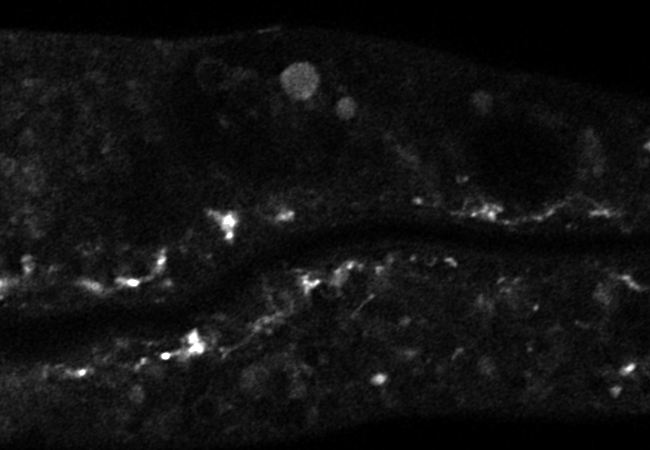

Supplement: Supplementary file 9 — Source data Fig. 7 [file 44318_2025_367_MOESM9_ESM.zip › SD figure 7 /7B/Fig_7_B_Roi/rabx-5 (RNAi)/Gut /ART MC 2023_03_02_RAB-5_RAB-7_sand-1_rabx-5cloned_02_Airyscan Processing-1-1-1-1.tif]

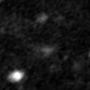

Supplement: Supplementary file 9 — Source data Fig. 7 [file 44318_2025_367_MOESM9_ESM.zip › SD figure 7 /7B/Fig_7_B_Roi/rabx-5 (RNAi)/Gut close up/ART C MGM 2023_03_02_RAB-5_RAB-7_sand-1_rabx-5cloned_02_Airyscan Processing-1-1-1-1.tif]

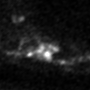

Supplement: Supplementary file 9 — Source data Fig. 7 [file 44318_2025_367_MOESM9_ESM.zip › SD figure 7 /7B/Fig_7_B_Roi/rabx-5 (RNAi)/Gut close up/ART C2 MGM 2023_03_02_RAB-5_RAB-7_sand-1_rabx-5cloned_02_Airyscan Processing-1-1-1-1.tif]

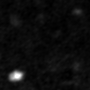

Supplement: Supplementary file 9 — Source data Fig. 7 [file 44318_2025_367_MOESM9_ESM.zip › SD figure 7 /7B/Fig_7_B_Roi/rabx-5 (RNAi)/Gut close up/ART C G 2023_03_02_RAB-5_RAB-7_sand-1_rabx-5cloned_02_Airyscan Processing-1-1-1-1-1.tif]

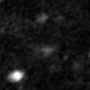

Supplement: Supplementary file 9 — Source data Fig. 7 [file 44318_2025_367_MOESM9_ESM.zip › SD figure 7 /7B/Fig_7_B_Roi/rabx-5 (RNAi)/Gut close up/ART C MC 2023_03_02_RAB-5_RAB-7_sand-1_rabx-5cloned_02_Airyscan Processing-1-1-1-1-1.tif]

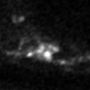

Supplement: Supplementary file 9 — Source data Fig. 7 [file 44318_2025_367_MOESM9_ESM.zip › SD figure 7 /7B/Fig_7_B_Roi/rabx-5 (RNAi)/Gut close up/ART C2 MC 2023_03_02_RAB-5_RAB-7_sand-1_rabx-5cloned_02_Airyscan Processing-1-1-1-1-1.tif]

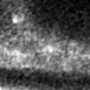

Supplement: Supplementary file 9 — Source data Fig. 7 [file 44318_2025_367_MOESM9_ESM.zip › SD figure 7 /7B/Fig_7_B_Roi/rabx-5 (RNAi)/Gut close up/ART C2 G 2023_03_02_RAB-5_RAB-7_sand-1_rabx-5cloned_02_Airyscan Processing-1-1-1-1-1.tif]

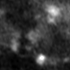

Supplement: Supplementary file 9 — Source data Fig. 7 [file 44318_2025_367_MOESM9_ESM.zip › SD figure 7 /7F/Fig_7_F_Roi/ubq-1 (RNAi)/Gut close up/C1-2024_08_05_RABX-5_RAB-5_ubq-1_05_Airyscan Processing-1-1+-2.tif]

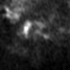

Supplement: Supplementary file 9 — Source data Fig. 7 [file 44318_2025_367_MOESM9_ESM.zip › SD figure 7 /7F/Fig_7_F_Roi/ubq-1 (RNAi)/Gut close up/C1-2024_08_05_RABX-5_RAB-5_ubq-1_05_Airyscan Processing-1-1+-1.tif]

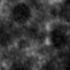

Supplement: Supplementary file 9 — Source data Fig. 7 [file 44318_2025_367_MOESM9_ESM.zip › SD figure 7 /7F/Fig_7_F_Roi/ubq-1 (RNAi)/Gut close up/C2-2024_08_05_RABX-5_RAB-5_ubq-1_05_Airyscan Processing-1-1+-2.png]

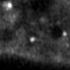

Supplement: Supplementary file 9 — Source data Fig. 7 [file 44318_2025_367_MOESM9_ESM.zip › SD figure 7 /7F/Fig_7_F_Roi/ubq-1 (RNAi)/Gut close up/C2-2024_08_05_RABX-5_RAB-5_ubq-1_05_Airyscan Processing-1-1+-1.png]

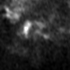

Supplement: Supplementary file 9 — Source data Fig. 7 [file 44318_2025_367_MOESM9_ESM.zip › SD figure 7 /7F/Fig_7_F_Roi/ubq-1 (RNAi)/Gut close up/2024_08_05_RABX-5_RAB-5_ubq-1_05_Airyscan Processing-1-1+-1.tif]

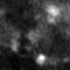

Supplement: Supplementary file 9 — Source data Fig. 7 [file 44318_2025_367_MOESM9_ESM.zip › SD figure 7 /7F/Fig_7_F_Roi/ubq-1 (RNAi)/Gut close up/2024_08_05_RABX-5_RAB-5_ubq-1_05_Airyscan Processing-1-1+-2.tif]

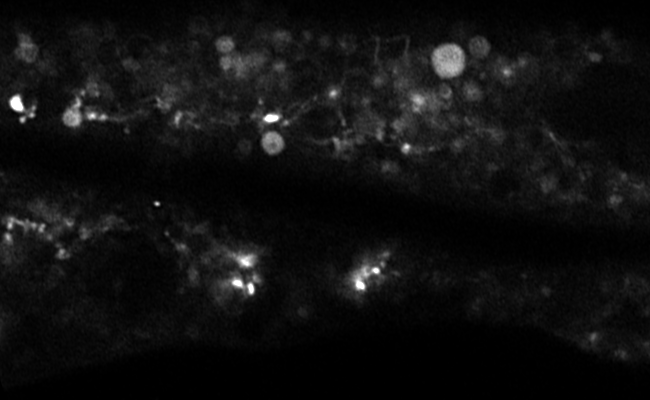

Supplement: Supplementary file 9 — Source data Fig. 7 [file 44318_2025_367_MOESM9_ESM.zip › SD figure 7 /7F/Fig_7_F_Roi/ubq-1 (RNAi)/Gut/C1-2024_08_05_RABX-5_RAB-5_ubq-1_05_Airyscan Processing-1-1+.tif]

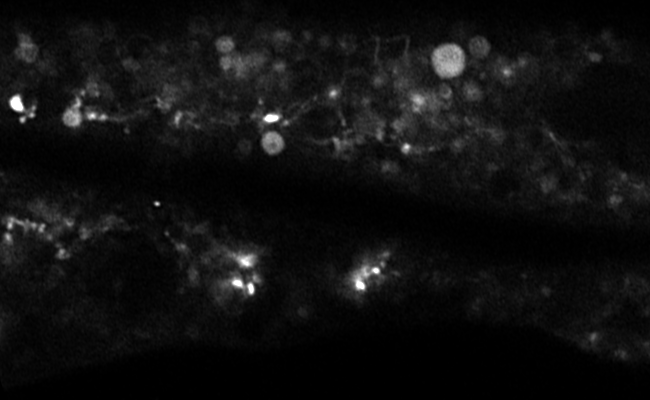

Supplement: Supplementary file 9 — Source data Fig. 7 [file 44318_2025_367_MOESM9_ESM.zip › SD figure 7 /7F/Fig_7_F_Roi/ubq-1 (RNAi)/Gut/2024_08_05_RABX-5_RAB-5_ubq-1_05_Airyscan Processing-1-1+.tif]

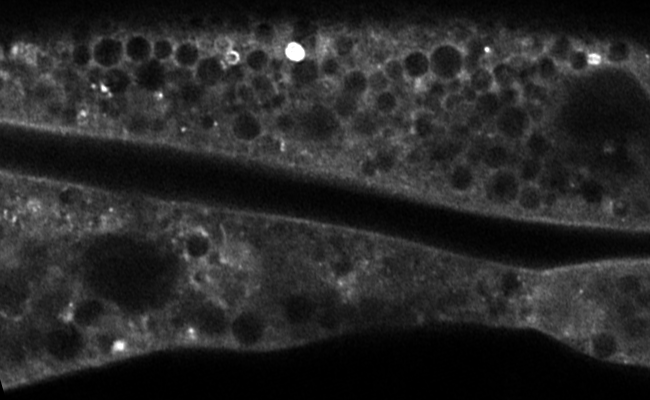

Supplement: Supplementary file 9 — Source data Fig. 7 [file 44318_2025_367_MOESM9_ESM.zip › SD figure 7 /7F/Fig_7_F_Roi/ubq-1 (RNAi)/Gut/C2-2024_08_05_RABX-5_RAB-5_ubq-1_05_Airyscan Processing-1-1+.tif]

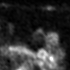

Supplement: Supplementary file 9 — Source data Fig. 7 [file 44318_2025_367_MOESM9_ESM.zip › SD figure 7 /7F/Fig_7_F_Roi/Mock/Gut close up/2024_08_05_RABX-5_RAB-5_neg_06_Airyscan Processing-1-1+-1.tif]

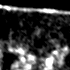

Supplement: Supplementary file 9 — Source data Fig. 7 [file 44318_2025_367_MOESM9_ESM.zip › SD figure 7 /7F/Fig_7_F_Roi/Mock/Gut close up/C2-2024_08_05_RABX-5_RAB-5_neg_06_Airyscan Processing-1-1+-1.png]

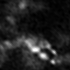

Supplement: Supplementary file 9 — Source data Fig. 7 [file 44318_2025_367_MOESM9_ESM.zip › SD figure 7 /7F/Fig_7_F_Roi/Mock/Gut close up/2024_08_05_RABX-5_RAB-5_neg_06_Airyscan Processing-1-1+-2.tif]

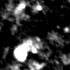

Supplement: Supplementary file 9 — Source data Fig. 7 [file 44318_2025_367_MOESM9_ESM.zip › SD figure 7 /7F/Fig_7_F_Roi/Mock/Gut close up/C2-2024_08_05_RABX-5_RAB-5_neg_06_Airyscan Processing-1-1+-2.png]

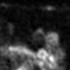

Supplement: Supplementary file 9 — Source data Fig. 7 [file 44318_2025_367_MOESM9_ESM.zip › SD figure 7 /7F/Fig_7_F_Roi/Mock/Gut close up/C1-2024_08_05_RABX-5_RAB-5_neg_06_Airyscan Processing-1-1+-1.tif]

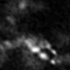

Supplement: Supplementary file 9 — Source data Fig. 7 [file 44318_2025_367_MOESM9_ESM.zip › SD figure 7 /7F/Fig_7_F_Roi/Mock/Gut close up/C1-2024_08_05_RABX-5_RAB-5_neg_06_Airyscan Processing-1-1+-2.tif]

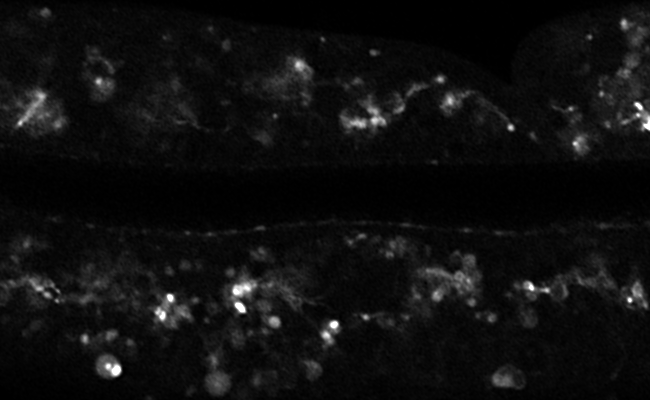

Supplement: Supplementary file 9 — Source data Fig. 7 [file 44318_2025_367_MOESM9_ESM.zip › SD figure 7 /7F/Fig_7_F_Roi/Mock/Gut/2024_08_05_RABX-5_RAB-5_neg_06_Airyscan Processing-1-1+.tif]

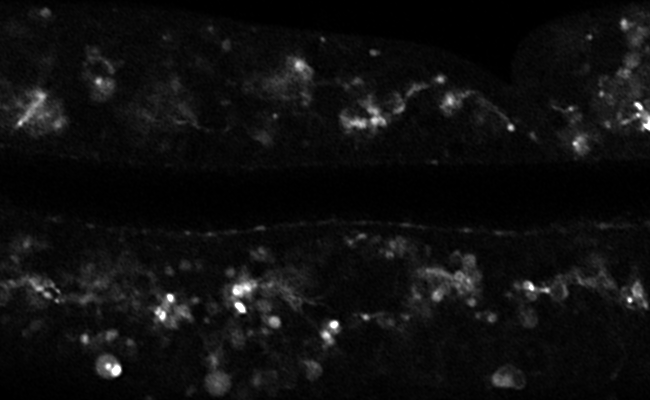

Supplement: Supplementary file 9 — Source data Fig. 7 [file 44318_2025_367_MOESM9_ESM.zip › SD figure 7 /7F/Fig_7_F_Roi/Mock/Gut/C1-2024_08_05_RABX-5_RAB-5_neg_06_Airyscan Processing-1-1+.tif]

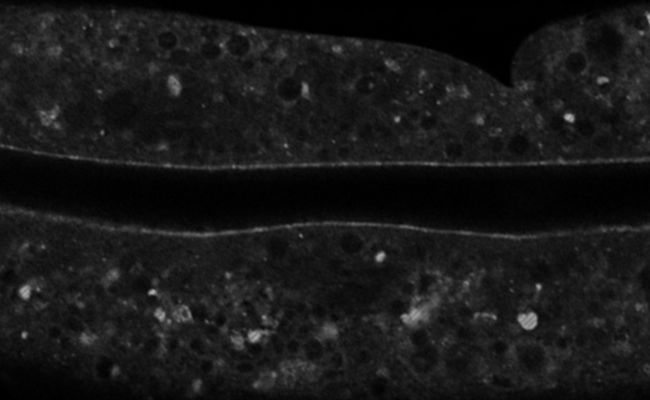

Supplement: Supplementary file 9 — Source data Fig. 7 [file 44318_2025_367_MOESM9_ESM.zip › SD figure 7 /7F/Fig_7_F_Roi/Mock/Gut/C2-2024_08_05_RABX-5_RAB-5_neg_06_Airyscan Processing-1-1+.tif]

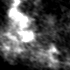

Supplement: Supplementary file 9 — Source data Fig. 7 [file 44318_2025_367_MOESM9_ESM.zip › SD figure 7 /7F/Fig_7_F_Roi/hgrs-1 (RNAi)/Gut close up/C2-2024_08_06_RABX-5_RAB-5_hgrs-1_06_Airyscan Processing-1-1+-2.png]

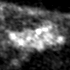

Supplement: Supplementary file 9 — Source data Fig. 7 [file 44318_2025_367_MOESM9_ESM.zip › SD figure 7 /7F/Fig_7_F_Roi/hgrs-1 (RNAi)/Gut close up/C2-2024_08_06_RABX-5_RAB-5_hgrs-1_06_Airyscan Processing-1-1+-1.png]

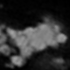

Supplement: Supplementary file 9 — Source data Fig. 7 [file 44318_2025_367_MOESM9_ESM.zip › SD figure 7 /7F/Fig_7_F_Roi/hgrs-1 (RNAi)/Gut close up/C1-2024_08_06_RABX-5_RAB-5_hgrs-1_06_Airyscan Processing-1-1+-1.tif]

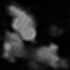

Supplement: Supplementary file 9 — Source data Fig. 7 [file 44318_2025_367_MOESM9_ESM.zip › SD figure 7 /7F/Fig_7_F_Roi/hgrs-1 (RNAi)/Gut close up/C1-2024_08_06_RABX-5_RAB-5_hgrs-1_06_Airyscan Processing-1-1+-2.tif]

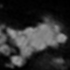

Supplement: Supplementary file 9 — Source data Fig. 7 [file 44318_2025_367_MOESM9_ESM.zip › SD figure 7 /7F/Fig_7_F_Roi/hgrs-1 (RNAi)/Gut close up/2024_08_06_RABX-5_RAB-5_hgrs-1_06_Airyscan Processing-1-1+-1.tif]

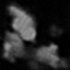

Supplement: Supplementary file 9 — Source data Fig. 7 [file 44318_2025_367_MOESM9_ESM.zip › SD figure 7 /7F/Fig_7_F_Roi/hgrs-1 (RNAi)/Gut close up/2024_08_06_RABX-5_RAB-5_hgrs-1_06_Airyscan Processing-1-1+-2.tif]

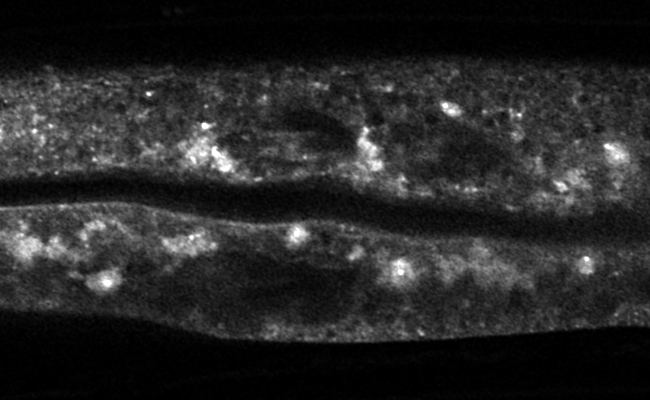

Supplement: Supplementary file 9 — Source data Fig. 7 [file 44318_2025_367_MOESM9_ESM.zip › SD figure 7 /7F/Fig_7_F_Roi/hgrs-1 (RNAi)/Gut/C2-2024_08_06_RABX-5_RAB-5_hgrs-1_06_Airyscan Processing-1-1+.tif]

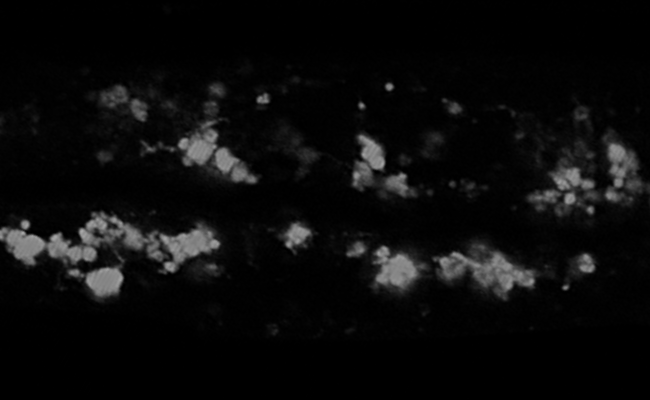

Supplement: Supplementary file 9 — Source data Fig. 7 [file 44318_2025_367_MOESM9_ESM.zip › SD figure 7 /7F/Fig_7_F_Roi/hgrs-1 (RNAi)/Gut/2024_08_06_RABX-5_RAB-5_hgrs-1_06_Airyscan Processing-1-1+.tif]

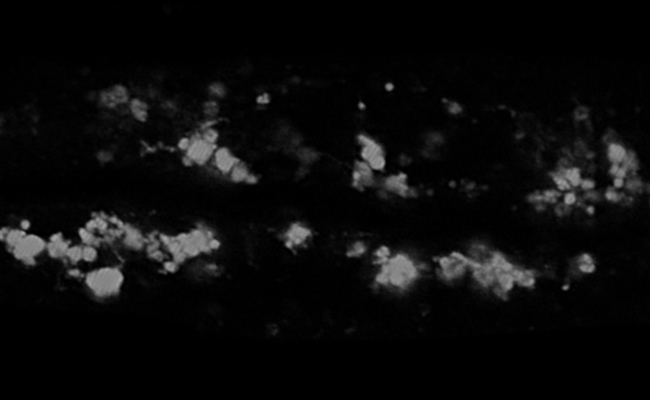

Supplement: Supplementary file 9 — Source data Fig. 7 [file 44318_2025_367_MOESM9_ESM.zip › SD figure 7 /7F/Fig_7_F_Roi/hgrs-1 (RNAi)/Gut/C1-2024_08_06_RABX-5_RAB-5_hgrs-1_06_Airyscan Processing-1-1+.tif]

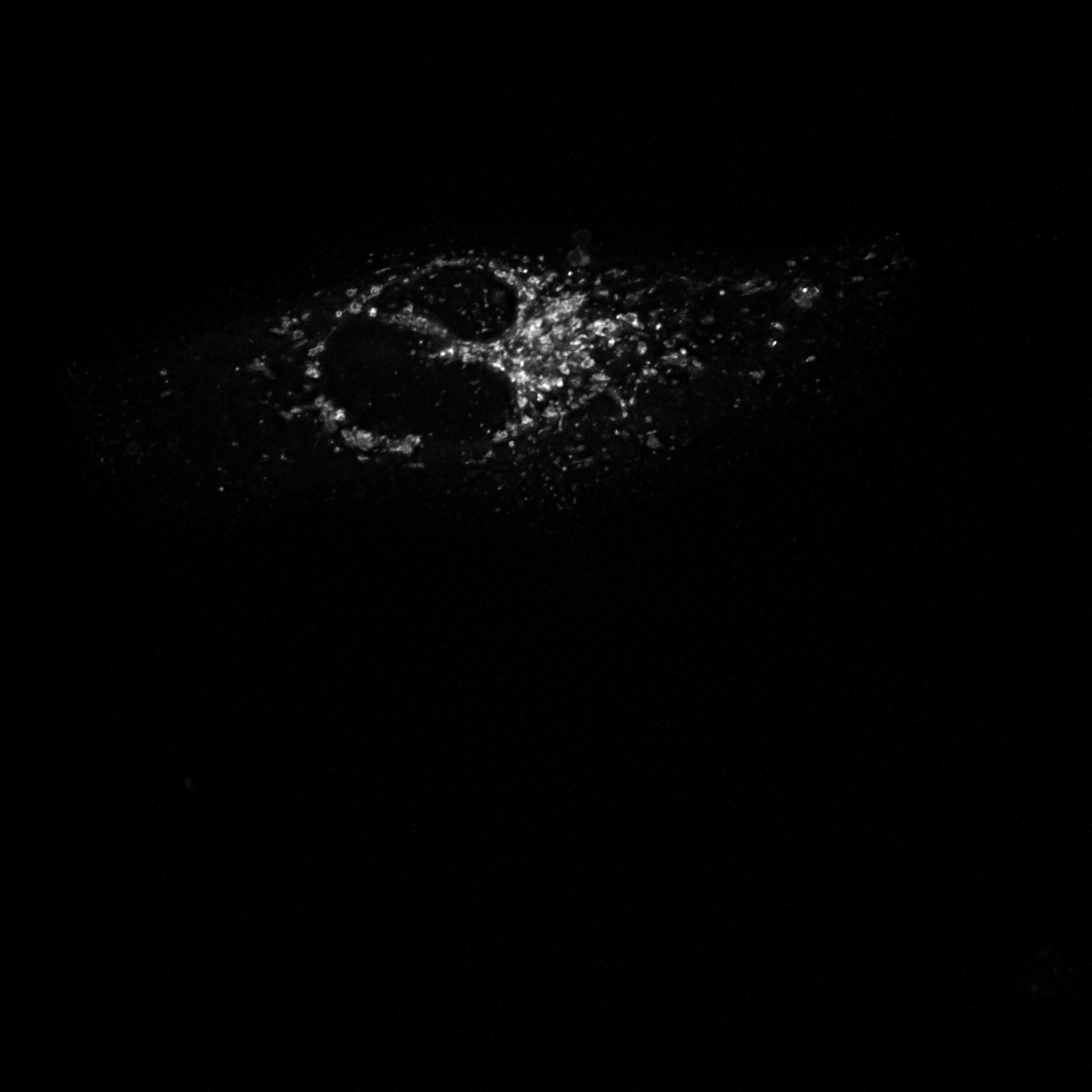

Supplement: Supplementary file 10 — Source data Fig. 8 [file 44318_2025_367_MOESM10_ESM.zip › SD figure 8/8A/Fig_8_A_data/CHPM6 KO/RAB7_Experiment-443_czi_633c5b42a0119_hrm.ics.tiff]

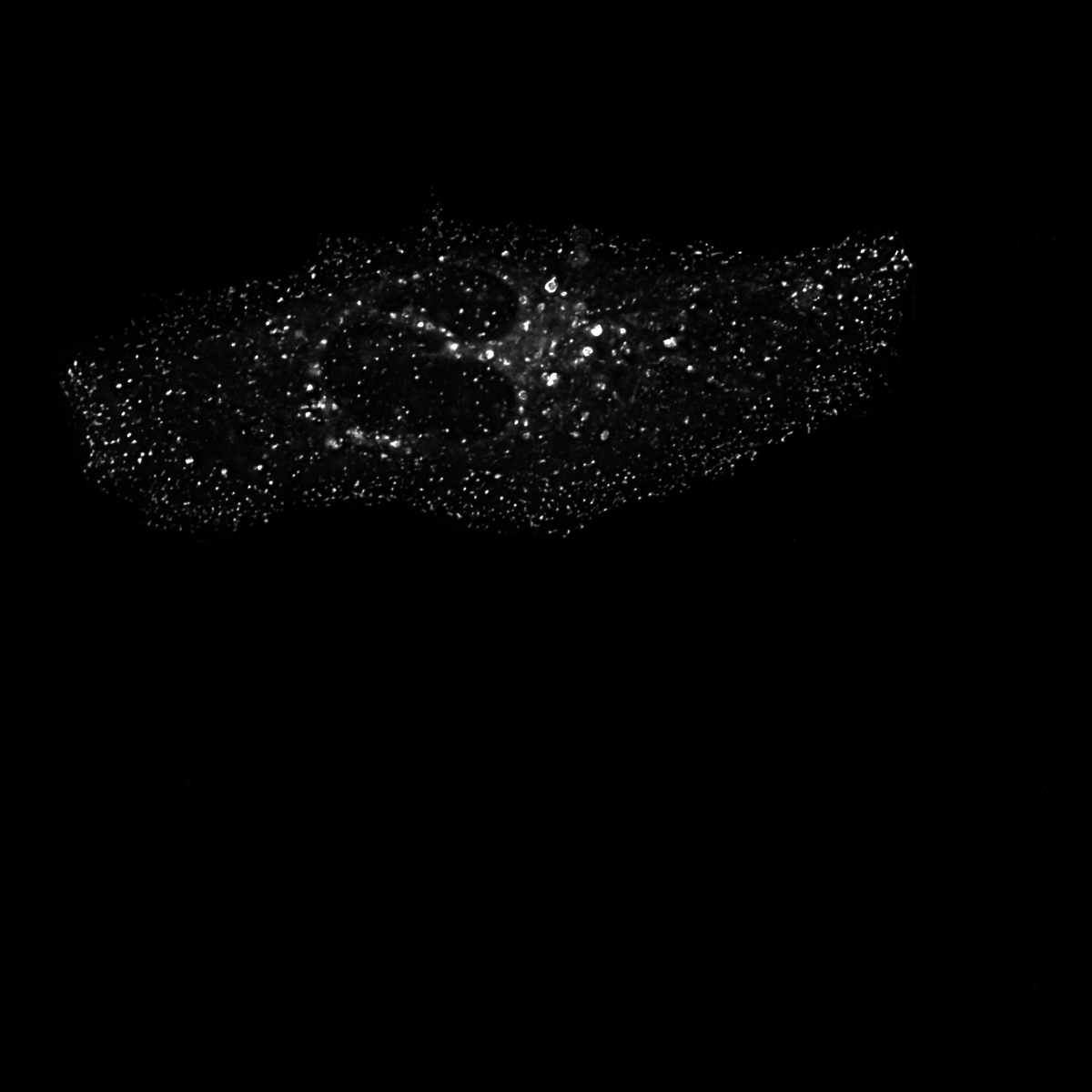

Supplement: Supplementary file 10 — Source data Fig. 8 [file 44318_2025_367_MOESM10_ESM.zip › SD figure 8/8A/Fig_8_A_data/CHPM6 KO/RAB5_Experiment-443_czi_633c5b42a0119_hrm.ics.tiff]

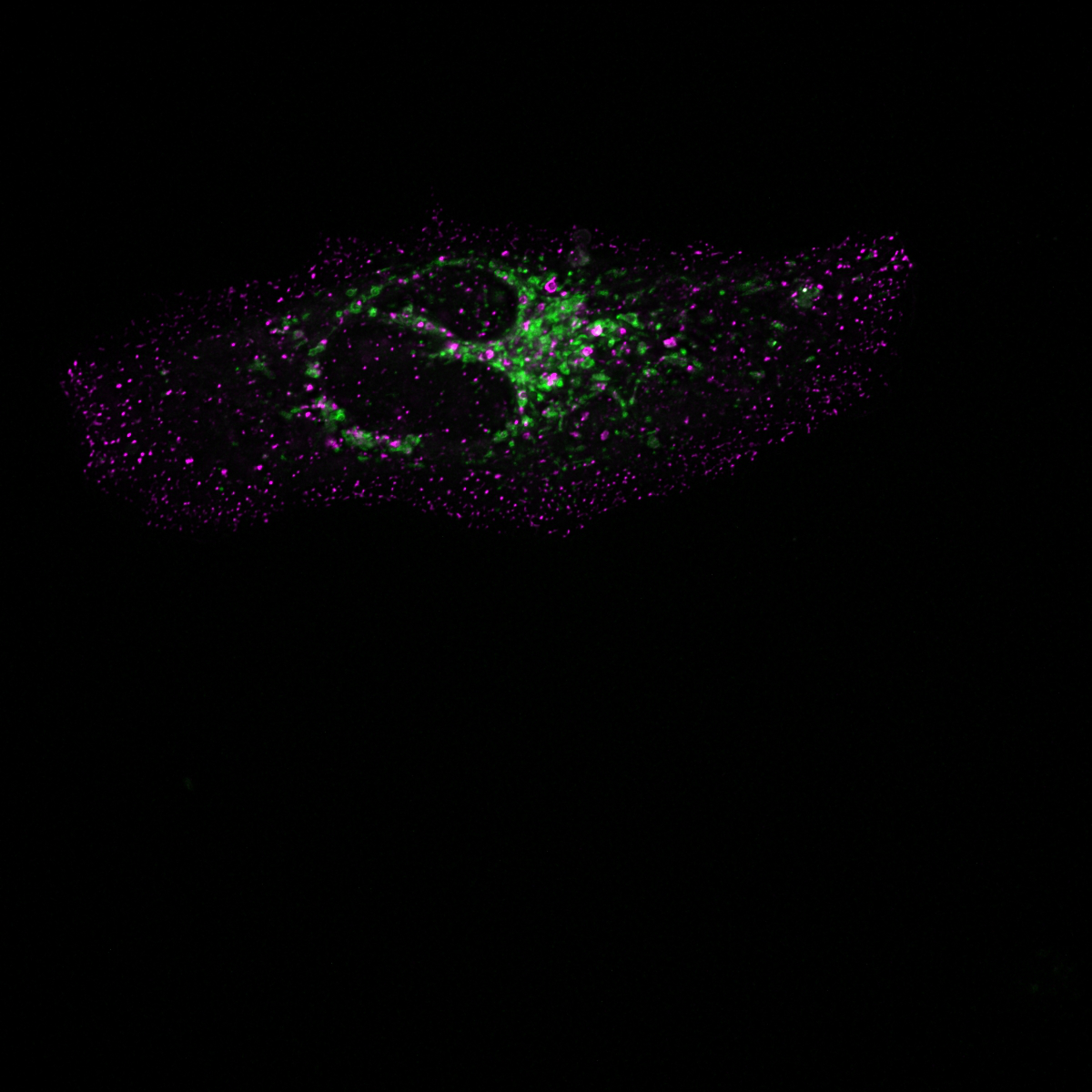

Supplement: Supplementary file 10 — Source data Fig. 8 [file 44318_2025_367_MOESM10_ESM.zip › SD figure 8/8A/Fig_8_A_data/CHPM6 KO/MERGED_Experiment-443_czi_633c5b42a0119_hrm.ics.tiff]

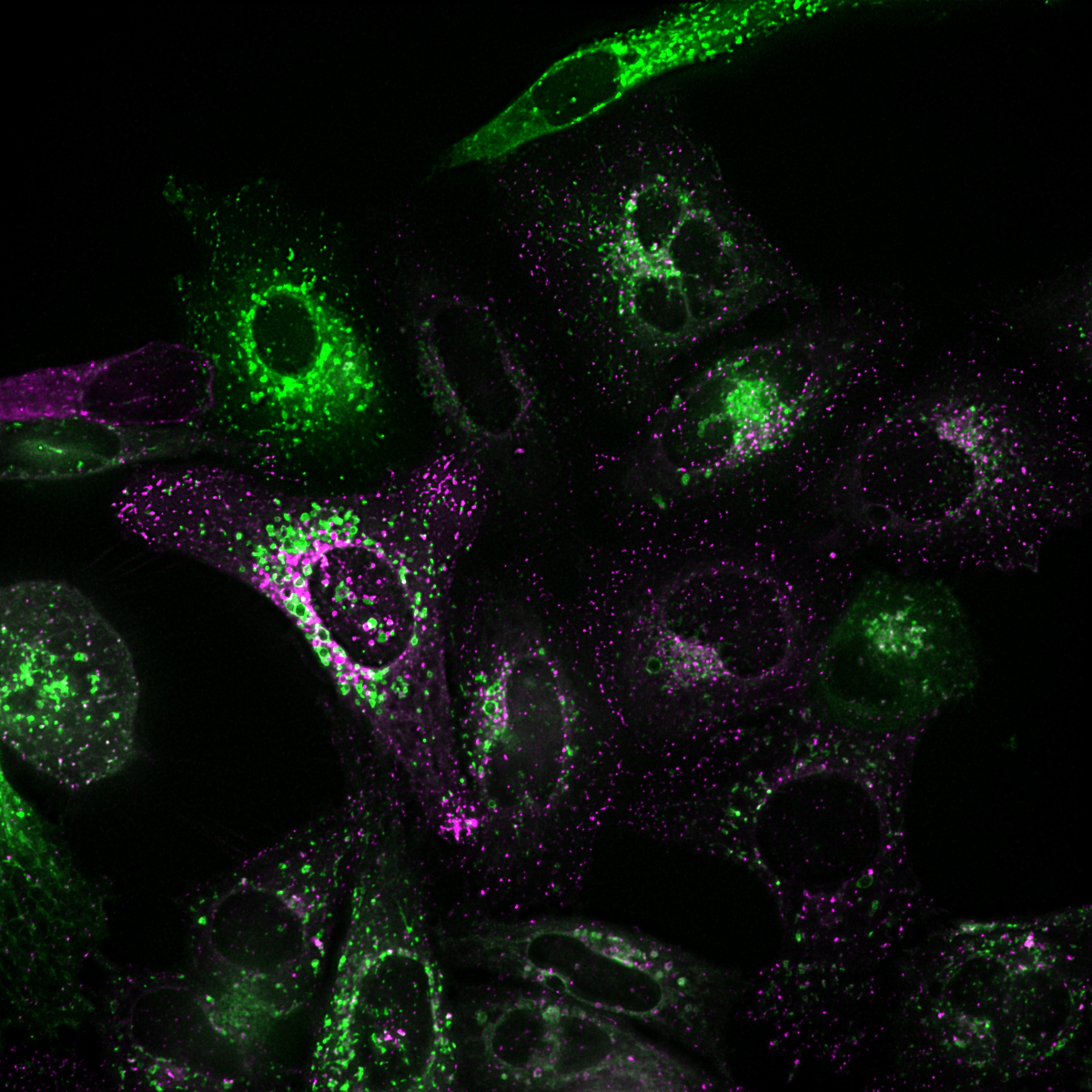

Supplement: Supplementary file 10 — Source data Fig. 8 [file 44318_2025_367_MOESM10_ESM.zip › SD figure 8/8A/Fig_8_A_data/HRS KO/MERGED_Experiment-358_czi_63175528cd0cf_hrm.ics.tiff]

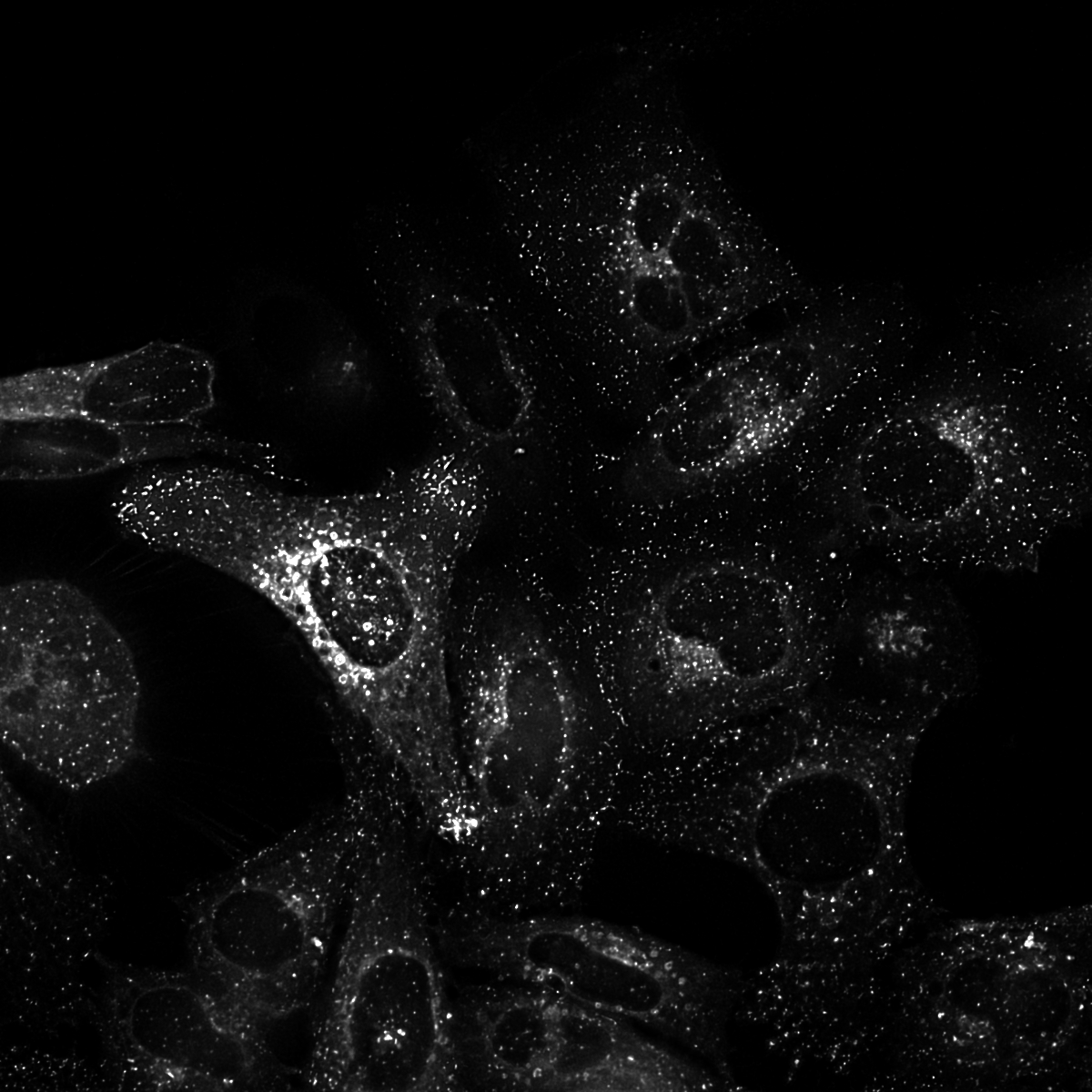

Supplement: Supplementary file 10 — Source data Fig. 8 [file 44318_2025_367_MOESM10_ESM.zip › SD figure 8/8A/Fig_8_A_data/HRS KO/RAB5_Experiment-358_czi_63175528cd0cf_hrm.ics.tiff]

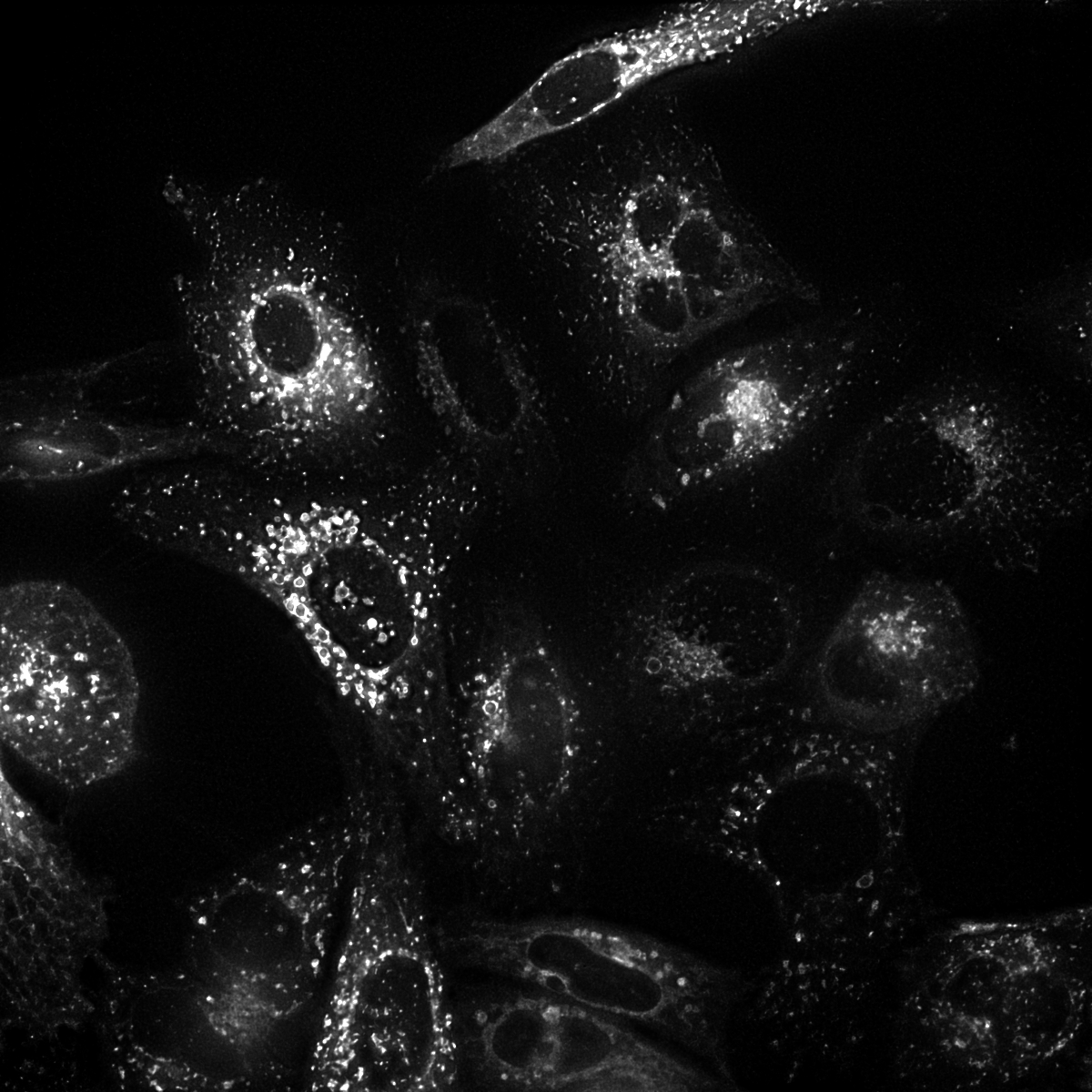

Supplement: Supplementary file 10 — Source data Fig. 8 [file 44318_2025_367_MOESM10_ESM.zip › SD figure 8/8A/Fig_8_A_data/HRS KO/RAB7_Experiment-358_czi_63175528cd0cf_hrm.ics.tiff]

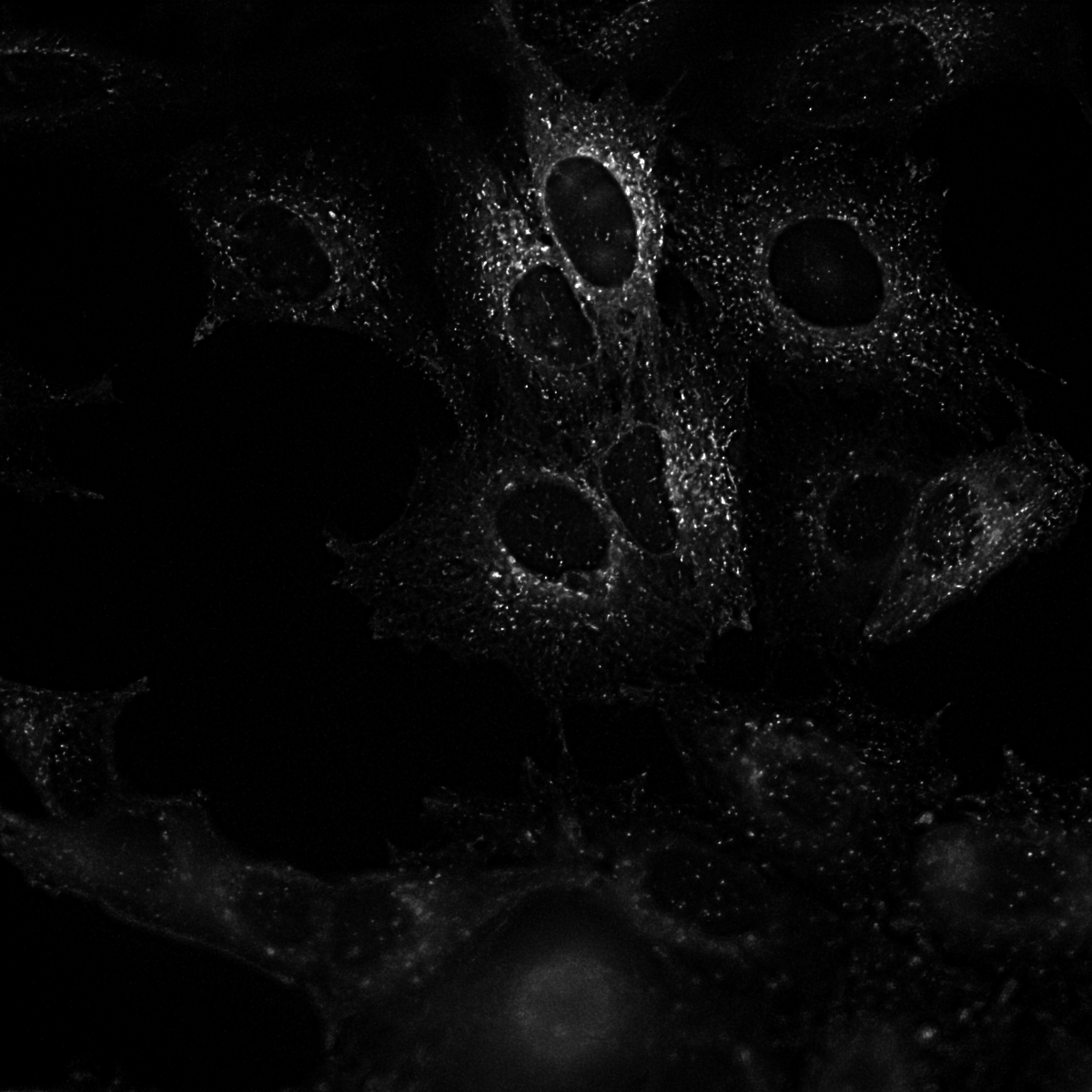

Supplement: Supplementary file 10 — Source data Fig. 8 [file 44318_2025_367_MOESM10_ESM.zip › SD figure 8/8A/Fig_8_A_data/CONTROL/RAB7_Experiment-386_czi_63175528d574f_hrm.ics.tiff]

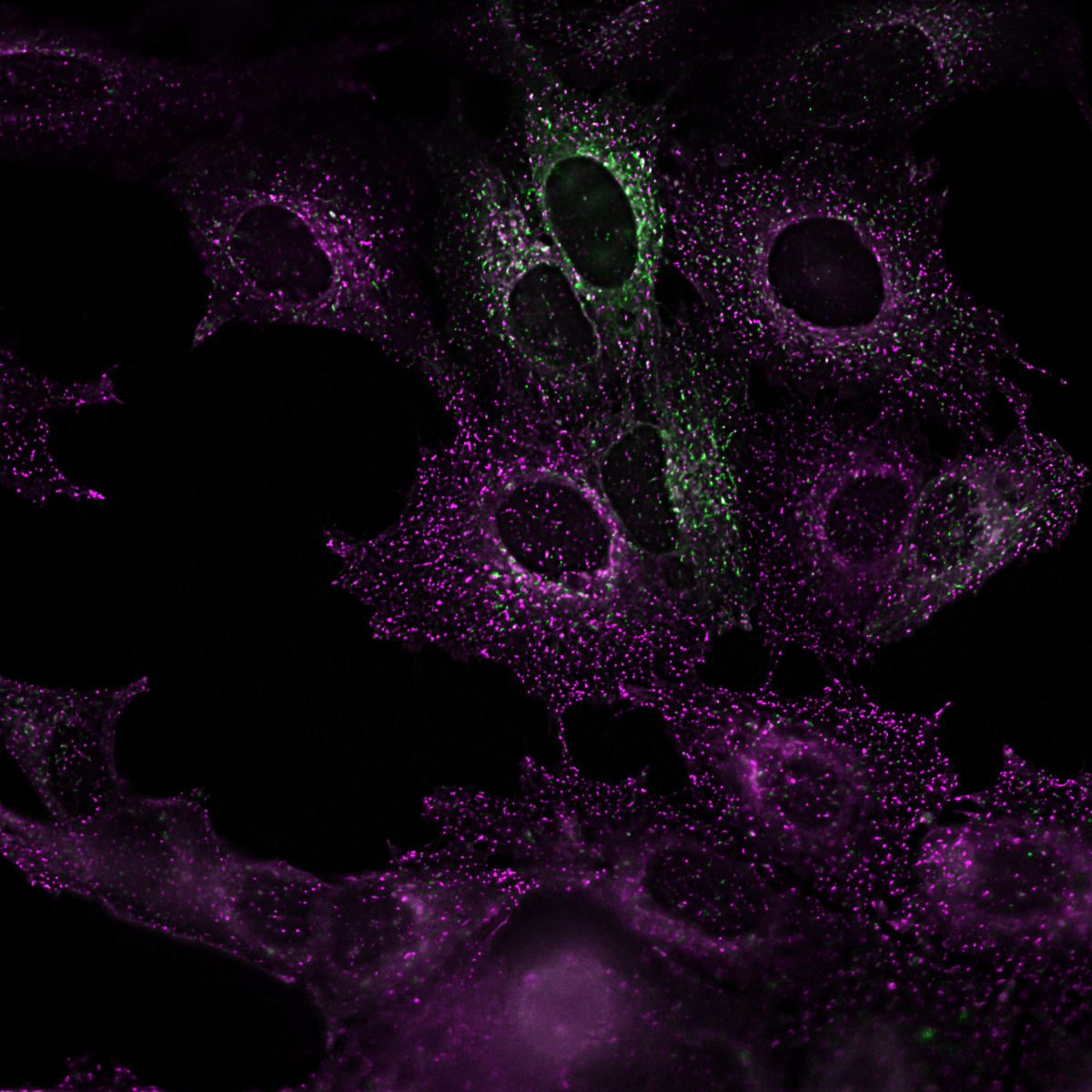

Supplement: Supplementary file 10 — Source data Fig. 8 [file 44318_2025_367_MOESM10_ESM.zip › SD figure 8/8A/Fig_8_A_data/CONTROL/MERGED_Experiment-386_czi_63175528d574f_hrm.ics.tiff]

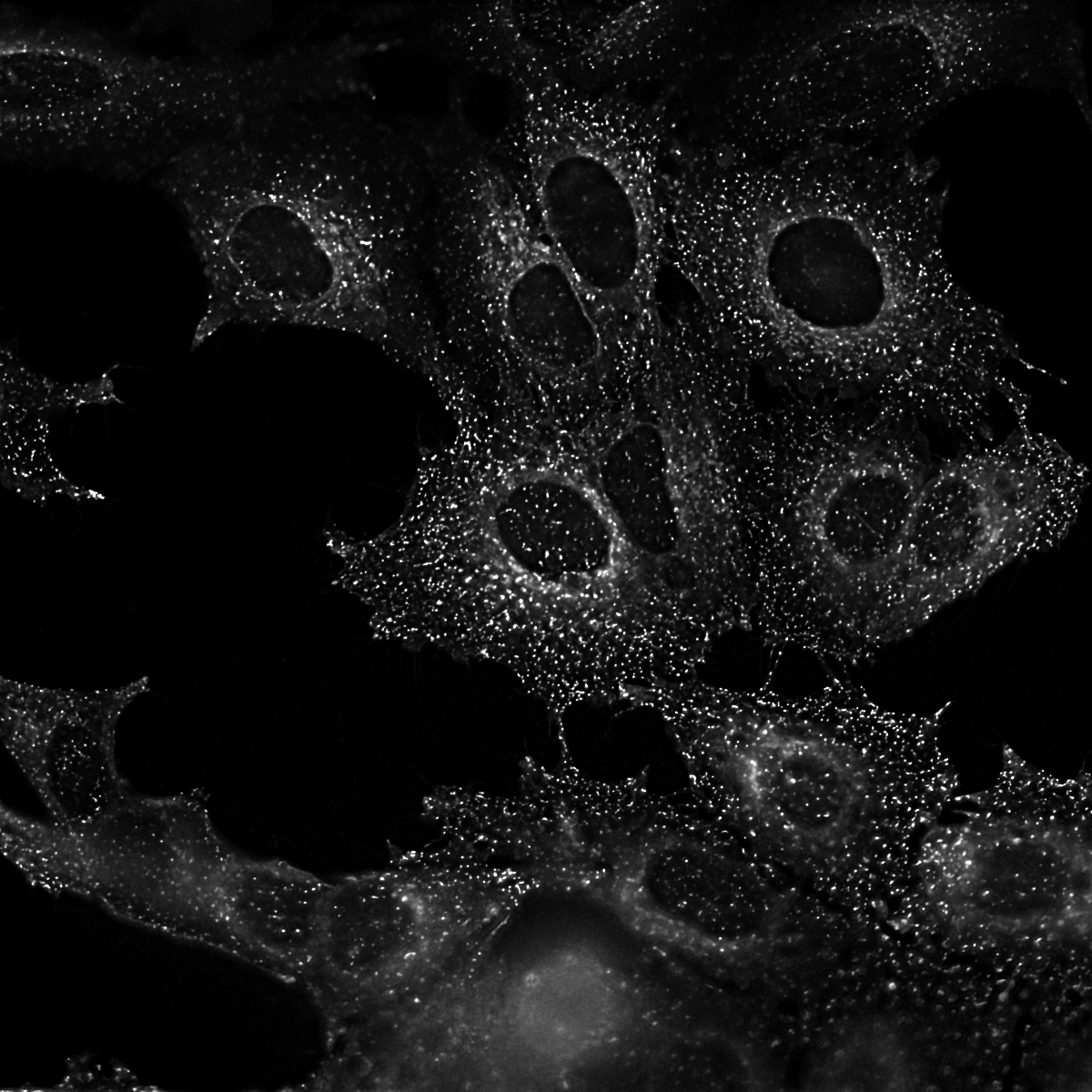

Supplement: Supplementary file 10 — Source data Fig. 8 [file 44318_2025_367_MOESM10_ESM.zip › SD figure 8/8A/Fig_8_A_data/CONTROL/RAB5_Experiment-386_czi_63175528d574f_hrm.ics.tiff]

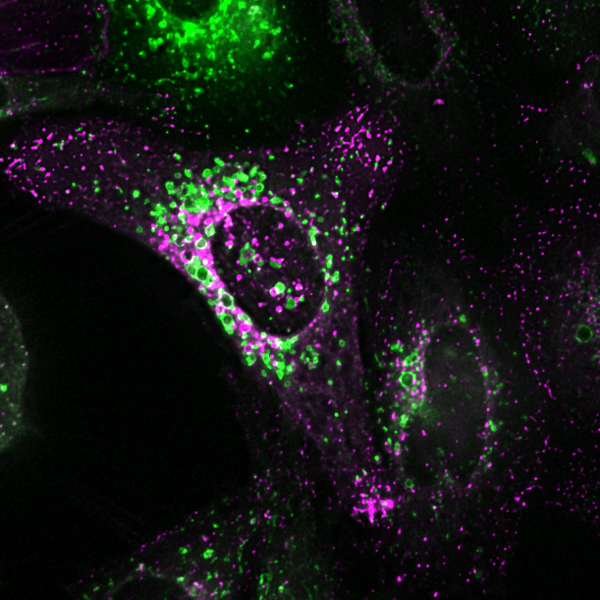

Supplement: Supplementary file 10 — Source data Fig. 8 [file 44318_2025_367_MOESM10_ESM.zip › SD figure 8/8A/Fig_8_A_Roi/HRS KO/MERGED_Experiment-358_czi_63175528cd0cf_hrm.ics.tiff]

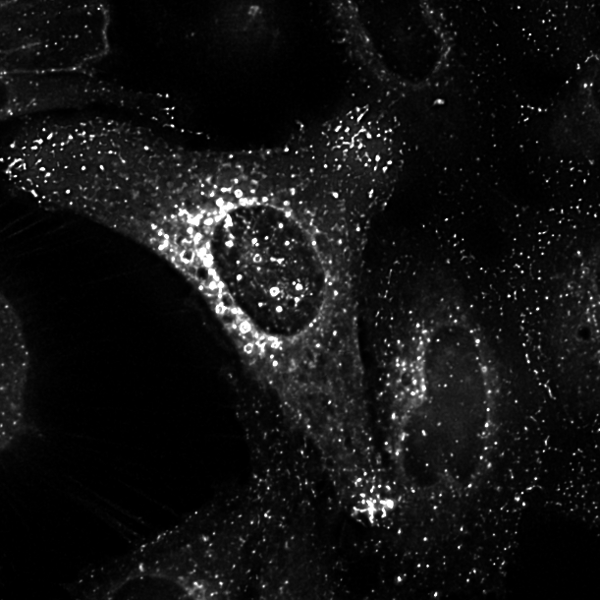

Supplement: Supplementary file 10 — Source data Fig. 8 [file 44318_2025_367_MOESM10_ESM.zip › SD figure 8/8A/Fig_8_A_Roi/HRS KO/RAB5_Experiment-358_czi_63175528cd0cf_hrm.ics.tiff]

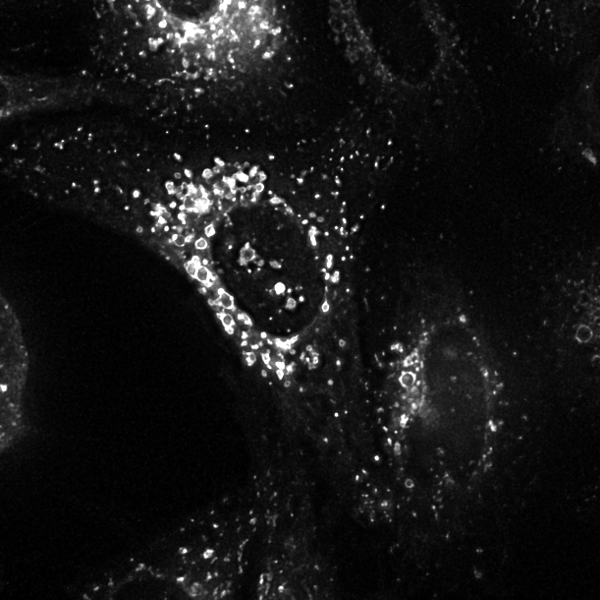

Supplement: Supplementary file 10 — Source data Fig. 8 [file 44318_2025_367_MOESM10_ESM.zip › SD figure 8/8A/Fig_8_A_Roi/HRS KO/RAB7_Experiment-358_czi_63175528cd0cf_hrm.ics.tiff]

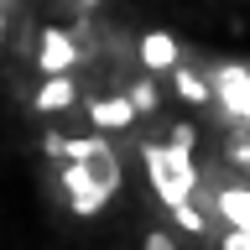

Supplement: Supplementary file 10 — Source data Fig. 8 [file 44318_2025_367_MOESM10_ESM.zip › SD figure 8/8A/Fig_8_A_Roi/HRS KO/RAB7 CU_Experiment-358_czi_63175528cd0cf_hrm.ics.tiff]

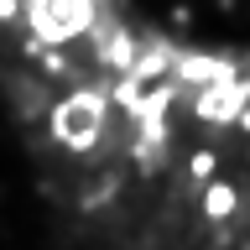

Supplement: Supplementary file 10 — Source data Fig. 8 [file 44318_2025_367_MOESM10_ESM.zip › SD figure 8/8A/Fig_8_A_Roi/HRS KO/RAB5 CU_Experiment-358_czi_63175528cd0cf_hrm.ics.tiff]

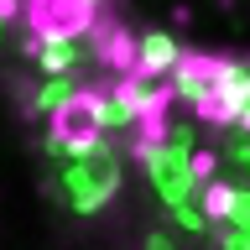

Supplement: Supplementary file 10 — Source data Fig. 8 [file 44318_2025_367_MOESM10_ESM.zip › SD figure 8/8A/Fig_8_A_Roi/HRS KO/MERGED CU_Experiment-358_czi_63175528cd0cf_hrm.ics.tiff]

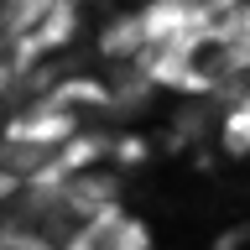

Supplement: Supplementary file 10 — Source data Fig. 8 [file 44318_2025_367_MOESM10_ESM.zip › SD figure 8/8A/Fig_8_A_Roi/CHMP6 KO/RAB7 CU_Experiment-443_czi_633c5b42a0119_hrm.ics.tiff]

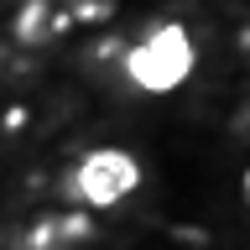

Supplement: Supplementary file 10 — Source data Fig. 8 [file 44318_2025_367_MOESM10_ESM.zip › SD figure 8/8A/Fig_8_A_Roi/CHMP6 KO/RAB5 CU_Experiment-443_czi_633c5b42a0119_hrm.ics.tiff]

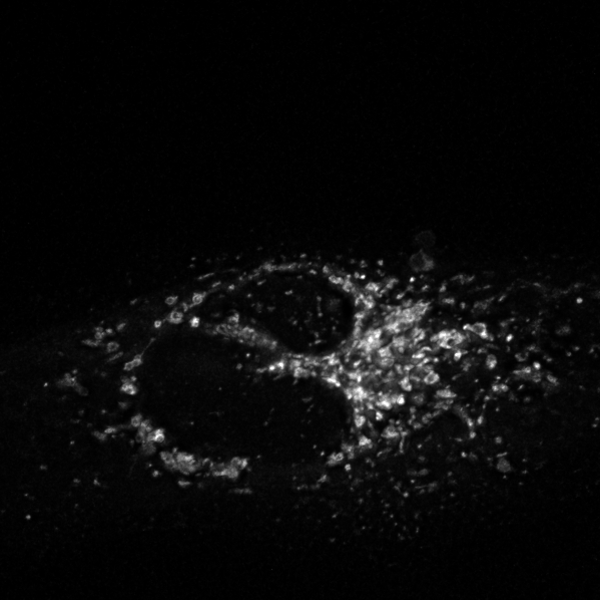

Supplement: Supplementary file 10 — Source data Fig. 8 [file 44318_2025_367_MOESM10_ESM.zip › SD figure 8/8A/Fig_8_A_Roi/CHMP6 KO/RAB7_Experiment-443_czi_633c5b42a0119_hrm.ics.tiff]

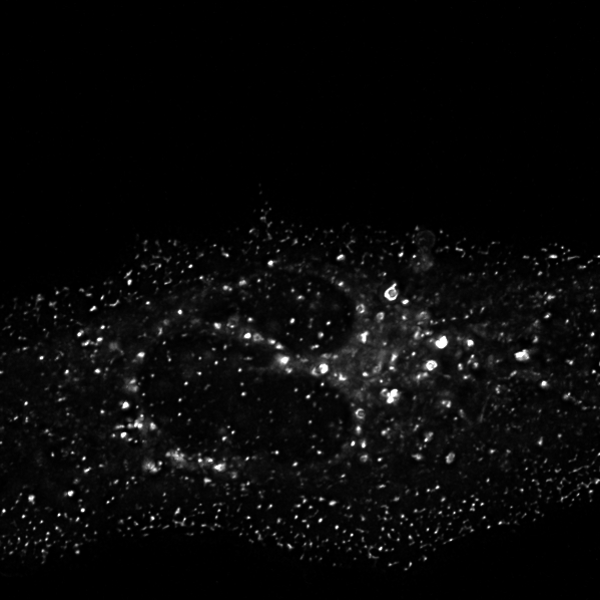

Supplement: Supplementary file 10 — Source data Fig. 8 [file 44318_2025_367_MOESM10_ESM.zip › SD figure 8/8A/Fig_8_A_Roi/CHMP6 KO/RAB5_Experiment-443_czi_633c5b42a0119_hrm.ics.tiff]

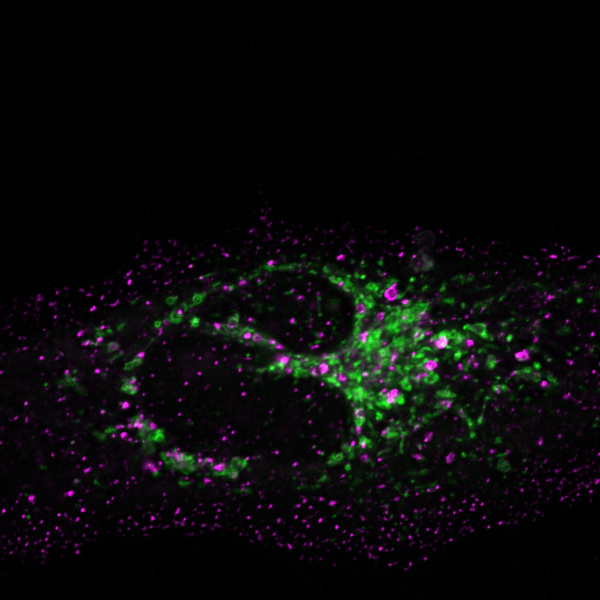

Supplement: Supplementary file 10 — Source data Fig. 8 [file 44318_2025_367_MOESM10_ESM.zip › SD figure 8/8A/Fig_8_A_Roi/CHMP6 KO/MERGED_Experiment-443_czi_633c5b42a0119_hrm.ics.tiff]

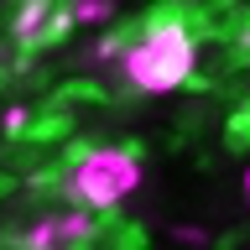

Supplement: Supplementary file 10 — Source data Fig. 8 [file 44318_2025_367_MOESM10_ESM.zip › SD figure 8/8A/Fig_8_A_Roi/CHMP6 KO/MERGED CU_Experiment-443_czi_633c5b42a0119_hrm.ics.tiff]

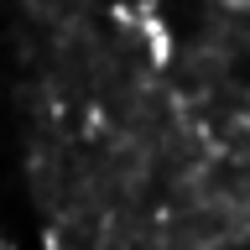

Supplement: Supplementary file 10 — Source data Fig. 8 [file 44318_2025_367_MOESM10_ESM.zip › SD figure 8/8A/Fig_8_A_Roi/CONTROL/RAB7 CU_Experiment-386_czi_63175528d574f_hrm.ics.tiff]

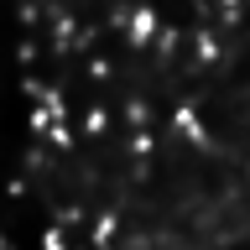

Supplement: Supplementary file 10 — Source data Fig. 8 [file 44318_2025_367_MOESM10_ESM.zip › SD figure 8/8A/Fig_8_A_Roi/CONTROL/RAB5 CU_Experiment-386_czi_63175528d574f_hrm.ics.tiff]

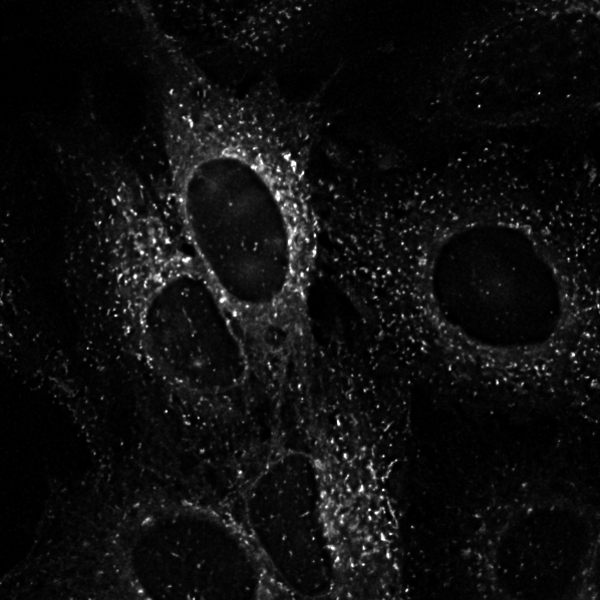

Supplement: Supplementary file 10 — Source data Fig. 8 [file 44318_2025_367_MOESM10_ESM.zip › SD figure 8/8A/Fig_8_A_Roi/CONTROL/RAB7_Experiment-386_czi_63175528d574f_hrm.ics.tiff]

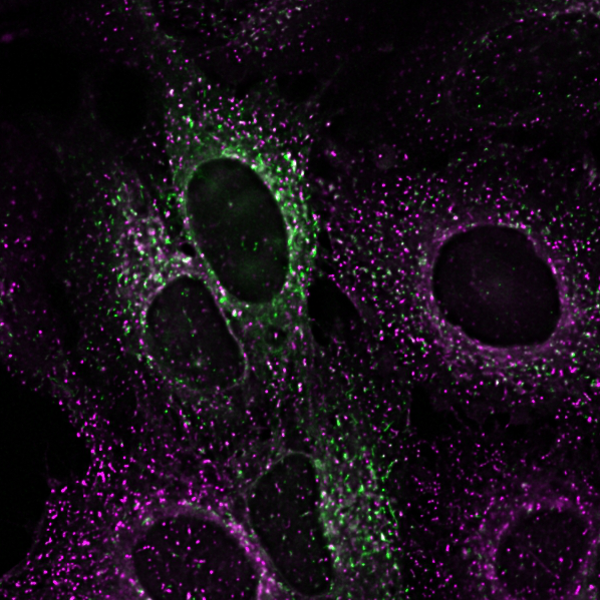

Supplement: Supplementary file 10 — Source data Fig. 8 [file 44318_2025_367_MOESM10_ESM.zip › SD figure 8/8A/Fig_8_A_Roi/CONTROL/MERGED_Experiment-386_czi_63175528d574f_hrm.ics.tiff]

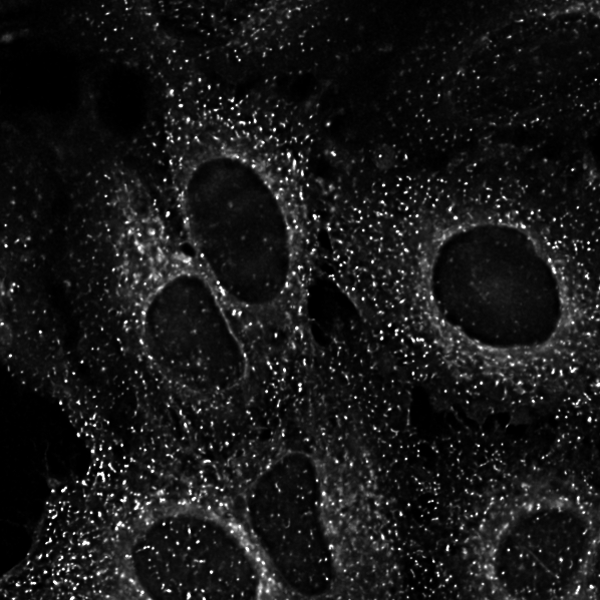

Supplement: Supplementary file 10 — Source data Fig. 8 [file 44318_2025_367_MOESM10_ESM.zip › SD figure 8/8A/Fig_8_A_Roi/CONTROL/RAB5_Experiment-386_czi_63175528d574f_hrm.ics.tiff]

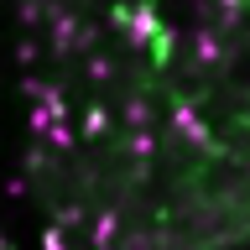

Supplement: Supplementary file 10 — Source data Fig. 8 [file 44318_2025_367_MOESM10_ESM.zip › SD figure 8/8A/Fig_8_A_Roi/CONTROL/MERGED CU_Experiment-386_czi_63175528d574f_hrm.ics.tiff]

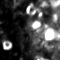

Supplement: Supplementary file 10 — Source data Fig. 8 [file 44318_2025_367_MOESM10_ESM.zip › SD figure 8/8D/Fig_8_D_Roi/CCZ1 KO/CCZ1 KO CU_Experiment-734_czi_63b690404947e_hrm.ics.tiff]

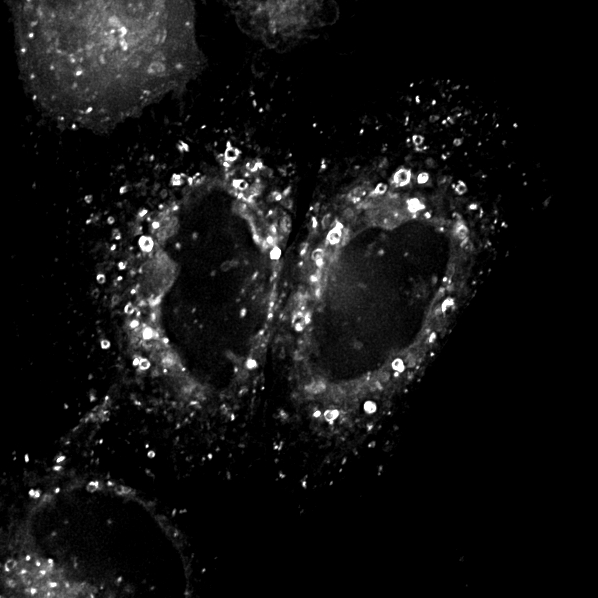

Supplement: Supplementary file 10 — Source data Fig. 8 [file 44318_2025_367_MOESM10_ESM.zip › SD figure 8/8D/Fig_8_D_Roi/CCZ1 KO/CCZ1 KO_Experiment-734_czi_63b690404947e_hrm.ics.tiff]

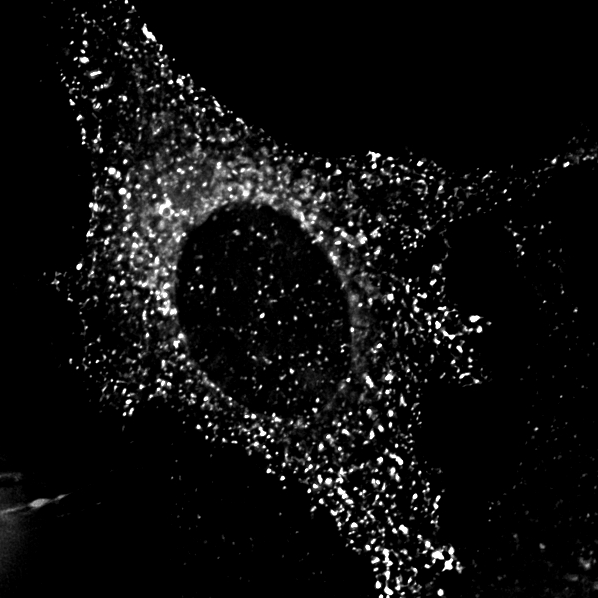

Supplement: Supplementary file 10 — Source data Fig. 8 [file 44318_2025_367_MOESM10_ESM.zip › SD figure 8/8D/Fig_8_D_Roi/CONTROL/CONTROL_Experiment-754_czi_63b690404e74d_hrm.ics.tiff]

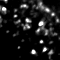

Supplement: Supplementary file 10 — Source data Fig. 8 [file 44318_2025_367_MOESM10_ESM.zip › SD figure 8/8D/Fig_8_D_Roi/CONTROL/CONTROL CU_Experiment-754_czi_63b690404e74d_hrm.ics.tiff]

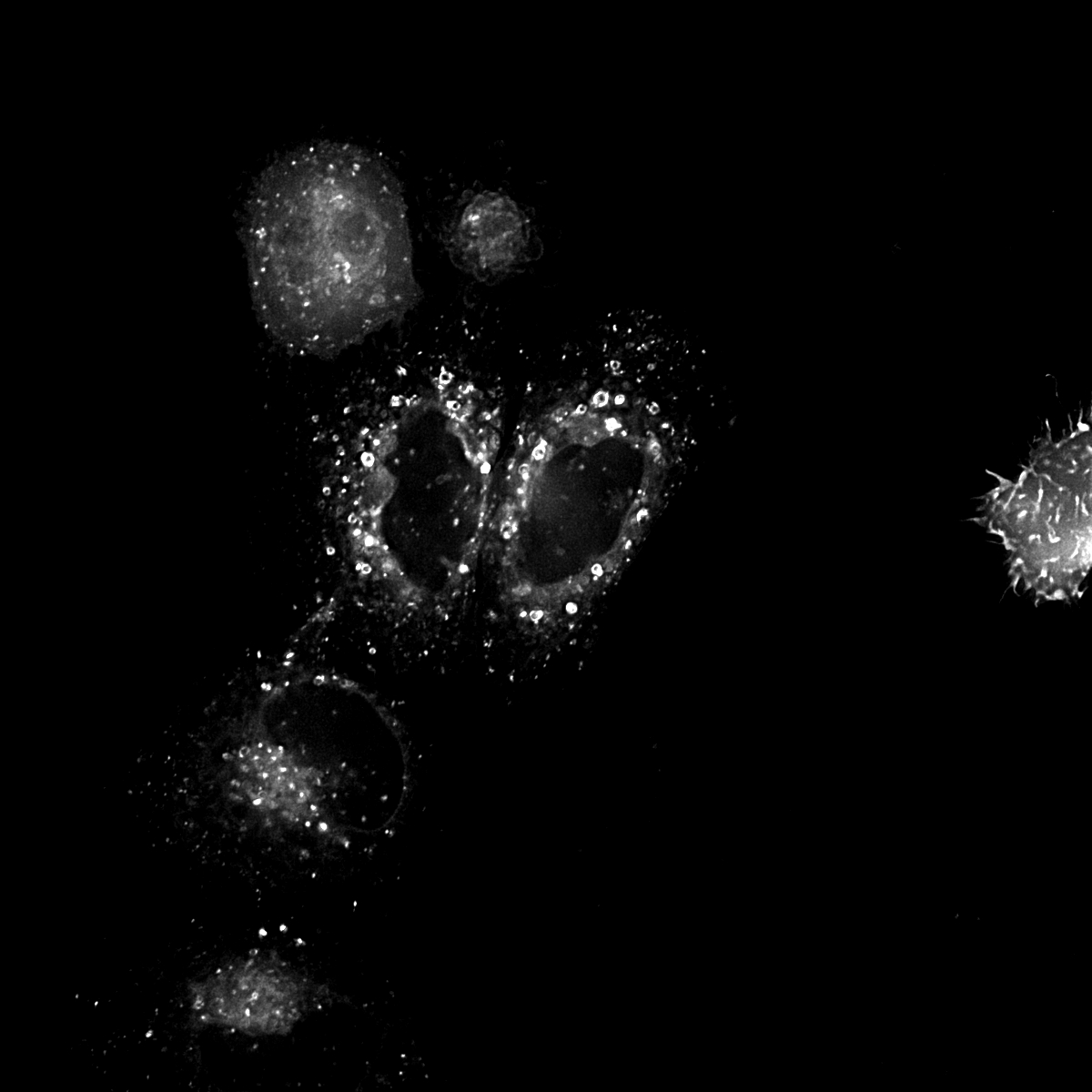

Supplement: Supplementary file 10 — Source data Fig. 8 [file 44318_2025_367_MOESM10_ESM.zip › SD figure 8/8D/Fig_8_D_data/CCZ1 KO/CCZ1 KO_Experiment-734_czi_63b690404947e_hrm.ics.tiff]

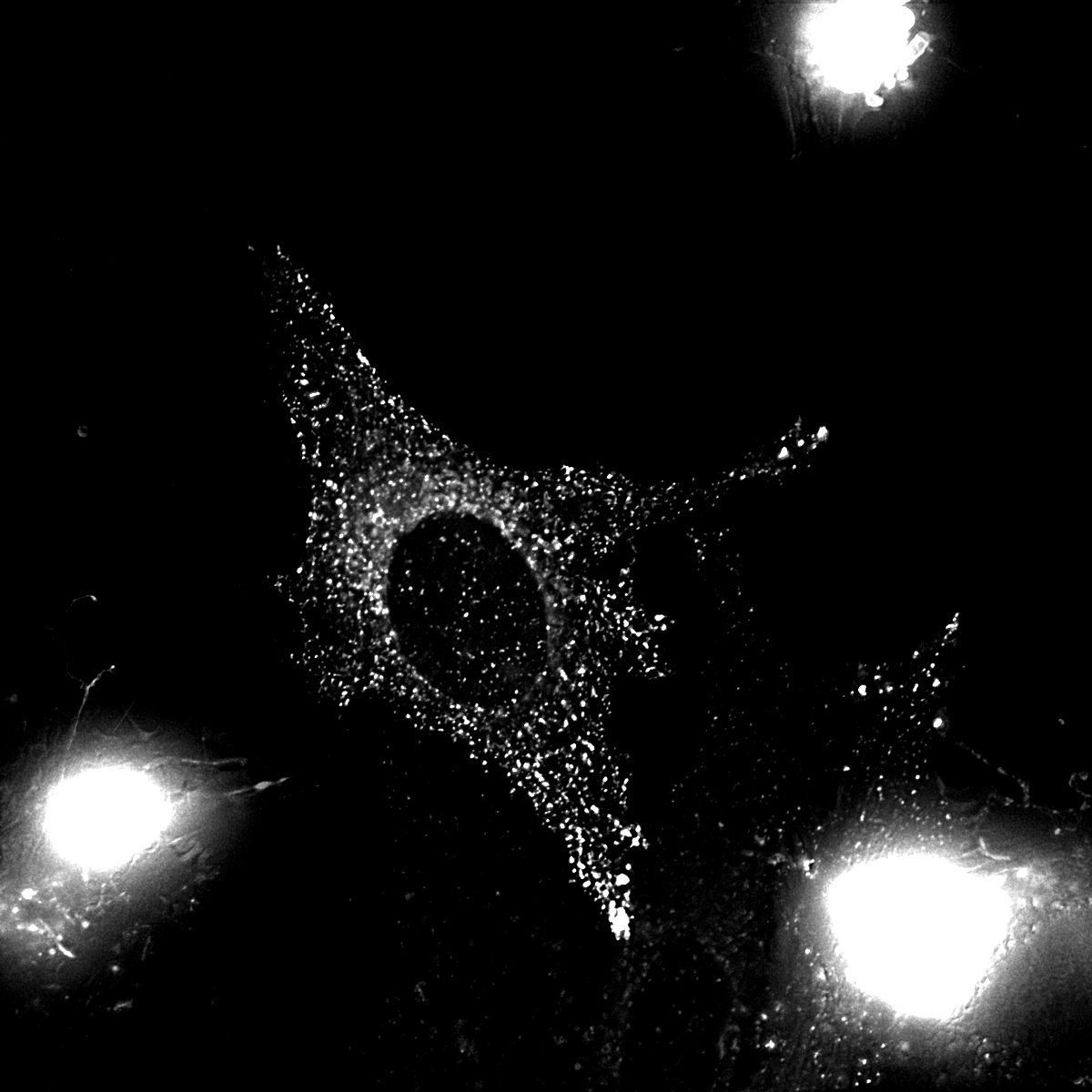

Supplement: Supplementary file 10 — Source data Fig. 8 [file 44318_2025_367_MOESM10_ESM.zip › SD figure 8/8D/Fig_8_D_data/CONTROL/CONTROL_Experiment-754_czi_63b690404e74d_hrm.ics.tiff]

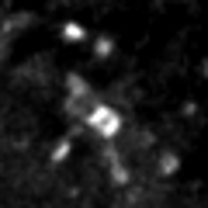

Supplement: Supplementary file 10 — Source data Fig. 8 [file 44318_2025_367_MOESM10_ESM.zip › SD figure 8/8E/Fig_8_E_Roi/CCZ1 KO/RAB7 CU_Experiment-782_czi_63b6908c06bb8_hrm.ics.tiff]

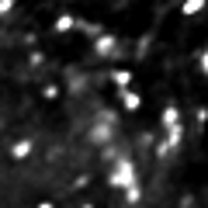

Supplement: Supplementary file 10 — Source data Fig. 8 [file 44318_2025_367_MOESM10_ESM.zip › SD figure 8/8E/Fig_8_E_Roi/CCZ1 KO/RAB5 CU_Experiment-782_czi_63b6908c06bb8_hrm.ics.tiff]

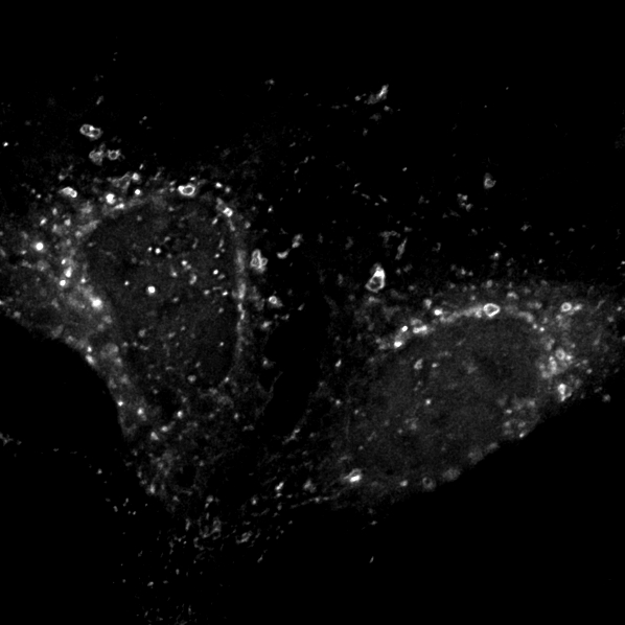

Supplement: Supplementary file 10 — Source data Fig. 8 [file 44318_2025_367_MOESM10_ESM.zip › SD figure 8/8E/Fig_8_E_Roi/CCZ1 KO/RAB7_Experiment-782_czi_63b6908c06bb8_hrm.ics.tiff]
