# Supplementary figures and images for: Coordination between ESCRT function and Rab conversion during endosome maturation (part 6 of 9)
Source: EMBO J. 2025 Feb 5;44(6):1574–607. doi: 10.1038/s44318-025-00367-7 (PMC11914609; doi:10.1038/s44318-025-00367-7)

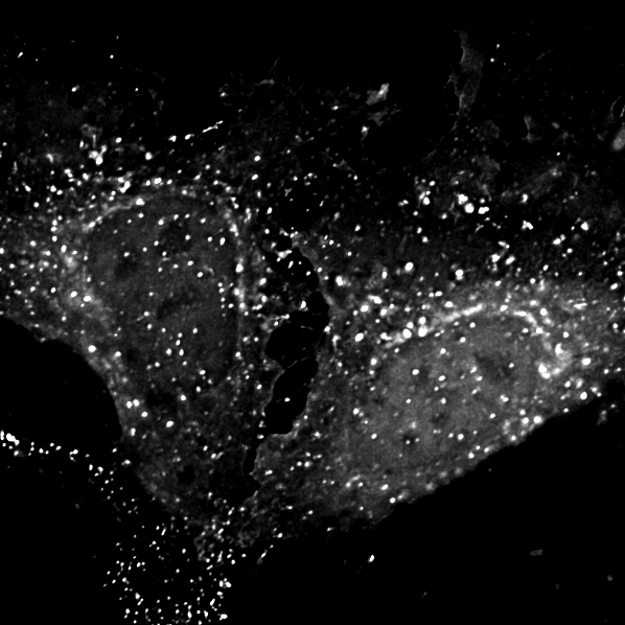

Supplement: Supplementary file 10 — Source data Fig. 8 [file 44318_2025_367_MOESM10_ESM.zip › SD figure 8/8E/Fig_8_E_Roi/CCZ1 KO/RAB5_Experiment-782_czi_63b6908c06bb8_hrm.ics.tiff]

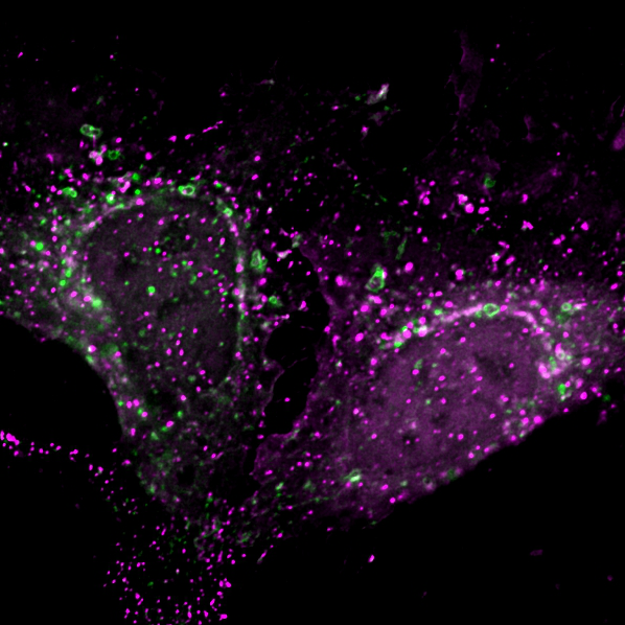

Supplement: Supplementary file 10 — Source data Fig. 8 [file 44318_2025_367_MOESM10_ESM.zip › SD figure 8/8E/Fig_8_E_Roi/CCZ1 KO/MERGED_Experiment-782_czi_63b6908c06bb8_hrm.ics.tiff]

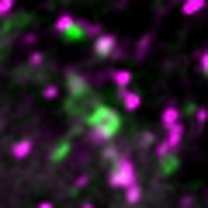

Supplement: Supplementary file 10 — Source data Fig. 8 [file 44318_2025_367_MOESM10_ESM.zip › SD figure 8/8E/Fig_8_E_Roi/CCZ1 KO/MERGED CU_Experiment-782_czi_63b6908c06bb8_hrm.ics.tiff]

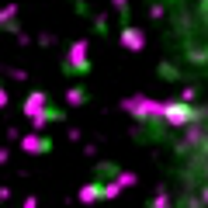

Supplement: Supplementary file 10 — Source data Fig. 8 [file 44318_2025_367_MOESM10_ESM.zip › SD figure 8/8E/Fig_8_E_Roi/CONTROL KO/MERGED CU_Experiment-798_czi_63b6908c0af1d_hrm.ics.tiff]

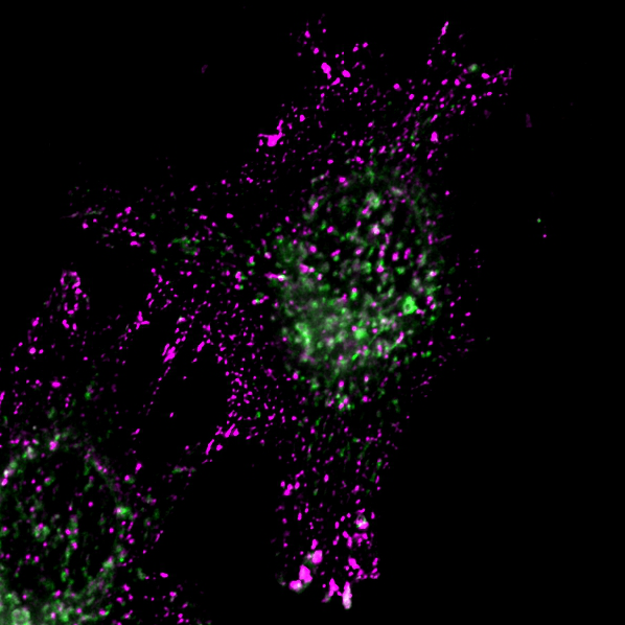

Supplement: Supplementary file 10 — Source data Fig. 8 [file 44318_2025_367_MOESM10_ESM.zip › SD figure 8/8E/Fig_8_E_Roi/CONTROL KO/MERGED_Experiment-798_czi_63b6908c0af1d_hrm.ics.tiff]

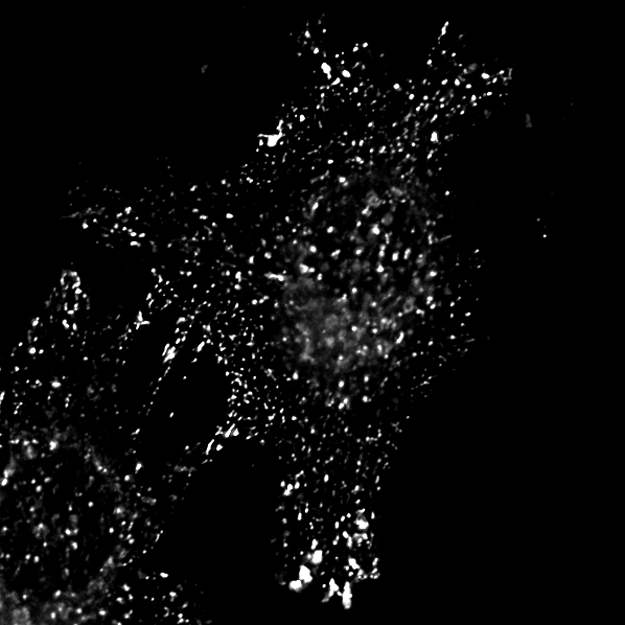

Supplement: Supplementary file 10 — Source data Fig. 8 [file 44318_2025_367_MOESM10_ESM.zip › SD figure 8/8E/Fig_8_E_Roi/CONTROL KO/RAB5_Experiment-798_czi_63b6908c0af1d_hrm.ics.tiff]

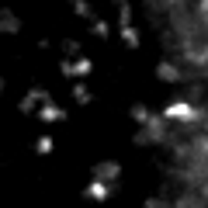

Supplement: Supplementary file 10 — Source data Fig. 8 [file 44318_2025_367_MOESM10_ESM.zip › SD figure 8/8E/Fig_8_E_Roi/CONTROL KO/RAB7 CU_Experiment-798_czi_63b6908c0af1d_hrm.ics.tiff]

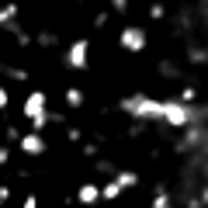

Supplement: Supplementary file 10 — Source data Fig. 8 [file 44318_2025_367_MOESM10_ESM.zip › SD figure 8/8E/Fig_8_E_Roi/CONTROL KO/RAB5 CU_Experiment-798_czi_63b6908c0af1d_hrm.ics.tiff]

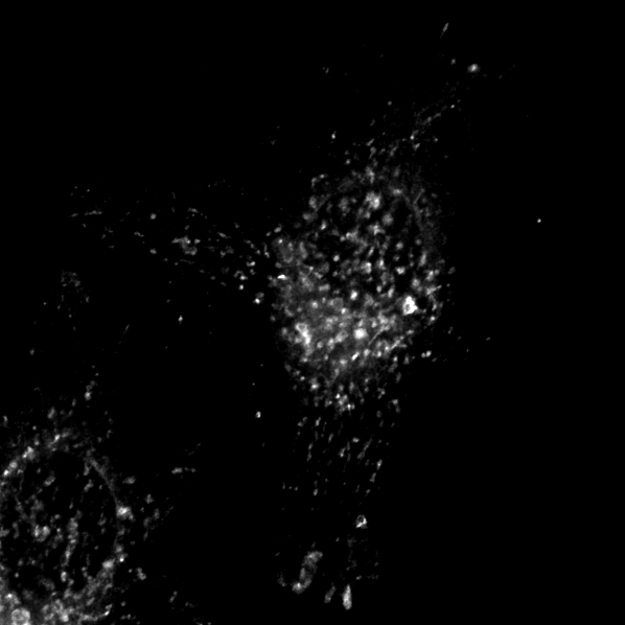

Supplement: Supplementary file 10 — Source data Fig. 8 [file 44318_2025_367_MOESM10_ESM.zip › SD figure 8/8E/Fig_8_E_Roi/CONTROL KO/RAB7_Experiment-798_czi_63b6908c0af1d_hrm.ics.tiff]

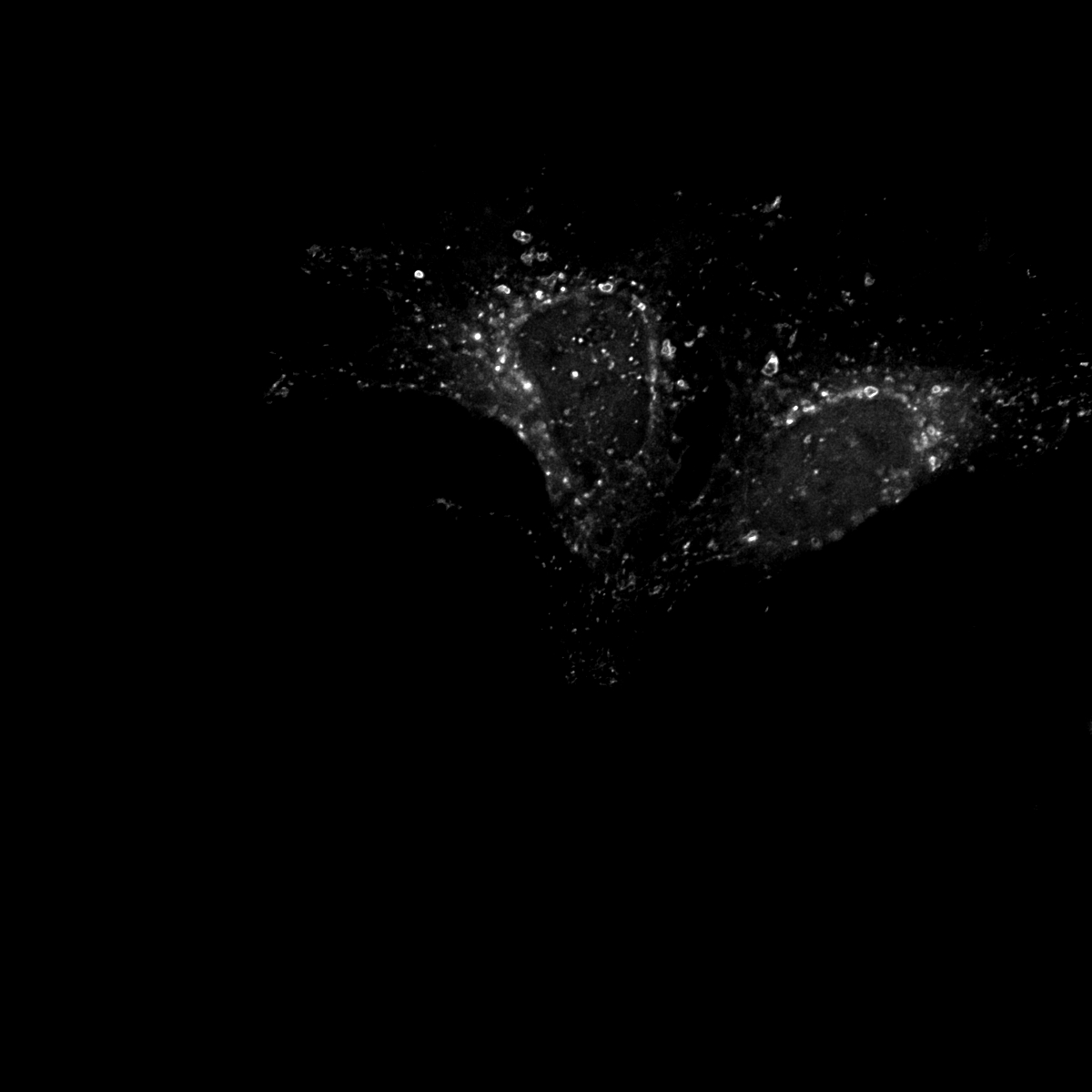

Supplement: Supplementary file 10 — Source data Fig. 8 [file 44318_2025_367_MOESM10_ESM.zip › SD figure 8/8E/Fig_8_E_data/CCZ1 KO/RAB7_Experiment-782_czi_63b6908c06bb8_hrm.ics.tiff]

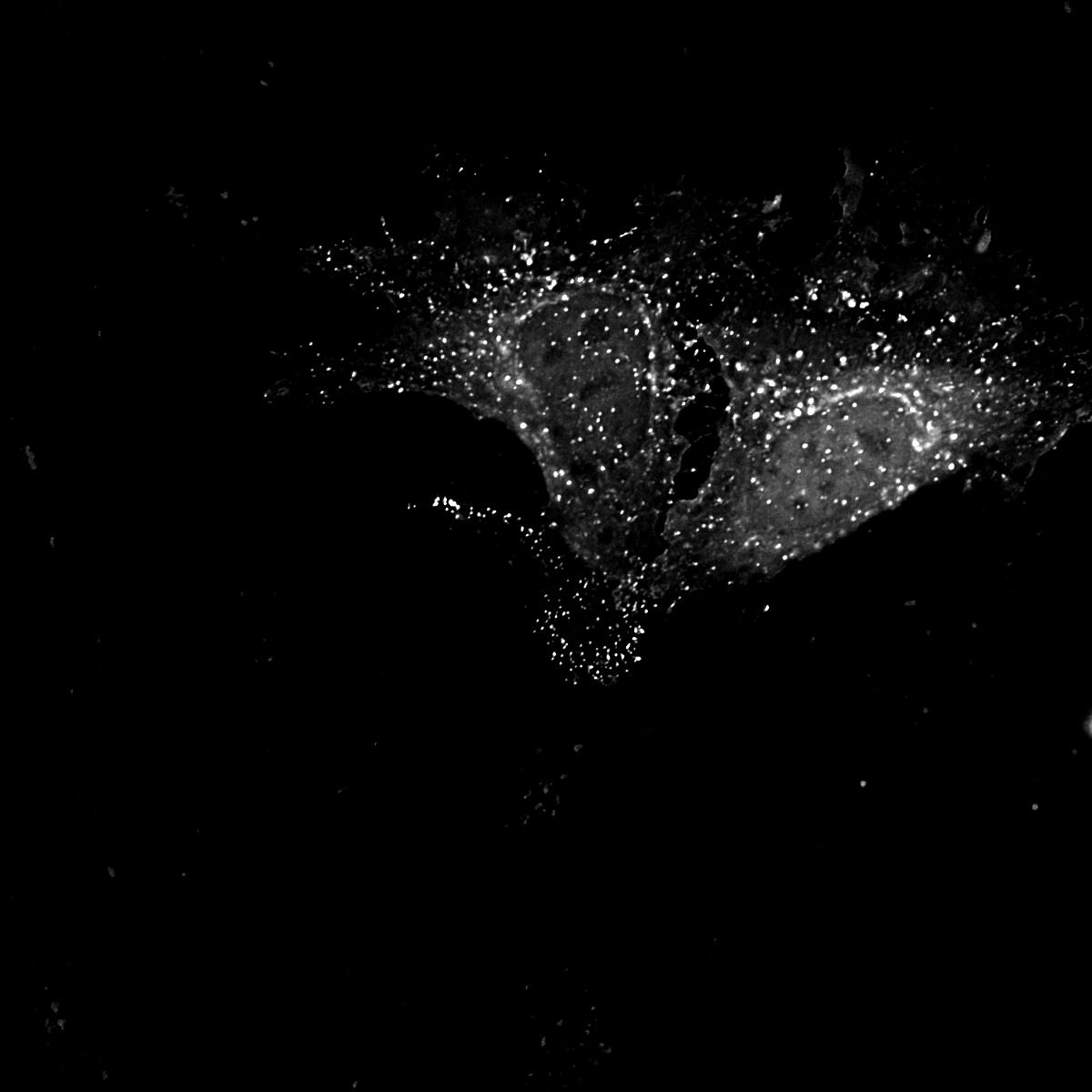

Supplement: Supplementary file 10 — Source data Fig. 8 [file 44318_2025_367_MOESM10_ESM.zip › SD figure 8/8E/Fig_8_E_data/CCZ1 KO/RAB5_Experiment-782_czi_63b6908c06bb8_hrm.ics.tiff]

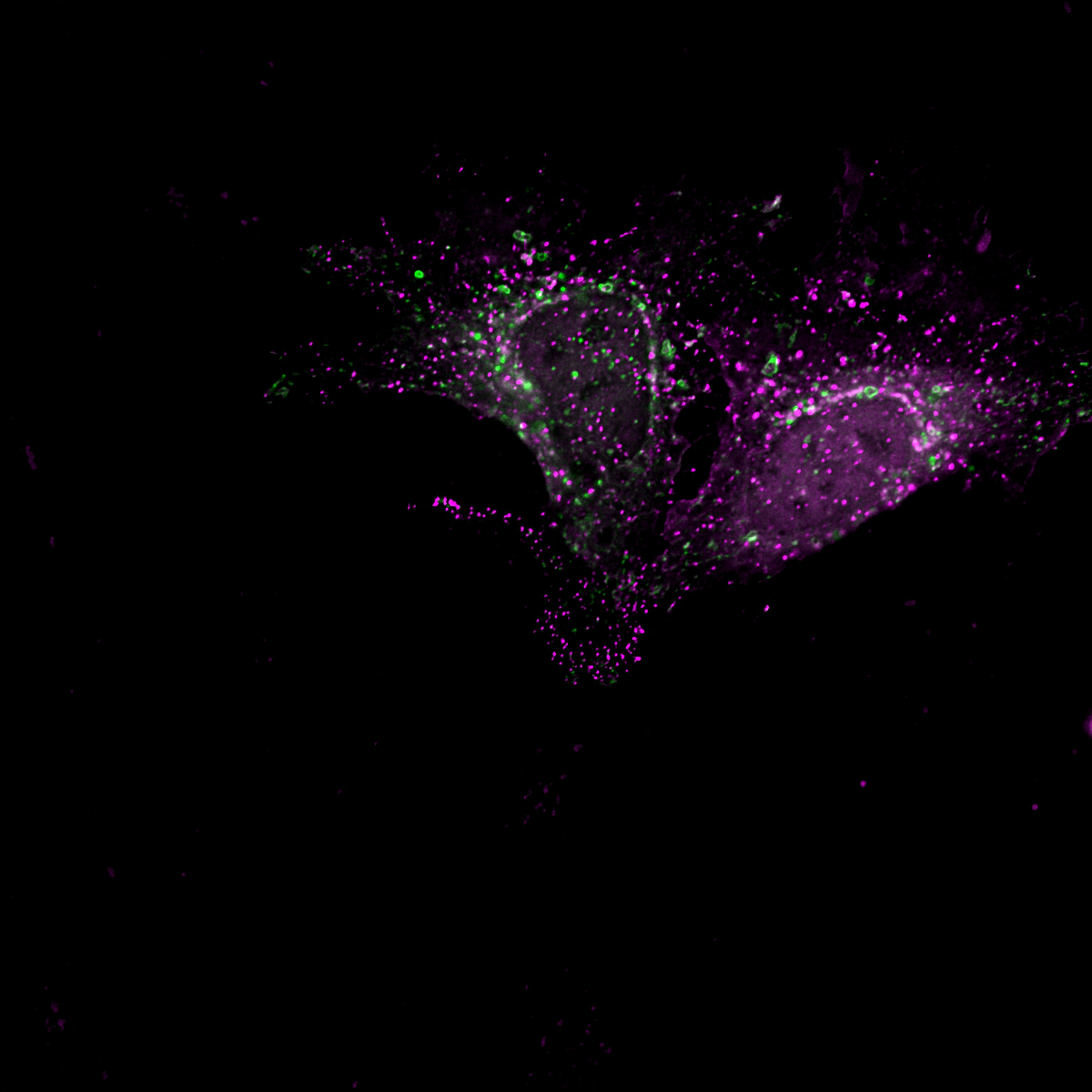

Supplement: Supplementary file 10 — Source data Fig. 8 [file 44318_2025_367_MOESM10_ESM.zip › SD figure 8/8E/Fig_8_E_data/CCZ1 KO/MERGED_Experiment-782_czi_63b6908c06bb8_hrm.ics.tiff]

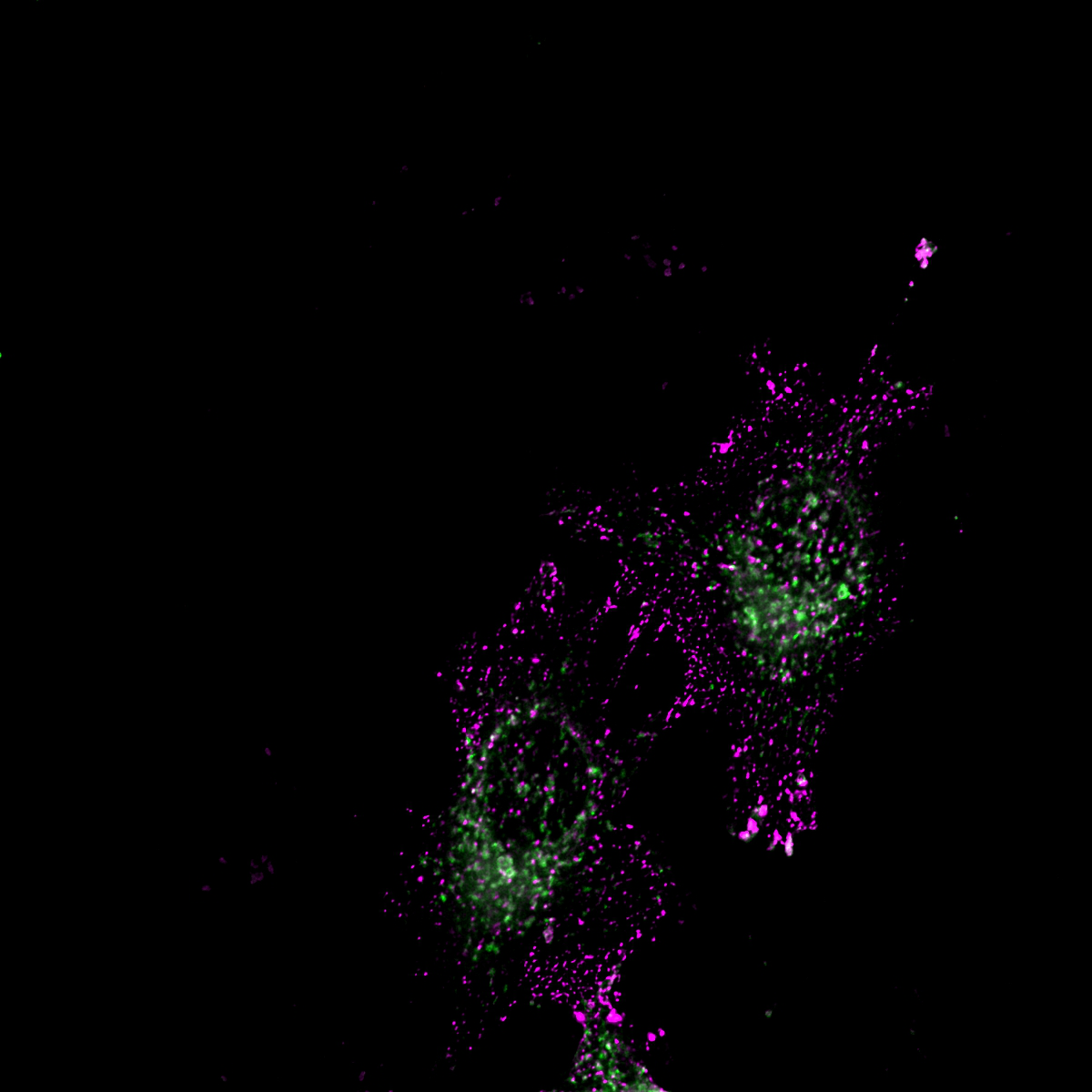

Supplement: Supplementary file 10 — Source data Fig. 8 [file 44318_2025_367_MOESM10_ESM.zip › SD figure 8/8E/Fig_8_E_data/CONTROL/MERGED_Experiment-798_czi_63b6908c0af1d_hrm.ics.tiff]

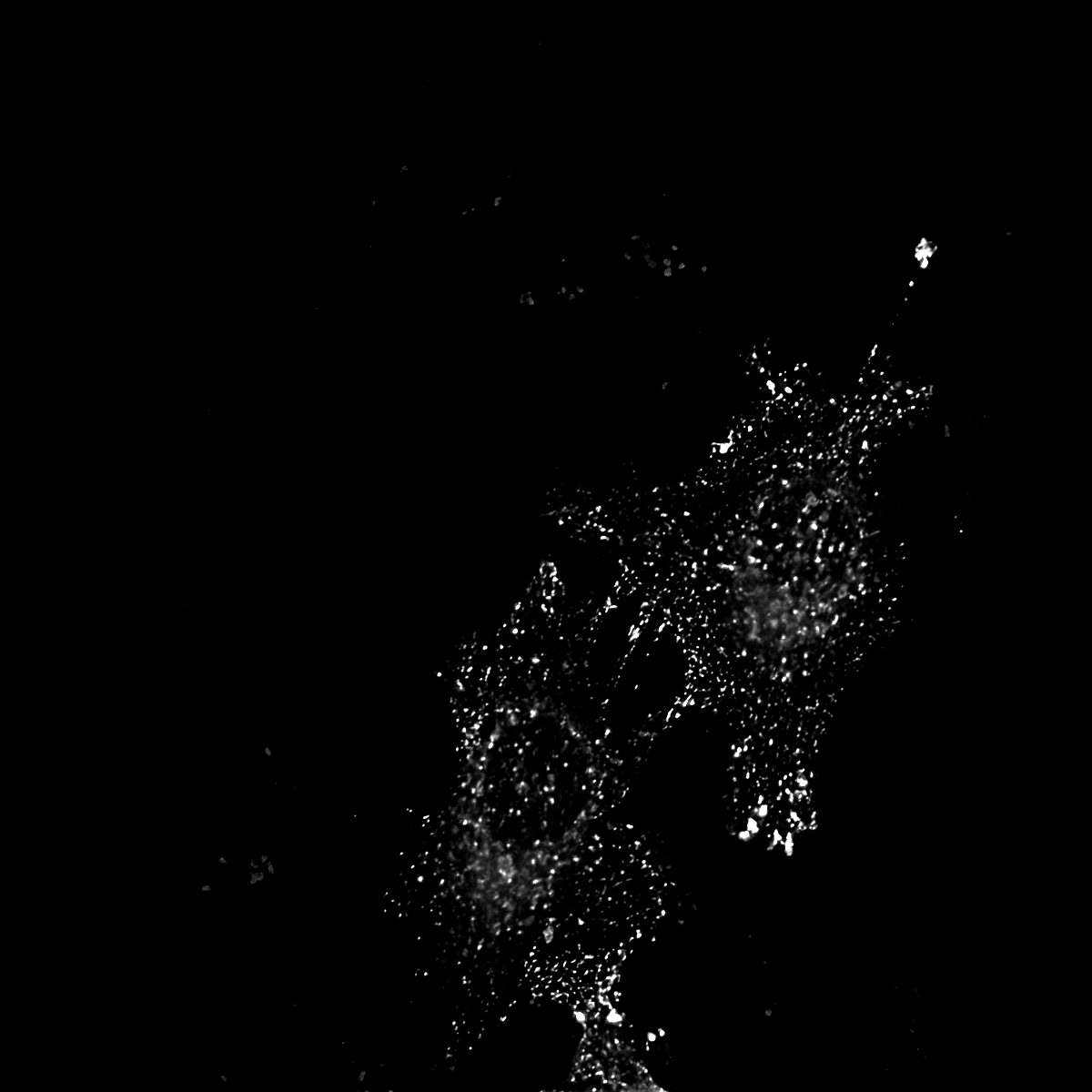

Supplement: Supplementary file 10 — Source data Fig. 8 [file 44318_2025_367_MOESM10_ESM.zip › SD figure 8/8E/Fig_8_E_data/CONTROL/RAB5_Experiment-798_czi_63b6908c0af1d_hrm.ics.tiff]

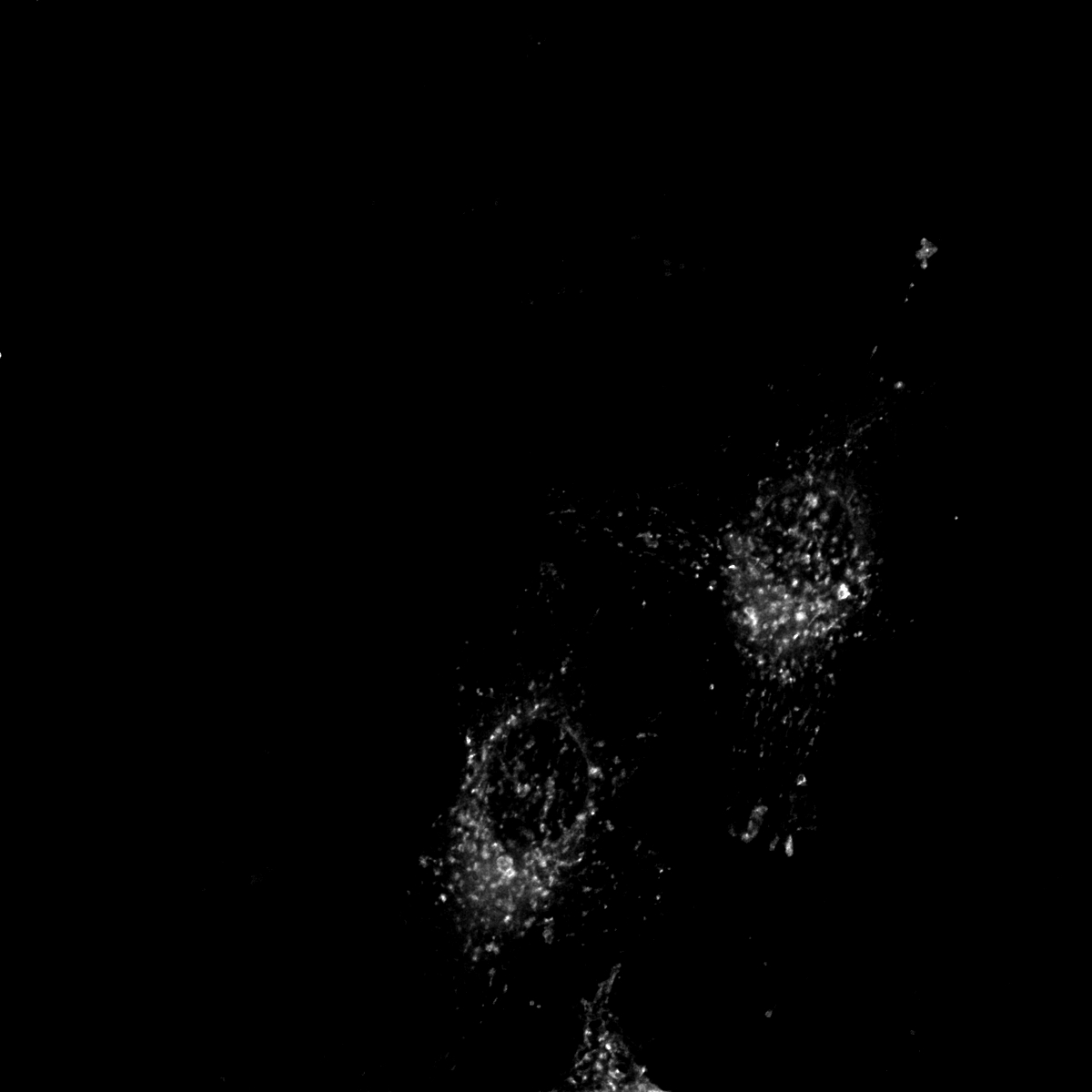

Supplement: Supplementary file 10 — Source data Fig. 8 [file 44318_2025_367_MOESM10_ESM.zip › SD figure 8/8E/Fig_8_E_data/CONTROL/RAB7_Experiment-798_czi_63b6908c0af1d_hrm.ics.tiff]

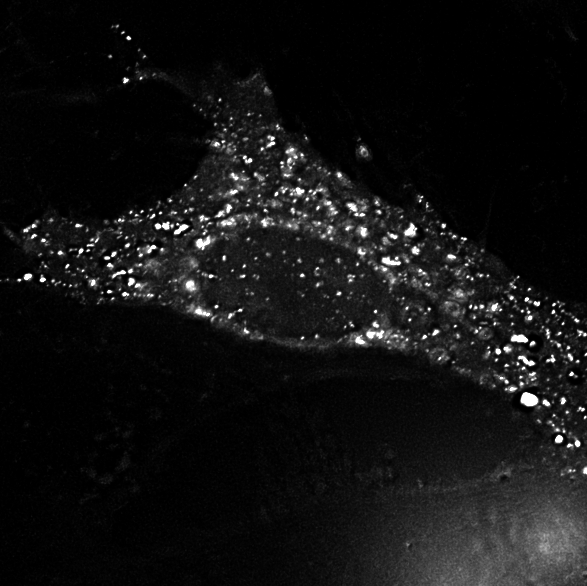

Supplement: Supplementary file 10 — Source data Fig. 8 [file 44318_2025_367_MOESM10_ESM.zip › SD figure 8/8H/Fig_8_H_Roi/CCZ1 KO/RAB5 + HRS/RAB5_Experiment-60_czi_63b6eeb0b9a2f_hrm.ics.tiff]

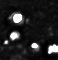

Supplement: Supplementary file 10 — Source data Fig. 8 [file 44318_2025_367_MOESM10_ESM.zip › SD figure 8/8H/Fig_8_H_Roi/CCZ1 KO/RAB5 + HRS/HRS CU_Experiment-60_czi_63b6eeb0b9a2f_hrm.ics.tiff]

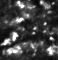

Supplement: Supplementary file 10 — Source data Fig. 8 [file 44318_2025_367_MOESM10_ESM.zip › SD figure 8/8H/Fig_8_H_Roi/CCZ1 KO/RAB5 + HRS/RAB5 CU_Experiment-60_czi_63b6eeb0b9a2f_hrm.ics.tiff]

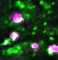

Supplement: Supplementary file 10 — Source data Fig. 8 [file 44318_2025_367_MOESM10_ESM.zip › SD figure 8/8H/Fig_8_H_Roi/CCZ1 KO/RAB5 + HRS/MERGED CU_Experiment-60_czi_63b6eeb0b9a2f_hrm.ics.tiff]

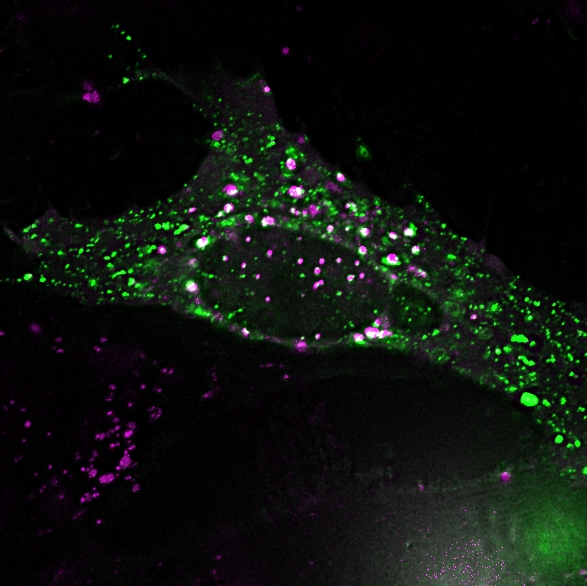

Supplement: Supplementary file 10 — Source data Fig. 8 [file 44318_2025_367_MOESM10_ESM.zip › SD figure 8/8H/Fig_8_H_Roi/CCZ1 KO/RAB5 + HRS/MERGED_Experiment-60_czi_63b6eeb0b9a2f_hrm.ics.tiff]

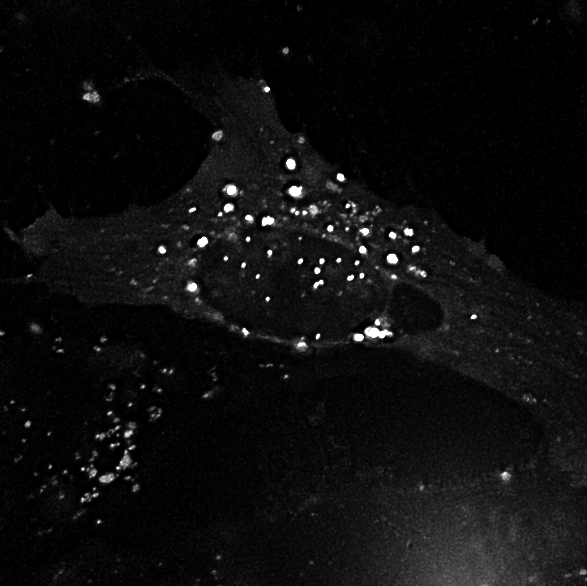

Supplement: Supplementary file 10 — Source data Fig. 8 [file 44318_2025_367_MOESM10_ESM.zip › SD figure 8/8H/Fig_8_H_Roi/CCZ1 KO/RAB5 + HRS/HRS_Experiment-60_czi_63b6eeb0b9a2f_hrm.ics.tiff]

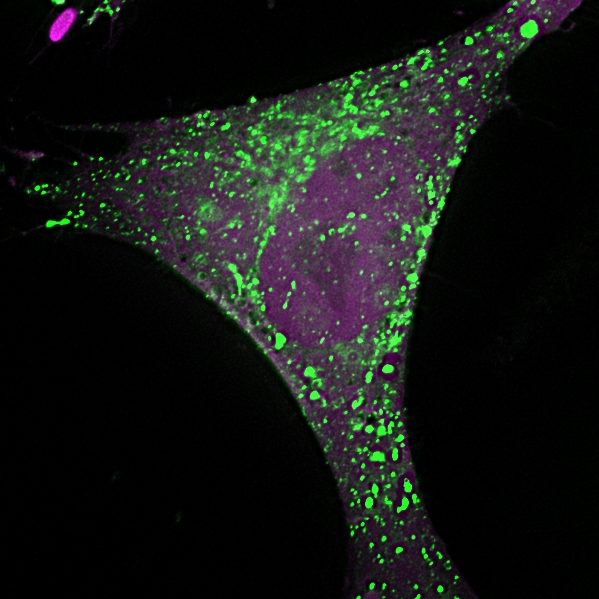

Supplement: Supplementary file 10 — Source data Fig. 8 [file 44318_2025_367_MOESM10_ESM.zip › SD figure 8/8H/Fig_8_H_Roi/CCZ1 KO/RAB5 + EV/MERGED_Experiment-21_czi_63b6eeb0b0283_hrm.ics.tiff]

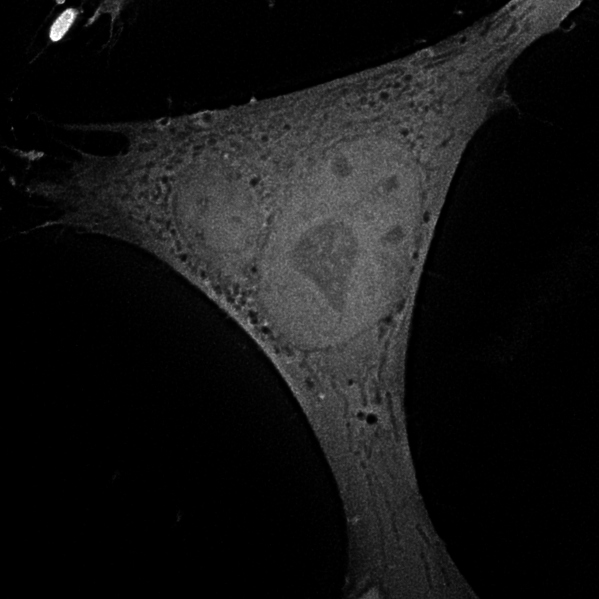

Supplement: Supplementary file 10 — Source data Fig. 8 [file 44318_2025_367_MOESM10_ESM.zip › SD figure 8/8H/Fig_8_H_Roi/CCZ1 KO/RAB5 + EV/EV_Experiment-21_czi_63b6eeb0b0283_hrm.ics.tiff]

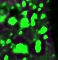

Supplement: Supplementary file 10 — Source data Fig. 8 [file 44318_2025_367_MOESM10_ESM.zip › SD figure 8/8H/Fig_8_H_Roi/CCZ1 KO/RAB5 + EV/MERGED CU_Experiment-21_czi_63b6eeb0b0283_hrm.ics.tiff]

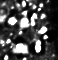

Supplement: Supplementary file 10 — Source data Fig. 8 [file 44318_2025_367_MOESM10_ESM.zip › SD figure 8/8H/Fig_8_H_Roi/CCZ1 KO/RAB5 + EV/RAB5 CU_Experiment-21_czi_63b6eeb0b0283_hrm.ics.tiff]

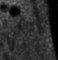

Supplement: Supplementary file 10 — Source data Fig. 8 [file 44318_2025_367_MOESM10_ESM.zip › SD figure 8/8H/Fig_8_H_Roi/CCZ1 KO/RAB5 + EV/EV CU_Experiment-21_czi_63b6eeb0b0283_hrm.ics.tiff]

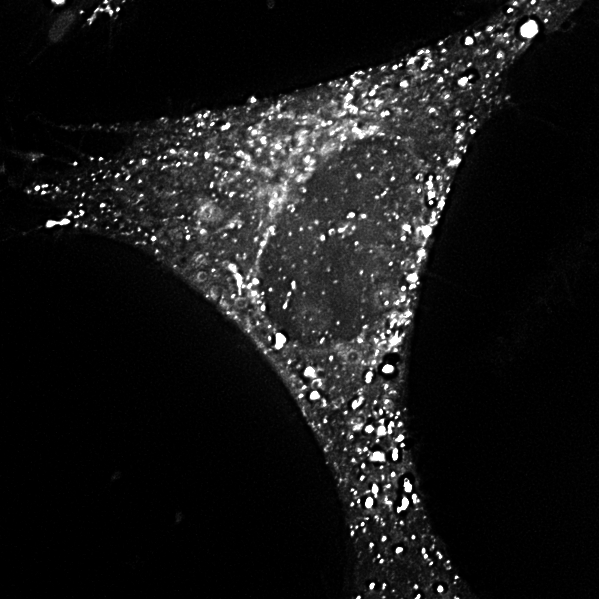

Supplement: Supplementary file 10 — Source data Fig. 8 [file 44318_2025_367_MOESM10_ESM.zip › SD figure 8/8H/Fig_8_H_Roi/CCZ1 KO/RAB5 + EV/RAB5_Experiment-21_czi_63b6eeb0b0283_hrm.ics.tiff]

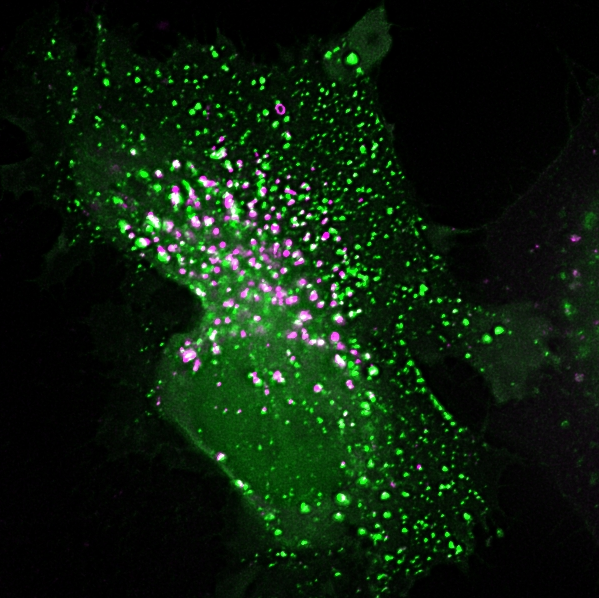

Supplement: Supplementary file 10 — Source data Fig. 8 [file 44318_2025_367_MOESM10_ESM.zip › SD figure 8/8H/Fig_8_H_Roi/CONTROL KO/RAB5 + HRS/MERGED_Experiment-53_czi_63b6eeb0b786b_hrm.ics.tiff]

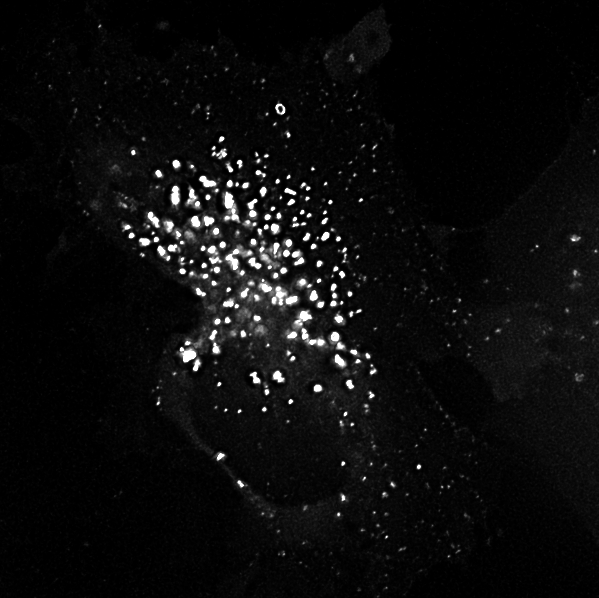

Supplement: Supplementary file 10 — Source data Fig. 8 [file 44318_2025_367_MOESM10_ESM.zip › SD figure 8/8H/Fig_8_H_Roi/CONTROL KO/RAB5 + HRS/HRS_Experiment-53_czi_63b6eeb0b786b_hrm.ics.tiff]

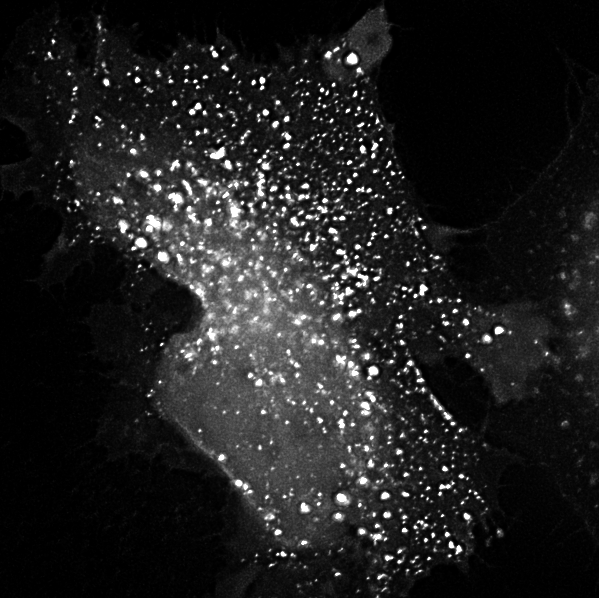

Supplement: Supplementary file 10 — Source data Fig. 8 [file 44318_2025_367_MOESM10_ESM.zip › SD figure 8/8H/Fig_8_H_Roi/CONTROL KO/RAB5 + HRS/RAB5_Experiment-53_czi_63b6eeb0b786b_hrm.ics.tiff]

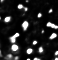

Supplement: Supplementary file 10 — Source data Fig. 8 [file 44318_2025_367_MOESM10_ESM.zip › SD figure 8/8H/Fig_8_H_Roi/CONTROL KO/RAB5 + HRS/HRS CU_Experiment-53_czi_63b6eeb0b786b_hrm.ics.tiff]

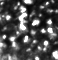

Supplement: Supplementary file 10 — Source data Fig. 8 [file 44318_2025_367_MOESM10_ESM.zip › SD figure 8/8H/Fig_8_H_Roi/CONTROL KO/RAB5 + HRS/RAB5 CU_Experiment-53_czi_63b6eeb0b786b_hrm.ics.tiff]

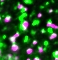

Supplement: Supplementary file 10 — Source data Fig. 8 [file 44318_2025_367_MOESM10_ESM.zip › SD figure 8/8H/Fig_8_H_Roi/CONTROL KO/RAB5 + HRS/MERGED CU_Experiment-53_czi_63b6eeb0b786b_hrm.ics.tiff]

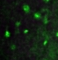

Supplement: Supplementary file 10 — Source data Fig. 8 [file 44318_2025_367_MOESM10_ESM.zip › SD figure 8/8H/Fig_8_H_Roi/CONTROL KO/RAB5 + EV/MERGED CU_Experiment-16_czi_63b6eeb0af22f_hrm.ics.tiff]

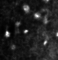

Supplement: Supplementary file 10 — Source data Fig. 8 [file 44318_2025_367_MOESM10_ESM.zip › SD figure 8/8H/Fig_8_H_Roi/CONTROL KO/RAB5 + EV/RAB5 CU_Experiment-16_czi_63b6eeb0af22f_hrm.ics.tiff]

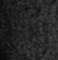

Supplement: Supplementary file 10 — Source data Fig. 8 [file 44318_2025_367_MOESM10_ESM.zip › SD figure 8/8H/Fig_8_H_Roi/CONTROL KO/RAB5 + EV/EV CU_Experiment-16_czi_63b6eeb0af22f_hrm.ics.tiff]

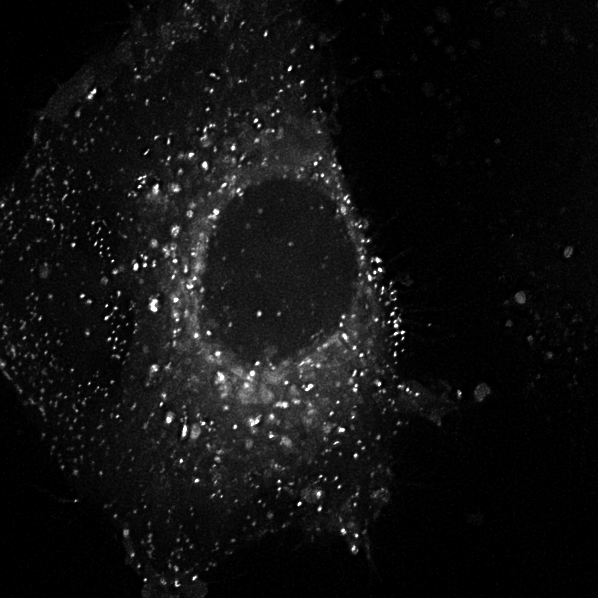

Supplement: Supplementary file 10 — Source data Fig. 8 [file 44318_2025_367_MOESM10_ESM.zip › SD figure 8/8H/Fig_8_H_Roi/CONTROL KO/RAB5 + EV/RAB5_Experiment-16_czi_63b6eeb0af22f_hrm.ics.tiff]

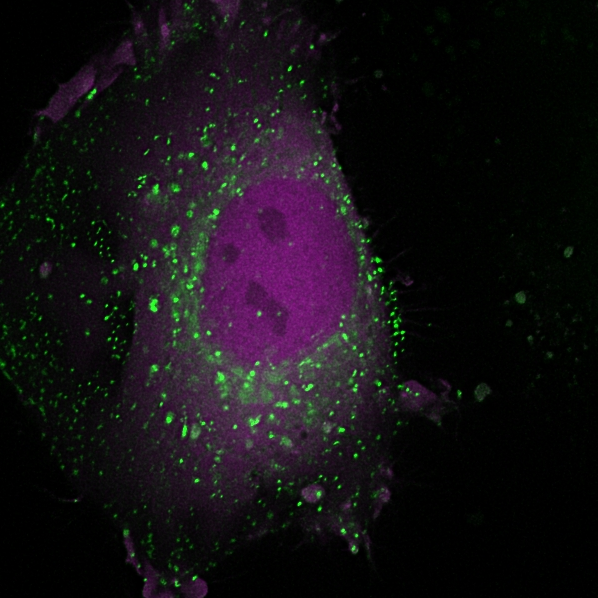

Supplement: Supplementary file 10 — Source data Fig. 8 [file 44318_2025_367_MOESM10_ESM.zip › SD figure 8/8H/Fig_8_H_Roi/CONTROL KO/RAB5 + EV/MERGED_Experiment-16_czi_63b6eeb0af22f_hrm.ics.tiff]

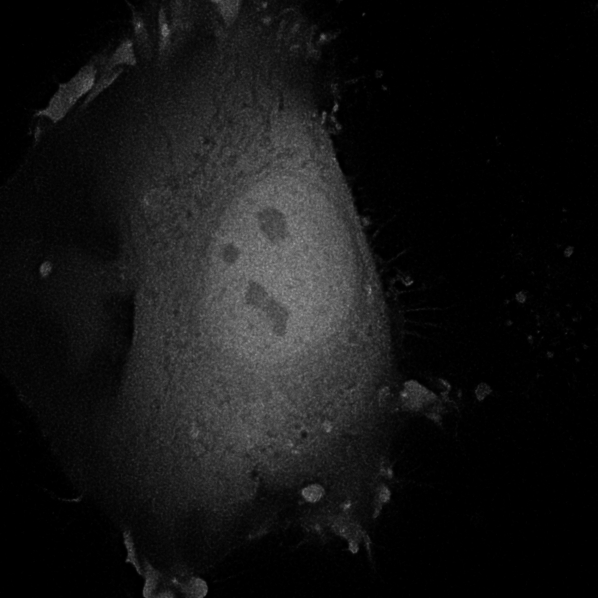

Supplement: Supplementary file 10 — Source data Fig. 8 [file 44318_2025_367_MOESM10_ESM.zip › SD figure 8/8H/Fig_8_H_Roi/CONTROL KO/RAB5 + EV/EV_Experiment-16_czi_63b6eeb0af22f_hrm.ics.tiff]

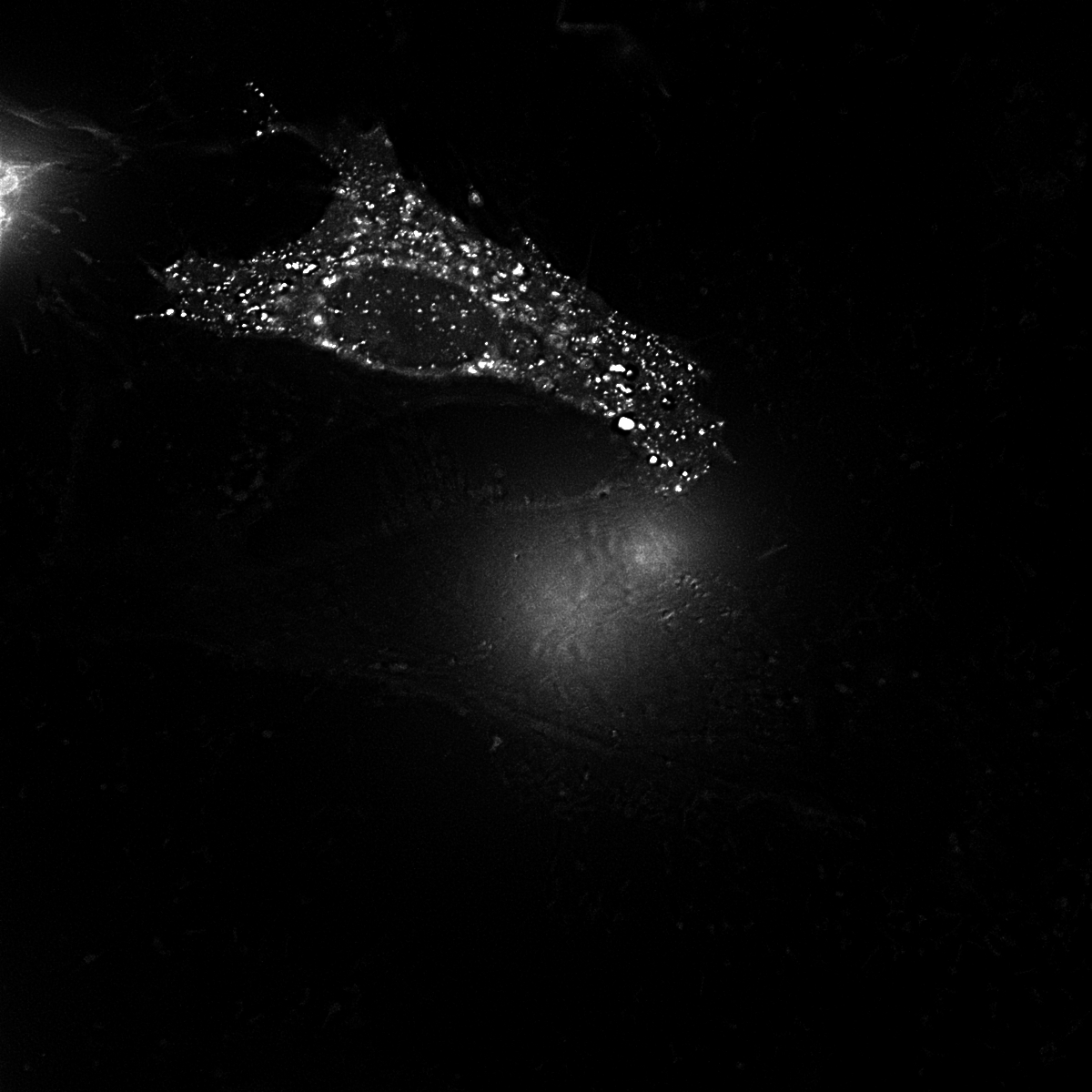

Supplement: Supplementary file 10 — Source data Fig. 8 [file 44318_2025_367_MOESM10_ESM.zip › SD figure 8/8H/Fig_8_H_data/CCZ1 KO/RAB5 + HRS/RAB5_Experiment-60_czi_63b6eeb0b9a2f_hrm.ics.tiff]

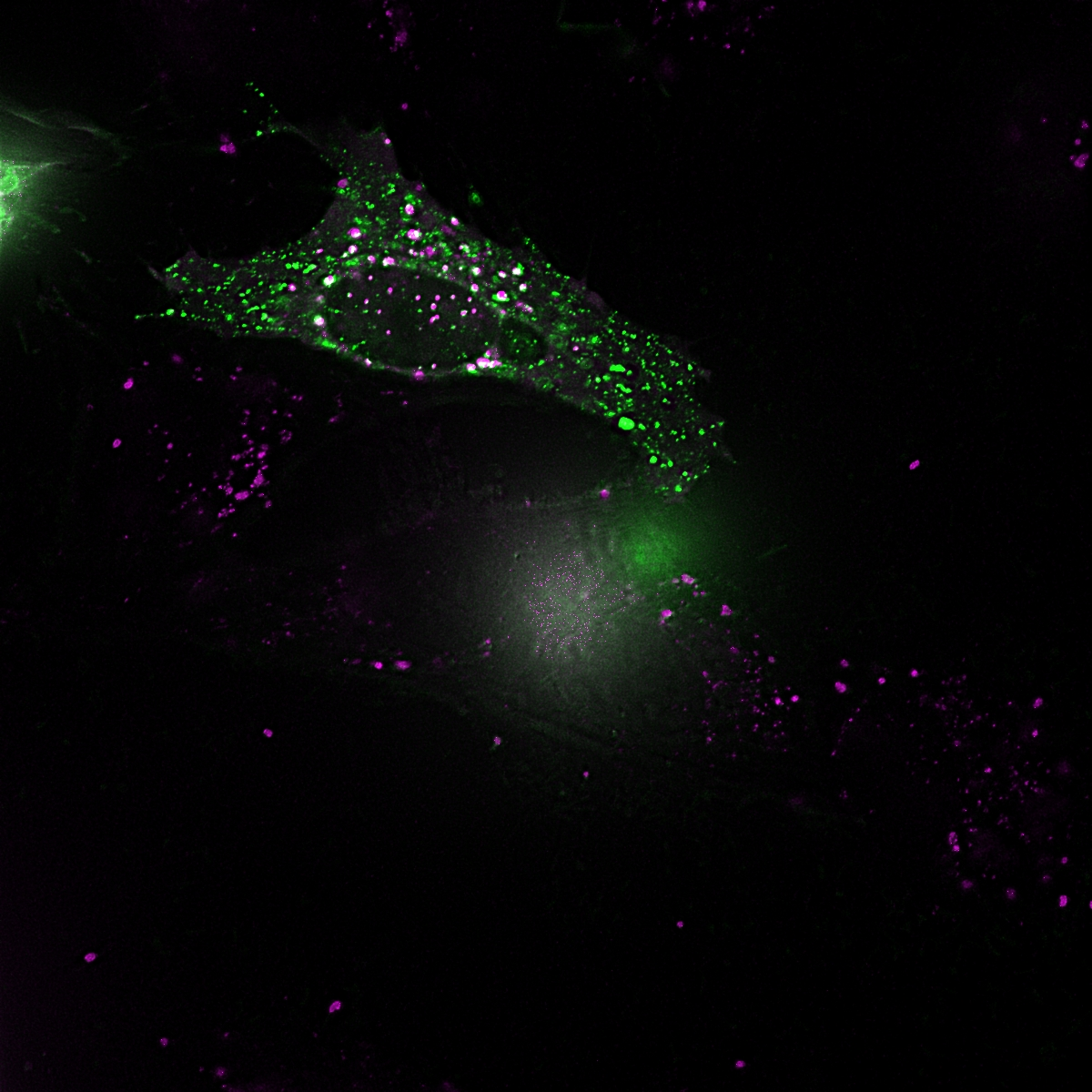

Supplement: Supplementary file 10 — Source data Fig. 8 [file 44318_2025_367_MOESM10_ESM.zip › SD figure 8/8H/Fig_8_H_data/CCZ1 KO/RAB5 + HRS/MERGED_Experiment-60_czi_63b6eeb0b9a2f_hrm.ics.tiff]

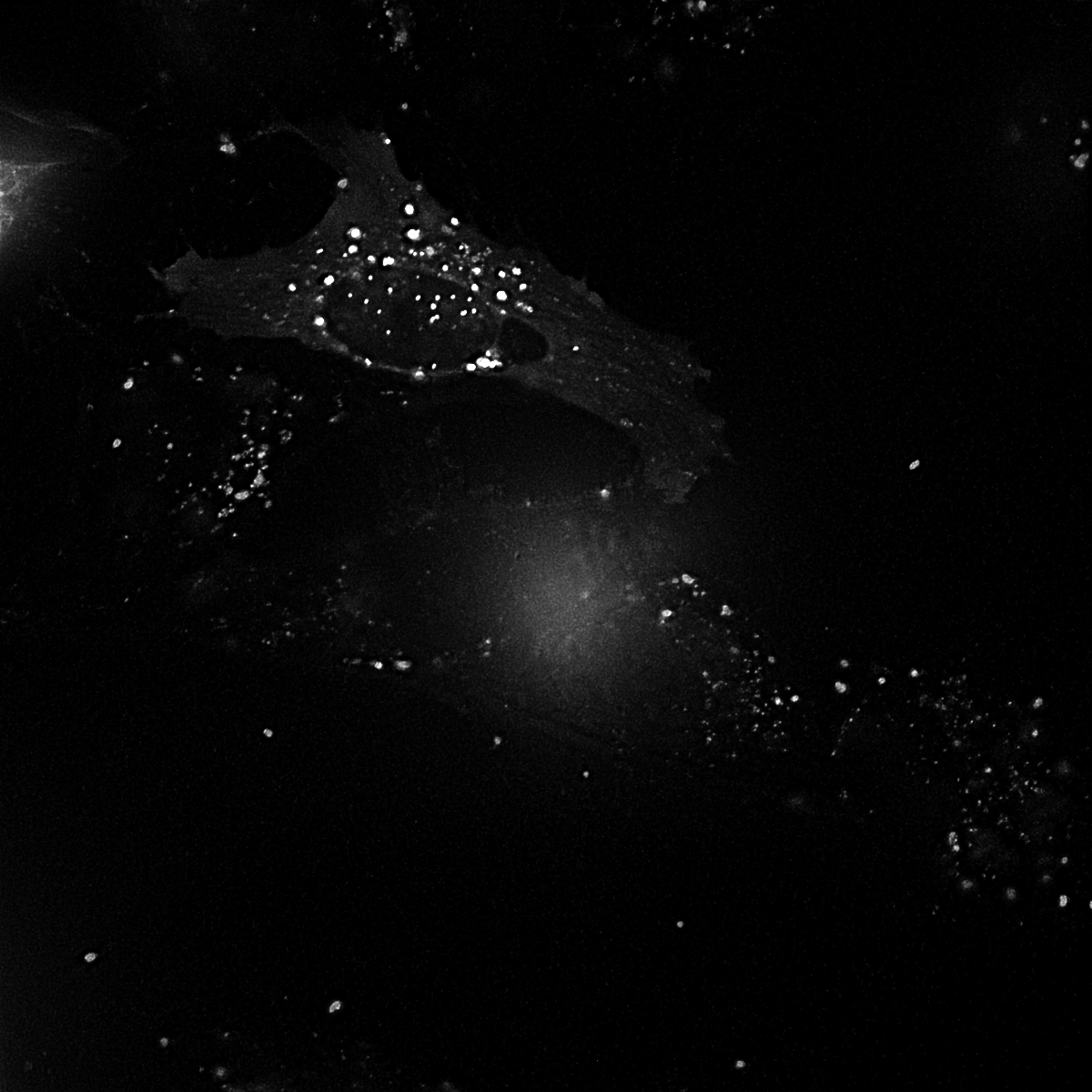

Supplement: Supplementary file 10 — Source data Fig. 8 [file 44318_2025_367_MOESM10_ESM.zip › SD figure 8/8H/Fig_8_H_data/CCZ1 KO/RAB5 + HRS/HRS_Experiment-60_czi_63b6eeb0b9a2f_hrm.ics.tiff]

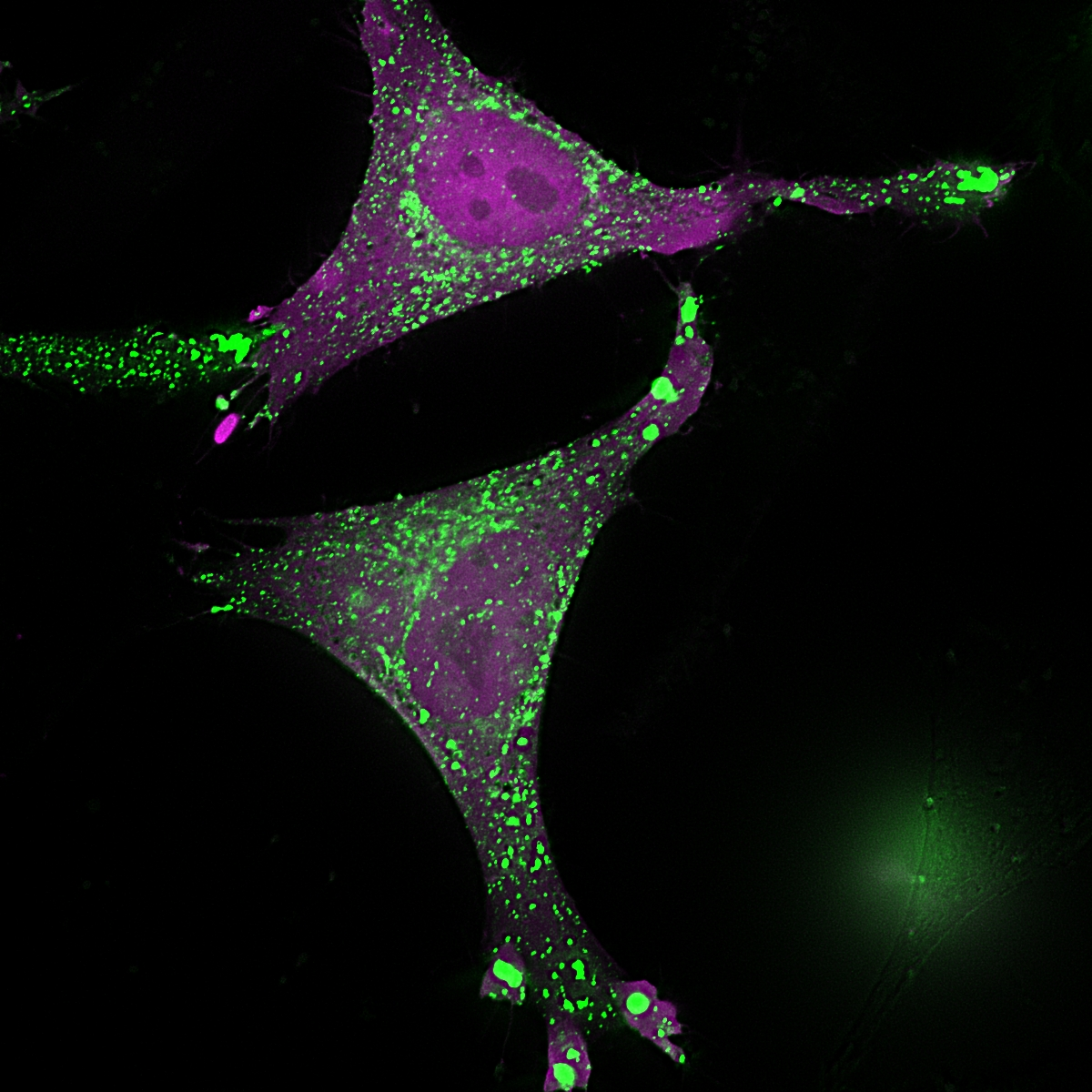

Supplement: Supplementary file 10 — Source data Fig. 8 [file 44318_2025_367_MOESM10_ESM.zip › SD figure 8/8H/Fig_8_H_data/CCZ1 KO/RAB5 + EV/MERGED_Experiment-21_czi_63b6eeb0b0283_hrm.ics.tiff]

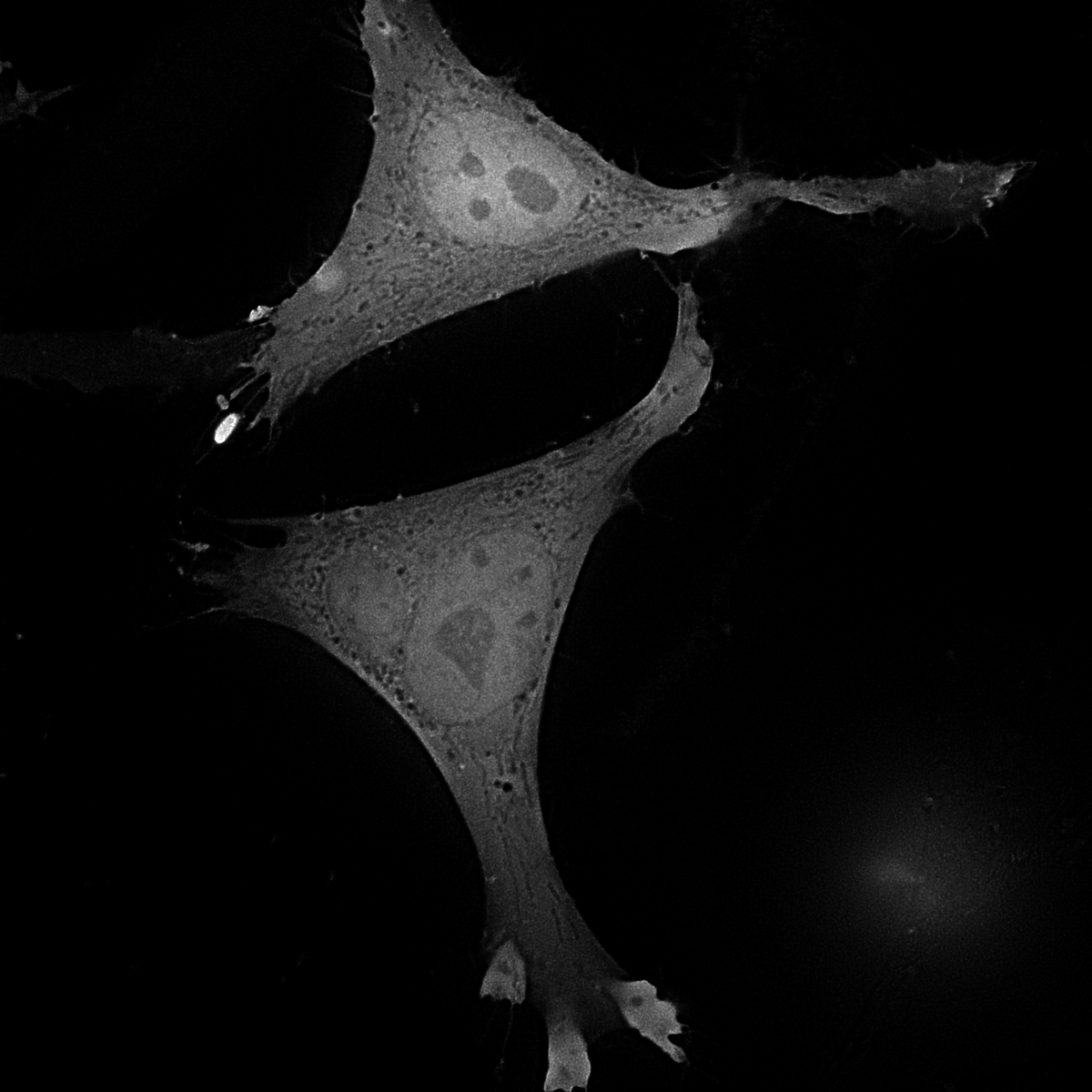

Supplement: Supplementary file 10 — Source data Fig. 8 [file 44318_2025_367_MOESM10_ESM.zip › SD figure 8/8H/Fig_8_H_data/CCZ1 KO/RAB5 + EV/EV_Experiment-21_czi_63b6eeb0b0283_hrm.ics.tiff]

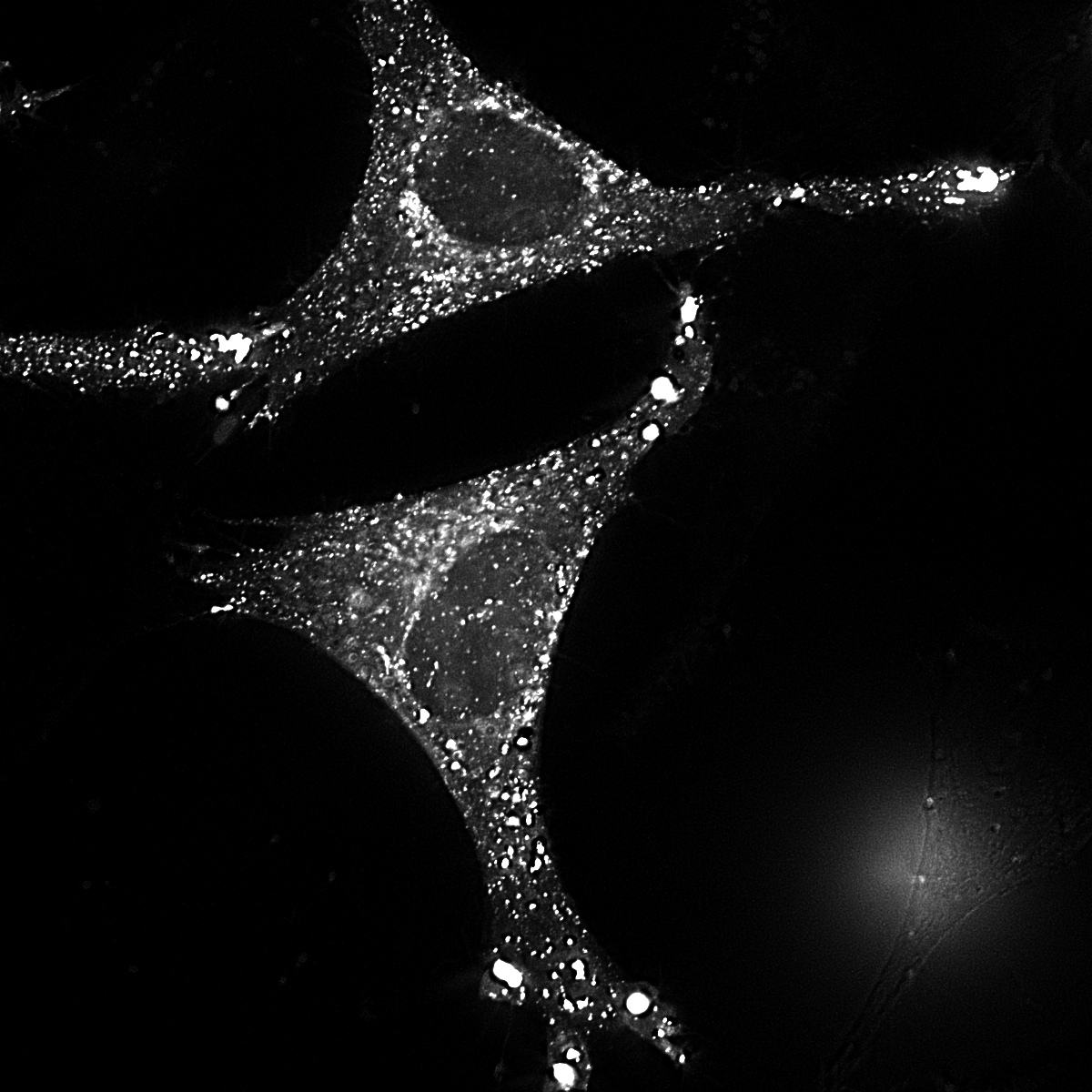

Supplement: Supplementary file 10 — Source data Fig. 8 [file 44318_2025_367_MOESM10_ESM.zip › SD figure 8/8H/Fig_8_H_data/CCZ1 KO/RAB5 + EV/RAB5_Experiment-21_czi_63b6eeb0b0283_hrm.ics.tiff]

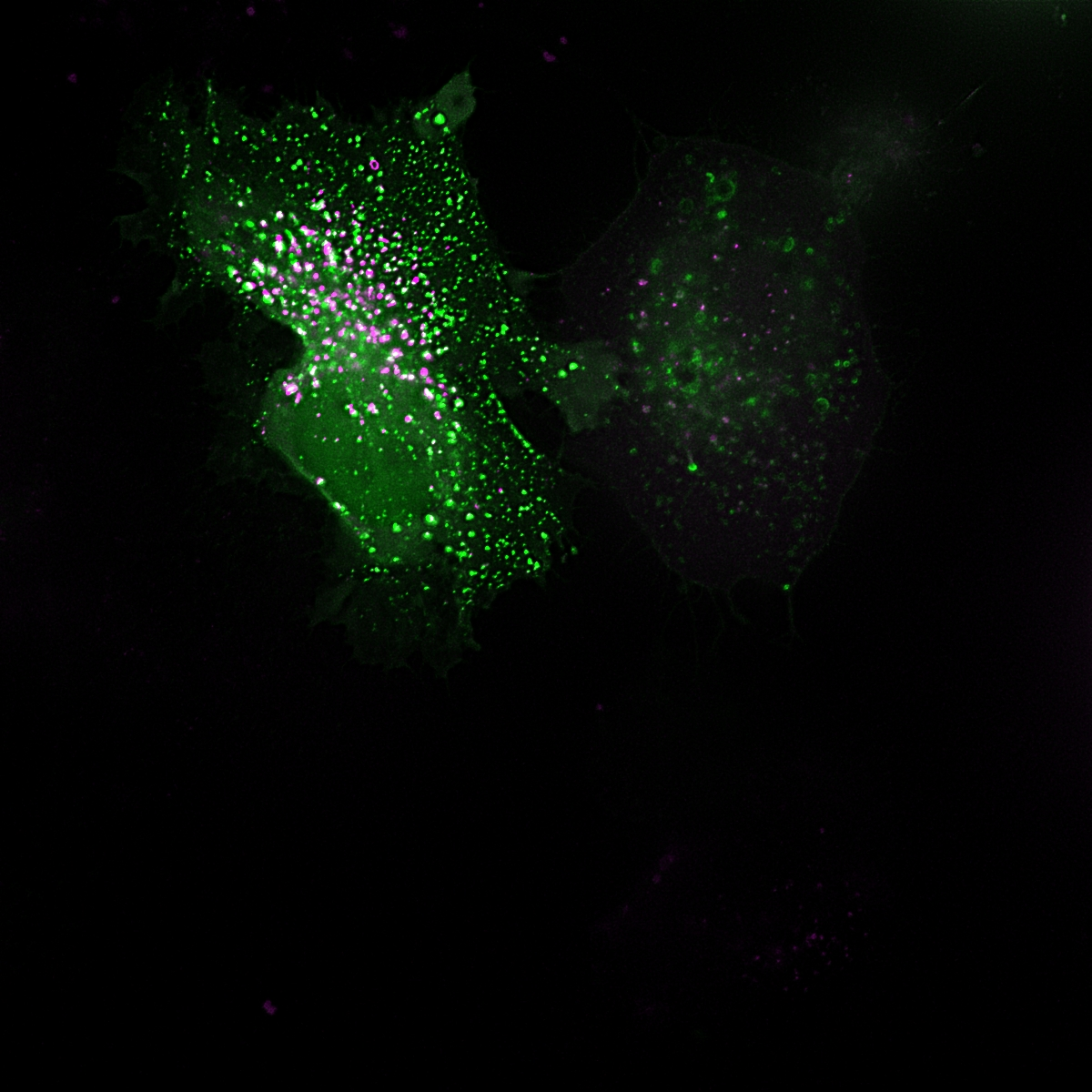

Supplement: Supplementary file 10 — Source data Fig. 8 [file 44318_2025_367_MOESM10_ESM.zip › SD figure 8/8H/Fig_8_H_data/CONTROL KO/RAB5 + HRS/MERGED_Experiment-53_czi_63b6eeb0b786b_hrm.ics.tiff]

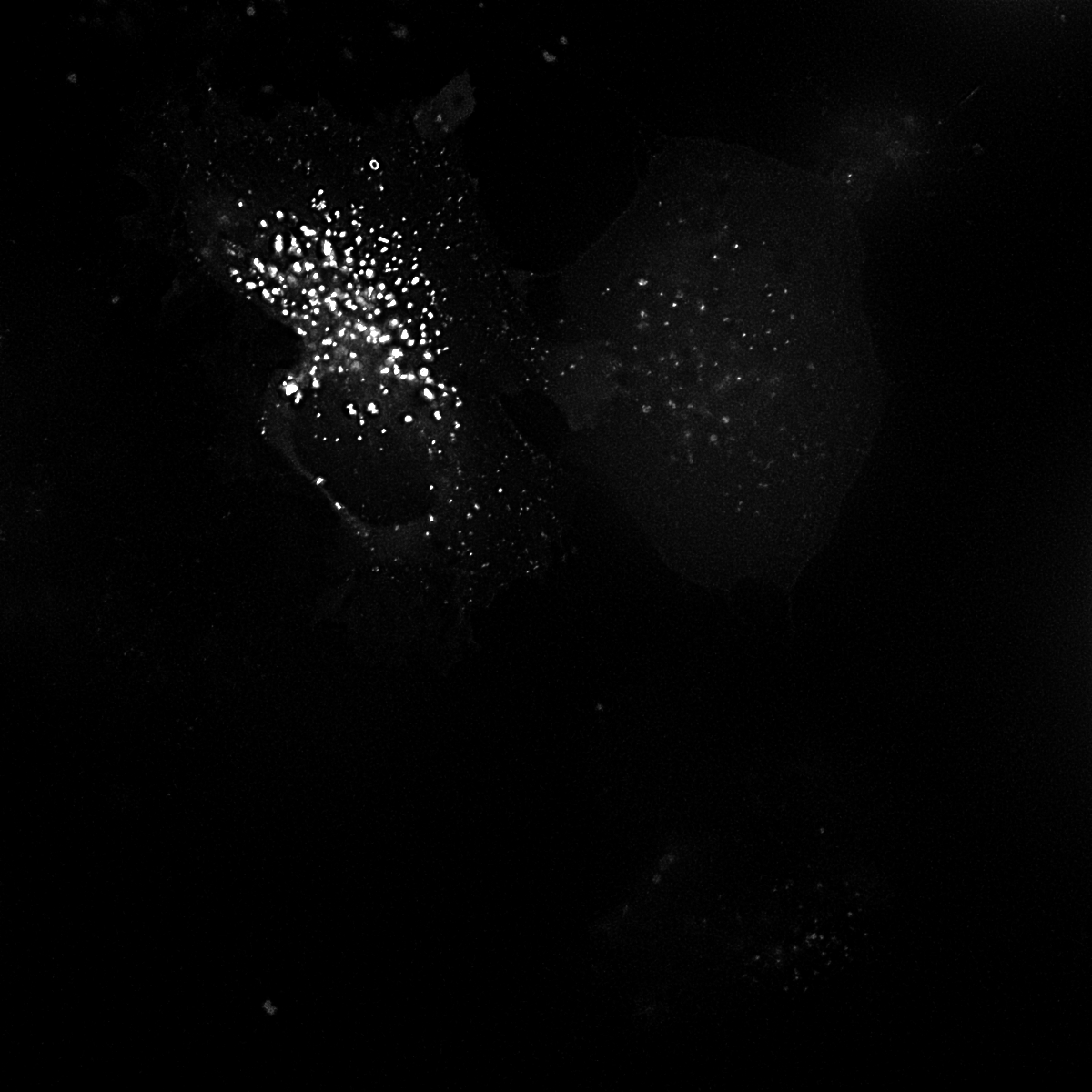

Supplement: Supplementary file 10 — Source data Fig. 8 [file 44318_2025_367_MOESM10_ESM.zip › SD figure 8/8H/Fig_8_H_data/CONTROL KO/RAB5 + HRS/HRS_Experiment-53_czi_63b6eeb0b786b_hrm.ics.tiff]

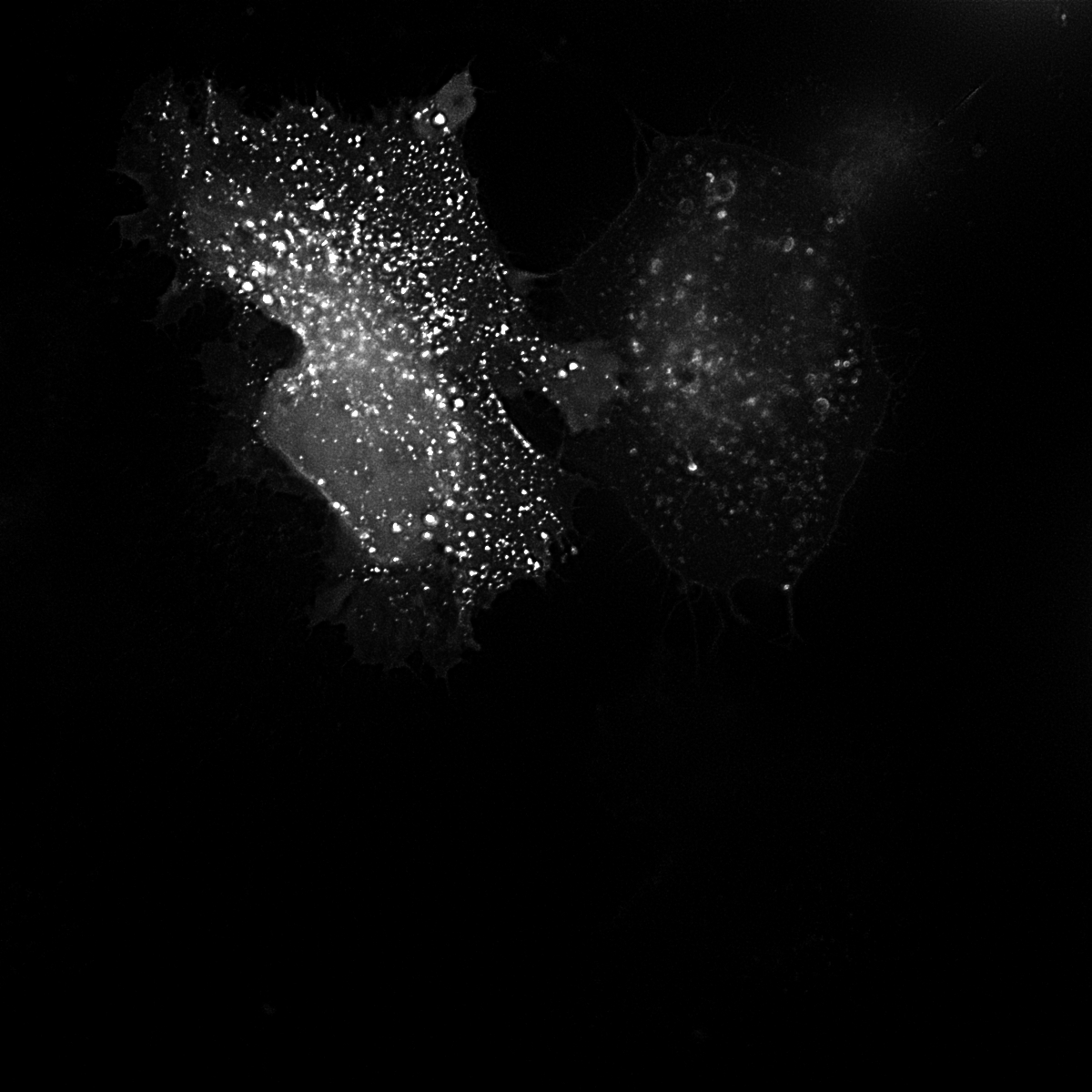

Supplement: Supplementary file 10 — Source data Fig. 8 [file 44318_2025_367_MOESM10_ESM.zip › SD figure 8/8H/Fig_8_H_data/CONTROL KO/RAB5 + HRS/RAB5_Experiment-53_czi_63b6eeb0b786b_hrm.ics.tiff]

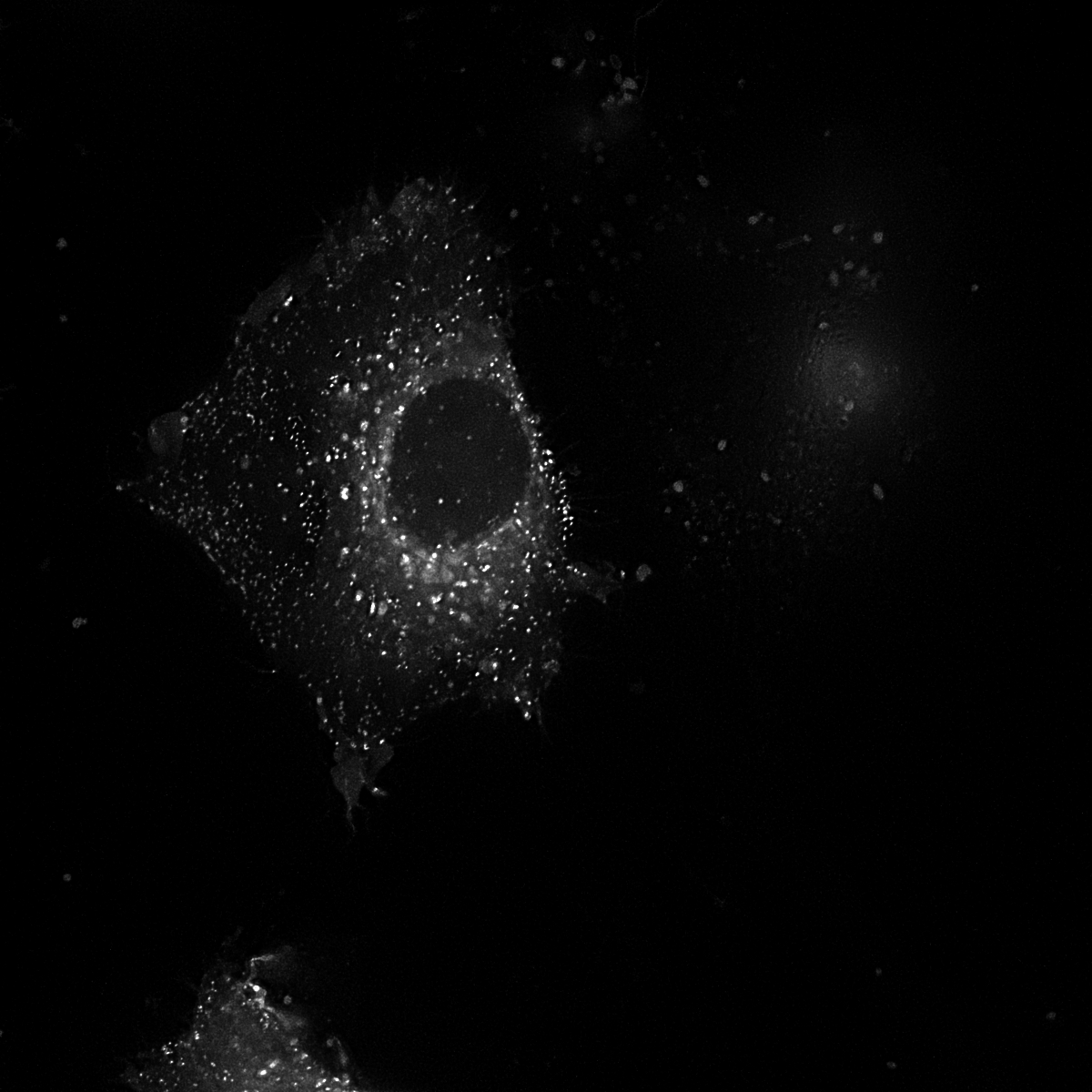

Supplement: Supplementary file 10 — Source data Fig. 8 [file 44318_2025_367_MOESM10_ESM.zip › SD figure 8/8H/Fig_8_H_data/CONTROL KO/RAB5 + EV/RAB5_Experiment-16_czi_63b6eeb0af22f_hrm.ics.tiff]

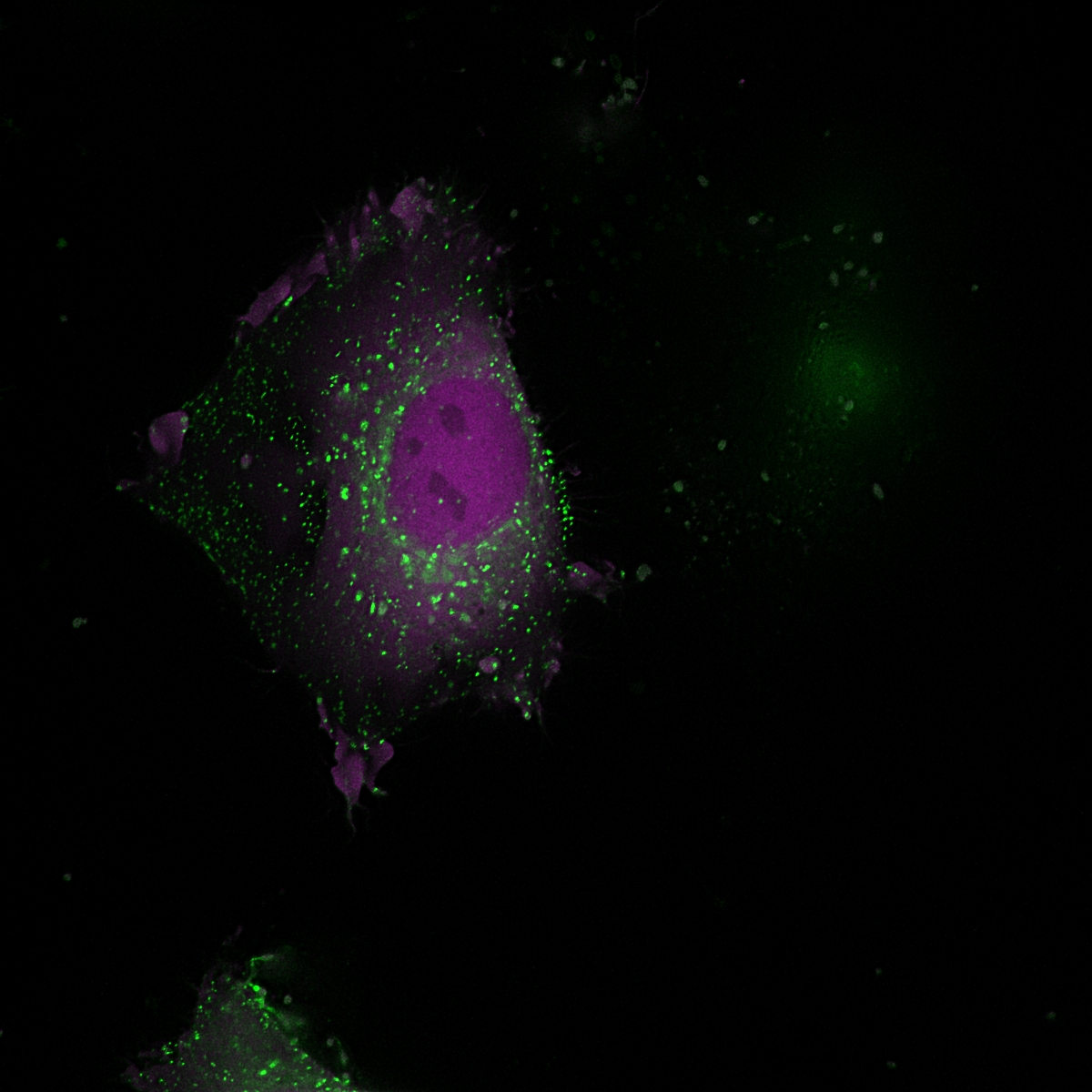

Supplement: Supplementary file 10 — Source data Fig. 8 [file 44318_2025_367_MOESM10_ESM.zip › SD figure 8/8H/Fig_8_H_data/CONTROL KO/RAB5 + EV/MERGED_Experiment-16_czi_63b6eeb0af22f_hrm.ics.tiff]

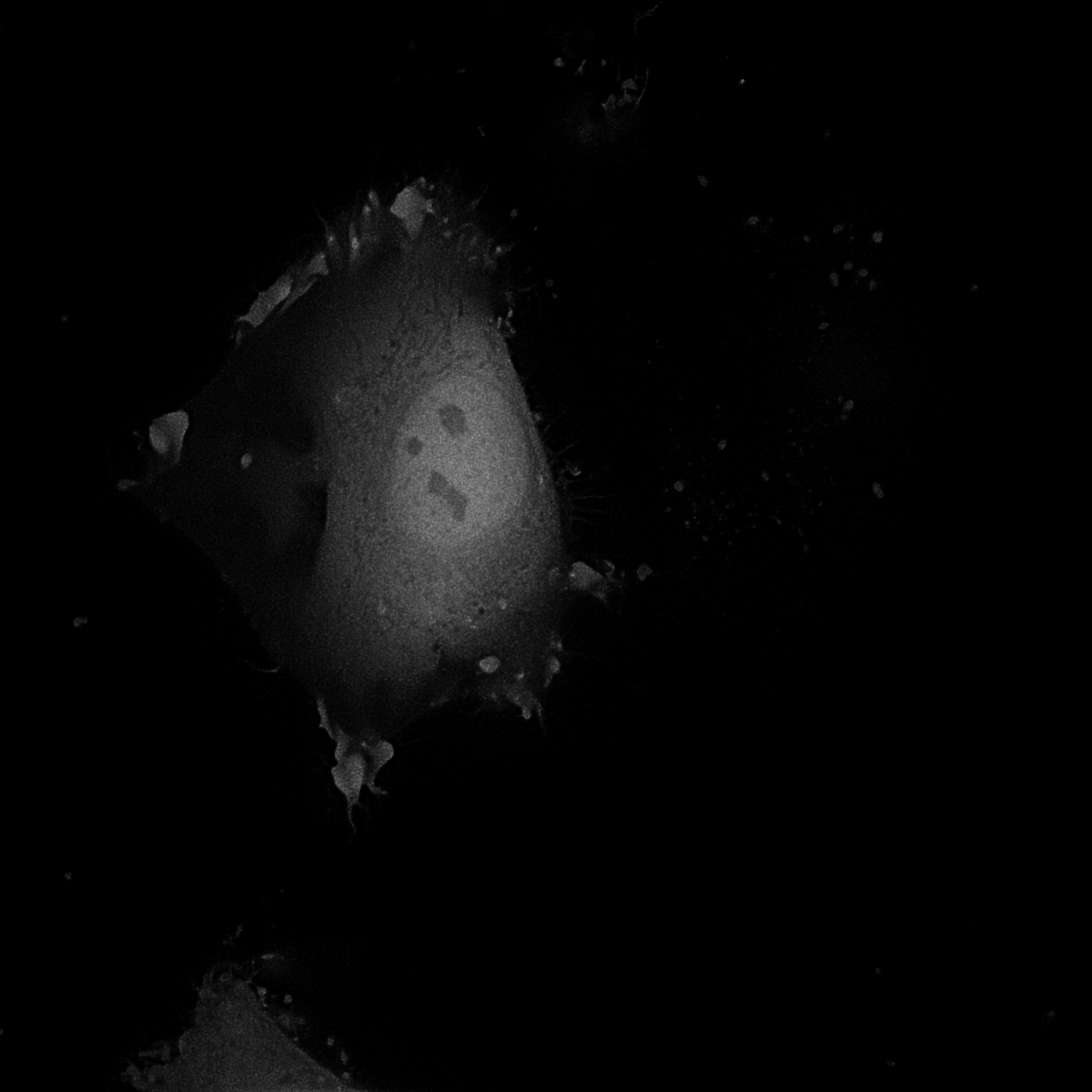

Supplement: Supplementary file 10 — Source data Fig. 8 [file 44318_2025_367_MOESM10_ESM.zip › SD figure 8/8H/Fig_8_H_data/CONTROL KO/RAB5 + EV/EV_Experiment-16_czi_63b6eeb0af22f_hrm.ics.tiff]

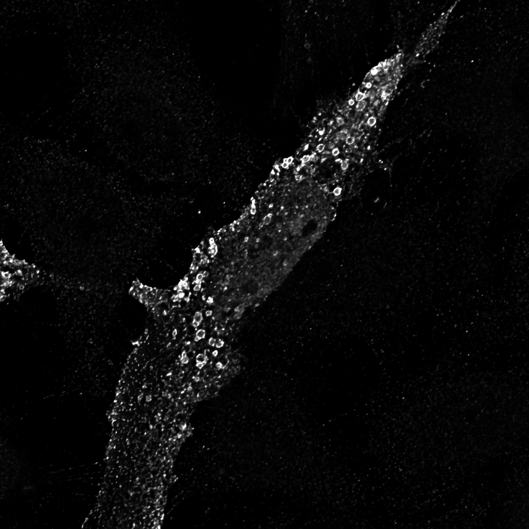

Supplement: Supplementary file 11 — Source data Fig. 9 [file 44318_2025_367_MOESM11_ESM.zip › SD figure 9/9A/Fig_9_A_Roi/CCZ1 KO/RABEX5 WT + RAB5/9_Experiment-203_czi_66d19c1c76e5d_hrm.ics.tiff]

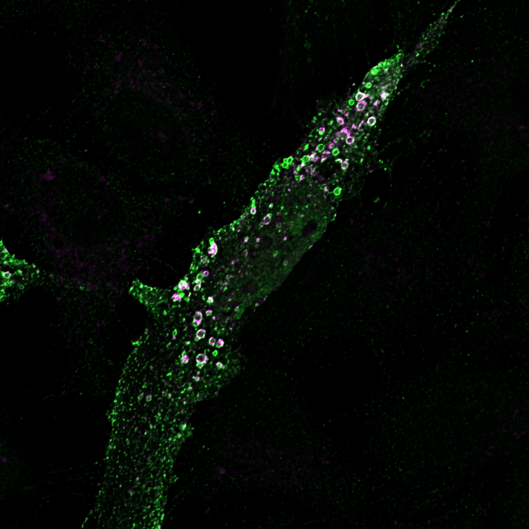

Supplement: Supplementary file 11 — Source data Fig. 9 [file 44318_2025_367_MOESM11_ESM.zip › SD figure 9/9A/Fig_9_A_Roi/CCZ1 KO/RABEX5 WT + RAB5/11_Experiment-203_czi_66d19c1c76e5d_hrm.ics.tiff]

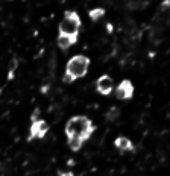

Supplement: Supplementary file 11 — Source data Fig. 9 [file 44318_2025_367_MOESM11_ESM.zip › SD figure 9/9A/Fig_9_A_Roi/CCZ1 KO/RABEX5 WT + RAB5/25_Experiment-203_czi_66d19c1c76e5d_hrm.ics.tiff]

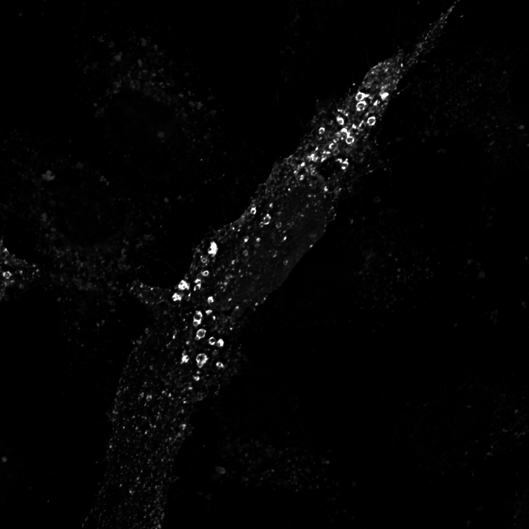

Supplement: Supplementary file 11 — Source data Fig. 9 [file 44318_2025_367_MOESM11_ESM.zip › SD figure 9/9A/Fig_9_A_Roi/CCZ1 KO/RABEX5 WT + RAB5/10_Experiment-203_czi_66d19c1c76e5d_hrm.ics.tiff]

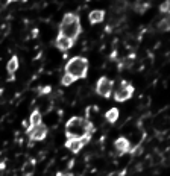

Supplement: Supplementary file 11 — Source data Fig. 9 [file 44318_2025_367_MOESM11_ESM.zip › SD figure 9/9A/Fig_9_A_Roi/CCZ1 KO/RABEX5 WT + RAB5/24_Experiment-203_czi_66d19c1c76e5d_hrm.ics.tiff]

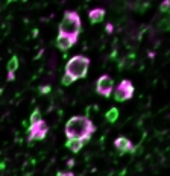

Supplement: Supplementary file 11 — Source data Fig. 9 [file 44318_2025_367_MOESM11_ESM.zip › SD figure 9/9A/Fig_9_A_Roi/CCZ1 KO/RABEX5 WT + RAB5/26_Experiment-203_czi_66d19c1c76e5d_hrm.ics.tiff]

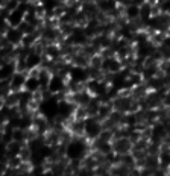

Supplement: Supplementary file 11 — Source data Fig. 9 [file 44318_2025_367_MOESM11_ESM.zip › SD figure 9/9A/Fig_9_A_Roi/CCZ1 KO/RABEX5 A58D and Y25A + RAB5/33_Experiment-224_czi_66d19c1c7b86f_hrm.ics.tiff]

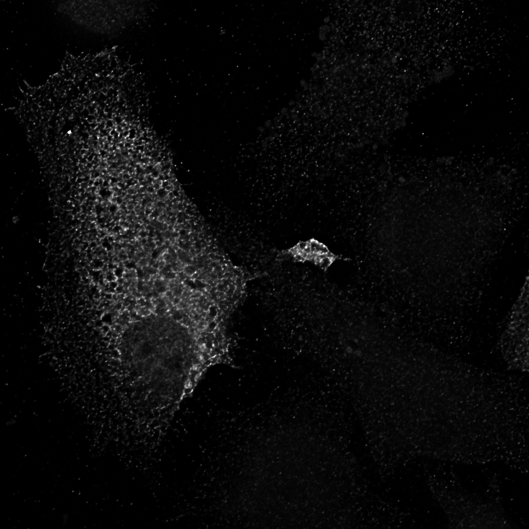

Supplement: Supplementary file 11 — Source data Fig. 9 [file 44318_2025_367_MOESM11_ESM.zip › SD figure 9/9A/Fig_9_A_Roi/CCZ1 KO/RABEX5 A58D and Y25A + RAB5/32_Experiment-224_czi_66d19c1c7b86f_hrm.ics.tiff]

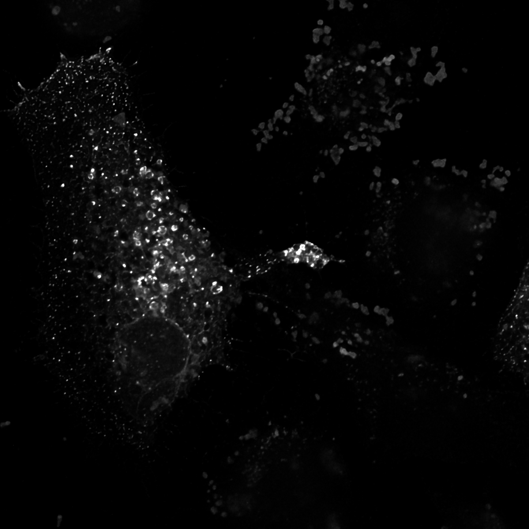

Supplement: Supplementary file 11 — Source data Fig. 9 [file 44318_2025_367_MOESM11_ESM.zip › SD figure 9/9A/Fig_9_A_Roi/CCZ1 KO/RABEX5 A58D and Y25A + RAB5/31_Experiment-224_czi_66d19c1c7b86f_hrm.ics.tiff]

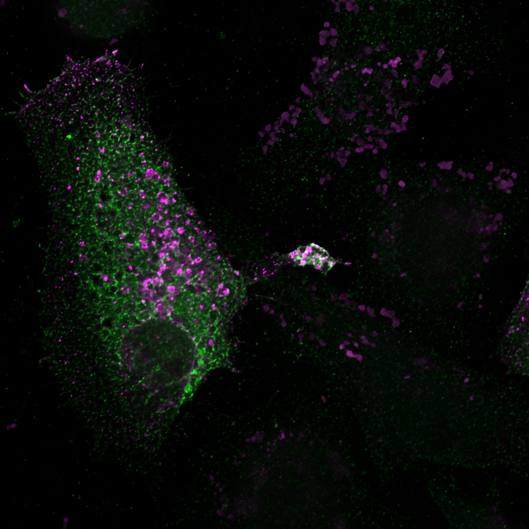

Supplement: Supplementary file 11 — Source data Fig. 9 [file 44318_2025_367_MOESM11_ESM.zip › SD figure 9/9A/Fig_9_A_Roi/CCZ1 KO/RABEX5 A58D and Y25A + RAB5/30_Experiment-224_czi_66d19c1c7b86f_hrm.ics.tiff]

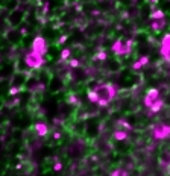

Supplement: Supplementary file 11 — Source data Fig. 9 [file 44318_2025_367_MOESM11_ESM.zip › SD figure 9/9A/Fig_9_A_Roi/CCZ1 KO/RABEX5 A58D and Y25A + RAB5/35_Experiment-224_czi_66d19c1c7b86f_hrm.ics.tiff]

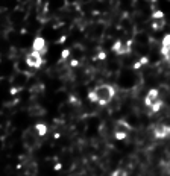

Supplement: Supplementary file 11 — Source data Fig. 9 [file 44318_2025_367_MOESM11_ESM.zip › SD figure 9/9A/Fig_9_A_Roi/CCZ1 KO/RABEX5 A58D and Y25A + RAB5/34_Experiment-224_czi_66d19c1c7b86f_hrm.ics.tiff]

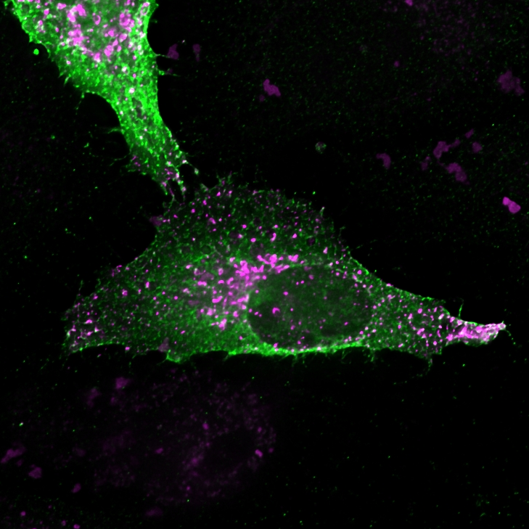

Supplement: Supplementary file 11 — Source data Fig. 9 [file 44318_2025_367_MOESM11_ESM.zip › SD figure 9/9A/Fig_9_A_Roi/CCZ1 KO/RABEX5 A58D + RAB5/14_Experiment-212_czi_66d19c1c789b1_hrm.ics.tiff]

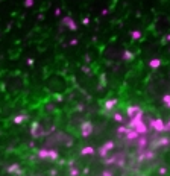

Supplement: Supplementary file 11 — Source data Fig. 9 [file 44318_2025_367_MOESM11_ESM.zip › SD figure 9/9A/Fig_9_A_Roi/CCZ1 KO/RABEX5 A58D + RAB5/29_Experiment-212_czi_66d19c1c789b1_hrm.ics.tiff]

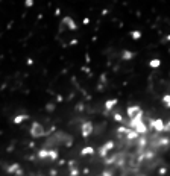

Supplement: Supplementary file 11 — Source data Fig. 9 [file 44318_2025_367_MOESM11_ESM.zip › SD figure 9/9A/Fig_9_A_Roi/CCZ1 KO/RABEX5 A58D + RAB5/28_Experiment-212_czi_66d19c1c789b1_hrm.ics.tiff]

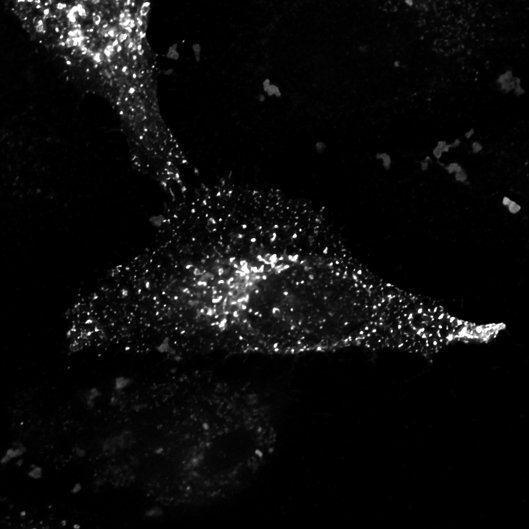

Supplement: Supplementary file 11 — Source data Fig. 9 [file 44318_2025_367_MOESM11_ESM.zip › SD figure 9/9A/Fig_9_A_Roi/CCZ1 KO/RABEX5 A58D + RAB5/13_Experiment-212_czi_66d19c1c789b1_hrm.ics.tiff]

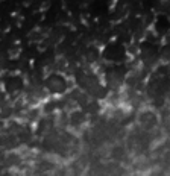

Supplement: Supplementary file 11 — Source data Fig. 9 [file 44318_2025_367_MOESM11_ESM.zip › SD figure 9/9A/Fig_9_A_Roi/CCZ1 KO/RABEX5 A58D + RAB5/27_Experiment-212_czi_66d19c1c789b1_hrm.ics.tiff]

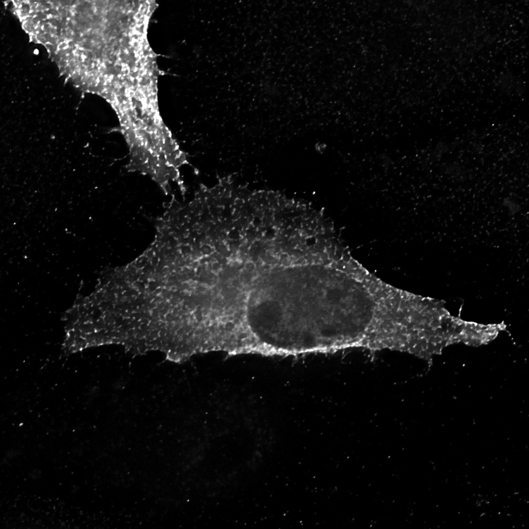

Supplement: Supplementary file 11 — Source data Fig. 9 [file 44318_2025_367_MOESM11_ESM.zip › SD figure 9/9A/Fig_9_A_Roi/CCZ1 KO/RABEX5 A58D + RAB5/12_Experiment-212_czi_66d19c1c789b1_hrm.ics.tiff]

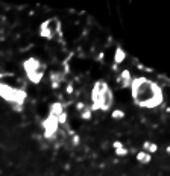

Supplement: Supplementary file 11 — Source data Fig. 9 [file 44318_2025_367_MOESM11_ESM.zip › SD figure 9/9A/Fig_9_A_Roi/Control KO/RABEX5 WT + RAB5/16_Experiment-173_czi_66d19c1c70998_hrm.ics.tiff]

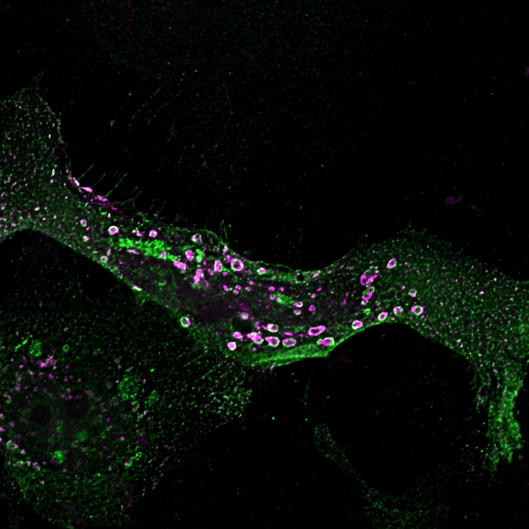

Supplement: Supplementary file 11 — Source data Fig. 9 [file 44318_2025_367_MOESM11_ESM.zip › SD figure 9/9A/Fig_9_A_Roi/Control KO/RABEX5 WT + RAB5/2_Experiment-173_czi_66d19c1c70998_hrm.ics.tiff]

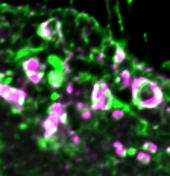

Supplement: Supplementary file 11 — Source data Fig. 9 [file 44318_2025_367_MOESM11_ESM.zip › SD figure 9/9A/Fig_9_A_Roi/Control KO/RABEX5 WT + RAB5/17_Experiment-173_czi_66d19c1c70998_hrm.ics.tiff]

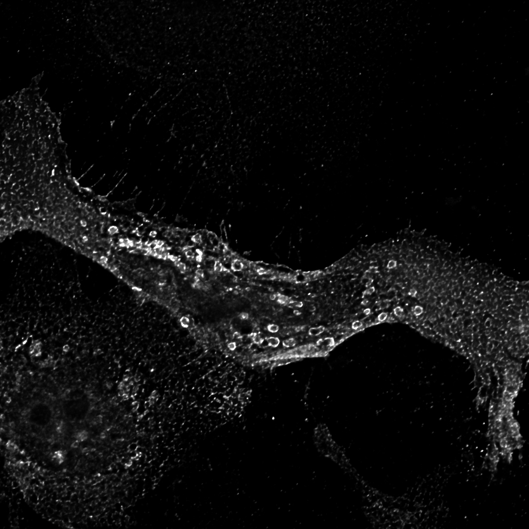

Supplement: Supplementary file 11 — Source data Fig. 9 [file 44318_2025_367_MOESM11_ESM.zip › SD figure 9/9A/Fig_9_A_Roi/Control KO/RABEX5 WT + RAB5/0_Experiment-173_czi_66d19c1c70998_hrm.ics.tiff]

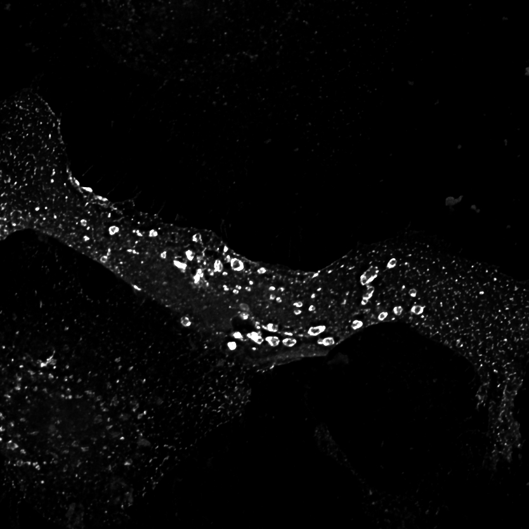

Supplement: Supplementary file 11 — Source data Fig. 9 [file 44318_2025_367_MOESM11_ESM.zip › SD figure 9/9A/Fig_9_A_Roi/Control KO/RABEX5 WT + RAB5/1_Experiment-173_czi_66d19c1c70998_hrm.ics.tiff]

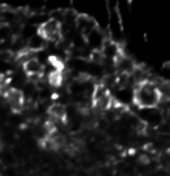

Supplement: Supplementary file 11 — Source data Fig. 9 [file 44318_2025_367_MOESM11_ESM.zip › SD figure 9/9A/Fig_9_A_Roi/Control KO/RABEX5 WT + RAB5/15_Experiment-173_czi_66d19c1c70998_hrm.ics.tiff]

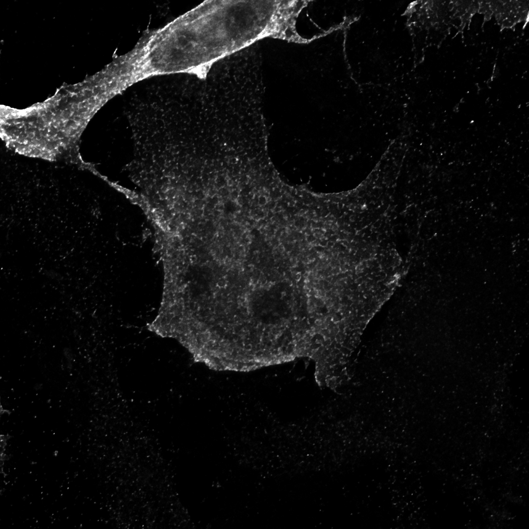

Supplement: Supplementary file 11 — Source data Fig. 9 [file 44318_2025_367_MOESM11_ESM.zip › SD figure 9/9A/Fig_9_A_Roi/Control KO/RABEX5 A58D and Y25A + RAB5/6_Experiment-192_czi_66d19c1c7490f_hrm.ics.tiff]

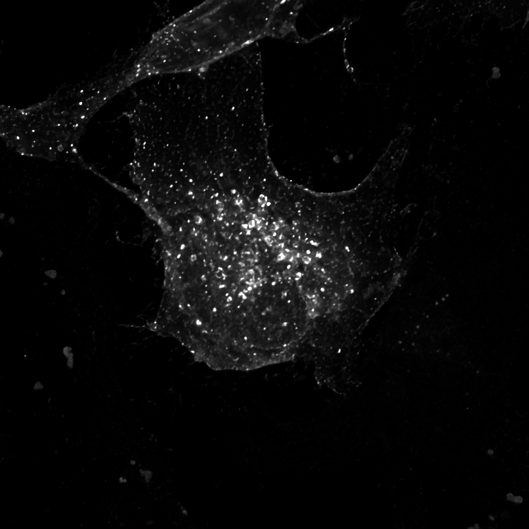

Supplement: Supplementary file 11 — Source data Fig. 9 [file 44318_2025_367_MOESM11_ESM.zip › SD figure 9/9A/Fig_9_A_Roi/Control KO/RABEX5 A58D and Y25A + RAB5/7_Experiment-192_czi_66d19c1c7490f_hrm.ics.tiff]

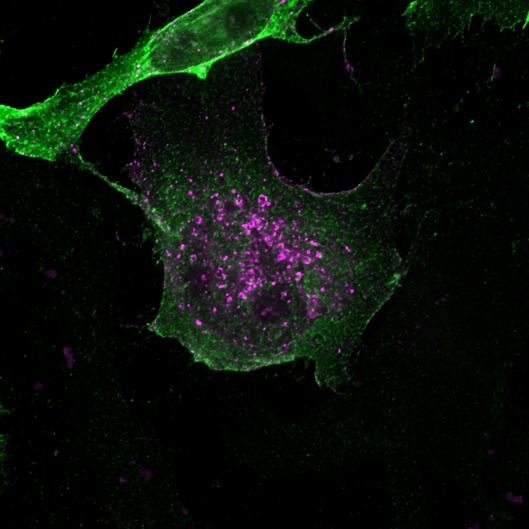

Supplement: Supplementary file 11 — Source data Fig. 9 [file 44318_2025_367_MOESM11_ESM.zip › SD figure 9/9A/Fig_9_A_Roi/Control KO/RABEX5 A58D and Y25A + RAB5/8_Experiment-192_czi_66d19c1c7490f_hrm.ics.tiff]

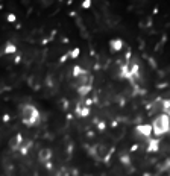

Supplement: Supplementary file 11 — Source data Fig. 9 [file 44318_2025_367_MOESM11_ESM.zip › SD figure 9/9A/Fig_9_A_Roi/Control KO/RABEX5 A58D and Y25A + RAB5/22_Experiment-192_czi_66d19c1c7490f_hrm.ics.tiff]

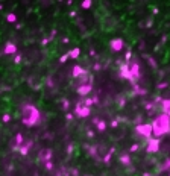

Supplement: Supplementary file 11 — Source data Fig. 9 [file 44318_2025_367_MOESM11_ESM.zip › SD figure 9/9A/Fig_9_A_Roi/Control KO/RABEX5 A58D and Y25A + RAB5/23_Experiment-192_czi_66d19c1c7490f_hrm.ics.tiff]

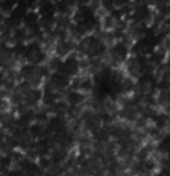

Supplement: Supplementary file 11 — Source data Fig. 9 [file 44318_2025_367_MOESM11_ESM.zip › SD figure 9/9A/Fig_9_A_Roi/Control KO/RABEX5 A58D and Y25A + RAB5/21_Experiment-192_czi_66d19c1c7490f_hrm.ics.tiff]

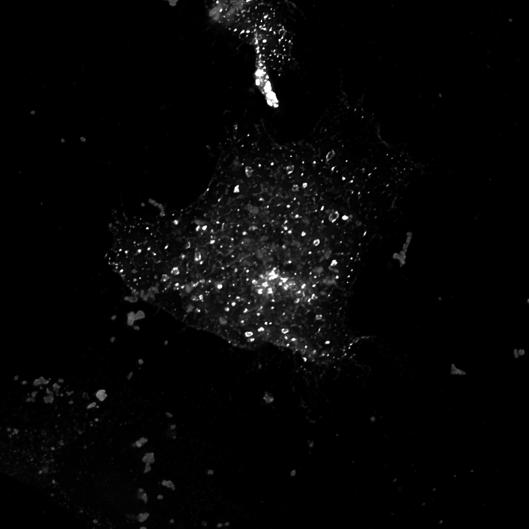

Supplement: Supplementary file 11 — Source data Fig. 9 [file 44318_2025_367_MOESM11_ESM.zip › SD figure 9/9A/Fig_9_A_Roi/Control KO/RABEX5 A58D + RAB5/4_Experiment-185_czi_66d19c1c7315a_hrm.ics.tiff]

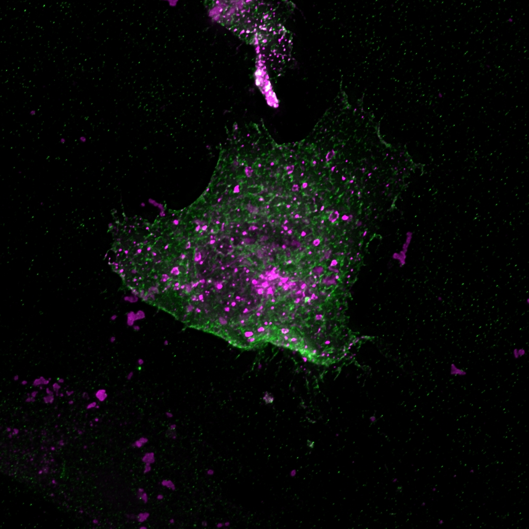

Supplement: Supplementary file 11 — Source data Fig. 9 [file 44318_2025_367_MOESM11_ESM.zip › SD figure 9/9A/Fig_9_A_Roi/Control KO/RABEX5 A58D + RAB5/5_Experiment-185_czi_66d19c1c7315a_hrm.ics.tiff]

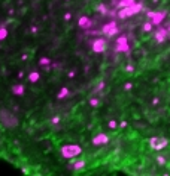

Supplement: Supplementary file 11 — Source data Fig. 9 [file 44318_2025_367_MOESM11_ESM.zip › SD figure 9/9A/Fig_9_A_Roi/Control KO/RABEX5 A58D + RAB5/20_Experiment-185_czi_66d19c1c7315a_hrm.ics.tiff]

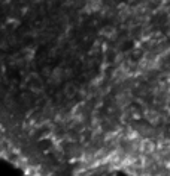

Supplement: Supplementary file 11 — Source data Fig. 9 [file 44318_2025_367_MOESM11_ESM.zip › SD figure 9/9A/Fig_9_A_Roi/Control KO/RABEX5 A58D + RAB5/18_Experiment-185_czi_66d19c1c7315a_hrm.ics.tiff]

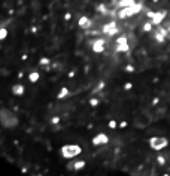

Supplement: Supplementary file 11 — Source data Fig. 9 [file 44318_2025_367_MOESM11_ESM.zip › SD figure 9/9A/Fig_9_A_Roi/Control KO/RABEX5 A58D + RAB5/19_Experiment-185_czi_66d19c1c7315a_hrm.ics.tiff]

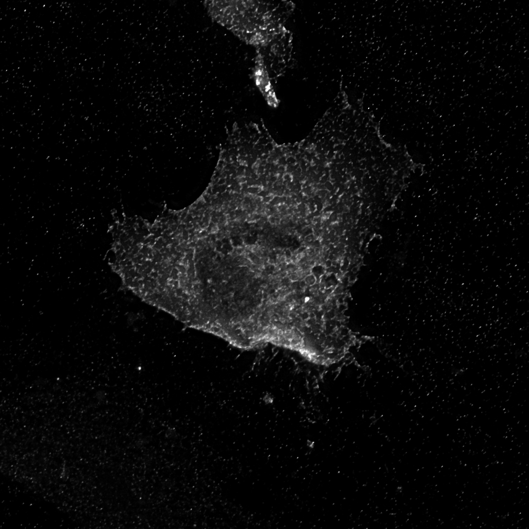

Supplement: Supplementary file 11 — Source data Fig. 9 [file 44318_2025_367_MOESM11_ESM.zip › SD figure 9/9A/Fig_9_A_Roi/Control KO/RABEX5 A58D + RAB5/3_Experiment-185_czi_66d19c1c7315a_hrm.ics.tiff]

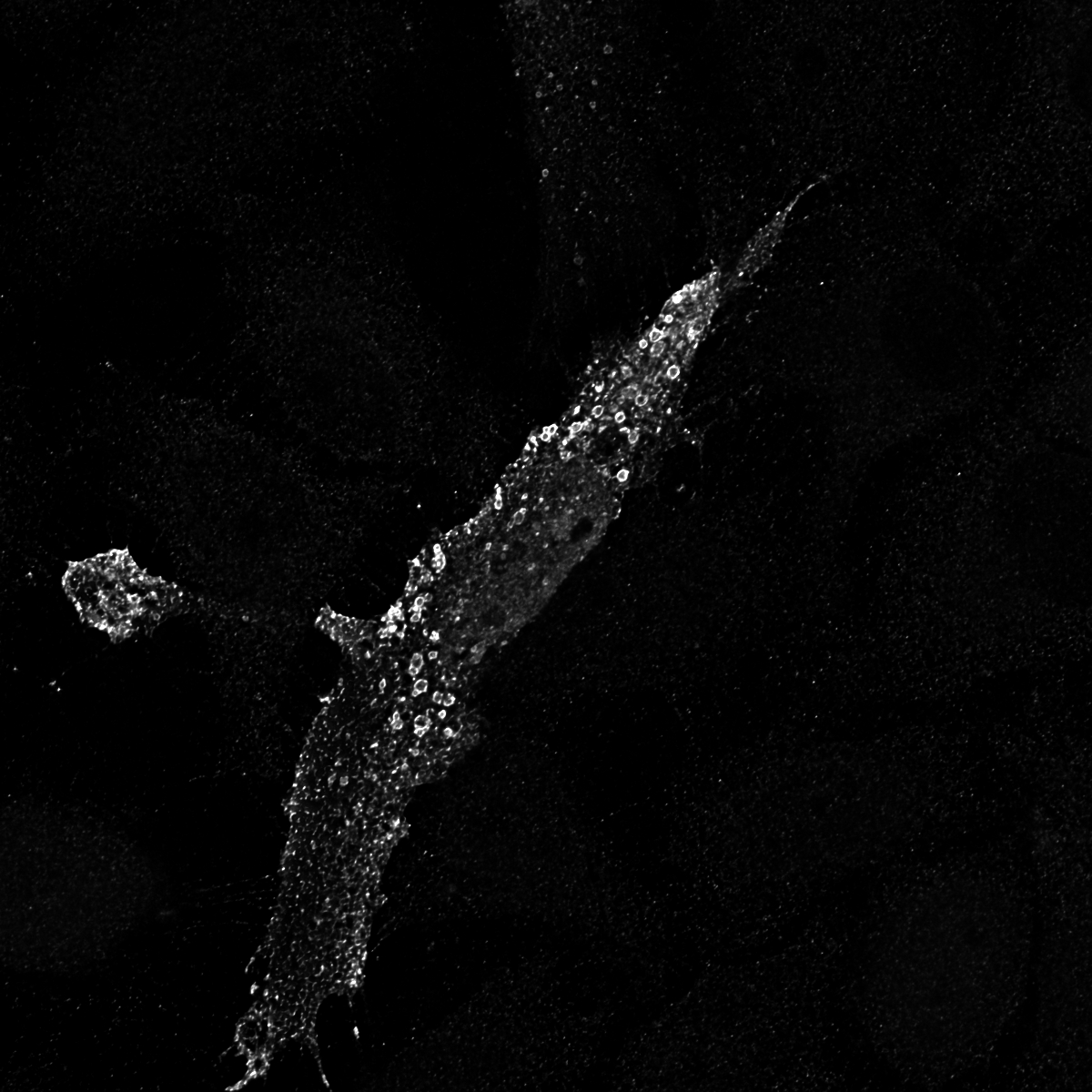

Supplement: Supplementary file 11 — Source data Fig. 9 [file 44318_2025_367_MOESM11_ESM.zip › SD figure 9/9A/Fig_9_A_data/CCZ1 KO/RABEX5 WT + RAB5/9_Experiment-203_czi_66d19c1c76e5d_hrm.ics.tiff]

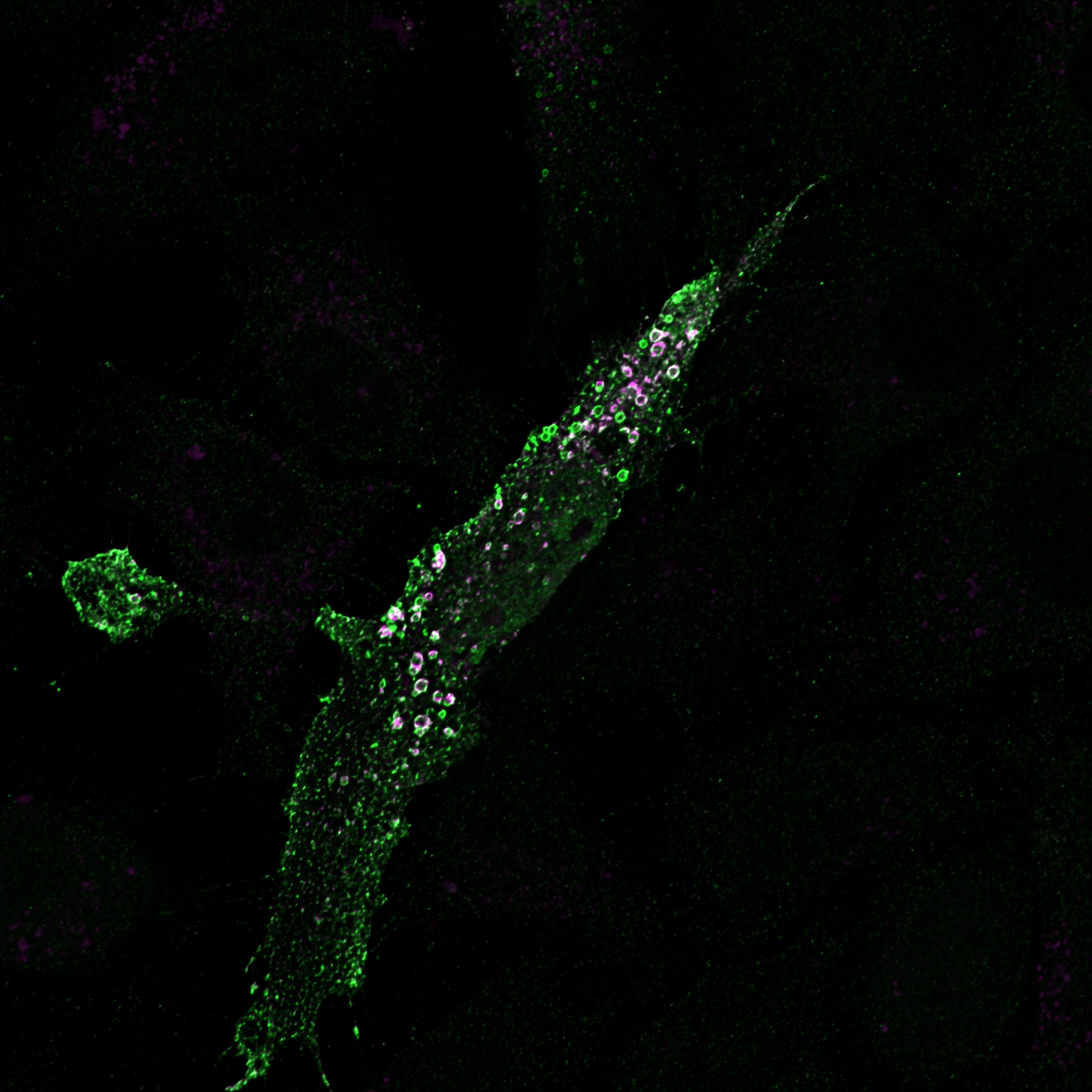

Supplement: Supplementary file 11 — Source data Fig. 9 [file 44318_2025_367_MOESM11_ESM.zip › SD figure 9/9A/Fig_9_A_data/CCZ1 KO/RABEX5 WT + RAB5/11_Experiment-203_czi_66d19c1c76e5d_hrm.ics.tiff]

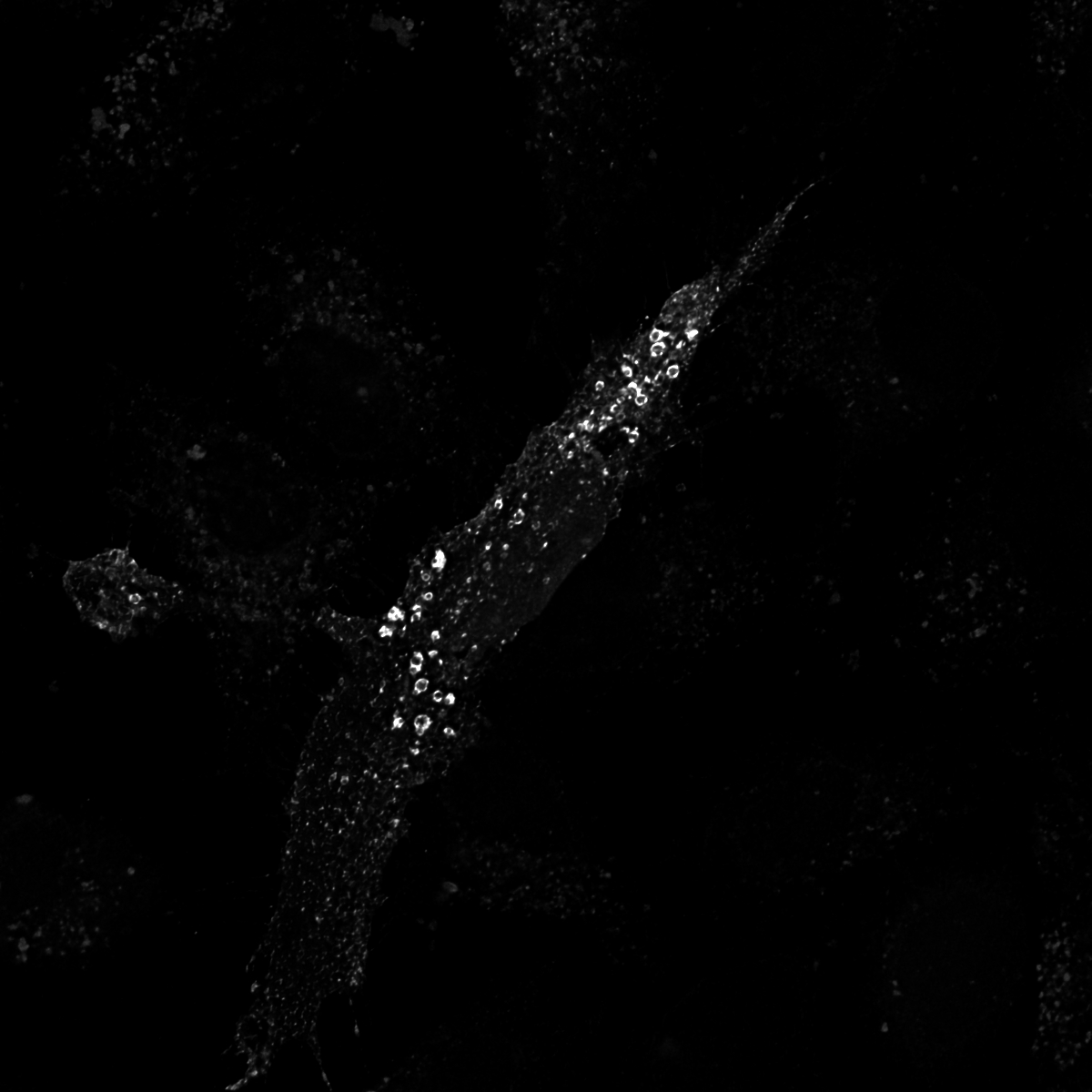

Supplement: Supplementary file 11 — Source data Fig. 9 [file 44318_2025_367_MOESM11_ESM.zip › SD figure 9/9A/Fig_9_A_data/CCZ1 KO/RABEX5 WT + RAB5/10_Experiment-203_czi_66d19c1c76e5d_hrm.ics.tiff]

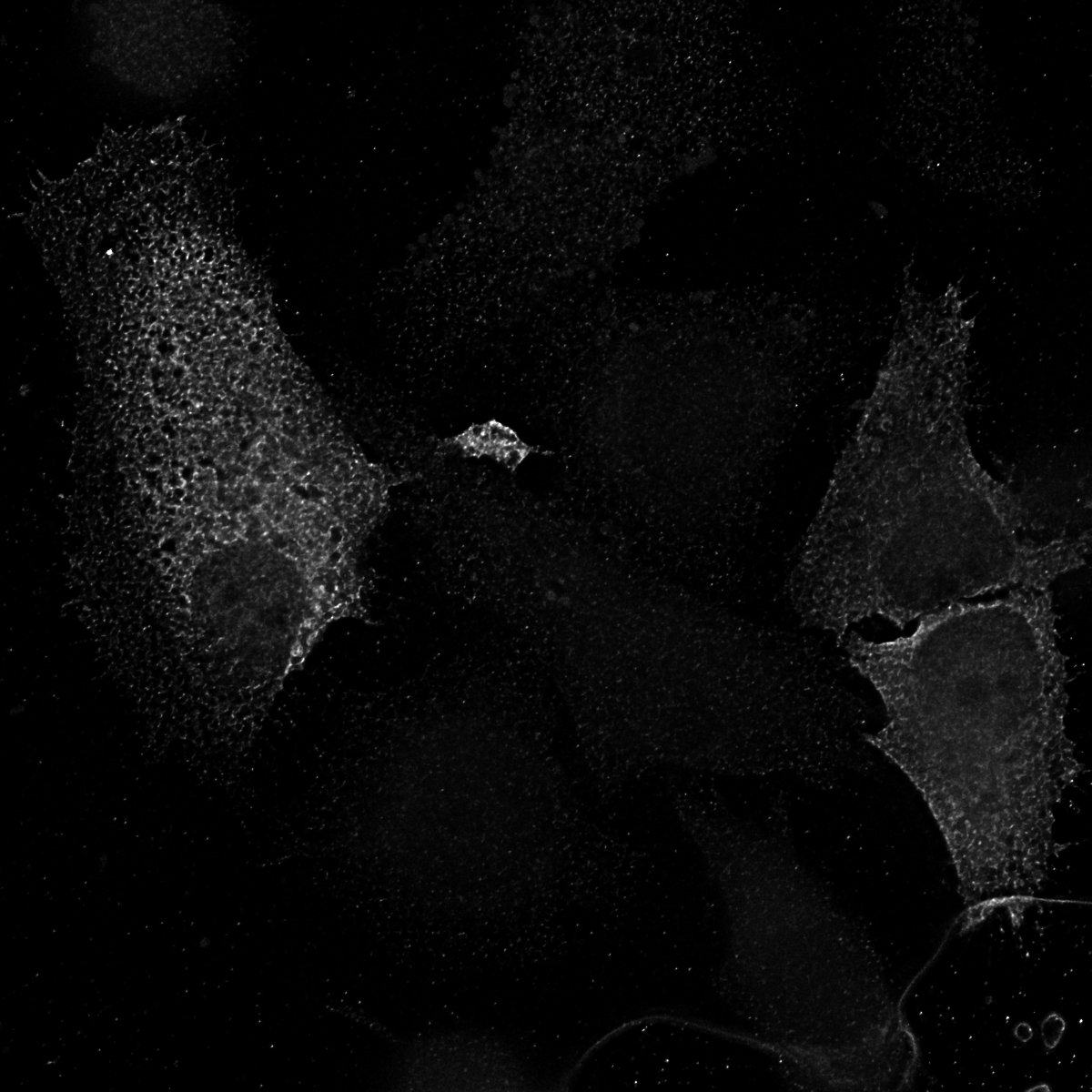

Supplement: Supplementary file 11 — Source data Fig. 9 [file 44318_2025_367_MOESM11_ESM.zip › SD figure 9/9A/Fig_9_A_data/CCZ1 KO/RABEX5 A58D and Y25A + RAB5/33_Experiment-224_czi_66d19c1c7b86f_hrm.ics.tiff]

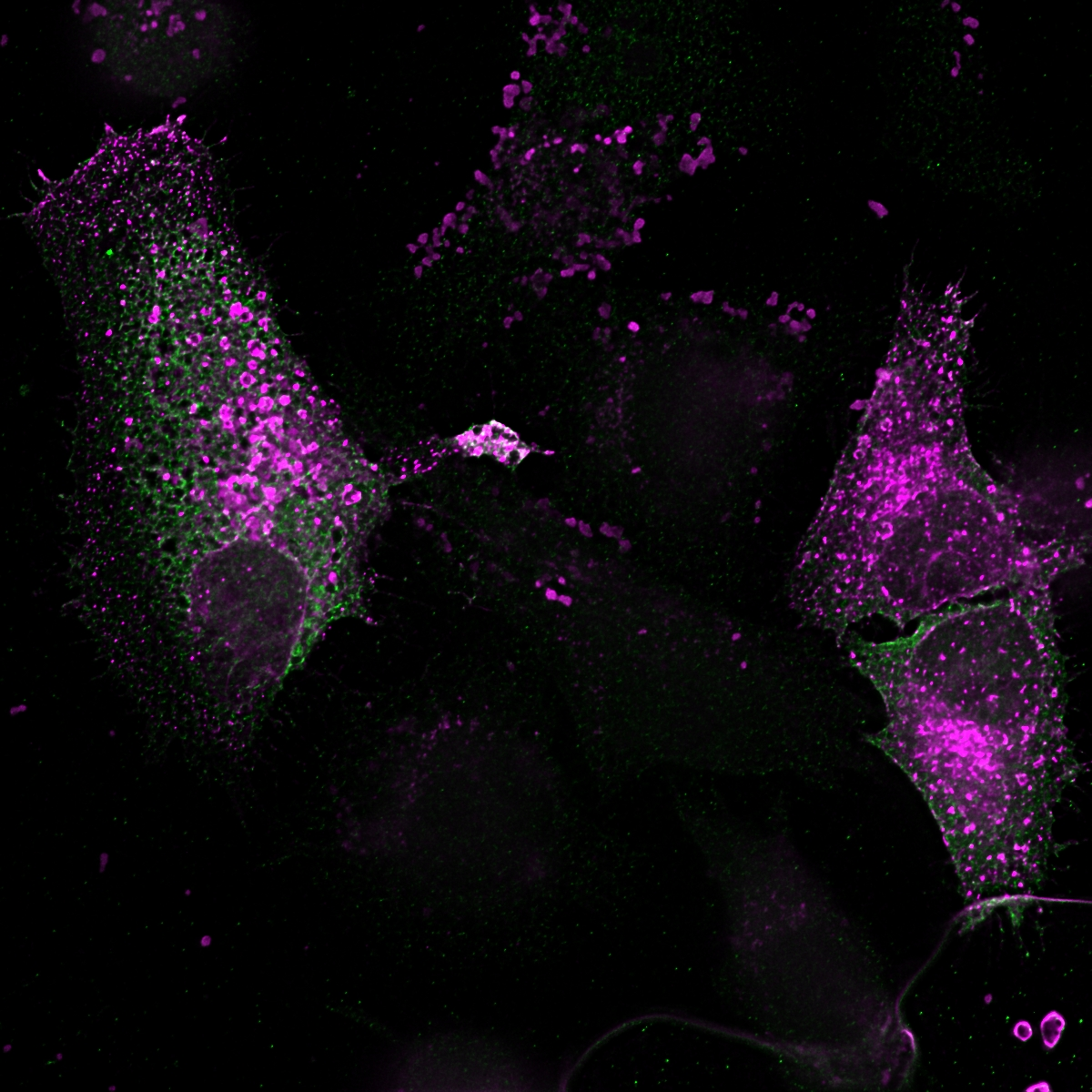

Supplement: Supplementary file 11 — Source data Fig. 9 [file 44318_2025_367_MOESM11_ESM.zip › SD figure 9/9A/Fig_9_A_data/CCZ1 KO/RABEX5 A58D and Y25A + RAB5/35_Experiment-224_czi_66d19c1c7b86f_hrm.ics.tiff]

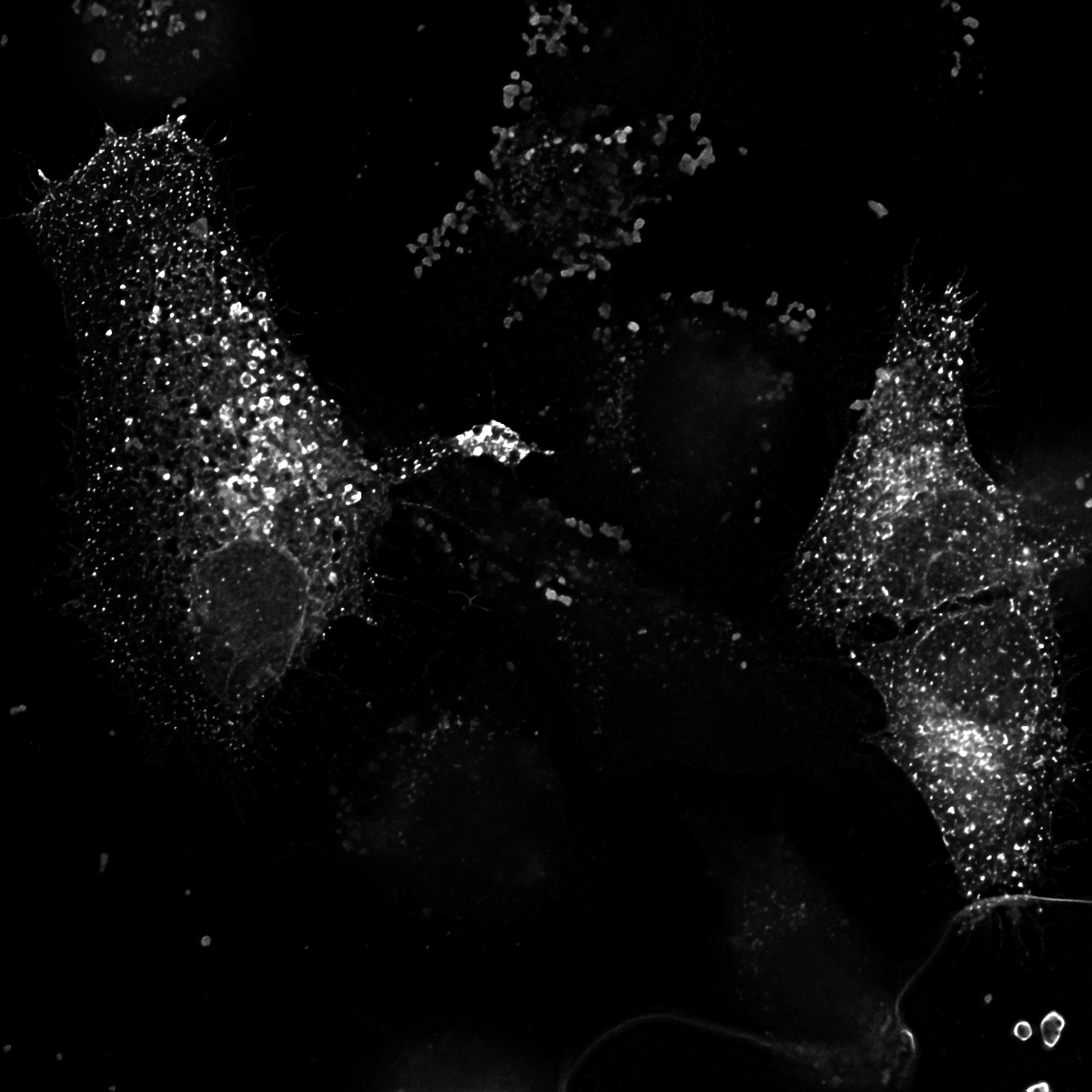

Supplement: Supplementary file 11 — Source data Fig. 9 [file 44318_2025_367_MOESM11_ESM.zip › SD figure 9/9A/Fig_9_A_data/CCZ1 KO/RABEX5 A58D and Y25A + RAB5/34_Experiment-224_czi_66d19c1c7b86f_hrm.ics.tiff]

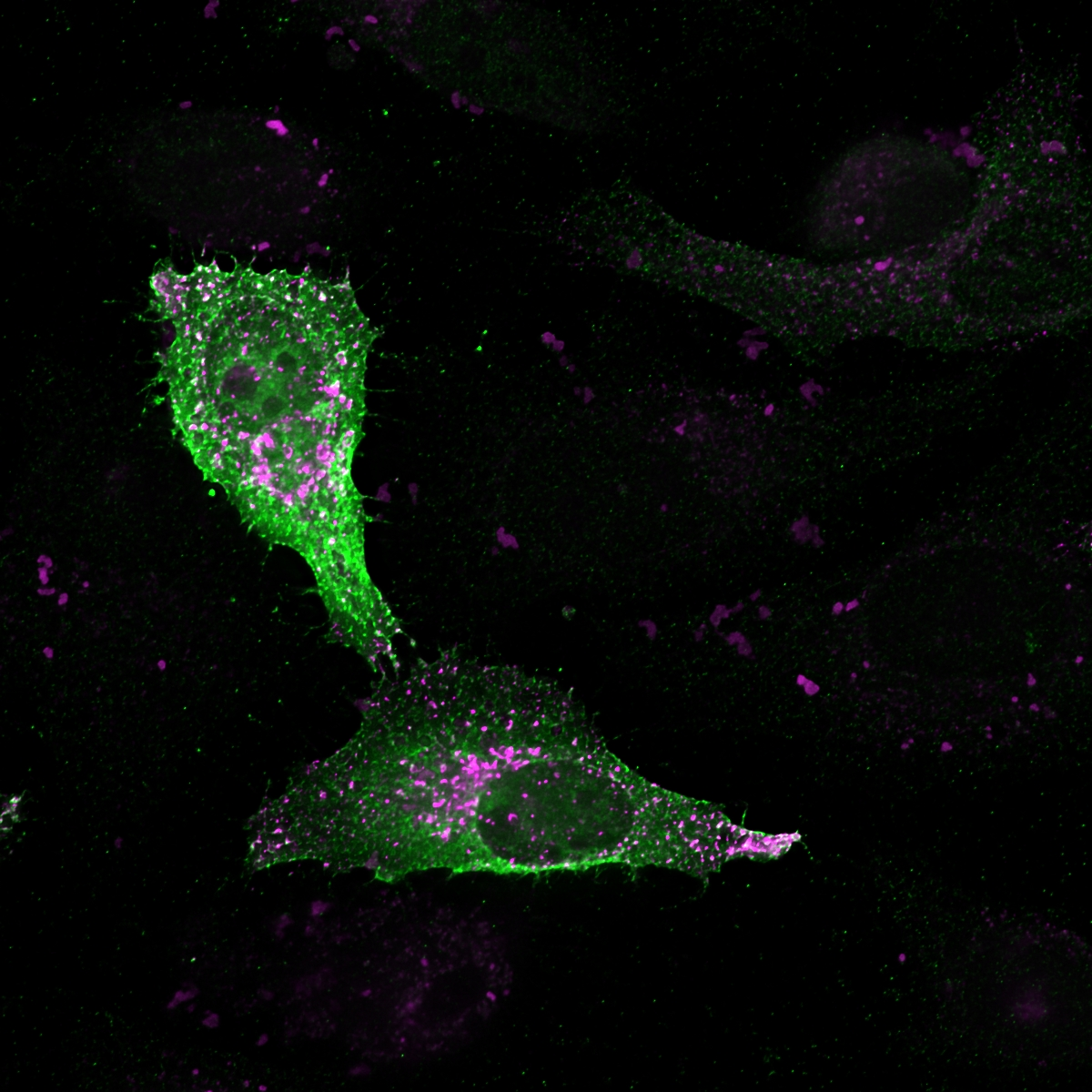

Supplement: Supplementary file 11 — Source data Fig. 9 [file 44318_2025_367_MOESM11_ESM.zip › SD figure 9/9A/Fig_9_A_data/CCZ1 KO/RABEX5 A58D + RAB5/14_Experiment-212_czi_66d19c1c789b1_hrm.ics.tiff]

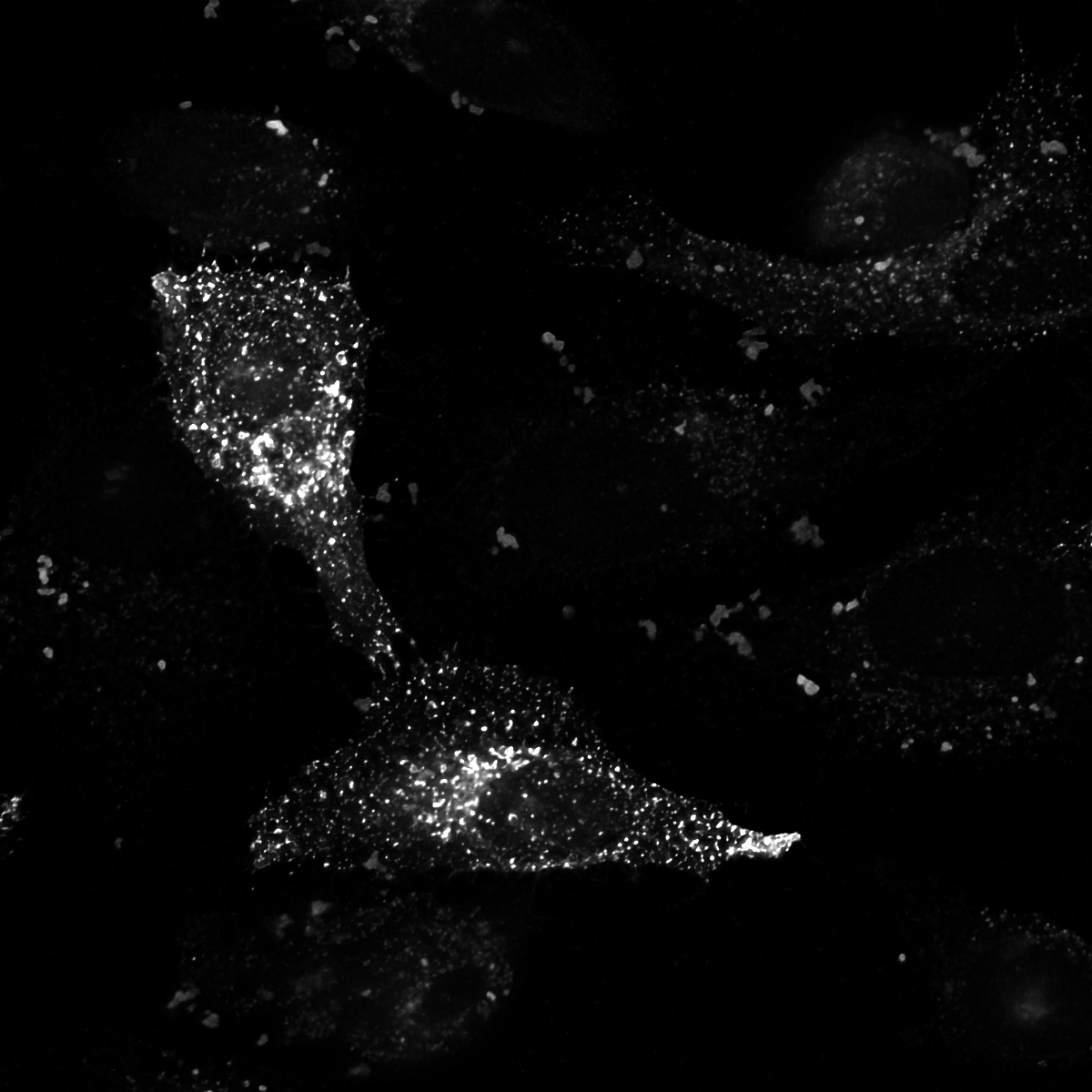

Supplement: Supplementary file 11 — Source data Fig. 9 [file 44318_2025_367_MOESM11_ESM.zip › SD figure 9/9A/Fig_9_A_data/CCZ1 KO/RABEX5 A58D + RAB5/13_Experiment-212_czi_66d19c1c789b1_hrm.ics.tiff]

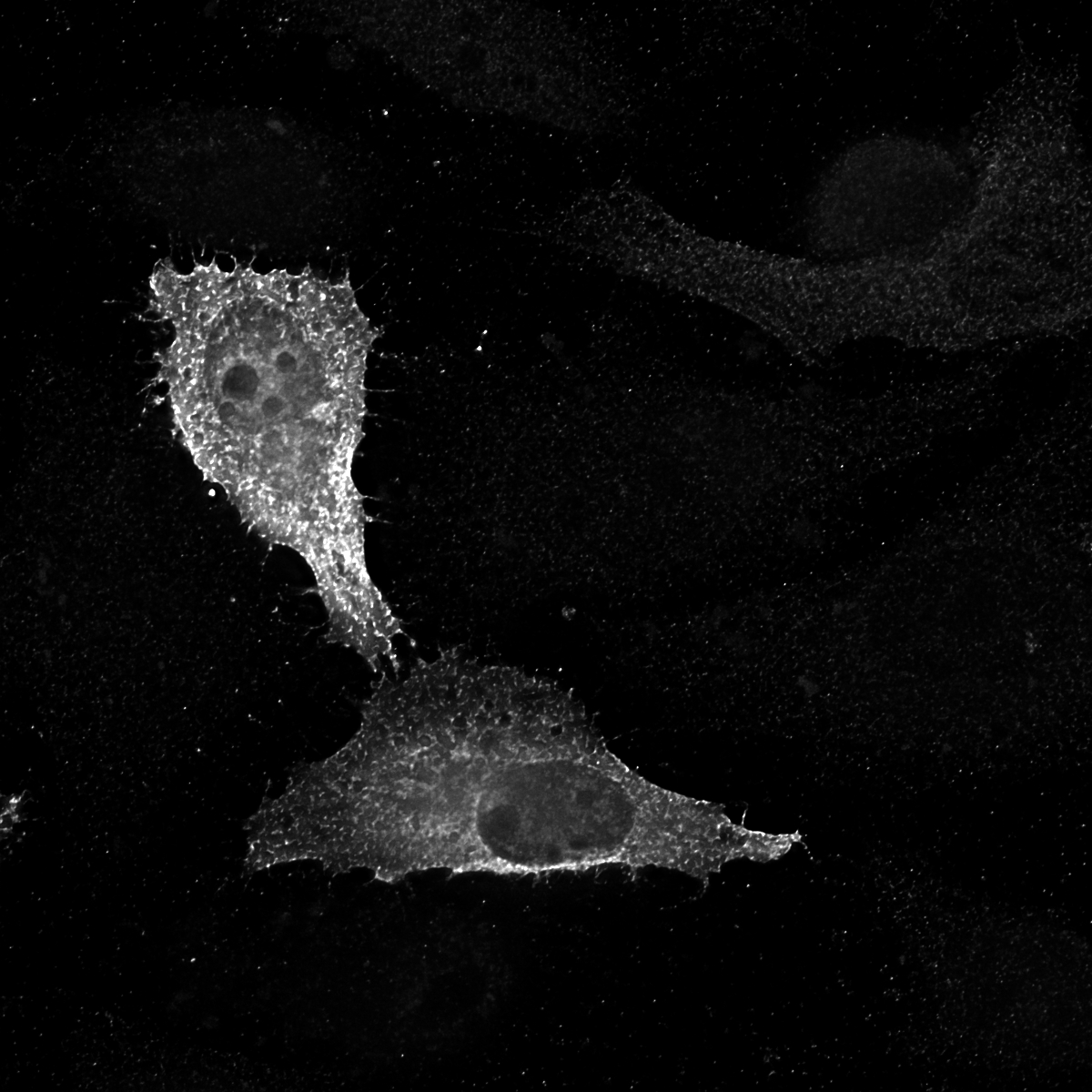

Supplement: Supplementary file 11 — Source data Fig. 9 [file 44318_2025_367_MOESM11_ESM.zip › SD figure 9/9A/Fig_9_A_data/CCZ1 KO/RABEX5 A58D + RAB5/12_Experiment-212_czi_66d19c1c789b1_hrm.ics.tiff]

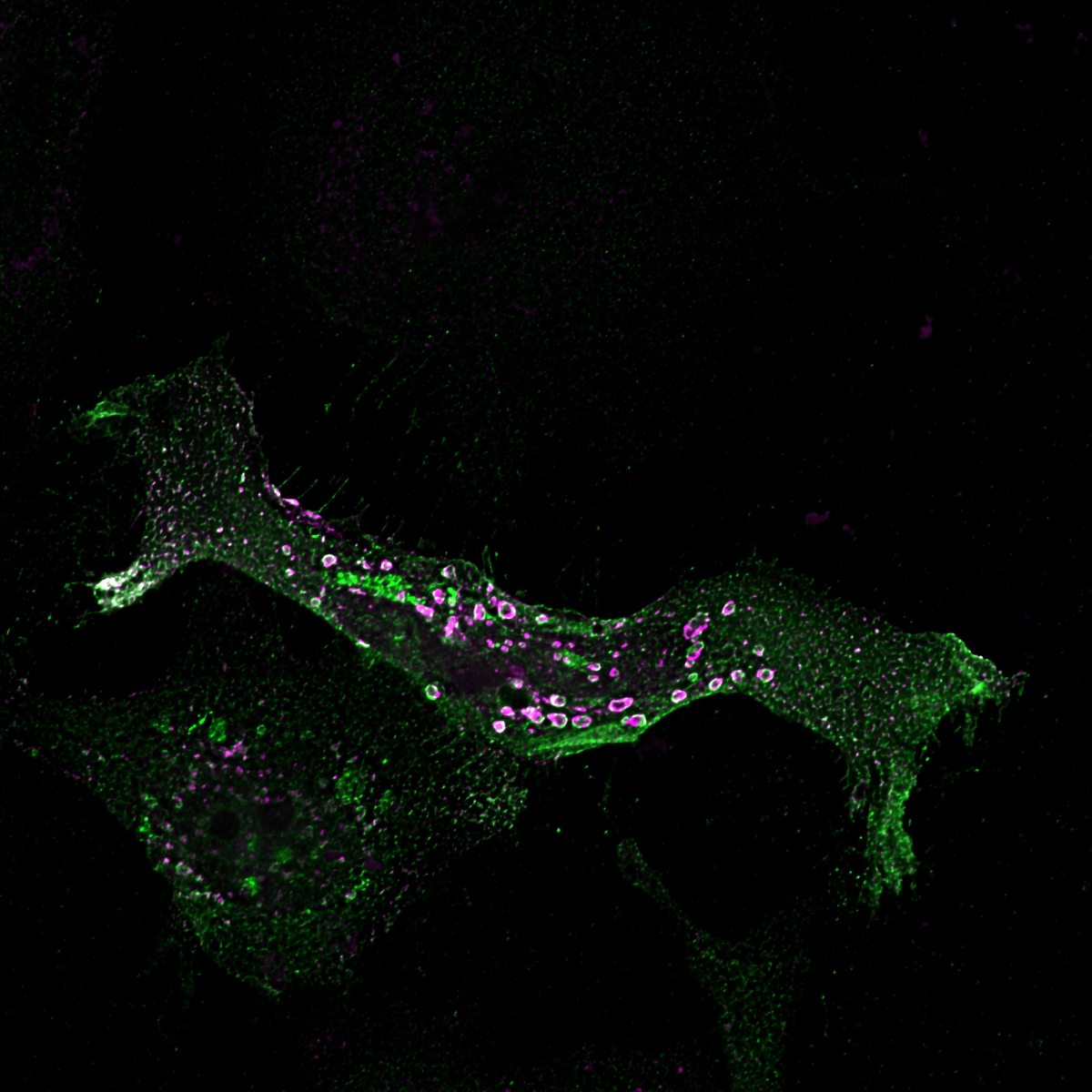

Supplement: Supplementary file 11 — Source data Fig. 9 [file 44318_2025_367_MOESM11_ESM.zip › SD figure 9/9A/Fig_9_A_data/Control KO/RABEX5 WT + RAB5/2_Experiment-173_czi_66d19c1c70998_hrm.ics.tiff]

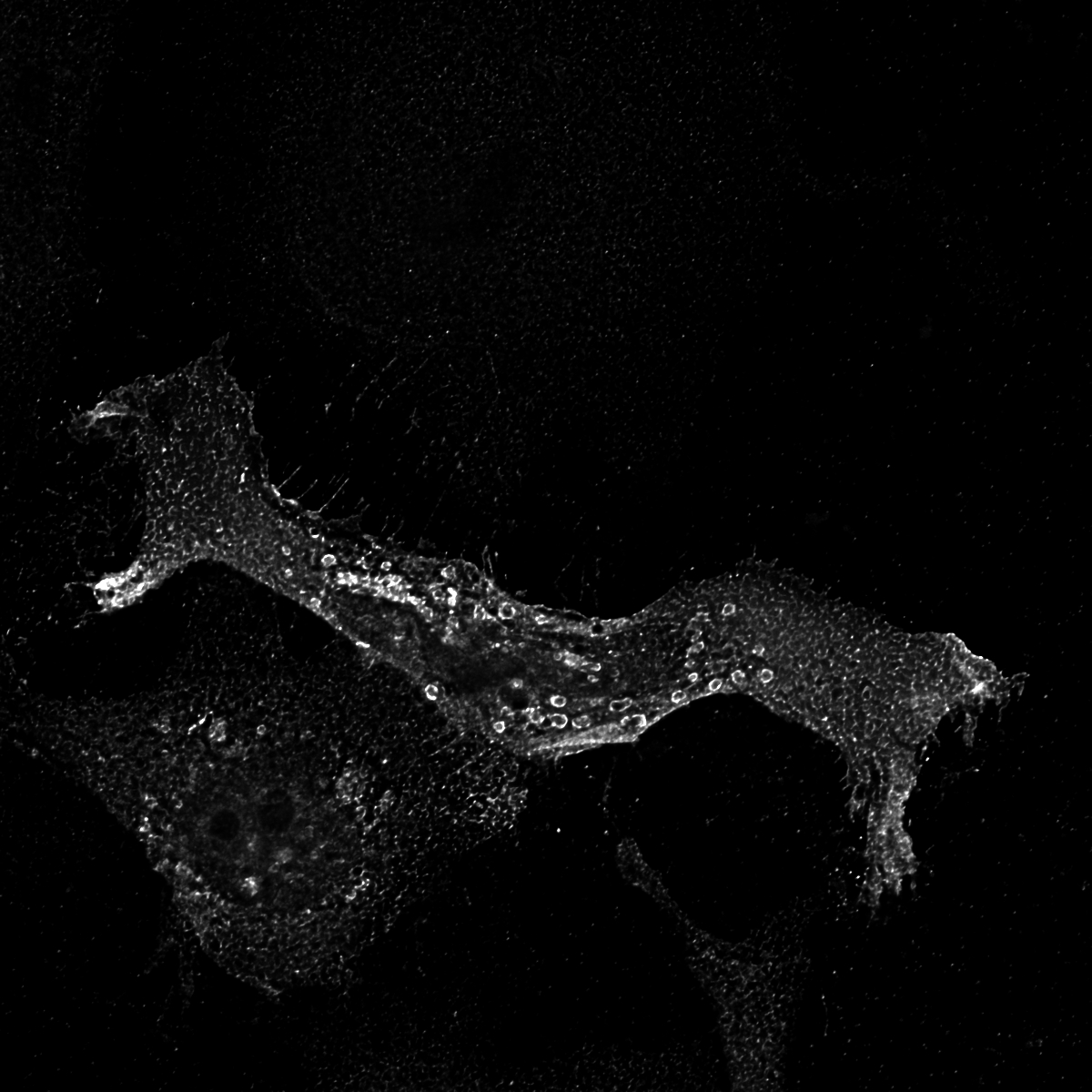

Supplement: Supplementary file 11 — Source data Fig. 9 [file 44318_2025_367_MOESM11_ESM.zip › SD figure 9/9A/Fig_9_A_data/Control KO/RABEX5 WT + RAB5/0_Experiment-173_czi_66d19c1c70998_hrm.ics.tiff]

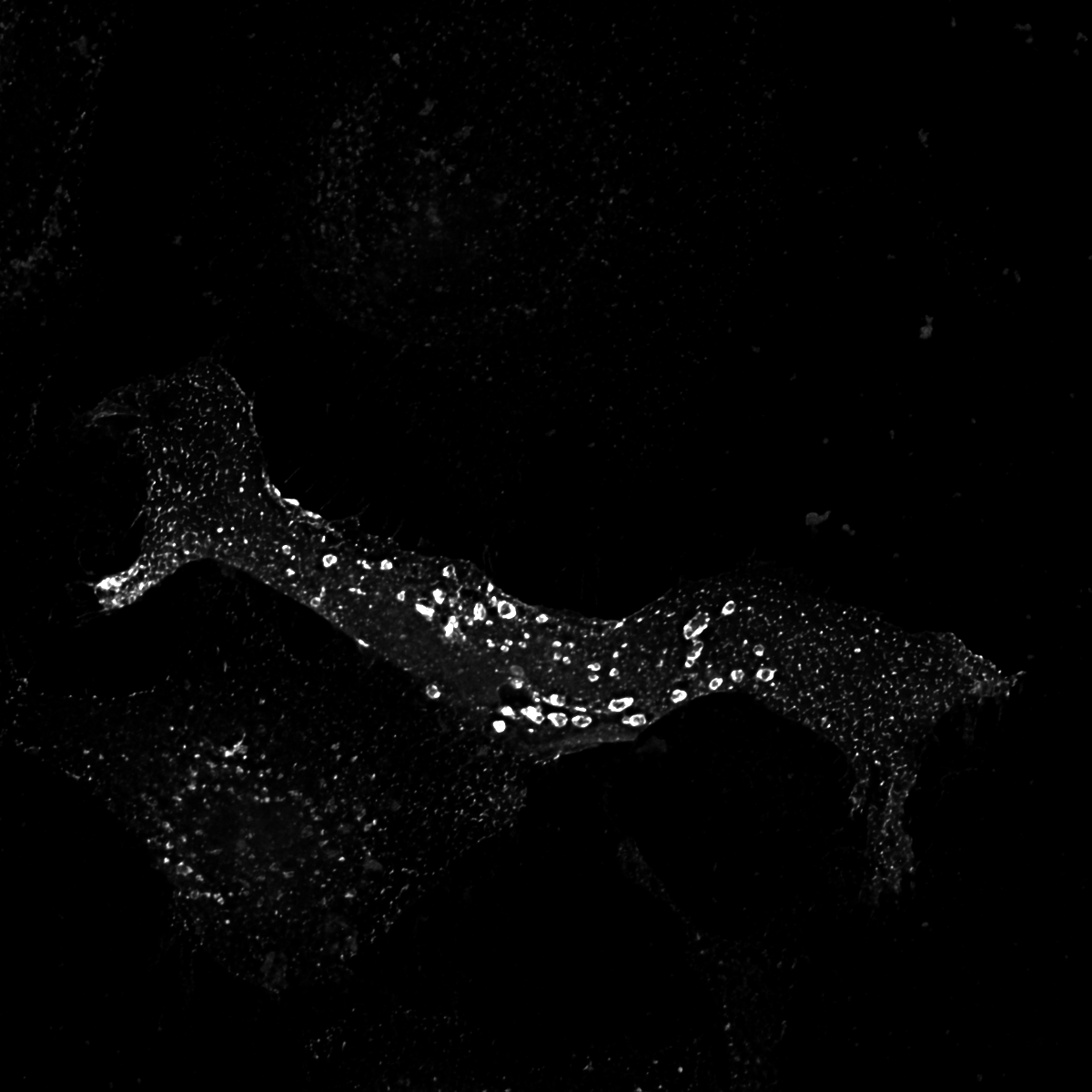

Supplement: Supplementary file 11 — Source data Fig. 9 [file 44318_2025_367_MOESM11_ESM.zip › SD figure 9/9A/Fig_9_A_data/Control KO/RABEX5 WT + RAB5/1_Experiment-173_czi_66d19c1c70998_hrm.ics.tiff]

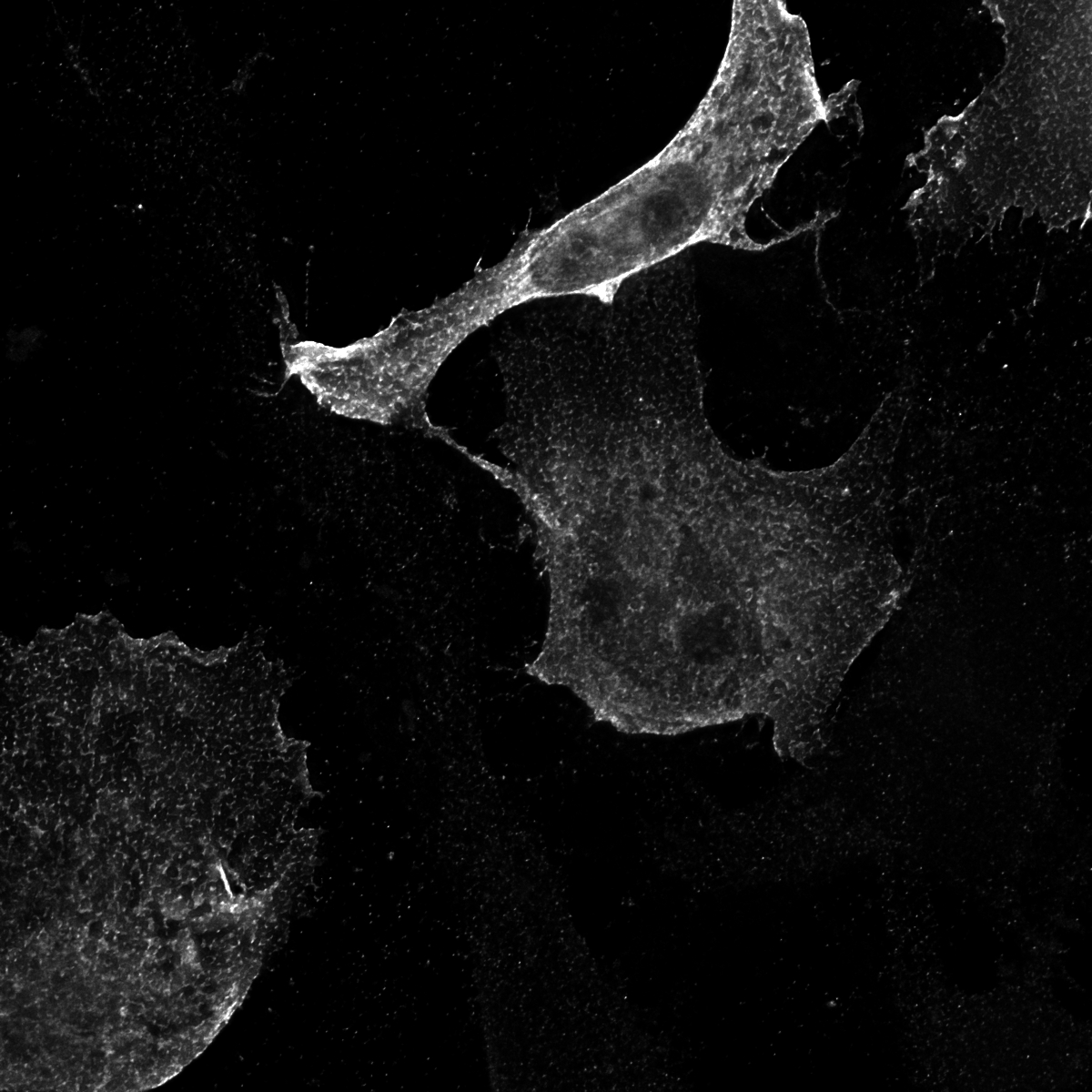

Supplement: Supplementary file 11 — Source data Fig. 9 [file 44318_2025_367_MOESM11_ESM.zip › SD figure 9/9A/Fig_9_A_data/Control KO/RABEX5 A58D and Y25A + RAB5/6_Experiment-192_czi_66d19c1c7490f_hrm.ics.tiff]
