# Supplementary figures and images for: Coordination between ESCRT function and Rab conversion during endosome maturation (part 7 of 9)
Source: EMBO J. 2025 Feb 5;44(6):1574–607. doi: 10.1038/s44318-025-00367-7 (PMC11914609; doi:10.1038/s44318-025-00367-7)

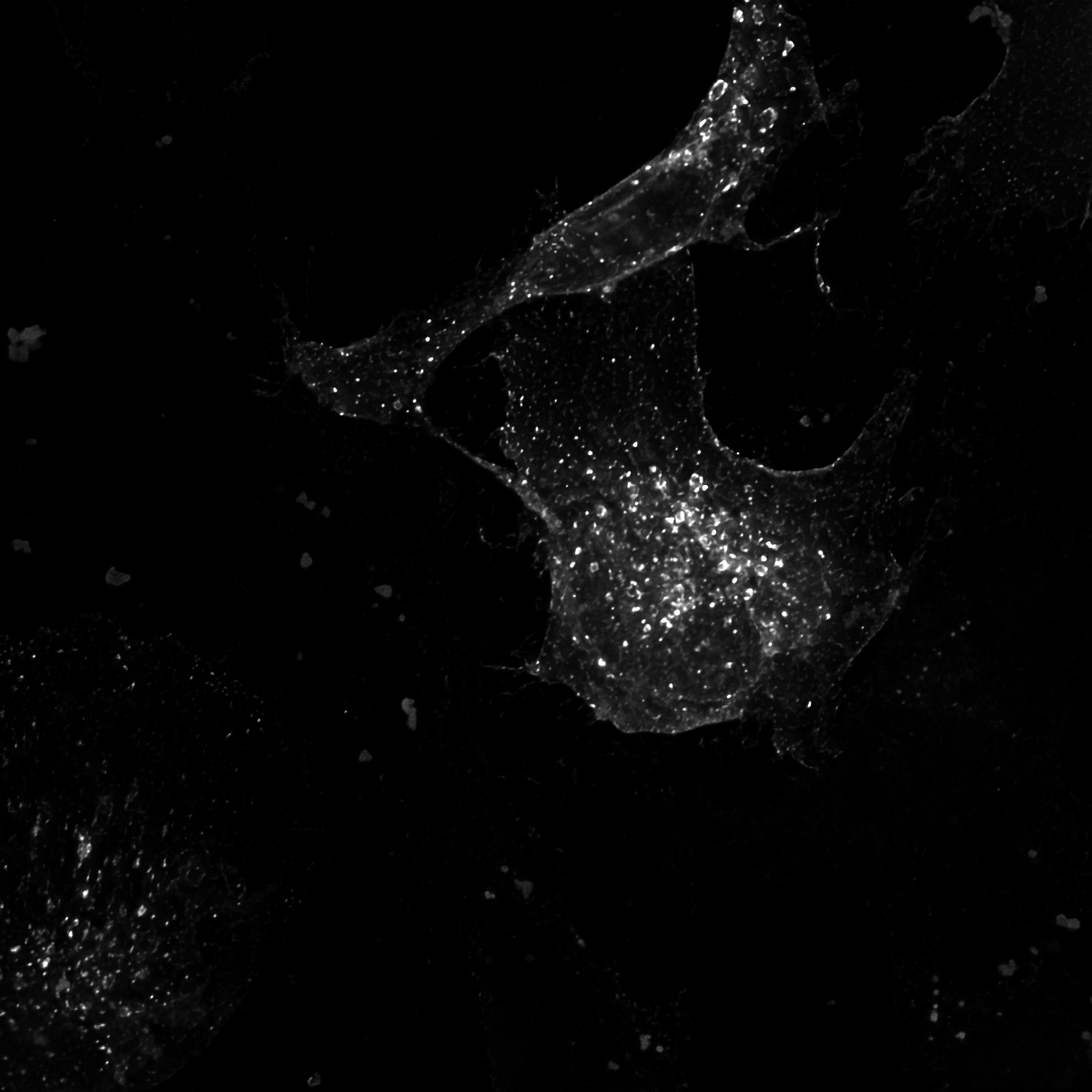

Supplement: Supplementary file 11 — Source data Fig. 9 [file 44318_2025_367_MOESM11_ESM.zip › SD figure 9/9A/Fig_9_A_data/Control KO/RABEX5 A58D and Y25A + RAB5/7_Experiment-192_czi_66d19c1c7490f_hrm.ics.tiff]

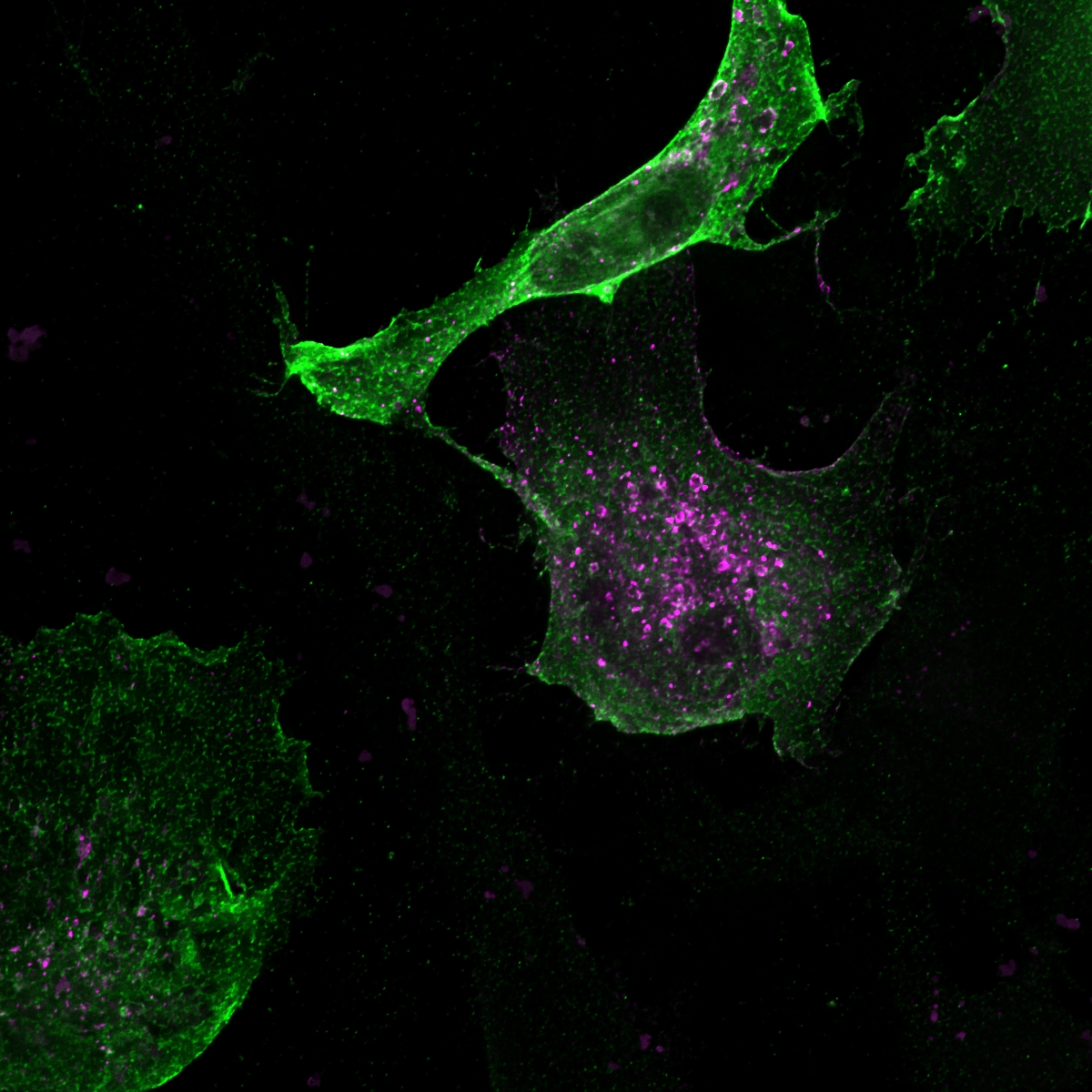

Supplement: Supplementary file 11 — Source data Fig. 9 [file 44318_2025_367_MOESM11_ESM.zip › SD figure 9/9A/Fig_9_A_data/Control KO/RABEX5 A58D and Y25A + RAB5/8_Experiment-192_czi_66d19c1c7490f_hrm.ics.tiff]

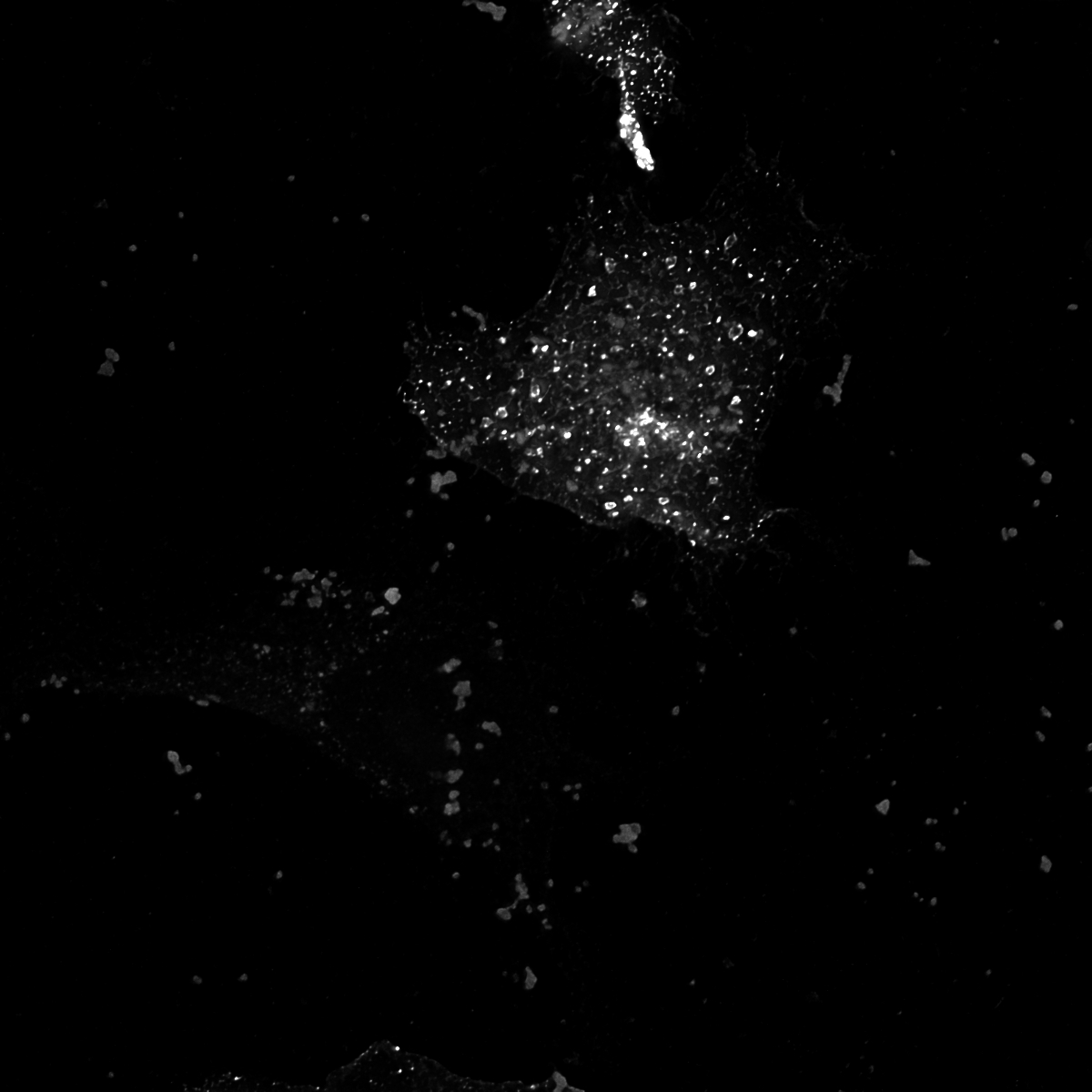

Supplement: Supplementary file 11 — Source data Fig. 9 [file 44318_2025_367_MOESM11_ESM.zip › SD figure 9/9A/Fig_9_A_data/Control KO/RABEX5 A58D + RAB5/4_Experiment-185_czi_66d19c1c7315a_hrm.ics.tiff]

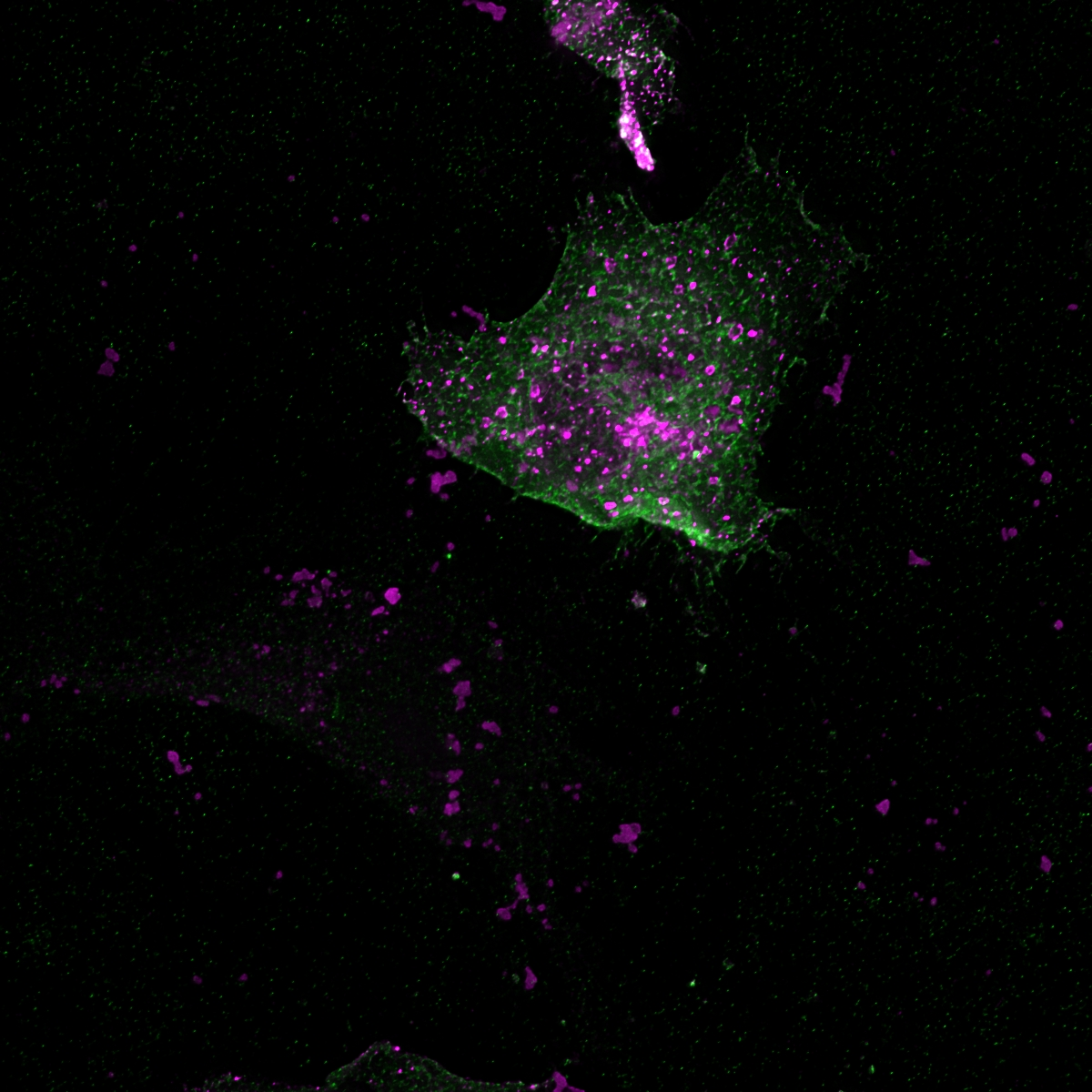

Supplement: Supplementary file 11 — Source data Fig. 9 [file 44318_2025_367_MOESM11_ESM.zip › SD figure 9/9A/Fig_9_A_data/Control KO/RABEX5 A58D + RAB5/5_Experiment-185_czi_66d19c1c7315a_hrm.ics.tiff]

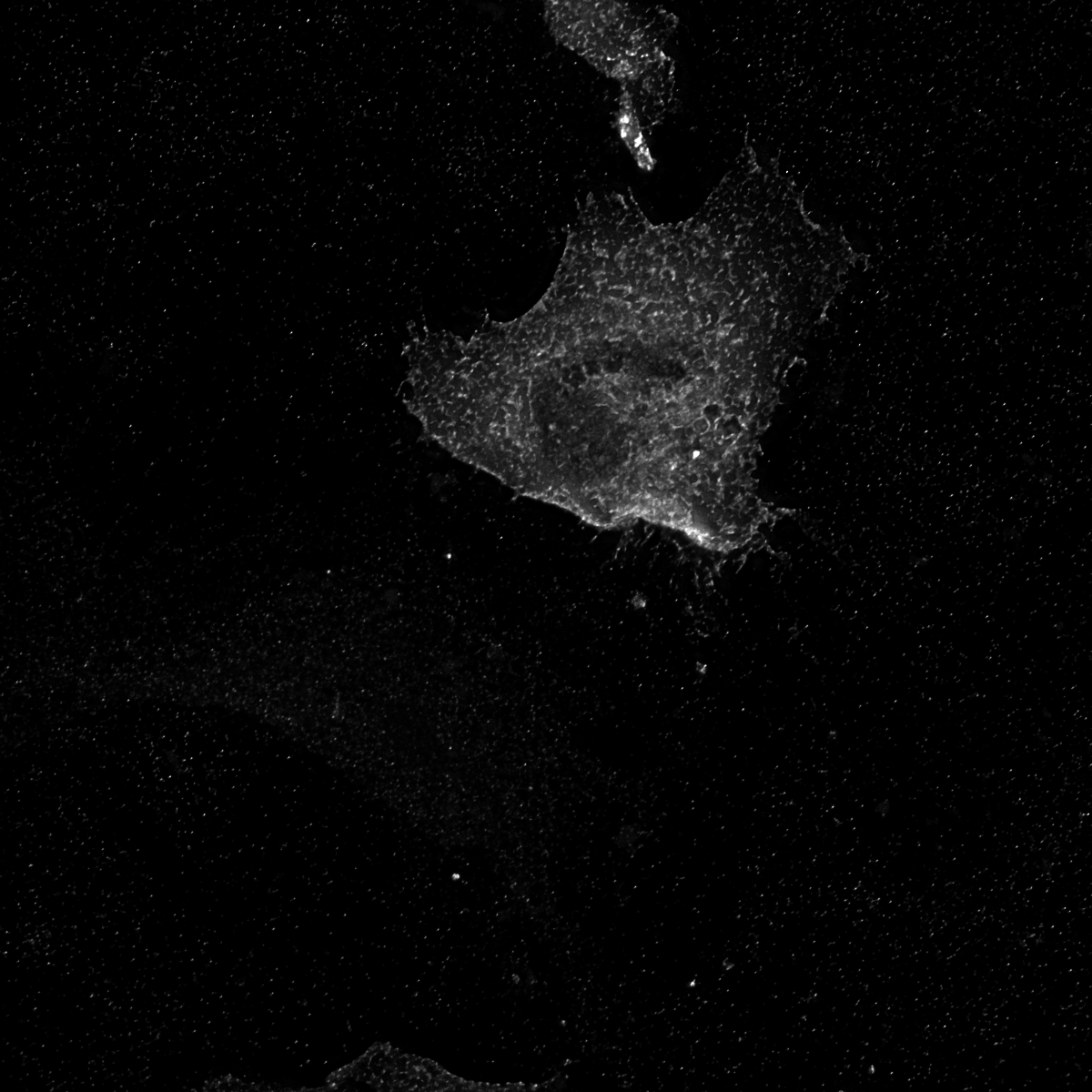

Supplement: Supplementary file 11 — Source data Fig. 9 [file 44318_2025_367_MOESM11_ESM.zip › SD figure 9/9A/Fig_9_A_data/Control KO/RABEX5 A58D + RAB5/3_Experiment-185_czi_66d19c1c7315a_hrm.ics.tiff]

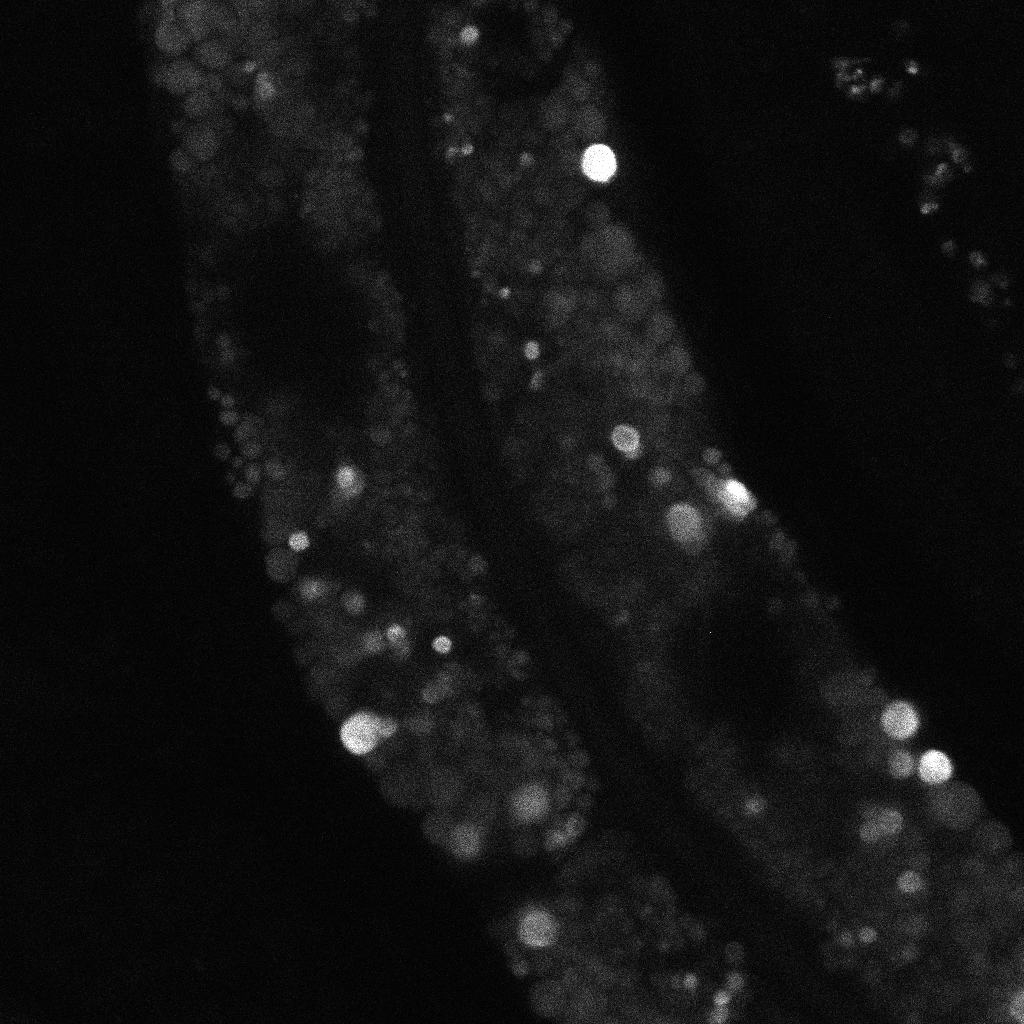

Supplement: Supplementary file 12 — Figure EV Source Data [file 44318_2025_367_MOESM12_ESM.zip › SD EV files/SD figure EV5/EV5A/EV_5_A_data /Mock/A Rab5GFP RAb7mCherrz control RNAi front_0010-1.tif]

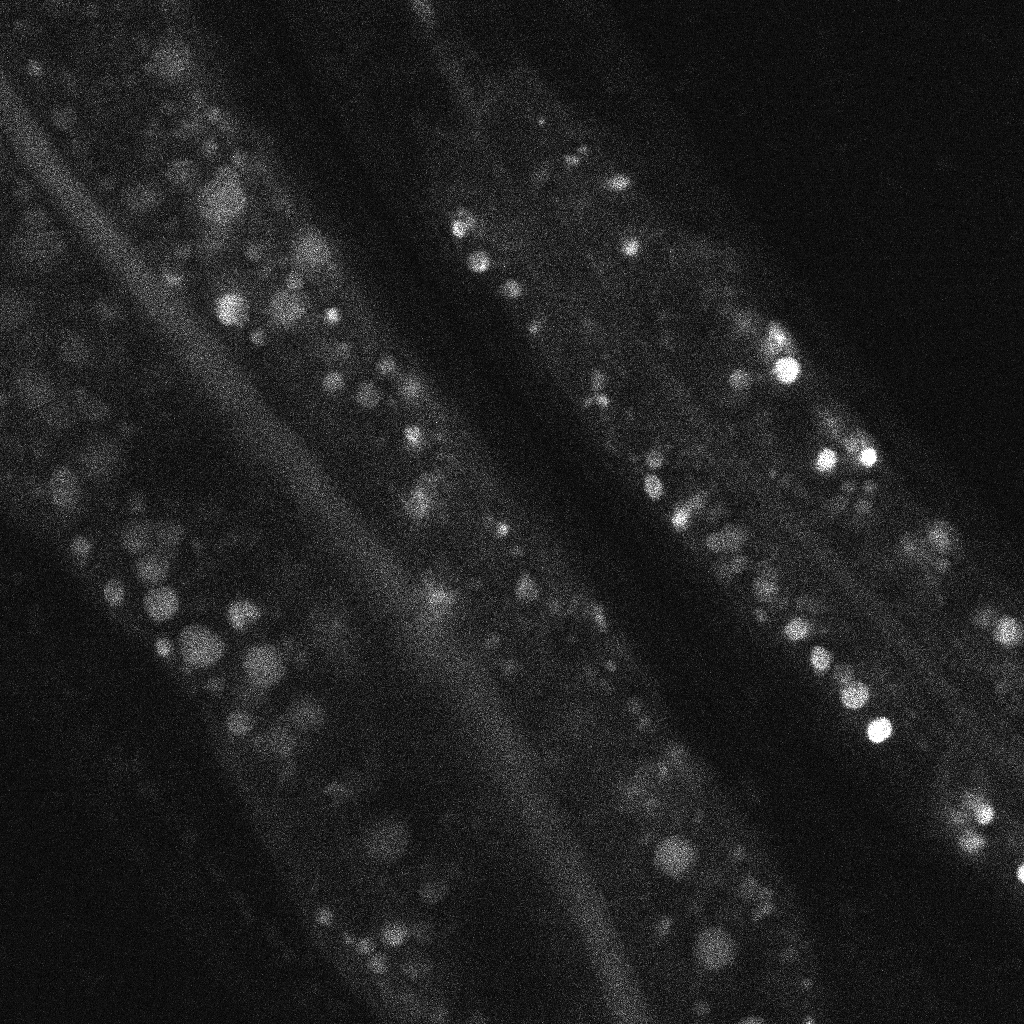

Supplement: Supplementary file 12 — Figure EV Source Data [file 44318_2025_367_MOESM12_ESM.zip › SD EV files/SD figure EV5/EV5A/EV_5_A_data /usp-50 (RNAi)/A Rab5GFP RAB7mCherrz usp50 RNAi front_0020-1.tif]

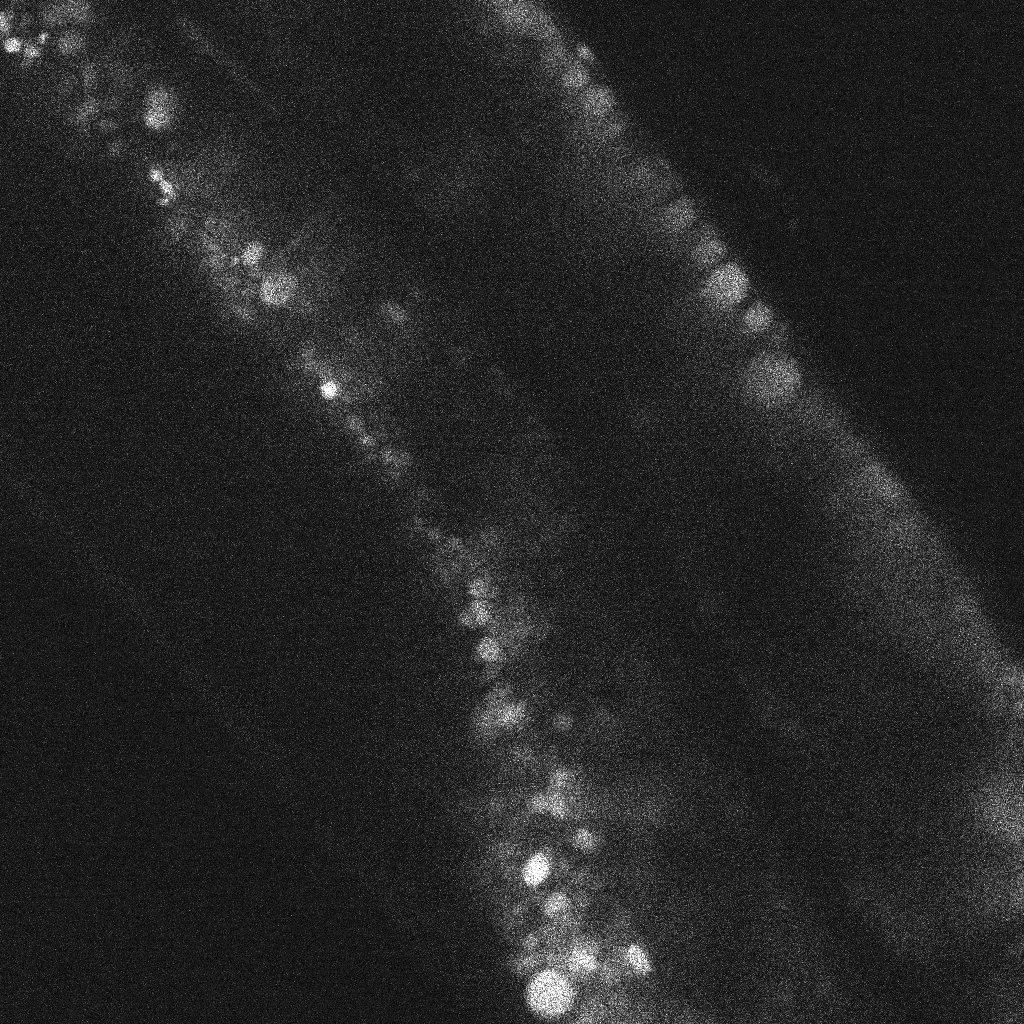

Supplement: Supplementary file 12 — Figure EV Source Data [file 44318_2025_367_MOESM12_ESM.zip › SD EV files/SD figure EV5/EV5A/EV_5_A_data /ubq-1 and control (RNAi)/A RAB5GFP RAB7mCherrz ubq1 and control RNAi 1 to 250 front_0004-1.tif]

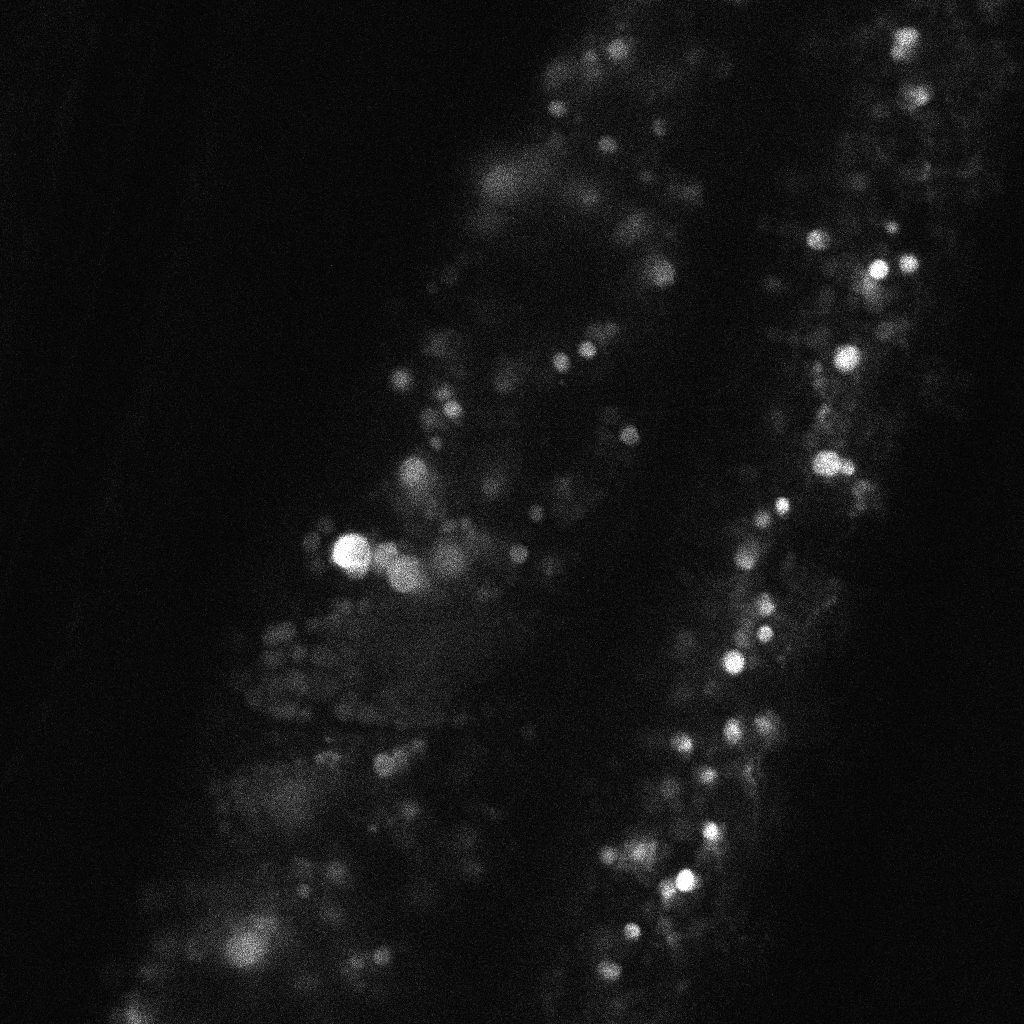

Supplement: Supplementary file 12 — Figure EV Source Data [file 44318_2025_367_MOESM12_ESM.zip › SD EV files/SD figure EV5/EV5C/EV_5_C_data/Mock/A RFPrab5 GFPubq control RNAi front_0013-1.tif]

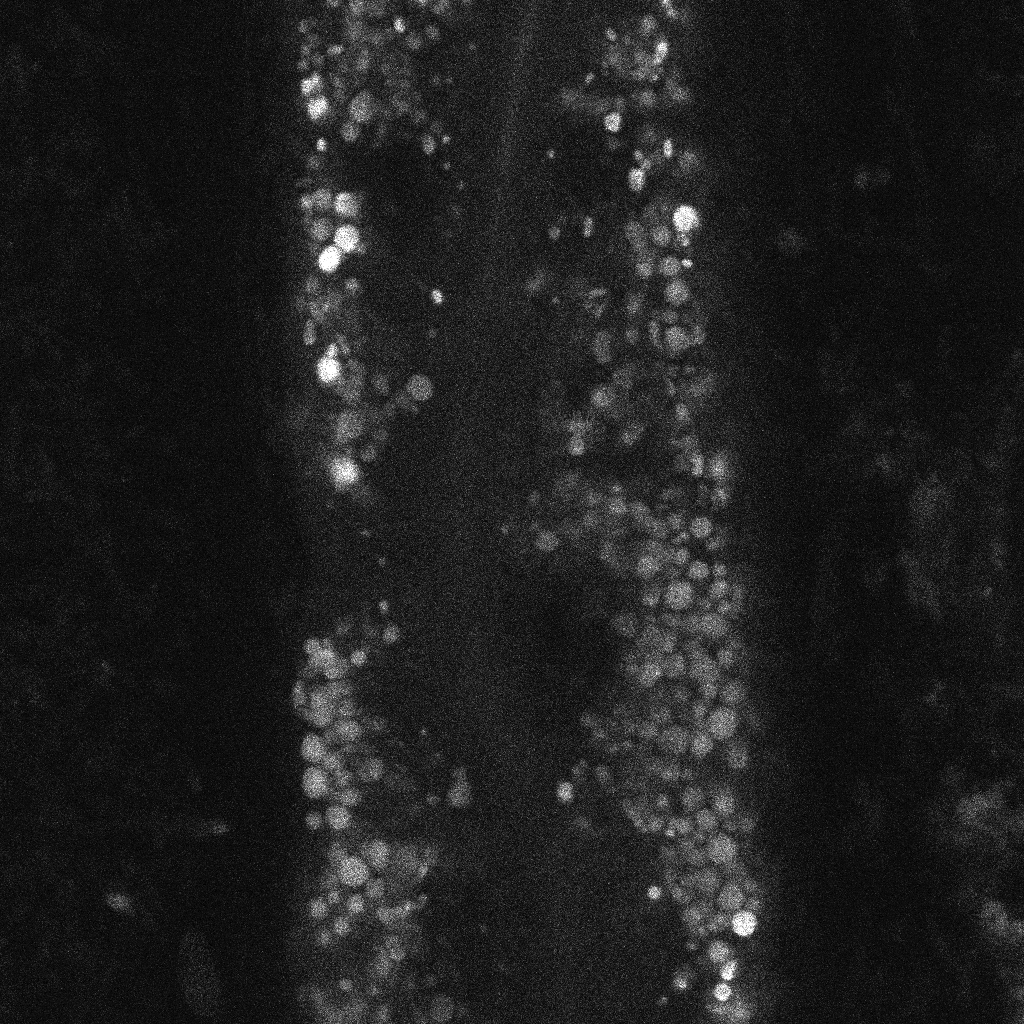

Supplement: Supplementary file 12 — Figure EV Source Data [file 44318_2025_367_MOESM12_ESM.zip › SD EV files/SD figure EV5/EV5C/EV_5_C_data/usp-50 (RNAi)/A RFPrab5 GFPubq usp50 RNAi front_0007-1.tif]

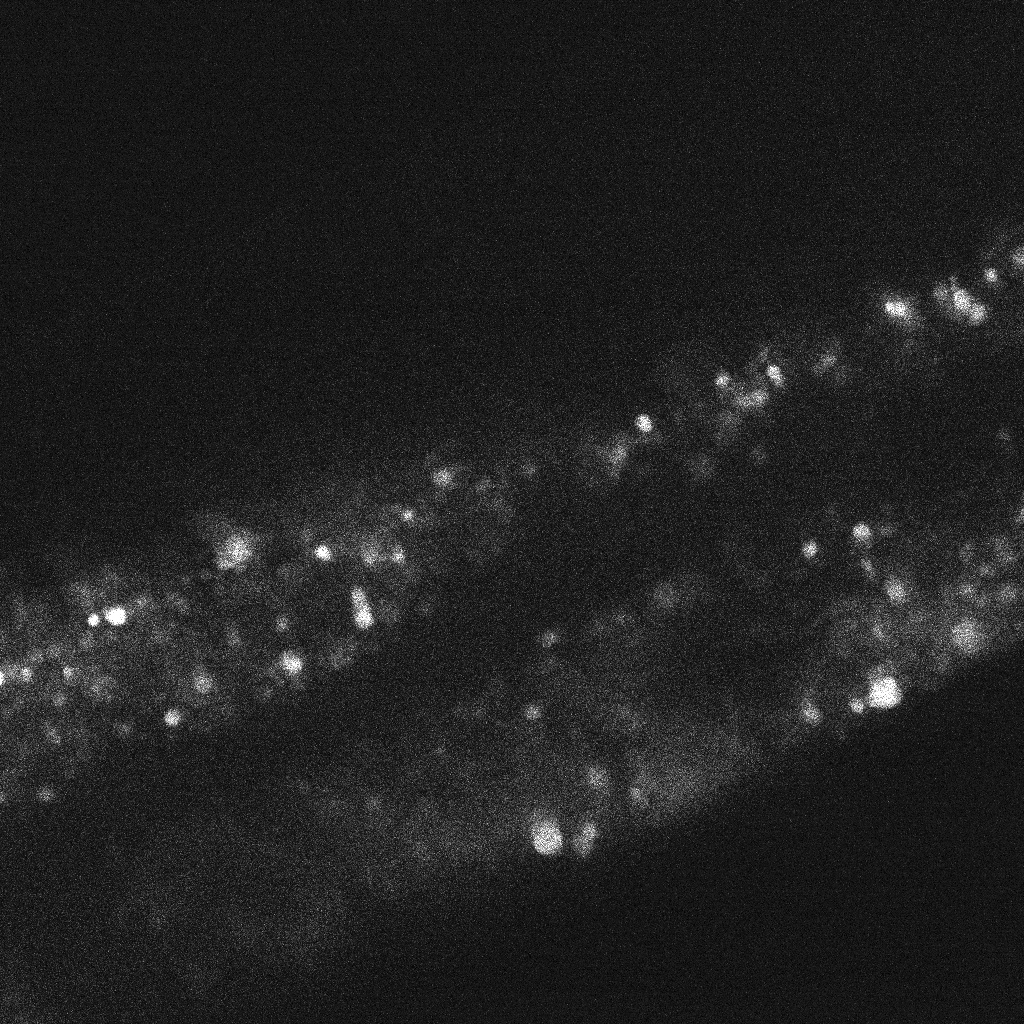

Supplement: Supplementary file 12 — Figure EV Source Data [file 44318_2025_367_MOESM12_ESM.zip › SD EV files/SD figure EV5/EV5C/EV_5_C_data/ubq-1 and control (RNAi)/A RFPrab5 GFPubq ubq1 and control RNAi 1 to 250 front_0010-1.tif]

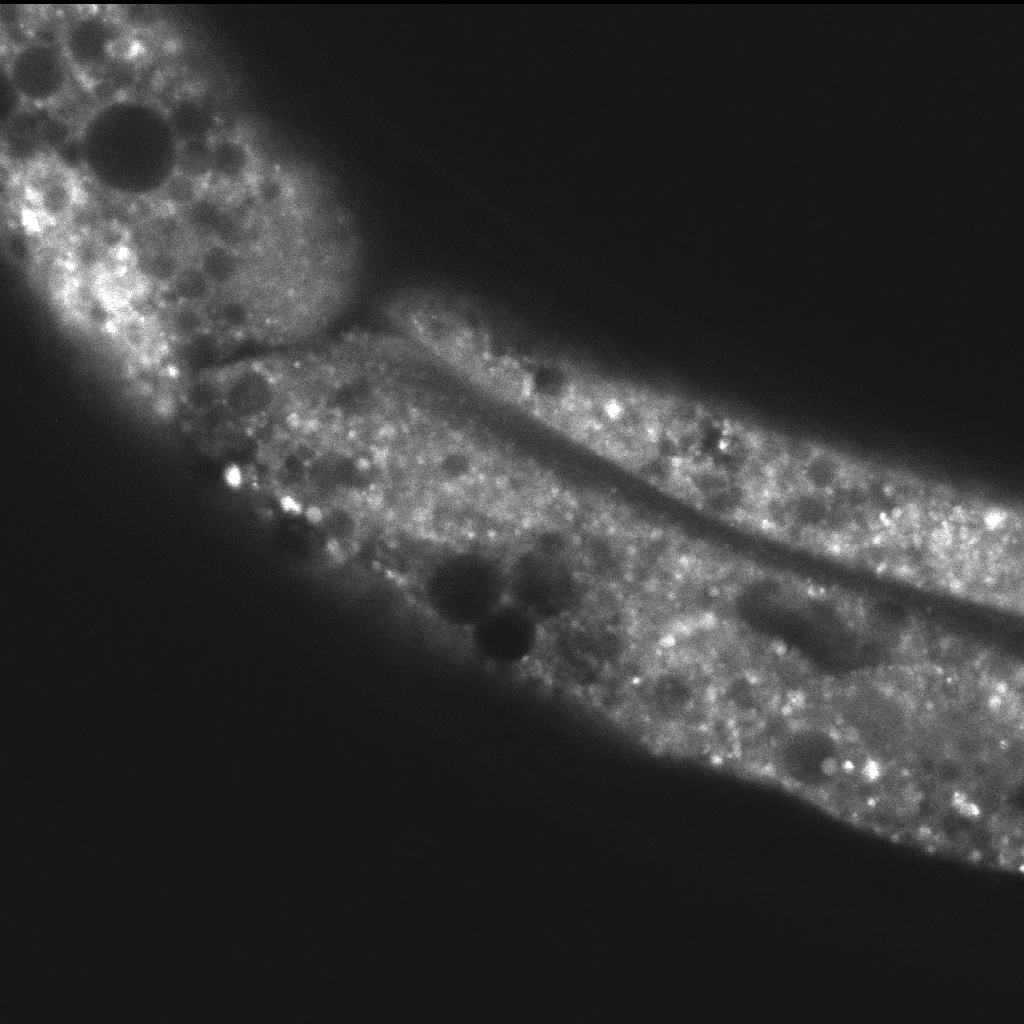

Supplement: Supplementary file 12 — Figure EV Source Data [file 44318_2025_367_MOESM12_ESM.zip › SD EV files/SD figure EV1/EV1A/EV_1_A_data/vps-32.1 (RNAi)/A rab5 rab7 vps 32.1 rnai front_0001-1.tif]

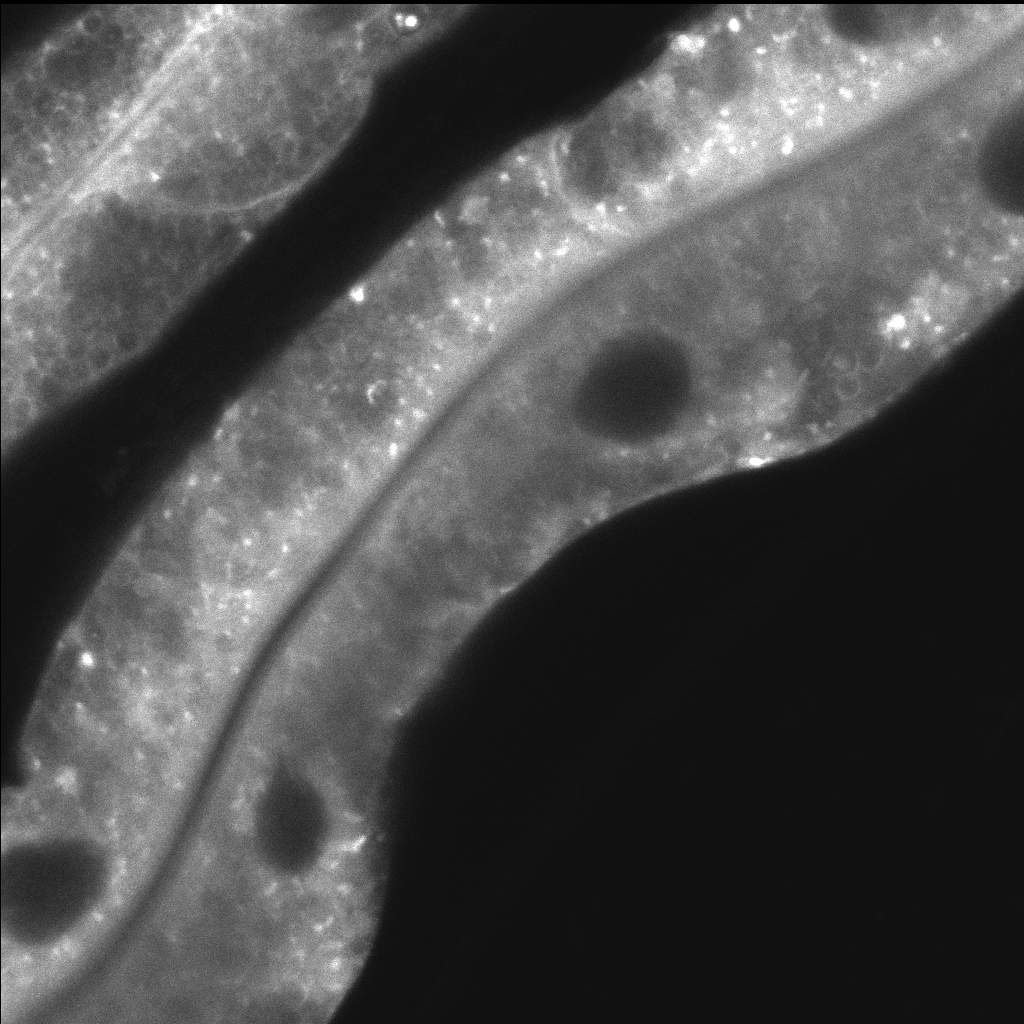

Supplement: Supplementary file 12 — Figure EV Source Data [file 44318_2025_367_MOESM12_ESM.zip › SD EV files/SD figure EV1/EV1A/EV_1_A_data/Mock/A rab5&rab7 control rnai front_0009-1.tif]

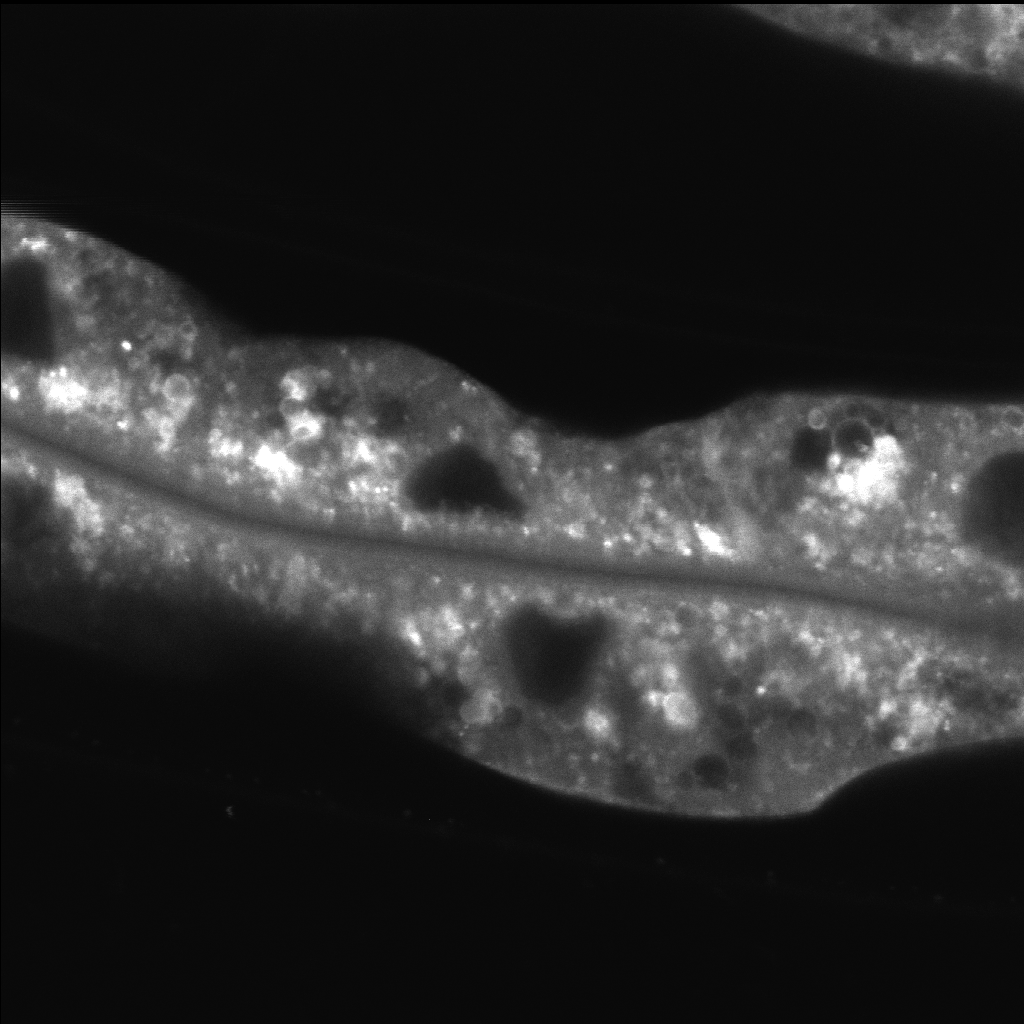

Supplement: Supplementary file 12 — Figure EV Source Data [file 44318_2025_367_MOESM12_ESM.zip › SD EV files/SD figure EV1/EV1A/EV_1_A_data/vps-28 (RNAi)/A rab5&rab7 vps28 rnai front_0005-1.tif]

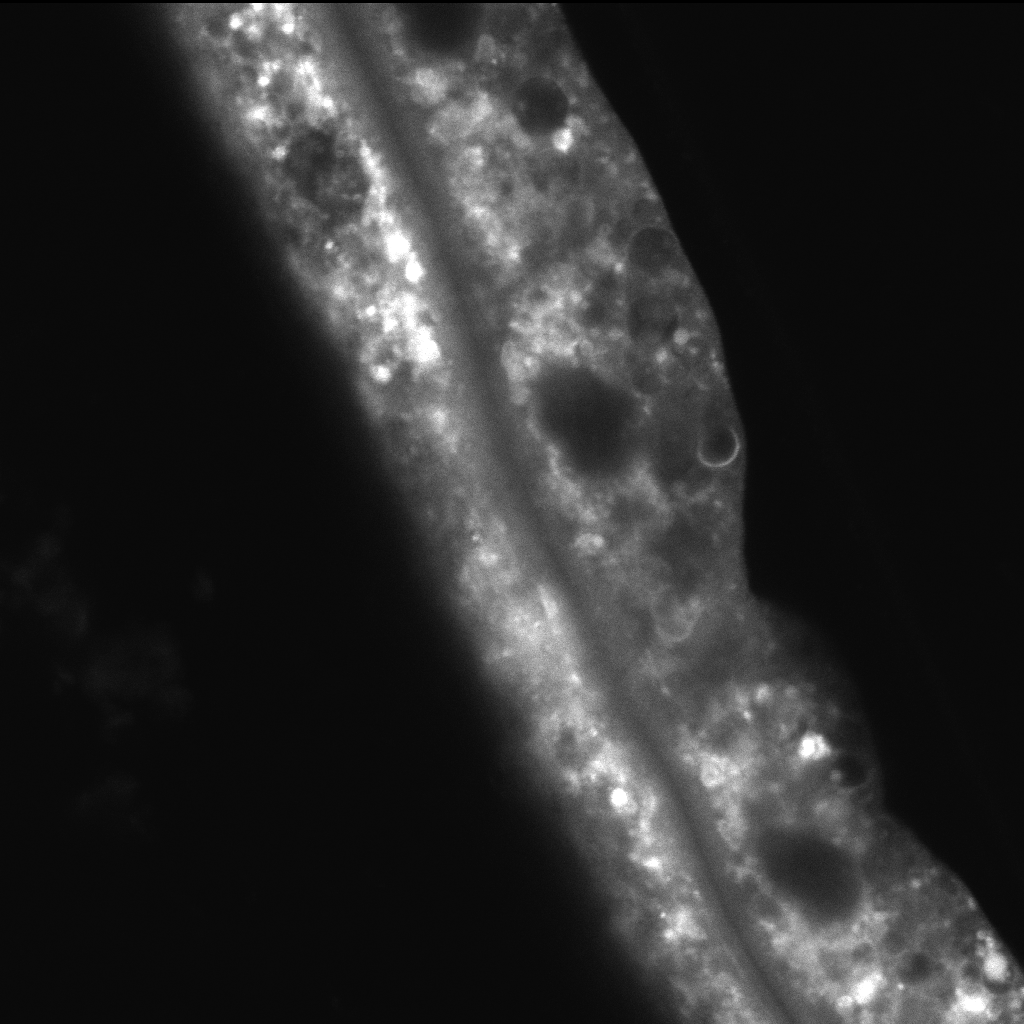

Supplement: Supplementary file 12 — Figure EV Source Data [file 44318_2025_367_MOESM12_ESM.zip › SD EV files/SD figure EV1/EV1A/EV_1_A_data/vps-24 (RNAi)/A rab 5 rab7 vps24 rnai front_0005-1.tif]

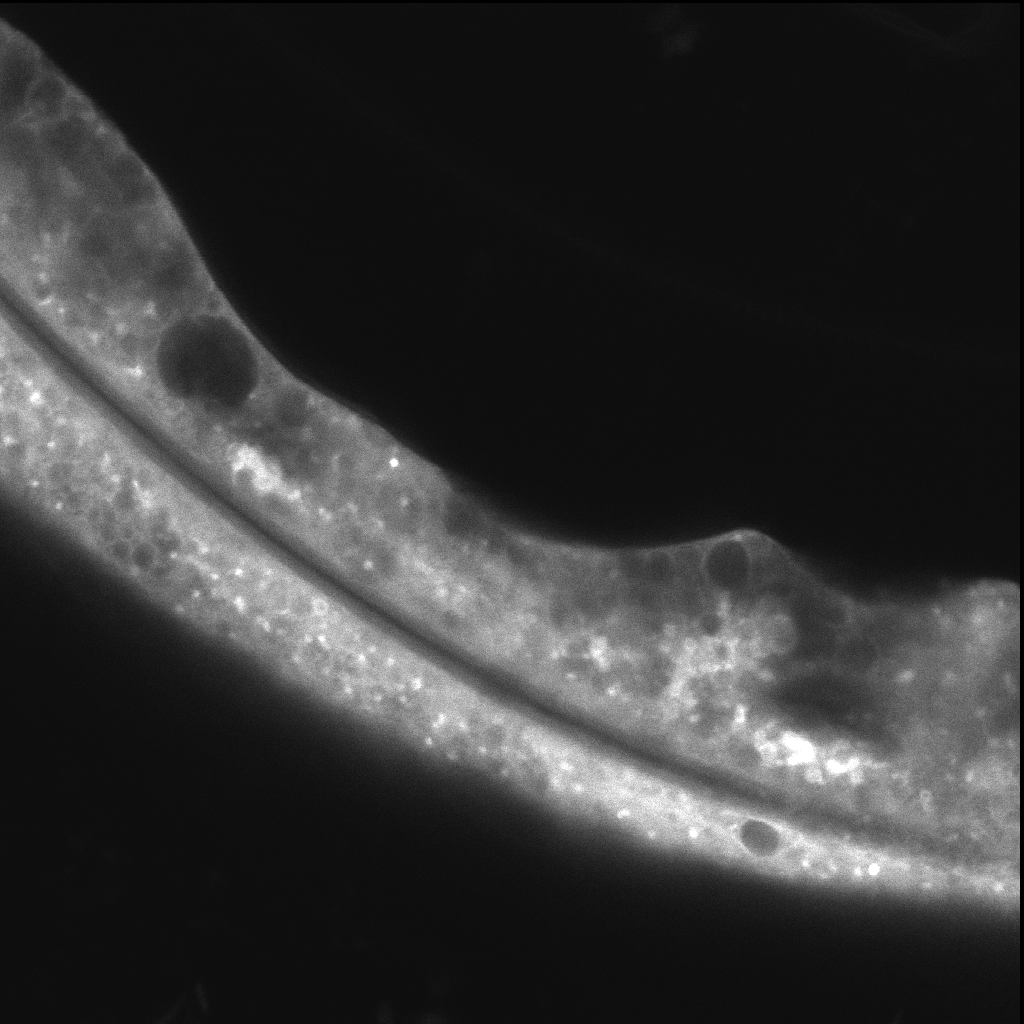

Supplement: Supplementary file 12 — Figure EV Source Data [file 44318_2025_367_MOESM12_ESM.zip › SD EV files/SD figure EV1/EV1A/EV_1_A_data/vps-60 (RNAi)/A rab5 rab7 vps60 rnai front_0005-1.tif]

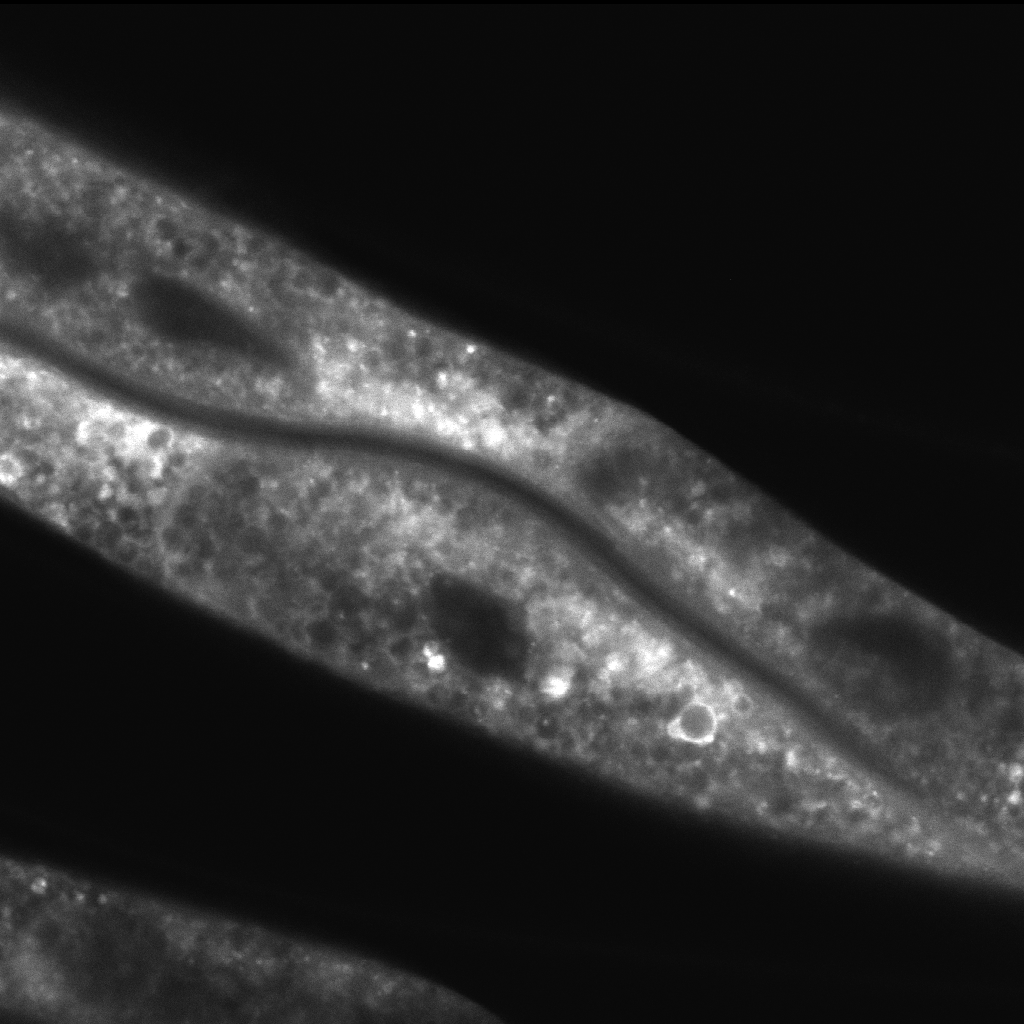

Supplement: Supplementary file 12 — Figure EV Source Data [file 44318_2025_367_MOESM12_ESM.zip › SD EV files/SD figure EV1/EV1A/EV_1_A_data/did-2 (RNAi)/A rab5 rab7 did2 rnai front_0006-1.tif]

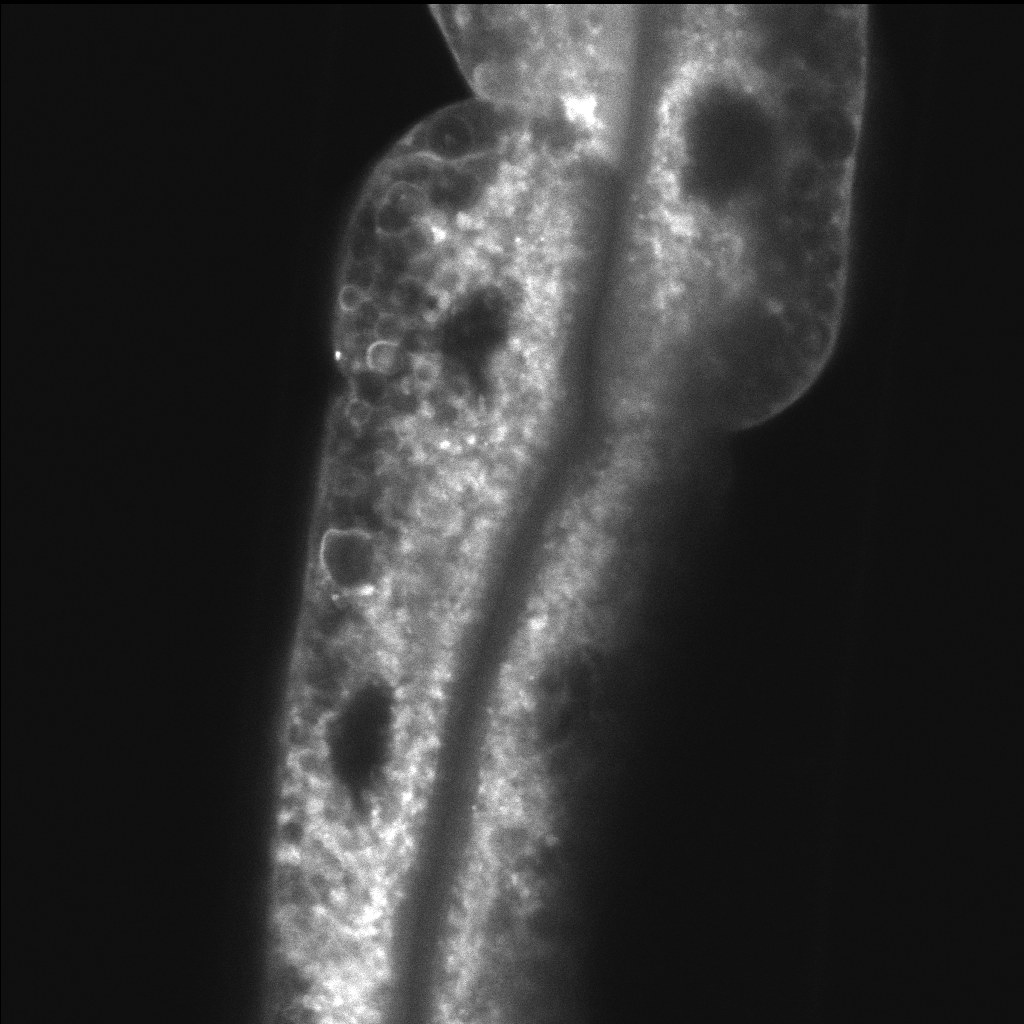

Supplement: Supplementary file 12 — Figure EV Source Data [file 44318_2025_367_MOESM12_ESM.zip › SD EV files/SD figure EV1/EV1A/EV_1_A_data/vps-37 (RNAi)/A rab5 rab7 vps37 rnai front_0002-1.tif]

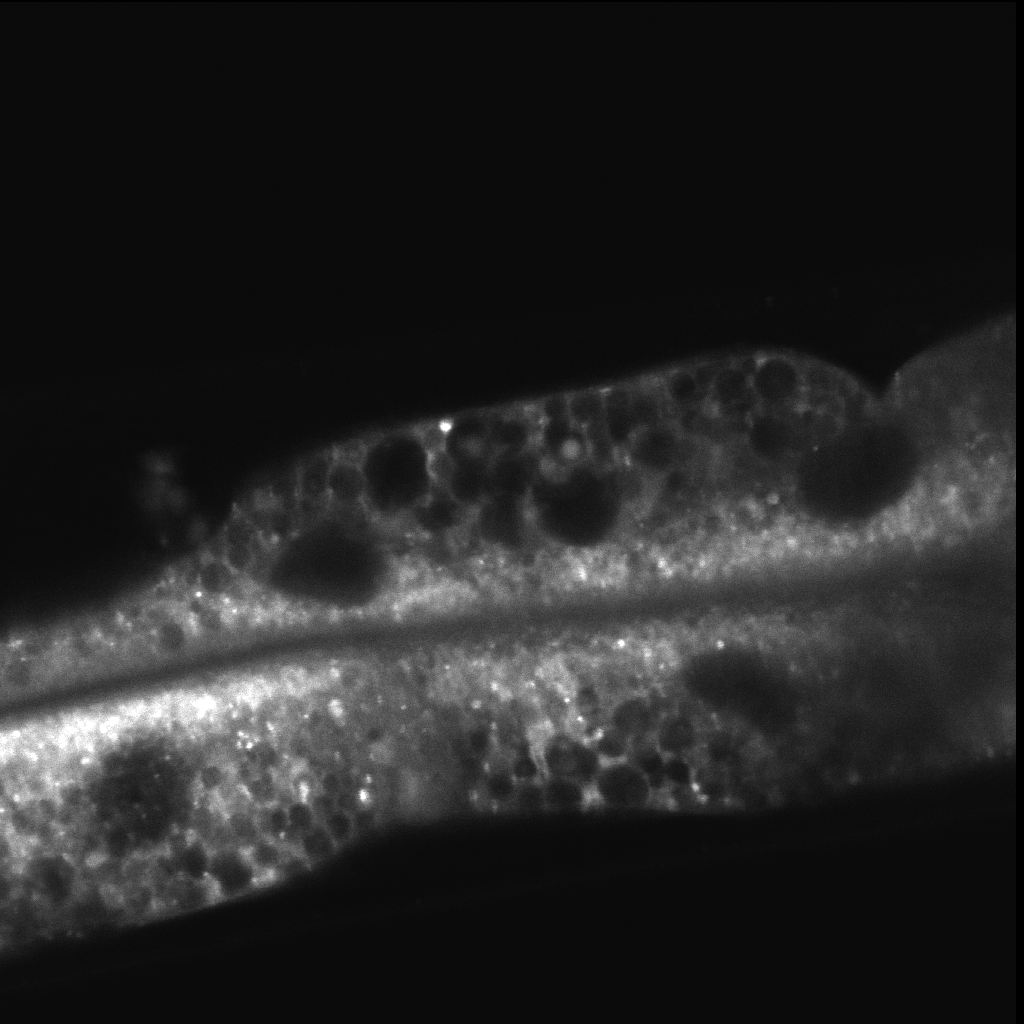

Supplement: Supplementary file 12 — Figure EV Source Data [file 44318_2025_367_MOESM12_ESM.zip › SD EV files/SD figure EV1/EV1B/EV_1_B_data/did-2 (RNAi) pre fed/A rab5 raby sand1 did2 rnai preefed front_0003-1-1.tif]

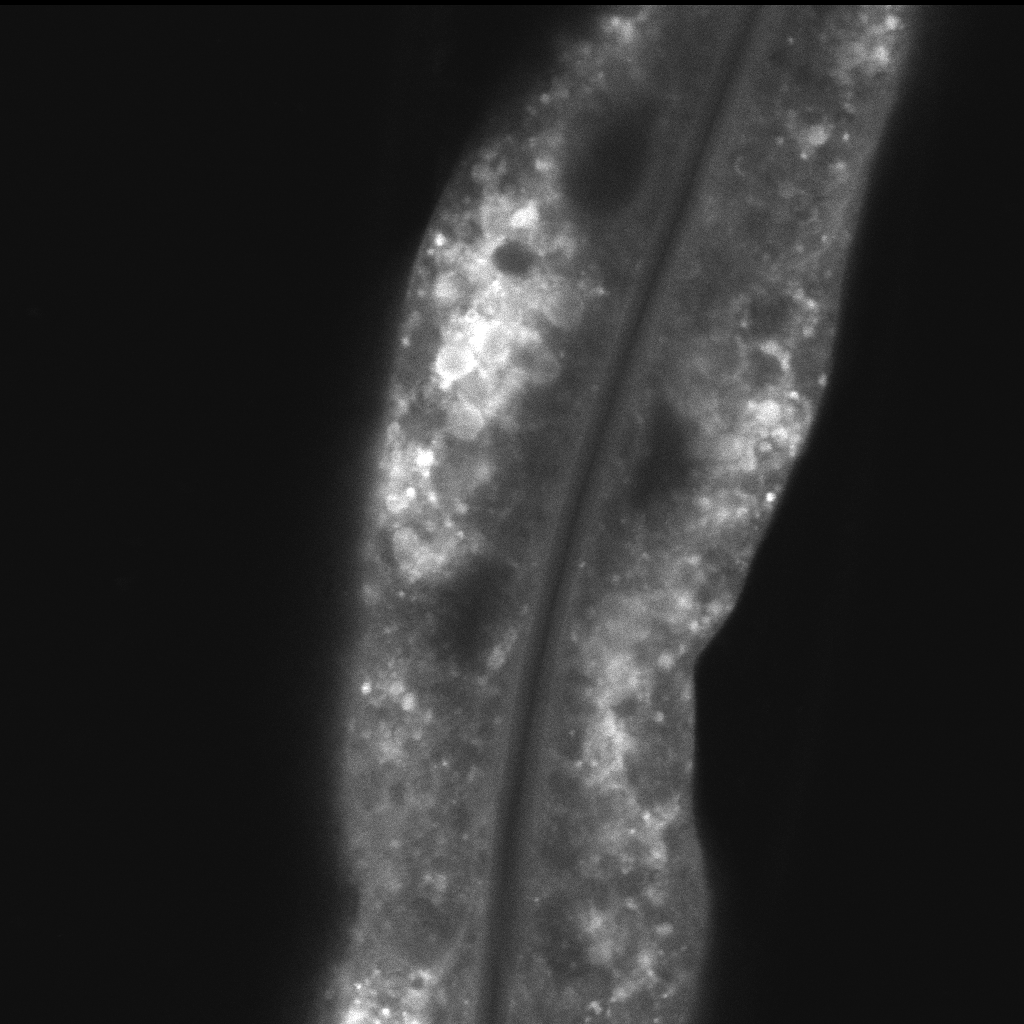

Supplement: Supplementary file 12 — Figure EV Source Data [file 44318_2025_367_MOESM12_ESM.zip › SD EV files/SD figure EV1/EV1B/EV_1_B_data/Mock/A rab5 rab7 sand1 control rnai front_0006-1.tif]

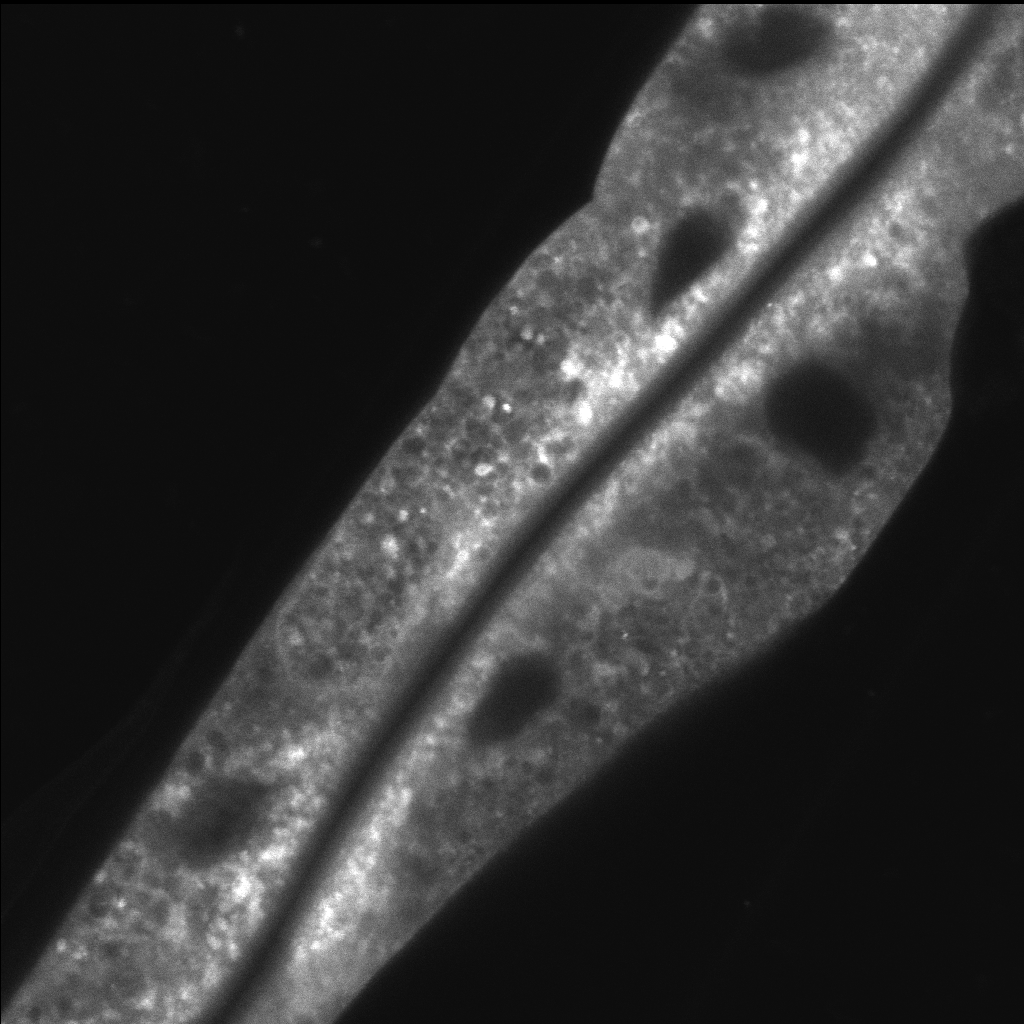

Supplement: Supplementary file 12 — Figure EV Source Data [file 44318_2025_367_MOESM12_ESM.zip › SD EV files/SD figure EV1/EV1B/EV_1_B_data/vps-28 (RNAi)/A rab5&rab7 sand1 vps28 rnai front_0009-1.tif]

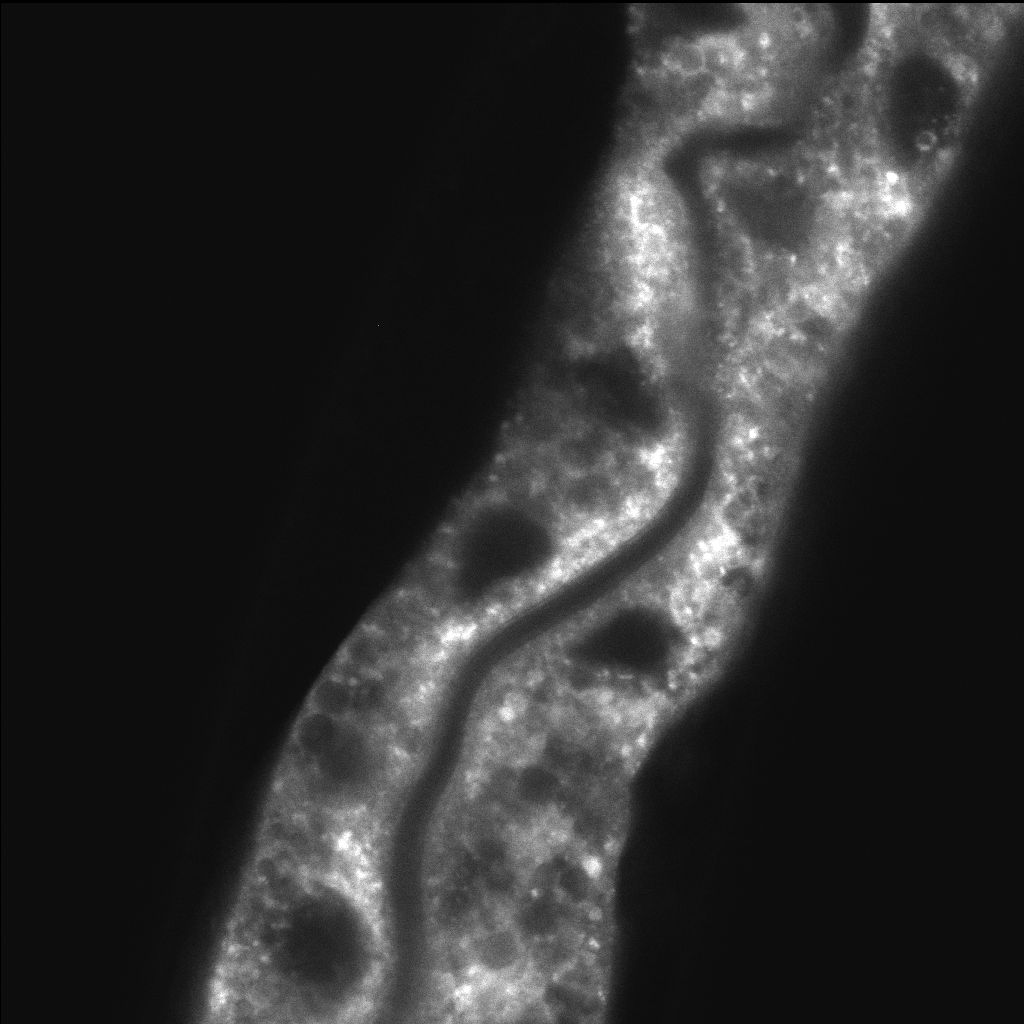

Supplement: Supplementary file 12 — Figure EV Source Data [file 44318_2025_367_MOESM12_ESM.zip › SD EV files/SD figure EV1/EV1B/EV_1_B_data/vps-24 (RNAi)/A rab5 rab7 sand1 vps24 rnai front_0001-1-1.tif]

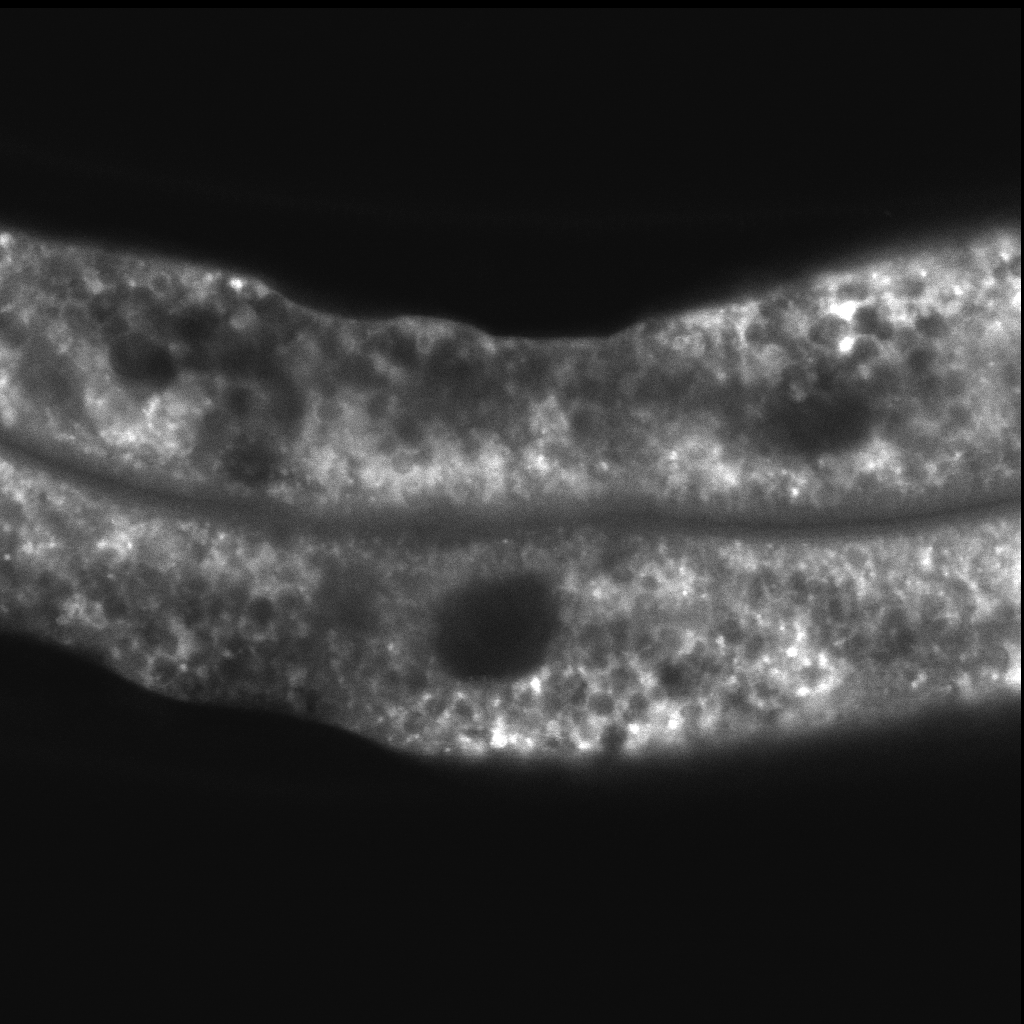

Supplement: Supplementary file 12 — Figure EV Source Data [file 44318_2025_367_MOESM12_ESM.zip › SD EV files/SD figure EV1/EV1B/EV_1_B_data/vps-32.1 (RNAi) pre fed/A rab5 rab7 sand1 pre fed vps32.1 rnai front_0005-1-1.tif]

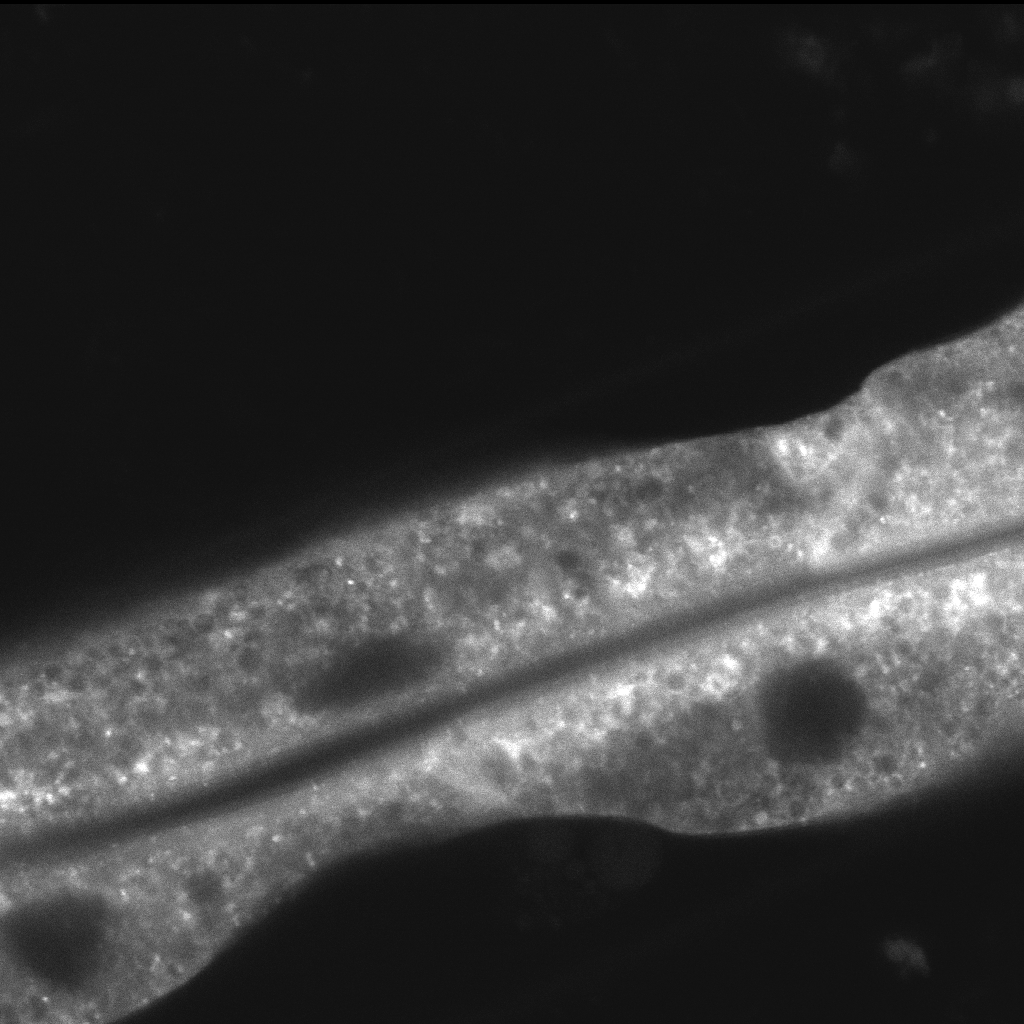

Supplement: Supplementary file 12 — Figure EV Source Data [file 44318_2025_367_MOESM12_ESM.zip › SD EV files/SD figure EV1/EV1B/EV_1_B_data/vps-60 (RNAi)/A rab5 rab7 sand1 vps60 rnai front_0009-1.tif]

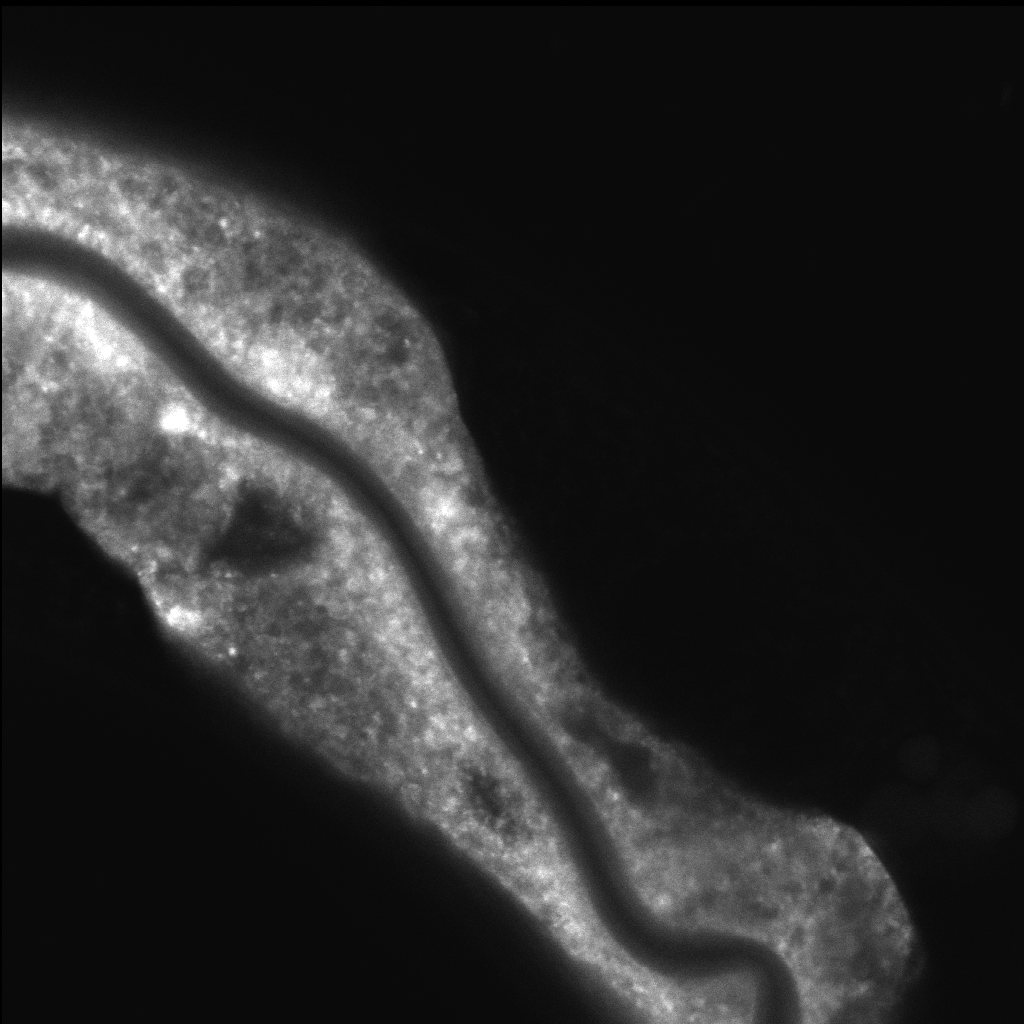

Supplement: Supplementary file 12 — Figure EV Source Data [file 44318_2025_367_MOESM12_ESM.zip › SD EV files/SD figure EV1/EV1B/EV_1_B_data/vps-37 (RNAi)/A rab5 rab7 sand1 vps37 rnai front_0007-1.tif]

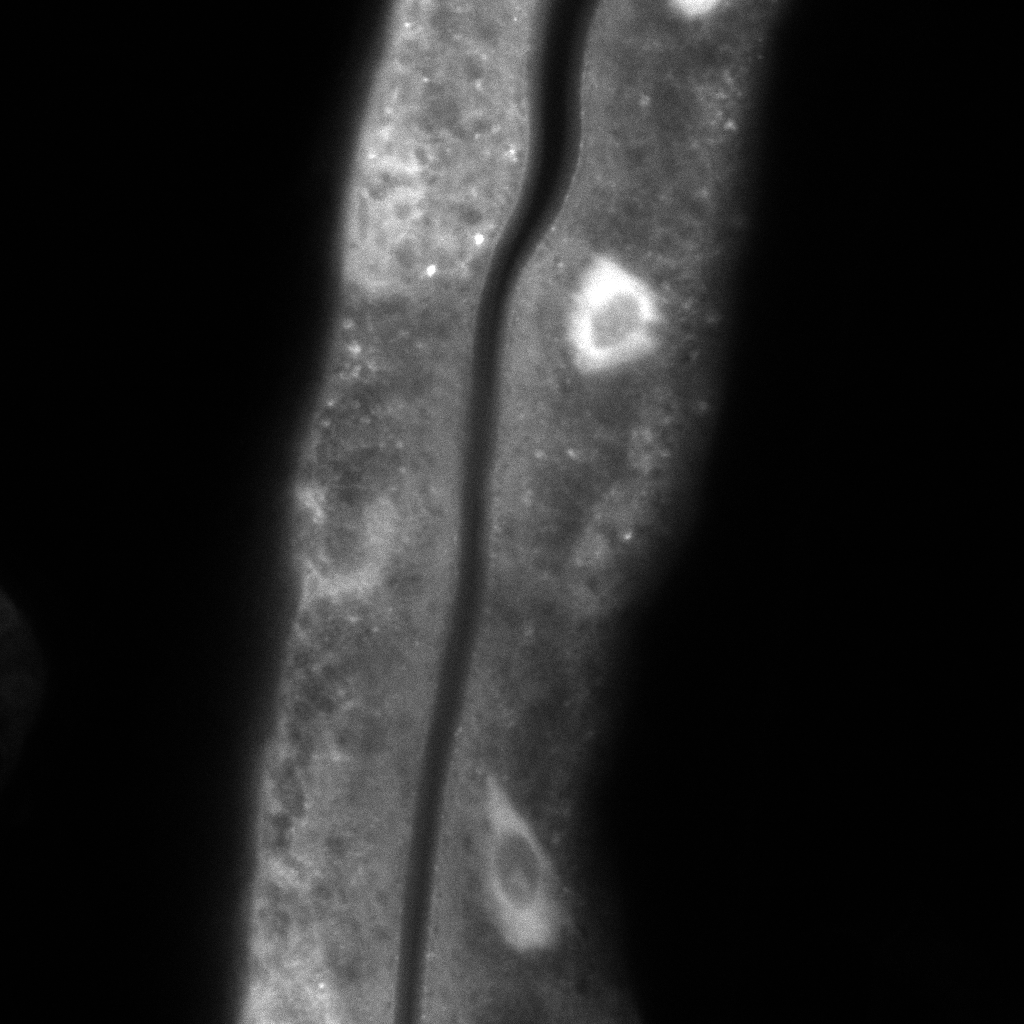

Supplement: Supplementary file 12 — Figure EV Source Data [file 44318_2025_367_MOESM12_ESM.zip › SD EV files/SD figure EV3/EV3A/EV_3_A_data/Mock/A ERT261 GFPubq control RNAi front_0003-1.tif]

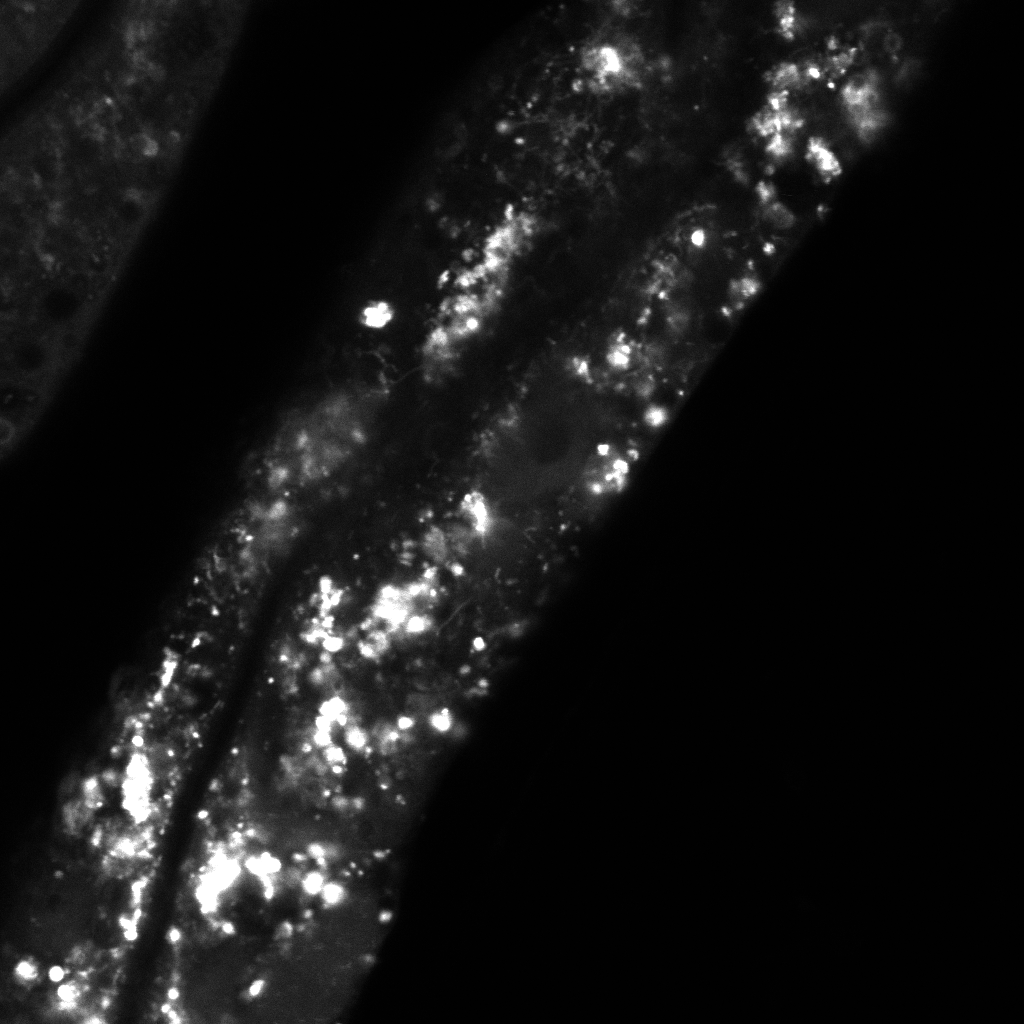

Supplement: Supplementary file 12 — Figure EV Source Data [file 44318_2025_367_MOESM12_ESM.zip › SD EV files/SD figure EV3/EV3A/EV_3_A_data/usp-50 (RNAi)/A ERT261 GFPubq usp50 RNAi front_0013-1.tif]

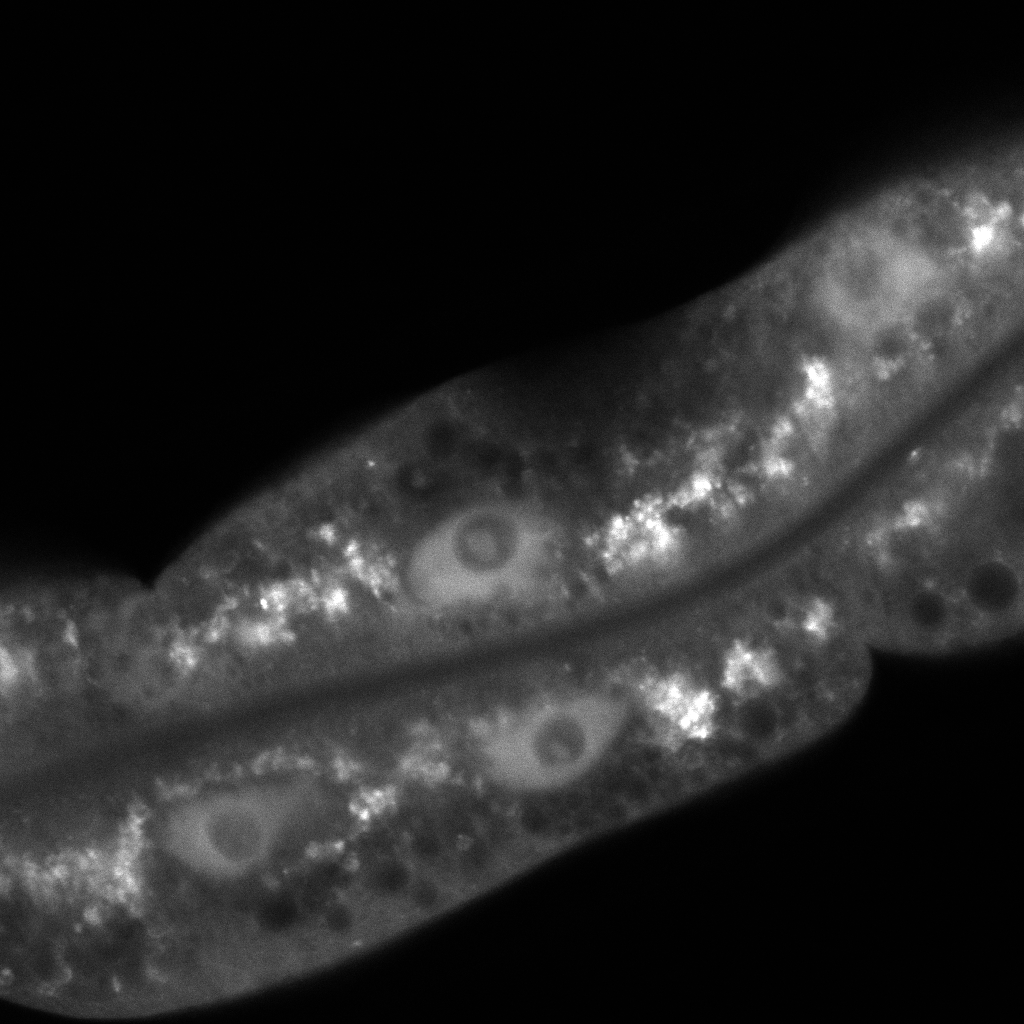

Supplement: Supplementary file 12 — Figure EV Source Data [file 44318_2025_367_MOESM12_ESM.zip › SD EV files/SD figure EV3/EV3A/EV_3_A_data/did-2 (RNAi)/A ERT261 GFPubq did2 RNAi front_0018-1.tif]

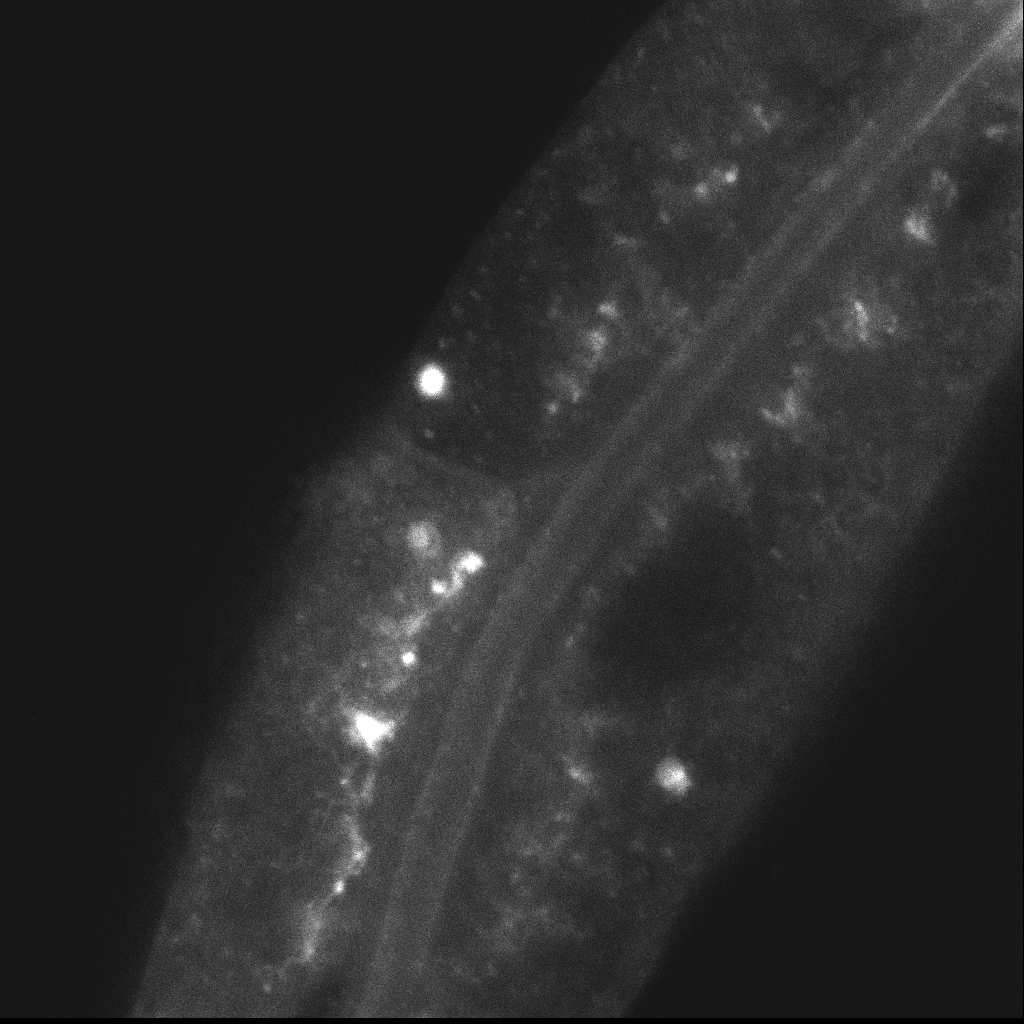

Supplement: Supplementary file 12 — Figure EV Source Data [file 44318_2025_367_MOESM12_ESM.zip › SD EV files/SD figure EV3/EV3D/EV_3_D_data/Mock/GFPvps27 RFPrab5 control RNAi front_0016 aligned.tif]

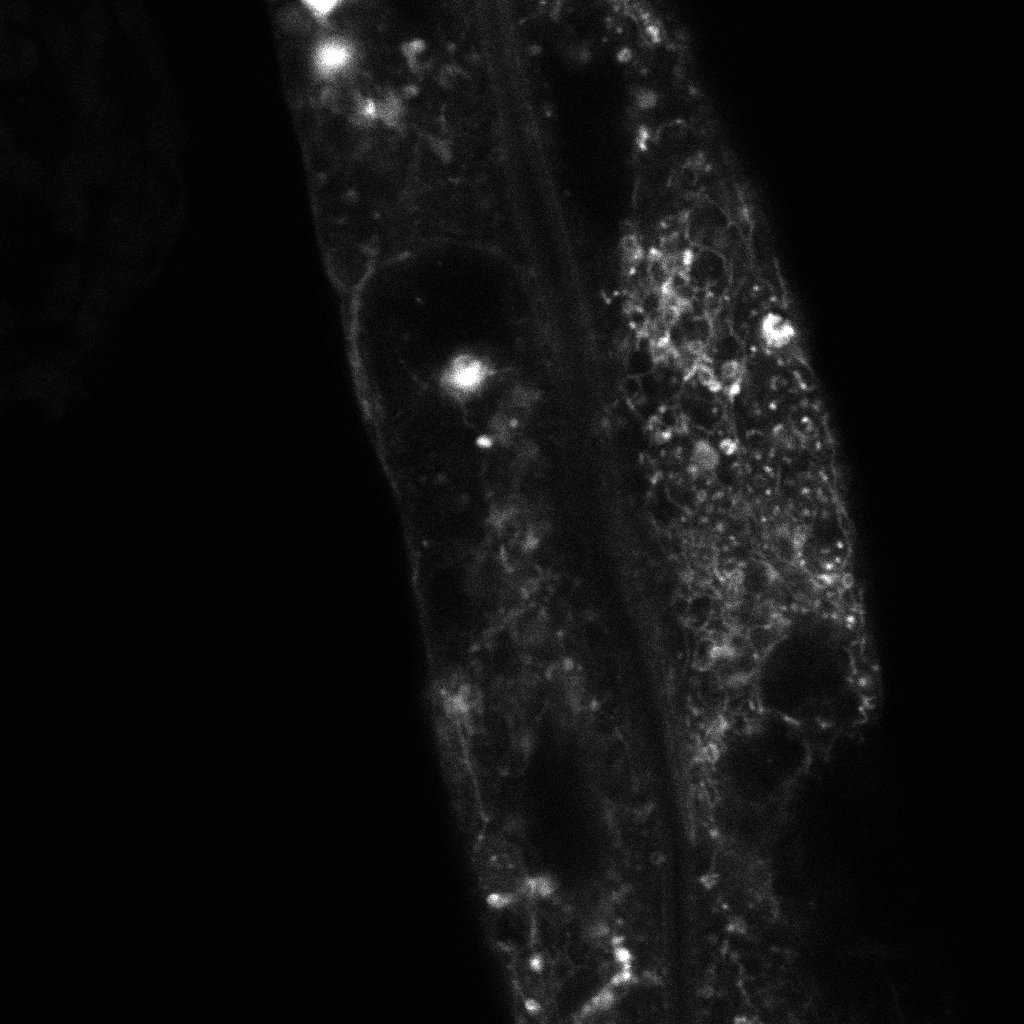

Supplement: Supplementary file 12 — Figure EV Source Data [file 44318_2025_367_MOESM12_ESM.zip › SD EV files/SD figure EV3/EV3D/EV_3_D_data/usp-50 (RNAi)/GFPvps27 RFPrab5 usp50 RNAi front_0009-1net.tif]

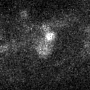

Supplement: Supplementary file 12 — Figure EV Source Data [file 44318_2025_367_MOESM12_ESM.zip › SD EV files/SD figure EV5/EV5A/EV_5_A_Roi /Mock/Gut close up/mCherry ART C MC Rab5GFP RAb7mCherrz control RNAi front_0010-1-1-1-1-1.tif]

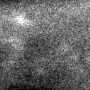

Supplement: Supplementary file 12 — Figure EV Source Data [file 44318_2025_367_MOESM12_ESM.zip › SD EV files/SD figure EV5/EV5A/EV_5_A_Roi /Mock/Gut close up/GFP ART C2 G Rab5GFP RAb7mCherrz control RNAi front_0010-1-1-1-1-1.tif]

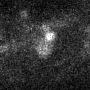

Supplement: Supplementary file 12 — Figure EV Source Data [file 44318_2025_367_MOESM12_ESM.zip › SD EV files/SD figure EV5/EV5A/EV_5_A_Roi /Mock/Gut close up/Merge ART C MGM Rab5GFP RAb7mCherrz control RNAi front_0010-1-1-1-1-1.tif]

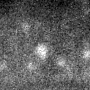

Supplement: Supplementary file 12 — Figure EV Source Data [file 44318_2025_367_MOESM12_ESM.zip › SD EV files/SD figure EV5/EV5A/EV_5_A_Roi /Mock/Gut close up/GFP ART C G Rab5GFP RAb7mCherrz control RNAi front_0010-1-1-1-1-1.tif]

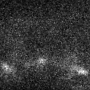

Supplement: Supplementary file 12 — Figure EV Source Data [file 44318_2025_367_MOESM12_ESM.zip › SD EV files/SD figure EV5/EV5A/EV_5_A_Roi /Mock/Gut close up/mCherry ART C2 MC Rab5GFP RAb7mCherrz control RNAi front_0010-1-1-1-1-1.tif]

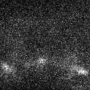

Supplement: Supplementary file 12 — Figure EV Source Data [file 44318_2025_367_MOESM12_ESM.zip › SD EV files/SD figure EV5/EV5A/EV_5_A_Roi /Mock/Gut close up/Merge ART C2 MGM Rab5GFP RAb7mCherrz control RNAi front_0010-1-1-1-1-1.tif]

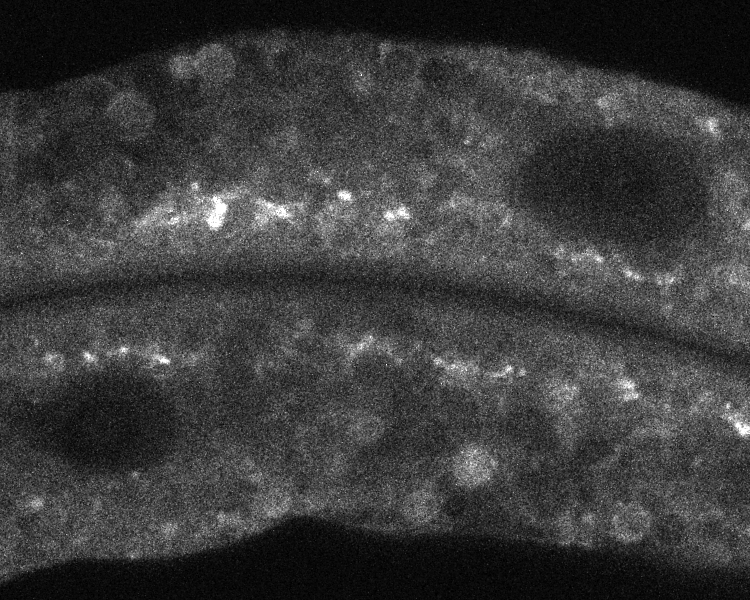

Supplement: Supplementary file 12 — Figure EV Source Data [file 44318_2025_367_MOESM12_ESM.zip › SD EV files/SD figure EV5/EV5A/EV_5_A_Roi /Mock/Gut/Merge ART MGM Rab5GFP RAb7mCherrz control RNAi front_0010-1-1-1-1.tif]

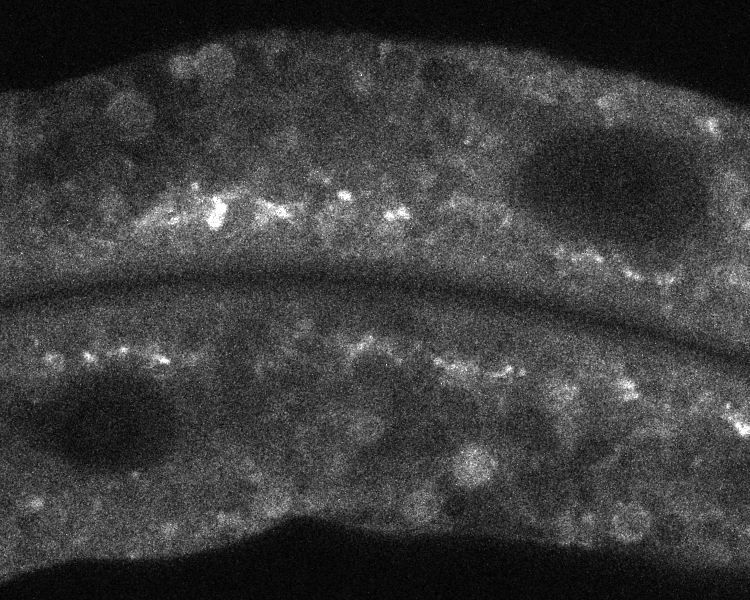

Supplement: Supplementary file 12 — Figure EV Source Data [file 44318_2025_367_MOESM12_ESM.zip › SD EV files/SD figure EV5/EV5A/EV_5_A_Roi /Mock/Gut/mCherry ART MC Rab5GFP RAb7mCherrz control RNAi front_0010-1-1-1-1.tif]

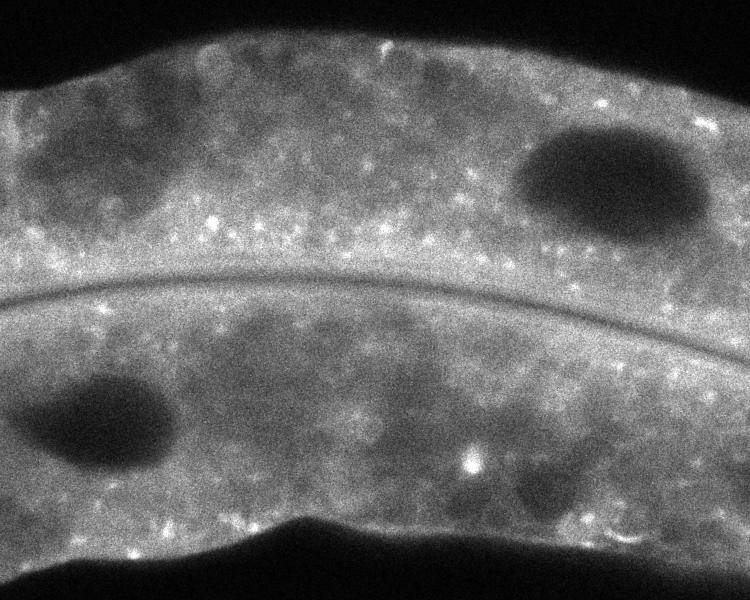

Supplement: Supplementary file 12 — Figure EV Source Data [file 44318_2025_367_MOESM12_ESM.zip › SD EV files/SD figure EV5/EV5A/EV_5_A_Roi /Mock/Gut/GFP ART G Rab5GFP RAb7mCherrz control RNAi front_0010-1-1-1-1.tif]

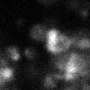

Supplement: Supplementary file 12 — Figure EV Source Data [file 44318_2025_367_MOESM12_ESM.zip › SD EV files/SD figure EV5/EV5A/EV_5_A_Roi /usp-50 (RNAi)/Gut close up/Merge ART C MGM Rab5GFP RAB7mCherrz usp50 RNAi front_0020-1-1-1-1-1.tif]

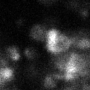

Supplement: Supplementary file 12 — Figure EV Source Data [file 44318_2025_367_MOESM12_ESM.zip › SD EV files/SD figure EV5/EV5A/EV_5_A_Roi /usp-50 (RNAi)/Gut close up/mCherry ART C MC Rab5GFP RAB7mCherrz usp50 RNAi front_0020-1-1-1-1-1.tif]

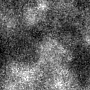

Supplement: Supplementary file 12 — Figure EV Source Data [file 44318_2025_367_MOESM12_ESM.zip › SD EV files/SD figure EV5/EV5A/EV_5_A_Roi /usp-50 (RNAi)/Gut close up/GFP ART C2 G Rab5GFP RAB7mCherrz usp50 RNAi front_0020-1-1-1-1-1.tif]

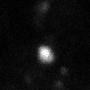

Supplement: Supplementary file 12 — Figure EV Source Data [file 44318_2025_367_MOESM12_ESM.zip › SD EV files/SD figure EV5/EV5A/EV_5_A_Roi /usp-50 (RNAi)/Gut close up/Merge ART C2 MGM Rab5GFP RAB7mCherrz usp50 RNAi front_0020-1-1-1-1-1.tif]

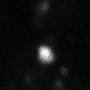

Supplement: Supplementary file 12 — Figure EV Source Data [file 44318_2025_367_MOESM12_ESM.zip › SD EV files/SD figure EV5/EV5A/EV_5_A_Roi /usp-50 (RNAi)/Gut close up/mCherry ART C2 MC Rab5GFP RAB7mCherrz usp50 RNAi front_0020-1-1-1-1-1.tif]

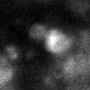

Supplement: Supplementary file 12 — Figure EV Source Data [file 44318_2025_367_MOESM12_ESM.zip › SD EV files/SD figure EV5/EV5A/EV_5_A_Roi /usp-50 (RNAi)/Gut close up/GFP ART C G Rab5GFP RAB7mCherrz usp50 RNAi front_0020-1-1-1-1-1.tif]

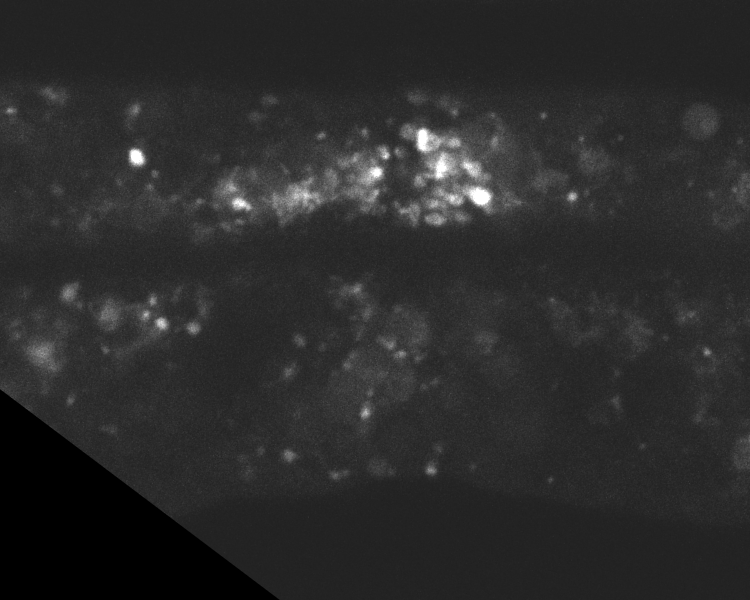

Supplement: Supplementary file 12 — Figure EV Source Data [file 44318_2025_367_MOESM12_ESM.zip › SD EV files/SD figure EV5/EV5A/EV_5_A_Roi /usp-50 (RNAi)/Gut/mCherry ART MC Rab5GFP RAB7mCherrz usp50 RNAi front_0020-1-1-1-1.tif]

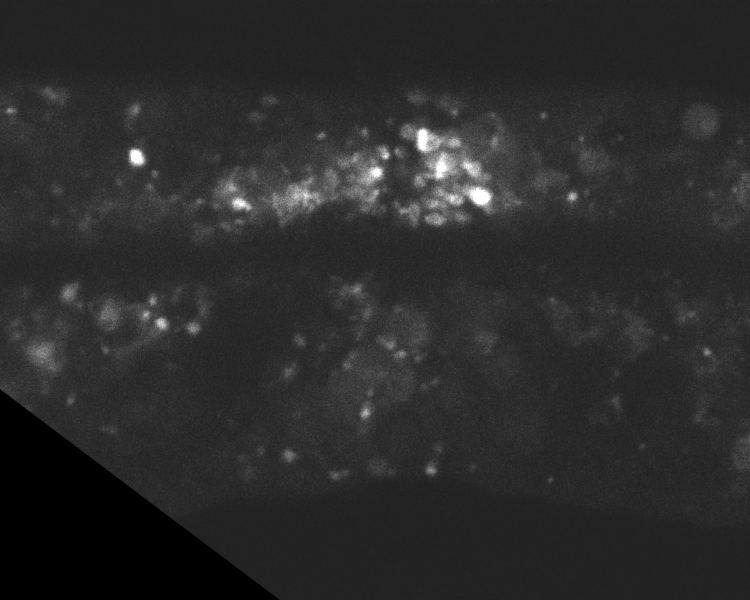

Supplement: Supplementary file 12 — Figure EV Source Data [file 44318_2025_367_MOESM12_ESM.zip › SD EV files/SD figure EV5/EV5A/EV_5_A_Roi /usp-50 (RNAi)/Gut/Merge ART MGM Rab5GFP RAB7mCherrz usp50 RNAi front_0020-1-1-1-1.tif]

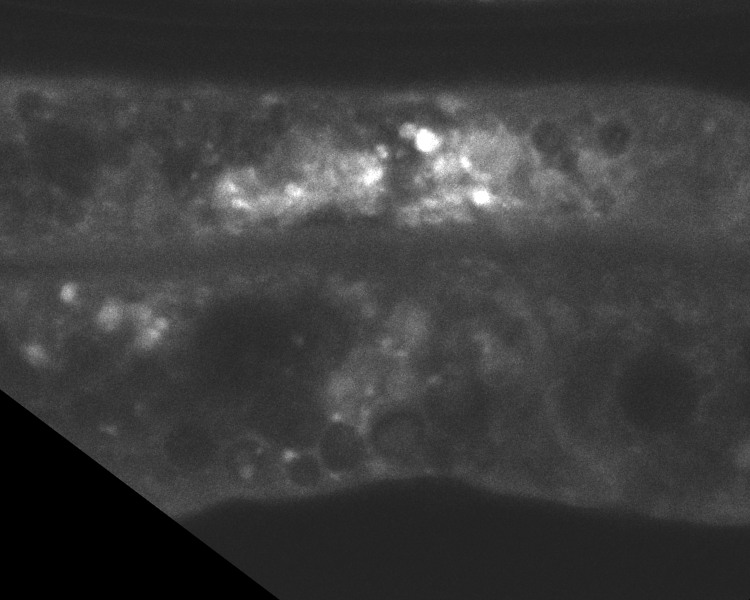

Supplement: Supplementary file 12 — Figure EV Source Data [file 44318_2025_367_MOESM12_ESM.zip › SD EV files/SD figure EV5/EV5A/EV_5_A_Roi /usp-50 (RNAi)/Gut/GFP ART G Rab5GFP RAB7mCherrz usp50 RNAi front_0020-1-1-1-1.tif]

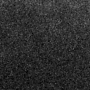

Supplement: Supplementary file 12 — Figure EV Source Data [file 44318_2025_367_MOESM12_ESM.zip › SD EV files/SD figure EV5/EV5A/EV_5_A_Roi /ubq-1 and control (RNAi)/Gut close up/GFP ART C2 G RAB5GFP RAB7mCherrz ubq1 and control RNAi 1 to 250 front_0004-1-1-1-1-1.tif]

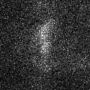

Supplement: Supplementary file 12 — Figure EV Source Data [file 44318_2025_367_MOESM12_ESM.zip › SD EV files/SD figure EV5/EV5A/EV_5_A_Roi /ubq-1 and control (RNAi)/Gut close up/mCherry ART C2 MC RAB5GFP RAB7mCherrz ubq1 and control RNAi 1 to 250 front_0004-1-1-1-1-1.tif]

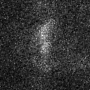

Supplement: Supplementary file 12 — Figure EV Source Data [file 44318_2025_367_MOESM12_ESM.zip › SD EV files/SD figure EV5/EV5A/EV_5_A_Roi /ubq-1 and control (RNAi)/Gut close up/Merge ART C2 MGM RAB5GFP RAB7mCherrz ubq1 and control RNAi 1 to 250 front_0004-1-1-1-1-1.tif]

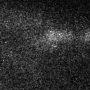

Supplement: Supplementary file 12 — Figure EV Source Data [file 44318_2025_367_MOESM12_ESM.zip › SD EV files/SD figure EV5/EV5A/EV_5_A_Roi /ubq-1 and control (RNAi)/Gut close up/mCherry ART C MC RAB5GFP RAB7mCherrz ubq1 and control RNAi 1 to 250 front_0004-1-1-1-1-1.tif]

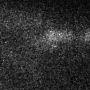

Supplement: Supplementary file 12 — Figure EV Source Data [file 44318_2025_367_MOESM12_ESM.zip › SD EV files/SD figure EV5/EV5A/EV_5_A_Roi /ubq-1 and control (RNAi)/Gut close up/Merge ART C MGM RAB5GFP RAB7mCherrz ubq1 and control RNAi 1 to 250 front_0004-1-1-1-1-1.tif]

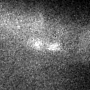

Supplement: Supplementary file 12 — Figure EV Source Data [file 44318_2025_367_MOESM12_ESM.zip › SD EV files/SD figure EV5/EV5A/EV_5_A_Roi /ubq-1 and control (RNAi)/Gut close up/GFP ART C G RAB5GFP RAB7mCherrz ubq1 and control RNAi 1 to 250 front_0004-1-1-1-1-1.tif]

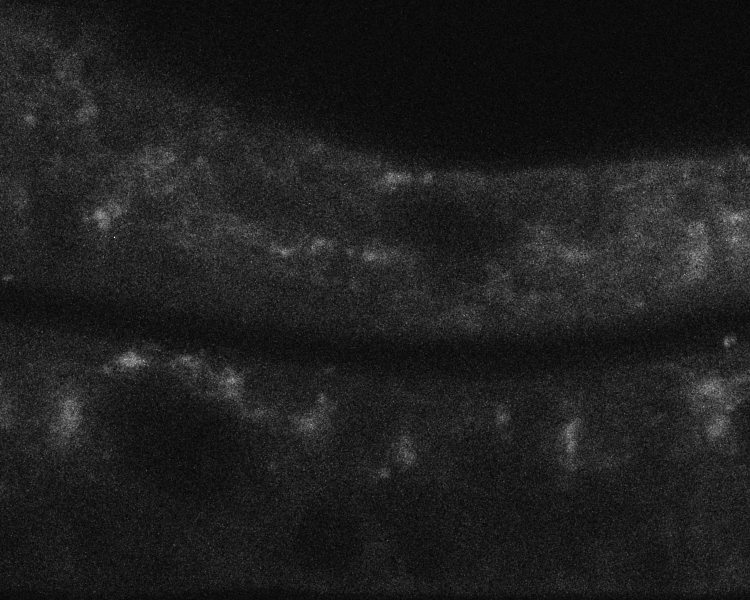

Supplement: Supplementary file 12 — Figure EV Source Data [file 44318_2025_367_MOESM12_ESM.zip › SD EV files/SD figure EV5/EV5A/EV_5_A_Roi /ubq-1 and control (RNAi)/Gut/mCherry ART MC RAB5GFP RAB7mCherrz ubq1 and control RNAi 1 to 250 front_0004-1-1-1-1.tif]

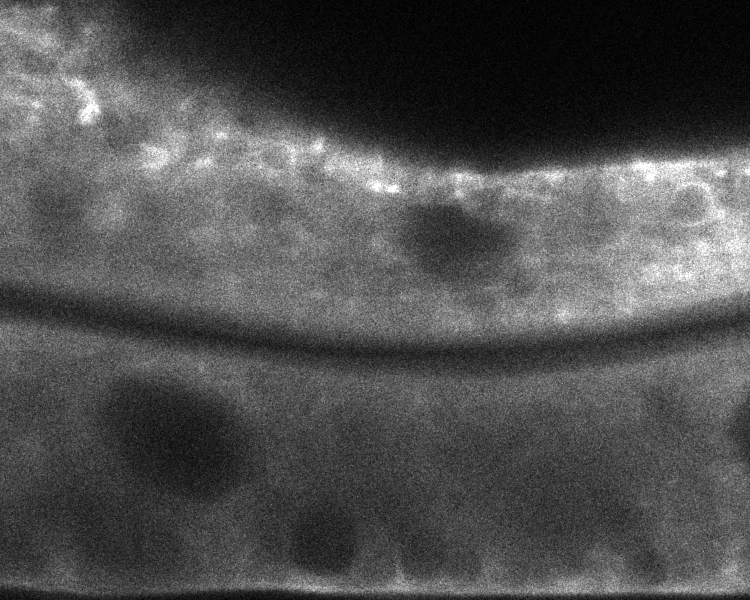

Supplement: Supplementary file 12 — Figure EV Source Data [file 44318_2025_367_MOESM12_ESM.zip › SD EV files/SD figure EV5/EV5A/EV_5_A_Roi /ubq-1 and control (RNAi)/Gut/GFP ART G RAB5GFP RAB7mCherrz ubq1 and control RNAi 1 to 250 front_0004-1-1-1-1.tif]

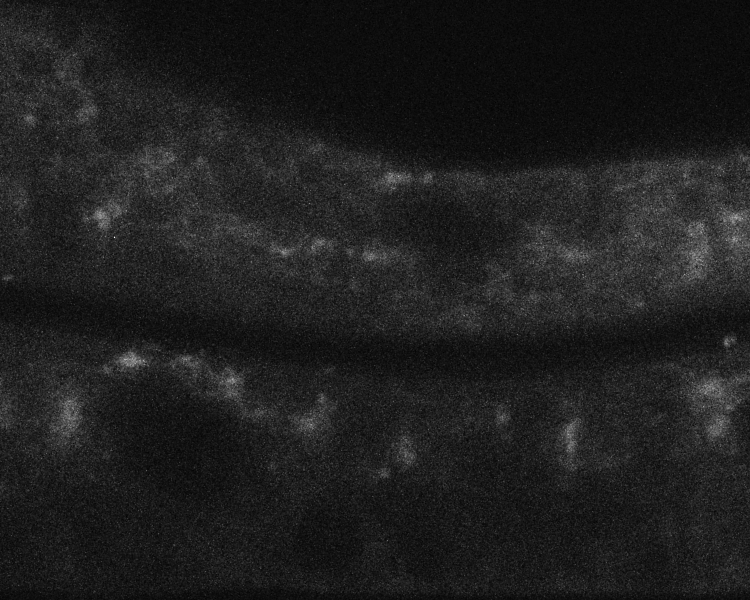

Supplement: Supplementary file 12 — Figure EV Source Data [file 44318_2025_367_MOESM12_ESM.zip › SD EV files/SD figure EV5/EV5A/EV_5_A_Roi /ubq-1 and control (RNAi)/Gut/Merge ART MGM RAB5GFP RAB7mCherrz ubq1 and control RNAi 1 to 250 front_0004-1-1-1-1.tif]

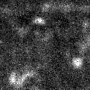

Supplement: Supplementary file 12 — Figure EV Source Data [file 44318_2025_367_MOESM12_ESM.zip › SD EV files/SD figure EV5/EV5C/EV_5_C_Roi/Mock/Gut close up/Merge ART C MGR RFPrab5 GFPubq control RNAi front_0013-1-1-1-1-1.tif]

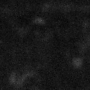

Supplement: Supplementary file 12 — Figure EV Source Data [file 44318_2025_367_MOESM12_ESM.zip › SD EV files/SD figure EV5/EV5C/EV_5_C_Roi/Mock/Gut close up/RFP ART C RF RFPrab5 GFPubq control RNAi front_0013-1-1-1-1-1.tif]

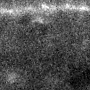

Supplement: Supplementary file 12 — Figure EV Source Data [file 44318_2025_367_MOESM12_ESM.zip › SD EV files/SD figure EV5/EV5C/EV_5_C_Roi/Mock/Gut close up/GFP ART C G RFPrab5 GFPubq control RNAi front_0013-1-1-1-1-1.tif]

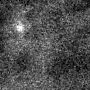

Supplement: Supplementary file 12 — Figure EV Source Data [file 44318_2025_367_MOESM12_ESM.zip › SD EV files/SD figure EV5/EV5C/EV_5_C_Roi/Mock/Gut close up/GFP ART C2 G RFPrab5 GFPubq control RNAi front_0013-1-1-1-2-1.tif]

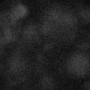

Supplement: Supplementary file 12 — Figure EV Source Data [file 44318_2025_367_MOESM12_ESM.zip › SD EV files/SD figure EV5/EV5C/EV_5_C_Roi/Mock/Gut close up/RFP ART C2 RF RFPrab5 GFPubq control RNAi front_0013-1-1-1-2-1.tif]

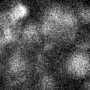

Supplement: Supplementary file 12 — Figure EV Source Data [file 44318_2025_367_MOESM12_ESM.zip › SD EV files/SD figure EV5/EV5C/EV_5_C_Roi/Mock/Gut close up/Merge ART C2 MGR RFPrab5 GFPubq control RNAi front_0013-1-1-1-2-1.tif]

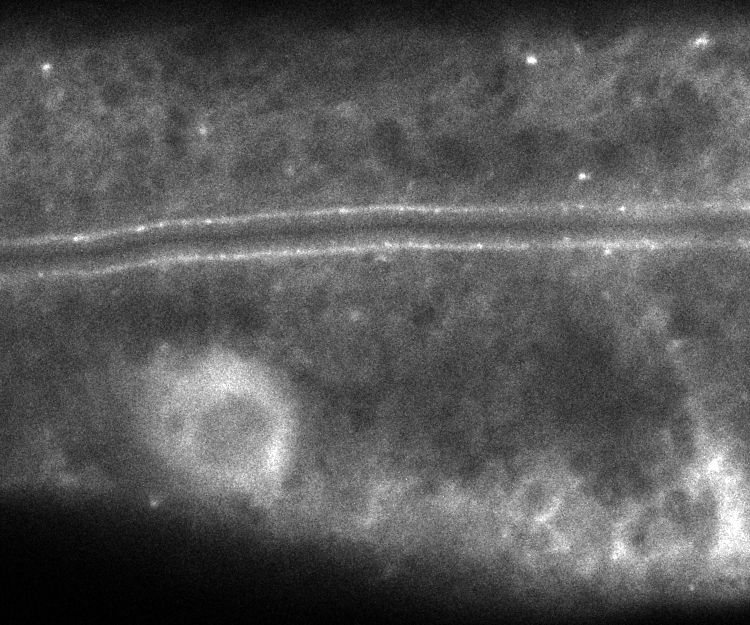

Supplement: Supplementary file 12 — Figure EV Source Data [file 44318_2025_367_MOESM12_ESM.zip › SD EV files/SD figure EV5/EV5C/EV_5_C_Roi/Mock/Gut/GFP ART G RFPrab5 GFPubq control RNAi front_0013-1-1-1-1.tif]

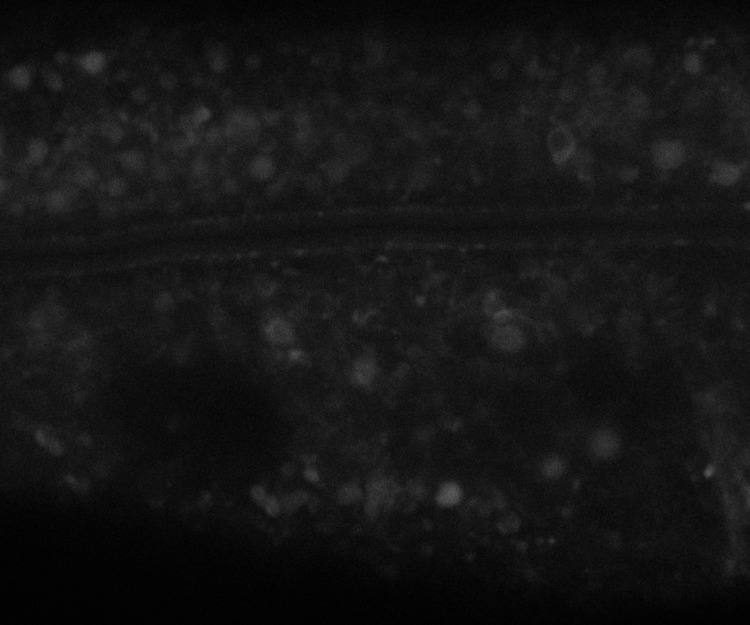

Supplement: Supplementary file 12 — Figure EV Source Data [file 44318_2025_367_MOESM12_ESM.zip › SD EV files/SD figure EV5/EV5C/EV_5_C_Roi/Mock/Gut/RFP ART RF RFPrab5 GFPubq control RNAi front_0013-1-1-1-1.tif]

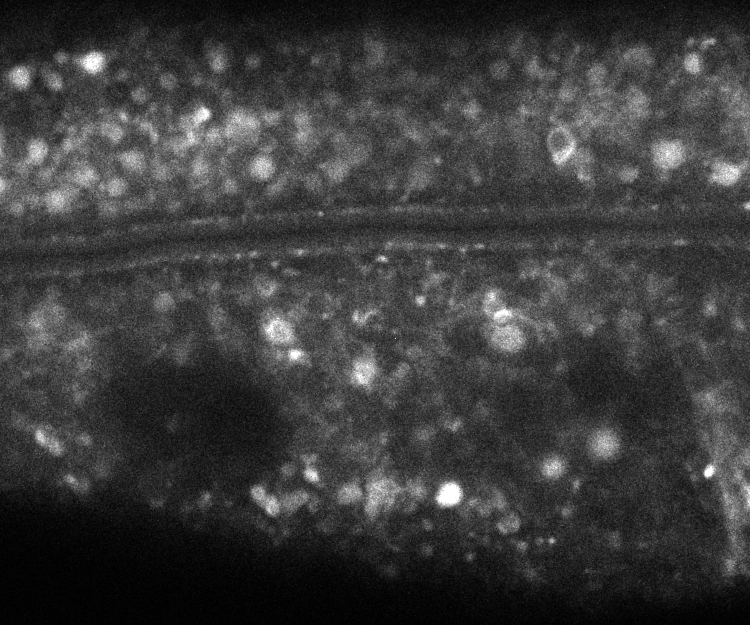

Supplement: Supplementary file 12 — Figure EV Source Data [file 44318_2025_367_MOESM12_ESM.zip › SD EV files/SD figure EV5/EV5C/EV_5_C_Roi/Mock/Gut/Merge ART MGR RFPrab5 GFPubq control RNAi front_0013-1-1-1-1.tif]

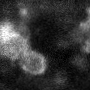

Supplement: Supplementary file 12 — Figure EV Source Data [file 44318_2025_367_MOESM12_ESM.zip › SD EV files/SD figure EV5/EV5C/EV_5_C_Roi/usp-50 (RNAi)/Gut close up/GFP ART C G RFPrab5 GFPubq usp50 RNAi front_0007-1-1-1-1-1.tif]

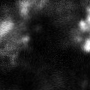

Supplement: Supplementary file 12 — Figure EV Source Data [file 44318_2025_367_MOESM12_ESM.zip › SD EV files/SD figure EV5/EV5C/EV_5_C_Roi/usp-50 (RNAi)/Gut close up/Merge ART C MGR RFPrab5 GFPubq usp50 RNAi front_0007-1-1-1-1-1.tif]

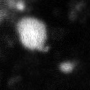

Supplement: Supplementary file 12 — Figure EV Source Data [file 44318_2025_367_MOESM12_ESM.zip › SD EV files/SD figure EV5/EV5C/EV_5_C_Roi/usp-50 (RNAi)/Gut close up/Merge ART C2 MGR RFPrab5 GFPubq usp50 RNAi front_0007-1-1-1-1-1.tif]

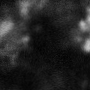

Supplement: Supplementary file 12 — Figure EV Source Data [file 44318_2025_367_MOESM12_ESM.zip › SD EV files/SD figure EV5/EV5C/EV_5_C_Roi/usp-50 (RNAi)/Gut close up/RFP ART C RF RFPrab5 GFPubq usp50 RNAi front_0007-1-1-1-1-1.tif]

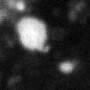

Supplement: Supplementary file 12 — Figure EV Source Data [file 44318_2025_367_MOESM12_ESM.zip › SD EV files/SD figure EV5/EV5C/EV_5_C_Roi/usp-50 (RNAi)/Gut close up/RFP ART C2 RF RFPrab5 GFPubq usp50 RNAi front_0007-1-1-1-1-1.tif]

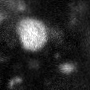

Supplement: Supplementary file 12 — Figure EV Source Data [file 44318_2025_367_MOESM12_ESM.zip › SD EV files/SD figure EV5/EV5C/EV_5_C_Roi/usp-50 (RNAi)/Gut close up/GFP ART C2 G RFPrab5 GFPubq usp50 RNAi front_0007-1-1-1-1-1.tif]

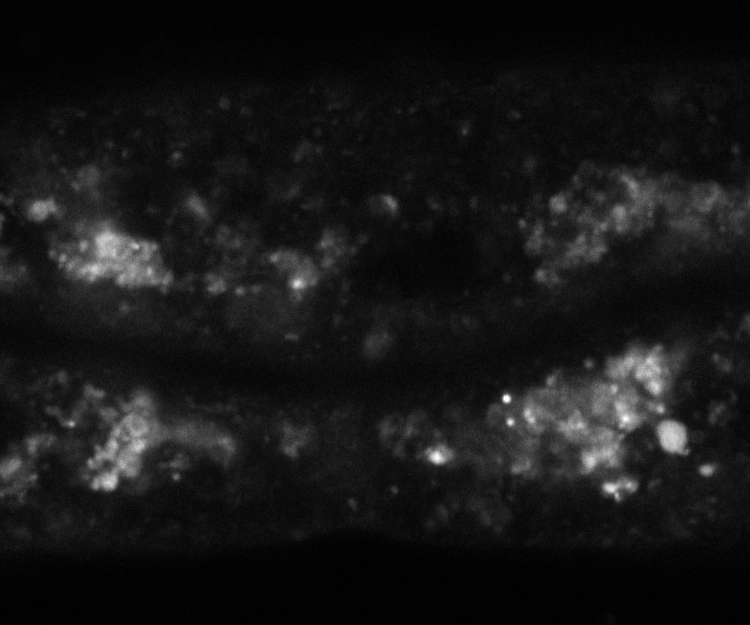

Supplement: Supplementary file 12 — Figure EV Source Data [file 44318_2025_367_MOESM12_ESM.zip › SD EV files/SD figure EV5/EV5C/EV_5_C_Roi/usp-50 (RNAi)/Gut/RFP ART RF RFPrab5 GFPubq usp50 RNAi front_0007-1-1-1-1.tif]

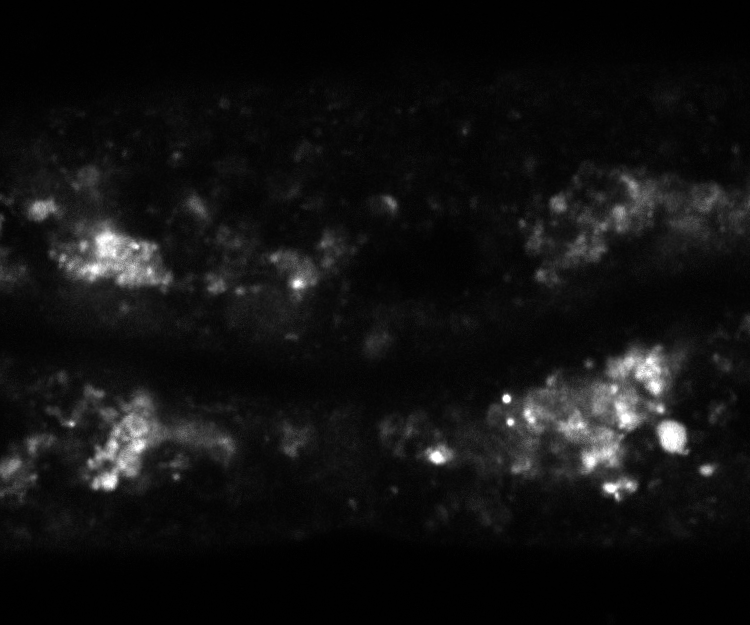

Supplement: Supplementary file 12 — Figure EV Source Data [file 44318_2025_367_MOESM12_ESM.zip › SD EV files/SD figure EV5/EV5C/EV_5_C_Roi/usp-50 (RNAi)/Gut/Merge ART MGR RFPrab5 GFPubq usp50 RNAi front_0007-1-1-1-1.tif]

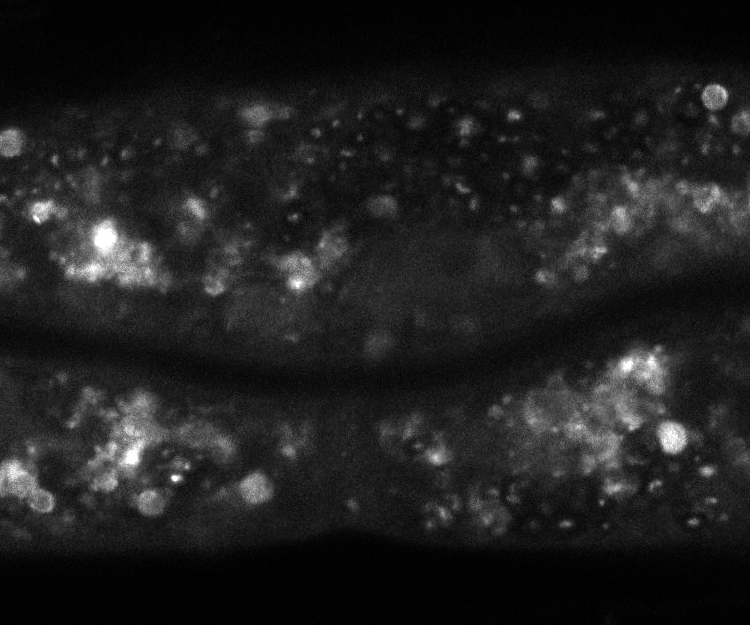

Supplement: Supplementary file 12 — Figure EV Source Data [file 44318_2025_367_MOESM12_ESM.zip › SD EV files/SD figure EV5/EV5C/EV_5_C_Roi/usp-50 (RNAi)/Gut/GFP ART G RFPrab5 GFPubq usp50 RNAi front_0007-1-1-1-1.tif]

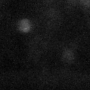

Supplement: Supplementary file 12 — Figure EV Source Data [file 44318_2025_367_MOESM12_ESM.zip › SD EV files/SD figure EV5/EV5C/EV_5_C_Roi/ubq-1 and control (RNAi)/Gut close up/RFP ART C2 RF RFPrab5 GFPubq ubq1 and control RNAi 1 to 250 front_0010-1-1-1-1-1.tif]

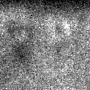

Supplement: Supplementary file 12 — Figure EV Source Data [file 44318_2025_367_MOESM12_ESM.zip › SD EV files/SD figure EV5/EV5C/EV_5_C_Roi/ubq-1 and control (RNAi)/Gut close up/GFP ART C G RFPrab5 GFPubq ubq1 and control RNAi 1 to 250 front_0010-1-1-1-1-1.tif]

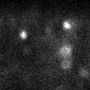

Supplement: Supplementary file 12 — Figure EV Source Data [file 44318_2025_367_MOESM12_ESM.zip › SD EV files/SD figure EV5/EV5C/EV_5_C_Roi/ubq-1 and control (RNAi)/Gut close up/Merge ART C MGR RFPrab5 GFPubq ubq1 and control RNAi 1 to 250 front_0010-1-1-1-1-1.tif]

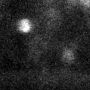

Supplement: Supplementary file 12 — Figure EV Source Data [file 44318_2025_367_MOESM12_ESM.zip › SD EV files/SD figure EV5/EV5C/EV_5_C_Roi/ubq-1 and control (RNAi)/Gut close up/Merge ART C2 MGR RFPrab5 GFPubq ubq1 and control RNAi 1 to 250 front_0010-1-1-1-1-1.tif]

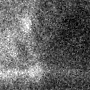

Supplement: Supplementary file 12 — Figure EV Source Data [file 44318_2025_367_MOESM12_ESM.zip › SD EV files/SD figure EV5/EV5C/EV_5_C_Roi/ubq-1 and control (RNAi)/Gut close up/GFP ART C2 G RFPrab5 GFPubq ubq1 and control RNAi 1 to 250 front_0010-1-1-1-1-1.tif]

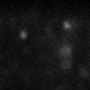

Supplement: Supplementary file 12 — Figure EV Source Data [file 44318_2025_367_MOESM12_ESM.zip › SD EV files/SD figure EV5/EV5C/EV_5_C_Roi/ubq-1 and control (RNAi)/Gut close up/RFP ART C RF RFPrab5 GFPubq ubq1 and control RNAi 1 to 250 front_0010-1-1-1-1-1.tif]

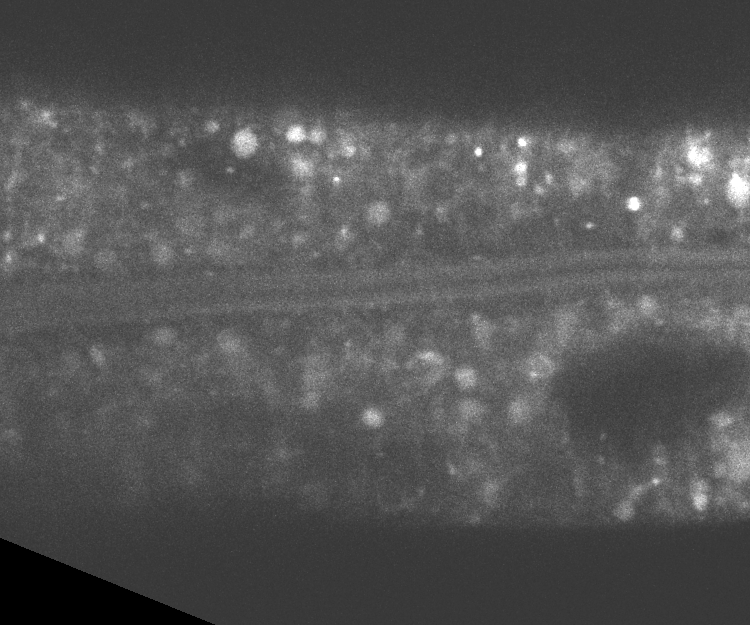

Supplement: Supplementary file 12 — Figure EV Source Data [file 44318_2025_367_MOESM12_ESM.zip › SD EV files/SD figure EV5/EV5C/EV_5_C_Roi/ubq-1 and control (RNAi)/Gut/RFP ART2 RF RFPrab5 GFPubq ubq1 and control RNAi 1 to 250 front_0010-1-1-1-1.tif]

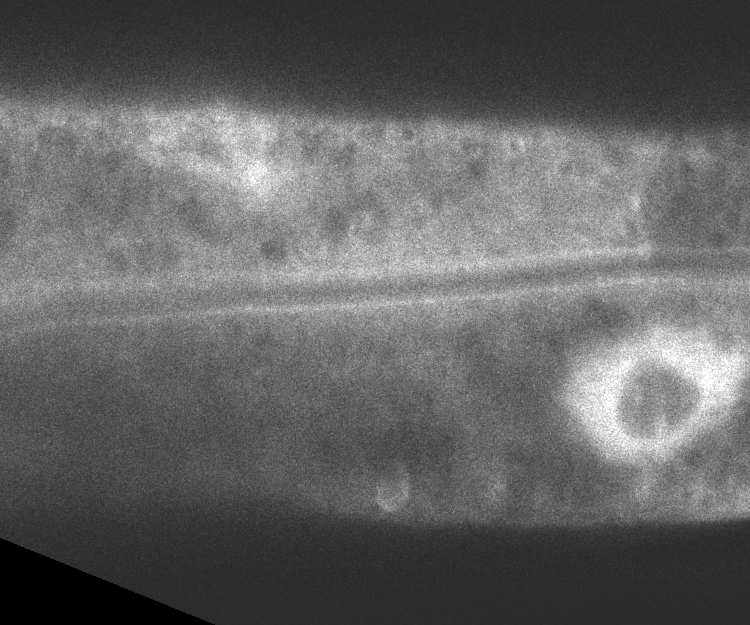

Supplement: Supplementary file 12 — Figure EV Source Data [file 44318_2025_367_MOESM12_ESM.zip › SD EV files/SD figure EV5/EV5C/EV_5_C_Roi/ubq-1 and control (RNAi)/Gut/GFP ART2 G RFPrab5 GFPubq ubq1 and control RNAi 1 to 250 front_0010-1-1-1-1.tif]

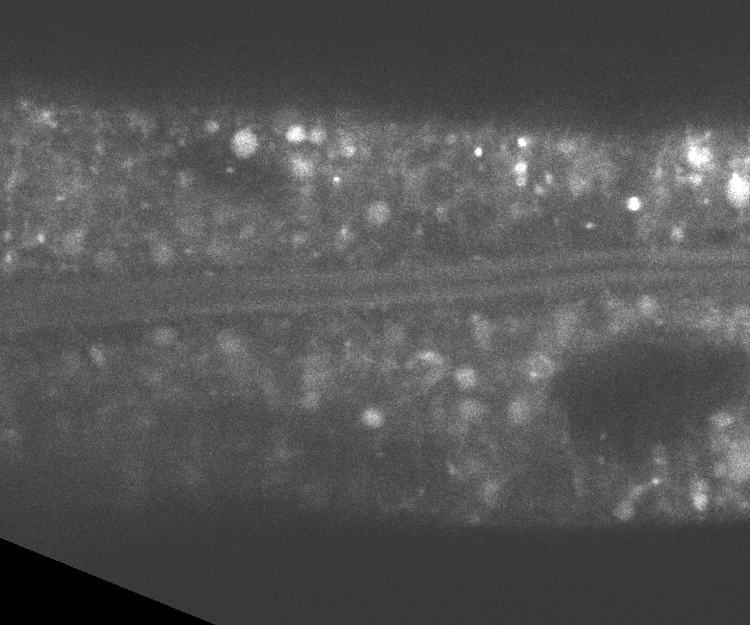

Supplement: Supplementary file 12 — Figure EV Source Data [file 44318_2025_367_MOESM12_ESM.zip › SD EV files/SD figure EV5/EV5C/EV_5_C_Roi/ubq-1 and control (RNAi)/Gut/Merge ART2 MGR RFPrab5 GFPubq ubq1 and control RNAi 1 to 250 front_0010-1-1-1-1.tif]

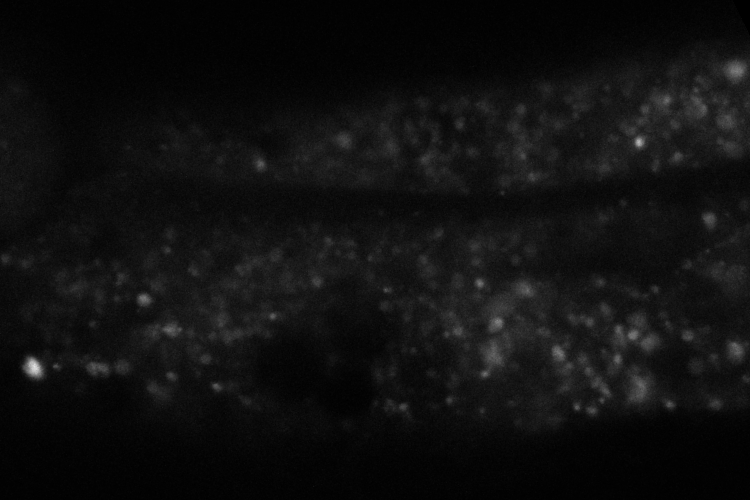

Supplement: Supplementary file 12 — Figure EV Source Data [file 44318_2025_367_MOESM12_ESM.zip › SD EV files/SD figure EV1/EV1A/EV_1_A_Roi/vps-32.1 (RNAi)/Gut /mCherry ART MC rab5 rab7 vps 32.1 rnai front_0001-1-1-1-1.tif]

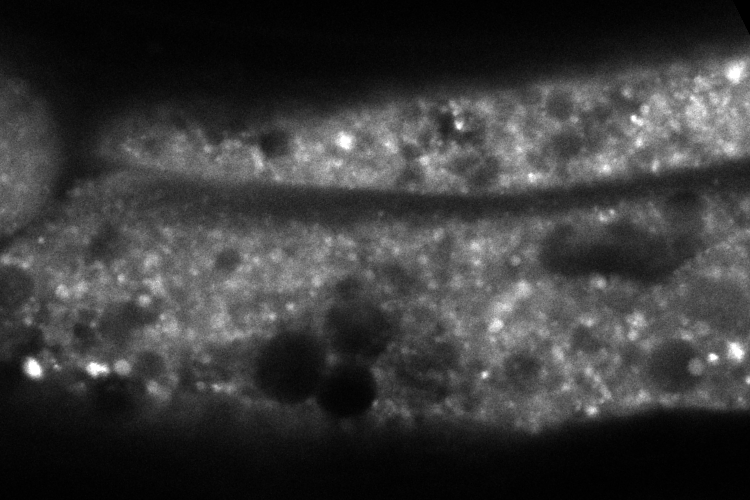

Supplement: Supplementary file 12 — Figure EV Source Data [file 44318_2025_367_MOESM12_ESM.zip › SD EV files/SD figure EV1/EV1A/EV_1_A_Roi/vps-32.1 (RNAi)/Gut /Merge ART MGM rab5 rab7 vps 32.1 rnai front_0001-1-1-1.tif]

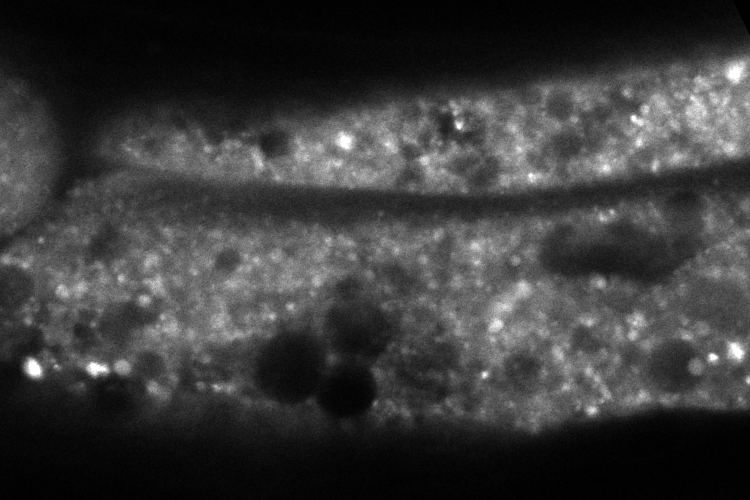

Supplement: Supplementary file 12 — Figure EV Source Data [file 44318_2025_367_MOESM12_ESM.zip › SD EV files/SD figure EV1/EV1A/EV_1_A_Roi/vps-32.1 (RNAi)/Gut /GFP ART G rab5 rab7 vps 32.1 rnai front_0001-1-1-1-1.tif]

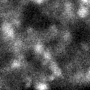

Supplement: Supplementary file 12 — Figure EV Source Data [file 44318_2025_367_MOESM12_ESM.zip › SD EV files/SD figure EV1/EV1A/EV_1_A_Roi/vps-32.1 (RNAi)/Gut close up/GFP ART C G rab5 rab7 vps 32.1 rnai front_0001-1-1-1-1-1.tif]

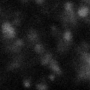

Supplement: Supplementary file 12 — Figure EV Source Data [file 44318_2025_367_MOESM12_ESM.zip › SD EV files/SD figure EV1/EV1A/EV_1_A_Roi/vps-32.1 (RNAi)/Gut close up/mCherry ART C MC rab5 rab7 vps 32.1 rnai front_0001-1-1-1-1-1.tif]

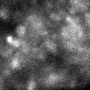

Supplement: Supplementary file 12 — Figure EV Source Data [file 44318_2025_367_MOESM12_ESM.zip › SD EV files/SD figure EV1/EV1A/EV_1_A_Roi/vps-32.1 (RNAi)/Gut close up/Merge ART C2 MGM rab5 rab7 vps 32.1 rnai front_0001-1-1-1-1.tif]

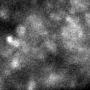

Supplement: Supplementary file 12 — Figure EV Source Data [file 44318_2025_367_MOESM12_ESM.zip › SD EV files/SD figure EV1/EV1A/EV_1_A_Roi/vps-32.1 (RNAi)/Gut close up/GFP ART C2 G rab5 rab7 vps 32.1 rnai front_0001-1-1-1-1-1.tif]

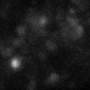

Supplement: Supplementary file 12 — Figure EV Source Data [file 44318_2025_367_MOESM12_ESM.zip › SD EV files/SD figure EV1/EV1A/EV_1_A_Roi/vps-32.1 (RNAi)/Gut close up/mCherry ART C2 MC rab5 rab7 vps 32.1 rnai front_0001-1-1-1-1-1.tif]

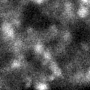

Supplement: Supplementary file 12 — Figure EV Source Data [file 44318_2025_367_MOESM12_ESM.zip › SD EV files/SD figure EV1/EV1A/EV_1_A_Roi/vps-32.1 (RNAi)/Gut close up/Merge ART C MGM rab5 rab7 vps 32.1 rnai front_0001-1-1-1-1.tif]

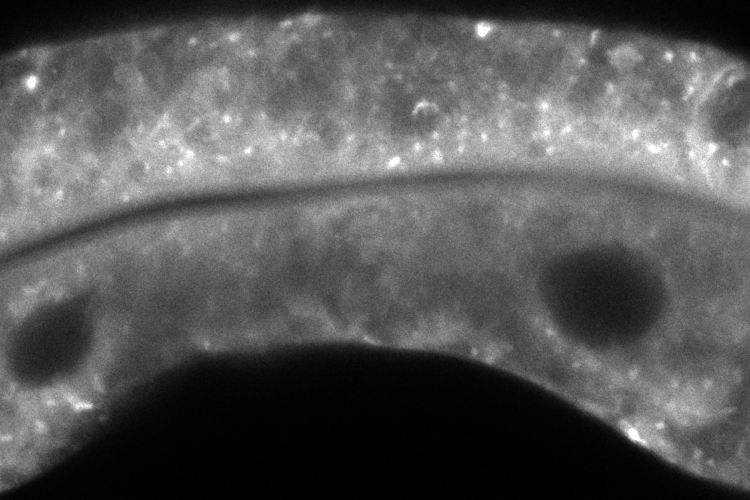

Supplement: Supplementary file 12 — Figure EV Source Data [file 44318_2025_367_MOESM12_ESM.zip › SD EV files/SD figure EV1/EV1A/EV_1_A_Roi/Mock/Gut /GFP ART G rab5&rab7 control rnai front_0009-1-1-1-1.tif]

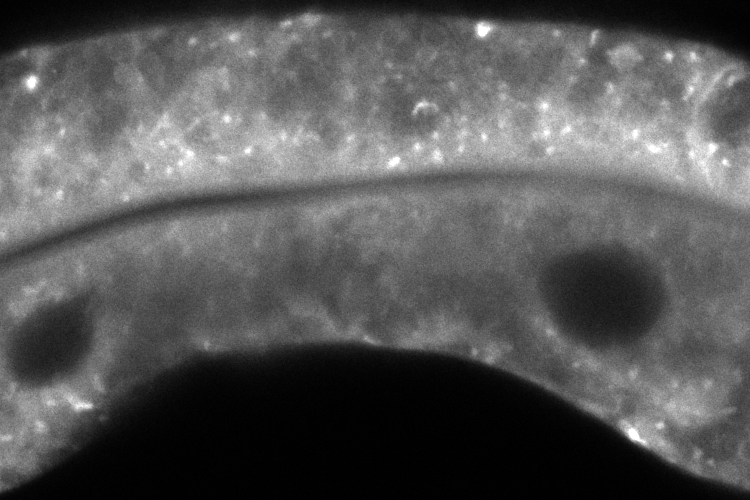

Supplement: Supplementary file 12 — Figure EV Source Data [file 44318_2025_367_MOESM12_ESM.zip › SD EV files/SD figure EV1/EV1A/EV_1_A_Roi/Mock/Gut /Merge ART MGM rab5&rab7 control rnai front_0009-1-1-1.tif]

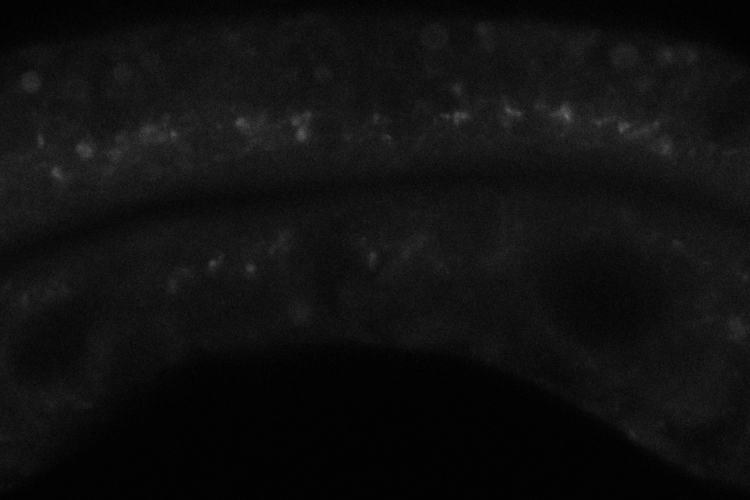

Supplement: Supplementary file 12 — Figure EV Source Data [file 44318_2025_367_MOESM12_ESM.zip › SD EV files/SD figure EV1/EV1A/EV_1_A_Roi/Mock/Gut /mCherry ART MC rab5&rab7 control rnai front_0009-1-1-1-1.tif]

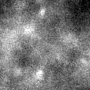

Supplement: Supplementary file 12 — Figure EV Source Data [file 44318_2025_367_MOESM12_ESM.zip › SD EV files/SD figure EV1/EV1A/EV_1_A_Roi/Mock/Gut close up/GFP ART C2 G rab5&rab7 control rnai front_0009-1-1-1-1-1.tif]

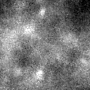

Supplement: Supplementary file 12 — Figure EV Source Data [file 44318_2025_367_MOESM12_ESM.zip › SD EV files/SD figure EV1/EV1A/EV_1_A_Roi/Mock/Gut close up/Merge ART C2 MGM rab5&rab7 control rnai front_0009-1-1-1-1.tif]

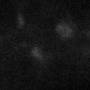

Supplement: Supplementary file 12 — Figure EV Source Data [file 44318_2025_367_MOESM12_ESM.zip › SD EV files/SD figure EV1/EV1A/EV_1_A_Roi/Mock/Gut close up/mCherry ART C2 MC rab5&rab7 control rnai front_0009-1-1-1-1-1.tif]

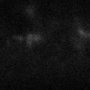

Supplement: Supplementary file 12 — Figure EV Source Data [file 44318_2025_367_MOESM12_ESM.zip › SD EV files/SD figure EV1/EV1A/EV_1_A_Roi/Mock/Gut close up/mCherry ART C MC rab5&rab7 control rnai front_0009-1-1-1-1-1.tif]
