# Supplementary figures and images for: Coordination between ESCRT function and Rab conversion during endosome maturation (part 8 of 9)
Source: EMBO J. 2025 Feb 5;44(6):1574–607. doi: 10.1038/s44318-025-00367-7 (PMC11914609; doi:10.1038/s44318-025-00367-7)

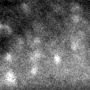

Supplement: Supplementary file 12 — Figure EV Source Data [file 44318_2025_367_MOESM12_ESM.zip › SD EV files/SD figure EV1/EV1A/EV_1_A_Roi/Mock/Gut close up/GFP ART C G rab5&rab7 control rnai front_0009-1-1-1-1-1.tif]

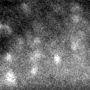

Supplement: Supplementary file 12 — Figure EV Source Data [file 44318_2025_367_MOESM12_ESM.zip › SD EV files/SD figure EV1/EV1A/EV_1_A_Roi/Mock/Gut close up/Merge ART C MGM rab5&rab7 control rnai front_0009-1-1-1-1.tif]

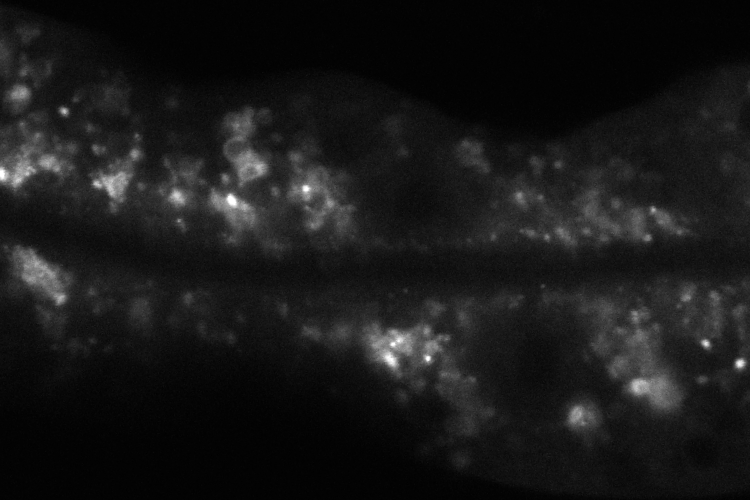

Supplement: Supplementary file 12 — Figure EV Source Data [file 44318_2025_367_MOESM12_ESM.zip › SD EV files/SD figure EV1/EV1A/EV_1_A_Roi/vps-28 (RNAi)/Gut /mCherry ART MC rab5&rab7 vps28 rnai front_0005-1-1-1-1.tif]

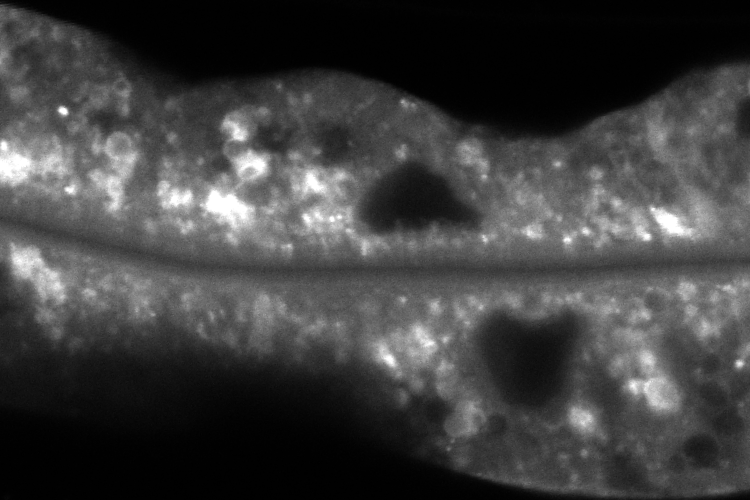

Supplement: Supplementary file 12 — Figure EV Source Data [file 44318_2025_367_MOESM12_ESM.zip › SD EV files/SD figure EV1/EV1A/EV_1_A_Roi/vps-28 (RNAi)/Gut /Merge ART MGM rab5&rab7 vps28 rnai front_0005-1-1-1.tif]

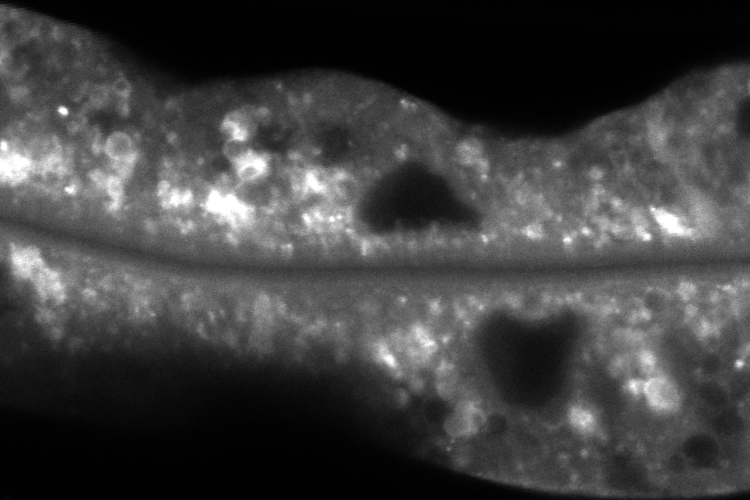

Supplement: Supplementary file 12 — Figure EV Source Data [file 44318_2025_367_MOESM12_ESM.zip › SD EV files/SD figure EV1/EV1A/EV_1_A_Roi/vps-28 (RNAi)/Gut /GFP ART G rab5&rab7 vps28 rnai front_0005-1-1-1-1.tif]

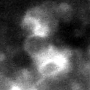

Supplement: Supplementary file 12 — Figure EV Source Data [file 44318_2025_367_MOESM12_ESM.zip › SD EV files/SD figure EV1/EV1A/EV_1_A_Roi/vps-28 (RNAi)/Gut close up/Merge ART C2 MGM rab5&rab7 vps28 rnai front_0005-1-1-1-1.tif]

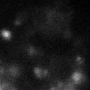

Supplement: Supplementary file 12 — Figure EV Source Data [file 44318_2025_367_MOESM12_ESM.zip › SD EV files/SD figure EV1/EV1A/EV_1_A_Roi/vps-28 (RNAi)/Gut close up/mCherry ART C MC rab5&rab7 vps28 rnai front_0005-1-1-1-1-1.tif]

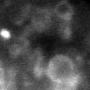

Supplement: Supplementary file 12 — Figure EV Source Data [file 44318_2025_367_MOESM12_ESM.zip › SD EV files/SD figure EV1/EV1A/EV_1_A_Roi/vps-28 (RNAi)/Gut close up/Merge ART C MGM rab5&rab7 vps28 rnai front_0005-1-1-1-1.tif]

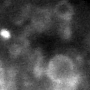

Supplement: Supplementary file 12 — Figure EV Source Data [file 44318_2025_367_MOESM12_ESM.zip › SD EV files/SD figure EV1/EV1A/EV_1_A_Roi/vps-28 (RNAi)/Gut close up/GFP ART C G rab5&rab7 vps28 rnai front_0005-1-1-1-1-1.tif]

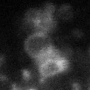

Supplement: Supplementary file 12 — Figure EV Source Data [file 44318_2025_367_MOESM12_ESM.zip › SD EV files/SD figure EV1/EV1A/EV_1_A_Roi/vps-28 (RNAi)/Gut close up/mCherry ART C2 MC rab5&rab7 vps28 rnai front_0005-1-1-1-1-1.tif]

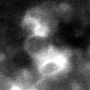

Supplement: Supplementary file 12 — Figure EV Source Data [file 44318_2025_367_MOESM12_ESM.zip › SD EV files/SD figure EV1/EV1A/EV_1_A_Roi/vps-28 (RNAi)/Gut close up/GFP ART C2 G rab5&rab7 vps28 rnai front_0005-1-1-1-1-1.tif]

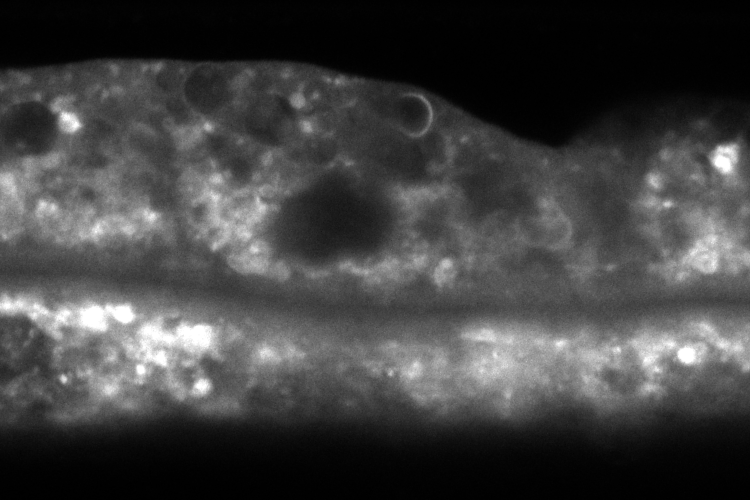

Supplement: Supplementary file 12 — Figure EV Source Data [file 44318_2025_367_MOESM12_ESM.zip › SD EV files/SD figure EV1/EV1A/EV_1_A_Roi/vps-24 (RNAi)/Gut /Merge ART MGM rab 5 rab7 vps24 rnai front_0005-1-1-1.tif]

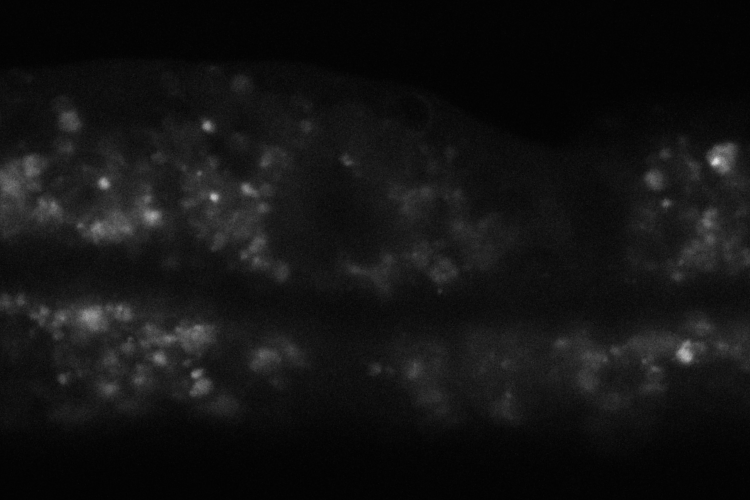

Supplement: Supplementary file 12 — Figure EV Source Data [file 44318_2025_367_MOESM12_ESM.zip › SD EV files/SD figure EV1/EV1A/EV_1_A_Roi/vps-24 (RNAi)/Gut /mCherry ART MC rab 5 rab7 vps24 rnai front_0005-1-1-1-1.tif]

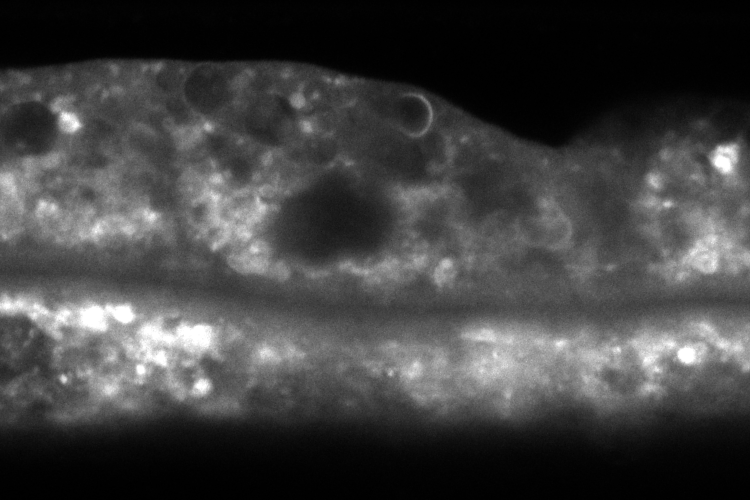

Supplement: Supplementary file 12 — Figure EV Source Data [file 44318_2025_367_MOESM12_ESM.zip › SD EV files/SD figure EV1/EV1A/EV_1_A_Roi/vps-24 (RNAi)/Gut /GFP ART G rab 5 rab7 vps24 rnai front_0005-1-1-1-1.tif]

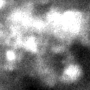

Supplement: Supplementary file 12 — Figure EV Source Data [file 44318_2025_367_MOESM12_ESM.zip › SD EV files/SD figure EV1/EV1A/EV_1_A_Roi/vps-24 (RNAi)/Gut close up/GFP ART C2 G rab 5 rab7 vps24 rnai front_0005-1-1-1-1-1.tif]

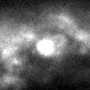

Supplement: Supplementary file 12 — Figure EV Source Data [file 44318_2025_367_MOESM12_ESM.zip › SD EV files/SD figure EV1/EV1A/EV_1_A_Roi/vps-24 (RNAi)/Gut close up/Merge ART C MGM rab 5 rab7 vps24 rnai front_0005-1-1-1-1.tif]

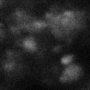

Supplement: Supplementary file 12 — Figure EV Source Data [file 44318_2025_367_MOESM12_ESM.zip › SD EV files/SD figure EV1/EV1A/EV_1_A_Roi/vps-24 (RNAi)/Gut close up/mCherry ART C2 MC rab 5 rab7 vps24 rnai front_0005-1-1-1-1-1.tif]

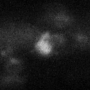

Supplement: Supplementary file 12 — Figure EV Source Data [file 44318_2025_367_MOESM12_ESM.zip › SD EV files/SD figure EV1/EV1A/EV_1_A_Roi/vps-24 (RNAi)/Gut close up/mCherry ART C MC rab 5 rab7 vps24 rnai front_0005-1-1-1-1-1.tif]

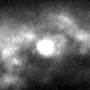

Supplement: Supplementary file 12 — Figure EV Source Data [file 44318_2025_367_MOESM12_ESM.zip › SD EV files/SD figure EV1/EV1A/EV_1_A_Roi/vps-24 (RNAi)/Gut close up/GFP ART C G rab 5 rab7 vps24 rnai front_0005-1-1-1-1-1.tif]

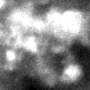

Supplement: Supplementary file 12 — Figure EV Source Data [file 44318_2025_367_MOESM12_ESM.zip › SD EV files/SD figure EV1/EV1A/EV_1_A_Roi/vps-24 (RNAi)/Gut close up/Merge ART C2 MGM rab 5 rab7 vps24 rnai front_0005-1-1-1-1.tif]

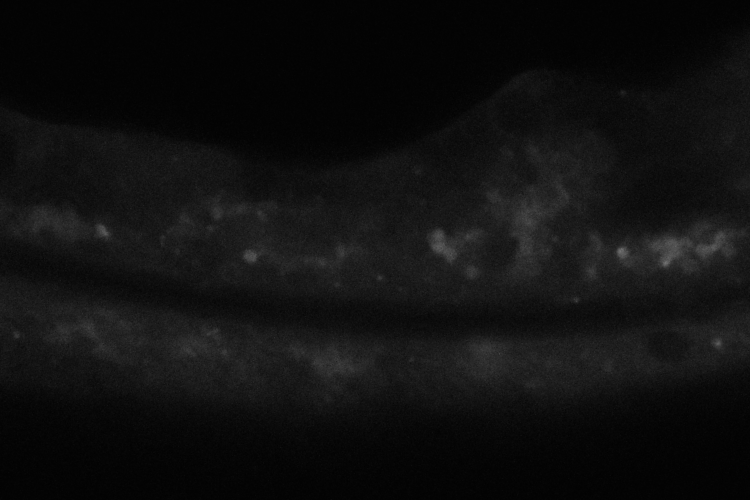

Supplement: Supplementary file 12 — Figure EV Source Data [file 44318_2025_367_MOESM12_ESM.zip › SD EV files/SD figure EV1/EV1A/EV_1_A_Roi/vps-60 (RNAi)/Gut /mCherry ART MC rab5 rab7 vps60 rnai front_0005-1-1-1-1.tif]

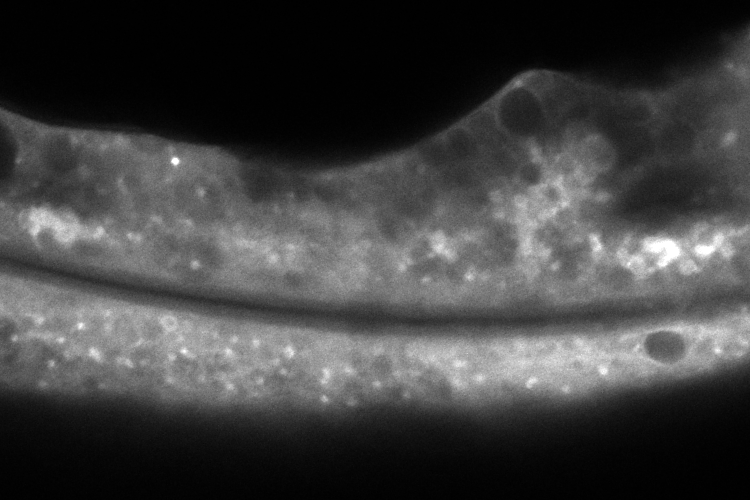

Supplement: Supplementary file 12 — Figure EV Source Data [file 44318_2025_367_MOESM12_ESM.zip › SD EV files/SD figure EV1/EV1A/EV_1_A_Roi/vps-60 (RNAi)/Gut /GFP ART G rab5 rab7 vps60 rnai front_0005-1-1-1-1.tif]

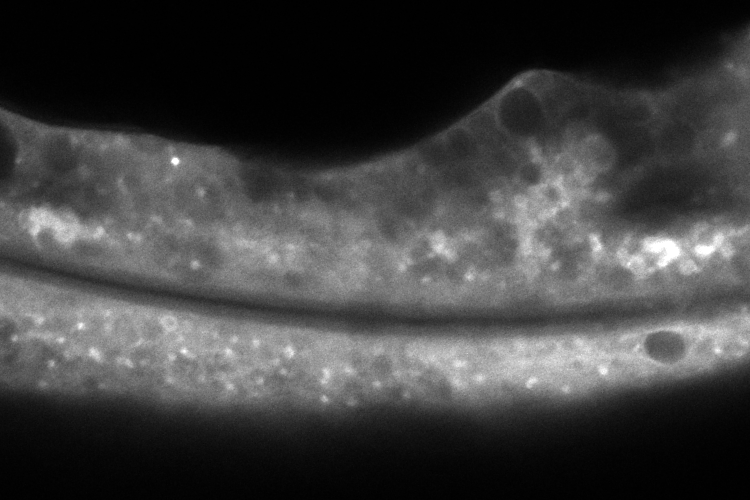

Supplement: Supplementary file 12 — Figure EV Source Data [file 44318_2025_367_MOESM12_ESM.zip › SD EV files/SD figure EV1/EV1A/EV_1_A_Roi/vps-60 (RNAi)/Gut /Merge ART MGM rab5 rab7 vps60 rnai front_0005-1-1-1.tif]

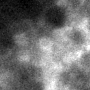

Supplement: Supplementary file 12 — Figure EV Source Data [file 44318_2025_367_MOESM12_ESM.zip › SD EV files/SD figure EV1/EV1A/EV_1_A_Roi/vps-60 (RNAi)/Gut close up/GFP ART C2 G rab5 rab7 vps60 rnai front_0005-1-1-1-1-1.tif]

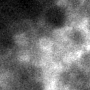

Supplement: Supplementary file 12 — Figure EV Source Data [file 44318_2025_367_MOESM12_ESM.zip › SD EV files/SD figure EV1/EV1A/EV_1_A_Roi/vps-60 (RNAi)/Gut close up/Merge ART C2 MGM rab5 rab7 vps60 rnai front_0005-1-1-1-1.tif]

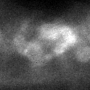

Supplement: Supplementary file 12 — Figure EV Source Data [file 44318_2025_367_MOESM12_ESM.zip › SD EV files/SD figure EV1/EV1A/EV_1_A_Roi/vps-60 (RNAi)/Gut close up/Merge ART C MGM rab5 rab7 vps60 rnai front_0005-1-1-1-1.tif]

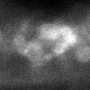

Supplement: Supplementary file 12 — Figure EV Source Data [file 44318_2025_367_MOESM12_ESM.zip › SD EV files/SD figure EV1/EV1A/EV_1_A_Roi/vps-60 (RNAi)/Gut close up/GFP ART C G rab5 rab7 vps60 rnai front_0005-1-1-1-1-1.tif]

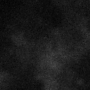

Supplement: Supplementary file 12 — Figure EV Source Data [file 44318_2025_367_MOESM12_ESM.zip › SD EV files/SD figure EV1/EV1A/EV_1_A_Roi/vps-60 (RNAi)/Gut close up/mCherry ART C2 MC rab5 rab7 vps60 rnai front_0005-1-1-1-1-1.tif]

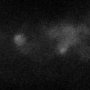

Supplement: Supplementary file 12 — Figure EV Source Data [file 44318_2025_367_MOESM12_ESM.zip › SD EV files/SD figure EV1/EV1A/EV_1_A_Roi/vps-60 (RNAi)/Gut close up/mCherry ART C MC rab5 rab7 vps60 rnai front_0005-1-1-1-1-1.tif]

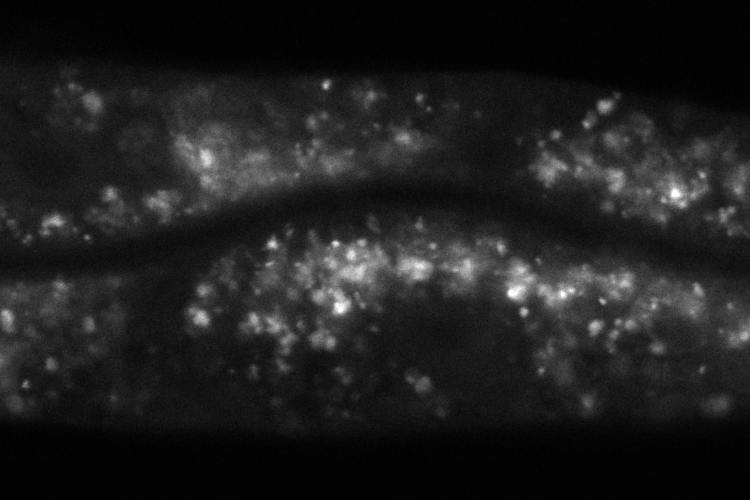

Supplement: Supplementary file 12 — Figure EV Source Data [file 44318_2025_367_MOESM12_ESM.zip › SD EV files/SD figure EV1/EV1A/EV_1_A_Roi/did-2 (RNAi)/Gut /mCherry ART MC rab5 rab7 did2 rnai front_0006-1-1-1-1.tif]

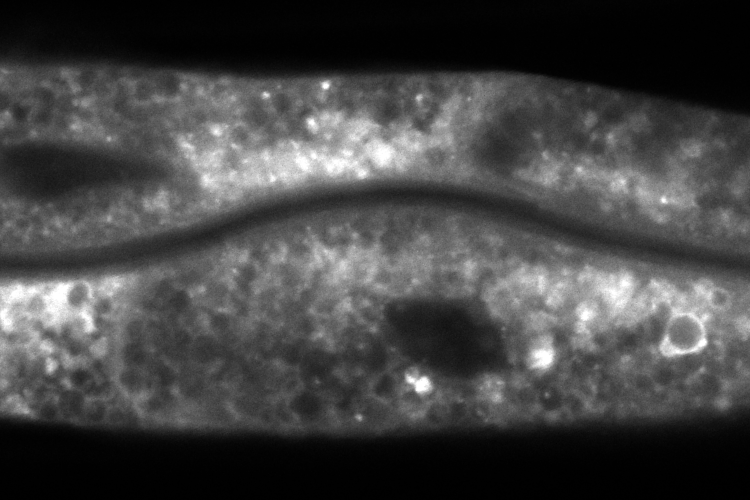

Supplement: Supplementary file 12 — Figure EV Source Data [file 44318_2025_367_MOESM12_ESM.zip › SD EV files/SD figure EV1/EV1A/EV_1_A_Roi/did-2 (RNAi)/Gut /Merge ART MGM rab5 rab7 did2 rnai front_0006-1-1-1.tif]

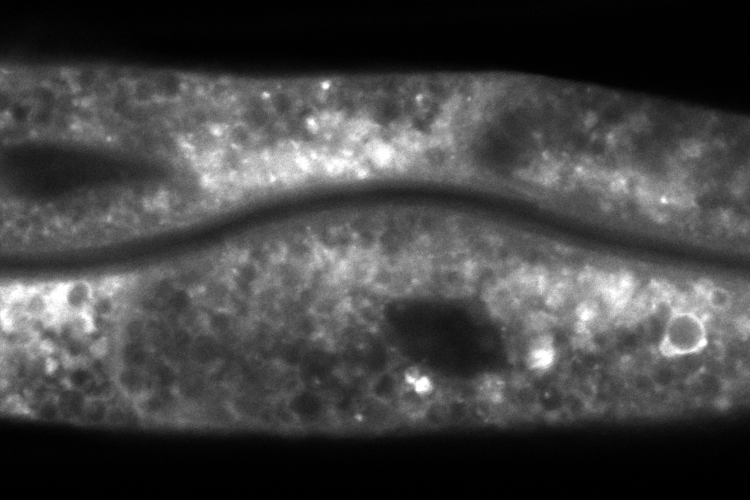

Supplement: Supplementary file 12 — Figure EV Source Data [file 44318_2025_367_MOESM12_ESM.zip › SD EV files/SD figure EV1/EV1A/EV_1_A_Roi/did-2 (RNAi)/Gut /GFP ART G rab5 rab7 did2 rnai front_0006-1-1-1-1.tif]

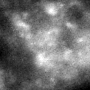

Supplement: Supplementary file 12 — Figure EV Source Data [file 44318_2025_367_MOESM12_ESM.zip › SD EV files/SD figure EV1/EV1A/EV_1_A_Roi/did-2 (RNAi)/Gut close up/GFP ART C3 G rab5 rab7 did2 rnai front_0006-1-1-1-1-1.tif]

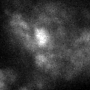

Supplement: Supplementary file 12 — Figure EV Source Data [file 44318_2025_367_MOESM12_ESM.zip › SD EV files/SD figure EV1/EV1A/EV_1_A_Roi/did-2 (RNAi)/Gut close up/mCherry ART C3 MC rab5 rab7 did2 rnai front_0006-1-1-1-1-1.tif]

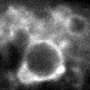

Supplement: Supplementary file 12 — Figure EV Source Data [file 44318_2025_367_MOESM12_ESM.zip › SD EV files/SD figure EV1/EV1A/EV_1_A_Roi/did-2 (RNAi)/Gut close up/Merge ART C MGM rab5 rab7 did2 rnai front_0006-1-1-1-1.tif]

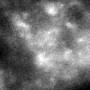

Supplement: Supplementary file 12 — Figure EV Source Data [file 44318_2025_367_MOESM12_ESM.zip › SD EV files/SD figure EV1/EV1A/EV_1_A_Roi/did-2 (RNAi)/Gut close up/Merge ART C3 MGM rab5 rab7 did2 rnai front_0006-1-1-1-1.tif]

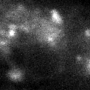

Supplement: Supplementary file 12 — Figure EV Source Data [file 44318_2025_367_MOESM12_ESM.zip › SD EV files/SD figure EV1/EV1A/EV_1_A_Roi/did-2 (RNAi)/Gut close up/mCherry ART C MC rab5 rab7 did2 rnai front_0006-1-1-1-1-1.tif]

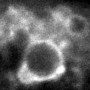

Supplement: Supplementary file 12 — Figure EV Source Data [file 44318_2025_367_MOESM12_ESM.zip › SD EV files/SD figure EV1/EV1A/EV_1_A_Roi/did-2 (RNAi)/Gut close up/GFP ART C G rab5 rab7 did2 rnai front_0006-1-1-1-1-1.tif]

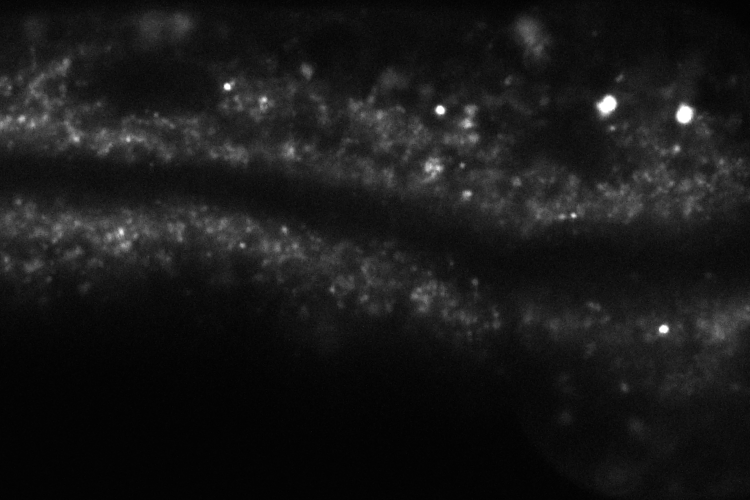

Supplement: Supplementary file 12 — Figure EV Source Data [file 44318_2025_367_MOESM12_ESM.zip › SD EV files/SD figure EV1/EV1A/EV_1_A_Roi/vps-37 (RNAi)/Gut /mCherry ART MC rab5 rab7 vps37 rnai front_0002-1-1-1-1.tif]

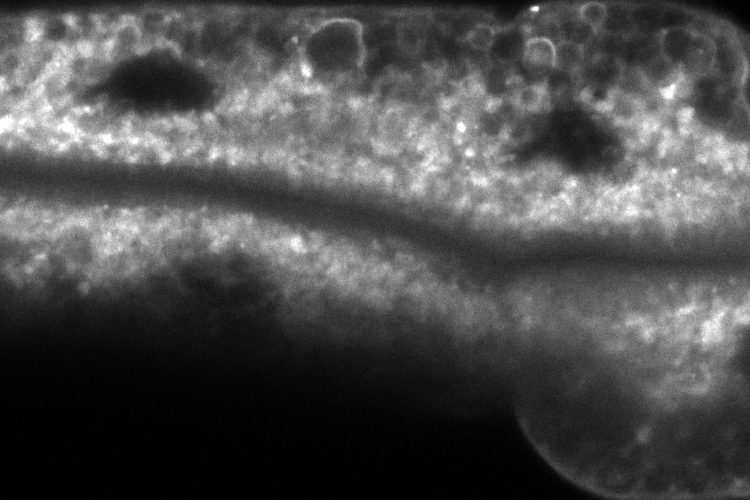

Supplement: Supplementary file 12 — Figure EV Source Data [file 44318_2025_367_MOESM12_ESM.zip › SD EV files/SD figure EV1/EV1A/EV_1_A_Roi/vps-37 (RNAi)/Gut /Merge ART MGM rab5 rab7 vps37 rnai front_0002-1-1-1.tif]

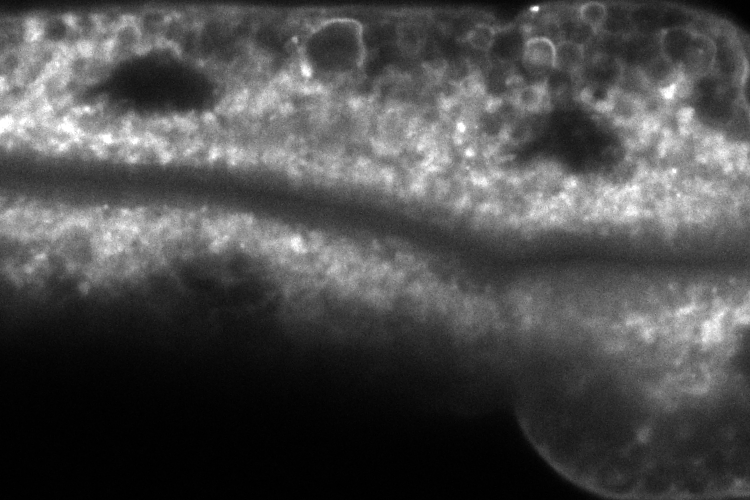

Supplement: Supplementary file 12 — Figure EV Source Data [file 44318_2025_367_MOESM12_ESM.zip › SD EV files/SD figure EV1/EV1A/EV_1_A_Roi/vps-37 (RNAi)/Gut /GFP ART G rab5 rab7 vps37 rnai front_0002-1-1-1-1.tif]

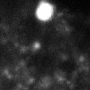

Supplement: Supplementary file 12 — Figure EV Source Data [file 44318_2025_367_MOESM12_ESM.zip › SD EV files/SD figure EV1/EV1A/EV_1_A_Roi/vps-37 (RNAi)/Gut close up/mCherry ART C MC rab5 rab7 vps37 rnai front_0002-1-1-1-1-1.tif]

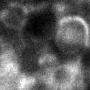

Supplement: Supplementary file 12 — Figure EV Source Data [file 44318_2025_367_MOESM12_ESM.zip › SD EV files/SD figure EV1/EV1A/EV_1_A_Roi/vps-37 (RNAi)/Gut close up/Merge ART C2 MGM rab5 rab7 vps37 rnai front_0002-1-1-1-1.tif]

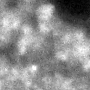

Supplement: Supplementary file 12 — Figure EV Source Data [file 44318_2025_367_MOESM12_ESM.zip › SD EV files/SD figure EV1/EV1A/EV_1_A_Roi/vps-37 (RNAi)/Gut close up/GFP ART C G rab5 rab7 vps37 rnai front_0002-1-1-1-1-1.tif]

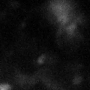

Supplement: Supplementary file 12 — Figure EV Source Data [file 44318_2025_367_MOESM12_ESM.zip › SD EV files/SD figure EV1/EV1A/EV_1_A_Roi/vps-37 (RNAi)/Gut close up/mCherry ART C2 MC rab5 rab7 vps37 rnai front_0002-1-1-1-1-1.tif]

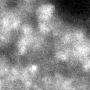

Supplement: Supplementary file 12 — Figure EV Source Data [file 44318_2025_367_MOESM12_ESM.zip › SD EV files/SD figure EV1/EV1A/EV_1_A_Roi/vps-37 (RNAi)/Gut close up/Merge ART C MGM rab5 rab7 vps37 rnai front_0002-1-1-1-1.tif]

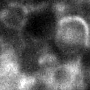

Supplement: Supplementary file 12 — Figure EV Source Data [file 44318_2025_367_MOESM12_ESM.zip › SD EV files/SD figure EV1/EV1A/EV_1_A_Roi/vps-37 (RNAi)/Gut close up/GFP ART C2 G rab5 rab7 vps37 rnai front_0002-1-1-1-1-1.tif]

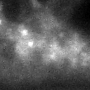

Supplement: Supplementary file 12 — Figure EV Source Data [file 44318_2025_367_MOESM12_ESM.zip › SD EV files/SD figure EV1/EV1B/EV_1_B_Roi/did-2 (RNAi) pre fed/Gut close up/Merge ART C2 MGM rab5 raby sand1 did2 rnai preefed front_0003-1-1-1-1-1.tif]

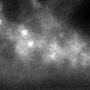

Supplement: Supplementary file 12 — Figure EV Source Data [file 44318_2025_367_MOESM12_ESM.zip › SD EV files/SD figure EV1/EV1B/EV_1_B_Roi/did-2 (RNAi) pre fed/Gut close up/GFP ART C2 G rab5 raby sand1 did2 rnai preefed front_0003-1-1-1-1-1-1.tif]

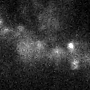

Supplement: Supplementary file 12 — Figure EV Source Data [file 44318_2025_367_MOESM12_ESM.zip › SD EV files/SD figure EV1/EV1B/EV_1_B_Roi/did-2 (RNAi) pre fed/Gut close up/mCherry ART C2 MC rab5 raby sand1 did2 rnai preefed front_0003-1-1-1-1-1-1.tif]

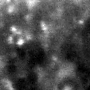

Supplement: Supplementary file 12 — Figure EV Source Data [file 44318_2025_367_MOESM12_ESM.zip › SD EV files/SD figure EV1/EV1B/EV_1_B_Roi/did-2 (RNAi) pre fed/Gut close up/Merge ART C MGM rab5 raby sand1 did2 rnai preefed front_0003-1-1-1-1-1.tif]

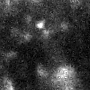

Supplement: Supplementary file 12 — Figure EV Source Data [file 44318_2025_367_MOESM12_ESM.zip › SD EV files/SD figure EV1/EV1B/EV_1_B_Roi/did-2 (RNAi) pre fed/Gut close up/mCherry ART C MC rab5 raby sand1 did2 rnai preefed front_0003-1-1-1-1-1-1.tif]

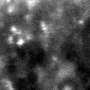

Supplement: Supplementary file 12 — Figure EV Source Data [file 44318_2025_367_MOESM12_ESM.zip › SD EV files/SD figure EV1/EV1B/EV_1_B_Roi/did-2 (RNAi) pre fed/Gut close up/GFP ART C G rab5 raby sand1 did2 rnai preefed front_0003-1-1-1-1-1-1.tif]

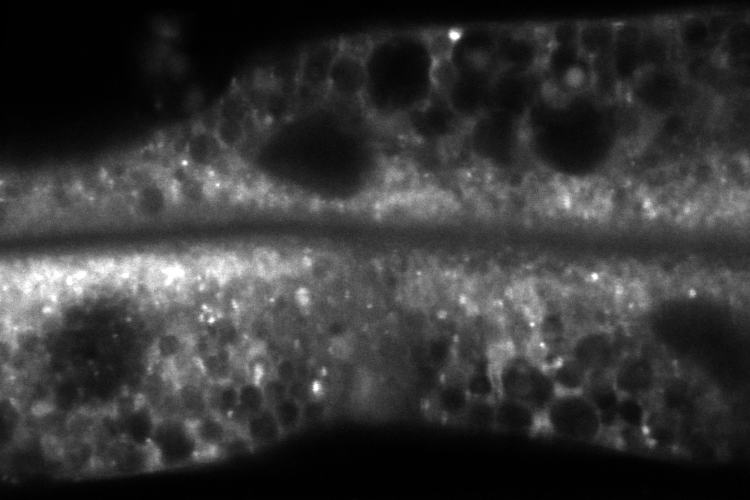

Supplement: Supplementary file 12 — Figure EV Source Data [file 44318_2025_367_MOESM12_ESM.zip › SD EV files/SD figure EV1/EV1B/EV_1_B_Roi/did-2 (RNAi) pre fed/Gut/GFP ART G rab5 raby sand1 did2 rnai preefed front_0003-1-1-1-1-1.tif]

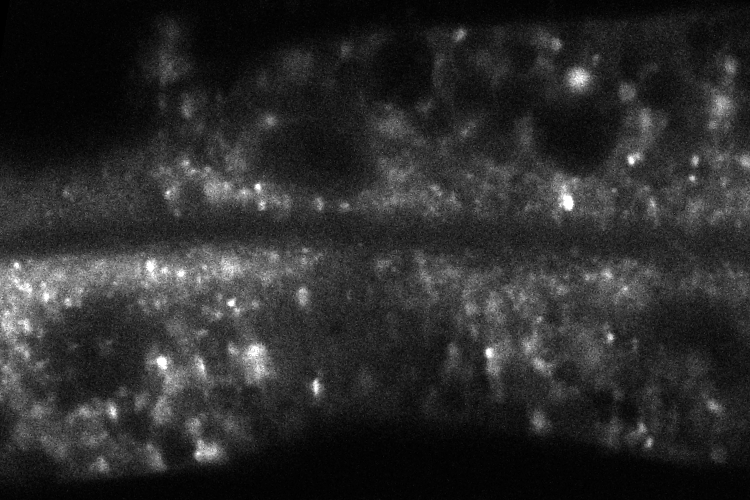

Supplement: Supplementary file 12 — Figure EV Source Data [file 44318_2025_367_MOESM12_ESM.zip › SD EV files/SD figure EV1/EV1B/EV_1_B_Roi/did-2 (RNAi) pre fed/Gut/mCherry ART MC rab5 raby sand1 did2 rnai preefed front_0003-1-1-1-1-1.tif]

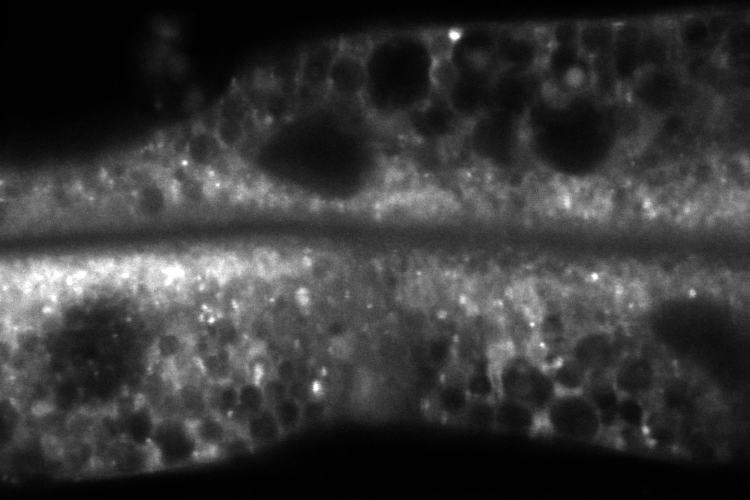

Supplement: Supplementary file 12 — Figure EV Source Data [file 44318_2025_367_MOESM12_ESM.zip › SD EV files/SD figure EV1/EV1B/EV_1_B_Roi/did-2 (RNAi) pre fed/Gut/Merge ART MGM rab5 raby sand1 did2 rnai preefed front_0003-1-1-1-1.tif]

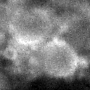

Supplement: Supplementary file 12 — Figure EV Source Data [file 44318_2025_367_MOESM12_ESM.zip › SD EV files/SD figure EV1/EV1B/EV_1_B_Roi/Mock/Gut close up/GFP ART C G rab5 rab7 sand1 control rnai front_0006-1-1-1-1-1.tif]

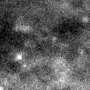

Supplement: Supplementary file 12 — Figure EV Source Data [file 44318_2025_367_MOESM12_ESM.zip › SD EV files/SD figure EV1/EV1B/EV_1_B_Roi/Mock/Gut close up/Merge ART C2 MGM rab5 rab7 sand1 control rnai front_0006-1-1-1-1.tif]

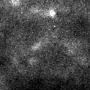

Supplement: Supplementary file 12 — Figure EV Source Data [file 44318_2025_367_MOESM12_ESM.zip › SD EV files/SD figure EV1/EV1B/EV_1_B_Roi/Mock/Gut close up/mCherry ART C MC rab5 rab7 sand1 control rnai front_0006-1-1-1-1-1.tif]

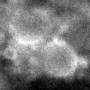

Supplement: Supplementary file 12 — Figure EV Source Data [file 44318_2025_367_MOESM12_ESM.zip › SD EV files/SD figure EV1/EV1B/EV_1_B_Roi/Mock/Gut close up/Merge ART C MGM rab5 rab7 sand1 control rnai front_0006-1-1-1-1.tif]

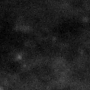

Supplement: Supplementary file 12 — Figure EV Source Data [file 44318_2025_367_MOESM12_ESM.zip › SD EV files/SD figure EV1/EV1B/EV_1_B_Roi/Mock/Gut close up/GFP ART C2 G rab5 rab7 sand1 control rnai front_0006-1-1-1-1-1.tif]

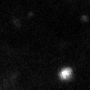

Supplement: Supplementary file 12 — Figure EV Source Data [file 44318_2025_367_MOESM12_ESM.zip › SD EV files/SD figure EV1/EV1B/EV_1_B_Roi/Mock/Gut close up/mCherry ART C2 MC rab5 rab7 sand1 control rnai front_0006-1-1-1-1-1.tif]

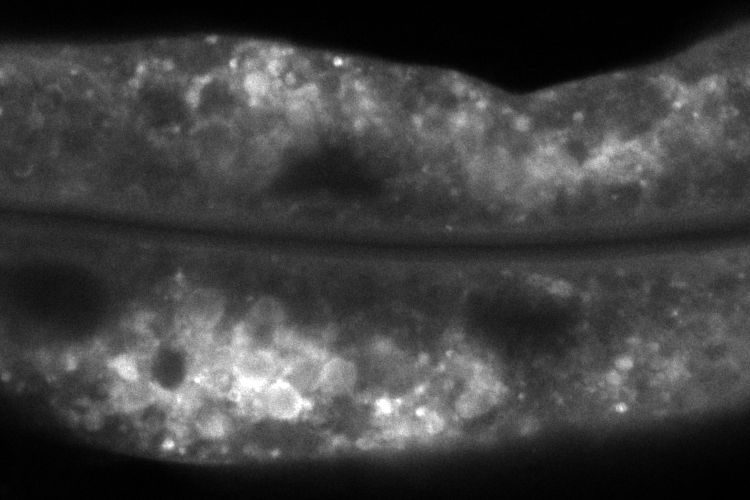

Supplement: Supplementary file 12 — Figure EV Source Data [file 44318_2025_367_MOESM12_ESM.zip › SD EV files/SD figure EV1/EV1B/EV_1_B_Roi/Mock/Gut/GFP ART G rab5 rab7 sand1 control rnai front_0006-1-1-1-1.tif]

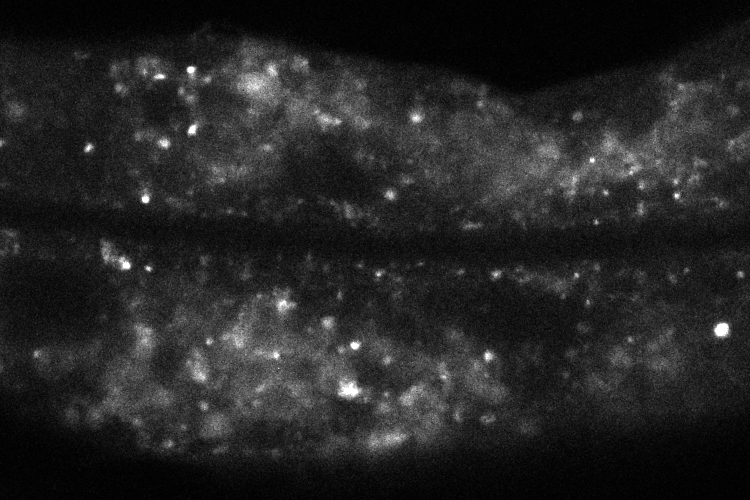

Supplement: Supplementary file 12 — Figure EV Source Data [file 44318_2025_367_MOESM12_ESM.zip › SD EV files/SD figure EV1/EV1B/EV_1_B_Roi/Mock/Gut/mCherry ART MC rab5 rab7 sand1 control rnai front_0006-1-1-1-1.tif]

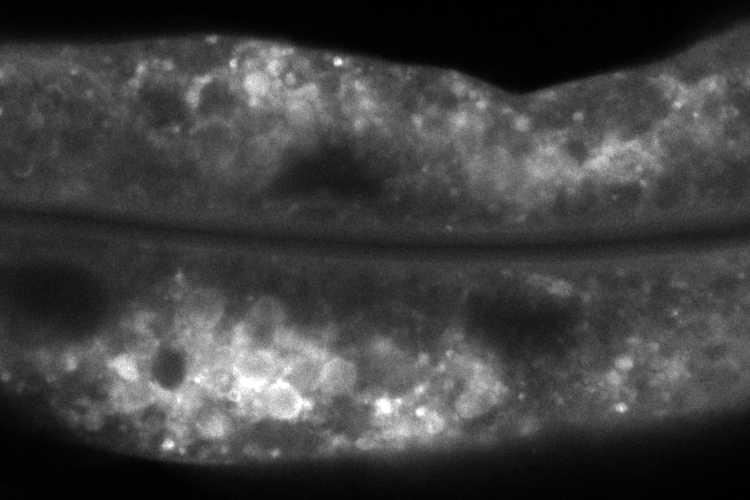

Supplement: Supplementary file 12 — Figure EV Source Data [file 44318_2025_367_MOESM12_ESM.zip › SD EV files/SD figure EV1/EV1B/EV_1_B_Roi/Mock/Gut/Merge ART MGM rab5 rab7 sand1 control rnai front_0006-1-1-1.tif]

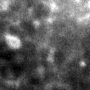

Supplement: Supplementary file 12 — Figure EV Source Data [file 44318_2025_367_MOESM12_ESM.zip › SD EV files/SD figure EV1/EV1B/EV_1_B_Roi/vps-28 (RNAi)/Gut close up/GFP ART C G rab5&rab7 sand1 vps28 rnai front_0009-1-1-1-1-1-1.tif]

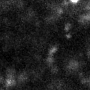

Supplement: Supplementary file 12 — Figure EV Source Data [file 44318_2025_367_MOESM12_ESM.zip › SD EV files/SD figure EV1/EV1B/EV_1_B_Roi/vps-28 (RNAi)/Gut close up/mCherry ART C MC rab5&rab7 sand1 vps28 rnai front_0009-1-1-1-1-1-1.tif]

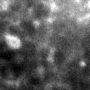

Supplement: Supplementary file 12 — Figure EV Source Data [file 44318_2025_367_MOESM12_ESM.zip › SD EV files/SD figure EV1/EV1B/EV_1_B_Roi/vps-28 (RNAi)/Gut close up/Merge ART C MGM rab5&rab7 sand1 vps28 rnai front_0009-1-1-1-1-1.tif]

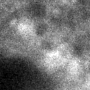

Supplement: Supplementary file 12 — Figure EV Source Data [file 44318_2025_367_MOESM12_ESM.zip › SD EV files/SD figure EV1/EV1B/EV_1_B_Roi/vps-28 (RNAi)/Gut close up/Merge ART C2 MGM rab5&rab7 sand1 vps28 rnai front_0009-1-1-1-1-1.tif]

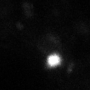

Supplement: Supplementary file 12 — Figure EV Source Data [file 44318_2025_367_MOESM12_ESM.zip › SD EV files/SD figure EV1/EV1B/EV_1_B_Roi/vps-28 (RNAi)/Gut close up/mCherry ART C2 MC rab5&rab7 sand1 vps28 rnai front_0009-1-1-1-1-1-1.tif]

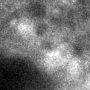

Supplement: Supplementary file 12 — Figure EV Source Data [file 44318_2025_367_MOESM12_ESM.zip › SD EV files/SD figure EV1/EV1B/EV_1_B_Roi/vps-28 (RNAi)/Gut close up/GFP ART C2 G rab5&rab7 sand1 vps28 rnai front_0009-1-1-1-1-1-1.tif]

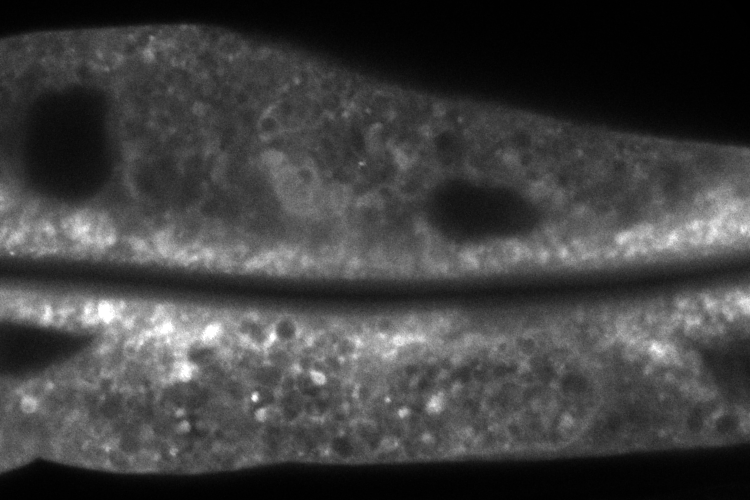

Supplement: Supplementary file 12 — Figure EV Source Data [file 44318_2025_367_MOESM12_ESM.zip › SD EV files/SD figure EV1/EV1B/EV_1_B_Roi/vps-28 (RNAi)/Gut/GFP ART G rab5&rab7 sand1 vps28 rnai front_0009-1-1-1-1-1.tif]

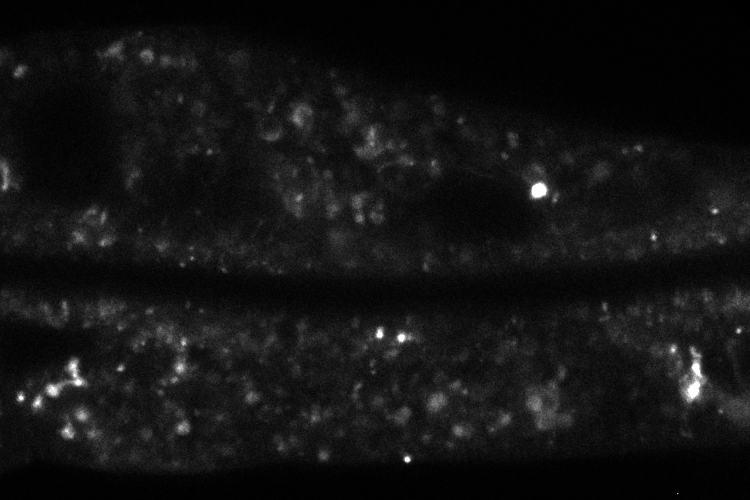

Supplement: Supplementary file 12 — Figure EV Source Data [file 44318_2025_367_MOESM12_ESM.zip › SD EV files/SD figure EV1/EV1B/EV_1_B_Roi/vps-28 (RNAi)/Gut/mCherry ART MC rab5&rab7 sand1 vps28 rnai front_0009-1-1-1-1-1.tif]

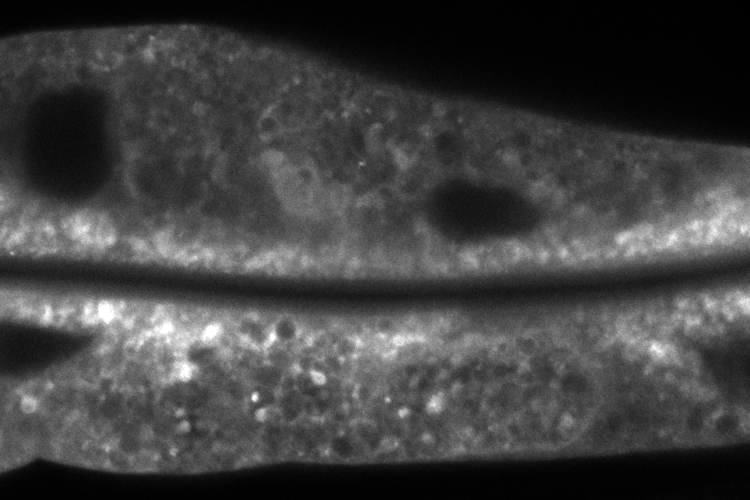

Supplement: Supplementary file 12 — Figure EV Source Data [file 44318_2025_367_MOESM12_ESM.zip › SD EV files/SD figure EV1/EV1B/EV_1_B_Roi/vps-28 (RNAi)/Gut/Merge ART MGM rab5&rab7 sand1 vps28 rnai front_0009-1-1-1-1.tif]

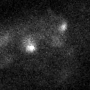

Supplement: Supplementary file 12 — Figure EV Source Data [file 44318_2025_367_MOESM12_ESM.zip › SD EV files/SD figure EV1/EV1B/EV_1_B_Roi/vps-24 (RNAi)/Gut close up/mCherry ART C2 MC rab5 rab7 sand1 vps24 rnai front_0001-1-1-1-1-1-1.tif]

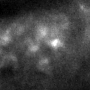

Supplement: Supplementary file 12 — Figure EV Source Data [file 44318_2025_367_MOESM12_ESM.zip › SD EV files/SD figure EV1/EV1B/EV_1_B_Roi/vps-24 (RNAi)/Gut close up/GFP ART C2 G rab5 rab7 sand1 vps24 rnai front_0001-1-1-1-1-1-1.tif]

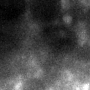

Supplement: Supplementary file 12 — Figure EV Source Data [file 44318_2025_367_MOESM12_ESM.zip › SD EV files/SD figure EV1/EV1B/EV_1_B_Roi/vps-24 (RNAi)/Gut close up/GFP ART C G rab5 rab7 sand1 vps24 rnai front_0001-1-1-1-1-1-1.tif]

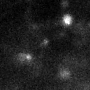

Supplement: Supplementary file 12 — Figure EV Source Data [file 44318_2025_367_MOESM12_ESM.zip › SD EV files/SD figure EV1/EV1B/EV_1_B_Roi/vps-24 (RNAi)/Gut close up/mCherry ART C MC rab5 rab7 sand1 vps24 rnai front_0001-1-1-1-1-1-1.tif]

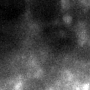

Supplement: Supplementary file 12 — Figure EV Source Data [file 44318_2025_367_MOESM12_ESM.zip › SD EV files/SD figure EV1/EV1B/EV_1_B_Roi/vps-24 (RNAi)/Gut close up/Merge ART C MGM rab5 rab7 sand1 vps24 rnai front_0001-1-1-1-1-1.tif]

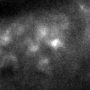

Supplement: Supplementary file 12 — Figure EV Source Data [file 44318_2025_367_MOESM12_ESM.zip › SD EV files/SD figure EV1/EV1B/EV_1_B_Roi/vps-24 (RNAi)/Gut close up/Merge ART C2 MGM rab5 rab7 sand1 vps24 rnai front_0001-1-1-1-1-1.tif]

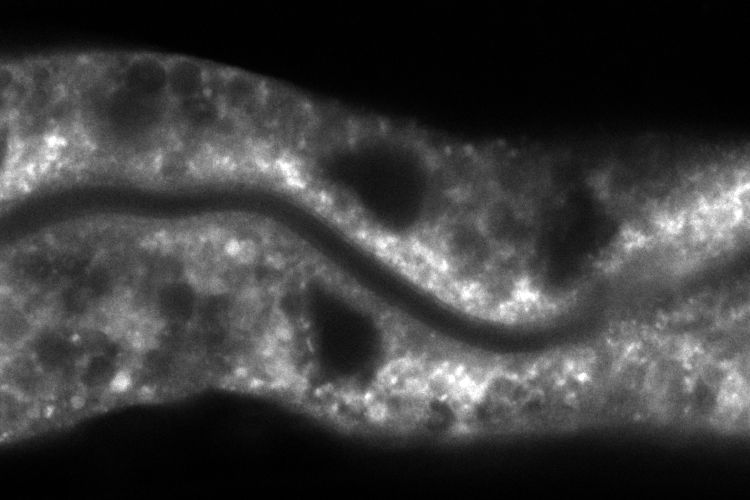

Supplement: Supplementary file 12 — Figure EV Source Data [file 44318_2025_367_MOESM12_ESM.zip › SD EV files/SD figure EV1/EV1B/EV_1_B_Roi/vps-24 (RNAi)/Gut/Merge ART MGM rab5 rab7 sand1 vps24 rnai front_0001-1-1-1-1.tif]

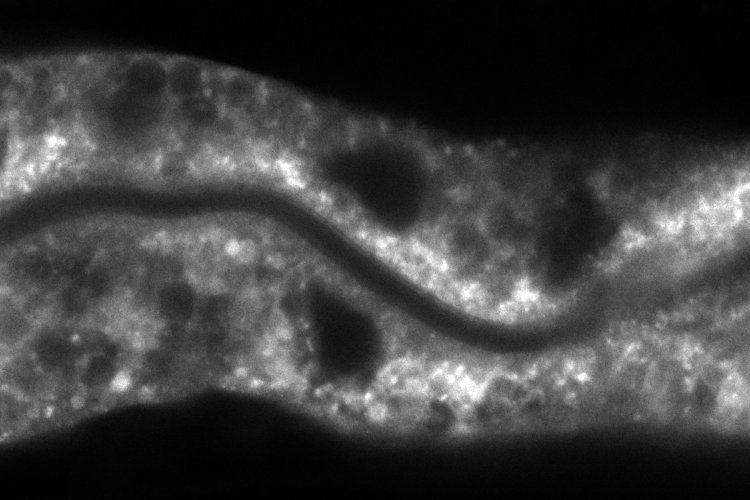

Supplement: Supplementary file 12 — Figure EV Source Data [file 44318_2025_367_MOESM12_ESM.zip › SD EV files/SD figure EV1/EV1B/EV_1_B_Roi/vps-24 (RNAi)/Gut/GFP ART G rab5 rab7 sand1 vps24 rnai front_0001-1-1-1-1-1.tif]

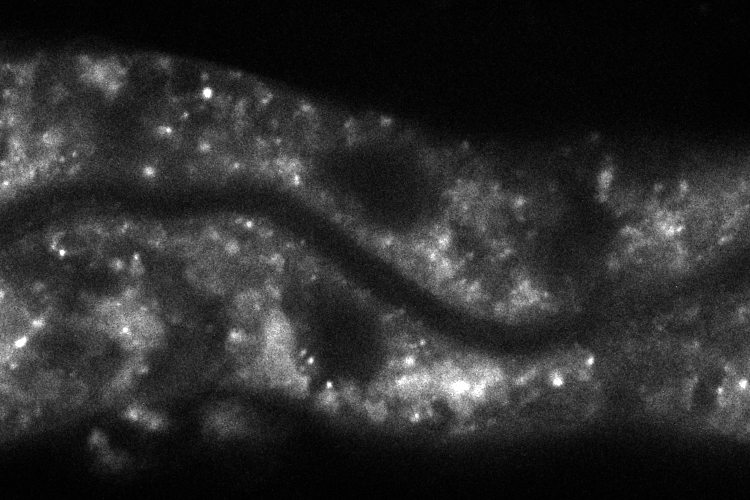

Supplement: Supplementary file 12 — Figure EV Source Data [file 44318_2025_367_MOESM12_ESM.zip › SD EV files/SD figure EV1/EV1B/EV_1_B_Roi/vps-24 (RNAi)/Gut/mCherry ART MC rab5 rab7 sand1 vps24 rnai front_0001-1-1-1-1-1.tif]

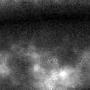

Supplement: Supplementary file 12 — Figure EV Source Data [file 44318_2025_367_MOESM12_ESM.zip › SD EV files/SD figure EV1/EV1B/EV_1_B_Roi/vps-32.1 (RNAi) pre fed/Gut close up/Merge ART C2 MGM rab5 rab7 sand1 pre fed vps32.1 rnai front_0005-1-1-1-1-1.tif]

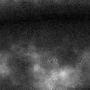

Supplement: Supplementary file 12 — Figure EV Source Data [file 44318_2025_367_MOESM12_ESM.zip › SD EV files/SD figure EV1/EV1B/EV_1_B_Roi/vps-32.1 (RNAi) pre fed/Gut close up/GFP ART C2 G rab5 rab7 sand1 pre fed vps32.1 rnai front_0005-1-1-1-1-1-1.tif]

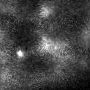

Supplement: Supplementary file 12 — Figure EV Source Data [file 44318_2025_367_MOESM12_ESM.zip › SD EV files/SD figure EV1/EV1B/EV_1_B_Roi/vps-32.1 (RNAi) pre fed/Gut close up/mCherry ART C MC rab5 rab7 sand1 pre fed vps32.1 rnai front_0005-1-1-1-1-1-1.tif]

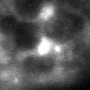

Supplement: Supplementary file 12 — Figure EV Source Data [file 44318_2025_367_MOESM12_ESM.zip › SD EV files/SD figure EV1/EV1B/EV_1_B_Roi/vps-32.1 (RNAi) pre fed/Gut close up/GFP ART C G rab5 rab7 sand1 pre fed vps32.1 rnai front_0005-1-1-1-1-1-1.tif]

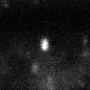

Supplement: Supplementary file 12 — Figure EV Source Data [file 44318_2025_367_MOESM12_ESM.zip › SD EV files/SD figure EV1/EV1B/EV_1_B_Roi/vps-32.1 (RNAi) pre fed/Gut close up/mCherry ART C2 MC rab5 rab7 sand1 pre fed vps32.1 rnai front_0005-1-1-1-1-1-1.tif]

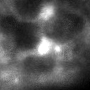

Supplement: Supplementary file 12 — Figure EV Source Data [file 44318_2025_367_MOESM12_ESM.zip › SD EV files/SD figure EV1/EV1B/EV_1_B_Roi/vps-32.1 (RNAi) pre fed/Gut close up/Merge ART C MGM rab5 rab7 sand1 pre fed vps32.1 rnai front_0005-1-1-1-1-1.tif]

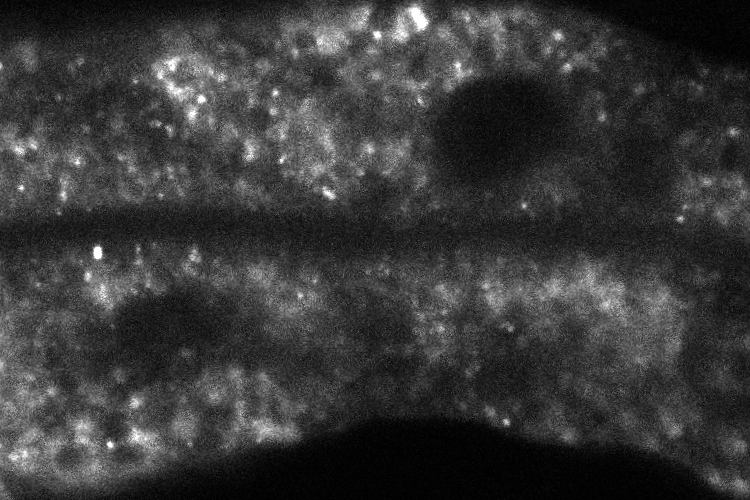

Supplement: Supplementary file 12 — Figure EV Source Data [file 44318_2025_367_MOESM12_ESM.zip › SD EV files/SD figure EV1/EV1B/EV_1_B_Roi/vps-32.1 (RNAi) pre fed/Gut/mCherry ART MC rab5 rab7 sand1 pre fed vps32.1 rnai front_0005-1-1-1-1-1.tif]

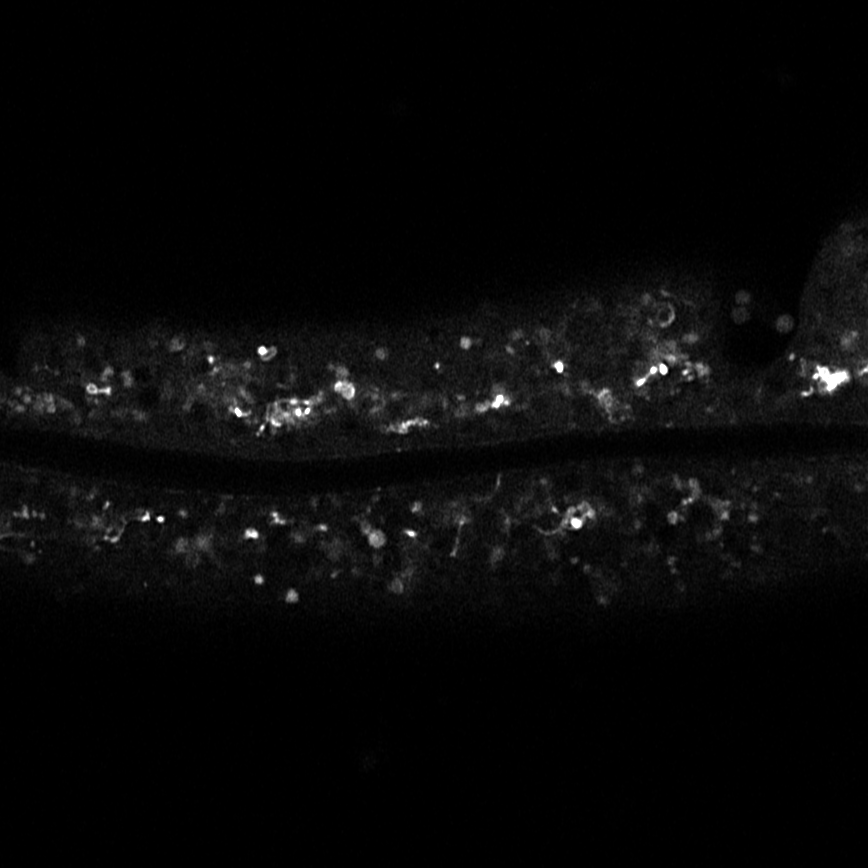

Supplement: Supplementary file 13 — Appendix Source Data [file 44318_2025_367_MOESM13_ESM.zip › SD Appendix files/SD Appendix figure S3/App 3B/App_Fig_3_B_data/Mock/2024_08_06_RABX-5_RAB-5_neg_08_Airyscan Processing-1.tif]

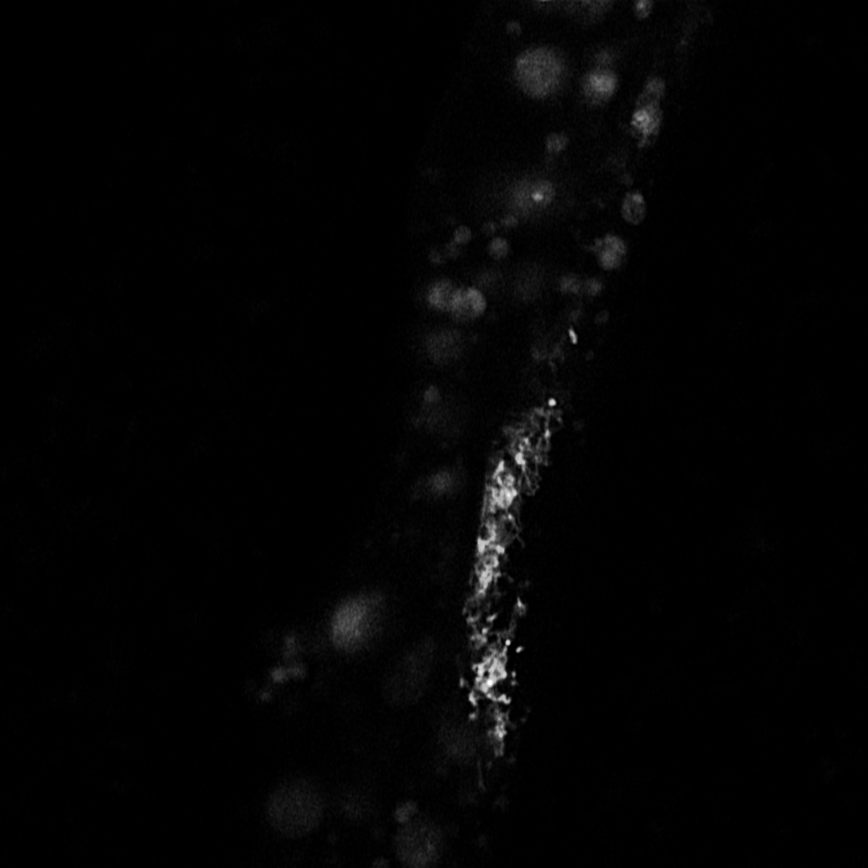

Supplement: Supplementary file 13 — Appendix Source Data [file 44318_2025_367_MOESM13_ESM.zip › SD Appendix files/SD Appendix figure S3/App 3B/App_Fig_3_B_data/ubq + hgrs-1 (RNAi)/2024_08_13_RABX-5_RAB-5_hgrs-1_ubq-1_14_Airyscan Processing-1.tif]

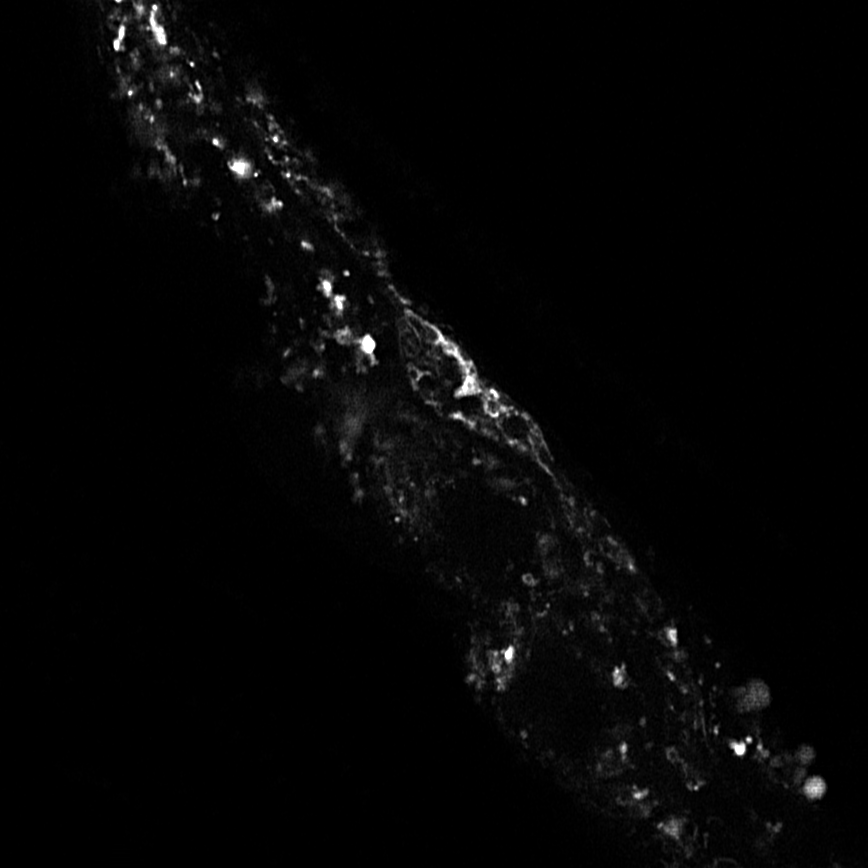

Supplement: Supplementary file 13 — Appendix Source Data [file 44318_2025_367_MOESM13_ESM.zip › SD Appendix files/SD Appendix figure S3/App 3B/App_Fig_3_B_data/ubq + hgrs-1 (RNAi)/2024_08_13_RABX-5_RAB-5_hgrs-1_ubq-1_01_Airyscan Processing-1.tif]

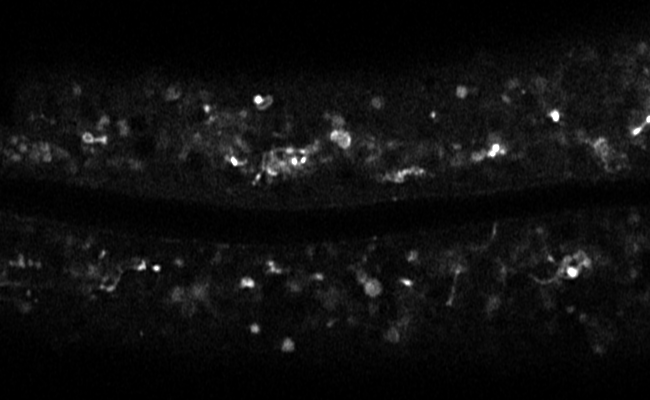

Supplement: Supplementary file 13 — Appendix Source Data [file 44318_2025_367_MOESM13_ESM.zip › SD Appendix files/SD Appendix figure S3/App 3B/App_Fig_3_B_Roi/Mock/Gut/C1-2024_08_06_RABX-5_RAB-5_neg_08_Airyscan Processing-1-1.tif]

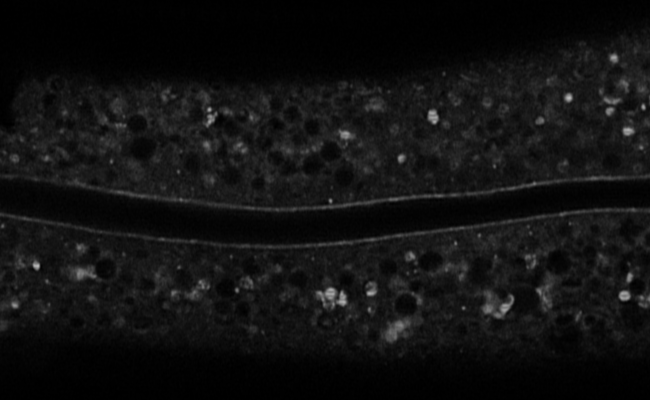

Supplement: Supplementary file 13 — Appendix Source Data [file 44318_2025_367_MOESM13_ESM.zip › SD Appendix files/SD Appendix figure S3/App 3B/App_Fig_3_B_Roi/Mock/Gut/C2-2024_08_06_RABX-5_RAB-5_neg_08_Airyscan Processing-1-1.tif]

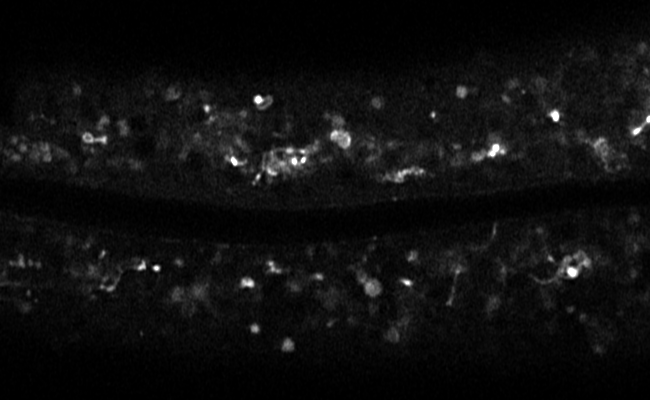

Supplement: Supplementary file 13 — Appendix Source Data [file 44318_2025_367_MOESM13_ESM.zip › SD Appendix files/SD Appendix figure S3/App 3B/App_Fig_3_B_Roi/Mock/Gut/2024_08_06_RABX-5_RAB-5_neg_08_Airyscan Processing-1-1.tif]

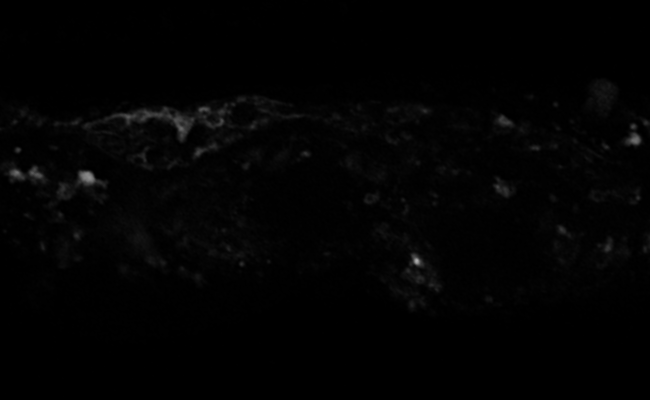

Supplement: Supplementary file 13 — Appendix Source Data [file 44318_2025_367_MOESM13_ESM.zip › SD Appendix files/SD Appendix figure S3/App 3B/App_Fig_3_B_Roi/ubq + hgrs-1 (RNAi)/Gut/C1-2024_08_13_RABX-5_RAB-5_hgrs-1_ubq-1_01_Airyscan Processing-1-1.tif]

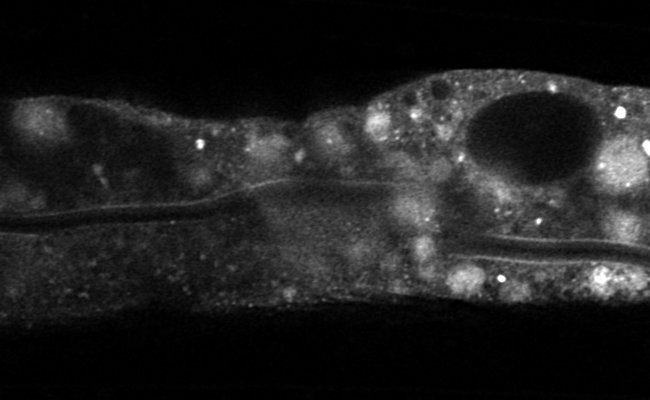

Supplement: Supplementary file 13 — Appendix Source Data [file 44318_2025_367_MOESM13_ESM.zip › SD Appendix files/SD Appendix figure S3/App 3B/App_Fig_3_B_Roi/ubq + hgrs-1 (RNAi)/Gut/C2-2024_08_13_RABX-5_RAB-5_hgrs-1_ubq-1_14_Airyscan Processing-1-1.tif]

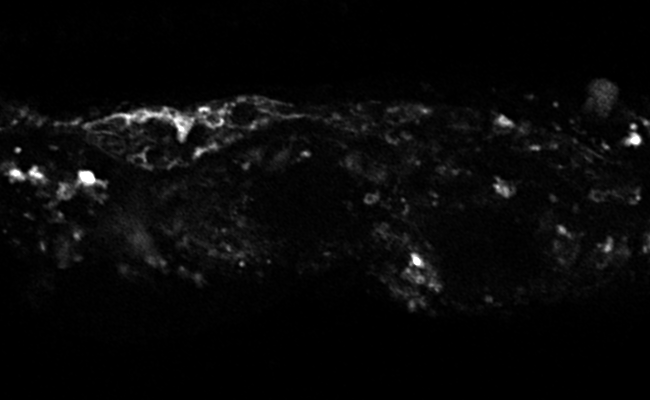

Supplement: Supplementary file 13 — Appendix Source Data [file 44318_2025_367_MOESM13_ESM.zip › SD Appendix files/SD Appendix figure S3/App 3B/App_Fig_3_B_Roi/ubq + hgrs-1 (RNAi)/Gut/2024_08_13_RABX-5_RAB-5_hgrs-1_ubq-1_01_Airyscan Processing-1-1.tif]

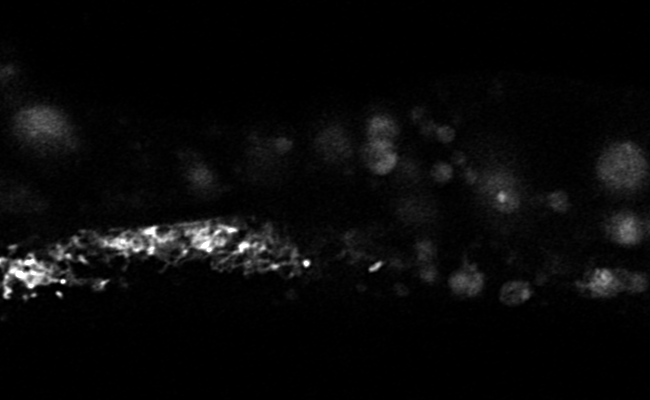

Supplement: Supplementary file 13 — Appendix Source Data [file 44318_2025_367_MOESM13_ESM.zip › SD Appendix files/SD Appendix figure S3/App 3B/App_Fig_3_B_Roi/ubq + hgrs-1 (RNAi)/Gut/2024_08_13_RABX-5_RAB-5_hgrs-1_ubq-1_14_Airyscan Processing-1-1.tif]
