# Supplementary figures and images for: Coordination between ESCRT function and Rab conversion during endosome maturation (part 9 of 9)
Source: EMBO J. 2025 Feb 5;44(6):1574–607. doi: 10.1038/s44318-025-00367-7 (PMC11914609; doi:10.1038/s44318-025-00367-7)

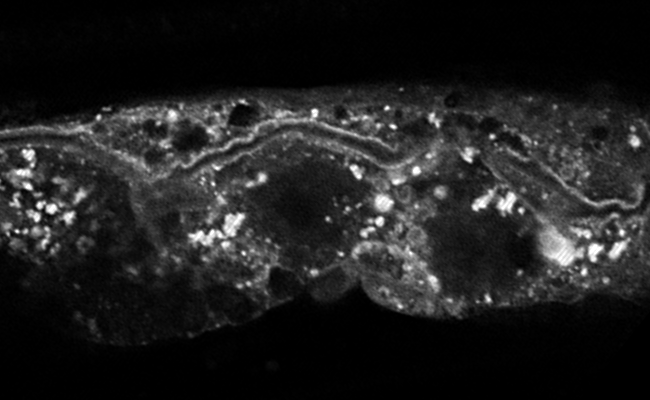

Supplement: Supplementary file 13 — Appendix Source Data [file 44318_2025_367_MOESM13_ESM.zip › SD Appendix files/SD Appendix figure S3/App 3B/App_Fig_3_B_Roi/ubq + hgrs-1 (RNAi)/Gut/C2-2024_08_13_RABX-5_RAB-5_hgrs-1_ubq-1_01_Airyscan Processing-1-1.tif]

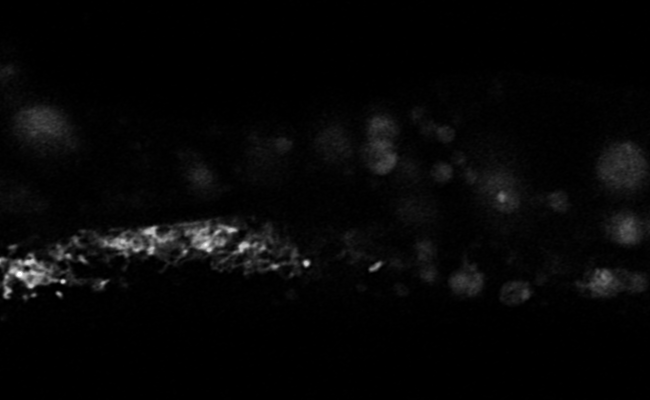

Supplement: Supplementary file 13 — Appendix Source Data [file 44318_2025_367_MOESM13_ESM.zip › SD Appendix files/SD Appendix figure S3/App 3B/App_Fig_3_B_Roi/ubq + hgrs-1 (RNAi)/Gut/C1-2024_08_13_RABX-5_RAB-5_hgrs-1_ubq-1_14_Airyscan Processing-1-1.tif]
