# Supplementary material for: Coordination between ESCRT function and Rab conversion during endosome maturation
Source: EMBO J. 2025 Feb 5;44(6):1574–607. doi: 10.1038/s44318-025-00367-7 (PMC11914609; doi:10.1038/s44318-025-00367-7)
Supplement: Supplementary file 14 — Expanded View Figures [file 44318_2025_367_MOESM14_ESM.pdf]

Expanded View Figures

Figure EV1. Knockdowns of core early and late ESCRT factors as well as knockdowns of additional ESCRT-III factors affect endosome maturation in WT and *sand-1(KO)*, related to Fig. 1.

(**A**) Additional ESCRT knockdowns performed in the ESCRT RNAi screen shown in Fig. 1A. White arrowheads pointing to GFP::RAB-5 and mCherry::RAB-7 positive structures, respectively in the individual channels. In the merges colocalization events are marked via yellow arrowheads (signals: RAB-5 > green and RAB-7 > magenta). (**B**) Further ESCRT knockdowns performed in the ESCRT RNAi screen shown in Fig. 1B. Consistent with data shown in Fig. 1B, knockdowns of further core early ESCRTs or a core late ESCRT factor cause no further enlargement of the through *sand-1(KO)* enlarged GFP::RAB-5 positive structures and have only minor effects on the colocalization of GFP::RAB-5 and mCherry::RAB-7. Similar effects on the colocalization of GFP::RAB-5 and mCherry::RAB-7 and the GFP::RAB-5 structure size are also observable in knockdowns of additional ESCRT-III factors. White arrowheads pointing to GFP::RAB-5 and mCherry::RAB-7 positive structures, respectively in the individual channels. In the merges colocalization events are marked via yellow arrowheads (signals: RAB-5 > green and RAB-7 > magenta). Data information: Merges were individually adjusted in all panels. Representative pictures with magnifications (white box) on the right are shown for each experiment (scale bars: 10 µm (main pictures) and 1 µm (magnifications)). Unprocessed images are available as source data. Source data are available online for this figure.

© The Author(s)

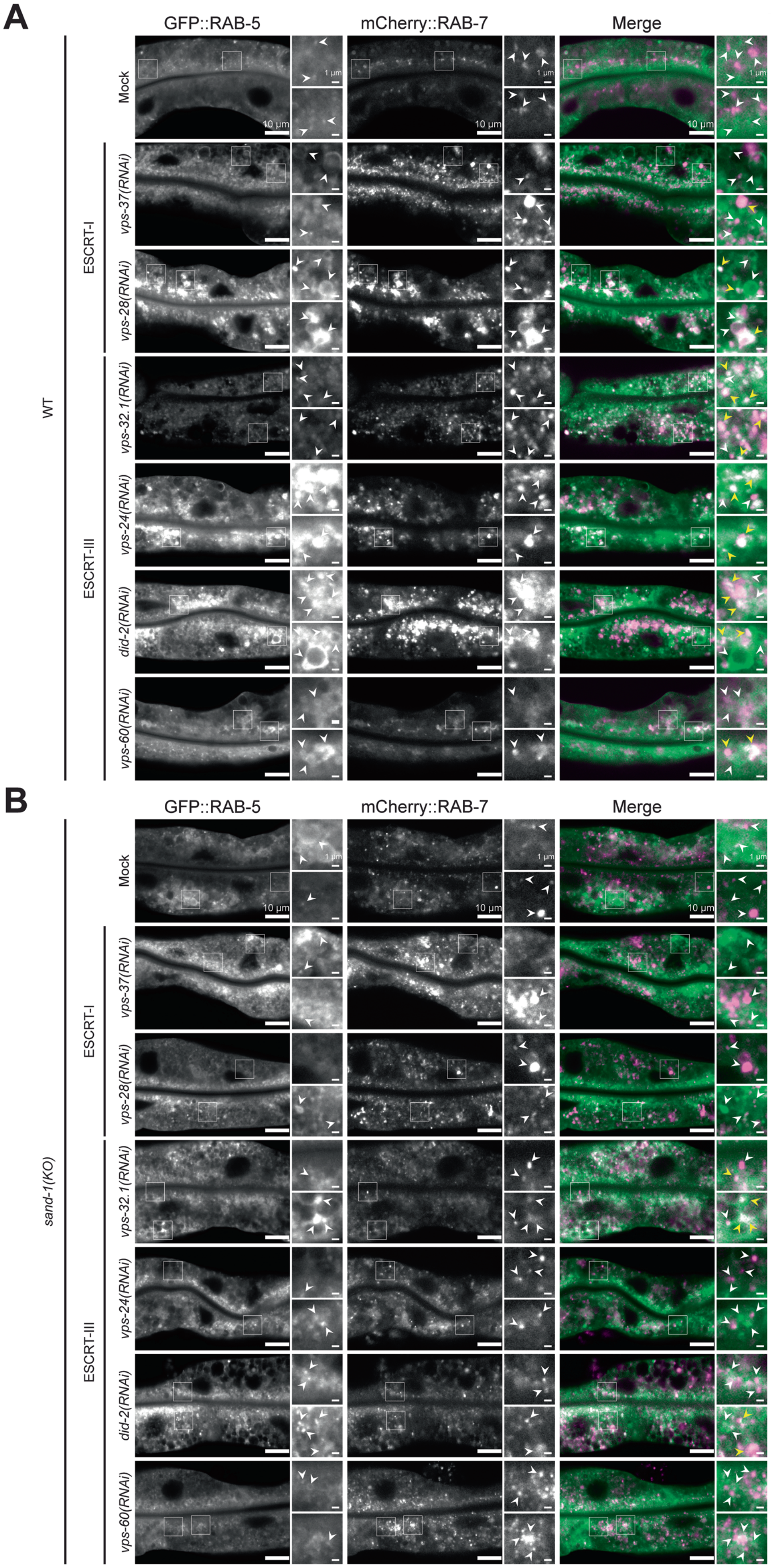
A
GFP::RAB-5
mCherry::RAB-7
Merge
Mock
vps-37(RNAi)
vps-28(RNAi)
vps-32.1(RNAi)
vps-24(RNAi)
did-2(RNAi)
vps-60(RNAi)
ESCRT-I
ESCRT-III
WT
10 µm
1 µm
B
GFP::RAB-5
mCherry::RAB-7
Merge
Mock
vps-37(RNAi)
vps-28(RNAi)
vps-32.1(RNAi)
vps-24(RNAi)
did-2(RNAi)
vps-60(RNAi)
ESCRT-I
ESCRT-III
sand-1(KO)

 © The Author(s)

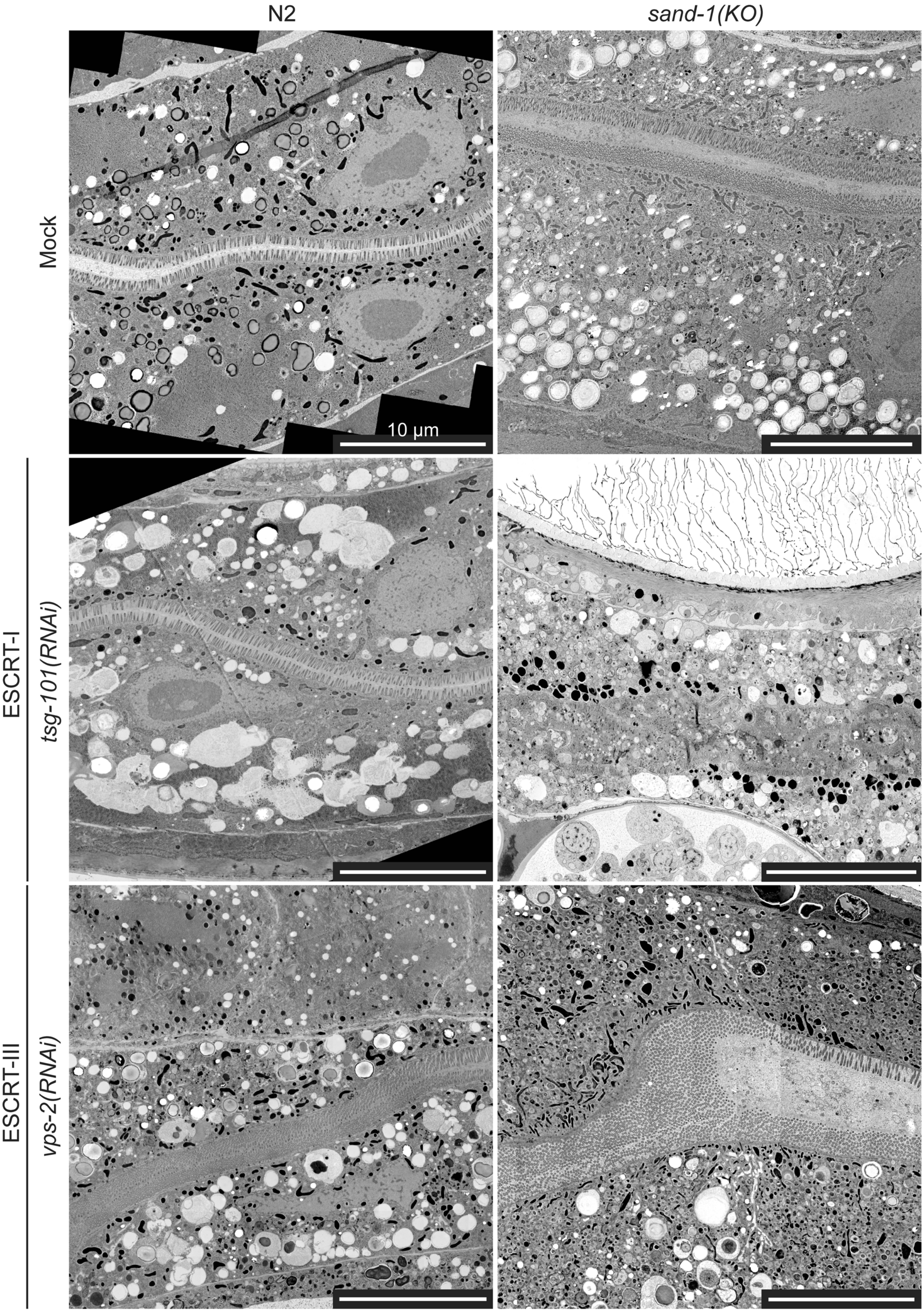
N2
sand-1(KO)
Mock
10 µm
ESCRT-I
tsg-101(RNAi)
ESCRT-III
vps-2(RNAi)

Figure EV2. ESCRT knockdowns affect the intracellular morphology in WT and *sand-1(KO)*, related to Fig. 2.

TEM overview pictures belonging to experiments shown in (Fig. 2A, C, E). The *sand-1(KO)* causes the formation of large granular structures accumulating in the basal region but does not increase the MVB size. Knockdown of *tsg-101* increases the size of endosomal structures, whereas *vps-2* knockdown causes no major changes in this regard but leads to the formation of additional small and big granular structures. The observed ESCRT RNAi effect are strain background independent. Data information: Representative overview TEM pictures are shown for each examined condition. Overview electron micrographs are individually adjusted and were generated with unstitched data sets (scale bars 10 µm). Unprocessed images are available as source data and online in the BioImage Resource.

 © The Author(s)

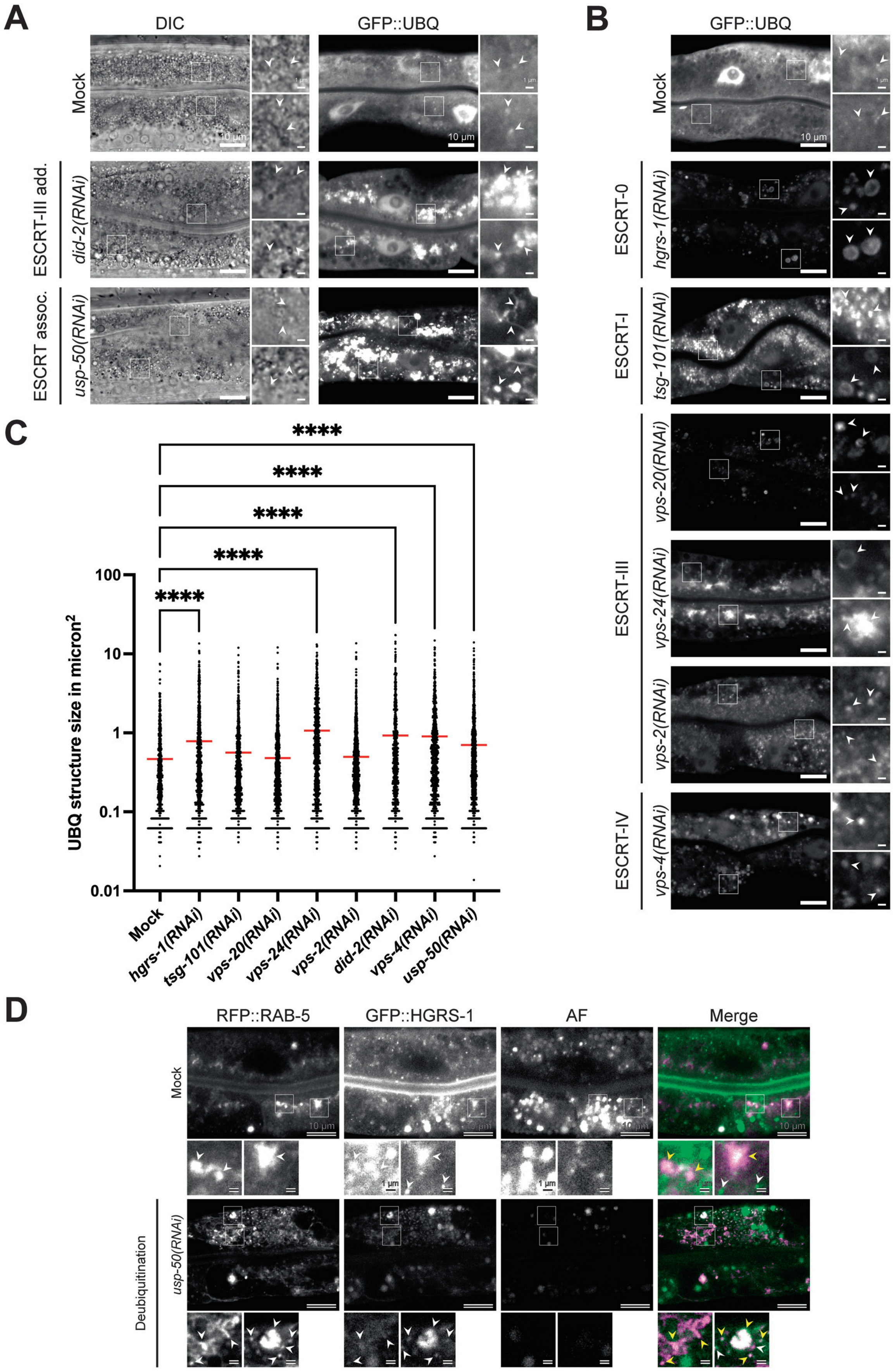
A
DIC
GFP::UBQ
Mock
ESCRT-III add.
did-2(RNAi)
ESCRT assoc.
usp-50(RNAi)
10 µm
1 µm
B
GFP::UBQ
Mock
ESCRT-0
hgrs-1(RNAi)
ESCRT-I
tsg-101(RNAi)
ESCRT-III
vps-20(RNAi)
vps-24(RNAi)
vps-2(RNAi)
ESCRT-IV
vps-4(RNAi)
C
UBQ structure size in micron²
100
10
1
0.1
0.01
****
Mock
hgrs-1(RNAi)
tsg-101(RNAi)
vps-20(RNAi)
vps-24(RNAi)
vps-2(RNAi)
did-2(RNAi)
vps-4(RNAi)
usp-50(RNAi)
D
RFP::RAB-5
GFP::HGRS-1
AF
Merge
Mock
Deubiquitination
usp-50(RNAi)

Figure EV3. *usp-50* and *did-2* knockdown cause UBQ aggregation in the cytoplasm and USP-50 presence is required for HGRS-1 stability in *C. elegans*, related to Fig. 4.

(**A**) The knockdown of *did-2* has only minor effects on nuclear GFP::UBQ levels and cause the formation of GFP::UBQ accumulations in addition to enlarged structures. *usp-50(RNAi)* however, generates GFP::UBQ accumulations, enlarged structures, tubular networks and strongly reduces nuclear GFP::UBQ levels. White arrowheads pointing to GFP::UBQ positive structures and corresponding areas in the DIC, respectively. (**B**) Images of early and late ESCRT knockdowns from Fig. 4 adjusted to equal brightness settings. *hgrs-1(RNAi)* and *vps-20(RNAi)* show strongly reduced GFP::UBQ levels in comparison to Mock as well as to the other ESCRT knockdowns. Some of the individual signals are marked by white arrowheads. (**C**) Quantification of the GFP::UBQ positive structure sizes belonging to (**A**) and Fig. 4A. The individual sizes of the measured GFP::UBQ positive structures are displayed for each examined condition in micron^2. For analysis shown in the graph 10 worms per condition ($n = 10$) and more than 9500 structures in total ($n > 9500$) were examined ($n = 3$ independent experiments). The mean particle size is shown for each condition as red line in the graph and the data are shown in log 10 scale (Y-axis). Obtained *P*-values: Mock vs. *hgrs-1(RNAi)* $P = 2.2E-6$, Mock vs. *tsg-101(RNAi)* $P = 0.3887$, Mock vs. *vps-20(RNAi)* $P > 0.9999$, Mock vs. *vps-24(RNAi)* $P < 1.0E-15$, Mock vs. *vps-2(RNAi)* $P = 0.1113$, Mock vs. *did-2(RNAi)* $P = 6.2E-9$, Mock vs. *vps-4(RNAi)* $P < 1.0E-15$, Mock vs. *usp-50(RNAi)* $P = 4.5E-7$. (**D**) The knockdown of *usp-50* causes a strong reduction of the GFP::HGRS-1 signal and generates tubular, network like RFP::RAB-5 positive structures and RFP::RAB-5 positive aggregates. In addition, some of the remaining GFP::HGRS-1 positive structures are also positive for RFP::RAB-5. White arrowheads marking GFP::HGRS-1 and RFP::RAB-5 positive structures, respectively in the individual channels. Yellow arrowheads indicating colocalization events in the merges (Signals: HGRS-1 > green and RAB-5 > magenta). The autofluorescence channel (AF) is not shown in the merge for simplification. Data information: Representative pictures with corresponding enlargements (white box) next to it are shown for each experiment (scale bars: 10 µm (main pictures) and 1 µm (enlargements)) in (**A**, **B** and **D**). Belonging DIC pictures elucidate the extent of the gut, marker independent are depicted for all experiments showing in (**A**). Kruskal–Wallis test with Dunn's multiple comparisons test was performed to determine the statistical significance between the examined conditions (**C**). Significance levels are displayed in the graph as $****P \le 0.0001$. Comparisons with $P > 0.05$ are not shown for simplification. Unprocessed images and statistical raw data are available as source data. Source data are available online for this figure.

 © The Author(s)

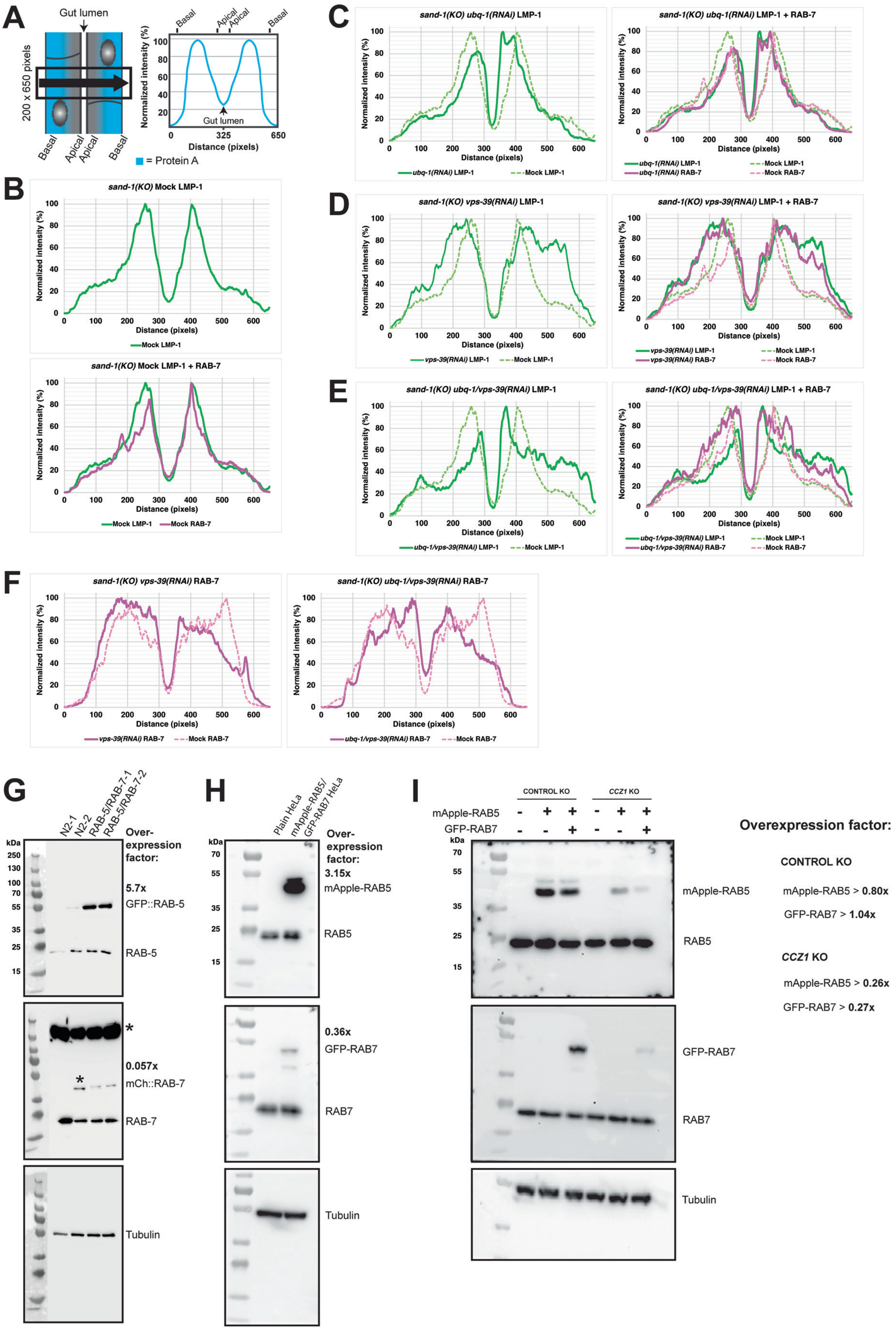
A
Gut lumen
200 x 650 pixels
Basal
Apical
Apical
Basal
Normalized intensity (%)
Distance (pixels)
Gut lumen
= Protein A
B
sand-1(KO) Mock LMP-1
Mock LMP-1
sand-1(KO) Mock LMP-1 + RAB-7
Mock LMP-1
Mock RAB-7
C
sand-1(KO) ubq-1(RNAi) LMP-1
ubq-1(RNAi) LMP-1
Mock LMP-1
sand-1(KO) ubq-1(RNAi) LMP-1 + RAB-7
ubq-1(RNAi) RAB-7
Mock RAB-7
D
sand-1(KO) vps-39(RNAi) LMP-1
vps-39(RNAi) LMP-1
sand-1(KO) vps-39(RNAi) LMP-1 + RAB-7
vps-39(RNAi) RAB-7
E
sand-1(KO) ubq-1/vps-39(RNAi) LMP-1
ubq-1/vps-39(RNAi) LMP-1
sand-1(KO) ubq-1/vps-39(RNAi) LMP-1 + RAB-7
ubq-1/vps-39(RNAi) RAB-7
F
sand-1(KO) vps-39(RNAi) RAB-7
sand-1(KO) ubq-1/vps-39(RNAi) RAB-7
G
N2-1
N2-2
RAB-5/RAB-7-1
RAB-5/RAB-7-2
kDa
Over-expression factor:
5.7x
GFP::RAB-5
RAB-5
0.057x
mCh::RAB-7
RAB-7
Tubulin
H
Plain HeLa
mApple-RAB5/ GFP-RAB7 HeLa
3.15x
mApple-RAB5
RAB5
0.36x
GFP-RAB7
RAB7
Tubulin
I
CONTROL KO
CCZ1 KO
mApple-RAB5
GFP-RAB7
RAB5
RAB7
Tubulin
Overexpression factor:
CONTROL KO
mApple-RAB5 > 0.80x
GFP-RAB7 > 1.04x
CCZ1 KO
mApple-RAB5 > 0.26x
GFP-RAB7 > 0.27x

Figure EV4. LMP-1 and RAB-7 area localizations in *sand-1(KO)* under reduced UBQ levels and knockdowns of *vps-39* and western blots of tagged RAB proteins in *C. elegans* strains and cell lines, related to Figs. 5, 6 and worm and cell culture experiments.

(**A**) Schematic representation of the approach used to generate the area plots shown in Figs. 5, 6 and EV4. A simplified worm gut with intestinal cells expressing a blue example protein is shown on the left side. The localization of this protein gets measured via a ROI (200 × 650 pixels) spanning the whole worm gut. This creates an area plot, highlighting the localization of the protein in the intestinal cells, shown on the right side. (**B**, **C**) Area plots belonging to Fig. 5I, K. Overexpression of mCherry::RAB-7 causes an apical localization of LMP-1::GFP which can be further augmented through RNAi mediated UBQ level reduction. (**D**, **E**) Area plots belonging to Fig. 6C, D. The knockdown of HOPS subunit *vps-39* abolishes the partial rescue of *sand-1(KO)* mediated by the mCherry::RAB-7 overexpression and causes a more basal distribution of LMP-1::GFP. This localization shift is less prominent if the UBQ levels are reduced together with the *vps-39* knockdown. (**F**) Examined mCherry::RAB-7 area localization belonging to Fig. 6A. The knockdown of *vps-39* impairs the recruitment of mCherry::RAB-7 to apical structures caused by the reduction of UBQ levels. The control group is the same like shown in Fig. 5B and the data were collected and analyzed like described for this experiment. (**G**) Western blot analysis of overexpression in worms carrying GFP::RAB-5 and mCherry::RAB-7 inserted transgenes (used in Fig. 1, Fig. 5, Fig. 6, Fig. 7, Fig. EV1 and Fig. EV5). The indicated worm lysates were loaded ($n = 2$ biological replicates) for N2 (wild-type) and RAB-5/RAB-7 worms with transgenes. The relative overexpression was calculated by the ratio between the endogenous lower band and the tagged upper band. The overexpression factor is indicated and is the mean of the two replicates (GFP::RAB-5: 5.7x, mCherry::RAB-7: 0.057x). The asterisks denote unspecific bands in the RAB-7 blot (middle panel). Tubulin was used as a loading control. (**H**) Western blot analysis of overexpression in HeLa cells stably expressing mApple-RAB5 and GFP-RAB7 was done (used in Fig. 8). The cell lysates from control and stably expressing mApple-RAB5 and GFP-RAB7 were separated by SDS PAGE. The relative overexpression was calculated by the ratio between the endogenous lower band and the tagged upper band. The overexpression factor is indicated and is a mean of three replicates (mApple-RAB5: 3.15x, GFP-RAB7: 0.36x). Tubulin was used as a loading control. (**I**) Western blot analysis of overexpression of transiently transfected mApple-RAB5 and GFP-RAB7 in Control KO and *CCZ1* KO cells was done (used in Fig. 8). The cell lysates from aforementioned conditions were separated by SDS PAGE. The relative overexpression was calculated by the ratio between the endogenous lower band and the tagged upper band. The overexpression factor is indicated and is a mean of three replicates (Control KO > mApple-RAB5: 0.8x, GFP-RAB7: 1.04x; *CCZ1* KO > mApple-RAB5: 0.26x, GFP-RAB7: 0.27x). Tubulin was used as a loading control. Data information: The individual mCherry::RAB-7 area plots for the merge area plots (**B–E**) are shown in Figs. 5K and 6D. Statistical raw data are available as source data (**B–I**). Source data are available online for this figure.

 © The Author(s)

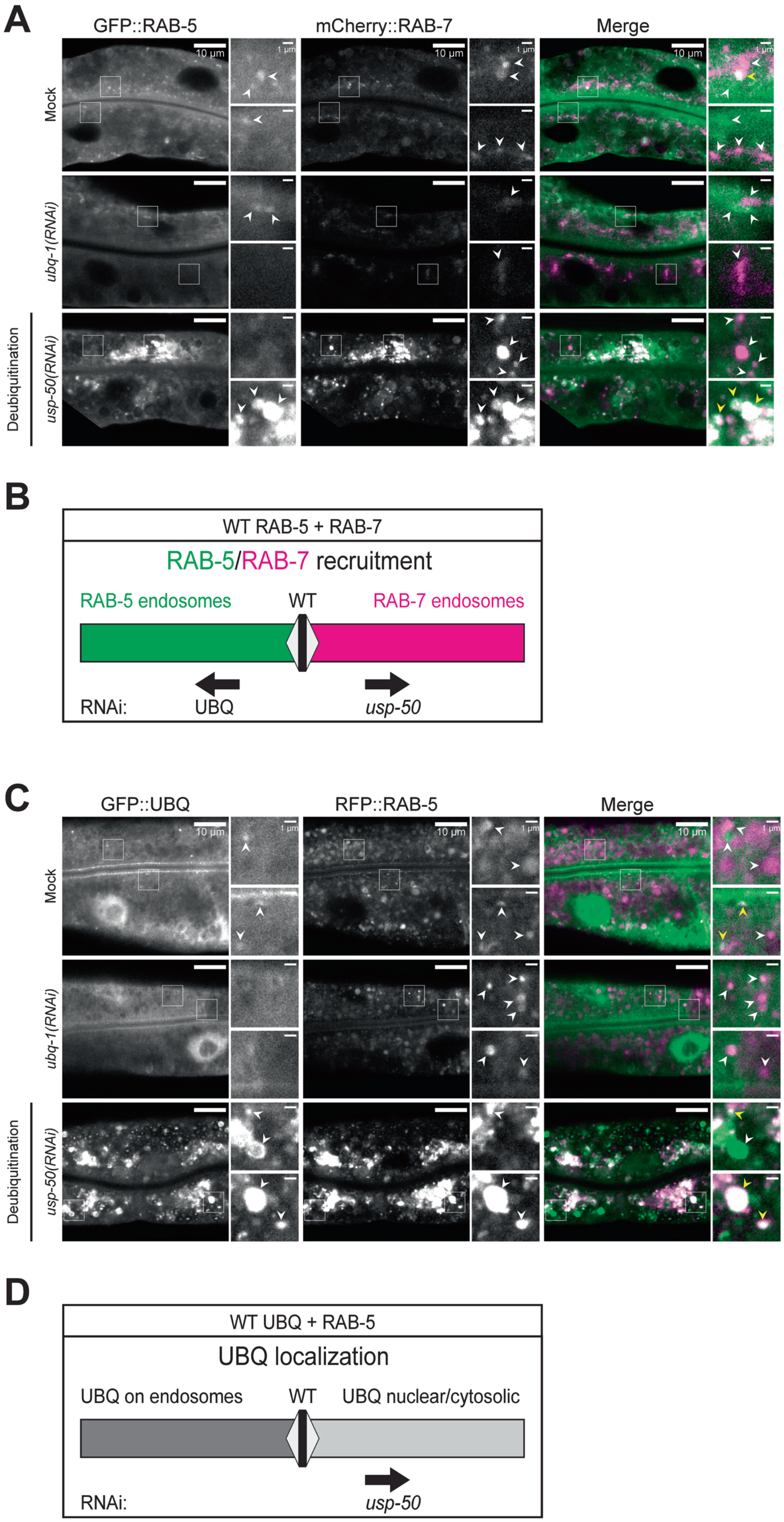
A
GFP::RAB-5
mCherry::RAB-7
Merge
10 µm
1 µm
Mock
ubq-1(RNAi)
Deubiquitination
usp-50(RNAi)
B
WT RAB-5 + RAB-7
RAB-5/RAB-7 recruitment
RAB-5 endosomes
WT
RAB-7 endosomes
RNAi:
UBQ
usp-50
C
GFP::UBQ
RFP::RAB-5
Merge
10 µm
1 µm
Mock
ubq-1(RNAi)
Deubiquitination
usp-50(RNAi)
D
WT UBQ + RAB-5
UBQ localization
UBQ on endosomes
WT
UBQ nuclear/cytosolic
RNAi:
usp-50

© The Author(s)

Figure EV5. UBQ reduction reduces the number of RAB-5 structures and *usp-50(RNAi)* causes colocalization of RAB-5 with RAB-7 and UBQ.

(**A**) The reduction of the UBQ level via RNAi causes a depletion of GFP::RAB-5 positive structures. Knockdown of *usp-50* causes a mislocalization of GFP::RAB and mCherry::RAB-7 and increases their colocalization. White arrowheads marking GFP::RAB-5 and mCherry::RAB-7 positive structures, respectively in the individual channels. Yellow arrowheads marking colocalization events in the merges (Signals: RAB-5 > green and RAB-7 > magenta). (**B**) Schematic representation of the endosomal RAB-5/RAB-7 balance in a *C. elegans* intestinal cell in WT background. Effects of UBQ reduction or *usp-50(RNAi)* on this balance are shown via black arrows. (**C**) The RNAi mediated UBQ level abatement causes the formation of smaller RFP::RAB-5 aggregates and leads to less prominent GFP::UBQ structures. *usp-50(RNAi)* causes the formation of large GFP::UBQ structures and aggregates which are often also RFP::RAB-5 positive and reduces the GFP::UBQ signal in the nucleus. White arrowheads marking GFP::UBQ and RFP::RAB-5 positive structures, respectively in the individual channels. Yellow arrowheads marking colocalization events in the merges (Signals: UBQ > green and RAB-5 > magenta). (**D**) Schematic representation of the UBQ distribution in an intestinal *C. elegans* cell expressing marked UBQ and RAB-5 in WT background. The effect of the *usp-50* knockdown on this equilibrium is shown via a black arrow. Data information: Merges were individually adjusted in all panels. Representative pictures with close ups (white box) on the right are shown for each experiment (scale bars: 10 µm (main pictures) and 1 µm (close ups) in (**A** and **C**); $n = 3$ independent experiments. Unprocessed images are available as source data. Source data are available online for this figure.

 © The Author(s)
